# Fast calculation of scattering patterns using hypergeometric function algorithms

**DOI:** 10.1038/s41598-023-27558-8

**Published:** 2023-01-15

**Authors:** Michael Wagener, Stephan Förster

**Affiliations:** 1 Jülich Centre for Neutron Science (JCNS-1/IBI-8), Forschungszentrum Jülich, 52425 Jülich, Germany; 2 Institute of Physical Chemistry, RWTH Aachen University, 52074 Aachen, Germany

**Keywords:** Computational science, Characterization and analytical techniques

## Abstract

The scattering of light, X-rays, electrons or neutrons by matter is used widespread for structural characterization from atomic to macroscopic length scales. With the advent of high-brilliance beam sources and the development fast, large area pixelated detectors, scattering patterns are now acquired at unprecedented frame rates and frame sizes. The slow analysis of these scattering patterns has evolved into a severe bottleneck retarding scientific insight. Here we introduce an algorithm based on the use of hypergeometric functions providing gains in computational speed of up to 10^5^ compared to present numerical integration algorithms. Hypergeometric functions provide analytical descriptions of geometrical shapes, can be rapidly computed as series and asymptotic expansions, and can be efficiently implemented in GPUs. The algorithm provides the necessary computational speed to calculate scattering patterns on timescales required for real-time experiment feedback, the analysis of large volumes of scattering data, and for the generation of training data sets for machine learning.

The scattering of light, X-rays, electrons or neutrons by matter is used widespread for materials structure characterization from atomic to macroscopic length scales^1,2^. To obtain multi-scale structural information requires for scattering experiments to acquire scattering patterns over large detector areas. Modern pixelated detectors therefore cover increasingly large areas with corresponding pixel numbers now exceeding 10^7^. Concomitantly, high-intensity beam sources such as lasers, fourth generation synchrotron sources, neutron spallation sources, aberration corrected electron microscopes and metal jet X-ray sources have become widely available. The combination of high-intensity beams with fast large area detectors now enable in situ and *operando* experiments elucidating rapid and complex structural changes, thereby gaining key insights into materials structural evolution, function, and performance. Commonly investigated materials and devices include high performance metal alloys, fibers, batteries, fuel cells and solar cells, nanomaterials, composites, polymers, colloids, membranes, as well as implants, drug delivery formulations and biological tissue.

This evolution has led to an unprecedented increase in the acquisition rate and volume of 1D and 2D scattering data such that the time needed for data analysis has become a major bottleneck in the process to gain materials insight. Therefore, software for scattering data reduction and analysis is continuously improved by introducing more efficient data analysis pipelines^3^, by GPU acceleration^4^, and the use of machine learning algorithms^5^. Yet, the computational speed for data analysis has not increased at a rate comparable to the increase of current data acquisition rates.

Multi-length scale analysis of scattering data of materials generally proceeds by modeling sub-structures with geometrical objects, which are linked and assembled into compound objects that are spatially distributed with a degree of positional and orientational order. Common geometrical objects include spheres, ellipsoids, parallelepipeds, cylinders, disks, polyhedrons, or flexible tubes or membranes whose surfaces can be mathematically described in closed analytical forms. This geometrical approach to model complex structures is also commonly used in computer simulations and in ray-tracing graphics algorithms.

The calculation of scattering patterns involves the computation of the Fourier transform of the assembled object structure, and the subsequent averaging over size, orientational and positional distributions of the objects characterizing the real material under investigation^6^. The calculation requires several numerical integrations to compute the Fourier transforms and to average over the distribution functions. This computation is time consuming and constitutes the bottleneck of the data analysis step. Therefore, there has been a long history of new important mathematical methods for the efficient computation and analysis of scattering functions^7–11^.

To reduce computation time, whenever possible, 2D data sets are azimuthally averaged to obtain 1D data sets with the number $N$ of data points reduced to $\sqrt{N}$. Yet, for the analysis of large data sets or for the generation of synthetic data sets to train neural networks, current algorithms are prohibitively slow^12^. In addition, the analysis of the full 2D-scattering pattern is required for a large class of synthetic and biological materials consisting of anisotropic structures or thin films. The fast computation of scattering data for all these cases is beyond the scope of current algorithms and software packages.

Here, we present an algorithm that is based on the use of hypergeometric functions to rapidly compute 1D- and 2D-scattering data. Hypergeometric functions provide a simple mathematical description of geometrical objects, have analytical Fourier transforms, and can be rapidly computed via series and asymptotic expansions with recursive coefficients. Compared to numerical integration schemes we observe gains in computation speed of > 10^5^. The algorithm can be efficiently parallelized and implemented into GPUs for further acceleration. This enables the computation of 2D scattering patterns at > 1 fps even for current 4k pixel detectors.

## Results

### Calculation of scattering patterns

**Figure 1:**
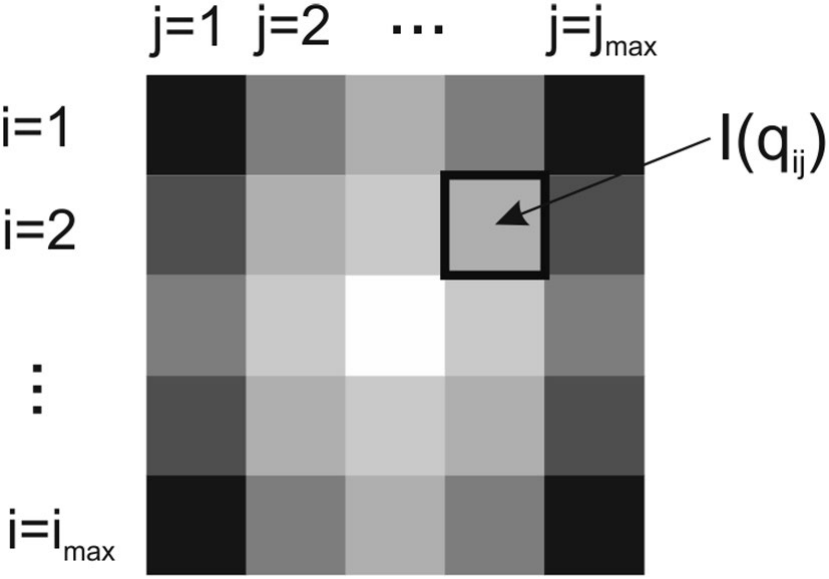
Pixel array with rows *i* and columns *j*, and the scattering intensity $I({{\varvec{q}}}_{{\varvec{i}}{\varvec{j}}})$. Modern 4 k pixel detectors have more than 16 million pixels for which scattering intensities need to be computed and compared to experimental data. It is a considerable computational challenge for current numerical integration algorithms.

The calculation of scattering patterns on pixel detectors requires the computation of the scattered intensity $I({{\varvec{q}}}_{{\varvec{i}}{\varvec{j}}})$ for each pixel. Modern 4k detectors have more than 16 million pixels. Figure 1 schematically illustrates an array of pixels $(i,j)$, for each of which the scattering intensity needs to be computed. Mathematically, the position $({x}_{ij},{y}_{ij})$ of each pixel corresponds to certain components of the scattering vector ${{\varvec{q}}}_{{\varvec{i}}{\varvec{j}}}$**,** which is related to experimental parameters including the wavelength $\lambda$ of the incoming beam, the sample-detector distance ${d}_{det}$, and the angle ${\vartheta }_{ij}$ enclosed by the scattered beam and the incoming beam. The scattering vector ${{\varvec{q}}}_{{\varvec{i}}{\varvec{j}}}$ is given by

1 $${{\varvec{q}}}_{{\varvec{i}}{\varvec{j}}}=\left(\begin{array}{c}{q}_{x,ij}\\ {q}_{y,ij}\\ {q}_{z,ij}\end{array}\right)=\frac{2\pi }{\lambda }\left(\begin{array}{c}\left({x}_{ij}/{d}_{det}\right)\mathrm{sin}{\vartheta }_{ij}\\ \left({y}_{ij}/{d}_{det}\right)\mathrm{sin}{\vartheta }_{ij}\\ 1-\mathrm{cos}{\vartheta }_{ij}\end{array}\right)\approx \frac{2\pi }{\lambda {d}_{det}}\left(\begin{array}{c}{x}_{ij}\\ {y}_{ij}\\ 0\end{array}\right)$$

The approximation on the right-hand side is the basis for small-angle scattering experiments.

For materials consisting of or modelled with assemblies of geometrical objects, the calculation of the scattered intensity $I({{\varvec{q}}}_{{\varvec{i}}{\varvec{j}}})$ proceeds via the calculation of their Fourier transform or scattering amplitude $F({{\varvec{q}}}_{{\varvec{i}}{\varvec{j}}})$**,** the formfactor $P({{\varvec{q}}}_{{\varvec{i}}{\varvec{j}}})$, and the lattice factor $Z({{\varvec{q}}}_{{\varvec{i}}{\varvec{j}}})$ describing their spatial assembly. $F({{\varvec{q}}}_{{\varvec{i}}{\varvec{j}}})$ is obtained by integrating the density of the object, $\rho \left({\varvec{r}}\right)$**,** multiplied with the phase factor ${e}^{i{{\varvec{q}}}_{{\varvec{i}}{\varvec{j}}}{\varvec{r}}}$ over the volume *V* of the object

2 $$F({{\varvec{q}}}_{{\varvec{i}}{\varvec{j}}})=\frac{1}{V}\iiint \rho \left({\varvec{r}}\right){e}^{i{{\varvec{q}}}_{{\varvec{i}}{\varvec{j}}}{\varvec{r}}}d{\varvec{r}}$$

Subsequently, the formfactor $P({{\varvec{q}}}_{{\varvec{i}}{\varvec{j}}})$ is obtained as the absolute square of the scattering amplitude $F({{\varvec{q}}}_{{\varvec{i}}{\varvec{j}}})$. In a third step, the formfactor is averaged over the size distribution $h(R)$ and the orientational distribution $h(\vartheta ,\varphi )$ of the ensemble of objects

3 $$\langle P({{\varvec{q}}}_{{\varvec{i}}{\varvec{j}}})\rangle =\frac{1}{2\pi }\iiint {\left|F\left({\varvec{q}}\right)\right|}^{2}h(R)h(\vartheta ,\varphi )dR\mathrm{sin}\vartheta d\vartheta d\varphi$$

If the objects are characterized by more than one characteristic dimension, e.g. for anisometric objects such as ellipsoids, cylinders, or parallelepipeds, the size distribution for each additional dimension needs to be taken into account. For common particle shapes, the number of required numerical integrations to calculate $\langle P({{\varvec{q}}}_{{\varvec{i}}{\varvec{j}}})\rangle$ increases from spheres (1), biaxial ellipsoids (3), cubes (3), cylinders (3), disks (3), super-ellipsoids (4), triaxial ellipsoids (5), parallelepipeds (5), octahedrons (5) to super-balls (5–7). This makes calculations of scattering patterns for modern large area pixel detectors prohibitively slow. In addition, the three-dimensional assembly of these objects represented by the lattice factor $Z({{\varvec{q}}}_{{\varvec{i}}{\varvec{j}}})$ needs to be included, at least at the level of the decoupling approximation^13,14^.

4 $${I({{\varvec{q}}}_{{\varvec{i}}{\varvec{j}}}) =\left(\Delta b\right)}^{2}{\rho }_{N}\langle P\left({{\varvec{q}}}_{{\varvec{i}}{\varvec{j}}}\right)\rangle \left[1+\frac{{\langle F\left({{\varvec{q}}}_{{\varvec{i}}{\varvec{j}}}\right)\rangle }^{2}}{\langle P\left({{\varvec{q}}}_{{\varvec{i}}{\varvec{j}}}\right)\rangle }\left(\langle Z\left({{\varvec{q}}}_{{\varvec{i}}{\varvec{j}}}\right)\rangle -1\right)G\left({{\varvec{q}}}_{{\varvec{i}}{\varvec{j}}}\right)\right]$$

where $\Delta b$ is a sample specific contrast factor, ${\rho }_{N}$ the particle number density, and $G(q)$ the Debye–Waller factor, requiring the calculation of a large number of Bragg-peak positions.

The main challenges for the calculation and analysis of large 2D scattering patterns and derived 1D-data sets are therefore (i) the large number of numerical integrations to compute $\langle P({{\varvec{q}}}_{{\varvec{i}}{\varvec{j}}})\rangle$, and (ii) the large computational effort to calculate $\langle Z({{\varvec{q}}}_{{\varvec{i}}{\varvec{j}}})\rangle$. The aim of the presented algorithm is.

1. to develop a method to solve as many integrations as possible analytically to perform the computation of $I({{\varvec{q}}}_{{\varvec{i}}{\varvec{j}}})$ on simple functions, and
2. to factorize $\langle P({{\varvec{q}}}_{{\varvec{i}}{\varvec{j}}})\rangle$ and $\langle Z({{\varvec{q}}}_{{\varvec{i}}{\varvec{j}}})\rangle$ into *q*-independent parts with coefficients ${c}_{n}$ which are the same for every pixel, and a remaining *q*-dependent part ${f}_{n}(q)$ which is simple and can be rapidly computed on every pixel by multiplication with the pre-calculated set ${c}_{n}$, thereby in addition allowing an efficient implementation in parallel computing algorithms and GPUs.

We show that the use of hypergeometric functions provides the needed methodology. Hypergeometric functions are usually used to compute special functions and have so far only been considered for the calculation of the Fourier transform of specific polymer core/shell structures, because they provided a closed analytical solution for shells with algebraic density profiles^15^. The benefit in the calculation and use of their *q*-independent series coefficients for the rapid computation of scattering patterns has not yet been considered.

### Algorithm

**Figure 2:**
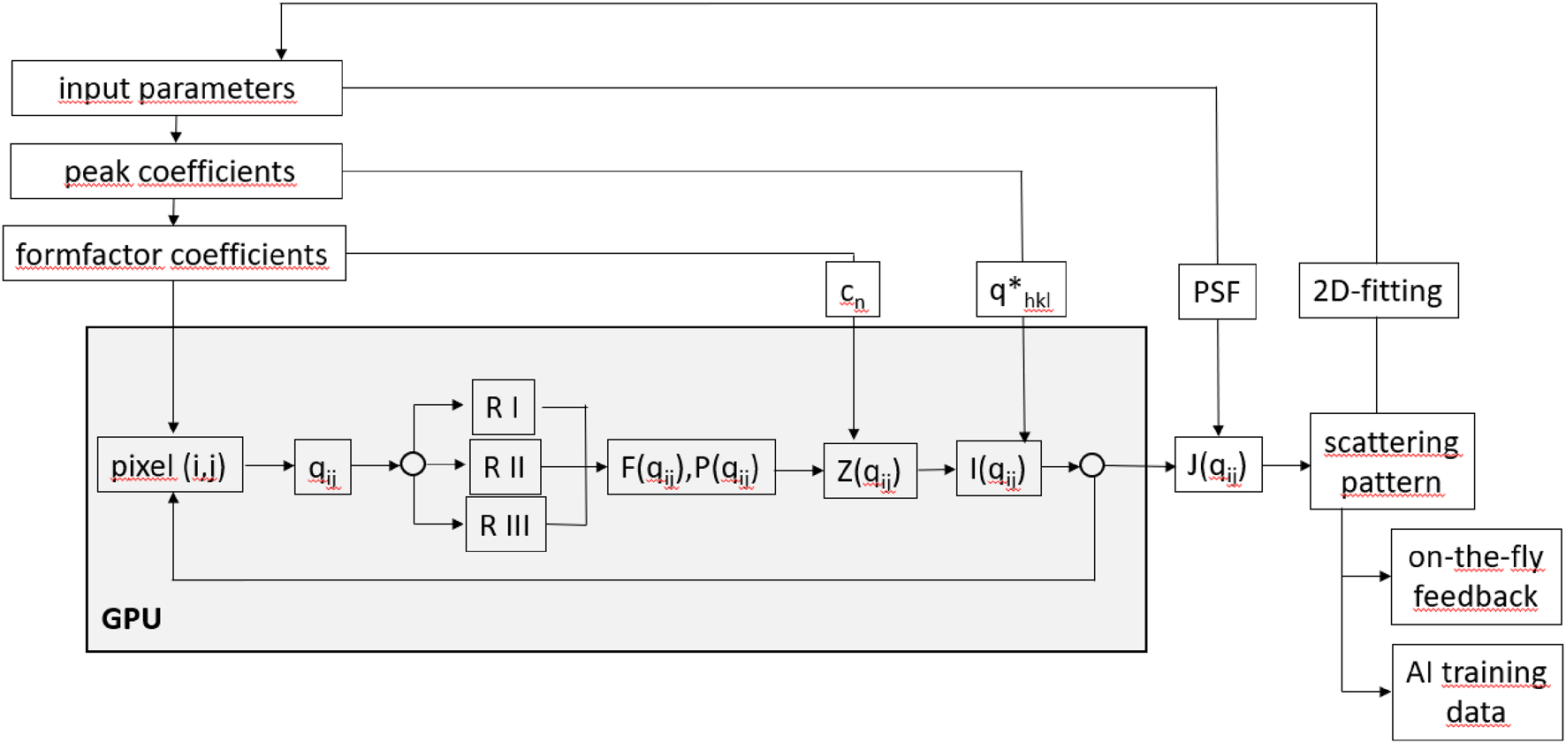
Algorithm to compute 2D-scattering patterns. Using the provided input parameters, the algorithm pre-calculates the coefficients for the reciprocal space vectors ${{\varvec{q}}}_{hkl}^{*}$ and the coefficients ${c}_{n}$. Within the $(i,j)$-loop for all pixels the scattering vector ${{\varvec{q}}}_{ij}$, and from this the scattering amplitude $F({{\varvec{q}}}_{ij})$ and the formfactor $P({{\varvec{q}}}_{ij})$ are calculated using the pre-calculated coefficients ${c}_{n}$. Subsequently, the pre-calculated coefficients for the reciprocal space vectors are used to calculate the lattice factor $Z({{\varvec{q}}}_{ij})$ to finally obtain the scattered intensity $I({{\varvec{q}}}_{ij})$. Since the calculations in the $(i,j)$-loop are mutually independent, they can be efficiently parallelized employing GPUs. The resulting scattering pattern can be convoluted with the beam or point spread function (PSF) to obtain $J({{\varvec{q}}}_{ij})$ for direct comparison to experiments. For 1D-data the i-loop to calculate $I({q}_{i})$ is divided into j blocks.

The corresponding algorithm is shown in Fig. 2. With the input parameters that describe the objects and their spatial assembly, the coefficients needed for the reciprocal space vectors ${{\varvec{q}}}_{hkl}^{*}$ together with the *q*-independent coefficients ${c}_{n}$ are calculated. The pixel intensities $I({{\varvec{q}}}_{{\varvec{i}}{\varvec{j}}})$ are calculated within the (i,j)-loop over all detector pixel rows and columns as in Fig. 1. For 1D-data the i-loop to calculate $I({q}_{i})$ is divided into j blocks.

The algorithm requires the pre-calculation of the coefficients ${c}_{n}$ of the series and the asymptotic expansions, which is not necessary for numerical integration schemes. We find the computational cost for the pre-calculations to be very small. It is overcompensated by an acceleration factor of up to 5^.^10^5^ to compute the scattering pattern in the subsequent (i,j)-loop.

### Hypergeometric functions

Hypergeometric functions have properties that are ideal for the required algorithm. The integral for the calculation of the Fourier transform (Eq. (2)) can be expressed in terms of the hypergeometric function ${}_{0}{F}_{1}\left(z\right)$ as^15^

5 $$F\left({\varvec{q}}\right)=\frac{1}{V}{\int }_{0}^{R}{}_{0}{F}_{1}\left(\frac{d}{2};-\frac{{q}^{2}{r}^{2}}{4}\right)\frac{2{\pi }^\frac{d}{2}}{\Gamma \left[\frac{d}{2}\right]}{r}^{d-1}dr={}_{0}{F}_{1}\left(\frac{d+2}{2};-\frac{{q}^{2}{R}^{2}}{4}\right)$$

with the volume $$V={\int }_{0}^{R}\frac{2{\pi }^\frac{d}{2}{r}^{2}}{\Gamma \left[\frac{d}{2}\right]}{r}^{d-1}dr$$. *R* is the size, *r* the radial distance, and *d* the dimensionality of the object. Equation (5) applies to objects that are rotationally symmetric in three dimensions ($$d=3$$, spheres), in two dimensions ($$d=2$$, cylinders) and formally in one dimension ($$d=1$$, platelets, lamellae). By rescaling, these cases can be extended to include biaxial and triaxial ellipsoids, octahedrons and superballs ($$d=3)$$, superellipsoids, dumbbells and lenses ($$d=2)$$, cubes and parallelepipeds ($$d=\mathrm{1,2},3)$$ as well as polymer chains and flexible tubes ($$d=1-2)$$. Thus, Eq. (5) applies to large class of objects that are relevant for the modeling of materials structures. The integral can be extended over the volume of compound objects, such that a large class of further, more complex object structures can be analytically described.

Hypergeometric functions can be calculated via series and asymptotic expansions^16,17^. A detailed derivation of the scattering amplitudes and formfactors is outlined in the Supporting Information (SI Sect. 1). We here provide the two main results:

- For small arguments *z*, the hypergeometric functions can be computed via a series expansion, which for the case of ${}_{0}{F}_{1}\left(z\right)$ is given by
  6 $${}_{0}{F}_{1}\left(\frac{d+2}{2};-\frac{{q}^{2}{R}^{2}}{4}\right)=\sum_{n=0}^{\infty }{\frac{1}{{\left(\frac{d+2}{2}\right)}_{n}n!}\left(-\frac{{q}^{2}{R}^{2}}{4}\right)}^{n}$$

where ${\left(u\right)}_{n}=\frac{\Gamma \left[u+n\right]}{\Gamma \left[i\right]}$ is the Pochhammer factorial.

(b) For large arguments $z$*,* the hypergeometric functions can be computed via an asymptotic expansion
  7 $${}_{0}{F}_{1}\left(\frac{d+2}{2};-\frac{{q}^{2}{R}^{2}}{4}\right)=\frac{\Gamma \left(\frac{d+2}{2}\right)}{{\left(\pi \right)}^\frac{1}{2}}{\left(\frac{qR}{2}\right)}^{\nu }\sum_{k=0}^{\infty }{c}_{k}\mathrm{cos}\left(qR+\frac{\pi \left(\nu -k\right)}{2}\right){\left(qR\right)}^{-k}$$

with $\nu =-\frac{d+2}{2}+\frac{1}{2}$. For the required numerical accuracy, it is sufficient to use just the first two terms of the expansion (7). When choosing common log-normal distributions, the averages over the terms of the series expansions $\langle {z}^{2n}\rangle$ (Eq. 6) and asymptotic expansions $\langle \frac{\mathrm{cos}(z)}{{z}^{k}}\rangle$ (Eq. 7) are simple linear and trigonometric functions. These are summarized in the Supporting Information (SI Sect. 2). The series expansion Eq. (6) and the asymptotic expansion Eq. (7) overlap and are the basis for the calculation of the *q*-independent coefficients ${c}_{n}$.

### Coefficients and formfactors in the isotropic case

We here illustrate the derivation of the coefficients ${c}_{n}$ with the simple example of a spherical object. In the Supporting Information we provide the coefficients for a comprehensive collection of geometrical objects including biaxial ellipsoids, triaxial ellipsoids, cylinders with circular and elliptical cross-sections, disks, cubes, parallelepipeds, superballs, super-ellipsoids, dumbbells, lenses and excluded volume polymer chains (SI Sect. 4) for a broad range of applications.

For spheres ($d=3$), the formfactor is obtained from $P(q)={F}^{2}(q)$ as

8 $$P\left(q\right)={{}_{0}F}_{1}^{2}(q)=\sum_{n=0}^{\infty }{\left(-\frac{{q}^{2}{R}^{2}}{4}\right)}^{n}\sum_{m=0}^{n}\frac{1}{{\left(\frac{5}{2}\right)}_{n-m}{\left(\frac{5}{2}\right)}_{m}\left(n-m\right)!m!}$$

In the Supporting Information (Eq. S.1.3.2) we show that for all dimensions *d* the double sum can be recast into a single sum which accelerates the computation significantly

9 $$\langle P\left(q\right)\rangle =\sum_{n=0}^{\infty }\frac{6\cdot {4}^{n}}{\left(\mathrm{n}+3\right)\left(\mathrm{n}+2\right)}\frac{1}{{\left(\frac{5}{2}\right)}_{n}n!}{\left(-\frac{{q}^{2}{R}^{2}}{4}\right)}^{n}$$

The average over the size distribution, characterized by a polydispersity parameter z, yields the series

10 $$\begin{aligned} P\left( q \right) = & \mathop \sum \limits_{n = 0}^{\infty } \frac{{6 \cdot 4^{n} }}{{\left( {{\text{n}} + 3} \right)\left( {{\text{n}} + 2} \right)}}\frac{{\left( {z + 1} \right)_{2n} }}{{\left( \frac{5}{2} \right)_{n} n!}}\left( { - \frac{{q^{2} R^{2} }}{{4\left( {z + 1} \right)^{2} }}} \right)^{n} = \mathop \sum \limits_{n = 0}^{\infty } c_{n} q^{2n} \\ c_{n} = & \frac{{6 \cdot 4^{n} \left( {z + 1} \right)_{2n} }}{{\left( {{\text{n}} + 3} \right)\left( {{\text{n}} + 2} \right)}}\left( { - \frac{{R^{2} }}{{4\left( {z + 1} \right)^{2} }}} \right)^{n} \\ \end{aligned}$$

The coefficients ${c}_{n}$ can be efficiently calculated via recursion relations for the powers, factorials, Gamma functions, Pochhammer factorials and binomial coefficients, as summarized in the Supporting Information (SI Sect. 10). Because of the recursion relations, the calculation of the coefficients is fast, can be encoded with few lines of source code, and without the necessity to compute special functions such as Bessel and Gamma functions or Pochhammer factorials.

The series expansion (Eq. (6)) converges for values of $qR<1-10$ (Regime I), depending on the polydispersity *z*. For $qR>1$ (Regime II) we use the asymptotic expansion (Eq. (7)) for spheres ($d=3$) which is

11 $$\langle P\left(q\right)\rangle =9\left[\langle \frac{{\left(\mathrm{sin}\left(qR\right)\right)}^{2}}{{(qR)}^{6}}\rangle -2\langle \frac{\mathrm{sin}\left(qR\right)\mathrm{cos}\left(qR\right)}{{(qR)}^{5}}\rangle +\langle \frac{{\left(\mathrm{cos}\left(qR\right)\right)}^{2}}{{(qR)}^{4}}\rangle \right]$$

The averages of the trigonometric functions are given in terms of simple cosine and arctan functions and are all summarized in the Supporting Information (SI Sect. 2).

For $qR\gg 1$ (Regime III) we only need to use the leading cosine term of the asymptotic expansion to derive the non-oscillating part of the asymptote, which is identical to the Porod-${q}^{-4}$-asymptote

12 $$\underset{q\to \infty }{\mathrm{lim}}\langle P\left(q\right)\rangle =\frac{9{(z+1)}^{4}}{2\mathrm{z}(\mathrm{z}-1)(\mathrm{z}-2)(\mathrm{z}-3)}\frac{1}{{(qR)}^{4}}$$

These three regimes are also indicated in the schematic algorithm description (RI, RII, RIII) in Fig. 2.

The consideration of size distributions has two advantages: (i) it provides a realistic description of materials structures, and (ii) at the same time mathematically leads to non-oscillating single-term asymptotes of the formfactor $P\left(q\right)$ in the hiqh-*q* range (Regime III regime), which can represent a significant part of the scattering patterns recorded by large area detectors. The calculation of the Porod asymptotes of anisometric objects^18–20^ has been rarely considered in literature, but is important for the algorithm and therefore outlined in the Supporting Information (SI Sect. 5).

**Figure 3:**
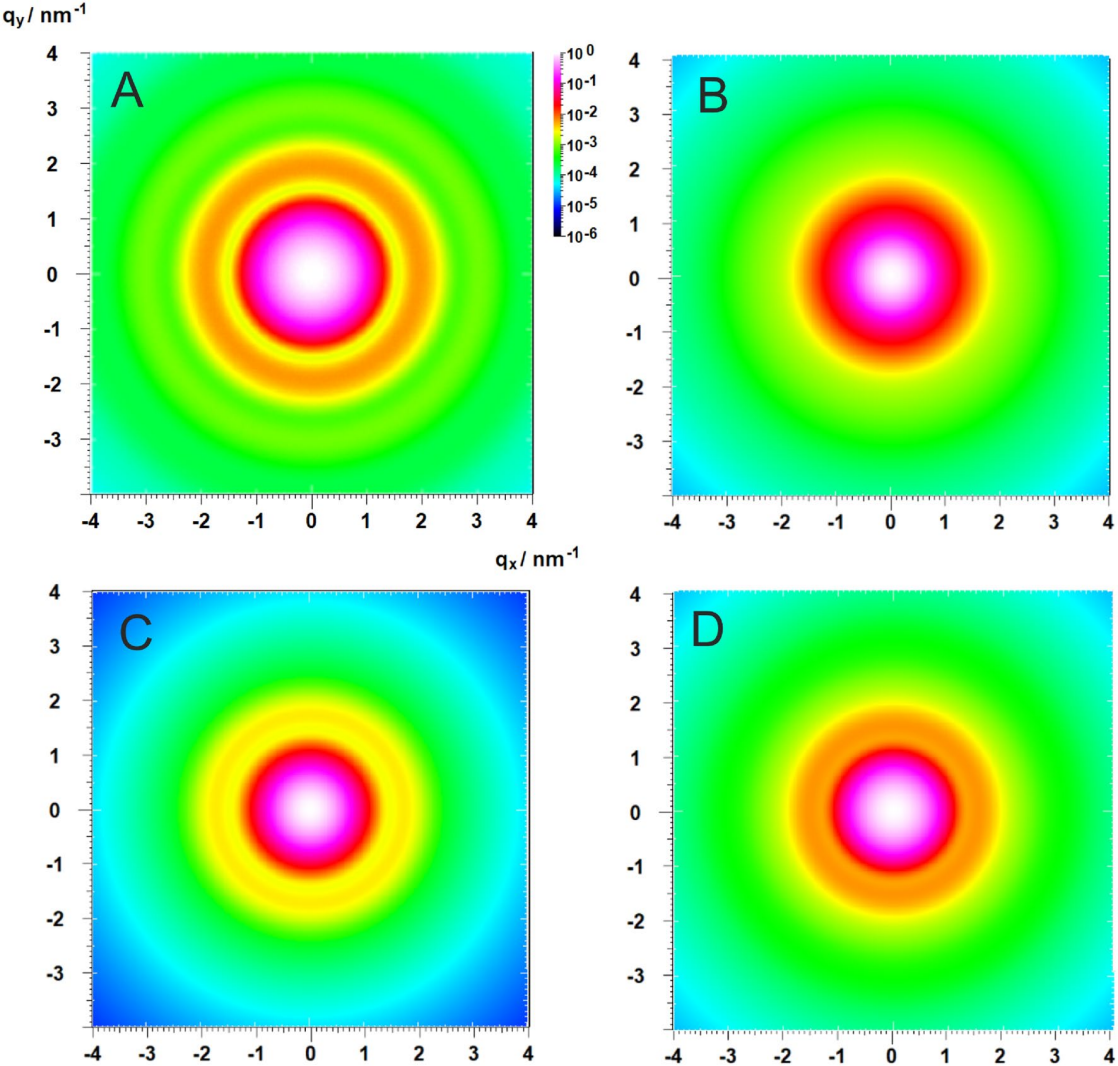
Plots of calculated formfactors of polydisperse geometrical objects to show the seamless overlap for the calculations in the three regimes I, II, III for spheres ((**A**) *R* = 3 nm) and triaxial ellipsoids ((**B**) *a* = 2 nm, *b* = 4 nm, *c* = 6 nm), and direct overlap of regimes I and III for superellipsoids ((**C**) *R* = 3 nm, *L* = 5 nm, *k* = 3.4) and superballs ((**D**) *a* = 2 nm, *b* = 2.5 nm, *c* = 3 nm, *k* = 5.5). The latter two have been chosen to demonstrate the rapid calculation of the formfactor of complex-shaped objects. The relative standard deviation of the particle sizes is σ = 0.08 in all cases. The CPU-times are provided and benchmarked in Fig. 6.

The computed scattering patterns in Fig. 3 show the seamless overlap of the series expansions (Regime I), asymptotic expansions (Regime II) and the Porod-Regime (Regime III) such that the formfactors can be computed rapidly over the complete *q*-range. The *q*-values for the I-II and II-III transitions can be pre-calculated during the calculations of the series cofficients ${c}_{n}$ (see Supporting Information (SI Sect. 3).

### Coefficients and formfactors in the anisotropic case

A further advantage of the rapid calculations using series and asymptotic expansions is in their application to compute the scattering patterns of oriented assemblies of isometric and anisometric objects. This requires orientational averaging, usually performed by two additional numerical integrations which is very time-consuming and therefore rarely done.

For the oriented case we need to specify the main axis ***L*** of the object with respect to a director ***D***. The particles may have a deviation angle $\delta$ with respect to the director ***D***, with a distribution function $h(\delta )$. We obtain simple expressions for the phases ***qL***, which can be directly implemented in the derived series and asymptotic expansions for rapid calculations. All details of the derivation are provided in the Supporting Information (SI Sect. 7).

As an illustration, we consider the case of an ensemble of cylindrical objects oriented along the x-axis with a deviation angle $\delta$. For the phase ${\varvec{q}}{\varvec{L}}$ we obtain the expression

13 $${\varvec{q}}{\varvec{L}}\left(\delta ,\chi \right)=L\left({q}_{x}\mathrm{cos}\left(\delta \right)-{q}_{y}\mathrm{sin}\left(\chi \right)\mathrm{sin}\left(\delta \right)\right)$$

where $\chi$ is the azimuthal angle on a cone with opening angle $\delta$. For the series and asymptotic expansions, we need the averaged phase term which is obtained as

14 $$\langle {\left({\varvec{q}}{\varvec{L}}\right)}^{2n}\rangle =\frac{\left(2n\right)!{{\varvec{L}}}^{2n}}{{4}^{n}}\sum_{l=0}^{n}\frac{{4}^{l}}{(2l)!{\left(\left(\mathrm{n}-\mathrm{l}\right)!\right)}^{2}}{\left({q}_{x}^{2}\right)}^{l}{\left({q}_{y}^{2}\right)}^{n-l}{H}_{2l,2n-2l}$$

with

$${H}_{2l,2n-2l}=\frac{\underset{0}{\overset{\pi /2}{\int }}{\left(\mathrm{cos}\left(\delta \right)\right)}^{2l}{\left(\mathrm{sin}\left(\delta \right)\right)}^{2n-2l}h\left(\delta \right)\mathrm{sin}\left(\delta \right)d\delta }{\underset{0}{\overset{\pi /2}{\int }}h\left(\delta \right)\mathrm{sin}\left(\delta \right)d\delta }$$

The integrals ${H}_{2l,2n-2l}$ need to be integrated numerically over the orientational distribution function $h\left(\delta \right)$, but are *q*-independent and therefore part of the coefficients ${c}_{n}$. The integral over $\frac{3{\mathrm{cos}}^{2}\left(\delta \right)-1}{2}$ directly provides the orientational order parameter *S*.

The phase term can be inserted into the cylinder formfactor series expansion as

15 $$\langle {P}_{\parallel }\left({\varvec{q}}\right)\rangle =\sum_{n=0}^{\infty }\frac{{4}^{n}}{(\mathrm{n}+1)}\frac{{\left(z+1\right)}_{2n}}{{\left(\frac{3}{2}\right)}_{n}n!}{\left(-\frac{{\left({\varvec{q}}{\varvec{L}}\right)}^{2}}{4{(z+1)}^{2}}\right)}^{n}$$

For rapid calculations the *q*-independent coefficients $${a}_{n}$$ and $${b}_{l,n}$$ are pre-calculated, such that the series can be quickly evaluated for each pixel ($${q}_{x},{q}_{y})$$

16 $$\langle {P}_{\parallel }\left({q}_{x},{q}_{y}\right)\rangle =\sum_{n=0}^{\infty }{a}_{n}\sum_{l=0}^{n}{b}_{l,n}{\left({q}_{x}^{2}\right)}^{l}{\left({q}_{y}^{2}\right)}^{n-l}$$

**Figure 4:**
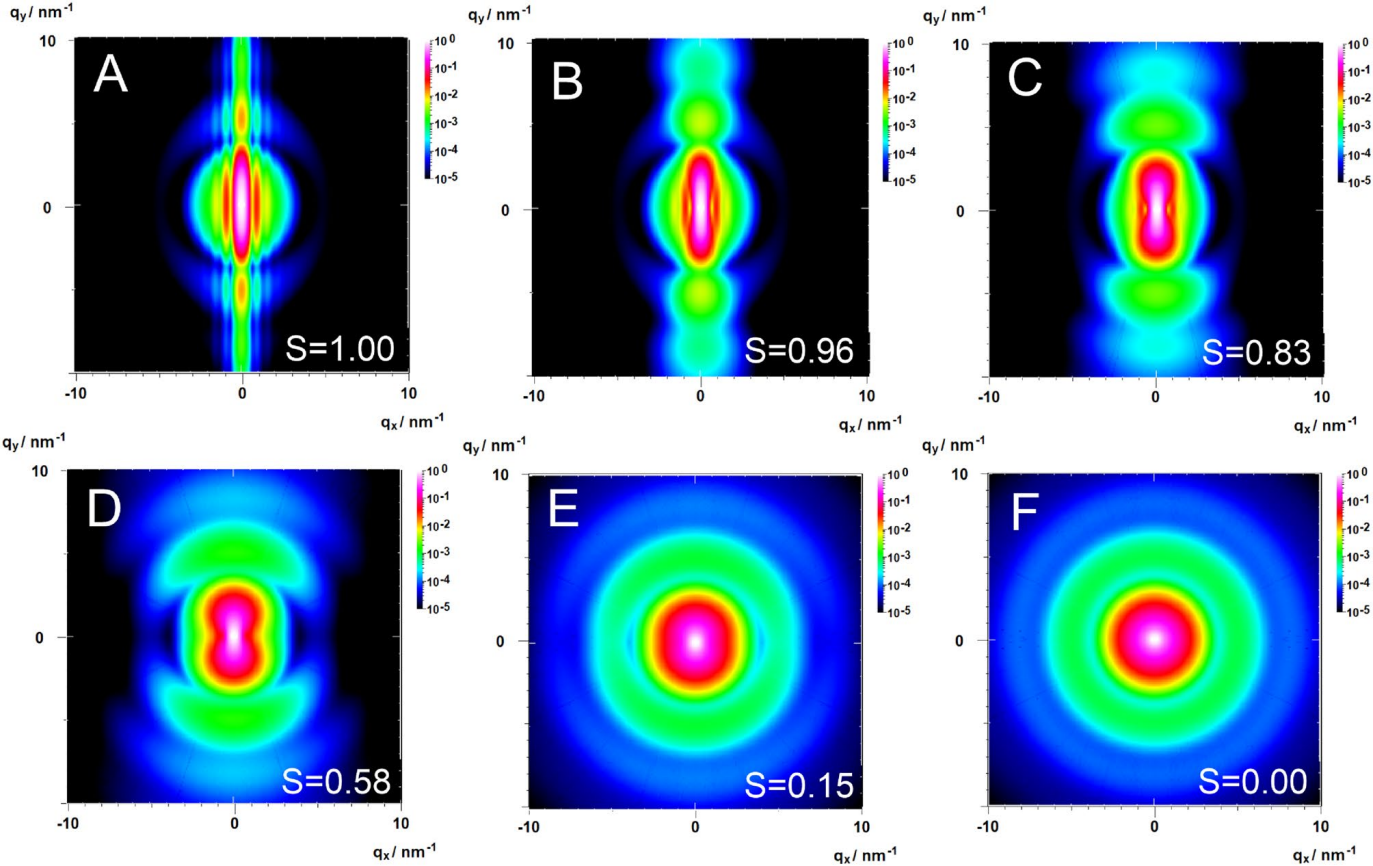
Calculated 2D-scattering patterns of oriented polydisperse cylinders aligned parallel to the x-axis with an orientational distribution varying between uniform alignment with order parameter S = 1.0 (**A**), S = 0.96 (**B**), S = 0.83 (**C**), S = 0.58 (**D**), S = 0.15 (**E**) to an isotropic orientational distribution with S = 0 (**F**) using Eqs. (14–16).

Similarly, the phase term $\langle {\left({\varvec{q}}{\varvec{L}}\right)}^{2n}\rangle$ can be inserted into the asymptotic expansions in Regime II and III (SI Sect. 7). The example in Fig. 4 demonstrates the seamless overlap of the series and asymptotic expansions in the two-dimensional anisotropic case for cylinders with high (A) to low (F) orientational order.

Similar expressions can be derived for uniaxial orientational distributions for disks, parallelepipeds, biaxial and triaxial ellipsoids and are provided in the Supporting Information (SI Sect. 7).

### Ordered and periodic structures

Most synthetic and biological materials consist of particles or objects that are assembled with varying degrees of order. Therefore, it is important to include crystallographic calculations in the algorithm. We will show that also these contributions can be factorized into a *q*-independent part that can pre-calculated and provided to the (i,j)-loop, where it is multiplied with a *q*-dependent part. For 1D-, 2D- and 3D-assemblies the calculations require to evaluate single, double or triple sums over all non-zero (hkl) Miller indices, such that pre-calculation schemes are very effective.

For the calculations, we need to specify the (uvw)-direction within the unit cell, which is parallel to the probe beam direction, i.e.${{\varvec{r}}}_{{\varvec{u}}{\varvec{v}}{\varvec{w}}}\boldsymbol{ }||\boldsymbol{ }{\varvec{n}}$. The unit cell is defined by the three edge lengths and the enclosed angles (a,b,c; α, β, γ). Then the direction vector is given as ${{\varvec{r}}}_{{\varvec{u}}{\varvec{v}}{\varvec{w}}}=u{{\varvec{a}}}_{{\varvec{A}}}+v{{\varvec{b}}}_{{\varvec{A}}}+w{{\varvec{c}}}_{{\varvec{A}}}$ in the unit cell coordinate system ${\varvec{A}}$^21^. We need to transform the unit cell base vectors ${({\varvec{a}}}_{{\varvec{A}}},{{\varvec{b}}}_{{\varvec{A}}},{{\varvec{c}}}_{{\varvec{A}}})$ to the orthonormal Carthesian lab-base coordinate system ${\varvec{E}}$ to obtain the set of base vectors ${({\varvec{a}}}_{{\varvec{E}}},{{\varvec{b}}}_{{\varvec{E}}},{{\varvec{c}}}_{{\varvec{E}}})$ using a transformation matrix **E.** These base vectors are then rotated to align with the (uvw)-direction parallel to the probe beam, i.e. ${{\varvec{r}}}_{{\varvec{u}}{\varvec{v}}{\varvec{w}}}\boldsymbol{ }||\boldsymbol{ }{\varvec{n}}$, using a rotation matrix ${\varvec{R}}$ to obtain the rotated base vectors ${({\varvec{a}}}_{{\varvec{E}}{\varvec{r}}},{{\varvec{b}}}_{{\varvec{E}}{\varvec{r}}},{{\varvec{c}}}_{{\varvec{E}}{\varvec{r}}})$. These vectors are subsequently transformed to the reciprocal space vectors $({{\varvec{a}}}^{*},{{\varvec{b}}}^{*},{{\varvec{c}}}^{*})$ using the metric matrix ${\varvec{G}}$. These three transformations can be compactly expressed as

17 $$\left(\begin{array}{c}{{\varvec{a}}}^{*}\\ {{\varvec{b}}}^{*}\\ {{\varvec{c}}}^{*}\end{array}\right)={\varvec{G}}={({\varvec{R}}{\varvec{M}}{{\varvec{a}}}_{{\varvec{A}}},{\varvec{R}}{\varvec{M}}{{\varvec{b}}}_{{\varvec{A}}},{\varvec{R}}{\varvec{M}}{{\varvec{b}}}_{{\varvec{A}}})}^{\boldsymbol{ }-1}$$

such that in the (i,j)-loop and the (h,k,l)-sums the respective reciprocal space vectors ${{\varvec{q}}}_{hkl}^{*}$ can be calculated from the precalculated reciprocal space vectors as ${{\varvec{q}}}_{hkl}^{*}=h{{\varvec{a}}}^{\boldsymbol{*}}+k{{\varvec{b}}}^{\boldsymbol{*}}+l{{\varvec{c}}}^{\boldsymbol{*}}$.

The matrices ${\varvec{M}},\boldsymbol{ }{\varvec{R}},\boldsymbol{ }{\varvec{G}}$ are derived and provided in the Supporting Information (SI Sect. 8). With the vector ${{\varvec{q}}}_{hkl}^{*}$ and predefined peak shape parameters, the peak shape function $L\left({\varvec{q}},{{\varvec{q}}}_{hkl}^{*}\right)$ can be calculated. Then the lattice factor of Eq. (4) can be computed by summing over all (hkl) sets of Miller indices where ${f}_{hkl}\ne 0$

18 $$Z\left({\varvec{q}}\right)=\sum_{h,k,l}{\left|{f}_{hkl}\right|}^{2}L({\varvec{q}},{{\varvec{q}}}_{hkl}^{*})$$

**Figure 5:**
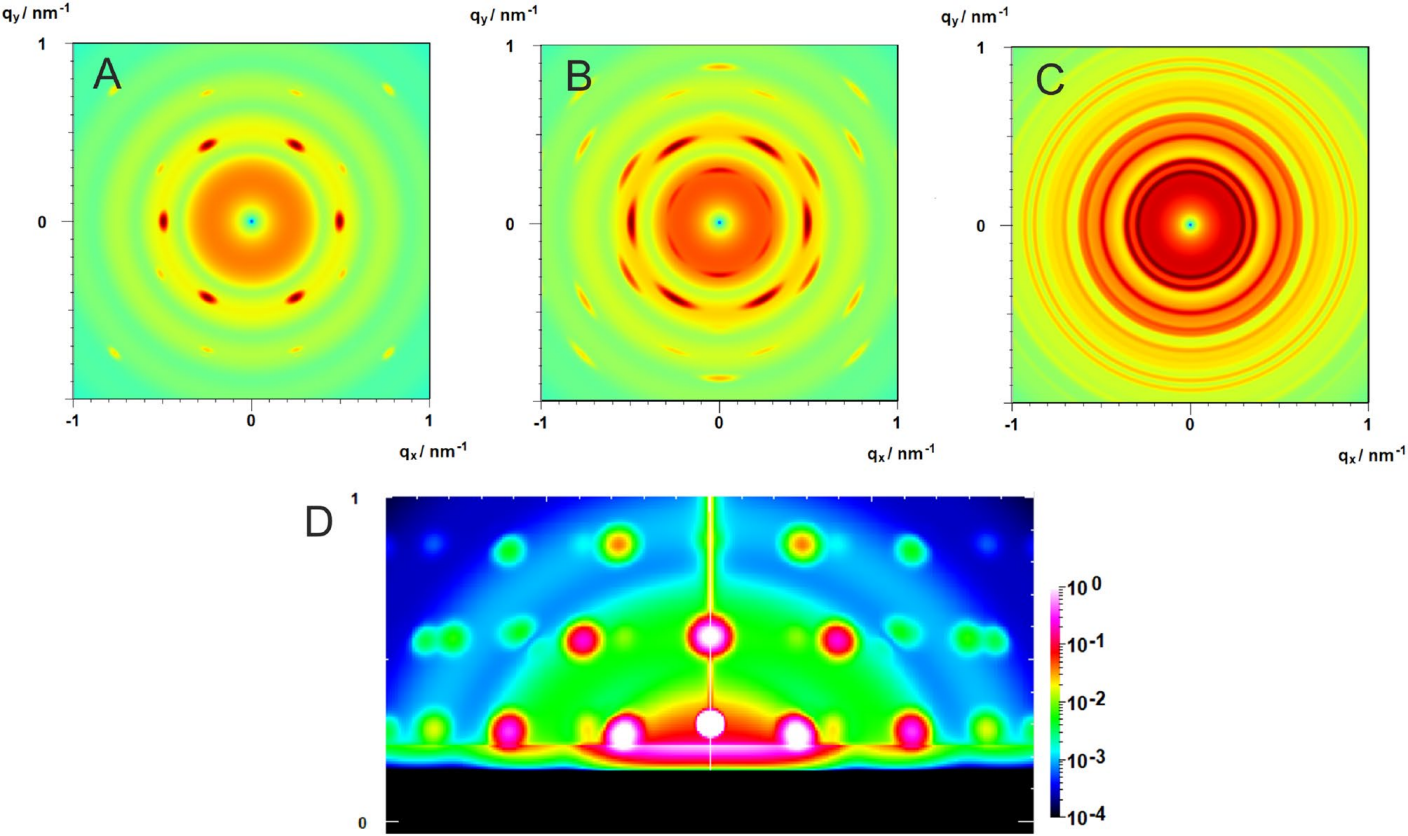
(**A–C**) Calculated 2D-scattering patterns with 4^.^10^6^ pixels (2k × 2k) for spheres (R = 14 nm, σ = 0.08) ordered in an FCC-lattice (a = 35 nm) with high orientational order (**A**), with intermediate orientational order (**B**) and with isotropic orientational distribution exhibiting Debye–Scherrer peaks (**C**). The calculation time for a scattering pattern is 480 ms using a standard consumer graphic card (SI, Sect. 10.2). (**D**) Calculated GISAS-pattern for spheres (R = 10, σ = 0.08) ordered in a BCC-lattice (*a* = 22 nm). The peaks are calculated assuming the beam direction to be parallel to the (001)-direction resulting in a (hk0)-fiber pattern of the BCC-lattice, which is typically observed for nanoparticle assemblies^25^.

For macroscopically isotropic materials, where Debye–Scherrer rings are observed (Fig. 5c), the azimuthal average of $Z\left({\varvec{q}}\right)$ can be obtained in closed analytical form, with *q*-independent coefficients that can be pre-calculated as indicated in Fig. 2 and used for the rapid calculation of 1D-data^22^.

### Grazing incidence scattering (GISAS)

Grazing-incidence small-angle scattering and diffraction (GISAS, GIXD) are scattering techniques using X-rays or neutrons to study nanostructured surfaces and thin films^23,24^. To extend the algorithm to include this important class of scattering experiments requires to include the Fresnel coefficients of transmission and reflection from the film surface and interfaces.

As an example, we consider a thin film containing an assembly of objects, whose scattered intensity $I({{\varvec{q}}}_{ij})$ is given by Eq. (4). The scattered intensity recorded in a GISAS experiment can be calculated within the framework of the Distorted Wave Born Approximation (DWBA)^23^. Therefore, the GISAS scattering intensity is computed as a sum over the four DWBA-terms representing the scattering/reflection events of the object assembly and the film/substrate interface

19 $${I}_{G}({{\varvec{q}}}_{ij})={\left|{T}_{i}\right|}^{2}{\left|{T}_{f}\right|}^{2}\left(I\left({{\varvec{q}}}_{1,ij}\right)+{\left|{R}_{i}\right|}^{2}I\left({{\varvec{q}}}_{2,ij}\right)+{\left|{R}_{f}\right|}^{2}I\left({{\varvec{q}}}_{3,ij}\right)+{\left|{R}_{i}\right|}^{2}{\left|{R}_{f}\right|}^{2}I\left({{\varvec{q}}}_{4,ij}\right)\right)$$

where the ${{\varvec{q}}}_{n,ij}$ are the scattering vectors of the four scattering/reflection events, and the ${T}_{i,f}$ and ${R}_{i,f}$ are the Fresnel transmission and reflection coefficients, respectively. In the Supporting Information (SI Sect. 9) we extend the calculation to include incident plane specular and diffuse scattering. In effect, we multiply the already calculated scattered intensity $I\left({{\varvec{q}}}_{ij}\right)$ with the Fresnel transmission and reflection coefficients, of which ${T}_{i}$ and ${R}_{i}$ are *q*-independent and can be precalculated together with the other coefficients.

To demonstrate the potential of the method, we show in Fig. 5D the calculated GISAS-pattern of polydisperse spheres assembled in a BCC-lattice within a thin film on a substrate. As typical features we observe the Yoneda peak at the critical scattering vector ${q}_{z,c}=0.23$ nm^−1^, and the incident plane diffuse and specular reflection. The peaks are calculated assuming the beam direction to be parallel to the (001)-direction resulting in a (hk0)-fiber pattern of the BCC-lattice, which is typically observed e.g. for nanoparticle assemblies^25^.

## Discussion

For benchmarking we compare CPU computing times between the series algorithm and conventional numerical integration schemes. We computed scattering patterns for the most common geometrical particle shapes that are used for modeling materials structures: spheres, biaxial ellipsoids, triaxial ellipsoids, cylinders, disks, and cubes. For all calculations a typical *q-*range was chosen comprising the low-*q* Guinier and the high-*q* Porod regimes. Moderate axial ratios from 3 to 8 for anisometric particles, and moderate polydispersities of σ = 0.1 were chosen for a fair comparison of the methods. Whenever possible, trigonometric functions in the integrands of the numerical integrations were substituted by non-oscillatory linear functions to gain computational speed. All details of the computations are summarized in the Supporting Information (SI Sect. 11.1). The calculations were performed for a range of data points of 50–10^5^. The smaller numbers are typical for the analysis of 1D-data sets, and the larger numbers typical for 2D-data sets for pixel detectors. The calculations were done on a single CPU core.

**Figure 6:**
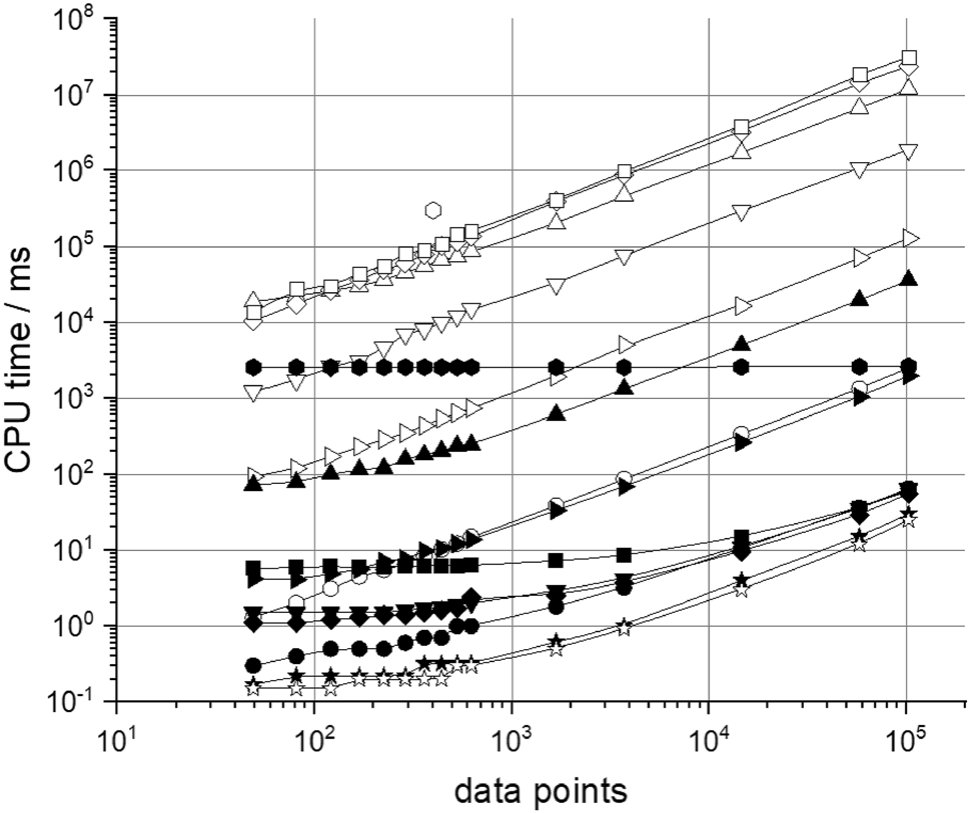
CPU-time to calculate an *N* data point scattering pattern by numerical integration (open symbols) and using the series algorithm (full symbols). We considered the cases of polydisperse spheres (filled circle, open circle), biaxial ellipsoids (filled right side triangle, open right side triangle), triaxial ellipsoids (filled triangle, open triangle), cylinders (filled down side triangle, open down side triangle), disks (filled diamond, open diamond), cubes (filled square, open square), and superellipsoids (open hexagon, filled hexagon). For comparison, also the CPU times for the simple analytical cases of monodisperse spheres (filled star, open star) are provided. For the series expansions we observe a low-*N* plateau due to the pre-calculation time for the *q*-independent coefficients. This computational cost is overcompensated by the subsequent much faster calculation of the scattering patterns. For large number of data points, we observe a gain of up to 5^.^10^5^. For the most common geometrical objects, the computation of one million data points in < 500 ms is possible on just a single core CPU.

Figure 6 shows the CPU-times as a function of the number of data points for different geometrical objects and the two calculation schemes. We find that even in the case of simple spherical particles for a small number of data points the series expansions are faster by a factor of 4, and therefore already have a computational benefit. For a large number of data points, e.g. for $N=$ ${10}^{5}$ in the asymptotic $t\sim {N}^{1}$-region, the series expansions for spheres are faster by a factor of 40 (see SI Sect. 11). For anisometric objects, the series expansion algorithm is faster by factor of up to 7^.^10^1^ for biaxial ellipsoids, 3^.^10^2^ for triaxial ellipsoids, 2^.^10^5^ for cylinders, and 5^.^10^5^ for disks and cubes. For most common objects the series algorithm allows to compute 10^6^ data points in < 500 ms on a single core CPU. Only for the case of monodisperse spheres with a simple analytical expression of the formfactor, the series algorithm is slightly slower, by ca. 20% (SI Sect. 11.3). The expansions and numerical integrations are performed with a relative precision of 10^−4^. In the Supporting Information (SI Sect. 11.2) we show that the expansions converge fast, such that reaching higher precisions require much smaller increases in CPU time compared to numerical integrations.

We also considered the most challenging case of polydisperse superballs. For superballs with edges of equal lengths ($a=b=c$), where five numerical integrations are necessary, CPU times of ca. 5 min. for 400 data points have been reported with optimized numerical integration routines^26^. We considered the more general case of superballs with unequal edges ($a\ne b\ne c$) as shown in Fig. 6. Here, the pre-calculation of the coefficients is the rate-limiting step requiring 2.5 s, which already is > 100-times faster compared to numerical integration. If we extrapolate the reported CPU time to 10^5^ data points, the gain in computational speed is > 10^7^.

The use of centro-symmetry of $I({{\varvec{q}}}_{ij})$ (Friedel’s law) and further symmetries of particles and lattices leads to a further at least two-fold reduction of CPU-time, enabling already > 1 fps calculation of 16 million data point 4k pixel detector scattering patterns with a single core CPUs as motivated in the Introductory Section.

The algorithm can be efficiently implemented into GPUs, because the calculations of the pixel scattering intensities $I({{\varvec{q}}}_{ij})$ are mutually independent. We demonstrate that already simple consumer graphic cards can accelerate the algorithm by a further factor of > 50, enabling sub-second 1D- and 2D-fitting of very large detector array data as demonstrated in the Supporting Information (SI Sect. 12). As applications we demonstrate in the Supporting Information the simulation of large 2D small-angle X-ray (SAXS), small-angle neutron (SANS) and small-angle light scattering patterns, as well as selected area electron diffraction (SAED) patterns with 2k- or 4k-detectors (SI Sect. 12). We furthermore show GPU-accelerated 2D-fitting, and examples of simulated data sets for the training of neural networks.

## Conclusions

We demonstrated an algorithm based on the use of hypergeometric functions that computes 1D-scattering data and 2D-scattering patterns of assemblies of geometrical objects up to a factor of > 10^5^ faster than conventional numerical integration schemes. This acceleration is possible, because hypergeometric functions can be efficiently computed via series and asymptotic expansions, the expansion coefficients can be rapidly calculated via recursion relations and are *q*-independent. They are therefore the same for every pixel and can be pre-calculated and provided to the (i,j)-pixel calculation loop as described in the algorithm in Fig. 2. Over large *q*-ranges, only one or two terms of the expansion are necessary to compute the scattering intensities with sufficient accuracy. The algorithm enables the fast calculation of scattering patterns of simple and complex objects with defined spatial and orientational distributions. Since the computations of the pixel scattering intensities are mutually independent, the calculation can be efficiently implemented into parallel algorithms for GPUs for further significant acceleration. The algorithm enables rapid calculation of large area 2D-scattering patterns and 1D-scattering data enabling high-throughput fitting of large 1D- and 2D-data sets, on-the-fly data analysis for steering scattering experiments, and fast training of neural networks. It thereby helps addressing the data analysis bottleneck for widespread application in the structural analysis of synthetic and biological materials using X-ray, neutron, light and electron scattering and diffraction experiments. The significant saving in computation time of factors of 10^5^–10^7^ furthermore considerably reduces computer energy consumption relevant for green IT.

## Methods

All mathematical and computational methods are described in the Supporting Information. We provide Mathematica and C++ source code for verification and description of the methods. We further provide the full C++ source code and a compiled executable standalone software. Under https://github.com/neutron-simlab/CrystalScatter.

## Supplementary Information

Supplementary Information 1.

Supplementary Video 1.

## Data Availability

All code and results presented in this paper are available open-source and open-access in the associated GitHub repository under https://github.com/neutron-simlab/CrystalScatter.
