# Supplementary material for: Fast calculation of scattering patterns using hypergeometric function algorithms
Source: Sci Rep. 2023 Jan 15;13:780. doi: 10.1038/s41598-023-27558-8 (PMC9841017; doi:10.1038/s41598-023-27558-8)
Supplement: Supplementary file 1 — Supplementary Information 1. [file 41598_2023_27558_MOESM1_ESM.pdf]

Supporting Information

Fast Calculation of Scattering Patterns Using Hypergeometric Function Algorithms

Michael Wagener [1] and Stephan Förster [1,2]*

[1] Jülich Centre for Neutron Science (JCNS-1/IBI-8), Forschungszentrum Jülich, 52425 Jülich, Germany
[2] Institute of Physical Chemistry, RWTH Aachen University, 52074 Aachen, Germany

Table of Contents

1. Hypergeometric Functions

1.1 Series and asymptotic expansions

1.2 Relation to scattering amplitudes and formfactors

1.3 Series coefficients

1.4 Core/shell structures

2. Averages over Powers and Trigonometric Functions

3. Q-limits of Series and Asymptotic Expansions

3.1. Limits of series expansions

3.2 Limits of asymptotic expansions

4. Isotropic Scattering Amplitudes and Formfactors

4.1 Spheres

4.2 Biaxial Ellipsoids

4.3 Triaxial Ellipsoids

4.4 Cube

4.5 Parallelepiped

4.6 Cylinder

4.7 Disk

4.8 Superball

4.9 Superellipsoid

4.9.1 Superellipsoidal rotation bodies

4.9.2 Superellipsoid-shaped lenses and dumbbells

4.9.3 Superellipsoid with ellipsoidal cross-section

4.10 Exluded Volume Chain

5. Porod Asymptotes for isotropic and anisotropic cases

6. Perfect Orientation

6.1 Parallelepiped

6.2 Cylinder and Disk

6.3 Biaxial Ellipsoid

6.4 Triaxial Ellipsoid

7. Axial Orientational distributions

7.1 Coordinate System

7.2. Orientational Distribution Functions

7.3. Calculation Scheme

7.4 Alignment parallel to x-axis

7.5 Alignment parallel to y-axis

7.6 Alignment parallel to z-axis

7.7. Alignment to any axis

7.8. Oriented Parallelepipeds

7.9. Oriented Ellipsoids

8. Ordered Lattices

9. Grazing Incidence Scattering Patterns

10. Implementation

10.1 Recursion relations

10.2 GPU implementation

11. Benchmark CPU times

12. Applications

12.1 X-ray, neutron, light, electron scattering applications

12.2 2D-Fitting

12.3 Training Data for Neural Networks

13. References

1. Hypergeometric Functions

1.1 Series and asymptotic expansions

Hypergeometric functions ${}_pF_q(z)$ can be defined via a hypergeometric series **(S.1.1.1)**

$$ {}_pF_q\left({a_1,\dots,a_p \atop b_1,\dots,b_q};z\right) = \sum_{n=0}^{\infty} \frac{(a_1)_n \dots (a_p)_n}{(b_1)_n \dots (b_q)_n} \frac{z^n}{n!} $$

with the Pochhammer factorial

$$ (x)_n = \frac{\Gamma(x+n)}{\Gamma(x)} = x(x+1)\dots(x+n-1) $$

In more general terms, the hypergeometric functions are defined via a Barnes contour integral **(S.1.1.2)**

$$ {}_pF_q\left({a_1,\dots,a_p \atop b_1,\dots,b_q};z\right) = \frac{1}{2\pi i} \frac{\prod_{m=0}^{q}\Gamma(b_m)}{\prod_{n=0}^{p}\Gamma(a_n)} \int_C \frac{\Gamma(-s) z^s \prod_{n=1}^{p}\Gamma(a_n+s)}{\prod_{m=0}^{q}\Gamma(b_m+s)} ds $$

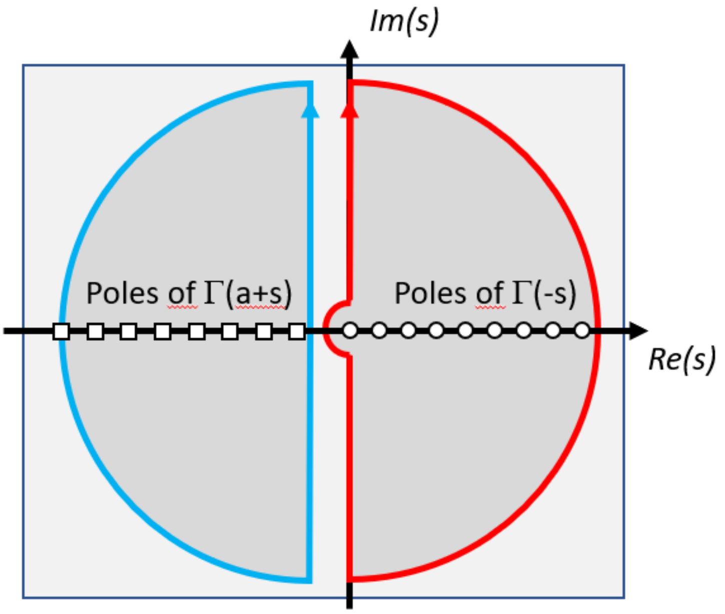

Fig. S1. Calculation of hypergeometric functions via Barnes contour integrals. The contour over the poles on the right-hand side of the complex plane provides the series expansion, whereas the contour over the poles on the left-hand side provides the asymptotic expansion.

The series expansion (Eq. 1.1.1) is obtained as a sum of the residues of the integrand in Eq. (1.1.2) over the poles on the right-hand side of the complex plane as shown in Fig. S1. It is valid

for $p \leq q+1$ for all $|z|<1$. By summation over the poles on the left-hand side we obtain the asymptotic expansion in reciprocal powers of z, which is valid for $|z|>1$.

A comprehensive overview over the series and asymptotic expansions of hypergeometric functions is provided in ref. [S1,11], and follows the derivations by Braaksma. [10] We are interested in the special case $p=q-1$ for which

$$\frac{\prod_{l=1}^{q-1}\Gamma(a_l)}{\prod_{l=1}^{q}\Gamma(b_l)}\,{}_{q-1}F_q\left(\begin{matrix}a_1,\dots,a_{q-1}\\ b_1,\dots,b_q\end{matrix};-z\right)\sim H_{q-1,q}(z)+E_{q-1,q}\left(ze^{-\pi i}\right)+E_{q-1,q}\left(ze^{\pi i}\right)$$

with

$$E_{p,q}(z)=(2\pi)^{\frac{p-q}{2}}\kappa^{-\nu-(\frac{1}{2})}e^{\kappa z^{1/\kappa}}\sum_{k=0}^{\infty}c_k\left(\kappa z^{\frac{1}{\kappa}}\right)^{\nu-k}$$

$$H_{p,q}(z)=\sum_{m=1}^{p}\sum_{k=0}^{\infty}\frac{(-1)^k}{k!}\Gamma(a_m+k)\frac{\prod_{l=1,\,l\neq m}^{p}\Gamma(a_l-a_m-k)}{\prod_{l=1}^{q}\Gamma(b_l-a_m-k)}z^{-a_m-k}$$

$$\kappa=q-p+1$$

$$\nu=a_1+\dots+a_p-b_1\dots-b_q+\frac{1}{2}(p-q)$$

$$c_0=1$$

$$c_k=-\frac{1}{k\kappa^{\kappa}}\sum_{m=0}^{k-1}c_m\,e_{k,m}$$

$$e_{k,m}=\sum_{j=1}^{q+1}\left(1-\nu-\kappa b_j+m\right)_{\kappa+k-m}\left(\frac{\prod_{l=1}^{p}(a_l-b_j)}{\prod_{l=1,\,l\neq j}^{q+1}(b_l-b_j)}\right)$$

and $b_{q+1}=1$.

For the particular case of ${}_0F_1(b_1;-z)$ we have

$$\frac{1}{\Gamma(b_1)}\,{}_0F_1(b_1;-z)\sim H_{0,1}(z)+E_{0,1}\left(ze^{-\pi i}\right)+E_{0,1}\left(ze^{\pi i}\right)$$

Since $H_{0,1}(z)=0$, we obtain

$$\begin{aligned}\frac{1}{\Gamma(b_1)}\,{}_0F_1(b_1;-z)&=E_{0,1}\left(ze^{-\pi i}\right)+E_{0,1}\left(ze^{\pi i}\right)\\&=(\pi)^{-\frac{1}{2}}z^{\nu/2}\sum_{k=0}^{\infty}c_k2^{-k}z^{-k/2}\frac{e^{i\left(2z^{\frac{1}{2}}+\frac{\pi(\nu-k)}{2}\right)}+e^{-i\left(2z^{\frac{1}{2}}+\frac{\pi(\nu-k)}{2}\right)}}{2}\\&=(\pi)^{-\frac{1}{2}}z^{\nu/2}\sum_{k=0}^{\infty}c_k\cos\left(2z^{\frac{1}{2}}+\frac{\pi(\nu-k)}{2}\right)\left(2z^{1/2}\right)^{-k}\end{aligned}$$

with

$$c_k = -\frac{1}{k\kappa^\kappa}\sum_{m=0}^{k-1} c_m\, e_{k,m}$$

$$c_1 = -\frac{1}{2^2} c_0 e_{1,0}$$

$$e_{k,m} = e_{1,0} = \sum_{j=1}^{3}\left(1-\nu-\kappa b_j+m\right)_{\kappa+k-m}\left(\frac{\prod_{l=1}^{p}(a_l-b_j)}{\prod_{\substack{l=1\\ l\neq j}}^{q+1}(b_l-b_j)}\right)$$

$$= (1-\nu-2b_1)_3\frac{(a_1-b_1)}{(b_2-b_1)(1-b_1)} + (1-\nu-2b_2)_3\frac{(a_1-b_2)}{(b_1-b_2)(1-b_2)} + (1-\nu-2b_3)_3\frac{(a_1-b_3)}{(b_1-1)(b_2-1)}$$

Mathematica code implementation for c_k: c_0, c_1, c_2

```
b1=(d+2)/2;
v=-b1+1/2;
c0=1;
c1=Simplify[(1/(4*(1-b1)))*c0*(Pochhammer[1-v-2*b1,3]+Pochhammer[1-v-2,3])]
c1=Simplify[3*(3-8*b1+4*b1*b1)/(16*(1-b1))]
c2=Simplify[(1/(8*(1-b1)))*(c0*(Pochhammer[1-v-2*b1,4]+Pochhammer[1-v-
2,4])+c1*(Pochhammer[1-v-2*b1+1,3]+Pochhammer[1-v-2+1,3]))]
```

Output1,2: c_1 $-\frac{3(-1+d^2)}{8d}$;

Output3: c_2 $\frac{-45+60d+18d^2-56d^3+27d^4-4d^5}{128d^2}$

Special values:

d	c_0	c_1	c_2
1	1	0	0
2	1	-9/16	3/512
3	1	-1	0

For ${}_1F_2(a_1; b_1, b_2; -z)$ we have

$$H_{1,2}(z) = \sum_{k=0}^{\infty}\frac{(-1)^k}{k!}\Gamma(a_1+k)\left(\frac{1}{\Gamma(b_1-a_1-k)\Gamma(b_2-a_1-k)}\right)z^{-a_1-k}$$

$$= z^{-a_1}\frac{\Gamma(a_1)}{\Gamma(b_1-a_1)\Gamma(b_2-a_1)}\sum_{k=0}^{\infty}\frac{(a_1)_k}{(b_1-a_1)_k(b_2-a_1)_k}\frac{(-1)^k}{k!}z^{-k}$$

$$\kappa = q-p+1 = 2-1+1 = 2$$

$$\nu = a_1-b_1-b_2+\frac{1}{2}(2-1) = a_1-b_1-b_2+\frac{1}{2}$$

$$e_{k,m} = e_{1,0} = \sum_{j=1}^{3} \left(1 - \nu - \kappa b_j + m\right)_{\kappa+k-m} \left(\frac{\prod_{l=1}^{p}(a_l - b_j)}{\prod_{\substack{l=1 \\ l \neq j}}^{q+1}(b_l - b_j)} \right)$$

$$= (1 - \nu - 2b_1)_3 \frac{(a_1 - b_1)}{(b_2 - b_1)(1 - b_1)} + (1 - \nu - 2b_2)_3 \frac{(a_1 - b_2)}{(b_1 - b_2)(1 - b_2)} + (1 - \nu - 2b_3)_3 \frac{(a_1 - b_3)}{(b_1 - 1)(b_2 - 1)}$$

Mathematica code implementation for c_k: c_0, c_1, c_2

```
cc0=1;
v=a1-b1-b2+1/2;
r1=(a1-b1)/((b2-b1)*(1-b1));
r2=(a1-b2)/((b1-b2)*(1-b2));
r3=(a1-1)/((b1-1)*(b2-1));
c1=(1-v-2*b1)*(2-v-2*b1)*(3-v-2*b1)*r1;
c2=(1-v-2*b2)*(2-v-2*b2)*(3-v-2*b2)*r2;
c3=(1-v-2)*(2-v-2)*(3-v-2)*r3;
e10=c1+c2+c3;
cc1=Simplify[cc0*e10/4]
d1=c1*(4-v-2*b1);
d2=c2*(4-v-2*b2);
d3=c3*(4-v-2);
e20=d1+d2+d3;
e1=(2-v-2*b1)*(3-v-2*b1)*(4-v-2*b1)*r1;
e2=(2-v-2*b2)*(3-v-2*b2)*(4-v-2*b2)*r2;
e3=(2-v-2)*(3-v-2)*(4-v-2)*r3;
e21=e1+e2+e3;
cc2=Simplify[(cc0*e20+cc1*e21)/4]
```

Output1: c_1 $\frac{1}{8}(3 - 12\text{a1}^2 + 4\text{b1}^2 - 8\text{b2} + 4\text{b2}^2 - 8\text{b1}(1 + \text{b2}) + 8\text{a1}(1 + \text{b1} + \text{b2}))$

Output1: c_2 $\frac{9\text{a1}^4}{4} + \frac{\text{b1}^4}{4} + \text{a1}^3(1 - 3\text{b1} - 3\text{b2}) - \text{b1}^3(1 + \text{b2}) + \text{a1}^2(-\frac{117}{8} + \text{b1} - \frac{\text{b1}^2}{2} + \text{b2} + 5\text{b1b2} - \frac{\text{b2}^2}{2}) + \text{b1}^2(\frac{39}{8} + \text{b2} + \frac{3\text{b2}^2}{2}) - \frac{1}{4}\text{b1}(31 + 39\text{b2} - 4\text{b2}^2 + 4\text{b2}^3) + \frac{1}{64}(177 - 496\text{b2} + 312\text{b2}^2 - 64\text{b2}^3 + 16\text{b2}^4) + \frac{1}{4}\text{a1}(31 + 4\text{b1}^3 + 39\text{b2} - 4\text{b2}^2 + 4\text{b2}^3 - 4\text{b1}^2(1 + \text{b2}) + \text{b1}(39 - 8\text{b2} - 4\text{b2}^2))$

Example: As an example, we illustrate the derivation of the series and asymptotic expansions as schematically shown in Fig. S1 for the hypergeometric function ${}_1F_2(a_1; b_1, b_2; z)$. It is defined via the Barnes-type contour integral (S.1.1.3)

$${}_1F_2(a_1; b_1, b_2; z) = \frac{1}{2\pi i} \frac{\Gamma(b_1)\Gamma(b_2)}{\Gamma(a_1)} \int_C \frac{\Gamma(a_1 + s)\Gamma(-s)z^s}{\Gamma(b_1 + s)\Gamma(b_2 + s)} ds$$

a) Series expansion: For the series expansion we consider the poles of $\Gamma(-s)$ which are on the right-hand side of the complex plane. The integral over functions $f(s)\Gamma(-s)$ can be computed via the corresponding residues at the poles of $\Gamma(-s)$

$$\int_C f(s)\Gamma(-s)ds = 2\pi i \sum_{n=0}^{N} \mathrm{Res}[f(s)\Gamma(-s); s_n]$$

The gamma function $\Gamma(-s)$ has poles at $s_n = n$. The residue of the product $f(s)\Gamma(-s)$ at a pole $s_n = n$ is given by

$$\mathrm{Res}[f(s)\Gamma(-s); s_n] = f(s_n)\frac{(-1)^n}{n!}$$

For the integrand in the contour integral we have

$$\sum_{n=0}^{N} \mathrm{Res}\left[\frac{\Gamma(a_1+s)z^s}{\Gamma(b_1+s)\Gamma(b_2+s)}\Gamma(-s); s_n\right] = \sum_{n=0}^{N} \frac{\Gamma(a_1+n)z^n}{\Gamma(b_1+n)\Gamma(b_2+n)}\frac{(-1)^n}{n!}$$

Therefore, we have for the hypergeometric function **(S.1.1.4)**

$${}_1F_2(a_1; b_1, b_2; z) = \frac{1}{2\pi i}\frac{\Gamma(b_1)\Gamma(b_2)}{\Gamma(a_1)}\int_C \frac{\Gamma(-s)z^s\Gamma(a_1+s)}{\Gamma(b_1+s)\Gamma(b_2+s)}ds$$

$$= \frac{1}{2\pi i}\frac{\Gamma(b_1)\Gamma(b_2)}{\Gamma(a_1)}2\pi i \sum_{n=0}^{N} \frac{\Gamma(a_1+n)}{\Gamma(b_1+n)\Gamma(b_2+n)}\frac{(-1)^n z^n}{n!}$$

$$= \sum_{n=0}^{N} \frac{(a_1)_n}{(b_1)_n(b_1)_n}\frac{(-z)^n}{n!}$$

The summation over the right-hand side poles of $\Gamma(-s)$ thus provides the series expansion.

b) Asymptotic expansion, non-oscillating part: For the asymptotic expansion we consider the poles of $\Gamma(a_1+s)$ which are on the left-hand side of the complex plane. The poles of $\Gamma(a_1+s)$ are $s_n = -(a_1+n)$. The integral therefore is equal to

$$\int_C f(s)\Gamma(a_1+s)ds = 2\pi i \sum_{n=0}^{N} \mathrm{Res}[f(s)\Gamma(a_1+s); s_n]$$

and the residues are

$$\mathrm{Res}[f(s)\Gamma(a_1+s); s_n] = f(s_n)\frac{(-1)^n}{n!}$$

For the integrand we have

$$\mathrm{Res}\left[\frac{\Gamma(-s)z^s}{\Gamma(b_1+s)\Gamma(b_2+s)}\Gamma(a_1+s); s_n\right] = \sum_{n=0}^{N} \frac{\Gamma(a_1+n)z^{-(a_1+n)}}{\Gamma(b_1-(a_1+n))\Gamma(b_2-(a_1+n))}\frac{(-1)^n}{n!}$$

We use the identity

$$\sin(\pi s) = \frac{\pi}{\Gamma(s)\Gamma(1-s)}$$

twice to rewrite a part of the integrand

$$\frac{1}{\Gamma(b_1-(a_1+n))\Gamma(b_2-(a_1+n))} =$$

$$= \frac{\sin(\pi(b_1-(a_1+n)))\,\Gamma(1-b_1+(a_1+n))\sin(\pi(b_2-(a_1+n)))\,\Gamma(1-b_2+(a_1+n))}{\pi^2}$$

$$= \frac{\sin\big(\pi(b_1-a_1)\big)\,\Gamma(1+a_1-b_1+n))\sin\big(\pi(b_2-a_1)\big)\,\Gamma(1+a_1-b_2+n))}{\pi^2}$$

$$= \frac{\pi^2\Gamma(1+a_1-b_1+n))\Gamma(1+a_1-b_2+n))}{\pi^2\Gamma\big(1-(b_1-a_1)\big)\Gamma(b_1-a_1)\Gamma\big(1-(b_2-a_1)\big)\Gamma(b_2-a_1)}$$

$$= \frac{\Gamma(1+a_1-b_1+n))\Gamma(1+a_1-b_2+n))}{\Gamma(1+a_1-b_1)\Gamma(b_1-a_1)\Gamma(1+a_1-b_2)\Gamma(b_2-a_1)}$$

The whole integral is then equal to

$$\sum_{n=0}^{N} \frac{\Gamma(a_1+n)z^{-(a_1+n)}}{\Gamma(b_1-(a_1+n))\Gamma(b_2-(a_1+n))}\frac{(-1)^n}{n!}$$

$$= \sum_{n=0}^{N} \frac{\Gamma(a_1+n)\Gamma(1+a_1-b_1+n)\Gamma(1+a_1-b_2+n)}{\Gamma(1+a_1-b_1)\Gamma(b_1-a_1)\Gamma(1+a_1-b_2)\Gamma(b_2-a_1)}\frac{(-1)^n}{n!}z^{-(a_1+n)}$$

$$= z^{-a_1}\frac{\Gamma(a_1)}{\Gamma(b_1-a_1)\Gamma(b_2-a_1)}\sum_{n=0}^{N}(a_1)_n(1+a_1-b_1)_n(1+a_1-b_2)_n\frac{(-z)^{-n}}{n!}$$

which leads to the asymptotic expansion **(S.1.1.5)**

$${}_1F_2(a_1;b_1,b_2;z) = \frac{\Gamma(b_1)\Gamma(b_2)}{\Gamma(b_1-a_1)\Gamma(b_2-a_1)}z^{-a_1}\sum_{n=0}^{N}(a_1)_n(1+a_1-b_1)_n(1+a_1-b_2)_n\frac{(-z)^{-n}}{n!}$$

c) Asymptotic expansion, oscillating part: The calculation of the oscillating part proceeds by replacing the gamma functions in the Barnes contour integral by their Stirling expansions as described in [10], to obtain [9]

$${}_1F_2(a_1;b_1,b_2;z) = \frac{\Gamma(b_1)\Gamma(b_2)}{\Gamma(a_1)}(\pi)^{-\frac{1}{2}}z^{\nu/2}\sum_{k=0}^{\infty}e_k\cos\left(2z^{\frac{1}{2}}+\frac{\pi(\nu-k)}{2}\right)\left(2z^{1/2}\right)^{-k}$$

$$\nu = a_1-b_1-b_2+\frac{1}{2}$$

$$e_0 = 1$$

$$e_1 = \frac{3}{8}-(b_1+b_2)+\frac{(b_1-b_2)^2-3a_1^2+2a_1(1+b_1+b_2)}{2}$$

Application: As an application we consider the special case of the sine integral, which can be defined as

$$\mathrm{Si}(x) = \int_0^x \frac{\sin(t)}{t} dt = x \ {}_1F_2\left(\frac{1}{2};\frac{3}{2},\frac{3}{2};-\frac{x^2}{4}\right)$$

According to Eq. (S.1.1.4) the sine integral can be represented as a series expansion

$$= x\sum_{n=0}^{\infty} \frac{\left(\frac{1}{2}\right)_n}{\left(\frac{3}{2}\right)_n\left(\frac{3}{2}\right)_n} \frac{\left(-\frac{x^2}{4}\right)^n}{n!}$$

Using Eq. (S.1.1.5) we can calculate the asymptote as

$${}_1F_2\left(a_1;b_1,b_1;-\frac{x^2}{4}\right) =$$

$$= \frac{\Gamma\left(\frac{3}{2}\right)\Gamma\left(\frac{3}{2}\right)}{\Gamma\left(\frac{3}{2}-\frac{1}{2}\right)\Gamma\left(\frac{3}{2}-\frac{1}{2}\right)}\left(\frac{x^2}{4}\right)^{-\frac{1}{2}}\sum_{n=0}^{N}\left(\frac{1}{2}\right)_n\left(1+\frac{1}{2}-\frac{3}{2}\right)_n\left(1+\frac{1}{2}-\frac{3}{2}\right)_n\frac{\left(-\frac{x^2}{4}\right)^{-n}}{n!}$$

$$= \Gamma^2\left(\frac{3}{2}\right)\frac{2}{\mathrm{x}}\sum_{n=0}^{N}\left(\frac{1}{2}\right)_n (0)_n(0)_n\frac{\left(-\frac{x^2}{4}\right)^{-n}}{n!}$$

Since $(0)_m = 1$ for $m = 0$ and $(0)_m = 0$ for $m > 0$, we only have one non-zero $m = 0$-term.

$${}_1F_2\left(a_1;b_1,b_1;-\frac{x^2}{4}\right) = \Gamma^2\left(\frac{3}{2}\right)\frac{2}{\mathrm{x}} = \frac{\pi}{4}\frac{2}{\mathrm{x}} = \frac{\pi}{2\mathrm{x}}$$

We therefore have for the sine integral the asymptote **<u>(S.1.1.6)</u>**

$$\mathrm{Si}(x) = x\ {}_1F_2\left(\frac{1}{2};\frac{3}{2},\frac{3}{2};-\frac{x^2}{4}\right) = x\frac{\pi}{2\mathrm{x}} = \frac{\pi}{2}$$

1.2 Relation to scattering amplitudes and formfactors

The scattering amplitude $F^{(d)}(q,R)$ of a *d*-dimensional object of size *R* is related to the Fourier transform of the phase $e^{i\boldsymbol{qr}}$ over the particle volume, which can be expressed in terms of a hypergeometric functions as **<u>(S.1.2.1)</u>**

$$F^{(d)}(q,R) = \int e^{i\boldsymbol{qr}} d\boldsymbol{r} = \ {}_0F_1\left(b_1;-\frac{q^2R^2}{4}\right)\sum_{n=0}^{\infty}\frac{1}{(b_1)_n n!}\left(-\frac{q^2R^2}{4}\right)^n$$

$$b_1 = \frac{d+2}{2}$$

The hypergeometric function can be expressed via the series expansion **(S.1.2.2)**

$${}_0F_1\left(b_1; -\frac{q^2R^2}{4}\right) = \sum_{n=0}^{\infty} \frac{1}{(b_1)_n n!}\left(-\frac{q^2R^2}{4}\right)^n$$

or via the asymptotic expansion **(S.1.2.3)**

$${}_0F_1\left(b_1; -\frac{q^2R^2}{4}\right) = \frac{\Gamma(b_1)}{\sqrt{\pi}}\left(\frac{qR}{2}\right)^{\nu} \sum_{k=0}^{\infty} c_k \cos\left(qR + \frac{\pi(\nu-k)}{2}\right)(qR)^{-k}$$

$$\nu = -b_1 + \frac{1}{2}$$

We only require the zeroth and first order term

$$F^{(d)}(q) = \frac{\Gamma(b)}{\sqrt{\pi}}\left(\frac{qR}{2}\right)^{\nu}\left(c_0 \cos\left(qR + \frac{\pi\nu}{2}\right) + c_1^{(d)} \cos\left(qR + \frac{\pi(\nu-1)}{2}\right)(qR)^{-1}\right)$$

$$c_0 = 1, c_1^{(1)} = 0, c_1^{(2)} = 9/8, c_1^{(3)} = -1$$

For disks we have $d = 1, b = \frac{3}{2}, \nu = -1, c_1 = 0$ **(S.1.2.4)**

$$F_1\left(\frac{3}{2}; -z\right) = \frac{\Gamma\left(\frac{3}{2}\right)}{(\pi)^{\frac{1}{2}}} z^{-\frac{1}{2}}\left(\cos\left(2z^{\frac{1}{2}} - \frac{\pi}{2}\right) + c_1 \cos\left(2z^{\frac{1}{2}} - \pi\right)\left(2z^{\frac{1}{2}}\right)^{-1}\right)$$

$$= \frac{\Gamma\left(\frac{3}{2}\right)}{(\pi)^{\frac{1}{2}}} z^{-\frac{1}{2}}\left(\sin\left(2z^{\frac{1}{2}}\right) - c_1 \cos\left(2z^{\frac{1}{2}}\right)\left(2z^{\frac{1}{2}}\right)^{-1}\right)$$

$$F_1\left(\frac{3}{2}; -\frac{q^2R^2}{4}\right) = \frac{\Gamma\left(\frac{3}{2}\right)}{(\pi)^{\frac{1}{2}}}\left(\frac{2}{qR}\right)(\sin(qR) - c_1 \cos(qR)\,(qR)^{-1})$$

$$F_1\left(\frac{3}{2}; -\frac{q^2R^2}{4}\right) = \frac{\Gamma\left(\frac{3}{2}\right)}{(\pi)^{\frac{1}{2}}}\left(\frac{2}{qR}\right)\sin(qR) = \frac{\sin(qR)}{qR}$$

$$P(q) = {}_0F_1^2\left(\frac{3}{2}; -\frac{q^2R^2}{4}\right) = (qR)^{-2}(\sin(qR))^2$$

For cylinders we have $d = 2, b = 2, \nu = -3/2, c_1 = -9/16$ **(S.1.2.5)**

$${}_0F_1(2; -z) = \frac{\Gamma(2)}{(\pi)^{\frac{1}{2}}} z^{-\frac{3}{4}}\left(\cos\left(2z^{\frac{1}{2}} - \frac{3\pi}{4}\right) + c_1 \cos\left(2z^{\frac{1}{2}} - \frac{5\pi}{4}\right)\left(2z^{\frac{1}{2}}\right)^{-1}\right)$$

$$= \frac{\Gamma(2)}{(\pi)^{\frac{1}{2}}} z^{-\frac{3}{4}}\left(\cos\left(2z^{\frac{1}{2}} - \frac{3\pi}{4}\right) + c_1 \cos\left(2z^{\frac{1}{2}} - \frac{5\pi}{4}\right)\left(2z^{\frac{1}{2}}\right)^{-1}\right)$$

$$F_1\left(2;-\frac{q^2R^2}{4}\right)= \frac{\Gamma(2)}{(\pi)^{\frac{1}{2}}}\left(\frac{2}{qR}\right)^{\frac{3}{2}}\left(\cos\left(qR-\frac{3\pi}{4}\right)-\frac{9}{16}\cos\left(qR-\frac{5\pi}{4}\right)(qR)^{-1}\right)$$

$$= \frac{1}{(\pi)^{\frac{1}{2}}}\left(\frac{2}{qR}\right)^{\frac{3}{2}}\left(\cos\left(qR-\frac{3\pi}{4}\right)-\frac{9}{16}\cos\left(qR-\frac{5\pi}{4}\right)(qR)^{-1}\right)$$

$$P(q)= {}_0F_1^2\left(2;-\frac{q^2R^2}{4}\right)$$

$$=\frac{4}{\pi}\left(\frac{1}{qR}\right)^3\left(1-\sin(2qR)-\frac{9}{8}\left(\frac{1}{qR}\right)\cos(2qR)+\left(\frac{9}{16}\right)^2\left(\frac{1}{qR}\right)^2 + \left(\frac{9}{16}\right)^2\left(\frac{1}{qR}\right)^2\sin(2qR)\right)$$

$$=\frac{4}{\pi}\left(\left(\frac{1}{qR}\right)^3-\left(\frac{1}{qR}\right)^3\sin(2qR)-\frac{9}{8}\left(\frac{1}{qR}\right)^4\cos(2qR)+\left(\frac{9}{16}\right)^2\left(\frac{1}{qR}\right)^5+\left(\frac{9}{16}\right)^2\left(\frac{1}{qR}\right)^5\sin(2qR)\right)$$

$$P(q)=\frac{4}{\pi}\left(\left(\frac{1}{qR}\right)^3-\left(\frac{1}{qR}\right)^3\sin(2qR)-\frac{9}{8}\left(\frac{1}{qR}\right)^4\cos(2qR)+\left(\frac{9}{16}\right)^2\left(\frac{1}{qR}\right)^5 + \left(\frac{9}{16}\right)^2\left(\frac{1}{qR}\right)^5\sin(2qR)\right)$$

For spheres we have $d=3, b=\frac{5}{2}, \nu=-2, c_1=-1$ with **(S.1.2.6)**

$${}_0F_1\left(\frac{5}{2};-z\right)= \frac{\Gamma\left(\frac{5}{2}\right)}{(\pi)^{\frac{1}{2}}}z^{-1}\left(\cos\left(2z^{\frac{1}{2}}-\pi\right)+c_1\cos\left(2z^{\frac{1}{2}}-\frac{3\pi}{2}\right)\left(2z^{\frac{1}{2}}\right)^{-1}\right)$$

$$= \frac{\Gamma\left(\frac{5}{2}\right)}{(\pi)^{\frac{1}{2}}}z^{-1}\left(-\cos\left(2z^{\frac{1}{2}}\right)-c_1\sin\left(2z^{\frac{1}{2}}\right)\left(2z^{\frac{1}{2}}\right)^{-1}\right)$$

$$F_1\left(\frac{5}{2};-\frac{q^2R^2}{4}\right)= \frac{\Gamma\left(\frac{5}{2}\right)}{(\pi)^{\frac{1}{2}}}\left(\frac{2}{qR}\right)^2(-\cos(qR)+\sin(qR)\,(qR)^{-1})$$

$$= 3\left(\frac{1}{qR}\right)^3(\sin(qR)-qR\cos(qR))$$

$$P(q)= {}_0F_1^2\left(\frac{5}{2};-\frac{q^2R^2}{4}\right)= 9\left(\frac{1}{qR}\right)^6(\sin(qR)-qR\cos(qR))^2$$

The following Tables provide scattering amplitudes and formfactors for particles of different dimensionalities $d=1,2,3$:

Table S-1: Scattering amplitudes **(S.1.2.7):**

$$\langle F^{(d)}(q)\rangle_R = \sum_{n=0}^{\infty} \frac{1}{\left(\frac{d+2}{2}\right)_n n!}\left(-\frac{q^2R^2}{4}\right)^n$$

$F^{(1)}(q)$		
	$= \frac{\sin(qR)}{qR}$	**S.1.2.8**
series	$= \sum_{n=0}^{\infty} \frac{1}{\left(\frac{3}{2}\right)_n n!}\left(-\frac{q^2R^2}{4}\right)^n$	**S.1.2.9**
asymptote	$= \frac{\sin(qR)}{qR}$	**S.1.2.10**
$F^{(2)}(q)$		
	$= \frac{2J_1(qR)}{qR}$	**S.1.2.11**
Series	$= \sum_{n=0}^{\infty} \frac{1}{(2)_n n!}\left(-\frac{q^2R^2}{4}\right)^n$	**S.1.2.12**
asymptote	$= \frac{2}{\sqrt{\pi}}\left(\frac{\sin(qR)}{(qR)^{3/2}} - \frac{\cos(qR)}{(qR)^{3/2}} + \frac{9}{16}\left(\frac{\cos(qR)}{(qR)^{5/2}} + \frac{\sin(qR)}{(qR)^{5/2}}\right)\right)$	**S.1.2.13**
$F^{(3)}(q)$		
	$= \frac{\sin(qR) - qR\cos(qR)}{(qR)^3}$	**S.1.2.14**
Series	$= \sum_{n=0}^{\infty} \frac{1}{\left(\frac{3}{2}\right)_n n!}\left(-\frac{q^2R^2}{4}\right)^n$	**S.1.2.15**
asymptote	$= \frac{\sin(qR)}{(qR)^3} - \frac{\cos(qR)}{(qR)^2}$	**S.1.2.16**

Table S-2: Scattering amplitudes, size-averaged **(S.1.2.17):**

$$\langle F^{(d)}(q)\rangle_R = \int_0^{\infty} F^{(d)}(q)R^s h(R)dR = \sum_{n=0}^{\infty} \frac{(z_R+s+1)_{2n}}{\left(\frac{d+2}{2}\right)_n n!}\left(-\frac{q^2R^2}{4(z_R+1)^2}\right)^n$$

$\langle F^{(1)}(q)\rangle_R$		
$s = 1$	$= \langle\frac{\sin(qR)}{qR}\rangle_R = \int_0^{\infty} \frac{\sin(qR)}{qR} R^s h(R)dR$	**S.1.2.18**
series	$= \sum_{n=0}^{\infty} \frac{(z_R+s+1)_{2n}}{\left(\frac{3}{2}\right)_n n!}\left(-\frac{q^2R^2}{4(z_R+1)^2}\right)^n$	**S.1.2.19**

asymptote	$=\langle\frac{\sin(qR)}{qR}\rangle_R$	**S.1.2.20**
$\langle F^{(2)}(q)\rangle_R$		
$s=2$	$=\langle\frac{2J_1(qR}{qR}\rangle_R=\int_0^\infty \frac{2J_1(qR}{qR}R^s h(R)dR$	**S.1.2.21**
series	$=\sum_{n=0}^{\infty}\frac{(z_R+s+1)_{2n}}{(2)_n n!}\left(-\frac{q^2R^2}{4(z_R+1)^2}\right)^n$	**S.1.2.22**
asymptote	$=\frac{2}{\sqrt{\pi}}\left(\langle\frac{\sin(qR)}{(qR)^{3/2}}\rangle_R-\langle\frac{\cos(qR)}{(qR)^{3/2}}\rangle_R+\frac{9}{16}\left(\langle\frac{\cos(qR)}{(qR)^{5/2}}\rangle_R+\langle\frac{\sin(qR)}{(qR)^{5/2}}\rangle_R\right)\right)$	**S.1.2.23**
$\langle F^{(3)}(q)\rangle_R$		
$s=3$	$=\langle\frac{\sin(qR)-qR\cos(qR)}{(qR)^3}\rangle_R=\int_0^\infty \frac{\sin(qR)-qR\cos(qR)}{(qR)^3}R^s h(R)dR$	**S.1.2.24**
series	$=\sum_{n=0}^{\infty}\frac{(z_R+s+1)_{2n}}{\left(\frac{5}{2}\right)_n n!}\left(-\frac{q^2R^2}{4(z_R+1)^2}\right)^n$	**S.1.2.25**
asymptote	$=\langle\frac{\sin(qR)}{(qR)^3}\rangle_R-\langle\frac{\cos(qR)}{(qR)^2}\rangle_R$	**S.1.2.26**

Table S-3: Scattering amplitudes, size-averaged, squared **(S.1.2.27):**

$$\langle F^{(d)}(q)\rangle_R^2=\left(\int_0^\infty F^{(d)}(q)R^s h(R)dR\right)^2=\sum_{n=0}^{\infty}\left(-\frac{q^2R^2}{4(z_R+1)^2}\right)^n\sum_{m=0}^{n}\frac{(z_R+s+1)_{2(n-m)}(z_R+s+1)_{2m}}{\left(\frac{d+2}{2}\right)_{n-m}(n-m)!\left(\frac{d+2}{2}\right)_n n!}$$

$\langle F^{(1)}(q)\rangle_R^2$		
$s=1$	$=\langle\frac{\sin(qR)}{qR}\rangle_R^2=\left(\int_0^\infty \frac{\sin(qR)}{qR}R^s h(R)RdR\right)^2$	**S.1.2.29**
series	$=\sum_{n=0}^{\infty}\left(-\frac{q^2R^2}{4(z_R+1)^2}\right)^n\sum_{m=0}^{n}\frac{(z_R+s+1)_{2(n-m)}(z_R++s1)_{2m}}{\left(\frac{3}{2}\right)_{n-m}(n-m)!\left(\frac{3}{2}\right)_n n!}$	**S.1.2.30**
asymptote	$=\left(\langle\frac{\sin(qR)}{qR}\rangle_R\right)^2$	**S.1.2.31**
$\langle F^{(2)}(q)\rangle_R^2$		
$s=2$	$=\langle\frac{2J_1(qR}{qR}\rangle_R^2=\left(\int_0^\infty \frac{2J_1(qR}{qR}R^s h(R)dR\right)^2$	**S.1.2.32**
series	$=\sum_{n=0}^{\infty}\left(-\frac{q^2R^2}{4(z_R+1)^2}\right)^n\sum_{m=0}^{n}\frac{(z_R+s+1)_{2(n-m)}(z_R++s1)_{2m}}{(2)_{n-m}(n-m)!(2)_n n!}$	**S.1.2.33**
asymptote	$=\frac{4}{\pi}\left(\langle\frac{\sin(qR)}{(qR)^{3/2}}\rangle_R-\langle\frac{\cos(qR)}{(qR)^{3/2}}\rangle_R+\frac{9}{16}\left(\langle\frac{\cos(qR)}{(qR)^{5/2}}\rangle_R+\langle\frac{\sin(qR)}{(qR)^{5/2}}\rangle_R\right)\right)^2$	**S.1.2.34**

$\langle F^{(3)}(q)\rangle_R^2$		
$s=3$	$\langle\frac{\sin(qR)-qR\cos(qR)}{(qR)^3}\rangle_R^2=\left(\int_0^\infty\frac{\sin(qR)-qR\cos(qR)}{(qR)^3}R^s h(R)dR\right)^2$	**S.1.2.35**
series	$=\sum_{n=0}^{\infty}\left(-\frac{q^2R^2}{4(z_R+1)^2}\right)^n\sum_{m=0}^{n}\frac{(z_R+s+1)_{2(n-m)}(z_R+s+1)_{2m}}{\left(\frac{5}{2}\right)_{n-m}(n-m)!\left(\frac{5}{2}\right)_n n!}$	**S.1.2.36**
asymptote	$=\left(\langle\frac{\sin(qR)}{(qR)^3}\rangle_R-\langle\frac{\cos(qR)}{(qR)^2}\rangle_R\right)^2$	**S.1.2.37**

Table S-4: Formfactors **(S.1.2.38):**

$$P^{(d)}(q)=\sum_{n=0}^{\infty}\frac{4^{b+n-1}\Gamma(b)\Gamma\left(b+n-\frac{1}{2}\right)}{\sqrt{\pi}\Gamma(2b+n-1)}\frac{1}{(b)_n n!}\left(-\frac{q^2R^2}{4}\right)^n$$

$$b=\frac{d+2}{2}$$

$P^{(1)}(q)$		
	$=\left(\frac{\sin(qR)}{qR}\right)^2$	**S.1.2.39**
series	$=\sum_{n=0}^{\infty}\frac{4^n}{(\mathrm{n}+1)}\frac{1}{\left(\frac{3}{2}\right)_n n!}\left(-\frac{q^2R^2}{4}\right)^n$	**S.1.2.40**
asymptote	$=\frac{(\sin(qR))^2}{(qR)^2}$	**S.1.2.41**
$P^{(2)}(q)$		
	$=\left(\frac{2J_1(qR}{qR}\right)^2$	**S.1.2.42**
series	$=\sum_{n=0}^{\infty}\frac{4^{n+1}\Gamma\left(n+\frac{3}{2}\right)}{\sqrt{\pi}\Gamma(n+3)}\frac{1}{(2)_n n!}\left(-\frac{q^2R^2}{4}\right)^n$	**S.1.2.43**
asymptote	$=\frac{4}{\pi}\left(\left(\frac{1}{qR}\right)^3-\frac{\sin(2qR)}{(qR)^3}-\frac{9}{8}\frac{\cos(2qR)}{(qR)^4}+\left(\frac{9}{16}\right)^2\left(\frac{1}{qR}\right)^5+\left(\frac{9}{16}\right)^2\frac{\sin(2qR)}{(qR)^5}\right)$	**S.1.2.44**
$P^{(3)}(q)$		
	$=\left(\frac{\sin(qR)-qR\cos(qR)}{(qR)^3}\right)^2$	**S.1.2.45**
series	$=\sum_{n=0}^{\infty}\frac{6\cdot 4^n}{(\mathrm{n}+3)(\mathrm{n}+2)}\frac{1}{\left(\frac{5}{2}\right)_n n!}\left(-\frac{q^2R^2}{4}\right)^n$	**S.1.2.46**
asymptote	$=9\left(\frac{(\cos(qR))^2}{(qR)^4}-\frac{\sin(qR)\cos(qR)}{(qR)^5}+\frac{(\sin(qR))^2}{(qR)^6}\right)$	**S.1.2.47**

Table S-5: Formfactors, size-averaged **(S.1.2.48):**

$\langle P^{(1)}(q)\rangle_R$		
$s=2$	$=\langle\left(\frac{\sin(qR)}{qR}\right)^2\rangle_R=\int_0^\infty\left(\frac{\sin(qR)}{qR}\right)^2 R^s h(R)dR$	**S.1.2.49**
series	$=\sum_{n=0}^{\infty}\frac{4^n}{(\mathrm{n}+1)}\frac{(z_r+s+1)_{2n}}{\left(\frac{3}{2}\right)_n n!}\left(-\frac{q^2R^2}{4(z_r+1)^2}\right)^n$	**S.1.2.50**
asymptote	$=\langle\left(\frac{\sin(qR)}{qR}\right)^2\rangle_R$	**S.1.2.51**
$\langle P^{(2)}(q)\rangle_R$		
$s=4$	$=\langle\left(\frac{2J_1(qR}{qR}\right)^2\rangle_R=\int_0^\infty\left(\frac{2J_1(qR}{qR}\right)^2 R^s h(R)dR$	**S.1.2.52**
series	$=\sum_{n=0}^{\infty}\frac{4^{n+1}\Gamma\left(n+\frac{3}{2}\right)}{\sqrt{\pi}\Gamma(n+3)}\frac{(z_r+s+1)_{2n}}{(2)_n n!}\left(-\frac{q^2R^2}{4(z_r+1)^2}\right)^n$	**S.1.2.53**
asymptote	$=\frac{4}{\pi}\left(\langle\left(\frac{1}{qR}\right)^3\rangle_R-\langle\frac{\sin(2qR)}{(qR)^3}\rangle_R-\frac{9}{8}\langle\frac{\cos(2qR)}{(qR)^4}\rangle_R+\left(\frac{9}{16}\right)^2\langle\left(\frac{1}{qR}\right)^5\rangle_R+\left(\frac{9}{16}\right)^2\langle\frac{\sin(2qR)}{(qR)^5}\rangle_R\right)$	**S.1.2.54**
$\langle P^{(3)}(q)\rangle_R$		
$s=6$	$=\langle\left(\frac{\sin(qR)-qR\cos(qR)}{(qR)^3}\right)^2\rangle_R=\int_0^\infty\left(\frac{\sin(qR)-qR\cos(qR)}{(qR)^3}\right)^2 R^s h(R)dR$	**S.1.2.55**
series	$=\sum_{n=0}^{\infty}\frac{6\cdot 4^n}{(\mathrm{n}+3)(\mathrm{n}+2)}\frac{(z_r+s+1)_{2n}}{\left(\frac{5}{2}\right)_n n!}\left(-\frac{q^2R^2}{4(z_r+1)^2}\right)^n$	**S.1.2.56**
asymptote	$=9\left(\langle\frac{(\cos(qR))^2}{(qR)^4}\rangle_R-\langle\frac{\sin(qR)\cos(qR)}{(qR)^5}\rangle_R+\langle\frac{(\sin(qR))^2}{(qR)^6}\rangle_R\right)$	**S.1.2.57**

1.3 Series coefficients

Coefficients for $\langle F^{(d)}(q)\rangle_R^2$: **(S.1.3.1**)

$$\langle F^{(d)}(q)\rangle_R^2=\sum_{n=0}^{\infty}\left(-\frac{q^2R^2}{4(z_R+1)^2}\right)^n f_n^{(d)}$$

$$f_n^{(d)}=\sum_{m=0}^{n}\frac{(z_r+s+1)_{2(n-m)}(z_r+s+1)_{2m}}{\left(\frac{d+2}{2}\right)_{n-m}\left(\frac{d+2}{2}\right)_m (n-m)!\,m!}$$

$$f_n^{(1)}=\sum_{m=0}^{n}\frac{(z_r+s+1)_{2(n-m)}(z_r+s+1)_{2m}}{\left(\frac{3}{2}\right)_{n-m}\left(\frac{3}{2}\right)_m (n-m)!\,m!}=\sum_{m=0}^{n}\frac{\Gamma\left[\frac{3}{2}\right]\Gamma\left[\frac{3}{2}\right](z_r+s+1)_{2(n-m)}(z_r+s+1)_{2m}}{\Gamma\left[n-m+\frac{3}{2}\right]\Gamma\left[m+\frac{3}{2}\right](n-m)!\,m!}$$

$$= \frac{\pi}{4} \sum_{m=0}^{n} \frac{(z_r + s + 1)_{2(n-m)} (z_r + s + 1)_{2m}}{\Gamma\left[n - m + \frac{3}{2}\right] \Gamma\left[m + \frac{3}{2}\right] (n - m)!\, m!}$$

$$f_n^{(2)} = \sum_{m=0}^{n} \frac{(z_r + s + 1)_{2(n-m)} (z_r + s + 1)_{2m}}{(2)_{n-m} (2)_m (n - m)!\, m!} = \sum_{m=0}^{n} \frac{\Gamma[2]\Gamma[2] (z_r + s + 1)_{2(n-m)} (z_r + s + 1)_{2m}}{\Gamma[n - m + 2]\Gamma[m + 2] (n - m)!\, m!}$$

$$= \sum_{m=0}^{n} \frac{(z_r + s + 1)_{2(n-m)} (z_r + s + 1)_{2m}}{(\mathrm{n - m + 1})!\, (\mathrm{m + 1})!\, (n - m)!\, m!}$$

$$f_n^{(3)} = \sum_{m=0}^{n} \frac{(z_r + s + 1)_{2(n-m)} (z_r + s + 1)_{2m}}{\left(\frac{5}{2}\right)_{n-m} \left(\frac{5}{2}\right)_m (n - m)!\, m!} = \sum_{m=0}^{n} \frac{\Gamma\left[\frac{5}{2}\right] \Gamma\left[\frac{5}{2}\right] (z_r + s + 1)_{2(n-m)} (z_r + s + 1)_{2m}}{\Gamma\left[n - m + \frac{5}{2}\right] \Gamma\left[m + \frac{5}{2}\right] (n - m)!\, m!}$$

$$= \frac{9\pi}{16} \sum_{m=0}^{n} \frac{(z_r + s + 1)_{2(n-m)} (z_r + s + 1)_{2m}}{\Gamma\left[n - m + \frac{5}{2}\right] \Gamma\left[m + \frac{5}{2}\right] (n - m)!\, m!}$$

Coefficients for $\langle P^{(d)}(q) \rangle_R$: **<u>(S.1.3.2)</u>**

$$\langle P^{(d)}(q) \rangle_R = \sum_{n=0}^{\infty} \frac{(z_r + s + 1)_{2n}}{\left(\frac{3}{2}\right)_n n!} \left(-\frac{q^2 R^2}{4(z_r + 1)^2}\right)^n p_n^{(d)}$$

$$p_n^{(d)} = \sum_{m=0}^{n} \frac{1}{(b)_{n-m} (b)_m (n - m)!\, m!} = \frac{4^{b+n-1} \Gamma(b) \Gamma\left(b + n - \frac{1}{2}\right)}{\sqrt{\pi}\, \Gamma(2b + n - 1)} \frac{1}{(b)_n n!}$$

$$p_n^{(1)} = \frac{4^n}{(\mathrm{n} + 1)}$$

$$p_n^{(2)} = \frac{4^{n+1} \Gamma\left(n + \frac{3}{2}\right)}{\sqrt{\pi}\, \Gamma(n + 3)}$$

$$p_n^{(3)} = \frac{6 \cdot 4^n}{(\mathrm{n} + 3)(\mathrm{n} + 2)}$$

1.4 Core/shell structures

For *d*-dimensional centro-symmetric domain we have for the scattering amplitude [9]

$$F(q) = \frac{\int_0^R {}_0F_1\left(\frac{d}{2}; -\frac{q^2 r^2}{4}\right) \frac{2\pi^{d/2}}{\Gamma[d/2]} r^{d-1} dr}{\int_0^R \frac{2\pi^{d/2}}{\Gamma[d/2]} r^{d-1} dr} = \frac{V_d(R)\; {}_0F_1\left(\frac{d+2}{2}; -\frac{q^2 R^2}{4}\right)}{V_d(R)}$$

with the volume

$$V_d(R) = \frac{2\pi^{d/2}}{d\Gamma[d/2]} R^d$$

For a single shell-domain between the lower radial limit R_1 and the upper radial limit R_2, and with a domain density ρ, the scattering amplitude is

$$F(q) = \frac{\int_{R_1}^{R_2} \rho \; {}_0F_1\left(\frac{d}{2}; -\frac{q^2r^2}{4}\right) \frac{2\pi^{d/2}}{\Gamma[d/2]} r^{d-1} dr}{\int_{R_1}^{R_2} \rho \frac{2\pi^{d/2}}{\Gamma[d/2]} r^{d-1} dr}$$

$$= \frac{\rho V_d(R_2) \; {}_0F_1\left(\frac{d+2}{2}; -\frac{q^2R_2^2}{4}\right) - \rho V_d(R_1) \; {}_0F_1\left(\frac{d+2}{2}; -\frac{q^2R_1^2}{4}\right)}{\rho V_d(R_2) - \rho V_d(R_1)}$$

For N concentric shell-domains i between the lower radial limit R_i and the upper radial limit R_{i+1}, and with a domain density ρ_i, the scattering amplitude is

$$F(q) = \frac{\sum_{i=1}^{N+1} \int_{R_{i-1}}^{R_i} \rho_i \; {}_0F_1\left(\frac{d}{2}; -\frac{q^2r^2}{4}\right) \frac{2\pi^{d/2}}{\Gamma[d/2]} r^{d-1} dr}{\sum_{i=1}^{N+1} \int_{R_{i-1}}^{R_i} \rho_i \frac{2\pi^{d/2}}{\Gamma[d/2]} r^{d-1} dr}$$

$$= \frac{\sum_{i=1}^{N+1} \left(\rho_i V_d(R_i) \; {}_0F_1\left(\frac{d+2}{2}; -\frac{q^2R_i^2}{4}\right) - \rho_i V_d(R_{i-1}) \; {}_0F_1\left(\frac{d+2}{2}; -\frac{q^2R_{i-1}^2}{4}\right) \right)}{\sum_{i=1}^{N+1} (\rho_i V_d(R_i) - \rho_i V_d(R_{i-1}))}$$

Since $R_0 = 0$ and $\rho_{N+1} = 0$ the sum over domains can be recast into a sum over interfaces

$$F(q) = \frac{\sum_{i=1}^{N} (\rho_i V_d(R_i) - \rho_{i+1} V_d(R_i)) \; {}_0F_1\left(\frac{d+2}{2}; -\frac{q^2R_i^2}{4}\right)}{\sum_{i=1}^{N} (\rho_i V_d(R_i) - \rho_{i+1} V_d(R_i))}$$

Using the expression for the volumes we have

$$F(q) = \frac{\sum_{i=1}^{N} \left(\rho_i R_i^d - \rho_{i+1} R_i^d\right) \; {}_0F_1\left(\frac{d+2}{2}; -\frac{q^2R_i^2}{4}\right)}{\sum_{i=1}^{N} \left(\rho_i R_i^d - \rho_{i+1} R_i^d\right)}$$

Further, we use $R_i = p_i R$ with $R = R_N$ the outer radius to obtain

$$F(q) = \frac{\sum_{i=1}^{N} \left(\rho_i p_i^d - \rho_{i+1} p_i^d\right) \; {}_0F_1\left(\frac{d+2}{2}; -\frac{q^2p_i^2R^2}{4}\right)}{\sum_{i=1}^{N} \left(\rho_i p_i^d - \rho_{i+1} p_i^d\right)}$$

For the formfactor we have the squared expression

$$P(q) = \frac{\left(\sum_{i=1}^{N} \left(\rho_i p_i^d - \rho_{i+1} p_i^d\right) \; {}_0F_1\left(\frac{d+2}{2}; -\frac{q^2p_i^2R^2}{4}\right)\right)^2}{\left(\sum_{i=1}^{N} \left(\rho_i p_i^d - \rho_{i+1} p_i^d\right)\right)^2}$$

$$= \frac{1}{\left(\sum_{i=1}^{N}(\rho_i - \rho_{i+1})p_i^d\right)^2}\left(\sum_{i=1}^{N}(\rho_i - \rho_{i+1})^2 p_i^{2d}\ {}_0F_1^2\left(\frac{d+2}{2}; -\frac{q^2p_i^2R^2}{4}\right)\right.$$

$$\left. + 2\sum_{i=1}^{N}\sum_{j=1}^{i-1}(\rho_i - \rho_{i+1})(\rho_i - \rho_{i+1})p_i^d p_j^d\ {}_0F_1\left(\frac{d+2}{2}; -\frac{q^2p_i^2R^2}{4}\right)\ {}_0F_1\left(\frac{d+2}{2}; -\frac{q^2p_j^2R^2}{4}\right)\right)$$

For a simple core/shell system (N=2) we therefore have

$$P(q) = \frac{\left(\sum_{i=1}^{2}(\rho_i p_i^d - \rho_{i+1}p_i^d)\ {}_0F_1\left(\frac{d+2}{2}; -\frac{q^2p_i^2R^2}{4}\right)\right)^2}{\left(\sum_{i=1}^{2}(\rho_i p_i^d - \rho_{i+1}p_i^d)\right)^2}$$

$$= \frac{\left(\rho_1 p_1^d\ {}_0F_1\left(\frac{d+2}{2}; -\frac{q^2p_1^2R^2}{4}\right) - \rho_2 p_1^d\ {}_0F_1\left(\frac{d+2}{2}; -\frac{q^2p_1^2R^2}{4}\right) + \rho_2 p_2^d\ {}_0F_1\left(\frac{d+2}{2}; -\frac{q^2p_2^2R^2}{4}\right)\right)^2}{\left(\rho_1 p_1^d - \rho_2 p_1^d + \rho_2 p_2^d\right)^2}$$

We use $\rho_3 = 0, p_2 = 1, p_1 = p, \rho_1 = 1, \rho_2 = \rho$ to rewrite

$$P(q) = \frac{\left(p^d\ {}_0F_1\left(\frac{d+2}{2}; -\frac{q^2p^2R^2}{4}\right) - \rho p^d\ {}_0F_1\left(\frac{d+2}{2}; -\frac{q^2p^2R^2}{4}\right) + \rho\ {}_0F_1\left(\frac{d+2}{2}; -\frac{q^2R^2}{4}\right)\right)^2}{(p^d - \rho p^d + \rho)^2}$$

$$= \frac{p^{2d}\ {}_0F_1^2\left(\frac{d+2}{2}; -\frac{q^2p^2R^2}{4}\right) - 2p^{2d}\rho\ {}_0F_1^2\left(\frac{d+2}{2}; -\frac{q^2p^2R^2}{4}\right)}{(p^d - \rho p^d + \rho)^2}$$

$$+ \frac{2p^d\rho\ {}_0F_1\left(\frac{d+2}{2}; -\frac{q^2p^2R^2}{4}\right)\ {}_0F_1\left(\frac{d+2}{2}; -\frac{q^2R^2}{4}\right) + \rho^2p^{2d}\ {}_0F_1^2\left(\frac{d+2}{2}; -\frac{q^2p^2R^2}{4}\right)}{(p^d - \rho p^d + \rho)^2}$$

$$+ \frac{-2\rho^2p^d\ {}_0F_1\left(\frac{d+2}{2}; -\frac{q^2p^2R^2}{4}\right)\ {}_0F_1\left(\frac{d+2}{2}; -\frac{q^2R^2}{4}\right) + \rho^2\ {}_0F_1^2\left(\frac{d+2}{2}; -\frac{q^2R^2}{4}\right)}{(p^d - \rho p^d + \rho)^2}$$

For $d = 1$, the hypergeometric functions are given by

$${}_0F_1^2\left(\frac{3}{2}; -\frac{q^2p_i^2R^2}{4}\right) = \sum_{n=0}^{\infty}\frac{4^n}{(\mathrm{n}+1)}\frac{1}{\left(\frac{3}{2}\right)_n n!}\left(-\frac{q^2p_i^2R^2}{4}\right)^n$$

$${}_0F_1\left(\frac{3}{2}; -\frac{q^2p_i^2R^2}{4}\right)\ {}_0F_1\left(\frac{3}{2}; -\frac{q^2p_j^2R^2}{4}\right) = \sum_{n=0}^{\infty}\left(-\frac{q^2R^2}{4(z+1)^2}\right)^n\sum_{m=0}^{n}\frac{p^{2m}}{\left(\frac{3}{2}\right)_m\left(\frac{3}{2}\right)_{n-m} m!\,(n-m)!}$$

For $d = 2$, the hypergeometric functions are given by

$${}_0F_1^2\left(2; -\frac{q^2p^2R^2}{4}\right) = \sum_{n=0}^{\infty}\frac{4\left(n+\frac{1}{2}\right)(2\mathrm{n})!}{(\mathrm{n}+2)!\,n!}\frac{1}{(2)_n n!}\left(-\frac{q^2p^2R^2}{4}\right)^n$$

$${}_0F_1\left(2; -\frac{q^2R^2}{4}\right)\ {}_0F_1\left(2; -\frac{q^2p^2R^2}{4}\right) = \sum_{n=0}^{\infty}\left(-\frac{q^2R^2}{4}\right)^n\sum_{m=0}^{n}\frac{p^{2m}}{(2)_m(2)_{n-m}m!\,(n-m)!}$$

For $d = 3$, the hypergeometric functions are given by

$$ {}_0F_1^2\left(\frac{5}{2}; -\frac{q^2p^2R^2}{4}\right) = \sum_{n=0}^{\infty} \frac{6\cdot 4^n}{(\mathrm{n}+3)(\mathrm{n}+2)} \frac{1}{\left(\frac{5}{2}\right)_n n!} \left(-\frac{q^2p^2R^2}{4}\right)^n $$

$$ {}_0F_1\left(\frac{5}{2}; -\frac{q^2p^2R^2}{4}\right) {}_0F_1\left(\frac{5}{2}; -\frac{q^2R^2}{4}\right) = \sum_{n=0}^{\infty} \left(-\frac{q^2R^2}{4}\right)^n \sum_{m=0}^{n} \frac{p^{2m}}{\left(\frac{5}{2}\right)_m \left(\frac{5}{2}\right)_{n-m} m!\,(n-m)!} $$

2. Averages over powers and trigonometric functions

We consider the Schulz-Zimm distribution:

$$ \langle f(x)\rangle_X = \int_0^{\infty} f(x)x^s h(x)dx $$

$$ h(x) = \frac{(z_X+1)^{z_X+s+1} x^{z_X}}{X^{z_X+s+1}\Gamma(z_X+s+1)} \exp\left[-(z_X+1)\frac{x}{X}\right] $$

$$ z_X = \frac{1-\sigma_X^2}{\sigma_X^2} $$

with σ_X the relative standard deviation. With $u = \frac{X}{z+1}$ we have

$$ \langle x^n\rangle = \frac{\Gamma[z+s+n+1]}{\Gamma[z+s+1]} u^n $$

$$ \langle x^{-n}\cos(ax)\rangle = \frac{\Gamma[z+s-n+1]}{\Gamma[z+s+1]} u^{-n} \frac{\cos[(z+s-n+1)\arctan(au)]}{(1+a^2u^2)^{\frac{z+s-n+1}{2}}} $$

$$ \langle x^{-n}\sin(ax)\rangle = \frac{\Gamma[z+s-n+1]}{\Gamma[z+s+1]} u^{-n} \frac{\sin[(z+s-n+1)\arctan(au)]}{(1+a^2u^2)^{\frac{z+s-n+1}{2}}} $$

$$ \langle x^{-n}(\cos(ax))^2\rangle = \frac{\Gamma[z+s-n+1]}{\Gamma[z+s+1]} \frac{1}{2} u^{-n} \left(1 + \frac{\cos[(z+s-n+1)\arctan(2au)]}{(1+4a^2u^2)^{\frac{z+s-n+1}{2}}}\right) $$

$$ \langle x^{-n}\cos(ax)\sin(ax)\rangle = \frac{\Gamma[z+s-n+1]}{\Gamma[z+s+1]} \frac{1}{2} u^{-n} \left(\frac{\sin[(z+s-n+1)\arctan(2au)]}{(1+4a^2u^2)^{\frac{z+s-n+1}{2}}}\right) $$

$$\langle x^{-n}(\sin(ax))^2\rangle = \frac{\Gamma[z+s-n+1]}{\Gamma[z+s+1]}\frac{1}{2}u^{-n}\left(1-\frac{\cos[(z+s-n+1)\arctan(2au)]}{(1+4a^2u^2)^{\frac{z+s-n+1}{2}}}\right)$$

In case for the averages over the trigonometric functions we have for all cases $n > 1$. Therefore, the ratio of Gamma functions can be expressed as

$$\frac{\Gamma[z-n+1]}{\Gamma[z+1]} = \prod_{i=-n}^{-1}\frac{1}{\mathrm{z+i+1}} = \frac{1}{\mathrm{z(z-1)\ldots(z-n+1)}}$$

3. Q-limits of series and asymptotic expansions

Depending on the argument z, the scattering amplutides and formfactors are computed via the series or asymptotic expansion. Of particular interest is the leading term of the non-oscillating part of the asymptotic expansion in the limit $z \to \infty$, which corresponds to the Porod-asymptote. Since the Porod-asymptote is a particular simple form $c_4 q_{i,j}^{-4}$ and usually applies to a large number of pixels (i,j) in the high-q area of the detector, we derive Porod-asymptotes for the anisotropic cases as they considerably increase computational speed.

Therefore, the formfactor is calculated for three regimes

1. Regime I: $0 < q \leq q'_{\mathrm{lim}} \cong 1$, series expansion
2. Regime II: $q'_{\mathrm{lim}} < q \leq q''_{\mathrm{lim}}$, asymptotic expansion
3. Regime III: $q''_{\mathrm{lim}} < q$, Porod asymptote

The limiting q-values can be obtained from the pre-calculated coefficients

3.1 Q-Limits of series expansions

Regime I/Regime II: The series expansions will eventually break down due to rounding errors. The value of q'_{lim} for which the series expansion

$$P(q) = \sum_{n=0}^{n_{max}} c_n q^{2n}$$

is still convergent is given by

$$q'_{\mathrm{lim}} = c_{\tilde{n}}^{-1/(2*\tilde{n})}$$

where $\tilde{n}$ is the first index n where $|c_n| < 10^{-100}$. If this does not occur until n_{max}, then $\tilde{n} = n_{max}$. In practice, typical values are $n_{max} \cong 100$.

For a given particle radius R, q'_{lim} is primarily dependent on the relative polydispersity σ. The following Table S-6 provides a comparison of pre-calculated q'_{lim} and actual values q'_{div} for the polydisperse sphere formfactor (Eq. S.1.2.56). Here, we extend to $n_{max} \cong 200$ to cover very large polydispersities. We observe that the pre-calculated values show good agreement with the observed limits, $q'_{\text{lim}} \cong q'_{\text{div}}$, such that both the pre-calculated value q'_{lim} and $\tilde{n}$ can be used to limit the series expansion to the convergence regime and to a maximum needed number of terms.

Table S-6:

σ	$\tilde{n}$	$c_{\tilde{n}}$	q'_{lim}	q'_{div}
0.95	200	1E93	0.59	0.52
0.90	200	1E75	0.65	0.66
0.8	200	1E35	0.82	0.83
0.7	200	1E-10	1.06	1.06
0.6	200	1E-61	1.42	1.4
0.5	165	1E-100	2.01	2.02
0.4	105	1E-100	2.99	3.04
0.3	75	1E-100	4.64	4.87
0.2	55	1E-100	8.11	8.23
0.15	50	1E-100	10.00	10.9
0.1	45	1E-100	12.92	14.1
0.05	42	1E-100	15.51	17.1
0.02	40	1E-100	17.78	17.8

Table S-7: Examples of cases summarized in Table S-6 at different polydispersities σ.

σ	c_n	$P(q)$
0.02	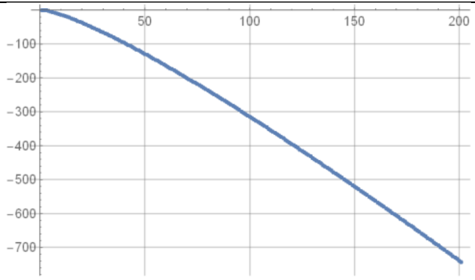 $\lvert c_n \rvert < 10^{-100}$ for $\tilde{n} = 40$, q'_{lim}=17.8	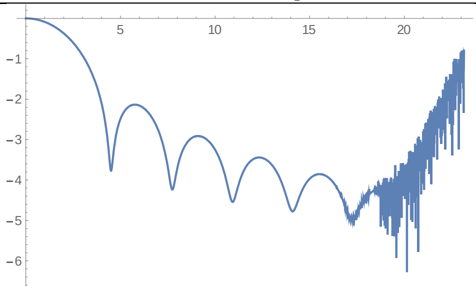 $q'_{\text{div}} = 17.8$
0.1	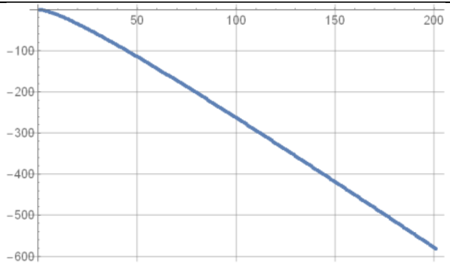 $\lvert c_n \rvert < 10^{-100}$ for $\tilde{n} = 45$, q'_{lim}=12.9	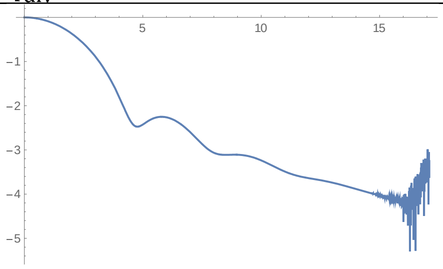 $q'_{\text{div}} = 14.1$

0.2	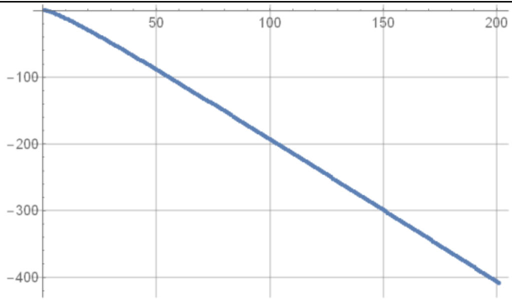 $|c_n| < 10^{-100}$ for $\widetilde{n} = 55$, q'_{lim}=8.1	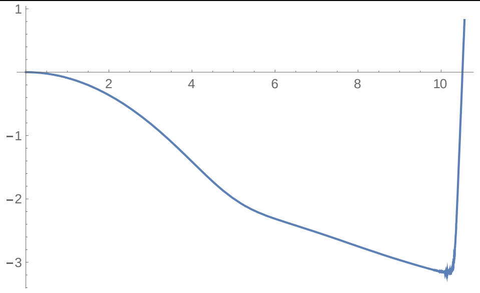 $q'_{\text{div}} = 8.2$
0.3	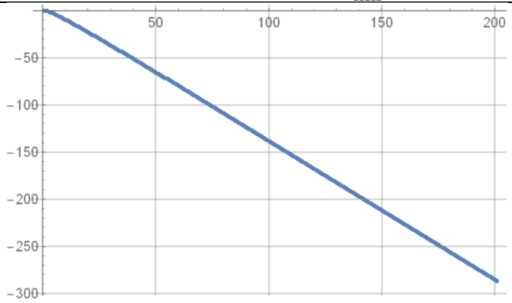 $|c_n| < 10^{-100}$ for $\widetilde{n} = 75$, q'_{lim}=4.6	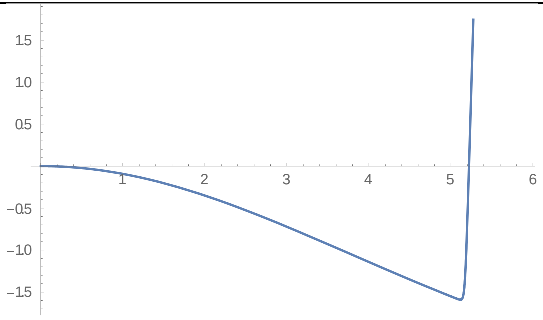 $q'_{\text{div}} = 4.9$
0.4	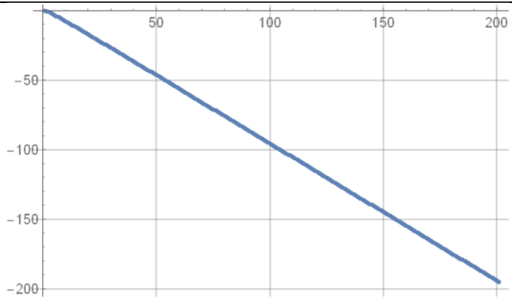 $|c_n| < 10^{-100}$ for $\widetilde{n} = 105$, q'_{lim}=3.0	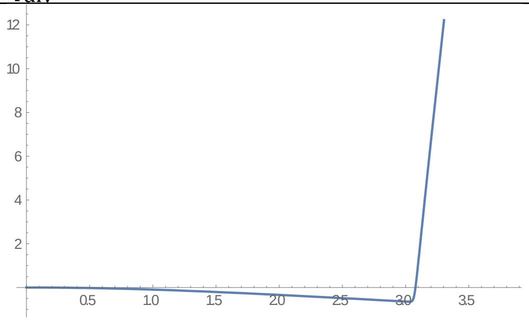 $q'_{\text{div}} = 3.04$
0.5	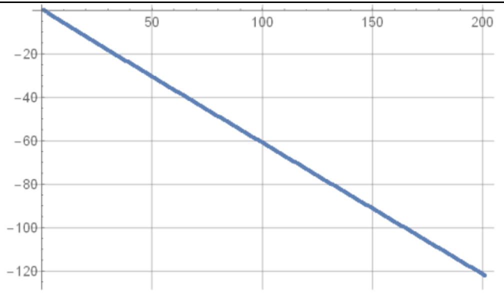 $|c_n| < 10^{-100}$ for $\widetilde{n} = 165$, q'_{lim}=2.0	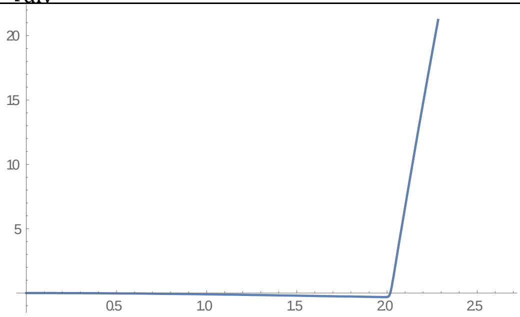 $q'_{\text{div}} = 2.05$
0.6	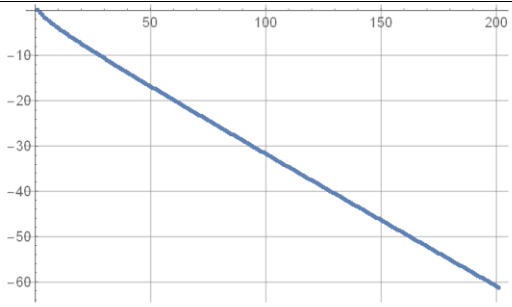 $|c_n| < 10^{-100}$ for $\widetilde{n} = n_{max} = 200$, q'_{lim}=1.4	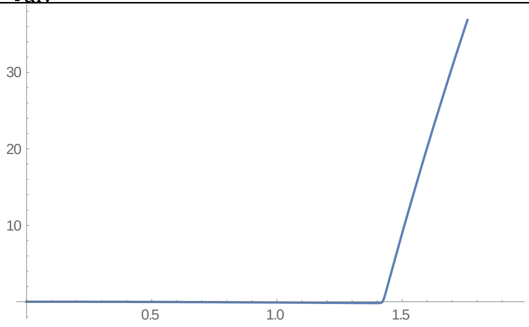 $q'_{\text{div}} = 1.45$

Therefore, the precalculation of coefficients yields

- n_{max} that is limited to the necessary value $\tilde{n}$ (e.g. just 55 for low polydispersities); it is straightforward to determine during calculation of the ascending coefficients,
- q'_{lim} to know during the calculation when to change from Regime I to Regime II;

Both values can be stored together with the coefficients c_n to compute the scattering curve.

For two-dimensional, anisotropic scattering patterns we have

$$P(q_x, q_y) = \sum_{n=0}^{n_{max}} \sum_{m=0}^{n} c_{n,m} q_x^m q_y^{n-m}$$

$$q'_{\text{x,lim}} = c_{\tilde{n},\tilde{n}}^{-1/(2*\tilde{n})}$$

$$q'_{\text{y,lim}} = c_{\tilde{n},0}^{-1/(2*\tilde{n})}$$

3.2 Q-Limits of asymptotic expansions

In Regime II we consider averages over trigonometric functions such as

$$\langle \frac{(\sin(ax))^2}{x^n} \rangle = \frac{\Gamma[z-n+1]}{\Gamma[z+s+1]} \frac{1}{2} u^{-n} \left(1 - \frac{\cos[(z+n+1)\arctan(2au)]}{(1+4a^2u^2)^{\frac{z-n+1}{2}}} \right)$$

These averages consist of a prefactor with the Porod-asymptote u^{-n}, multiplied with a sum containing an oscillatory function of the type

$$u(q) = \frac{\cos\left[a \arctan\left(\frac{2qR}{z+1}\right)\right]}{\left(1 + \left(\frac{2qR}{z+1}\right)^2\right)^{\frac{a}{2}}}$$

For large q the values of this function become very small, i.e. smaller than $u(q) < \varepsilon$, such that beyond a certain corresponding value q''_{lim} the oscillations are damped and the Porod asymptote is sufficient for calculations of the scattering curve. We thus calculate q''_{lim} from the condition $u(q) = \varepsilon$, and take into account that for large arguments $\arctan(x) \to \pi/2$

$$q''_{\text{lim}} = \frac{z+1}{2R} \left(\left| \left(\frac{\cos\left[\frac{a\pi}{2}\right]}{\varepsilon} \right)^{\frac{2}{a}} - 1 \right| \right)^{1/2}$$

with a reasonable value $\varepsilon = 0.01$.

As an example, we take the formfactor for a polydisperse sphere (Eq. S.1.2.57) with $R = 1$, $a = z + 1$ and $z = (1 - \sigma^2)/\sigma^2$. The following Table provides comparisons between calculated values of q''_{lim} and observed values q''_{osc} where oscillations are completely damped, and the formfactor enter the Porod regime.

Table S-8: Comparison of calculated (q''_{lim}) and observed (q''_{osc}) values for the transition from Regime II with formfactor oscillations to the Porod Regime III. The plots show lg(P(q)) as a function of q.

$P(q)$	
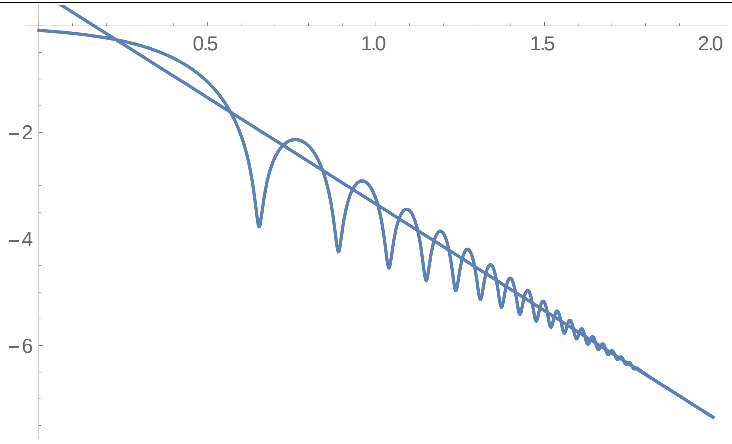	$\sigma = 0.02$<br>$q''_{\mathrm{lim}} = 76.0$<br>$\lg(q''_{\mathrm{osc}}) = 1.88$<br>$q''_{\mathrm{osc}} = 75.9$
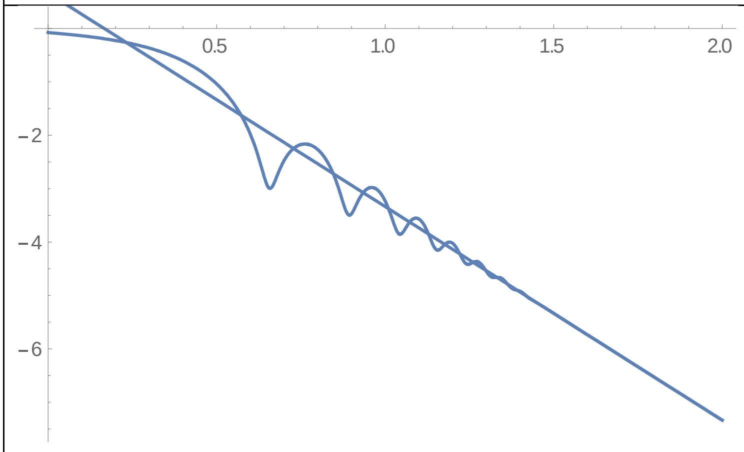	$\sigma = 0.05$<br>$q''_{\mathrm{lim}} = 30.7$<br>$\lg(q''_{\mathrm{osc}}) = 1.49$<br>$q''_{\mathrm{osc}} = 30.9$

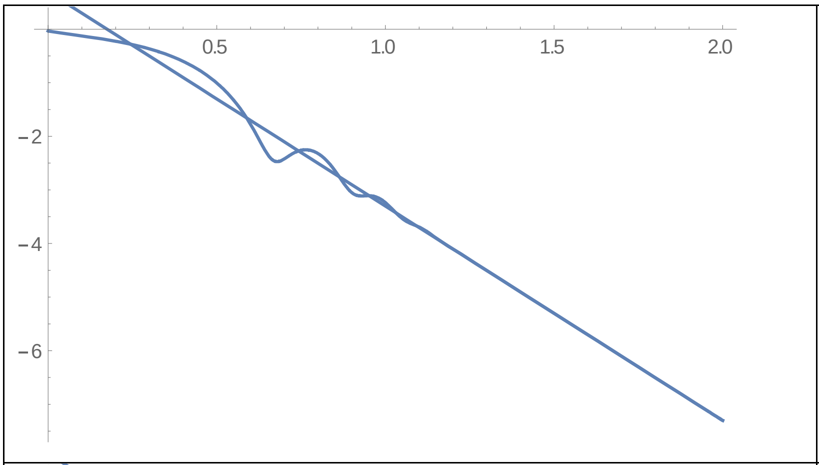

$\sigma = 0.10$

$q''_{\mathrm{lim}} = 15.9$

$\lg(q''_{\mathrm{osc}}) = 1.20$

$q''_{\mathrm{osc}} = 15.8$

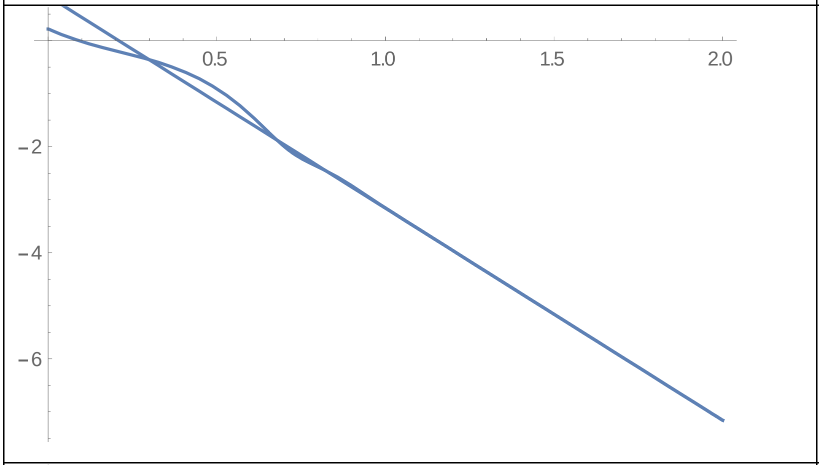

$\sigma = 0.20$

$q''_{\mathrm{lim}} = 12.1$

$\lg(q''_{\mathrm{osc}}) = 1.08$

$q''_{\mathrm{osc}} = 12.0$

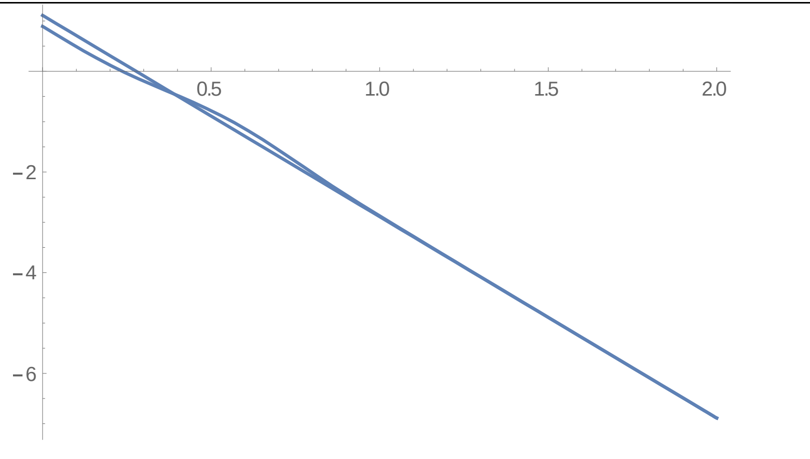

$\sigma = 0.30$

$q''_{\mathrm{lim}} = 6.17$

$\lg(q''_{\mathrm{osc}}) = 0.78$

$q''_{\mathrm{osc}} = 6.03$

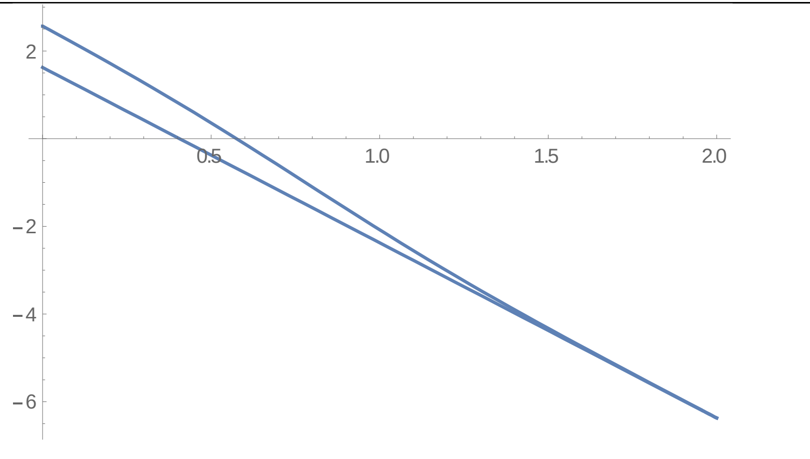

$\sigma = 0.40$

$q''_{\mathrm{lim}} = 23.6$

$\lg(q''_{\mathrm{osc}}) = 1.37$

$q''_{\mathrm{osc}} = 23.44$

In effect, the limiting q-values $q'_{\lim}$ and $q''_{\lim}$ can be pre-calculated such that the calculations can be performed in each of the convergence regimes. If the scattering intensity decays into a flat baseline at high q, a further preset $q'''_{\lim}$ could be provided to save computation time.

4. Isotropic Scattering Amplitudes and Formfactors

4.1 Spheres

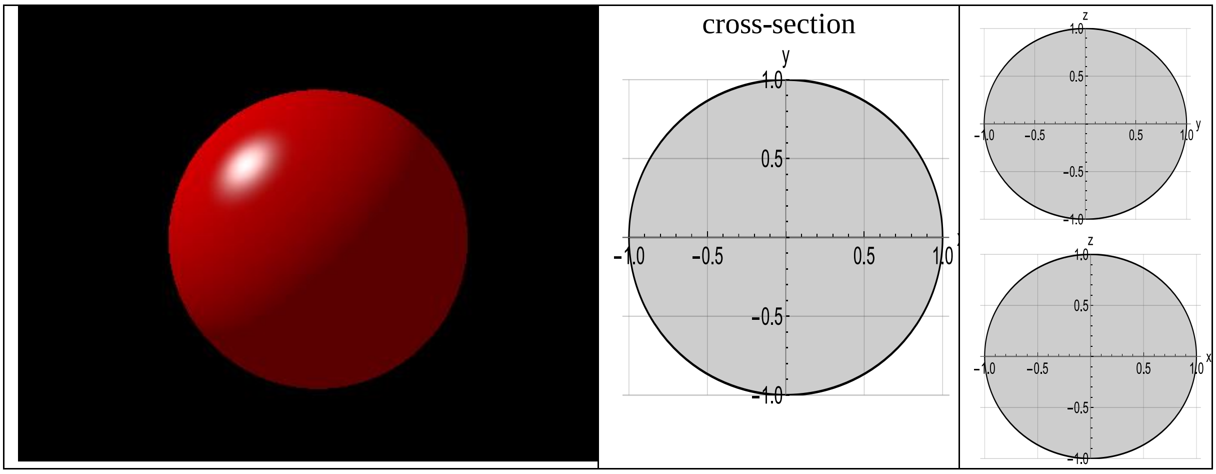

Fig. S2: Sphere with cross-sections in the (*x*,*y*)-, (*x*,*z*)-, and (*y*,*z*)-planes.

4.1.1. Formfactor P(q)

Integral representation. Conventionally, the formfactor of polydisperse spheres is calculated as (S.4.1.1.1)

$$\langle P^{(3)}(q)\rangle_R = 9\left\langle\left(\frac{\sin(qR) - qR\cos(qR)}{(qR)^3}\right)^2\right\rangle_R = 9\int_0^{\infty}\left(\frac{\sin(qR) - qR\cos(qR)}{(qR)^3}\right)^2 R^s h(R)dR$$

with $s = 2d = 6$.

Regime I. With $\langle P^{(3)}(q)\rangle_R$ from Table I we have **(S.4.1.1.2)**

$$\langle P^{(3)}(q)\rangle_R = \sum_{n=0}^{\infty} p_n^{(3)} \frac{(z+s+1)_{2n}}{\left(\frac{5}{2}\right)_n n!}\left(-\frac{q^2R^2}{4(z+1)^2}\right)^n$$

$$= \sum_{n=0}^{\infty} \frac{6\cdot 4^n}{(\mathrm{n}+3)(\mathrm{n}+2)} \frac{(z+s+1)_{2n}}{\left(\frac{5}{2}\right)_n n!}\left(-\frac{q^2R^2}{4(z+1)^2}\right)^n$$

$$= \sum_{n=0}^{\infty} c_n q^{2n}$$

with the desired q-independent coefficients c_n. ***Sphere, polydisperse, P(q)***

Regime II. The asymptotic expansions in terms of trigonometric functions are given by **(S.4.1.1.3)**

$$\langle P^{(3)}(q)\rangle_R = \langle\frac{(\cos(qR))^2}{(qR)^4}\rangle_R - \langle\frac{\sin(qR)\cos(qR)}{(qR)^5}\rangle_R + \langle\frac{(\sin(qR))^2}{(qR)^6}\rangle_R$$

The averaged trigonometric functions are

$$\langle\frac{(\cos(qR))^2}{(qR)^4}\rangle_R = \frac{\Gamma[z+s-3]}{2\Gamma[z+s+1]} x^{-4}\left(1+\frac{\cos[(z+s-3)\arctan(2x)]}{(1+4x^2)^{\frac{z+s-3}{2}}}\right)$$

$$\langle\frac{\sin(qR)\cos(qR)}{(qR)^5}\rangle_R = \frac{\Gamma[z+s-4]}{2\Gamma[z+s+1]} x^{-5}\left(\frac{\sin[(z+s-4)\arctan(2x)]}{(1+4x^2)^{\frac{z+s-4}{2}}}\right)$$

$$\langle\frac{(\sin(qR))^2}{(qR)^6}\rangle_R = \frac{\Gamma[z+s-5]}{2\Gamma[z+s+1]} x^{-6}\left(1-\frac{\cos[(z+s-5)\arctan(2x)]}{(1+4x^2)^{\frac{z+s-5}{2}}}\right)$$

with $x = \frac{qR}{z+1}$. Since $\frac{\Gamma[z+s-n]}{\Gamma[z+s+1]} = \prod_{i=0}^{n}\frac{1}{(z+s-i)}$ the evaluation of Gamma functions is not required.

Regime III. The non-oscillating part of the asymptotic expansion or equivalently the Porod-asymptote is obtained from the leading cosine term **(S.4.1.1.4)**

$$\lim_{q\to\infty} P(q) = \frac{9(z+s+1)^4}{2(\mathrm{z+s})(\mathrm{z+s}-1)(\mathrm{z+s}-2)(\mathrm{z+s}-3)}\frac{1}{(qR)^4}$$

It can alternatively be derived from the Porod-relation **(S.4.1.1.5)**

$$\lim_{q\to\infty} P(q) = \frac{2\pi}{q^4}\langle\frac{A}{V^2}\rangle = \frac{(z+s+1)^4}{(\mathrm{z+s})(\mathrm{z+s}-1)(\mathrm{z+s}-2)(\mathrm{z+s}-3)}\frac{2\pi}{q^4}\frac{A}{V^2}$$

With the area of a sphere $A = 4\pi R^2$ and the volume $V = \frac{4\pi}{3}R^3$ we obtain the same equation (S.4.1.1.4).

The following graph shows that the series expansion (Regime I), the asymptotic expansion (Regime) and the Porod-Regime (Regime III) well overlap, such that the formfactor can be evaluated over the complete q-range.

Mathematica code implementation:

```
R=1;
sigma=0.1;
z=(1-sigma*sigma)/(sigma*sigma);
area=4*Pi*R*R;
vol=4*Pi*R*R*R/3;
nmax=70;
q=10^lq;
qs=Sqrt[qx*qx+qy*qy];
qr=(q*R)/(z+1);
qsr=(qs*R)/(z+1);
fxp=(Gamma[z-3]/(2*Gamma[z+1]))*(qr^(-4))*(1+Cos[(z-3)*ArcTan[2*qr]]/((1+4*qr*qr)^((z-3)/2)));
fyp=(Gamma[z-4]/(2*Gamma[z+1]))*(qr^(-5))*Sin[(z-4)*ArcTan[2*qr]]/((1+4*qr*qr)^((z-4)/2));
fzp=(Gamma[z-5]/(2*Gamma[z+1]))*(qr^(-6))*(1-Cos[(z-5)*ArcTan[2*qr]]/((1+4*qr*qr)^((z-5)/2)));
Pqq=9*(fxp-2*fyp+fzp);
apor=Gamma[z-3]*((z+1)^4)/Gamma[z+1];
Pqpor=apor*2*Pi*area/((q^4)*vol*vol);
Pq1=Sum[6*(4^n)*Pochhammer[z+1,2*n]*((-q*q*R*R/(4*(z+1)*(z+1)))^n)/((n+3)*(n+2)*Pochhammer[5/2,n]*(n!)),{n, 0,
nmax}];
Pq1s=Sum[6*(4^n)*Pochhammer[z+1,2*n]*((-qs*qs*R*R/(4*(z+1)*(z+1)))^n)/((n+3)*(n+2)*Pochhammer[5/2,n]*(n!)),{n, 0,
nmax}];
lim=1.5;
pl2=Plot[Log[10,Pq1], {lq , -1,lim}, PlotRange->{-6,1}, GridLines->{Automatic}, LabelStyle->Directive[Black,16],AxesLabel-
>{"log q","lg P(q)"}, AxesOrigin->{-1,-6},TicksStyle->Directive[Black,12],PlotStyle->{Black,Dashed}] ;
pl1=Plot[Log[10,Pqq], {lq , -1,lim}, PlotRange->{-6,1}, GridLines->{Automatic}, LabelStyle->Directive[Black,16],AxesLabel-
>{"log q","lg P(q)"}, AxesOrigin->{-1,-6},TicksStyle->Directive[Black,12],PlotStyle->{Blue}] ;
pl3=Plot[Log[10,Pqpor], {lq , -1,lim}, PlotRange->{-6,1}, GridLines->{Automatic}, LabelStyle->Directive[Black,16],AxesLabel-
>{"log q","lg P(q)"}, AxesOrigin->{-1,-6},TicksStyle->Directive[Black,10],PlotStyle->{Red}] ;
Show[pl3,pl1,pl2]
lims=12;
pl4=DensityPlot[Log[10,Pq1s],{qx, -lims, lims}, {qy, -lims, lims}, PlotRange->{-8,0}, PlotPoints->50, PlotLegends->Automatic,
LabelStyle->Directive[Black,12],AxesLabel->Automatic]
```

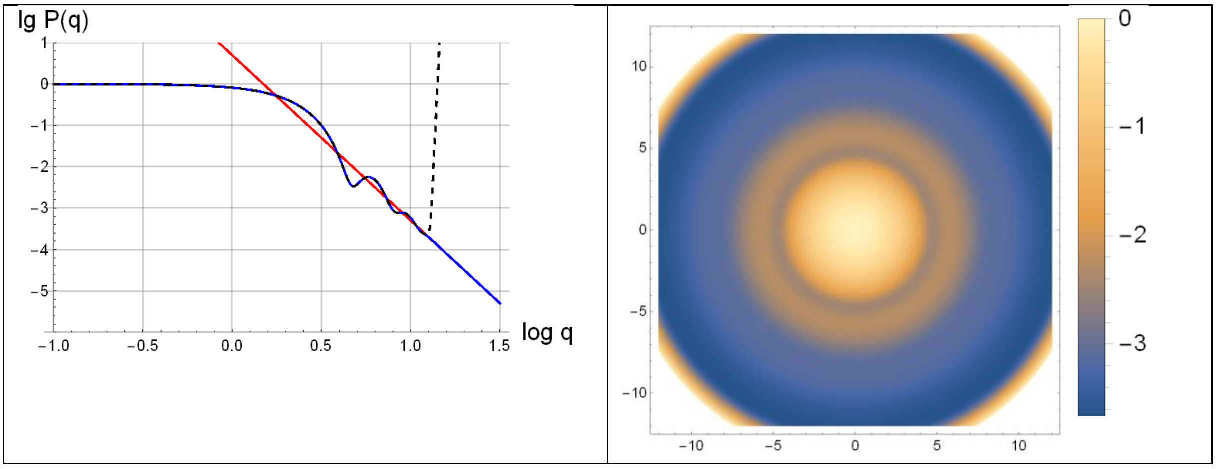

Fig. S3: Formfactor of polydisperse spheres.

4.1.2. Scattering amplitude F(q)

Integral representation.

$$\langle F^{(3)}(q)\rangle_R^2 = \langle\frac{\sin(qR) - qR\cos(qR)}{(qR)^3}\rangle_R^2 = \left(\int_0^\infty \frac{\sin(qR) - qR\cos(qR)}{(qR)^3} R^s h(R) dR\right)^2$$

with $s = d = 3$.

Regime I. (S.4.1.2.1)

$$\langle F^{(3)}(q)\rangle_R^2 = \sum_{n=0}^{\infty}\left(-\frac{q^2R^2}{4(z+1)^2}\right)^n f_n^{(3)}$$

$$= \frac{9\pi}{16}\sum_{n=0}^{\infty}\left(-\frac{q^2R^2}{4(z+1)^2}\right)^n \sum_{m=0}^{n}\frac{(z_r+s+1)_{2(n-m)}(z_r+s+1)_{2m}}{\Gamma\left[n-m+\frac{5}{2}\right]\Gamma\left[m+\frac{5}{2}\right](n-m)!\,m!}$$

$$= \frac{9\pi}{16}\sum_{n=0}^{\infty} c_n q^2$$

Sphere, polydisperse, F(q)

$$c_n = \left(-\frac{q^2R^2}{4(z+1)^2}\right)^n \sum_{m=0}^{n}\frac{(z_r+s+1)_{2(n-m)}(z_r+s+1)_{2m}}{\Gamma\left[n-m+\frac{5}{2}\right]\Gamma\left[m+\frac{5}{2}\right](n-m)!\,m!}$$

Regime II. (S.4.1.2.2)

$$\langle F^{(3)}(q)\rangle_R^2 = 9\left(\langle\frac{\sin(qR)}{(qR)^3}\rangle_R - \langle\frac{\cos(qR)}{(qR)^2}\rangle_R\right)^2$$

with

$$\langle\frac{\cos(qR)}{(qR)^2}\rangle = \frac{\Gamma[z+s-1]}{\Gamma[z+s+1]}\left(\frac{qR}{z+1}\right)^{-2}\frac{\cos\left[(z+s-1)\arctan\left(\frac{qR}{z+1}\right)\right]}{\left(1+\left(\frac{qR}{z+1}\right)^2\right)^{\frac{z+s-1}{2}}}$$

$$\langle\frac{\sin(qR)}{(qR)^3}\rangle = \frac{\Gamma[z+s-2]}{\Gamma[z+s+1]}\left(\frac{qR}{z+1}\right)^{-3}\frac{\sin\left[(z+s-2)\arctan\left(\frac{qR}{z+1}\right)\right]}{\left(1+\left(\frac{qR}{z+1}\right)^2\right)^{\frac{z+s-2}{2}}}$$

Mathematica code implementation:

```
R=1;
sigma=0.1;
z=(1-sigma*sigma)/(sigma*sigma);
nmax=70;
```

```
q=10^lq;
qs=Sqrt[qx*qx+qy*qy];
qr=(q*R)/(z+1);
qsr=(qs*R)/(z+1);
fxf=(Gamma[z-2]/(Gamma[z+1]))*(qr^(-3))*Sin[(z-2)*ArcTan[qr]]/((1+qr*qr)^((z-2)/2));
fyf=(Gamma[z-1]/(Gamma[z+1]))*(qr^(-2))*Cos[(z-1)*ArcTan[qr]]/((1+qr*qr)^((z-1)/2));
Fqq=9*((fxf-fyf)^2);
fff=Table[Sum[Pochhammer[z+1,2*(n-m)]*Pochhammer[z+1,2*m]/(Pochhammer[5/2,n-m]*Pochhammer[5/2,m]*((n-
m)!)*(m!)), {m, 0, n}],{n, 0, nmax}];
Fq1=Sum[(((-q*q*R*R/(4*(z+1)*(z+1)))^n)*fff[[n+1]]), {n, 0, nmax}];
Fq1s=Sum[(((-qs*qs*R*R/(4*(z+1)*(z+1)))^n)*fff[[n+1]]), {n, 0, nmax}];
lim=1.5;
pl2=Plot[Log[10,Fq1], {lq , -1,lim}, PlotRange->{-8,1}, GridLines->{Automatic}, LabelStyle->Directive[Black,16],AxesLabel-
>{"log q","lg F^2(q)"}, AxesOrigin->{-1,-8},TicksStyle->Directive[Black,12],PlotStyle->{Black,Dashed}] ;
pl1=Plot[Log[10,Fqq], {lq , -1,lim}, PlotRange->{-8,1}, GridLines->{Automatic}, LabelStyle->Directive[Black,16],AxesLabel-
>{"log q","lg F^2(q)"}, AxesOrigin->{-1,-8},TicksStyle->Directive[Black,12],PlotStyle->{Blue}] ;
Show[pl2,pl1]
lims=15;
pl4=DensityPlot[Log[10,Fq1s],{qx, -lims, lims}, {qy, -lims, lims}, PlotRange->{-8,0}, PlotPoints->50, PlotLegends->Automatic,
LabelStyle->Directive[Black,12],AxesLabel->Automatic]
```

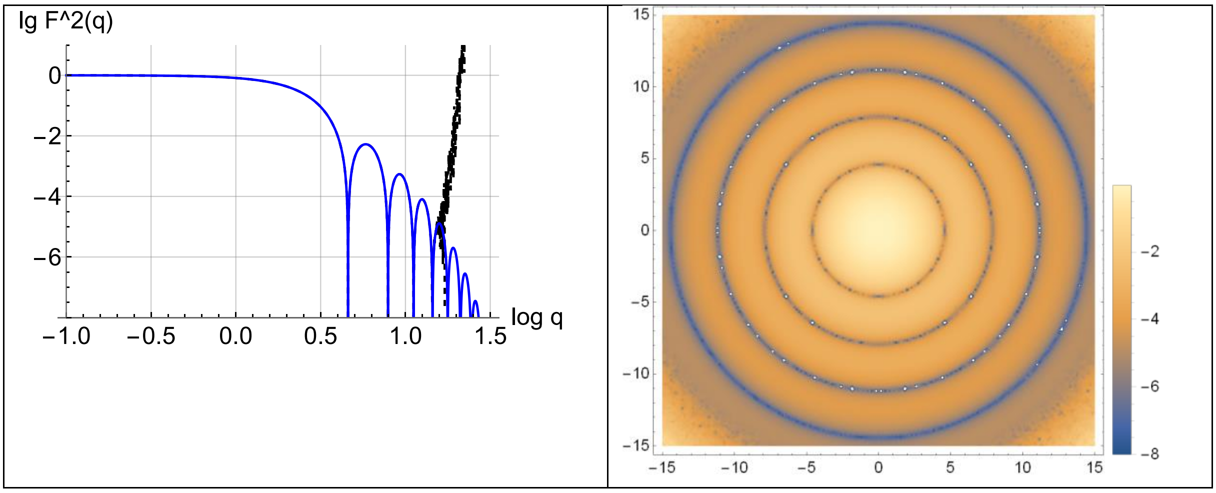

Fig. S4: Scattering amplitude of polydisperse spheres.

4.2 Biaxial Ellipsoids

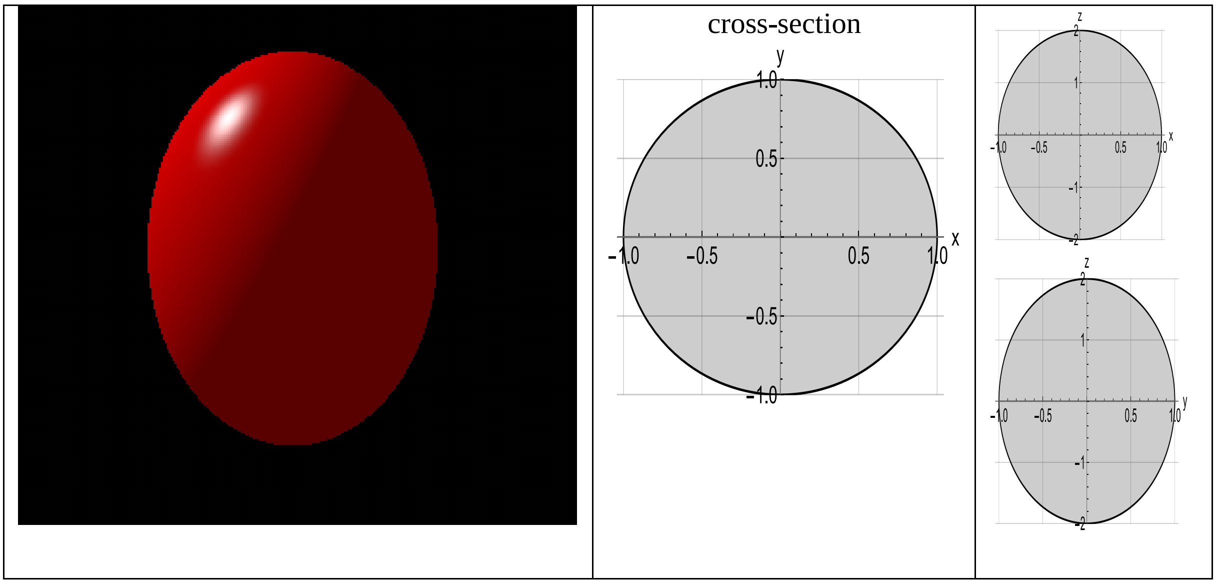

Fig. S5: Biaxial ellipsoid with cross-sections in the (*x*,*y*)-, (*x*,*z*)-, and (*y*,*z*)-planes.

4.2.1. Formfactor P(q)

Integral representation. Conventionally, the formfactor of polydisperse biaxial ellipsoid is calculated as **(S.4.2.1.1)**

$$\langle P(q)\rangle = 9\int_0^{\infty}\int_0^{\pi/2} \frac{\left(\sin\left(qR(\theta)\right) - qR(\theta)\cos\left(qR(\theta)\right)\right)^2}{\left(qR(\theta)\right)^6} R^s(\theta)h\left(R(\theta)\right)dR\sin\theta\, d\theta$$

$$R(\theta) = \sqrt{(a\sin\theta)^2 + (c\cos\theta)^2}$$

with $s = 6$.

The integral can be simplified by substituting $\cos\theta = x$ with $\frac{dx}{d\theta} = \frac{d(\cos\theta)}{d\theta} = -\sin\theta$ and therefore $\sin\theta\, d\theta = -d(\cos\theta) = -dx$. We further use $(\sin\theta)^2 = (1-(\cos\theta)^2)$, $\cos(0) = 1$, $\cos(\frac{\pi}{2}) = 0$ and $\varepsilon = c/a$ to obtain the integral **(S.4.2.1.2)**

$$\langle P(q)\rangle = 9\int_0^{\infty}\int_0^{1} \frac{\left(\sin\left(qR(x)\right) - qR(x)\cos\left(qR(x)\right)\right)^2}{\left(qR(x)\right)^6} R^s(x)h\left(R(x)\right)dRdx$$

$$R(x) = a(1 + (\varepsilon^2 - 1)x^2)^{1/2}$$

which requires fewer integration iterations and reduces the computing time by ca. 1/2.

Regime I. The series expansion for the formfactor at a given angle θ is given by

$$\langle P(q,\theta)\rangle = \sum_{n=0}^{\infty} p_n^{(3)} \frac{(z+s+1)_{2n}}{\left(\frac{5}{2}\right)_n n!}\left(-\frac{q^2R^2(\theta)}{4(z_r+1)^2}\right)^n$$

The formfactor needs to be integrated over the angle, which involves integrating the factors $R^{2n}(\theta)$ over the angle θ as

$$\langle R^{2n}(\theta)\rangle = \int_0^{\pi/2} R^{2n}(\theta)\sin\theta\, d\theta = a^{2n}\int_0^{\pi/2} ((\sin\theta)^2 + (\varepsilon\cos\theta)^2)^n \sin\theta\, d\theta$$

With the same substitution we obtain

$$e_n^{(\theta)} = a^{2n}\int_0^1 (1+(\varepsilon^2-1)x^2)^n dx = a^{2n}\sum_{m=0}^{n}\binom{n}{m}(\varepsilon^2-1)^m\int_0^1 x^{2m}dx$$

$$= a^{2n}\sum_{m=0}^{n}\binom{n}{m}\frac{(\varepsilon^2-1)^m}{2m+1} = a^{2n}e_n^{(\theta)}$$

$$e_n^{(\theta)} = \sum_{m=0}^{n}\frac{n!}{m!\,(n-m)!}\frac{(\varepsilon^2-1)^m}{2m+1}$$

Therefore, we have for the averaged series expansion **<u>(S.4.2.1.3)</u>**

$$\langle P(q)\rangle = \sum_{n=0}^{\infty} p_n^{(3)} e_n^{(\theta)} \frac{(z+s+1)_{2n}}{\left(\frac{5}{2}\right)_n n!}\left(-\frac{q^2a^2}{4(z+1)^2}\right)^n = \sum_{n=0}^{\infty} c_n q^{2n}$$

$$c_n = p_n^{(3)} e_n^{(\theta)} \frac{(z+s+1)_{2n}}{\left(\frac{5}{2}\right)_n n!}\left(-\frac{a^2}{4(z+1)^2}\right)^n$$

Biaxial Ellipsoid, polydisperse, P(q), isotropic

Simplification:

$$c_n = \frac{6\cdot 4^n}{(\mathrm{n}+3)(\mathrm{n}+2)}\frac{(z+s+1)_{2n}}{\left(\frac{5}{2}\right)_n n!}\left(-\frac{a^2}{4(z+1)^2}\right)^n \sum_{m=0}^{n}\frac{n!}{m!\,(n-m)!}\frac{(\varepsilon^2-1)^m}{2m+1}$$

$$= \frac{6}{(\mathrm{n}+3)(\mathrm{n}+2)}\frac{(z+s+1)_{2n}}{\left(\frac{5}{2}\right)_n}\left(-\frac{a^2}{(z+1)^2}\right)^n \sum_{m=0}^{n}\frac{1}{m!\,(n-m)!}\frac{(\varepsilon^2-1)^m}{2m+1}$$

Regime II. The asymptotic expansions in terms of trigonometric functions in the polydisperse need a numerical integration and are **(S.4.2.1.4)**

$$\langle P(q)\rangle = 9\int_0^{\pi/2}\left[\langle\frac{\left(\sin\left(qR(\theta)\right)\right)^2}{(qR(\theta))^6}\rangle - 2\langle\frac{\sin\left(qR(\theta)\right)\cos\left(qR(\theta)\right)}{(qR(\theta))^5}\rangle + \langle\frac{\left(\cos\left(qR(\theta)\right)\right)^2}{(qR(\theta))^4}\rangle\right]\sin\theta\, d\theta$$

This integration is not needed, because series expansion from Regime I overlaps with Porod-asymptote in Regime III.

Regime III. Similar as for the sphere Porod asymptote, it can be derived from the leading cosine function as **(S.4.2.1.5)**

$$\lim_{q\to\infty} P(q) = \frac{9}{2}\frac{\Gamma[z+s-3]}{\Gamma[z+s+1]}\frac{(z+1)^4}{q^4}\langle\frac{1}{(R(\theta))^4}\rangle$$

with

$$\int_0^{\pi/2}\frac{1}{(R(\theta))^4}\sin\theta\, d\theta = \begin{cases}\frac{1}{2}\left(\frac{1}{1+a}+\frac{1}{\sqrt{a}}\arctan\left(\sqrt{a}\right)\right) & a=\varepsilon^2-1 \quad c>a, prolate\\ \frac{1}{2}\left(\frac{1}{1-a}+\frac{1}{\sqrt{a}}\operatorname{arctanh}\left(\sqrt{a}\right)\right) & a=1-\varepsilon^2 \quad c<a, oblate\end{cases}$$

The same equation can again be derived via the surface-to-volume ratio.

$$\lim_{q\to\infty} P(q) = \frac{2\pi}{q^4}\langle\frac{A}{V^2}\rangle = \frac{(z+s+1)^4}{(\mathrm{z+s})(\mathrm{z+s-1})(\mathrm{z+s-2})(\mathrm{z+s-3})}\frac{2\pi}{q^4}\frac{A}{V^2}$$

With the volume

$$V = \frac{4\pi a^2 c}{3}$$

and the surface area

$$A = \begin{cases}2\pi a\left(a+\frac{c^2}{\sqrt{c^2-a^2}}\arcsin\left(\frac{\sqrt{c^2-a^2}}{c}\right)\right) & c>a \quad prolate\\ 2\pi a\left(a+\frac{c^2}{\sqrt{a^2-c^2}}\operatorname{arcsinh}\left(\frac{\sqrt{a^2-c^2}}{c}\right)\right) & c<a \quad oblate\end{cases}$$

In any case, the Porod-asymptote is obtained in closed analytical form.

Mathematica code implementation:

```
a=1;
c=2;
eps=c/a;
sigma=0.1;
z=(1-sigma*sigma)/(sigma*sigma);
```

```
area=2*Pi*a*(a+(c*c/Sqrt[(c*c-a*a)])*ArcSin[Sqrt[c*c-a*a]/c]);
vol=4*Pi*a*a*c/3;
nmax=70;
q=10^lq;
qs=Sqrt[qx*qx+qy*qy];
R=Sqrt[((a*Sin[theta])^2)+((c*Cos[theta])^2)];
qr=(q*R)/(z+1);
qrs=(qs*R)/(z+1);
fxp=(Gamma[z-3]/(2*Gamma[z+1]))*(qr^(-4))*(1+Cos[(z-3)*ArcTan[2*qr]]/((1+4*qr*qr)^((z-3)/2)));
fyp=(Gamma[z-4]/(2*Gamma[z+1]))*(qr^(-5))*Sin[(z-4)*ArcTan[2*qr]]/((1+4*qr*qr)^((z-4)/2));
fzp=(Gamma[z-5]/(2*Gamma[z+1]))*(qr^(-6))*(1-Cos[(z-5)*ArcTan[2*qr]]/((1+4*qr*qr)^((z-5)/2)));
Pqq=9*NIntegrate[(fxp-2*fyp+fzp)*Sin[theta], {theta, 0, Pi/2}];
apor=Gamma[z-3]*((z+1)^4)/Gamma[z+1];
Pqpor=apor*2*Pi*area/((q^4)*vol*vol);
eee=Table[Sum[Binomial[n,m]*((eps*eps-1)^(n-m))/(2*(n-m)+1), {m, 0, n}],{n, 0, nmax}];
Pq1=Sum[6*(4^n)*Pochhammer[z+1,2*n]*((-
q*q*a*a/(4*(z+1)*(z+1)))^n)*eee[[n+1]]/((n+3)*(n+2)*Pochhammer[5/2,n]*(n!)),{n, 0, nmax}];
Pq1s=Sum[6*(4^n)*Pochhammer[z+1,2*n]*((-
qs*qs*a*a/(4*(z+1)*(z+1)))^n)*eee[[n+1]]/((n+3)*(n+2)*Pochhammer[5/2,n]*(n!)),{n, 0, nmax}];
lim=1.5;
pl2=Plot[Log[10,Pq1], {lq , -1,lim}, PlotRange->{-6,1}, GridLines->{Automatic}, LabelStyle->Directive[Black,16],AxesLabel-
>{"log q","lg P(q)"}, AxesOrigin->{-1,-6},TicksStyle->Directive[Black,12],PlotStyle->{Black,Dashed}] ;
pl1=Plot[Log[10,Pqq], {lq , -1,lim}, PlotRange->{-6,1}, GridLines->{Automatic}, LabelStyle->Directive[Black,16],AxesLabel-
>{"log q","lg P(q)"}, AxesOrigin->{-1,-6},TicksStyle->Directive[Black,12],PlotStyle->{Blue}] ;
pl3=Plot[Log[10,Pqpor], {lq , -1,lim}, PlotRange->{-6,1}, GridLines->{Automatic}, LabelStyle->Directive[Black,16],AxesLabel-
>{"log q","lg P(q)"}, AxesOrigin->{-1,-6},TicksStyle->Directive[Black,10],PlotStyle->{Red}] ;
Show[pl2,pl1,pl3]
lims=8;
pl4=DensityPlot[Log[10,Pq1s],{qx, -lims, lims}, {qy, -lims, lims}, PlotRange->{-8,0}, PlotPoints->50, PlotLegends->Automatic,
LabelStyle->Directive[Black,12],AxesLabel->Automatic]
```

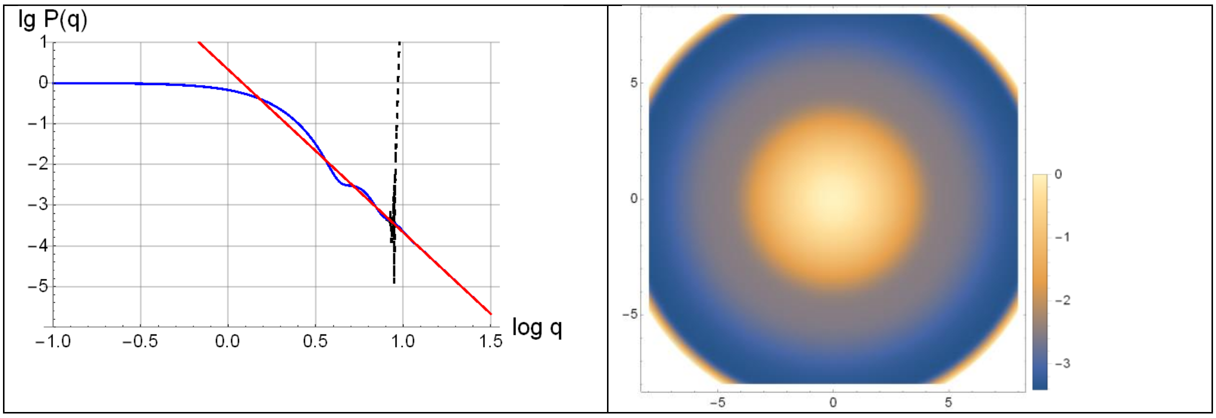

Fig. S6: Formfactor of a polydisperse isotropic biaxial ellipsoids.

4.2.2. Scattering Amplitude F(q)

Integral representation. **(S.4.2.2.1)**

$$\langle F(q)\rangle^2 = 9\int_0^{\pi/2}\left(\int_0^{\infty}\frac{\sin\big(qR(\theta)\big)-qR(\theta)\cos\big(qR(\theta)\big)}{\big(qR(\theta)\big)^3}R^s(\theta)h\big(R(\theta)\big)dr\right)^2\sin\theta\, d\theta$$

$$R(\theta)=\sqrt{(a\sin\theta)^2+(c\cos\theta)^2}$$

with $s=3$

Regime I: The angular dependent scattering amplitude is

$$\langle F(q,\theta)\rangle^2=\sum_{n=0}^{\infty} f_n^{(3)}\left(-\frac{q^2R^2(\theta)}{4(z+1)^2}\right)^n$$

The factor $\langle R^{2n}(\theta)\rangle$ has been calucated above as

$$\langle R^{2n}(\theta)\rangle = a^{2n}\sum_{l=0}^{n}\frac{n!}{l!\,(n-l)!}\frac{(\varepsilon^2-1)^l}{2l+1}=a^{2n}e_n^{(\theta)}$$

Therefore, the angular averaged scattering amplitude is **(S.4.2.2.2)**

$$\langle F(q)\rangle^2=\sum_{n=0}^{\infty} f_n^{(3)}e_n^{(\theta)}\left(-\frac{q^2a^2}{4(z+1)^2}\right)^n$$

$$=\frac{9\pi}{16}\sum_{n=0}^{\infty}\left(-\frac{q^2a^2}{4(z+1)^2}\right)^n\left(\sum_{m=0}^{n}\frac{(z+s+1)_{2(n-m)}(z+s+1)_{2m}}{\Gamma\left[n-m+\frac{5}{2}\right]\Gamma\left[m+\frac{5}{2}\right](n-m)!\,m!}\right)\left(\sum_{l=0}^{n}\frac{n!}{l!\,(n-l)!}\frac{(\varepsilon^2-1)^l}{2l+1}\right)$$

$$=\frac{9\pi}{16}\sum_{n=0}^{\infty}c_nq^2$$

Biaxial Ellipsoid, polydisperse, F(q), isotropic

$$c_n=\left(-\frac{a^2}{4(z+1)^2}\right)^n\left(\sum_{m=0}^{n}\frac{(z+s+1)_{2(n-m)}(z+s+1)_{2m}}{\Gamma\left[n-m+\frac{5}{2}\right]\Gamma\left[m+\frac{5}{2}\right](n-m)!\,m!}\right)\left(\sum_{l=0}^{n}\frac{n!}{l!\,(n-l)!}\frac{(\varepsilon^2-1)^l}{2l+1}\right)$$

Regime II: **(S.4.2.2.3)**

$$\langle F(q)\rangle^2=9\left[\langle\frac{\left(\sin\big(qR(\theta)\big)-qR(\theta)\cos\big(qR(\theta)\big)\right)}{q^3R^3(\theta)}\rangle\right]^2=9\left[\langle\frac{\sin(qR)}{q^3R^3}\rangle-\langle\frac{\cos(qR)}{q^2R^2}\rangle\right]^2$$

$$\langle F(q)\rangle^2=9\int_0^{\pi/2}\left(\langle\frac{\sin\big(qR(\theta)\big)}{q^3R^3(\theta)}\rangle-\langle\frac{\cos\big(qR(\theta)\big)}{q^2R^2}\rangle\right)^2\sin\theta\, d\theta$$

with

$$\langle\frac{\cos\big(qR(\theta)\big)}{(qR(\theta))^2}\rangle = \frac{\Gamma[z+s-1]}{\Gamma[z+s+1]}\left(\frac{qR(\theta)}{z+1}\right)^{-2}\frac{\cos\left[(z+s-1)\arctan\left(\frac{qR(\theta)}{z+1}\right)\right]}{\left(1+\left(\frac{qR(\theta)}{z+1}\right)^2\right)^{\frac{z+s-1}{2}}}$$

$$\langle\frac{\sin\big(qR(\theta)\big)}{(qR(\theta))^3}\rangle = \frac{\Gamma[z+s-2]}{\Gamma[z+s+1]}\left(\frac{qR(\theta)}{z+1}\right)^{-3}\frac{\sin\left[(z+s-2)\arctan\left(\frac{qR(\theta)}{z+1}\right)\right]}{\left(1+\left(\frac{qR(\theta)}{z+1}\right)^2\right)^{\frac{z+s-2}{2}}}$$

Mathematica code implementation:

```
a=1;
c=2;
eps=c/a;
sigma=0.1;
z=(1-sigma*sigma)/(sigma*sigma);
area=2*Pi*a*(a+(c*c/Sqrt[(c*c-a*a)])*ArcSin[Sqrt[c*c-a*a]/c]);
vol=4*Pi*a*a*c/3;
nmax=70;
q=10^lq;
qs=Sqrt[qx*qx+qy*qy];
R=Sqrt[((a*Sin[theta])^2)+((c*Cos[theta])^2)];
qr=(q*R)/(z+1);
qrs=(qs*R)/(z+1);
fxf=(Gamma[z-2]/(Gamma[z+1]))*(qr^(-3))*Sin[(z-2)*ArcTan[qr]]/((1+qr*qr)^((z-2)/2));
fyf=(Gamma[z-1]/(Gamma[z+1]))*(qr^(-2))*Cos[(z-1)*ArcTan[qr]]/((1+qr*qr)^((z-1)/2));
Fqq=9*NIntegrate[((fxf-fyf)^2)*Sin[theta], {theta, 0, Pi/2}];
fff=Table[Sum[Pochhammer[z+1,2*(n-m)]*Pochhammer[z+1,2*m]/(Pochhammer[5/2,n-m]*Pochhammer[5/2,m]*((n-
m)!)*(m!)), {m, 0, n}],{n, 0, nmax}];
eee=Table[Sum[Binomial[n,m]*((eps*eps-1)^(n-m))/(2*(n-m)+1), {m, 0, n}],{n, 0, nmax}];
Fq1=Sum[(((-q*q*a*a/(4*(z+1)*(z+1)))^n)*eee[[n+1]]*fff[[n+1]]), {n, 0, nmax}];
Fq1s=Sum[(((-qs*qs*a*a/(4*(z+1)*(z+1)))^n)*eee[[n+1]]*fff[[n+1]]), {n, 0, nmax}];
lim=1.5;
pl2=Plot[Log[10,Fq1], {lq , -1,lim}, PlotRange->{-6,1}, GridLines->{Automatic}, LabelStyle->Directive[Black,16],AxesLabel-
>{"log q","lg F^2(q)"}, AxesOrigin->{-1,-6},TicksStyle->Directive[Black,12],PlotStyle->{Black,Dashed}] ;
pl1=Plot[Log[10,Fqq], {lq , -1,lim}, PlotRange->{-6,1}, GridLines->{Automatic}, LabelStyle->Directive[Black,16],AxesLabel-
>{"log q","lg F^2(q)"}, AxesOrigin->{-1,-6},TicksStyle->Directive[Black,12],PlotStyle->{Blue}] ;
Show[pl2,pl1]
lims=8;
pl4=DensityPlot[Log[10,Fq1s],{qx, -lims, lims}, {qy, -lims, lims}, PlotRange->{-8,0}, PlotPoints->50, PlotLegends->Automatic,
LabelStyle->Directive[Black,12],AxesLabel->Automatic]
```

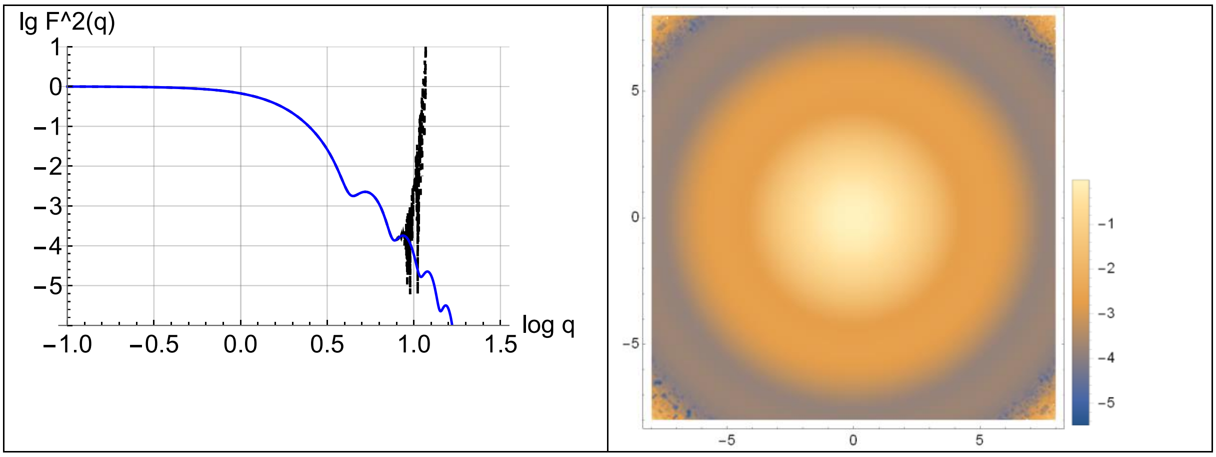

Fig. S7: Scattering amplitude of a polydisperse isotropic biaxial ellipsoids.

4.3 Triaxial Ellipsoids

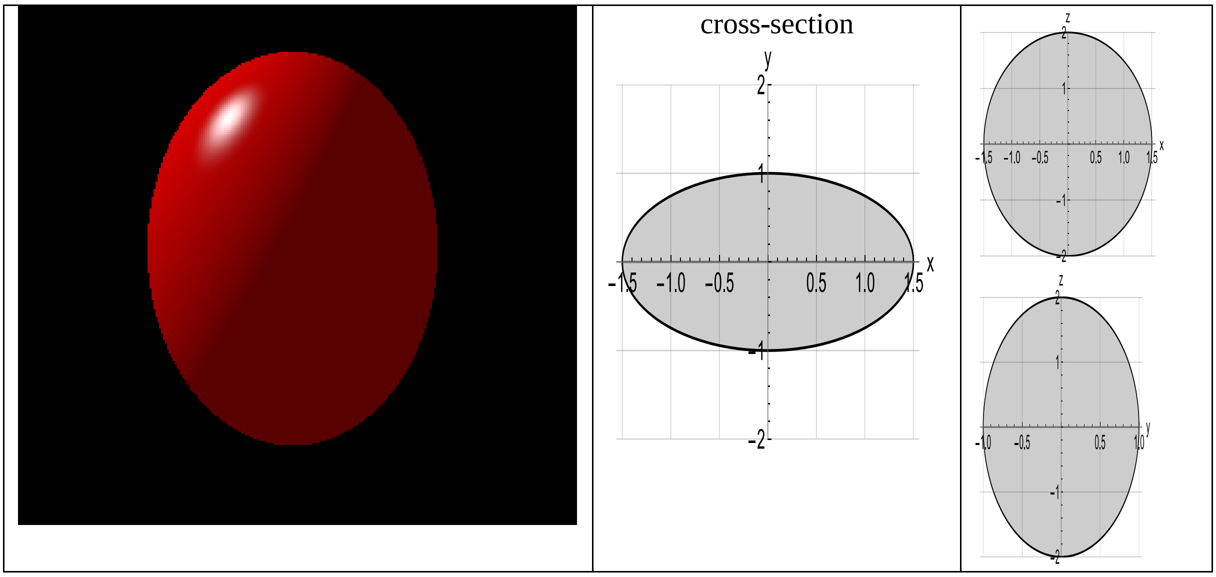

Fig. S8: Triaxial ellipsoid with cross-sections in the (*x*,*y*)-, (*x*,*z*)-, and (*y*,*z*)-planes.

4.3.1. Formfactor P(q)

Integral representation. Conventionally, the formfactor of a polydisperse triaxial ellipsoid calculated as **(S.4.3.1.1)**

$$\langle P(q)\rangle = \frac{18}{\pi}\int_0^{\infty}\int_0^{\pi/2}\int_0^{\pi/2} \frac{\left(\sin\left(qR(\phi,\theta)\right) - qR(\phi,\theta)\cos\left(qR(\phi,\theta)\right)\right)^2}{\left(qR(\phi,\theta)\right)^6} R^s\left((\phi,\theta)\right)h\left(R(\phi,\theta)\right)dR\sin\theta\, d\phi d\theta$$

$$R(\phi,\theta) = \sqrt{(a\sin\theta\cos\phi)^2 + (b\sin\theta\sin\phi)^2 + (c\cos\theta)^2}$$

with $s = 6$. The integral can be simplified by substitution as **(S.4.3.1.2)**

$$\langle P(q)\rangle = \frac{18}{\pi}\int_0^{\infty}\int_0^{\pi/2}\int_0^{1} \frac{\left(\sin\left(qR(\phi,x)\right) - qR(\phi,x)\cos\left(qR(\phi,x)\right)\right)^2}{\left(qR(\phi,x)\right)^6} R^s\left((\phi,x)\right)h\left(R(\phi,x)\right)dRdxd\phi$$

$$R(\phi,x) = \sqrt{(a^2(\cos\phi)^2 + a^2(\sin\phi)^2)(1-x^2) + c^2x^2}$$

where we note a printing error of the brackets in the often used equation in the book by Feigin and Svergun.

Regime I. The angular dependent series expansion for the formfactor is

$$\langle P(q,\phi,\theta)\rangle = \sum_{n=0}^{\infty} p_n^{(3)} \frac{(z+s+1)_{2n}}{\left(\frac{5}{2}\right)_n n!}\left(-\frac{q^2R^2(\phi,\theta)}{4(z+1)^2}\right)^n$$

We need to integrate the factors $R^{2n}(\phi,\theta)$ over the angles as

$$\langle R^{2n}(\phi,\theta)\rangle = \frac{2}{\pi}\int_0^{\frac{\pi}{2}}\int_0^{\frac{\pi}{2}} R^{2n}(\phi,\theta)\sin\theta\, d\theta d\phi$$

$$= \frac{2}{\pi}\int_0^{\frac{\pi}{2}}\int_0^{\frac{\pi}{2}} ((a\sin\theta\cos\phi)^2 + (b\sin\theta\sin\phi)^2 + (c\cos\theta)^2)^n \sin\phi\, d\theta d\phi$$

$$= \frac{2a^{2n}}{\pi}\int_0^{\frac{\pi}{2}}\int_0^{\frac{\pi}{2}} ([(\cos\phi)^2 + \delta^2(\sin\phi)^2](1-(\cos\theta)^2) + \varepsilon^2(\cos\theta)^2)^n \sin\theta\, d\theta d\phi$$

with the ratios $\delta = b/a$ and $\varepsilon = c/a$. With the same substation $\cos\theta = x$ as above we obtain

$$\langle R^{2n}(\phi,\theta)\rangle = \frac{2a^{2n}}{\pi}\int_0^{\frac{\pi}{2}}\int_0^{1} (p + (\varepsilon^2 - p)x^2)^n\, dxd\phi$$

with $p = (\cos\phi)^2 + \delta^2(\sin\phi)^2$. This can be expressed in the form of a binomial as

$$\langle R^{2n}(\phi,\theta)\rangle = \frac{2a^{2n}}{\pi}\sum_{m=0}^{n}\binom{n}{m}\int_0^{\frac{\pi}{2}}\int_0^{1} p^m(\varepsilon^2 - p)^{n-m}x^{2(n-m)}\, dxd\phi$$

The integral over dx can be performed analyticaly to obtain

$$\langle R^{2n}(\phi,\theta)\rangle = \frac{2a^{2n}}{\pi}\sum_{m=0}^{n}\binom{n}{m}\frac{1}{2(n-m)+1}\int_0^{\frac{\pi}{2}}((\cos\phi)^2+\delta^2(\sin\phi)^2)^m(\varepsilon^2-(\cos\phi)^2-\delta^2(\sin\phi)^2)^{n-m}d\phi$$

$$= \frac{2a^{2n}}{\pi}\sum_{m=0}^{n}\binom{n}{m}\frac{1}{2(n-m)+1}\int_0^{\frac{\pi}{2}}(\delta^2+(1-\delta^2)(\cos\phi)^2)^m(\varepsilon^2-\delta^2+(\delta^2-1)(\cos\phi)^2)^{n-m}d\phi$$

The integrand can be recast into binomials to yield

$$\langle R^{2n}(\phi,\theta)\rangle = \frac{2a^{2n}}{\pi}\sum_{m=0}^{n}\binom{n}{m}\frac{1}{2(n-m)+1}\int_0^{\frac{\pi}{2}}\sum_{k=0}^{m}\binom{m}{k}\delta^{2(m-k)}(1-\delta^2)^k(\cos\phi)^{2k}\sum_{l=0}^{n-m}\binom{n-m}{l}(\varepsilon^2-\delta^2)^{n-m-l}(\delta^2-1)^l(\cos\phi)^{2l}\,d\phi$$

The integrals can be performed analytically as

$$\int_0^{\pi/2}(\cos\phi)^n d\phi = \frac{\sqrt{\pi}\Gamma\left(\frac{n+1}{2}\right)}{2\Gamma\left(\frac{n}{2}+1\right)}$$

$$\int_0^{\pi/2}(\cos\phi)^{2k+2l}d\phi = \frac{\sqrt{\pi}\Gamma\left(\frac{2k+2l+1}{2}\right)}{2\Gamma\left(\frac{2k+2l}{2}+1\right)}$$

and can be inserted into the above expression

$$\langle R^{2n}(\phi,\theta)\rangle = \frac{2a^{2n}}{\pi}\sum_{m=0}^{n}\binom{n}{m}\frac{1}{2(n-m)+1}\sum_{k=0}^{m}\binom{m}{k}\delta^{2(m-k)}(1-\delta^2)^k\sum_{l=0}^{n-m}\binom{n-m}{l}(\varepsilon^2-\delta^2)^{n-m-l}(\delta^2-1)^l\frac{\sqrt{\pi}\Gamma\left(\frac{2k+2l+1}{2}\right)}{2\Gamma\left(\frac{2k+2l}{2}+1\right)}$$

$$= \frac{a^{2n}n!}{\sqrt{\pi}}\sum_{m=0}^{n}\frac{1}{2(n-m)+1}\sum_{k=0}^{m}\frac{\delta^{2(m-k)}(1-\delta^2)^k}{k!\,(m-k)!}\sum_{l=0}^{n-m}\frac{(\varepsilon^2-\delta^2)^{n-m-l}(\delta^2-1))^l}{l!\,(n-m-l)!}\frac{\Gamma\left(k+l+\frac{1}{2}\right)}{(\mathrm{k}+\mathrm{l})!}$$

$$= a^{2n}e_n^{(\theta,\phi)}$$

with

$$e_n^{(\theta,\phi)} = \frac{n!}{\sqrt{\pi}}\sum_{m=0}^{n}\frac{1}{2(n-m)+1}\sum_{k=0}^{m}\frac{\delta^{2(m-k)}(1-\delta^2)^k}{k!\,(m-k)!}\sum_{l=0}^{n-m}\frac{(\varepsilon^2-\delta^2)^{n-m-l}(\delta^2-1))^l}{l!\,(n-m-l)!}\frac{\Gamma\left(k+l+\frac{1}{2}\right)}{(\mathrm{k}+\mathrm{l})!}$$

The formfactor can then be written as **(S.4.3.1.3)**

$$P(q) = \sum_{n=0}^{\infty}p_n^{(3)}e_n^{(\theta,\phi)}\frac{(z+s+1)_{2n}}{\left(\frac{5}{2}\right)_n n!}\left(-\frac{q^2a^2}{4(z+1)^2}\right)^n = \sum_{n=0}^{\infty}c_n q^{2n}$$

with

$$c_n = p_n^{(3)}e_n^{(\theta,\phi)}\frac{(z+s+1)_{2n}}{\left(\frac{5}{2}\right)_n n!}\left(-\frac{a^2}{4(z+1)^2}\right)^n$$

Triaxial Ellipsoid, polydisperse, P(q), isotropic

Simplification:

$$c_n = \frac{6 \cdot 4^n}{(n+3)(n+2)} \frac{(z+s+1)_{2n}}{\left(\frac{5}{2}\right)_n n!} \left(-\frac{a^2}{4(z+1)^2}\right)^n \frac{n!}{\sqrt{\pi}} \sum_{m=0}^{n} \frac{1}{2(n-m)+1} \sum_{k=0}^{m} \frac{\delta^{2(m-k)}(1-\delta^2)^k}{k!\,(m-k)!} \sum_{l=0}^{n-m} \frac{(\varepsilon^2-\delta^2)^{n-m-l}(\delta^2-1))^l}{l!\,(n-m-l)!} \frac{\Gamma\left(k+l+\frac{1}{2}\right)}{(k+l)!}$$

$$= \frac{6(z+s+1)_{2n}}{(n+3)(n+2)\sqrt{\pi}\left(\frac{5}{2}\right)_n} \left(-\frac{a^2}{(z+1)^2}\right)^n \sum_{m=0}^{n} \frac{1}{2(n-m)+1} \sum_{k=0}^{m} \frac{\delta^{2(m-k)}(1-\delta^2)^k}{k!\,(m-k)!} \sum_{l=0}^{n-m} \frac{(\varepsilon^2-\delta^2)^{n-m-l}(\delta^2-1))^l}{l!\,(n-m-l)!} \frac{\Gamma\left(k+l+\frac{1}{2}\right)}{(k+l)!}$$

Regime II. The asymptotic expansions in terms of trigonometric functions are **(S.4.3.1.4)**

$$\langle P(q)\rangle = \frac{18}{\pi} \int_0^{\pi/2} \int_0^{\pi/2} \left[\langle \frac{\left(\sin\left(qR(\phi,\theta)\right)\right)^2}{(qR(\phi,\theta))^6} \rangle - 2 \langle \frac{\sin\left(qR(\phi,\theta)\right) \cos\left(qR(\phi,\theta)\right)}{(qR(\phi,\theta))^5} \rangle + \langle \frac{\left(\cos\left(qR(\phi,\theta)\right)\right)^2}{(qR(\phi,\theta))^4} \rangle \right] \sin\theta \, d\theta d\phi$$

These are two numerical integrations, which however can be performed fast as the oscillations are damped at high q. In addition, there is direct overlap between the series expansion in Regime I and the Porod asymptote in Regime III.

Regime III. The asymptotic expansion linear in q is could be obtained by a double numerical integration of the leading cosine term. It is simpler to use the surface-to-volume ratio with **(S.4.3.1.5)**

$$\lim_{q\to\infty} P(q) = \frac{2\pi}{q^4} \langle \frac{A}{V^2} \rangle = \frac{\Gamma(z+s-3)(z+1)^4}{\Gamma(z+s+1)} \frac{2\pi}{q^4} \frac{A}{V^2}$$

where the volume is given by $V = \frac{4\pi}{3} abc$. The area A is computed numerically from the equation of the ellipsoid in terms of the semiaxes

$$\left(\frac{x}{a}\right)^2 + \left(\frac{y}{b}\right)^2 + \left(\frac{z}{c}\right)^2 = 1$$

Then we can express $z(x,y)$ as

$$z = c\sqrt{1 - \left(\frac{x}{a}\right)^2 - \left(\frac{y}{b}\right)^2}$$

The partial derivatives are

$$z_x = \frac{\partial z}{\partial x} = -\frac{cx}{a\sqrt{1 - \left(\frac{x}{a}\right)^2 - \left(\frac{y}{b}\right)^2}}$$

$$z_y = \frac{\partial z}{\partial y} = -\frac{cy}{b\sqrt{1 - \left(\frac{x}{a}\right)^2 - \left(\frac{y}{b}\right)^2}}$$

The area element for the surface integration is

$$\Delta S = \sqrt{1 + z_x^2 + z_y^2}$$

Such that we can calculate the surface area as

$$A = 8 \int_0^a \int_0^{b\sqrt{1-\left(\frac{x}{a}\right)^2}} \Delta S dy dx$$

A good approximation is provided by

$$A = 4\pi \left(\frac{(ab)^p + (bc)^p + (ac)^p}{3} \right)^{1/p}$$

With $p = 1.6075 \approx 8/5$ (after Knud Thomson), with error < 1.06%.

Mathematica code implementation:

```
a=1;
b=2;
c=3;
nmax=75;
eee=Table[(2/Pi)*N[Sum[((n!)/(2*(n-m)+1))*Sum[((((a*a-b*b)^k))*((b*b)^(m-k))/((k!)*((m-k)!)))*Sum[((b*b-a*a)^l)*((c*c-
b*b)^(n-m-l))*Sqrt[Pi]*Gamma[k+l+1/2]/((l!)*((n-m-l)!)*2*((k+l)!)),{l,0,n-m}],{k, 0, m}],{m, 0, n}]], {n, 0, nmax}];

sigma=0.10;
z=(1-sigma*sigma)/(sigma*sigma);
q=10^lq;
qs=Sqrt[qx*qx+qy*qy];
(* zzz=c*Sqrt[1-(x*x/(a*a))-(y*y/(b*b))];
dzdx=-((c x)/(a^2 Sqrt[1-x^2/a^2-y^2/b^2]));
dzdy=-((c y)/(b^2 Sqrt[1-x^2/a^2-y^2/b^2]));
dS=Sqrt[1+dzdx*dzdx+dzdy*dzdy];
areax=Integrate[dS, {y, 0,b*Sqrt[1-(x*x/(a*a))]}]; *)
pc=1.6075;
area1=4*Pi*((((a*b)^(pc))+((a*c)^(pc))+((b*c)^(pc)))/3)^(1/pc);
vol=4*Pi*a*b*c/3;
R=Sqrt[((a*Sin[theta]*Cos[phi])^2)+((b*Sin[theta]*Sin[phi])^2)+((c*Cos[theta])^2)];
qr=(q*R)/(z+1);
qrs=(qs*R)/(z+1);
fxp=(Gamma[z-3]/(2*Gamma[z+1]))*(qr^(-4))*(1+Cos[(z-3)*ArcTan[2*qr]]/((1+4*qr*qr)^((z-3)/2)));
fyp=(Gamma[z-4]/(2*Gamma[z+1]))*(qr^(-5))*Sin[(z-4)*ArcTan[2*qr]]/((1+4*qr*qr)^((z-4)/2));
fzp=(Gamma[z-5]/(2*Gamma[z+1]))*(qr^(-6))*(1-Cos[(z-5)*ArcTan[2*qr]]/((1+4*qr*qr)^((z-5)/2)));
Pqq=9*NIntegrate[(fxp-2*fyp+fzp)*Sin[theta], {phi, 0, Pi/2}, {theta, 0, Pi/2}]/(Pi/2);
apor=Gamma[z-3]*((z+1)^4)/Gamma[z+1];
(* Pqpor=apor*2*Pi*area/((q^4)*vol*vol); *)
Pqpor1=apor*2*Pi*area1/((q^4)*vol*vol);
Pq1=Sum[6*(4^n)*Pochhammer[z+1,2*n]*((-
q*q/(4*(z+1)*(z+1)))^n)*eee[[n+1]]/((n+3)*(n+2)*Pochhammer[5/2,n]*(n!)),{n, 0, nmax}];
Pq1s=Sum[6*(4^n)*Pochhammer[z+1,2*n]*((-
qs*qs/(4*(z+1)*(z+1)))^n)*eee[[n+1]]/((n+3)*(n+2)*Pochhammer[5/2,n]*(n!)),{n, 0, nmax}];
lim=1.5;
pl2=Plot[Log[10,Pq1], {lq , -1,lim}, PlotRange->{-6,1}, GridLines->{Automatic}, LabelStyle->Directive[Black,16],AxesLabel-
>{"log q","lg P(q)"}, AxesOrigin->{-1,-6},TicksStyle->Directive[Black,12],PlotStyle->{Black,Dashed}] ;
pl1=Plot[Log[10,Pqq], {lq , -1,lim}, PlotRange->{-6,1}, GridLines->{Automatic}, LabelStyle->Directive[Black,16],AxesLabel-
>{"log q","lg P(q)"}, AxesOrigin->{-1,-6},TicksStyle->Directive[Black,12],PlotStyle->{Blue}];
pl3=Plot[Log[10,Pqpor1], {lq , -1,lim}, PlotRange->{-6,1}, GridLines->{Automatic}, LabelStyle->Directive[Black,16],AxesLabel-
>{"log q","lg P(q)"}, AxesOrigin->{-1,-6},TicksStyle->Directive[Black,10],PlotStyle->{Red}] ;
```

```
Show[pl2,pl1,pl3]
lims=6;
pl4=DensityPlot[Log[10,Pq1s],{qx, -lims, lims}, {qy, -lims, lims}, PlotRange->{-8,0}, PlotPoints->50, PlotLegends->Automatic, LabelStyle->Directive[Black,12],AxesLabel->Automatic]
```

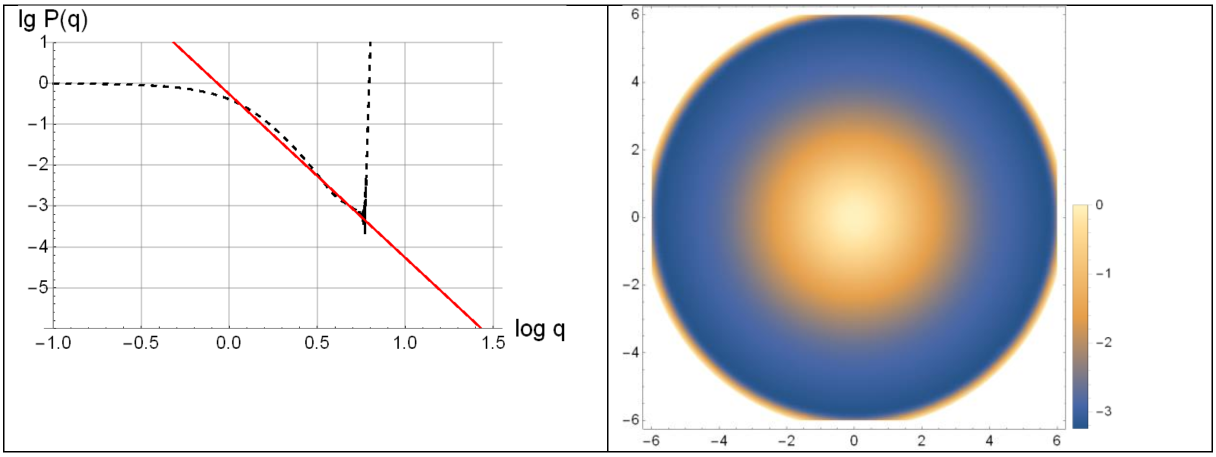

Fig. S9: Formfactor of a polydisperse isotropic triaxial ellipsoids.

4.3.2. Scattering Amplitude F(q)

Integral representation. <u>(S.4.3.2.1)</u>

$$\langle F(q)\rangle^2 = \frac{18}{\pi}\int_0^{\pi/2}\int_0^{\pi/2}\left(\int_0^{\infty}\frac{\sin(qR(\phi,\theta)) - qR(\phi,\theta)\cos(qR(\phi,\theta))}{(qR(\phi,\theta))^3}R^s((\phi,\theta))h(R(\phi,\theta))dr\right)^2 \sin\theta\, d\theta\, d\phi$$

with $s = 3$.

Regime I: We can use the angular averaged radii as above to obtain **<u>(S.4.3.2.2)</u>**

$$\langle F(q)\rangle^2 = \sum_{n=0}^{\infty} f_n^{(3)} e_n^{(\theta,\phi)}\left(-\frac{q^2a^2}{4(z+1)^2}\right)^n = \sum_{n=0}^{\infty} c_n q^2$$

Triaxial Ellipsoid, polydisperse, isotropic, F(q)

Regime II: (S.4.3.2.3)

$$\langle F(q)\rangle^2 = \frac{18}{\pi}\int_0^{\pi/2}\int_0^{\pi/2}\left(\langle\frac{\sin(qR(\phi,\theta))}{q^3R^3(\phi,\theta)}\rangle - \langle\frac{\cos(qR(\phi,\theta))}{q^2R^2(\phi,\theta)}\rangle\right)^2 \sin\theta\, d\theta\, d\phi$$

with

$$\langle\frac{\cos\big(qR(\phi,\theta)\big)}{(qR(\phi,\theta))^2}\rangle = \frac{\Gamma[z+s-1]}{\Gamma[z+s+1]}\left(\frac{qR(\phi,\theta)}{z+1}\right)^{-2} \frac{\cos\left[(z+s-1)\arctan\left(\frac{qR(\phi,\theta)}{z+1}\right)\right]}{\left(1+\left(\frac{qR(\phi,\theta)}{z+1}\right)^2\right)^{\frac{z+s-1}{2}}}$$

$$\langle\frac{\sin\big(qR(\phi,\theta)\big)}{(qR(\phi,\theta))^3}\rangle = \frac{\Gamma[z+s-2]}{\Gamma[z+s+1]}\left(\frac{qR(\phi,\theta)}{z+1}\right)^{-3} \frac{\sin\left[(z+s-2)\arctan\left(\frac{qR(\phi,\theta)}{z+1}\right)\right]}{\left(1+\left(\frac{qR(\phi,\theta)}{z+1}\right)^2\right)^{\frac{z+s-2}{2}}}$$

Mathematica code implementation:

```
a=1;
b=2;
c=3;
nmax=75;
eee=Table[(2/Pi)*N[Sum[((n!)/(2*(n-m)+1))*Sum[((((a*a-b*b)^k))*((b*b)^(m-k))/((k!)*((m-k)!)))*Sum[((b*b-a*a)^l)*((c*c-b*b)^(n-m-l))*Sqrt[Pi]*Gamma[k+l+1/2]/((l!)*((n-m-l)!)*2*((k+l)!)),{l,0,n-m}],{k, 0, m}],{m, 0, n}]], {n, 0, nmax}];

sigma=0.10;
z=(1-sigma*sigma)/(sigma*sigma);
q=10^lq;
qs=Sqrt[qx*qx+qy*qy];
R=Sqrt[((a*Sin[theta]*Cos[phi])^2)+((b*Sin[theta]*Sin[phi])^2)+((c*Cos[theta])^2)];
qr=(q*R)/(z+1);
qrs=(qs*R)/(z+1);
fxf=(Gamma[z-2]/(Gamma[z+1]))*(qr^(-3))*Sin[(z-2)*ArcTan[qr]]/((1+qr*qr)^((z-2)/2));
fyf=(Gamma[z-1]/(Gamma[z+1]))*(qr^(-2))*Cos[(z-1)*ArcTan[qr]]/((1+qr*qr)^((z-1)/2));
(* Fqq=9*NIntegrate[((fxf-fyf)^2)*Sin[theta], {phi, 0, Pi/2}, {theta, 0, Pi/2}]/(Pi/2);*)
fff=Table[Sum[Pochhammer[z+1,2*(n-m)]*Pochhammer[z+1,2*m]/(Pochhammer[5/2,n-m]*Pochhammer[5/2,m]*((n-m)!)*(m!)), {m, 0, n}],{n, 0, nmax}];
Fq1=Sum[(((-q*q/(4*(z+1)*(z+1)))^n)*eee[[n+1]]*fff[[n+1]]), {n, 0, nmax}];
Fq1s=Sum[(((-qs*qs/(4*(z+1)*(z+1)))^n)*eee[[n+1]]*fff[[n+1]]), {n, 0, nmax}];
lim=1.5;
pl2=Plot[Log[10,Fq1], {lq , -1,lim}, PlotRange->{-6,1}, GridLines->{Automatic}, LabelStyle->Directive[Black,16],AxesLabel->{"log q","lg F^2(q)"}, AxesOrigin->{-1,-6},TicksStyle->Directive[Black,12],PlotStyle->{Black,Dashed}] ;
(* pl1=Plot[Log[10,Fqq], {lq , -1,lim}, PlotRange�{-6,1}, GridLines->{Automatic}, LabelStyle->Directive[Black,16],AxesLabel->{"log q","lg F^2(q)"}, AxesOrigin->{-1,-6},TicksStyle->Directive[Black,12],PlotStyle->{Blue}]; *)
Show[pl2]
lims=7;
pl4=DensityPlot[Log[10,Fq1s],{qx, -lims, lims}, {qy, -lims, lims}, PlotRange->{-8,0}, PlotPoints->50, PlotLegends->Automatic, LabelStyle->Directive[Black,12],AxesLabel->Automatic]
```

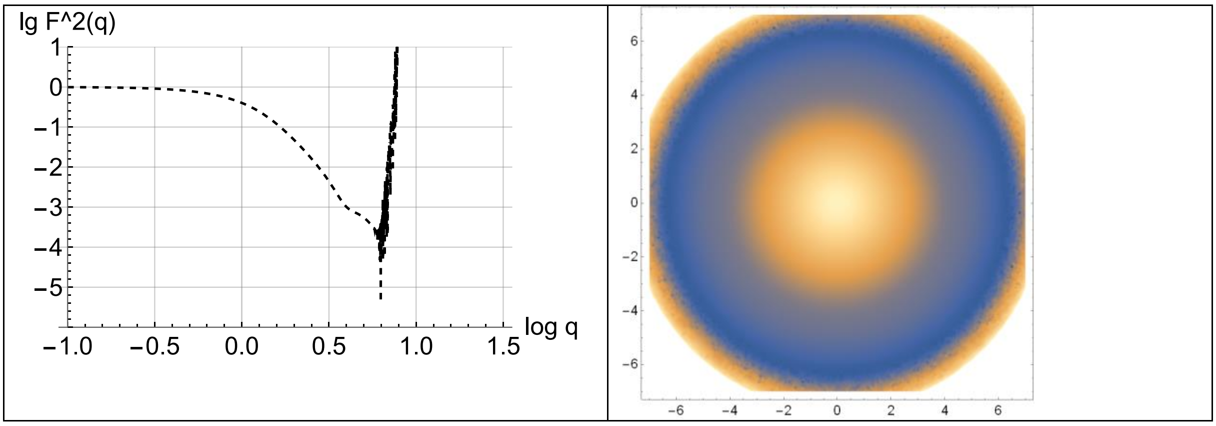

Fig. S10: Scattering amplitude of a polydisperse isotropic triaxial ellipsoids.

4.4 Cube

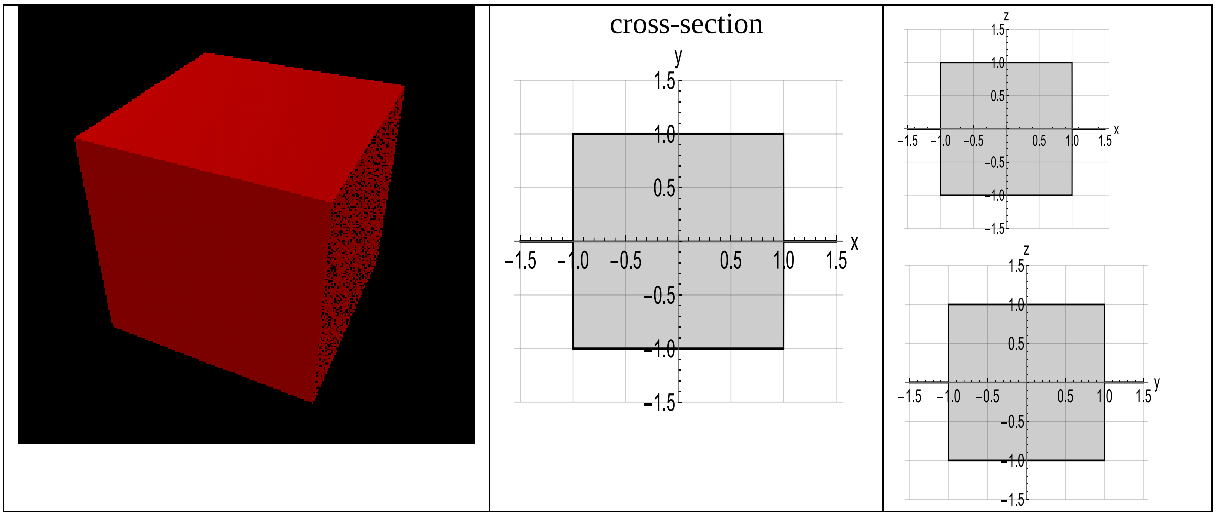

Fig. S11: Cube with cross-sections in the (*x*,*y*)-, (*x*,*z*)-, and (*y*,*z*)-planes.

4.4.1. Formfactor P(q)

Integral representation. (S.4.4.1.1)

$$\langle P_{cube}(q)\rangle_a = \frac{2}{\pi}\int_0^{\frac{\pi}{2}}\int_0^{\frac{\pi}{2}} \langle P^{(1)}(q_x a)\rangle_a \, \langle P^{(1)}(q_y a)\rangle_a \langle P^{(1)}(q_z a)\rangle_a \sin\theta \, d\theta d\phi$$

with

$$q_x = q\sin\theta\cos\phi$$

$$q_y = q\sin\theta\sin\phi$$

$$q_z = q\cos\theta$$

and thus

$$\langle P_{cube}(q)\rangle_a = \frac{2}{\pi}\int_0^{\frac{\pi}{2}}\int_0^{\frac{\pi}{2}}\left[\int_0^{\infty}\left(\frac{\sin(qa\sin\theta\cos\phi)}{qa\sin\theta\cos\phi}\right)^2 a^s h(a)da\int_0^{\infty}\left(\frac{\sin(qa\sin\theta\sin\phi)}{qa\sin\theta\sin\phi}\right)^2 a^s h(a)da\int_0^{\infty}\left(\frac{\sin(qa\cos\theta)}{qa\cos\theta}\right)^2 a^s h(a)da\right]\sin\theta\, d\theta d\phi$$

with $s = 2$.

Regime I. With $\langle P^{(1)}(q)\rangle_a$ from Table I we have the angular dependent series

$$\langle P_{cube}(q,\theta,\phi)\rangle_a = \left(\sum_{n=0}^{\infty}\frac{4^n}{(\mathrm{n}+1)}\frac{(z+s+1)_{2n}}{\left(\frac{3}{2}\right)_n n!}\left(-\frac{(qa\sin\theta\cos\phi)^2}{4(z+1)^2}\right)^n\right)\left(\sum_{m=0}^{\infty}\frac{4^m}{(\mathrm{n}+1)}\frac{(z+s+1)_{2m}}{\left(\frac{3}{2}\right)_m m!}\left(-\frac{(qa\sin\theta\sin\phi)^2}{4(z+1)^2}\right)^m\right)\left(\sum_{l=0}^{\infty}\frac{4^l}{(\mathrm{l}+1)}\frac{(z+s+1)_{2l}}{\left(\frac{3}{2}\right)_l l!}\left(-\frac{(qa\cos\theta)^2}{4(z+1)^2}\right)^l\right)$$

The series is integrated over the angles

$$P(q) = \frac{2}{\pi}\sum_{n=0}^{\infty}\left(-\frac{q^2a^2}{4(z+1)^2}\right)^n\frac{4^n}{(\mathrm{n}+1)}\frac{(z+s+1)_{2n}}{\left(\frac{3}{2}\right)_n n!}\sum_{m=0}^{\infty}\left(-\frac{q^2a^2}{4(z+1)^2}\right)^m\frac{4^m}{(\mathrm{m}+1)}\frac{(z+s+1)_{2m}}{\left(\frac{3}{2}\right)_m m!}\sum_{l=0}^{\infty}\left(-\frac{q^2a^2}{4(z+1)^2}\right)^l\frac{4^l}{(\mathrm{l}+1)}\frac{(z+s+1)_{2l}}{\left(\frac{3}{2}\right)_l l!}$$
$$\int_0^{\frac{\pi}{2}}\int_0^{\frac{\pi}{2}}(\cos\phi)^{2n}(\sin\phi)^{2m}(\sin\theta)^{2n+2m}(\cos\theta)^{2l}\sin\theta\, d\theta d\phi$$

The double integral can be solved analytically to obtain

$$\int_0^{\frac{\pi}{2}}\int_0^{\frac{\pi}{2}}(\cos\phi)^{2n}(\sin\phi)^{2m}(\sin\theta)^{2n+2m}(\cos\theta)^{2l}\sin\theta\, d\theta d\phi = \frac{\Gamma\left(l+\frac{1}{2}\right)\Gamma\left(m+\frac{1}{2}\right)\Gamma\left(n+\frac{1}{2}\right)}{4\Gamma\left(n+m+l+\frac{3}{2}\right)}$$

This can be inserted into the series expansion

$$P(q) = \frac{2}{4\pi}\sum_{n=0}^{\infty}\left(-\frac{q^2a^2}{4(z+1)^2}\right)^n\frac{4^n\Gamma\left(n+\frac{1}{2}\right)}{(\mathrm{n}+1)}\frac{(z+s+1)_{2n}}{\left(\frac{3}{2}\right)_n n!}\sum_{m=0}^{\infty}\left(-\frac{q^2a^2}{4(z+1)^2}\right)^m\frac{4^m\Gamma\left(m+\frac{1}{2}\right)}{(\mathrm{m}+1)}\frac{(z+s+1)_{2m}}{\left(\frac{3}{2}\right)_m m!}\sum_{l=0}^{\infty}\left(-\frac{q^2a^2}{4(z+1)^2}\right)^l\frac{4^l\Gamma\left(l+\frac{1}{2}\right)}{(\mathrm{l}+1)}\frac{(z+s+1)_{2l}}{\left(\frac{3}{2}\right)_l l!\,\Gamma\left(n+m+l+\frac{3}{2}\right)}$$

The Pochhammer factorials can be expressed in terms of Gamma functions as

$$P(q) = \frac{2}{4\pi}\sum_{n=0}^{\infty}\left(-\frac{q^2a^2}{4(z+1)^2}\right)^n\frac{4^n\Gamma\left(n+\frac{1}{2}\right)}{(\mathrm{n}+1)}\frac{(z+s+1)_{2n}\Gamma\left(\frac{3}{2}\right)}{\Gamma\left(n+\frac{3}{2}\right)n!}\sum_{m=0}^{\infty}\left(-\frac{q^2a^2}{4(z+1)^2}\right)^m\frac{4^m\Gamma\left(m+\frac{1}{2}\right)}{(\mathrm{m}+1)}\frac{(z+s+1)_{2m}\Gamma\left(\frac{3}{2}\right)}{\Gamma\left(m+\frac{3}{2}\right)m!}\sum_{l=0}^{\infty}\left(-\frac{q^2a^2}{4(z+1)^2}\right)^l\frac{4^l\Gamma\left(l+\frac{1}{2}\right)}{(\mathrm{l}+1)}\frac{\Gamma\left(\frac{3}{2}\right)(z+s+1)_{2l}}{\Gamma\left(l+\frac{3}{2}\right)l!\,\Gamma\left(n+m+l+\frac{3}{2}\right)}$$

$$= \frac{\pi^{1/2}}{16\pi}\sum_{n=0}^{\infty}\left(-\frac{q^2a^2}{4(z+1)^2}\right)^n\frac{4^n}{(\mathrm{n}+1)}\frac{(z+s+1)_{2n}}{\left(n+\frac{1}{2}\right)n!}\sum_{m=0}^{\infty}\left(-\frac{q^2a^2}{4(z+1)^2}\right)^m\frac{4^m}{(\mathrm{m}+1)}\frac{(z+s+1)_{2m}}{\left(m+\frac{1}{2}\right)m!}\sum_{l=0}^{\infty}\left(-\frac{q^2a^2}{4(z+1)^2}\right)^l\frac{4^l}{(\mathrm{l}+1)}\frac{(z+s+1)_{2l}}{\left(l+\frac{1}{2}\right)l!\,\Gamma\left(n+m+l+\frac{3}{2}\right)}$$

The three infinite series can finally be expressed via the Cauchy summation as **<u>(S.4.4.1.2)</u>**

$$P(q) = \frac{\pi^{1/2}}{16\pi}\sum_{n=0}^{\infty}\frac{4^n}{\Gamma\left(n+\frac{3}{2}\right)}\left(-\frac{q^2a^2}{4(z+1)^2}\right)^n\sum_{m=0}^{n}\frac{(z+s+1)_{2(n-m)}}{\left(n-m+\frac{1}{2}\right)(n-m+1)!}\sum_{l=0}^{m}\frac{(z+s+1)_{2(m-l)}}{\left(m-l+\frac{1}{2}\right)(m-l+1)!}\frac{(z+s+1)_{2l}}{\left(l+\frac{1}{2}\right)(l+1)!}$$

$$= \frac{\pi^{1/2}}{16\pi}\sum_{n=0}^{\infty}\frac{4^n}{\Gamma\left(n+\frac{3}{2}\right)}\left(-\frac{q^2a^2}{4(z+1)^2}\right)^n c_n$$

with

$$c_n = \sum_{m=0}^{n} \frac{(z+s+1)_{2(n-m)}}{\left(n-m+\frac{1}{2}\right)(n-m+1)!} \sum_{l=0}^{m} \frac{(z+s+1)_{2(m-l)}}{\left(m-l+\frac{1}{2}\right)(m-l+1)!} \frac{(z+s+1)_{2l}}{\left(l+\frac{1}{2}\right)(l+1)!}$$

Cube, polydisperse, isotropic, P(q)

Regime II. <u>(S.4.4.1.3)</u>

$$\langle P_{cube}(q)\rangle_a = \frac{2}{\pi} \int_0^{\frac{\pi}{2}} \int_0^{\frac{\pi}{2}} \langle\left(\frac{\sin(q_x a)}{q_x a}\right)^2\rangle_a \langle\left(\frac{\sin(q_y a)}{q_y a}\right)^2\rangle_a \langle\left(\frac{\sin(q_z a)}{q_z a}\right)^2\rangle_a \, d\theta d\phi$$

$$\langle\left(\frac{\sin(q_i a)}{q_i a}\right)^2\rangle = \frac{\Gamma[z+s-n+1]}{\Gamma[z+s+1]} \frac{1}{2} u^{-n} \left(1 - \frac{\cos[(z+s-n+1)\arctan(2au)]}{(1+4a^2u^2)^{\frac{z+s-n+1}{2}}}\right)$$

$$u = \frac{q_i a}{z+1}$$

Regime III. The Porod asymptote is calculated from the surface area and the volume

$$A = 24a^2$$

$$V = 8a^3$$

Since the edge length is 2a, with the average

$$\langle\frac{1}{x^4}\rangle = \frac{\Gamma(z+s-3)}{\Gamma(z+s+1)} \left(\frac{z+1}{x}\right)^4$$

With $s = 6$, such that we have for the Porod asymptote **<u>(S.4.4.1.4)</u>**

$$P(q) = \frac{2\pi}{q^4} \langle\frac{A}{V^2}\rangle = \frac{\Gamma(z+s-3)(z+1)^4}{\Gamma(z+s+1)} \frac{2\pi}{q^4} \frac{A}{V^2}$$

The following graph shows the formfactors from the series expansions compared to the numerical integrations and the Porod-asymptote. We note the overlap of the series expansion and the Porod-asymptote, such that numerical integration is only necessary at special conditions. As the coefficients can be precalculated, the series expansion is rapidly computed.

Mathematica code implementation:

```
a=1;
b=1;
c=1;
sigma=0.1;
z=(1-sigma*sigma)/(sigma*sigma);
area=8*(a*b+a*c+b*c);
vol=8*a*b*c;
nmax=80;
q=10^lq;
```

```
qs=Sqrt[qx*qx+qy*qy];
a1p=Gamma[z-1]/(2*Gamma[z+1]);
qa=q*a*Cos[phi]*Sin[theta]/(z+1);
qb=q*b*Sin[phi]*Sin[theta]/(z+1);
qc=q*c*Cos[theta]/(z+1);
fxp=a1p*(1/(qa*qa))*(1-Cos[(z-1)*ArcTan[2*qa]]/((1+4*qa*qa)^((z-1)/2)));
fyp=a1p*(1/(qb*qb))*(1-Cos[(z-1)*ArcTan[2*qb]]/((1+4*qb*qb)^((z-1)/2)));
fzp=a1p*(1/(qc*qc))*(1-Cos[(z-1)*ArcTan[2*qc]]/((1+4*qc*qc)^((z-1)/2)));
(* Pqq=NIntegrate[fxp*fyp*fzp*Sin[theta], {phi, 0, Pi/2}, {theta, 0, Pi/2}]/(Pi/2);  *)
apor=Gamma[z-3]*((z+1)^4)/Gamma[z+1];
Pqpor=apor*2*Pi*area/((q^4)*vol*vol);
fff=Table[Sum[Pochhammer[z+1,2*(n-m)]*Pochhammer[z+1,2*m]/(Pochhammer[3/2,n-m]*Pochhammer[3/2,m]*((n-
m)!)*(m!)), {m, 0, n}],{n, 0, nmax}];
Pq1=(Sqrt[Pi]/16)*Sum[((4^n)*((-q*q/(4*(z+1)*(z+1)))^n)/Gamma[n+3/2])*Sum[(Pochhammer[z+1,2*(n-m)]*((a*a)^(n-
m))/((n-m+1/2)*((n-m+1)!)))*Sum[(Pochhammer[z+1,2*(m-l)]*((b*b)^(m-l))/((m-l+1/2)*((m-
l+1)!)))*(Pochhammer[z+1,2*l]*((c*c)^l)/((l+1/2)*((l+1)!))),{l, 0, m}], {m, 0, n}], {n, 0, nmax}];
Pq1s=(Sqrt[Pi]/16)*Sum[((4^n)*((-qs*qs/(4*(z+1)*(z+1)))^n)/Gamma[n+3/2])*Sum[(Pochhammer[z+1,2*(n-m)]*((a*a)^(n-
m))/((n-m+1/2)*((n-m+1)!)))*Sum[(Pochhammer[z+1,2*(m-l)]*((b*b)^(m-l))/((m-l+1/2)*((m-
l+1)!)))*(Pochhammer[z+1,2*l]*((c*c)^l)/((l+1/2)*((l+1)!))),{l, 0, m}], {m, 0, n}], {n, 0, nmax}];
lim=1.5;
pl2=Plot[Log[10,Pq1], {lq , -1,lim}, PlotRange->{-6,1}, GridLines->{Automatic}, LabelStyle->Directive[Black,16],AxesLabel-
>{"log q","lg P(q)"}, AxesOrigin->{-1,-6},TicksStyle->Directive[Black,12],PlotStyle->{Black,Dashed}] ;
(* pl1=Plot[Log[10,Pqq], {lq , -1,lim}, PlotRange�{-6,1}, GridLines->{Automatic}, LabelStyle->Directive[Black,16],AxesLabel-
>{"log q","lg P(q)"}, AxesOrigin->{-1,-6},TicksStyle->Directive[Black,12],PlotStyle->{Blue}]; *)
pl3=Plot[Log[10,Pqpor], {lq , -1,lim}, PlotRange->{-6,1}, GridLines->{Automatic}, LabelStyle->Directive[Black,16],AxesLabel-
>{"log q","lg P(q)"}, AxesOrigin->{-1,-6},TicksStyle->Directive[Black,10],PlotStyle->{Red}] ;
Show[pl2,pl3]
lims=12;
pl4=DensityPlot[Log[10,Pq1s],{qx, -lims, lims}, {qy, -lims, lims}, PlotRange->{-8,0}, PlotPoints->50, PlotLegends->Automatic,
LabelStyle->Directive[Black,12],AxesLabel->Automatic]
```

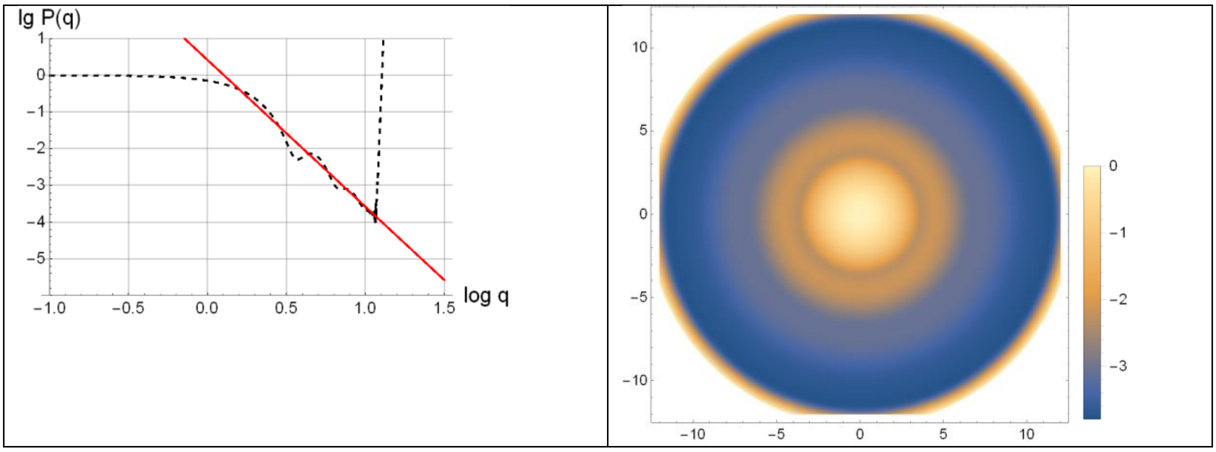

Fig. S13: Formfactor of polydisperse isotropic cubes.

4.4.2. Scattering Amplitude F(q)

Integral representation. **(S.4.4.2.1)**

$$\langle F_{cube}(q)\rangle_a^2 = \frac{2}{\pi}\int_0^{\frac{\pi}{2}}\int_0^{\frac{\pi}{2}} \left(\langle F^{(1)}(q_x a)\rangle_a \langle F^{(1)}(q_y b)\rangle_b \langle F^{(1)}(q_z c)\rangle_c\right)^2 \sin\theta\, d\theta d\phi$$

$$= \frac{2}{\pi}\int_0^{\frac{\pi}{2}}\int_0^{\frac{\pi}{2}} \left(\int_0^{\infty} \frac{\sin(qa\sin\theta\cos\phi)}{qa\sin\theta\cos\phi} a^s h(a) da \int_0^{\infty} \frac{\sin(qb\sin\theta\sin\phi)}{qb\sin\theta\sin\phi} b^s h(b) db \int_0^{\infty} \frac{\sin(qc\cos\theta)}{qc\cos\theta} c^s h(c) dc\right)^2 \sin\theta\, d\theta d\phi$$

with $s = 1$.

Regime I: We can use the same angular averages as for the formfactor to obtain

$$\langle F(q)\rangle^2 = \frac{2}{\pi}\sum_{n=0}^{\infty}\left(-\frac{q^2a^2}{4(z+1)^2}\right)^n f_n^{(1)} \sum_{m=0}^{\infty}\left(-\frac{q^2a^2}{4(z+1)^2}\right)^m f_m^{(1)} \sum_{l=0}^{\infty}\left(-\frac{q^2a^2}{4(z+1)^2}\right)^l f_l^{(1)} \frac{\Gamma\left(l+\frac{1}{2}\right)\Gamma\left(m+\frac{1}{2}\right)\Gamma\left(n+\frac{1}{2}\right)}{4\Gamma\left(n+m+l+\frac{3}{2}\right)}$$

$$= \frac{2}{\pi}\sum_{n=0}^{\infty}\left(-\frac{q^2a^2}{4(z+1)^2}\right)^n \Gamma\left(n+\frac{1}{2}\right) f_n^{(1)} \sum_{m=0}^{\infty}\left(-\frac{q^2a^2}{4(z+1)^2}\right)^m \Gamma\left(m+\frac{1}{2}\right) f_m^{(1)} \sum_{l=0}^{\infty}\left(-\frac{q^2a^2}{4(z+1)^2}\right)^l f_l^{(1)} \frac{\Gamma\left(l+\frac{1}{2}\right)}{4\Gamma\left(n+m+l+\frac{3}{2}\right)}$$

Using Cauchy summation we obtain **(S.4.4.2.2)**

$$\langle F(q)\rangle^2 = \frac{1}{2\pi}\sum_{n=0}^{\infty}\frac{1}{\Gamma\left(n+\frac{3}{2}\right)}\left(-\frac{q^2a^2}{4(z+1)^2}\right)^n \sum_{m=0}^{n}\Gamma\left(n-m+\frac{1}{2}\right) f_{n-m}^{(1)} \sum_{l=0}^{m} f_{m-l}^{(1)} f_l^{(1)} \Gamma\left(m-l+\frac{1}{2}\right)\Gamma\left(l+\frac{1}{2}\right)$$

$$\langle F(q)\rangle^2 = \frac{1}{2\pi}\sum_{n=0}^{\infty}\frac{1}{\Gamma\left(n+\frac{3}{2}\right)}\left(-\frac{q^2a^2}{4(z+1)^2}\right)^n c_n$$

$$c_n = \sum_{m=0}^{n}\Gamma\left(n-m+\frac{1}{2}\right) f_{n-m}^{(1)} \sum_{l=0}^{m} f_{m-l}^{(1)} f_l^{(1)} \Gamma\left(m-l+\frac{1}{2}\right)\Gamma\left(l+\frac{1}{2}\right)$$

$$f_n^{(1)} = \frac{\pi}{4}\sum_{m=0}^{n}\frac{(z_r+s+1)_{2(n-m)}(z_r+s+1)_{2m}}{\Gamma\left[n-m+\frac{3}{2}\right]\Gamma\left[m+\frac{3}{2}\right](n-m)!\,m!}$$

Cube, polydisperse, isotropic, F(q)

Regime II: **(S.4.4.2.3)**

$$\langle F(q)\rangle^2 = \frac{2}{\pi}\int_0^{\pi/2}\int_0^{\pi/2}\left(\langle\frac{\sin(qa\sin\theta\cos\phi)}{qa\sin\theta\cos\phi}\rangle\langle\frac{\sin(qa\sin\theta\sin\phi)}{qa\sin\theta\sin\phi}\rangle\langle\frac{\sin(qa\cos\theta)}{qa\cos\theta}\rangle\right)^2\sin\theta\, d\theta\, d\phi$$

We need

$$\langle\frac{\sin(q_x a)}{q_x a}\rangle = \frac{\Gamma[z+s-1]}{\Gamma[z+s+1]}\left(\frac{q_x a}{z+1}\right)^{-2}\frac{\sin\left[(z+s-1)\arctan\left(\frac{q_x a}{z+1}\right)\right]}{\left(1+\left(\frac{q_x a}{z+1}\right)^2\right)^{\frac{z+s-1}{2}}}$$

Mathematica code implementation:

```
a=1;
b=1;
c=1;
sigma=0.1;
z=(1-sigma*sigma)/(sigma*sigma);
area=8*(a*b+a*c+b*c);
vol=8*a*b*c;
nmax=80;
q=10^lq;
a1f=Gamma[z]/(Gamma[z+1]);
qa=q*a*Cos[phi]*Sin[theta]/(z+1);
qb=q*b*Sin[phi]*Sin[theta]/(z+1);
qc=q*c*Cos[theta]/(z+1);
fxf=a1f*(1/qa)*Sin[z*ArcTan[qa]]/((1+qa*qa)^(z/2));
fyf=a1f*(1/qb)*Sin[z*ArcTan[qb]]/((1+qb*qb)^(z/2));
fzf=a1f*(1/qc)*Sin[z*ArcTan[qc]]/((1+qc*qc)^(z/2));
(* Fqq=NIntegrate[fxf*fxf*fyf*fyf*fzf*fzf*Sin[theta], {phi, 0, Pi/2}, {theta, 0, Pi/2}]/(Pi/2); *)
fff=Table[Sum[Pochhammer[z+1,2*(n-m)]*Pochhammer[z+1,2*m]/(Pochhammer[3/2,n-m]*Pochhammer[3/2,m]*((n-
m)!)*(m!)), {m, 0, n}],{n, 0, nmax}];
(* Fq7=(1/(2*Pi))*Sum[(Gamma[n+1/2]*((-q*q*a*a/(4*(z+1)*(z+1)))^n)*fff[[n+1]])*Sum[(Gamma[m+1/2]*((-
q*q*b*b/(4*(z+1)*(z+1)))^m)*fff[[m+1]])*Sum[(Gamma[l+1/2]*((-
q*q*c*c/(4*(z+1)*(z+1)))^l)*fff[[l+1]])/(Gamma[n+m+l+3/2]),{l, 0, nmax}], {m, 0, nmax}], {n, 0, nmax}];  *)
Fq1=(1/(2*Pi))*Sum[(((-q*q/(4*(z+1)*(z+1)))^n)/Gamma[n+3/2])*Sum[(Gamma[n-m+1/2]*((a*a)^(n-m))*fff[[n-
m+1]])*Sum[(Gamma[m-l+1/2]*Gamma[l+1/2]*((b*b)^(m-l))*((c*c)^l)*fff[[m-l+1]]*fff[[l+1]]),{l, 0, m}], {m, 0,n}], {n, 0,
nmax}];
Fq1s=(1/(2*Pi))*Sum[(((-qs*qs/(4*(z+1)*(z+1)))^n)/Gamma[n+3/2])*Sum[(Gamma[n-m+1/2]*((a*a)^(n-m))*fff[[n-
m+1]])*Sum[(Gamma[m-l+1/2]*Gamma[l+1/2]*((b*b)^(m-l))*((c*c)^l)*fff[[m-l+1]]*fff[[l+1]]),{l, 0, m}], {m, 0,n}], {n, 0,
nmax}];
lim=1.5;
pl2=Plot[Log[10,Fq1], {lq , -1,lim}, PlotRange->{-6,1}, GridLines->{Automatic}, LabelStyle->Directive[Black,16],AxesLabel-
>{"log q","lg F^2(q)"}, AxesOrigin->{-1,-6},TicksStyle->Directive[Black,12],PlotStyle->{Black,Dashed}] ;
(* pl1=Plot[Log[10,Fqq], {lq , -1,lim}, PlotRange[illegible]{-6,1}, GridLines->{Automatic}, LabelStyle->Directive[Black,16],AxesLabel-
>{"log q","lg F^2(q)"}, AxesOrigin->{-1,-6},TicksStyle->Directive[Black,12],PlotStyle->{Blue}]; *)
Show[pl2]
lims=15;
pl4=DensityPlot[Log[10,Fq1s],{qx, -lims, lims}, {qy, -lims, lims}, PlotRange->{-8,0}, PlotPoints->50, PlotLegends->Automatic,
LabelStyle->Directive[Black,12],AxesLabel->Automatic]
```

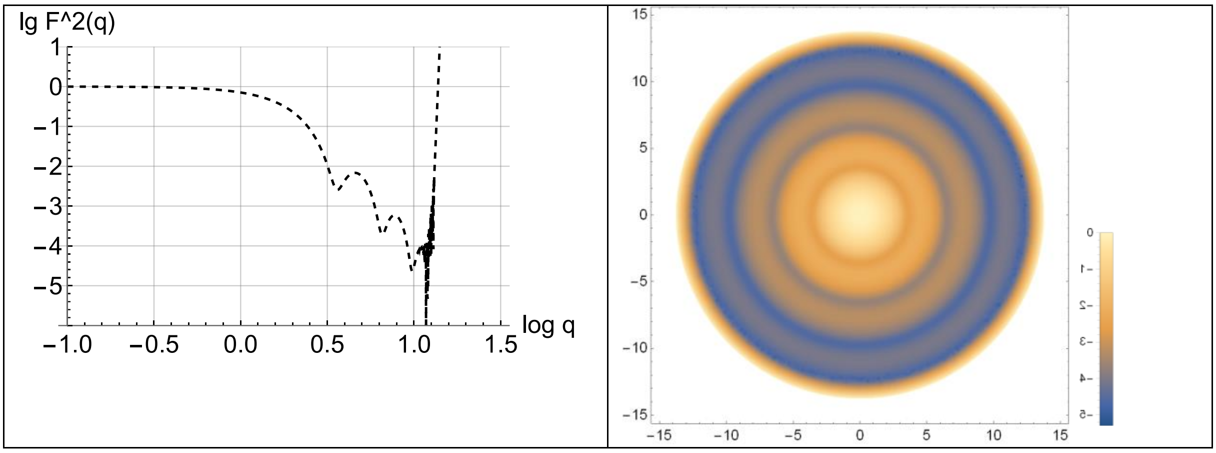

Fig. S14: Scattering amplitude of polydisperse isotropic cubes.

4.5 Parallelepiped

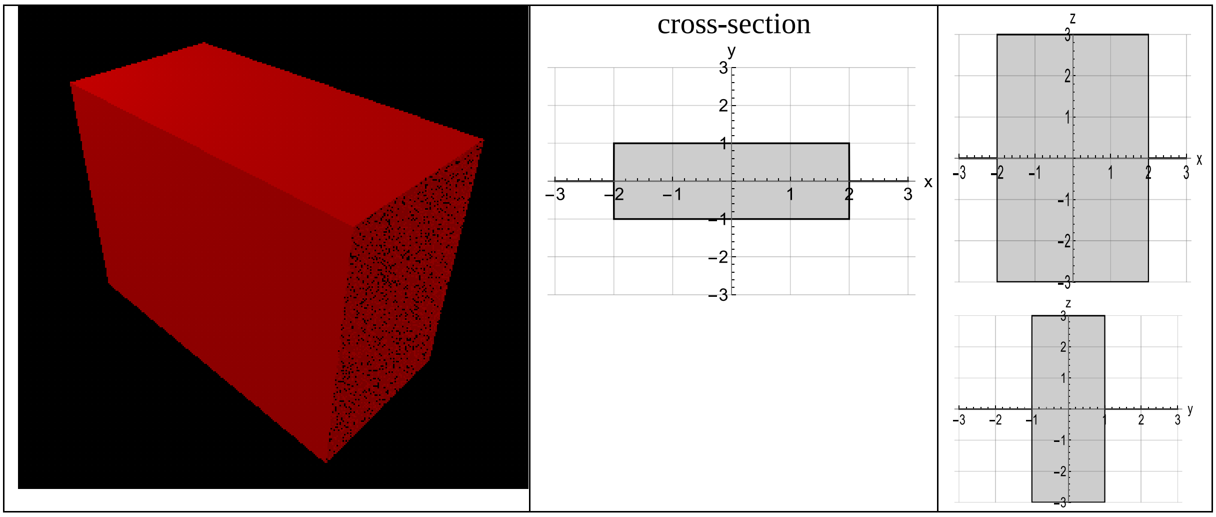

Fig. S15: Parallelepiped with cross-sections in the (*x*,*y*)-, (*x*,*z*)-, and (*y*,*z*)-planes.

4.5.1 Formfactor P(q)

Integral representation. **(S.4.5.1.1)**

$$\langle P_{pa}(q)\rangle_{a,b,c} = \frac{2}{\pi}\int_0^{\frac{\pi}{2}}\int_0^{\frac{\pi}{2}} \langle P^{(1)}(q_x a)\rangle_a \, \langle P^{(1)}(q_y b)\rangle_b \langle P^{(1)}(q_z c)\rangle_c \sin\theta \, d\theta d\phi$$

$$=\frac{2}{\pi}\int_0^{\frac{\pi}{2}}\int_0^{\frac{\pi}{2}}\left[\int_0^{\infty}\left(\frac{\sin(qa\sin\theta\cos\phi)}{qa\sin\theta\cos\phi}\right)^2 a^s h(a)da\int_0^{\infty}\left(\frac{\sin(qb\sin\theta\sin\phi)}{qb\sin\theta\sin\phi}\right)^2 b^s h(b)db\int_0^{\infty}\left(\frac{\sin(qc\cos\theta)}{qc\cos\theta}\right)^2 c^s h(c)dc\right]\sin\theta\, d\theta d\phi$$

Regime I. We assume that the size distributions are mutually independent and can be factorized. Then we have the same integral as for the cube, i.e. **(S.4.5.1.2)**

$$P(q) = \frac{2}{\pi}\sum_{n=0}^{\infty}\left(-\frac{q^2a^2}{4(z+1)^2}\right)^n\frac{4^n}{(n+1)}\frac{(z+s+1)_{2n}}{\left(\frac{3}{2}\right)_n n!}\sum_{m=0}^{\infty}\left(-\frac{q^2b^2}{4(z+1)^2}\right)^m\frac{4^m}{(m+1)}\frac{(z+s+1)_{2m}}{\left(\frac{3}{2}\right)_m m!}\sum_{l=0}^{\infty}\left(-\frac{q^2c^2}{4(z+1)^2}\right)^l\frac{4^l}{(l+1)}\frac{(z+s+1)_{2l}}{\left(\frac{3}{2}\right)_l l!}$$
$$\int_0^{\frac{\pi}{2}}\int_0^{\frac{\pi}{2}}(\cos\phi)^{2n}(\sin\phi)^{2m}(\sin\theta)^{2n+2m}(\cos\theta)^{2l}\sin\theta\, d\theta d\phi$$

With the ratios $\delta = b/a$ and $\varepsilon = c/a$ we obtain analogously **(S.4.5.1.3)**

$$\langle P(q)\rangle = \frac{\pi^{1/2}}{16\pi}\sum_{n=0}^{\infty}\frac{4^n}{\Gamma\left(n+\frac{3}{2}\right)}\left(-\frac{q^2a^2}{4(z+1)^2}\right)^n\sum_{m=0}^{n}\frac{(z+s+1)_{2(n-m)}}{\left(n-m+\frac{1}{2}\right)(n-m+1)!}\sum_{l=0}^{m}\frac{\delta^{2(m-l)}\varepsilon^{2l}(z+s+1)_{2(m-l)}}{\left(m-l+\frac{1}{2}\right)(m-l+1)!}\frac{(z+s+1)_{2l}}{\left(l+\frac{1}{2}\right)(l+1)!}$$

$$=\frac{\pi^{1/2}}{16\pi}\sum_{n=0}^{\infty}\frac{4^n}{\Gamma\left(n+\frac{3}{2}\right)}\left(-\frac{q^2a^2}{4(z+1)^2}\right)^n c_n$$

with

$$c_n = \sum_{m=0}^{n}\frac{(z+s+1)_{2(n-m)}}{\left(n-m+\frac{1}{2}\right)(n-m+1)!}\sum_{l=0}^{m}\frac{\delta^{2(m-l)}\varepsilon^{2l}(z+s+1)_{2(m-l)}}{\left(m-l+\frac{1}{2}\right)(m-l+1)!}\frac{(z+s+1)_{2l}}{\left(l+\frac{1}{2}\right)(l+1)!}$$

Parallelepiped, polydisperse, isotropic, P(q)

Regime II. (S.4.5.1.4)

$$\langle P_{cube}(q)\rangle_a = \frac{2}{\pi}\int_0^{\frac{\pi}{2}}\int_0^{\frac{\pi}{2}}\langle\left(\frac{\sin(q_xa)}{q_xa}\right)^2\rangle_a\langle\left(\frac{\sin(q_yb)}{q_yb}\right)^2\rangle_b\langle\left(\frac{\sin(q_zc)}{q_zc}\right)^2\rangle_c\sin\theta\, d\theta d\phi$$

$$\langle\left(\frac{\sin(q_xa)}{q_xa}\right)^2\rangle_a = \frac{\Gamma[z+s-1]}{\Gamma[z+s+1]}\frac{1}{2}u^{-2}\left(1-\frac{\cos[(z+s-n+1)\arctan(2u)]}{(1+4u^2)^{\frac{z+s-1}{2}}}\right)$$

$$u = \frac{q_ix}{z+1}$$

Regime III. The Porod asymptote is calculated from the surface area and the volume

$$A = 8(ab+ac+bc)$$

$$V = abc$$

With the average

$$\langle\frac{1}{x^4}\rangle = \frac{\Gamma(z+s-3)}{\Gamma(z+s+1)}\left(\frac{z+1}{x}\right)^4$$

With $s = 6$, such that we have for the Porod asymptote **(S.4.5.1.5)**

$$\lim_{q\to\infty} P(q) = \frac{2\pi}{q^4}\langle\frac{A}{V^2}\rangle = \frac{\Gamma(z+s-3)(z+1)^4}{\Gamma(z+s+1)}\frac{2\pi}{q^4}\frac{A}{V^2}$$

The following graph shows the formfactors from the series expansions compared to the numerical integrations and the Porod-asymptote. We note the overlap of the series expansion and the Porod-asymptote, such that numerical integration is only necessary at special conditions. As the coefficients can be precalculated, the series expansion is rapidly computed.

Mathematica code implementation:

```
a=1;
b=2;
c=3;
sigma=0.1;
z=(1-sigma*sigma)/(sigma*sigma);
area=8*(a*b+a*c+b*c);
vol=8*a*b*c;
nmax=80;
q=10^lq;
qs=Sqrt[qx*qx+qy*qy];
a1p=Gamma[z-1]/(2*Gamma[z+1]);
qa=q*a*Cos[phi]*Sin[theta]/(z+1);
qb=q*b*Sin[phi]*Sin[theta]/(z+1);
qc=q*c*Cos[theta]/(z+1);
fxp=a1p*(1/(qa*qa))*(1-Cos[(z-1)*ArcTan[2*qa]]/((1+4*qa*qa)^((z-1)/2)));
fyp=a1p*(1/(qb*qb))*(1-Cos[(z-1)*ArcTan[2*qb]]/((1+4*qb*qb)^((z-1)/2)));
fzp=a1p*(1/(qc*qc))*(1-Cos[(z-1)*ArcTan[2*qc]]/((1+4*qc*qc)^((z-1)/2)));
(* Pqq=NIntegrate[fxp*fyp*fzp*Sin[theta], {phi, 0, Pi/2}, {theta, 0, Pi/2}]/(Pi/2); *)
apor=Gamma[z-3]*((z+1)^4)/Gamma[z+1];
Pqpor=apor*2*Pi*area/((q^4)*vol*vol);
Pq1=(Sqrt[Pi]/16)*Sum[((4^n)*((-q*q/(4*(z+1)*(z+1)))^n)/Gamma[n+3/2])*Sum[(Pochhammer[z+1,2*(n-m)]*((a*a)^(n-
m))/((n-m+1/2)*((n-m+1)!)))*Sum[(Pochhammer[z+1,2*(m-l)]*((b*b)^(m-l))/((m-l+1/2)*((m-
l+1)!)))*(Pochhammer[z+1,2*l]*((c*c)^l)/((l+1/2)*((l+1)!))),{l, 0, m}], {m, 0, n}], {n, 0, nmax}];
Pq1s=(Sqrt[Pi]/16)*Sum[((4^n)*((-qs*qs/(4*(z+1)*(z+1)))^n)/Gamma[n+3/2])*Sum[(Pochhammer[z+1,2*(n-m)]*((a*a)^(n-
m))/((n-m+1/2)*((n-m+1)!)))*Sum[(Pochhammer[z+1,2*(m-l)]*((b*b)^(m-l))/((m-l+1/2)*((m-
l+1)!)))*(Pochhammer[z+1,2*l]*((c*c)^l)/((l+1/2)*((l+1)!))),{l, 0, m}], {m, 0, n}], {n, 0, nmax}];
lim=1.5;
pl2=Plot[Log[10,Pq1], {lq , -1,lim}, PlotRange->{-6,1}, GridLines->{Automatic}, LabelStyle->Directive[Black,16],AxesLabel-
>{"log q","lg P(q)"}, AxesOrigin->{-1,-6},TicksStyle->Directive[Black,12],PlotStyle->{Black,Dashed}] ;
(* pl1=Plot[Log[10,Pqq], {lq , -1,lim}, PlotRange□{-6,1}, GridLines->{Automatic}, LabelStyle->Directive[Black,16],AxesLabel-
>{"log q","lg P(q)"}, AxesOrigin->{-1,-6},TicksStyle->Directive[Black,12],PlotStyle->{Blue}]; *)
pl3=Plot[Log[10,Pqpor], {lq , -1,lim}, PlotRange->{-6,1}, GridLines->{Automatic}, LabelStyle->Directive[Black,16],AxesLabel-
>{"log q","lg P(q)"}, AxesOrigin->{-1,-6},TicksStyle->Directive[Black,10],PlotStyle->{Red}] ;
Show[pl2,pl1,pl3]
lims=6;
pl4=DensityPlot[Log[10,Pq1s],{qx, -lims, lims}, {qy, -lims, lims}, PlotRange->{-8,0}, PlotPoints->50, PlotLegends->Automatic,
LabelStyle->Directive[Black,12],AxesLabel->Automatic]
```

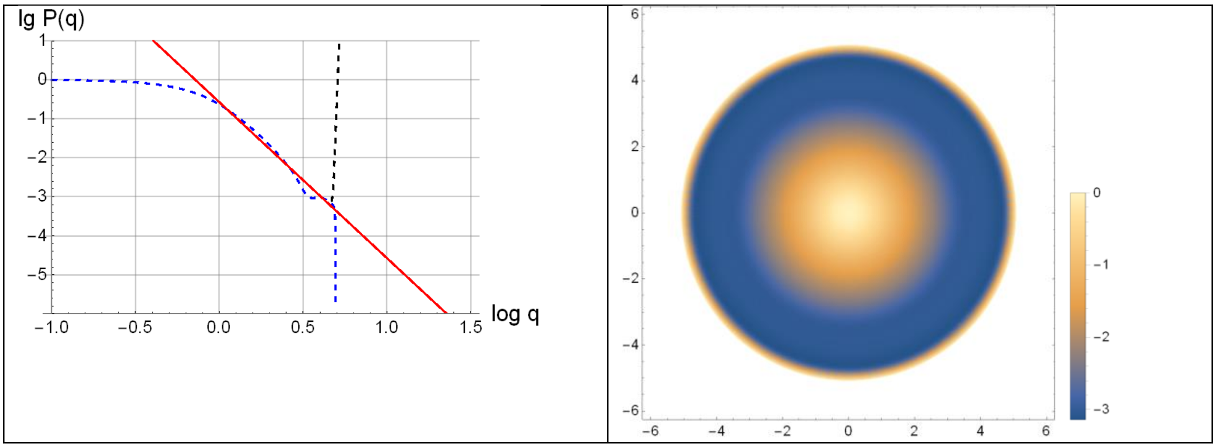

Fig. S16: Formfactor of a polydisperse isotropic parallelepipeds.

4.5.2. Scattering Amplitude F(q)

Integral representation. **(S.4.5.2.1)**

$$\langle F_{pa}(q)\rangle_{a,b,c}^2 = \frac{2}{\pi}\int_0^{\frac{\pi}{2}}\int_0^{\frac{\pi}{2}} \left(\langle F^{(1)}(q_x a)\rangle_a \langle F^{(1)}(q_y b)\rangle_b \langle F^{(1)}(q_z c)\rangle_c\right)^2 \sin\theta\, d\theta d\phi$$

$$= \frac{2}{\pi}\int_0^{\frac{\pi}{2}}\int_0^{\frac{\pi}{2}} \left(\int_0^{\infty} \frac{\sin(qa\sin\theta\cos\phi)}{qa\sin\theta\cos\phi} h(a)da \int_0^{\infty} \frac{\sin(qb\sin\theta\sin\phi)}{qb\sin\theta\sin\phi} h(b)db \int_0^{\infty} \frac{\sin(qc\cos\theta)}{qc\cos\theta} h(c)dc\right)^2 \sin\theta\, d\theta d\phi$$

Regime I: **(S.4.5.2.2)**

$$\langle F(q)\rangle^2 = \frac{2}{\pi}\sum_{n=0}^{\infty}\left(-\frac{q^2a^2(\sin\theta\cos\phi)^2}{4(z+1)^2}\right)^n f_n^{(1)} \sum_{m=0}^{\infty}\left(-\frac{q^2b^2(\sin\theta\sin\phi)^2}{4(z+1)^2}\right)^m f_m^{(1)} \sum_{l=0}^{\infty}\left(-\frac{q^2c^2(\cos\theta)^2}{4(z+1)^2}\right)^l f_l^{(1)}$$

$$\langle F(q)\rangle^2 = \frac{2}{\pi}\sum_{n=0}^{\infty}\left(-\frac{q^2a^2}{4(z+1)^2}\right)^n f_n^{(1)} \sum_{m=0}^{\infty}\left(-\frac{q^2b^2}{4(z+1)^2}\right)^m f_m^{(1)} \sum_{l=0}^{\infty}\left(-\frac{q^2c^2}{4(z+1)^2}\right)^l f_l^{(1)}$$
$$\int_0^{\frac{\pi}{2}}\int_0^{\frac{\pi}{2}} (\cos\phi)^{2n}\,(\sin\phi)^{2m}(\sin\theta)^{2n+2m}(\cos\theta)^{2l}\sin\theta\, d\theta d\phi$$

$$\int_0^{\frac{\pi}{2}}\int_0^{\frac{\pi}{2}} (\cos\phi)^{2n}\,(\sin\phi)^{2m}(\sin\theta)^{2n+2m}(\cos\theta)^{2l}\sin\theta\, d\theta d\phi = \frac{\Gamma\left(l+\frac{1}{2}\right)\Gamma\left(m+\frac{1}{2}\right)\Gamma\left(n+\frac{1}{2}\right)}{4\Gamma\left(n+m+l+\frac{3}{2}\right)}$$

$$\langle F(q)\rangle^2 = \frac{2}{\pi}\sum_{n=0}^{\infty}\left(-\frac{q^2a^2}{4(z+1)^2}\right)^n f_n^{(1)} \sum_{m=0}^{\infty}\left(-\frac{q^2b^2}{4(z+1)^2}\right)^m f_m^{(1)} \sum_{l=0}^{\infty}\left(-\frac{q^2c^2}{4(z+1)^2}\right)^l f_l^{(1)} \frac{\Gamma\left(l+\frac{1}{2}\right)\Gamma\left(m+\frac{1}{2}\right)\Gamma\left(n+\frac{1}{2}\right)}{4\Gamma\left(n+m+l+\frac{3}{2}\right)}$$

$$\langle F(q)\rangle^2 = \frac{2}{\pi}\sum_{n=0}^{\infty}\left(-\frac{q^2a^2}{4(z+1)^2}\right)^n \Gamma\left(n+\frac{1}{2}\right) f_n^{(1)} \sum_{m=0}^{\infty}\left(-\frac{q^2b^2}{4(z+1)^2}\right)^m \Gamma\left(m+\frac{1}{2}\right) f_m^{(1)} \sum_{l=0}^{\infty}\left(-\frac{q^2c^2}{4(z+1)^2}\right)^l f_l^{(1)} \frac{\Gamma\left(l+\frac{1}{2}\right)}{4\Gamma\left(n+m+l+\frac{3}{2}\right)}$$

$$\langle F(q)\rangle^2 = \frac{2}{\pi}\sum_{n=0}^{\infty}\left(-\frac{q^2a^2}{4(z+1)^2}\right)^n \Gamma\left(n+\frac{1}{2}\right) f_n^{(1)} \sum_{m=0}^{\infty}\left(-\frac{q^2a^2\delta^2}{4(z+1)^2}\right)^m \Gamma\left(m+\frac{1}{2}\right) f_m^{(1)} \sum_{l=0}^{\infty}\left(-\frac{q^2a^2\varepsilon^2}{4(z+1)^2}\right)^l f_l^{(1)} \frac{\Gamma\left(l+\frac{1}{2}\right)}{4\Gamma\left(n+m+l+\frac{3}{2}\right)}$$

$$\langle F(q)\rangle^2 = \frac{2}{\pi 4}\sum_{n=0}^{\infty}\sum_{m=0}^{n}\sum_{l=0}^{m}\left(-\frac{q^2a^2}{4(z+1)^2}\right)^{n-m} \Gamma\left(n-m+\frac{1}{2}\right) f_{n-m}^{(1)} \left(-\frac{q^2a^2\delta^2}{4(z+1)^2}\right)^{m-l} \Gamma\left(m-l+\frac{1}{2}\right) f_{m-l}^{(1)} \left(-\frac{q^2a^2\varepsilon^2}{4(z+1)^2}\right)^l f_l^{(1)} \frac{\Gamma\left(l+\frac{1}{2}\right)}{\Gamma\left(n-m+m-l+l+\frac{3}{2}\right)}$$

$$\langle F(q)\rangle^2 = \frac{1}{2\pi}\sum_{n=0}^{\infty}\frac{1}{\Gamma\left(n+\frac{3}{2}\right)}\left(-\frac{q^2a^2}{4(z+1)^2}\right)^n \sum_{m=0}^{n}\Gamma\left(n-m+\frac{1}{2}\right) f_{n-m}^{(1)} \sum_{l=0}^{m}\delta^{2(m-l)}\varepsilon^{2l} f_{m-l}^{(1)} f_l^{(1)} \Gamma\left(m-l+\frac{1}{2}\right)\Gamma\left(l+\frac{1}{2}\right)$$

with the final expression **(S.4.5.2.3)**

$$\langle F(q)\rangle^2 = \frac{1}{2\pi}\sum_{n=0}^{\infty}\frac{1}{\Gamma\left(n+\frac{3}{2}\right)}\left(-\frac{q^2a^2}{4(z+1)^2}\right)^n c_n$$

$$c_n = \sum_{m=0}^{n}\Gamma\left(n-m+\frac{1}{2}\right) f_{n-m}^{(1)} \sum_{l=0}^{m}\delta^{2(m-l)}\varepsilon^{2l} f_{m-l}^{(1)} f_l^{(1)} \Gamma\left(m-l+\frac{1}{2}\right)\Gamma\left(l+\frac{1}{2}\right)$$

Parallelepiped, polydisperse, isotropic, F(q)

Regime II: (S.4.5.2.4)

$$\langle F(q)\rangle^2 = \frac{2}{\pi}\int_0^{\pi/2}\int_0^{\pi/2}\left(\langle\frac{\sin(qa\sin\theta\cos\phi)}{qa\sin\theta\cos\phi}\rangle\langle\frac{\sin(qb\sin\theta\sin\phi)}{qb\sin\theta\sin\phi}\rangle\langle\frac{\sin(qc\cos\theta)}{qc\cos\theta}\rangle\right)^2 \sin\theta\, d\theta\, d\phi$$

where we need

$$\langle\frac{\sin(q_i x)}{q_i x}\rangle = \frac{\Gamma[z+s-1]}{\Gamma[z+s+1]}\left(\frac{q_i x}{z+1}\right)^{-2} \frac{\sin\left[(z+s-1)\arctan\left(\frac{q_i x}{z+1}\right)\right]}{\left(1+\left(\frac{q_i x}{z+1}\right)^2\right)^{\frac{z+s-1}{2}}}$$

Mathematica code implementation:

```
a=1;
b=2;
c=3;
sigma=0.1;
z=(1-sigma*sigma)/(sigma*sigma);
nmax=80;
```

```
q=10^lq;
a1f=Gamma[z]/(Gamma[z+1]);
qa=q*a*Cos[phi]*Sin[theta]/(z+1);
qb=q*b*Sin[phi]*Sin[theta]/(z+1);
qc=q*c*Cos[theta]/(z+1);
fxf=a1f*(1/qa)*Sin[z*ArcTan[qa]]/((1+qa*qa)^(z/2));
fyf=a1f*(1/qb)*Sin[z*ArcTan[qb]]/((1+qb*qb)^(z/2));
fzf=a1f*(1/qc)*Sin[z*ArcTan[qc]]/((1+qc*qc)^(z/2));
(* Fqq=NIntegrate[fxf*fxf*fyf*fyf*fzf*fzf*Sin[theta], {phi, 0, Pi/2}, {theta, 0, Pi/2}]/(Pi/2); *)
fff=Table[Sum[Pochhammer[z+1,2*(n-m)]*Pochhammer[z+1,2*m]/(Pochhammer[3/2,n-m]*Pochhammer[3/2,m]*((n-
m)!)*(m!)), {m, 0, n}],{n, 0, nmax}];
(* Fq7=(1/(2*Pi))*Sum[(Gamma[n+1/2]*((-q*q*a*a/(4*(z+1)*(z+1)))^n)*fff[[n+1]])*Sum[(Gamma[m+1/2]*((-
q*q*b*b/(4*(z+1)*(z+1)))^m)*fff[[m+1]])*Sum[(Gamma[l+1/2]*((-
q*q*c*c/(4*(z+1)*(z+1)))^l)*fff[[l+1]])/(Gamma[n+m+l+3/2]),{l, 0, nmax}], {m, 0, nmax}], {n, 0, nmax}];  *)
Fq1=(1/(2*Pi))*Sum[(((-q*q/(4*(z+1)*(z+1)))^n)/Gamma[n+3/2])*Sum[(Gamma[n-m+1/2]*((a*a)^(n-m))*fff[[n-
m+1]])*Sum[(Gamma[m-l+1/2]*Gamma[l+1/2]*((b*b)^(m-l))*((c*c)^l)*fff[[m-l+1]]*fff[[l+1]]),{l, 0, m}], {m, 0,n}], {n, 0,
nmax}];
Fq1s=(1/(2*Pi))*Sum[(((-qs*qs/(4*(z+1)*(z+1)))^n)/Gamma[n+3/2])*Sum[(Gamma[n-m+1/2]*((a*a)^(n-m))*fff[[n-
m+1]])*Sum[(Gamma[m-l+1/2]*Gamma[l+1/2]*((b*b)^(m-l))*((c*c)^l)*fff[[m-l+1]]*fff[[l+1]]),{l, 0, m}], {m, 0,n}], {n, 0,
nmax}];
lim=1.5;
pl2=Plot[Log[10,Fq1], {lq , -1,lim}, PlotRange->{-6,1}, GridLines->{Automatic}, LabelStyle->Directive[Black,16],AxesLabel-
>{"log q","lg F^2(q)"}, AxesOrigin->{-1,-6},TicksStyle->Directive[Black,12],PlotStyle->{Black,Dashed}] ;
(* pl1=Plot[Log[10,Fqq], {lq , -1,lim}, PlotRange□{-6,1}, GridLines->{Automatic}, LabelStyle->Directive[Black,16],AxesLabel-
>{"log q","lg F^2(q)"}, AxesOrigin->{-1,-6},TicksStyle->Directive[Black,12],PlotStyle->{Blue}]; *)
Show[pl2]
lims=5;
pl4=DensityPlot[Log[10,Fq1s],{qx, -lims, lims}, {qy, -lims, lims}, PlotRange->{-8,0}, PlotPoints->50, PlotLegends->Automatic,
LabelStyle->Directive[Black,12],AxesLabel->Automatic]
```

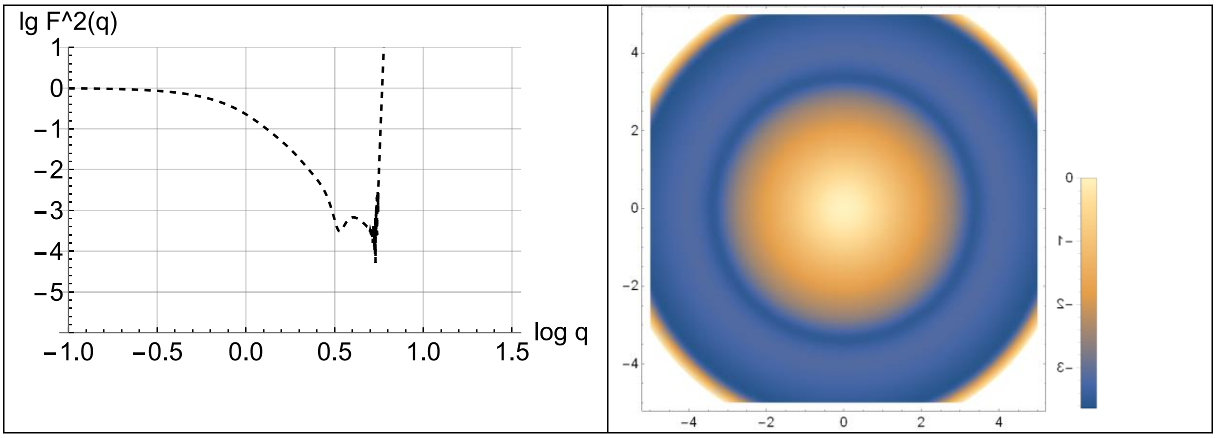

Fig. S17: Scattering amplitude of a polydisperse isotropic parallelepipeds.

4.6 Cylinders

The formfactor of cylinders is given by **(S.4.6.1)**

$$\langle P_{cyl}(q)\rangle_{L,R} = \int_0^{\frac{\pi}{2}} \langle P^{(1)}(q_z L)\rangle_L \langle P^{(2)}(q_r R)\rangle_R \sin\theta \, d\theta$$

$$q_z = q\cos\theta$$

$$q_r = q\sin\theta$$

with the longitudinal part

$$P^{(1)}(q_z L) = P_{\parallel}(q_z L) = \left(\frac{\sin(qL\cos\theta)}{qL\cos\theta}\right)^2$$

and the cross-sectional part

$$P^{(2)}(q_r R) = P_{\perp}(q_r R) = \left(\frac{2J_1(qR\sin\theta}{qR\sin\theta}\right)^2$$

Therefore, the cylinder formfactor can be expressed as (S.4.6.2)

$$\langle P_{cyl}(q)\rangle_{L,R} = \int_0^{\pi/2} \left(\int_0^{\infty} \left(\frac{\sin(qL\cos\theta)}{qL\cos\theta}\right)^2 L^{s_L} h(L) dL \int_0^{\infty} \left(\frac{2J_1(qR\sin\theta}{qR\sin\theta}\right)^2 R^{s_R} h(R) dR\right) \sin\theta \, d\theta$$

$$\langle P(q)\rangle = \int_0^{\pi/2} \langle\left(\frac{\sin(qL\cos\theta)}{qL\cos\theta}\right)^2\rangle_L \langle\left(\frac{2J_1(qR\sin\theta}{qR\sin\theta}\right)^2\rangle_R \sin\theta \, d\theta$$

$$\langle P(q)\rangle = \int_0^{\pi/2} \langle P_{\parallel}(qL\cos\theta)\rangle_L \langle P_{\perp}(qR\sin\theta)\rangle_R \sin\theta \, d\theta$$

with $s_L = 2$ and $s_R = 4$.

4.6.1 Small axial ratios

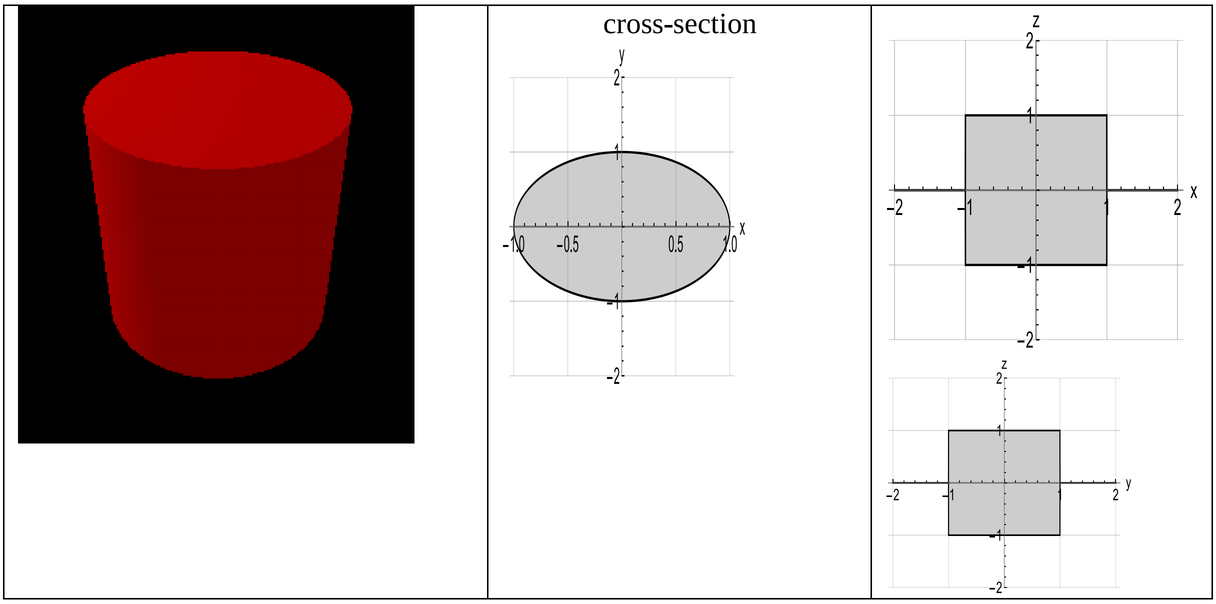

Fig. S18: Cylinder with cross-sections in the (*x*,*y*)-, (*x*,*z*)-, and (*y*,*z*)-planes.

4.6.1.1 Formfactor

For small axial ratios the cylinder formfactor needs to be numerically integrated as (S.4.6.1.1.1)

$$\langle P(q)\rangle = \int_0^{\pi/2} \langle\left(\frac{\sin(qL\cos\theta)}{qL\cos\theta}\right)^2\rangle_L \langle\left(\frac{2J_1(qR\sin\theta)}{qR\sin\theta}\right)^2\rangle_R \sin\theta\, d\theta$$

Regime I. With the series expansions (S.4.6.1.1.2)

$$\langle\left(\frac{\sin(qL\cos\theta)}{qL\cos\theta}\right)^2\rangle_L = \sum_{n=0}^{\infty}\frac{4^n}{(\mathrm{n}+1)}\frac{(z_L+s_L+1)_{2n}}{\left(\frac{3}{2}\right)_n n!}\left(-\frac{q^2L^2(\cos(\theta))^2}{4(z_L+1)^2}\right)^n$$

and (S.4.6.1.1.3)

$$\langle\left(\frac{2J_1(qR\sin\theta)}{qR\sin\theta}\right)^2\rangle_R = \sum_{n=0}^{\infty}\frac{4^{n+1}\Gamma\left(n+\frac{3}{2}\right)}{\sqrt{\pi}\Gamma(n+3)}\frac{(z_R+s_R+1)_{2n}}{(2)_n n!}\left(-\frac{q^2R^2(\sin(\theta))^2}{4(z_R+1)^2}\right)^n$$

we have for the formfactor

$$P(q) = \sum_{n=0}^{\infty}\frac{4^n}{(\mathrm{n}+1)}\frac{(z_L+s_L+1)_{2n}}{\left(\frac{3}{2}\right)_n n!}\left(-\frac{q^2L^2(\cos(\theta))^2}{4(z_L+1)^2}\right)^n \sum_{m=0}^{\infty}\frac{4^{m+1}\Gamma\left(m+\frac{3}{2}\right)}{\sqrt{\pi}\Gamma(m+3)}\frac{(z_R+s_R+1)_{2m}}{(2)_m m!}\left(-\frac{q^2R^2(\sin(\theta))^2}{4(z_R+1)^2}\right)^m$$

which we need to integrate over the angles (S.4.6.1.1.4)

$$P(q) = \sum_{n=0}^{\infty} \frac{4^n}{(\mathrm{n}+1)} \frac{(z_L+s_L+1)_{2n}}{\left(\frac{3}{2}\right)_n n!} \left(-\frac{q^2L^2}{4(z_L+1)^2}\right)^n \sum_{m=0}^{\infty} \frac{4^{m+1}\Gamma\left(m+\frac{3}{2}\right)}{\sqrt{\pi}\Gamma(m+3)} \frac{(z_R+s_R+1)_{2m}}{(2)_m m!} \left(-\frac{q^2R^2}{4(z_R+1)^2}\right)^m \int_0^{\frac{\pi}{2}} (\cos(\theta))^{2n}(\sin(\theta))^{2m}\sin\theta\, d\theta$$

$$\int_0^{\frac{\pi}{2}} (\cos(\theta))^{2n}(\sin(\theta))^{2m+1} d\theta = \frac{2}{\pi}\frac{\Gamma\left(n+\frac{1}{2}\right)\Gamma(m+1)}{2\Gamma\left(n+m+\frac{3}{2}\right)} = \frac{\Gamma\left(n+\frac{1}{2}\right)\mathrm{m}!}{2\Gamma\left(n+m+\frac{3}{2}\right)}$$

$$= \sum_{n=0}^{\infty} \frac{4^n}{(\mathrm{n}+1)} \frac{(z_L+s_L+1)_{2n}}{\left(\frac{3}{2}\right)_n n!} \left(-\frac{q^2L^2}{4(z_L+1)^2}\right)^n \sum_{m=0}^{\infty} \frac{4^{m+1}\Gamma\left(m+\frac{3}{2}\right)}{\sqrt{\pi}\Gamma(m+3)} \frac{(z_R+s_R+1)_{2m}}{(2)_m m!} \left(-\frac{q^2R^2}{4(z_R+1)^2}\right)^m \frac{\Gamma\left(n+\frac{1}{2}\right)\Gamma(m+1)}{2\Gamma\left(n+m+\frac{3}{2}\right)}$$

$$= \sum_{n=0}^{\infty} \frac{4^n}{(\mathrm{n}+1)} \frac{(z_L+s_L+1)_{2n}}{\left(\frac{3}{2}\right)_n n!} \left(-\frac{q^2L^2}{4(z_L+1)^2}\right)^n \sum_{m=0}^{\infty} \frac{4^{m+1}\Gamma\left(m+\frac{3}{2}\right)}{\sqrt{\pi}\Gamma(m+3)} \frac{(z_R+s_R+1)_{2m}}{(2)_m m!} \left(-\frac{q^2R^2}{4(z_R+1)^2}\right)^m \frac{\Gamma\left(n+\frac{1}{2}\right)\mathrm{m}!}{2\Gamma\left(n+m+\frac{3}{2}\right)}$$

$$= \sum_{n=0}^{\infty} \frac{4^n\Gamma\left(n+\frac{1}{2}\right)}{(\mathrm{n}+1)} \frac{(z_L+s_L+1)_{2n}}{\left(\frac{3}{2}\right)_n n!} \left(-\frac{q^2L^2}{4(z_L+1)^2}\right)^n \sum_{m=0}^{\infty} \frac{4^{m+1}\Gamma\left(m+\frac{3}{2}\right)}{\sqrt{\pi}\Gamma(m+3)} \frac{(z_R+s_R+1)_{2m}}{(2)_m m!} \left(-\frac{q^2R^2}{4(z_R+1)^2}\right)^m \frac{\mathrm{m}!}{2\Gamma\left(n+m+\frac{3}{2}\right)}$$

$$= \sum_{n=0}^{\infty} \frac{4^n\Gamma\left(n+\frac{1}{2}\right)}{\left(n+\frac{1}{2}\right)} \frac{\Gamma\left(\frac{3}{2}\right)(z_L+s_L+1)_{2n}}{\Gamma\left(n+\frac{1}{2}\right)(n+1)!} \left(-\frac{q^2L^2}{4(z_L+1)^2}\right)^n \sum_{m=0}^{\infty} \frac{4^{m+1}\Gamma\left(m+\frac{3}{2}\right)}{\sqrt{\pi}(\mathrm{m}+2)!} \frac{\Gamma(2)(z_R+s_R+1)_{2m}}{\Gamma(m+2)} \left(-\frac{q^2R^2}{4(z_R+1)^2}\right)^m \frac{1}{2\Gamma\left(n+m+\frac{3}{2}\right)}$$

$$= \sum_{n=0}^{\infty} \frac{4^n}{\left(n+\frac{1}{2}\right)} \frac{\sqrt{\pi}(z_L+s_L+1)_{2n}}{2(n+1)!} \left(-\frac{q^2L^2}{4(z_L+1)^2}\right)^n \sum_{m=0}^{\infty} \frac{4^{m+1}\Gamma\left(m+\frac{3}{2}\right)}{\sqrt{\pi}(\mathrm{m}+2)!} \frac{(z_R+s_R+1)_{2m}}{(\mathrm{m}+1)!} \left(-\frac{q^2R^2}{4(z_R+1)^2}\right)^m \frac{1}{2\Gamma\left(n+m+\frac{3}{2}\right)}$$

$$= \frac{1}{4}\sum_{n=0}^{\infty} \frac{4^n(z_L+s_L+1)_{2n}}{\left(n+\frac{1}{2}\right)(n+1)!} \left(-\frac{q^2L^2}{4(z_L+1)^2}\right)^n \sum_{m=0}^{\infty} \frac{4^{m+1}\Gamma\left(m+\frac{3}{2}\right)(z_R+s_R+1)_{2m}}{\Gamma\left(n+m+\frac{3}{2}\right)(\mathrm{m}+2)!\,(\mathrm{m}+1)!} \left(-\frac{q^2R^2}{4(z_R+1)^2}\right)^m$$

Cylinder, polydisperse, short axial ratio, isotropic, P(q)

Not clear if this converges

$$= \frac{1}{4}\sum_{n=0}^{\infty}\sum_{m=0}^{n} \frac{4^{n-m}(z_L+s_L+1)_{2(n-m)}}{\left(n-m+\frac{1}{2}\right)(n-m+1)!} \left(-\frac{q^2L^2}{4(z_L+1)^2}\right)^{n-m} \frac{4^{m+1}\Gamma\left(m+\frac{3}{2}\right)(z_R+s_R+1)_{2m}}{\Gamma\left(n-m+m+\frac{3}{2}\right)(\mathrm{m}+2)!\,(\mathrm{m}+1)!} \left(-\frac{q^2R^2}{4(z_R+1)^2}\right)^m$$

Regime II.

We need to integrate (S.4.6.1.1.5)

$$\langle P(q)\rangle = \int_0^{\pi/2} \langle\left(\frac{\sin(qL\cos\theta)}{qL\cos\theta}\right)^2\rangle_L \langle\left(\frac{2J_1(qR\sin\theta}{qR\sin\theta}\right)^2\rangle_R \sin\theta\, d\theta$$

Where in the integrand we use the Regime I,II,III-parts. We note the asymptotes

$$\langle\left(\frac{\sin(q_x a)}{q_x a}\right)^2\rangle_a = \frac{\Gamma[z+s-1]}{\Gamma[z+s+1]}\frac{1}{2}u^{-2}\left(1-\frac{\cos[(z+s-n+1)\arctan(2u)]}{(1+4u^2)^{\frac{z+s-1}{2}}}\right)$$

$$u = \frac{q_i x}{z+1}$$

$$\langle\frac{4(J_1(qR\sin\theta)^2}{q^2(R\sin\theta)^2}\rangle = \frac{4}{\pi}\left(\left(\frac{1}{qR}\right)^3 - \left(\frac{1}{qR}\right)^3\sin(2qR) - \frac{9}{8}\left(\frac{1}{qR}\right)^4\cos(2qR) + \left(\frac{9}{16}\right)^2\left(\frac{1}{qR}\right)^5 + \left(\frac{9}{16}\right)^2\left(\frac{1}{qR}\right)^5\sin(2qR)\right)$$

$$\langle(qR)^{-3}\rangle = \frac{\Gamma[z+s-2]}{\Gamma[z+s+1]}\left(\frac{qR}{z+1}\right)^{-3}$$

$$\langle\frac{\sin(2qR)}{(qR)^3}\rangle = \frac{\Gamma[z+s-2]}{\Gamma[z+s+1]}\left(\frac{qR}{z+1}\right)^{-3}\frac{\sin\left[(z+s-2)\arctan\left(\frac{2qR}{z+1}\right)\right]}{\left(1+\left(\frac{2qR}{z+1}\right)^2\right)^{\frac{z+s-2}{2}}}$$

$$\langle\frac{\cos(2qR)}{(qR)^4}\rangle = \frac{\Gamma[z+s-3]}{\Gamma[z+s+1]}\left(\frac{qR}{z+1}\right)^{-4}\frac{\cos\left[(z+s-3)\arctan\left(\frac{2qR}{z+1}\right)\right]}{\left(1+\left(\frac{2qR}{z+1}\right)^2\right)^{\frac{z+s-3}{2}}}$$

$$\langle(qR)^{-5}\rangle = \frac{\Gamma[z+s-4]}{\Gamma[z+s+1]}\left(\frac{x}{z+1}\right)^{-4}$$

$$\langle\frac{\sin(2qR)}{(qR)^5}\rangle = \frac{\Gamma[z+s-4]}{\Gamma[z+s+1]}\left(\frac{qR}{z+1}\right)^{-5}\frac{\sin\left[(z+s-4)\arctan\left(\frac{2qR}{z+1}\right)\right]}{\left(1+\left(\frac{2qR}{z+1}\right)^2\right)^{\frac{z+s-4}{2}}}$$

Regime III.

We calculate the Porod asymptote from the surface-to-volume ratio as (S.4.6.1.1.6)

$$P(q) = \frac{\Gamma(z+s-3)(z+1)^4}{\Gamma(z+s+1)}\frac{2\pi}{q^4}\frac{A}{V^2}$$

$$A = 2\pi R^2 + 2\pi R(2L)$$

$$V = \pi R^2(2L)$$

with $s = 6$ and using (2L), because L was the semiaxis in the calculations.

Mathematica code implementation:

```
L=1.5;
R=1;
sigma=0.1;
z=(1-sigma*sigma)/(sigma*sigma);
nmax=120;
q=10^lq;
qs=Sqrt[qx*qx+qy*qy];
(* Pqa=Sum[((4^n)*Pochhammer[z+1,2*n]*((-q*q*L*L/(4*(z+1)*(z+1)))^n)/((n+1)*Pochhammer[3/2,n]*(n!))), {n, 0, nmax}];
Pqb=Sum[((4^(m+1))*Gamma[m+3/2]*Pochhammer[z+1,2*m]*((-
q*q*R*R/(4*(z+1)*(z+1)))^m)/(Sqrt[Pi]*Gamma[m+3]*Pochhammer[2,m]*(m!))), {m, 0, nmax}];
Pqc=Sum[((4^n)*Pochhammer[z+1,2*n]*((-
q*q*L*L/(4*(z+1)*(z+1)))^n)/((n+1)*Pochhammer[3/2,n]*(n!)))*Sum[((4^(m+1))*Gamma[m+3/2]*Pochhammer[z+1,2*m]*(
(-
q*q*R*R/(4*(z+1)*(z+1)))^m)*Gamma[n+1/2]*(m!)/(Sqrt[Pi]*Gamma[m+3]*Pochhammer[2,m]*(m!)*2*Gamma[n+m+3/2]
)), {m, 0, nmax}], {n, 0, nmax}];  *)
Pq1=(1/4)*Sum[((4^(n+1))*((-q*q/(4*(z+1)*(z+1)))^n)/Gamma[n+3/2])*Sum[Pochhammer[z+1,2*(n-
m)]*Pochhammer[z+1,2*m]*Gamma[m+3/2]*(L^(2*(n-m)))*(R^(2*m))/((n-m+1)*(n-m+1/2)*((n-m)!)*((m+2)!)*((m+1)!)),
{m, 0, n}],{n, 0, nmax}];
Pq1s=(1/4)*Sum[((4^(n+1))*((-qs*qs/(4*(z+1)*(z+1)))^n)/Gamma[n+3/2])*Sum[Pochhammer[z+1,2*(n-
m)]*Pochhammer[z+1,2*m]*Gamma[m+3/2]*(L^(2*(n-m)))*(R^(2*m))/((n-m+1)*(n-m+1/2)*((n-m)!)*((m+2)!)*((m+1)!)),
{m, 0, n}],{n, 0, nmax}];
area=2*Pi*R*R+2*Pi*R*(2*L);
vol=Pi*R*R*(2*L);
apor=Gamma[z-3]*((z+1)^4)/Gamma[z+1];
Pqpor=apor*2*Pi*area/((q^4)*vol*vol);
(* ql=(q*L*Cos[theta])/(z+1);
qr=(q*R*Sin[theta])/(z+1);
flmon=(Sin[ql]/ql)^2;
frmon=(2*BesselJ[1,qr]/qr)^2;
Pqintmon=NIntegrate[flmon*Sin[theta], {theta, 0, Pi/2}];
fl=(Gamma[z-1]/(2*Gamma[z+1]))*(ql^(-2))*(1-Cos[(z-1)*ArcTan[2*ql]]/((1+4*ql*ql)^((z-1)/2)));
fr1=(Gamma[z-2]/(Gamma[z+1]))*(qr^(-3));
fr2=(Gamma[z-2]/(Gamma[z+1]))*(qr^(-3))*Sin[(z-2)*ArcTan[2*qr]]/((1+4*qr*qr)^((z-2)/2));
fr3=(Gamma[z-3]/(Gamma[z+1]))*(qr^(-4))*Cos[(z-3)*ArcTan[2*qr]]/((1+4*qr*qr)^((z-3)/2));
fr=(4/Pi)*(fr1-fr2-(9/8)*fr3);
Pqint=NIntegrate[fl*fr*Sin[theta], {theta, 0, Pi/2}]; *)
lim=1.5;
pl2=Plot[Log[10,Pq1], {lq , -1,lim}, PlotRange->{-6,1}, GridLines->{Automatic}, LabelStyle->Directive[Black,16],AxesLabel-
>{"log q","lg P(q)"}, AxesOrigin->{-1,-6},TicksStyle->Directive[Black,12],PlotStyle->{Black,Dashed}] ;
(* pl1=Plot[Log[10,Pqc], {lq , -1,lim}, PlotRange[illegible]{-6,1}, GridLines->{Automatic}, LabelStyle->Directive[Black,16],AxesLabel-
>{"log q","lg P(q)"}, AxesOrigin->{-1,-6},TicksStyle->Directive[Black,12],PlotStyle->{Blue}]; *)
pl3=Plot[Log[10,Pqpor], {lq , -1,lim}, PlotRange->{-6,1}, GridLines->{Automatic}, LabelStyle->Directive[Black,16],AxesLabel-
>{"log q","lg P(q)"}, AxesOrigin->{-1,-6},TicksStyle->Directive[Black,10],PlotStyle->{Red}] ;
Show[pl2,pl3]
lims=9;
pl4=DensityPlot[Log[10,Pq1s],{qx, -lims, lims}, {qy, -lims, lims}, PlotRange->{-8,0}, PlotPoints->50, PlotLegends->Automatic,
LabelStyle->Directive[Black,12],AxesLabel->Automatic]
```

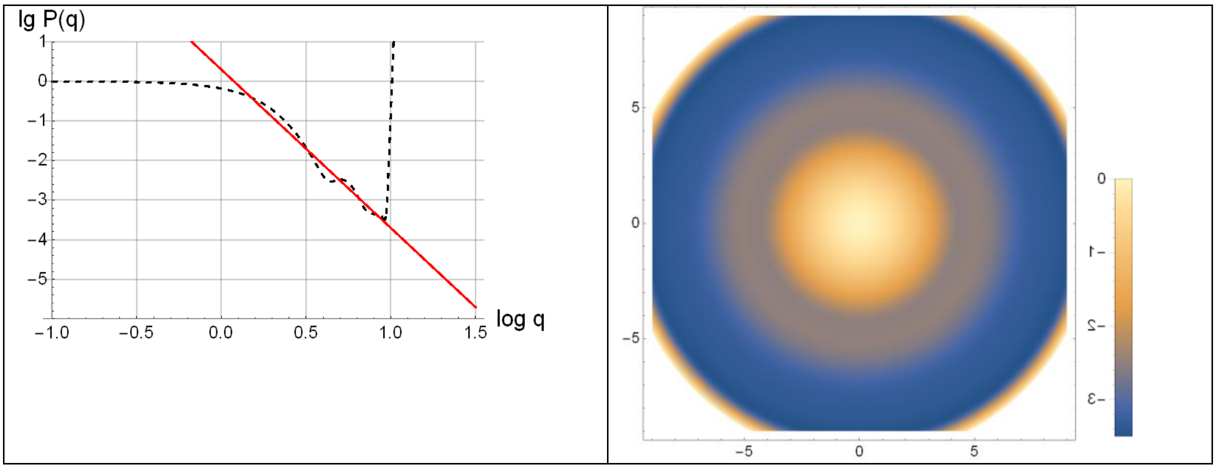

Fig. S19: Exact formfactor of polydisperse isotropic cylinders with circular cross-section.

The figure shows calculations for small axial ratios L/R=2, 1, 0.5 where Regimes I and III overlap and therefore calculations are possible without numerical integration. For larger axial ratios numerical integration over the size averaged sine and Bessel functions is possible by switching between the respective series and asymptotic expansions within the integration unit. The Respective equations are provided in the Supporting Information.

4.6.1.2 Scattering Amplitude F(q)

(S.4.6.1.2.1)

$$\langle F_{cyl}(q)\rangle_{L,R}^2 = \int_0^{\frac{\pi}{2}} \left(\langle F^{(1)}(q_z L)\rangle_L \langle F^{(2)}(q_r R)\rangle_R\right)^2 \sin\theta \, d\theta$$

with the longitudinal part

$$F^{(1)}(q_z L) = F_{\parallel}(q_z L) = \frac{\sin(qL\cos\theta)}{qL\cos\theta}$$

and the cross-sectional part

$$F^{(2)}(q_r R) = F_{\perp}(q_r R) = \frac{2J_1(qR\sin\theta)}{qR\sin\theta}$$

$$\langle F^2(q)\rangle = \int_0^{\frac{\pi}{2}} \left(\int_0^{\infty} \frac{\sin(qL\cos\theta)}{qL\cos\theta} L^{s_L} h(L) dL\right)^2 \left(\int_0^{\infty} \frac{2J_1(qR\sin\theta)}{qR\sin\theta} R^{s_R} h(R) dR\right)^2 \sin\theta \, d\theta$$

$$= \int_0^{\frac{\pi}{2}} \langle \frac{\sin(qL\cos\theta)}{qL\cos\theta} \rangle_L^2 \langle \frac{2J_1(qR\sin\theta)}{qR\sin\theta} \rangle_R^2 \sin\theta \, d\theta$$

With $s_L = 1$ and $s_R = 2$.

Regime I. (S.4.6.1.2.2)

$$\left(\langle F^{(1)}(q_z L)\rangle_L\right)^2 = \sum_{n=0}^{\infty} \left(-\frac{(qL\cos\theta)^2}{4(z_L+1)^2}\right)^n \sum_{m=0}^{n} \frac{(z_L+s_L+1)_{2(n-m)}(z_L+s_L+1)_{2m}}{\left(\frac{3}{2}\right)_{n-m}(n-m)!\left(\frac{3}{2}\right)_n n!}$$

$$\left(\langle F^{(2)}(q_r R)\rangle_R\right)^2 = \sum_{n=0}^{\infty} \left(-\frac{(qR\sin\theta)^2}{4(z_R+1)^2}\right)^n \sum_{m=0}^{n} \frac{(z_R+s_R+1)_{2(n-m)}(z_R+s_R+1)_{2m}}{(2)_{n-m}(n-m)!\,(2)_n n!}$$

$$\langle F_{cyl}(q)\rangle_{L,R}^2 = \left(\sum_{n=0}^{\infty} \left(-\frac{(qL\cos\theta)^2}{4(z_L+1)^2}\right)^n \sum_{m=0}^{n} \frac{(z_L+s_L+1)_{2(n-m)}(z_L+s_L+1)_{2m}}{\left(\frac{3}{2}\right)_{n-m}(n-m)!\left(\frac{3}{2}\right)_n n!}\right)\left(\sum_{n=0}^{\infty} \left(-\frac{(qR\sin\theta)^2}{4(z_R+1)^2}\right)^n \sum_{m=0}^{n} \frac{(z_R+s_R+1)_{2(n-m)}(z_R+s_R+1)_{2m}}{(2)_{n-m}(n-m)!\,(2)_n n!}\right)$$

$$\langle F_{cyl}(q)\rangle_{L,R}^2 = \left(\sum_{n=0}^{\infty} \left(-\frac{(qL)^2}{4(z_L+1)^2}\right)^n \sum_{m=0}^{n} \frac{(z_L+s_L+1)_{2(n-m)}(z_L+s_L+1)_{2,}}{\left(\frac{3}{2}\right)_{n-m}(n-m)!\left(\frac{3}{2}\right)_n n!}\right)\left(\sum_{n=0}^{\infty} \left(-\frac{(qR)^2}{4(z_R+1)^2}\right)^n \sum_{m=0}^{n} \frac{(z_R+s_R+1)_{2(n-m)}(z_R+s_R+1)_{2m}}{(2)_{n-m}(n-m)!\,(2)_n n!}\right) \int_0^{\frac{\pi}{2}} (\cos(\theta))^{2n}(\sin(\theta))^{2m}\sin\theta\, d\theta$$

$$\int_0^{\frac{\pi}{2}} (\cos(\theta))^{2n}(\sin(\theta))^{2m+1} d\theta = \frac{\Gamma\left(n+\frac{1}{2}\right)\mathrm{m}!}{2\Gamma\left(n+m+\frac{3}{2}\right)}$$

$$\langle F_{cyl}(q)\rangle_{L,R}^2 = \left(\sum_{n=0}^{\infty} \left(-\frac{(qL)^2}{4(z_L+1)^2}\right)^n \sum_{n'=0}^{n} \frac{(z_L+s_L+1)_{2(n-n')}(z_L+s_L+1)_{2n',}}{\left(\frac{3}{2}\right)_{n-n'}(n-n')!\left(\frac{3}{2}\right)_{n'} n'!}\right)\left(\sum_{m=0}^{\infty} \left(-\frac{(qR)^2}{4(z_R+1)^2}\right)^m \sum_{m'=0}^{m} \frac{(z_R+s_R+1)_{2(m-m')}(z_R+s_R+1)_{2m'}}{(2)_{m-m'}(m-m')!\,(2)_{m'} m'!}\right)\frac{\Gamma\left(n+\frac{1}{2}\right)\mathrm{m}!}{2\Gamma\left(n+m+\frac{3}{2}\right)}$$

$$= \sum_{n=0}^{\infty} \Gamma\left(n+\frac{1}{2}\right)\left(-\frac{(qL)^2}{4(z_L+1)^2}\right)^n \sum_{n'=0}^{n} \frac{(z_L+s_L+1)_{2(n-n')}(z_L+s_L+1)_{2n',}}{\left(\frac{3}{2}\right)_{n-n'}(n-n')!\left(\frac{3}{2}\right)_{n'} n'!} \sum_{m=0}^{\infty} \frac{\mathrm{m}!}{2\Gamma\left(n+m+\frac{3}{2}\right)}\left(-\frac{(qR)^2}{4(z_R+1)^2}\right)^n \sum_{m'=0}^{m} \frac{(z_R+s_R+1)_{2(m-m')}(z_R+s_R+1)_{2m'}}{(2)_{m-m'}(m-m')!\,(2)_{m'} m'!}$$

Cylinder, polydisperse, short axial ratio, isotropic, F(q)

Regime II.

Here, Eq. (S.4.6.1.2.1)

$$\langle F_{cyl}(q)\rangle^2_{L,R} = \int_0^{\frac{\pi}{2}} \langle\frac{\sin(qL\cos\theta)}{qL\cos\theta}\rangle^2_L \langle\frac{2J_1(qR\sin\theta)}{qR\sin\theta}\rangle^2_R \sin\theta\, d\theta$$

needs to be numerically integrated. The averages are

$$\langle\frac{\sin(qL\cos\theta)}{qL\cos\theta}\rangle^2_L = \frac{\Gamma[z+s-1]}{\Gamma[z+s+1]}\left(\frac{qL\cos\theta}{z+1}\right)^{-2} \frac{\sin\left[(z+s-1)\arctan\left(\frac{qL\cos\theta}{z+1}\right)\right]}{\left(1+\left(\frac{qL\cos\theta}{z+1}\right)^2\right)^{\frac{z+s-1}{2}}}$$

$$\langle\frac{2J_1(qR\sin\theta)}{qR\sin\theta}\rangle^2_R = \frac{4}{\pi}\left(\langle\frac{\sin(qR\sin\theta)}{(qR\sin\theta)^{3/2}}\rangle_R - \langle\frac{\cos(qR\sin\theta)}{(qR\sin\theta)^{3/2}}\rangle_R + \frac{9}{16}\left(\langle\frac{\cos(qR\sin\theta)}{(qR\sin\theta)^{5/2}}\rangle_R + \langle\frac{\sin(qR\sin\theta)}{(qR\sin\theta)^{5/2}}\rangle_R\right)\right)^2$$

$$\langle\frac{\sin(qR\sin\theta)}{(qR\sin\theta)^{3/2}}\rangle_R = \frac{\Gamma[z-\frac{1}{2}]}{\Gamma[z+1]}\left(\frac{qR\sin\theta}{z+1}\right)^{-3/2} \frac{\sin\left[\left(z-\frac{1}{2}\right)\arctan\left(\frac{qR\sin\theta}{z+1}\right)\right]}{\left(1+\left(\frac{qR\sin\theta}{z+1}\right)^2\right)^{\frac{z-\frac{1}{2}}{2}}}$$

$$\langle\frac{\cos(qR\sin\theta)}{(qR\sin\theta)^{3/2}}\rangle_R = \frac{\Gamma[z-\frac{1}{2}]}{\Gamma[z+1]}\left(\frac{qR\sin\theta}{z+1}\right)^{-3/2} \frac{\cos\left[\left(z-\frac{1}{2}\right)\arctan\left(\frac{qR\sin\theta}{z+1}\right)\right]}{\left(1+\left(\frac{qR\sin\theta}{z+1}\right)^2\right)^{\frac{z-\frac{1}{2}}{2}}}$$

$$\langle\frac{\sin(qR\sin\theta)}{(qR\sin\theta)^{5/2}}\rangle_R = \frac{\Gamma[z-\frac{3}{2}]}{\Gamma[z+1]}\left(\frac{qR\sin\theta}{z+1}\right)^{-5/2} \frac{\sin\left[\left(z-\frac{3}{2}\right)\arctan\left(\frac{qR\sin\theta}{z+1}\right)\right]}{\left(1+\left(\frac{qR\sin\theta}{z+1}\right)^2\right)^{\frac{z-\frac{3}{2}}{2}}}$$

$$\langle\frac{\cos(qR\sin\theta)}{(qR\sin\theta)^{5/2}}\rangle_R = \frac{\Gamma[z-\frac{3}{2}]}{\Gamma[z+1]}\left(\frac{qR\sin\theta}{z+1}\right)^{-5/2} \frac{\cos\left[\left(z-\frac{3}{2}\right)\arctan\left(\frac{qR\sin\theta}{z+1}\right)\right]}{\left(1+\left(\frac{qR\sin\theta}{z+1}\right)^2\right)^{\frac{z-\frac{3}{2}}{2}}}$$

Mathematica code implementation:

```
L=1.5;
R=1;
sigma=0.1;
z=(1-sigma*sigma)/(sigma*sigma);
nmax=70;
q=10^lq;
qs=Sqrt[qx*qx+qy*qy];
ffl=Table[Sum[Pochhammer[z+1,2*(n-m)]*Pochhammer[z+1,2*m]/(Pochhammer[3/2,n-m]*Pochhammer[3/2,m]*((n-
m)!)*(m!)), {m, 0, n}],{n, 0, nmax}];
ffr=Table[Sum[Pochhammer[z+1,2*(n-m)]*Pochhammer[z+1,2*m]/(Pochhammer[2,n-m]*Pochhammer[2,m]*((n-
m)!)*(m!)), {m, 0, n}],{n, 0, nmax}];
(* Pqa=Sum[((4^n)*Pochhammer[z+1,2*n]*((-q*q*L*L/(4*(z+1)*(z+1)))^n)/((n+1)*Pochhammer[3/2,n]*(n!))), {n, 0, nmax}];
Pqb=Sum[((4^(m+1))*Gamma[m+3/2]*Pochhammer[z+1,2*m]*((-
q*q*R*R/(4*(z+1)*(z+1)))^m)/(Sqrt[Pi]*Gamma[m+3]*Pochhammer[2,m]*(m!))), {m, 0, nmax}];
```

```
Pqc=Sum[((4^n)*Pochhammer[z+1,2*n]*((-
q*q*L*L/(4*(z+1)*(z+1)))^n)/((n+1)*Pochhammer[3/2,n]*(n!)))*Sum[((4^(m+1))*Gamma[m+3/2]*Pochhammer[z+1,2*m]*(
(-
q*q*R*R/(4*(z+1)*(z+1)))^m)*Gamma[n+1/2]*(m!)/(Sqrt[Pi]*Gamma[m+3]*Pochhammer[2,m]*(m!)*2*Gamma[n+m+3/2]
)), {m, 0, nmax}], {n, 0, nmax}];  *)
Fq1=(1/2)*Sum[(Gamma[n+1/2]*((-q*q*L*L/(4*(z+1)*(z+1)))^n)*ffl[[n+1]])*Sum[((m!)*((-
q*q*R*R/(4*(z+1)*(z+1)))^m)*ffr[[m+1]]/Gamma[n+m+3/2]), {m, 0, nmax}],{n, 0, nmax}];
Fq1s=(1/2)*Sum[(Gamma[n+1/2]*((-qs*qs*L*L/(4*(z+1)*(z+1)))^n)*ffl[[n+1]])*Sum[((m!)*((-
qs*qs*R*R/(4*(z+1)*(z+1)))^m)*ffr[[m+1]]/Gamma[n+m+3/2]), {m, 0, nmax}],{n, 0, nmax}];
(* ql=(q*L*Cos[theta])/(z+1);
qr=(q*R*Sin[theta])/(z+1);
flmon=(Sin[ql]/ql)^2;
frmon=(2*BesselJ[1,qr]/qr)^2;
Pqintmon=NIntegrate[flmon*Sin[theta], {theta, 0, Pi/2}];
fl=(Gamma[z-1]/(2*Gamma[z+1]))*(ql^(-2))*(1-Cos[(z-1)*ArcTan[2*ql]]/((1+4*ql*ql)^((z-1)/2)));
fr1=(Gamma[z-2]/(Gamma[z+1]))*(qr^(-3));
fr2=(Gamma[z-2]/(Gamma[z+1]))*(qr^(-3))*Sin[(z-2)*ArcTan[2*qr]]/((1+4*qr*qr)^((z-2)/2));
fr3=(Gamma[z-3]/(Gamma[z+1]))*(qr^(-4))*Cos[(z-3)*ArcTan[2*qr]]/((1+4*qr*qr)^((z-3)/2));
fr=(4/Pi)*(fr1-fr2-(9/8)*fr3);
Pqint=NIntegrate[fl*fr*Sin[theta], {theta, 0, Pi/2}]; *)
lim=1.5;
pl2=Plot[Log[10,Fq1], {lq , -1,lim}, PlotRange->{-6,1}, GridLines->{Automatic}, LabelStyle->Directive[Black,16],AxesLabel-
>{"log q","lg F^2(q)"}, AxesOrigin->{-1,-6},TicksStyle->Directive[Black,12],PlotStyle->{Black}] ;
(* pl1=Plot[Log[10,Pqc], {lq , -1,lim}, PlotRange{-6,1}, GridLines->{Automatic}, LabelStyle->Directive[Black,16],AxesLabel-
>{"log q","lg F^2(q)"}, AxesOrigin->{-1,-6},TicksStyle->Directive[Black,12],PlotStyle->{Blue}]; *)
Show[pl2]
lims=9.0;
(* pl4=DensityPlot[Log[10,Fq1s],{qx, -lims, lims}, {qy, -lims, lims}, PlotRange->{-8,0}, PlotPoints->50, PlotLegends->Automatic,
LabelStyleDirective[Black,12],AxesLabel->Automatic] *)
```

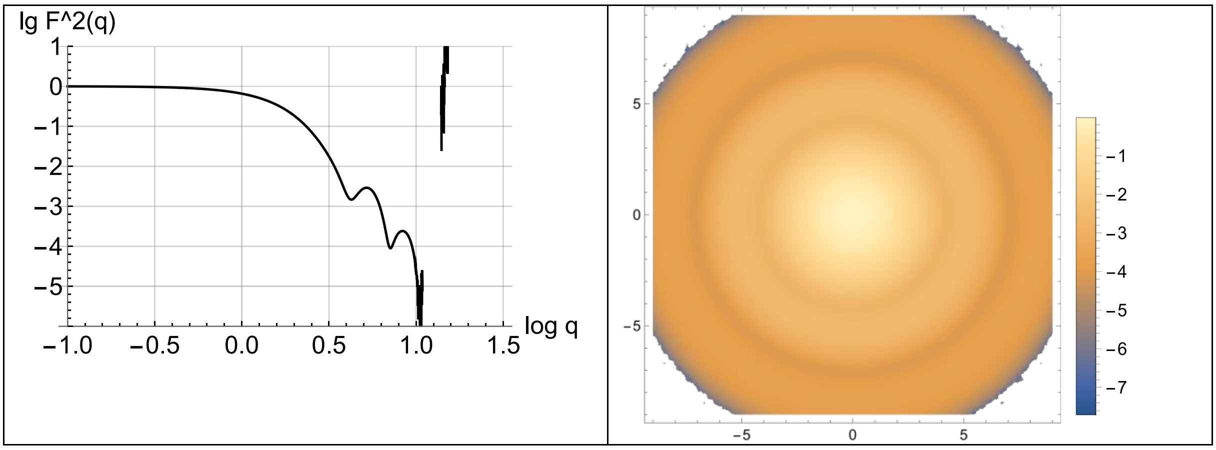

Fig. S20: Exact scattering amplitude of polydisperse isotropic cylinders with circular cross-section, cross-sectional part.

4.6.2 Large axial ratios

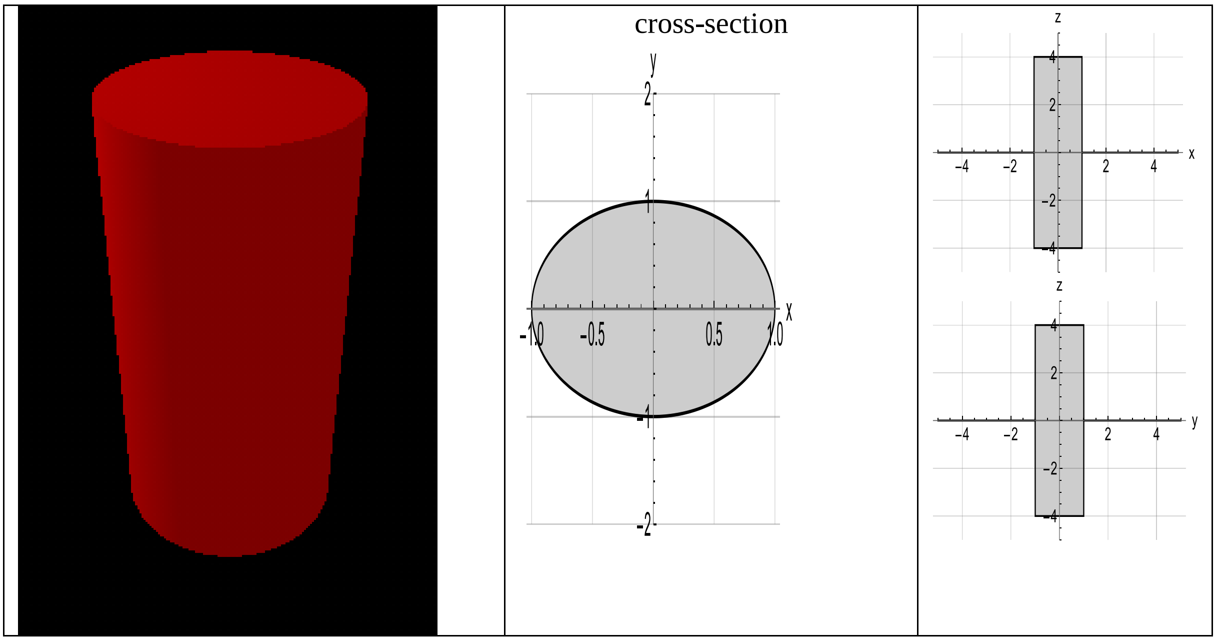

Fig. S21: Cylinder with cross-sections in the (*x*,*y*)-, (*x*,*z*)-, and (*y*,*z*)-planes.

4.6.2.1 Formfactor

For large axial ratios $L/R > 3$ a very good approximation is (S.4.6.2.1.1)

$$P(q) = \langle P_{\perp}(qR)\rangle \int_{0}^{\frac{\pi}{2}} \langle P_{\parallel}(qL\cos\theta)\rangle \sin\theta \, d\theta$$

where the brackets already indicate the average over the size distribution over the length L and the cross-sectional radius R.

Regime I. We can therefore employ Eq. (S.4.6.1.1.2) to average for the longitudinal part

$$P_{\parallel}(q) = \sum_{n=0}^{\infty} \frac{4^n}{(\mathrm{n}+1)} \frac{(z+s_L+1)_{2n}}{\left(\frac{3}{2}\right)_n n!} \left(-\frac{q^2L^2}{4(z+1)^2}\right)^n \int_{0}^{\frac{\pi}{2}} (\cos(\theta))^{2n} \sin\theta \, d\theta$$

The integral can be solved analytically to obtain

$$\int_{0}^{\frac{\pi}{2}} (\cos(\theta))^{2n} \sin\theta \, d\theta = \frac{1}{2n+1}$$

Therefore, we arrive at the series expansion (S.4.6.2.1.2)

$$P_{\|}(q) = \sum_{n=0}^{\infty} \frac{4^n}{(2n+1)(n+1)} \frac{(z_L + s_L + 1)_{2n}}{\left(\frac{3}{2}\right)_n n!} \left(-\frac{q^2L^2}{4(z+1)^2}\right)^n = \sum_{n=0}^{\infty} c_{n,\|}^{(1)} q^{2n}$$

$$c_{n,||}^{(1)} = \frac{4^n}{(2n+1)(n+1)} \frac{(z_L + s_L + 1)_{2n}}{\left(\frac{3}{2}\right)_n n!} \left(-\frac{L^2}{4(z+1)^2}\right)^n$$

with the q-independent coefficient $c_{n,||}^{(1)}$. For the cross-sectional part we have

$$\langle P_{\perp}(q)\rangle = \sum_{n=0}^{\infty} \frac{4^{n+1}\Gamma\left(n+\frac{3}{2}\right)}{\sqrt{\pi}\Gamma(n+3)} \frac{(z_R + s_R + 1)_{2n}}{(2)_n n!} \left(-\frac{q^2R^2}{4(z+1)^2}\right)^n = \sum_{n=0}^{\infty} c_{n,\perp}^{(2)} q^{2n}$$

$$c_{n,\perp}^{(2)} = \frac{4^{n+1}\Gamma\left(n+\frac{3}{2}\right)}{\sqrt{\pi}\Gamma(n+3)} \frac{(z_R + s_R + 1)_{2n}}{(2)_n n!} \left(-\frac{R^2}{4(z+1)^2}\right)^n$$

Cylinder, polydisperse, factorized, isotropic, P(q)

Relation to sine integral

We consider the monodisperse case

$$P_{\|}(q) = \sum_{n=0}^{\infty} \frac{4^n}{(n+1)} \frac{1}{(2n+1)} \frac{1}{\left(\frac{3}{2}\right)_n n!} \left(-\frac{q^2L^2}{16}\right)^n$$

and rewrite using $z\Gamma(z) = \Gamma(z+1)$ and the definition of the Pochhammer factorial

$$\frac{1}{(2n+1)} = \frac{1}{2\left(n+\frac{1}{2}\right)} = \frac{\Gamma\left(n+\frac{1}{2}\right)}{2\Gamma\left(n+\frac{3}{2}\right)} = \frac{\left(\frac{1}{2}\right)_n}{\left(\frac{3}{2}\right)_n}$$

and use further

$$\frac{1}{(2n+1)} = \frac{2(n+1)}{2n+1} - 1 = 2(n+1)\frac{\left(\frac{1}{2}\right)_n}{\left(\frac{3}{2}\right)_n} - 1$$

This is inserted into the series expansion and use Eq. (sin(x)/x)^2

$$P_{\|}(q) = \sum_{n=0}^{\infty} \frac{4^n}{(n+1)} \left(2(n+1)\frac{\left(\frac{1}{2}\right)_n}{\left(\frac{3}{2}\right)_n} - 1\right) \frac{1}{\left(\frac{3}{2}\right)_n n!} \left(-\frac{q^2L^2}{16}\right)^n$$

$$= \sum_{n=0}^{\infty} \frac{4^n}{(n+1)} 2(n+1) \frac{\left(\frac{1}{2}\right)_n}{\left(\frac{3}{2}\right)_n} \frac{1}{\left(\frac{3}{2}\right)_n n!} \left(-\frac{q^2L^2}{16}\right)^n - \sum_{n=0}^{\infty} \frac{4^n}{(n+1)} \frac{1}{\left(\frac{3}{2}\right)_n n!} \left(-\frac{q^2L^2}{16}\right)^n$$

$$= 2\sum_{n=0}^{\infty}\frac{\left(\frac{1}{2}\right)_n}{\left(\frac{3}{2}\right)_n\left(\frac{3}{2}\right)_n n!}\left(-\frac{q^2L^2}{4}\right)^n - \left(\frac{\sin\left(\frac{qL}{2}\right)}{\left(\frac{qL}{2}\right)}\right)^2$$

$$= 2\ {}_1F_2\left(\frac{1}{2};\frac{3}{2},\frac{3}{2};-\frac{q^2L^2}{4}\right) - \left(\frac{\sin\left(\frac{qL}{2}\right)}{\left(\frac{qL}{2}\right)}\right)^2$$

$$= 2\frac{qL}{qL}\ {}_1F_2\left(\frac{1}{2};\frac{3}{2},\frac{3}{2};-\frac{q^2L^2}{4}\right) - \left(\frac{\sin\left(\frac{qL}{2}\right)}{\left(\frac{qL}{2}\right)}\right)^2$$

$$= \frac{2}{qL}\mathrm{Si}(qL) - \left(\frac{\sin\left(\frac{qL}{2}\right)}{\left(\frac{qL}{2}\right)}\right)^2$$

with the sine integral

$$\mathrm{Si}(z) = \int_0^z \frac{\sin(t)}{t}dt = z\ {}_1F_2\left(\frac{1}{2};\frac{3}{2},\frac{3}{2};-\frac{z^2}{4}\right)$$

Regime II. The asymptote for $P_{\parallel}(q)$ is then given by the asymptote of (S.4.6.2.1.3)

$$P_{\parallel}(q) = \int_0^{\frac{\pi}{2}} \frac{\sin(qL\cos\theta)^2}{(qL\cos\theta)^2}\sin\theta\, d\theta = \frac{Si[2qL]}{qL} - \frac{(\sin(qL))^2}{(qL)^2}$$

We will show that the series expansion has a large convergence regime, such that it overlaps with the Porod asymptote in Regime II and therefore does not have to be calculated.

For the cross-sectional part we derive the expansion in terms of trigonometric functions as (S.4.6.2.1.4)

$$P_{\perp}(q) = \frac{4}{\pi}\left(\left(\frac{1}{qR}\right)^3 - \left(\frac{1}{qR}\right)^3\sin(2qR) - \frac{9}{8}\left(\frac{1}{qR}\right)^4\cos(2qR) + \left(\frac{9}{16}\right)^2\left(\frac{1}{qR}\right)^5 + \left(\frac{9}{16}\right)^2\left(\frac{1}{qR}\right)^5\sin(2qR)\right)$$

$$\langle (qR)^{-3}\rangle = \frac{\Gamma[z+s-2]}{\Gamma[z+s+1]}\left(\frac{qR}{z+1}\right)^{-3}$$

$$\langle (qR)^{-3}(\sin(2qR))\rangle = \frac{\Gamma[z+s-2]}{\Gamma[z+s+1]}\left(\frac{qR}{z+1}\right)^{-3}\frac{\sin\left[(z+s-2)\arctan\left(\frac{2qR}{z+1}\right)\right]}{\left(1+\left(\frac{2qR}{z+1}\right)^2\right)^{\frac{z+s-2}{2}}}$$

$$\langle (qR)^{-4}(\cos(2qR))\rangle = \frac{\Gamma[z+s-3]}{\Gamma[z+s+1]}\left(\frac{qR}{z+1}\right)^{-4}\frac{\cos\left[(z+s-3)\arctan\left(\frac{2qR}{z+1}\right)\right]}{\left(1+\left(\frac{2qR}{z+1}\right)^2\right)^{\frac{z+s-3}{2}}}$$

$$\langle (qR)^{-5}\rangle = \frac{\Gamma[z+s-4]}{\Gamma[z+s+1]}\left(\frac{x}{z+1}\right)^{-4}$$

$$\langle (qR)^{-5}(\sin(2qR))\rangle = \frac{\Gamma[z+s-4]}{\Gamma[z+s+1]}\left(\frac{qR}{z+1}\right)^{-5}\frac{\sin\left[(z+s-4)\arctan\left(\frac{2qR}{z+1}\right)\right]}{\left(1+\left(\frac{2qR}{z+1}\right)^2\right)^{\frac{z+s-4}{2}}}$$

Regime III. In the limit of high q we use the asymptote of the sine-integral Eq. (S.1.1.6) and obtain (S.4.6.2.1.5)

$$\lim_{q\to\infty} P_{\parallel}(q) = \lim_{q\to\infty}\frac{Si[2qL]}{qL} = \frac{\pi}{2qL}$$

It can be averaged over the size distribution to obtain

$$\lim_{q\to\infty} P(q) = \frac{\pi}{2}\frac{1}{\mathrm{z}}\left(\frac{z+1}{qL}\right)^1$$

For the cross-sectional part we use the leading term in Eq. (xx) to obtain

$$\lim_{q\to\infty} P_{\perp}(q) = \frac{4}{\pi}\left(\frac{1}{qR}\right)^3$$

It can be averaged over the size distribution to obtain (S.4.6.2.1.6)

$$\lim_{q\to\infty} P_{\perp}(q) = \frac{4}{\pi}\frac{1}{\mathrm{z(z-1)}}\left(\frac{z+1}{qR}\right)^3$$

In the following we demonstrate the good overlap which serves for a rapid calculation of the formfactor (include also integral representation for comparison; later needed to convince in the benchmark). Also include 2D-pattern.

Mathematica code implementation:

```
L=3;
R=1;
sigma=0.1;
z=(1-sigma*sigma)/(sigma*sigma);
nmax=100;
q=10^lq;
qs=Sqrt[qx*qx+qy*qy];
Pqpar=(Sqrt[Pi]/2)*Sum[(4^n)*Pochhammer[z+1,2*n]*((-
q*q*L*L/(4*(z+1)*(z+1)))^n)/((2*n+1)*(n+1)*Gamma[n+3/2]*(n!)), {n, 0, nmax}];
Pqcross=Sum[4*(n+1/2)*((2*n)!)*Pochhammer[z+1,2*n]*((-q*q*R*R/(4*(z+1)*(z+1)))^n)/(((n+2)!)*(n!)*((n+1)!)*(n!)), {n, 0,
nmax}];
Pqpars=(Sqrt[Pi]/2)*Sum[(4^n)*Pochhammer[z+1,2*n]*((-
qs*qs*L*L/(4*(z+1)*(z+1)))^n)/((2*n+1)*(n+1)*Gamma[n+3/2]*(n!)), {n, 0, nmax}];
```

```
Pqcrosss=Sum[4*(n+1/2)*((2*n)!)*Pochhammer[z+1,2*n]*((-qs*qs*R*R/(4*(z+1)*(z+1)))^n)/(((n+2)!)*(n!)*((n+1)!)*(n!)),
{n, 0, nmax}];
ql=q*L/(z+1);
qr=q*R/(z+1);
frr1=(Gamma[z-2]/(Gamma[z+1]))*(qr^(-3));
frr2=(Gamma[z-2]/(Gamma[z+1]))*(qr^(-3))*Sin[(z-2)*ArcTan[2*qr]]/((1+4*qr*qr)^((z-2)/2));
frr3=(Gamma[z-3]/(Gamma[z+1]))*(qr^(-4))*Cos[(z-3)*ArcTan[2*qr]]/((1+4*qr*qr)^((z-3)/2));
frr4=(Gamma[z-4]/(Gamma[z+1]))*(qr^(-5));
frr5=(Gamma[z-4]/(Gamma[z+1]))*(qr^(-5))*Sin[(z-4)*ArcTan[2*qr]]/((1+4*qr*qr)^((z-4)/2));
frasy=(4/Pi)*(frr1-frr2-(9/8)*frr3+(9/16)*(9/16)*frr4+(9/16)*(9/16)*frr5);
Pqparas=(Pi/(2*z))*(ql^(-1))-(Gamma[z-1]/(2*Gamma[z+1]))*(ql^(-2));
Pqcrossas=(4*Gamma[z-2]/(Pi*Gamma[z+1]))*(qr^(-3));
lim1=2;
lim2=2;
pl1=Plot[Log[10,Pqpar], {lq , -1,lim1}, PlotRange->{-2,1}, GridLines->{Automatic}, LabelStyle->Directive[Black,16],AxesLabel-
>{"log q","lg P(q)"}, AxesOrigin->{-1,-2},TicksStyle->Directive[Black,12],PlotStyle->{Black,Dashed}] ;
pl2=Plot[Log[10,Pqparas], {lq , -1,lim1}, PlotRange->{-2,1}, GridLines->{Automatic}, LabelStyle-
>Directive[Black,16],AxesLabel->{"log q","lg P(q)"}, AxesOrigin->{-1,-2},TicksStyle->Directive[Black,12],PlotStyle->{Red}] ;
(* pl1=Plot[Log[10,Pqc], {lq , -1,lim}, PlotRange□{-6,1}, GridLines->{Automatic}, LabelStyle->Directive[Black,16],AxesLabel-
>{"log q","lg P(q)"}, AxesOrigin->{-1,-6},TicksStyle->Directive[Black,12],PlotStyle->{Blue}]; *)
pl3=Plot[Log[10,Pqcross], {lq , -1,lim2}, PlotRange->{-6,1}, GridLines->{Automatic}, LabelStyle-
>Directive[Black,16],AxesLabel->{"log q","lg P(q)"}, AxesOrigin->{-1,-6},TicksStyle->Directive[Black,12],PlotStyle-
>{Black,Dashed}] ;
pl4=Plot[Log[10,Pqcrossas], {lq , -1,lim2}, PlotRange->{-6,1}, GridLines->{Automatic}, LabelStyle-
>Directive[Black,16],AxesLabel->{"log q","lg P(q)"}, AxesOrigin->{-1,-6},TicksStyle->Directive[Black,12],PlotStyle->{Red}] ;
pl4a=Plot[Log[10,frasy], {lq , -1,lim2}, PlotRange->{-6,1}, GridLines->{Automatic}, LabelStyle->Directive[Black,16],AxesLabel-
>{"log q","lg P(q)"}, AxesOrigin->{-1,-6},TicksStyle->Directive[Black,12],PlotStyle->{Blue}] ;
Show[pl1,pl2]
Show[pl3,pl4,pl4a]
lims=9;
pl5=DensityPlot[Log[10,Pqpars],{qx, -lims, lims}, {qy, -lims, lims}, PlotRange->{-8,0}, PlotPoints->50, PlotLegends-
>Automatic, LabelStyle->Directive[Black,12],AxesLabel->Automatic]
pl6=DensityPlot[Log[10,Pqcrosss],{qx, -lims, lims}, {qy, -lims, lims}, PlotRange->{-8,0}, PlotPoints->50, PlotLegends-
>Automatic, LabelStyle->Directive[Black,12],AxesLabel->Automatic]
```

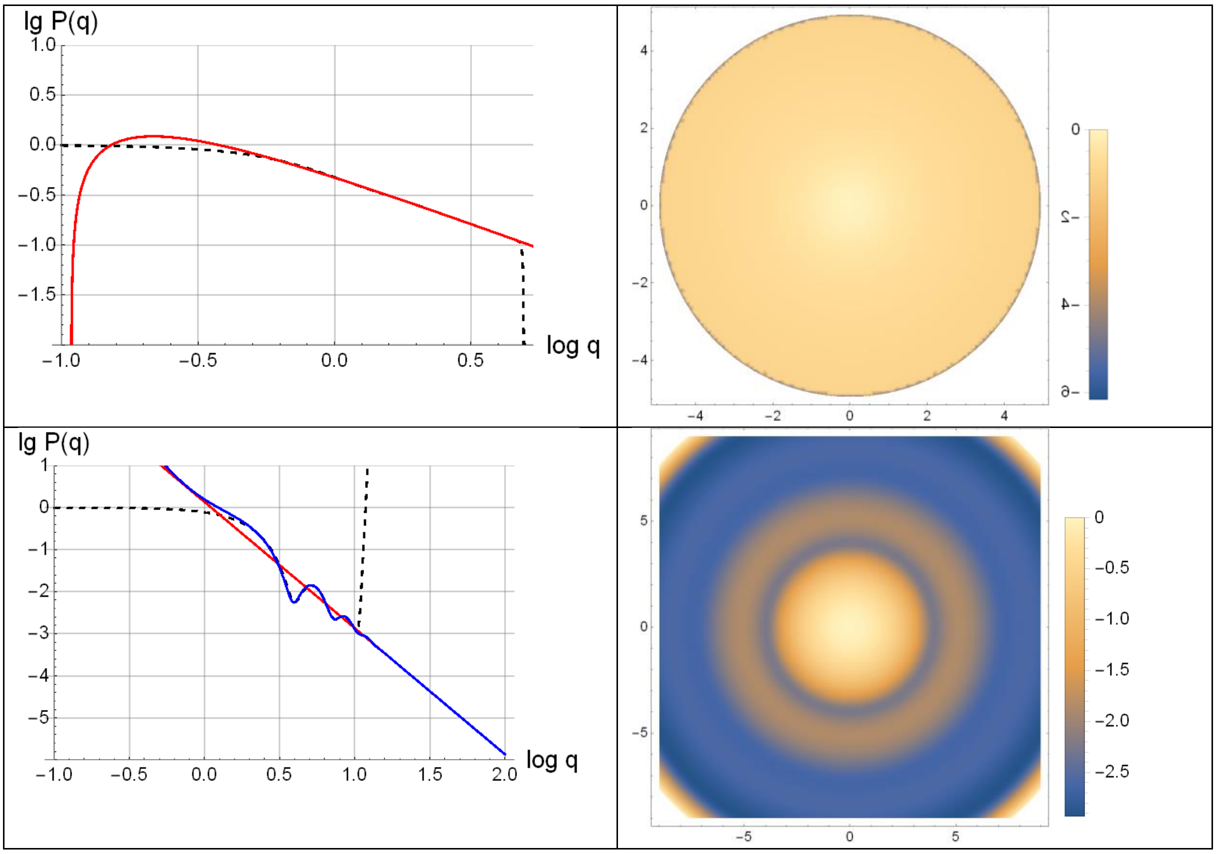

Fig. S22: Factorized formfactor of polydisperse isotropic cylinders with circular cross-section, axial and cross-sectional parts.

4.6.2.2 Scattering Amplitude F(q)

(S.4.6.2.2.1)

$$\langle F_{cyl}(q)\rangle_{L,R}^{2} = \left(\langle F^{(2)}(qR)\rangle_R\right)^2 \int_0^{\frac{\pi}{2}} \left(\langle F^{(1)}(q_z L)\rangle_L\right)^2 \sin\theta \, d\theta$$

with the longitudinal part

$$F^{(1)}(q_z L) = F_{\parallel}(q_z L) = \frac{\sin(qL\cos\theta)}{qL\cos\theta}$$

and the cross-sectional part

$$F^{(2)}(q_r R) = F_{\perp}(q_r R) = \frac{2J_1(qR\sin\theta)}{qR\sin\theta}$$

Regime I.

(S.4.6.2.2.2)

$$\left(\langle F^{(1)}(q_z L)\rangle_L\right)^2$$

$$= \sum_{n=0}^{\infty}\left(-\frac{(qL\cos\theta)^2}{4(z_L+1)^2}\right)^n \sum_{m=0}^{n}\frac{(z_L+s_L+1)_{2(n-m)}(z_L+s_L+1)_{2m}}{\left(\frac{3}{2}\right)_{n-m}(n-m)!\left(\frac{3}{2}\right)_n n!}\int_0^{\frac{\pi}{2}}(\cos(\theta))^{2n}\sin\theta\, d\theta$$

$$= \sum_{n=0}^{\infty}\left(-\frac{(qL)^2}{4(z_L+1)^2}\right)^n \sum_{m=0}^{n}\frac{(z_L+s_L+1)_{2(n-m)}(z_L+s_L+1)_{2m}}{\left(\frac{3}{2}\right)_{n-m}(n-m)!\left(\frac{3}{2}\right)_n n!}\frac{1}{2n+1}$$

$$= \sum_{n=0}^{\infty}\frac{1}{2n+1}\left(-\frac{(qL)^2}{4(z_L+1)^2}\right)^n \sum_{m=0}^{n}\frac{(z_L+s_L+1)_{2(n-m)}(z_L+s_L+1)_{2m}}{\left(\frac{3}{2}\right)_{n-m}(n-m)!\left(\frac{3}{2}\right)_n n!}$$

Cylinder, polydisperse, factorized, F(q), isotropic

Regime II.

(S.4.6.2.2.3)

$$F(q) = \langle\frac{2J_1(qR)}{qR}\rangle^2\int_0^{\frac{\pi}{2}}\langle\frac{\sin(qL\cos\theta)}{qL\cos\theta}\rangle^2\sin\theta\, d\theta$$

$$\langle(qR)^{-\frac{3}{2}}\sin(qR)\rangle = \frac{\Gamma[z-\frac{1}{2}]}{\Gamma[z+1]}\left(\frac{qR}{z+1}\right)^{-3/2}\frac{\sin\left[\left(z-\frac{1}{2}\right)\arctan\left(\frac{qR}{z+1}\right)\right]}{\left(1+\left(\frac{qR}{z+1}\right)^2\right)^{\frac{z-\frac{1}{2}}{2}}}$$

$$\langle(qR)^{-\frac{3}{2}}\cos(qR)\rangle = \frac{\Gamma[z-\frac{1}{2}]}{\Gamma[z+1]}\left(\frac{qR}{z+1}\right)^{-3/2}\frac{\cos\left[\left(z-\frac{1}{2}\right)\arctan\left(\frac{qR}{z+1}\right)\right]}{\left(1+\left(\frac{qR}{z+1}\right)^2\right)^{\frac{z-\frac{1}{2}}{2}}}$$

$$\langle(qR)^{-\frac{5}{2}}\sin(qR)\rangle = \frac{\Gamma[z-\frac{3}{2}]}{\Gamma[z+1]}\left(\frac{qR}{z+1}\right)^{-5/2}\frac{\sin\left[\left(z-\frac{3}{2}\right)\arctan\left(\frac{qR}{z+1}\right)\right]}{\left(1+\left(\frac{qR}{z+1}\right)^2\right)^{\frac{z-\frac{3}{2}}{2}}}$$

$$\langle(qR)^{-\frac{5}{2}}\cos(qR)\rangle = \frac{\Gamma[z-\frac{3}{2}]}{\Gamma[z+1]}\left(\frac{qR}{z+1}\right)^{-5/2}\frac{\cos\left[\left(z-\frac{3}{2}\right)\arctan\left(\frac{qR}{z+1}\right)\right]}{\left(1+\left(\frac{qR}{z+1}\right)^2\right)^{\frac{z-\frac{3}{2}}{2}}}$$

Mathematica code implementation:

```
L=3;
R=1;
sigma=0.1;
z=(1-sigma*sigma)/(sigma*sigma);
nmax=100;
q=10^lq;
qs=Sqrt[qx*qx+qy*qy];
fff=Table[Sum[Pochhammer[z+1,2*(n-m)]*Pochhammer[z+1,2*m]/(Pochhammer[2,n-m]*Pochhammer[2,m]*((n-m)!)*(m!)), {m, 0, n}],{n, 0, nmax}];
Fqcross=Sum[(((-q*q*R*R/(4*(z+1)*(z+1)))^n)*fff[[n+1]]), {n, 0, nmax}];
Fqcrosss=Sum[(((-qs*qs*R*R/(4*(z+1)*(z+1)))^n)*fff[[n+1]]), {n, 0, nmax}];
qr=q*R/(z+1);
fcrossa=(Gamma[z-1/2]/Gamma[z+1])*(qr^(-(3/2)))*Sin[(z-1/2)*ArcTan[qr]]/((1+qr*qr)^((z-1/2)/2));
fcrossb=(Gamma[z-1/2]/Gamma[z+1])*(qr^(-(3/2)))*Cos[(z-1/2)*ArcTan[qr]]/((1+qr*qr)^((z-1/2)/2));
fcrossc=(Gamma[z-3/2]/Gamma[z+1])*(qr^(-(5/2)))*Sin[(z-3/2)*ArcTan[qr]]/((1+qr*qr)^((z-3/2)/2));
fcrossd=(Gamma[z-3/2]/Gamma[z+1])*(qr^(-(5/2)))*Cos[(z-3/2)*ArcTan[qr]]/((1+qr*qr)^((z-3/2)/2));
Fqcrossas=((2/Sqrt[Pi])*(fcrossa-fcrossb+(9/16)*(fcrossc+fcrossd)))^2;
lim2=2;
pl3=Plot[Log[10,Fqcross], {lq , -1,lim2}, PlotRange->{-6,1}, GridLines->{Automatic}, LabelStyle->Directive[Black,16],AxesLabel->{"log q","lg F^2(q)"}, AxesOrigin->{-1,-6},TicksStyle->Directive[Black,12],PlotStyle->{Black,Dashed}] ;
pl4=Plot[Log[10,Fqcrossas], {lq , -1,lim2}, PlotRange->{-6,1}, GridLines->{Automatic}, LabelStyle->Directive[Black,16],AxesLabel->{"log q","lg F^2(q)"}, AxesOrigin->{-1,-6},TicksStyle->Directive[Black,12],PlotStyle->{Red}] ;
Show[pl3,pl4]
lims=15;
pl5=DensityPlot[Log[10,Fqcrosss],{qx, -lims, lims}, {qy, -lims, lims}, PlotRange->{-8,0}, PlotPoints->50, PlotLegends->Automatic, LabelStyle->Directive[Black,12],AxesLabel->Automatic]
```

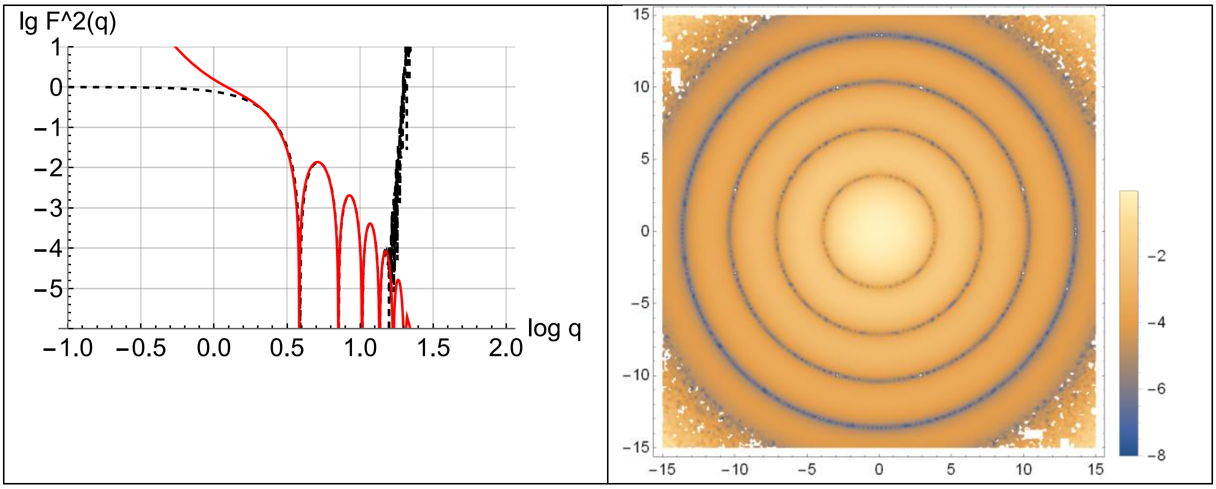

Fig. S23: Factorized scattering amplitude of polydisperse isotropic cylinders with circular cross-section, cross-sectional part.

4.6.4 Homogeneous cylinder with elliptical cross-section

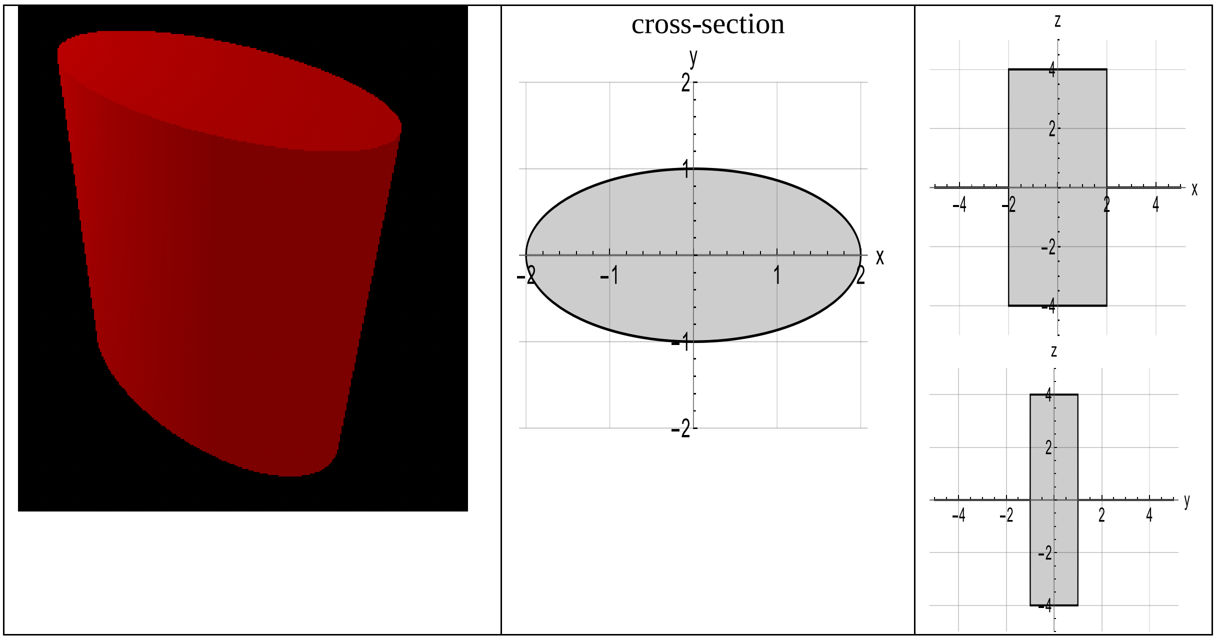

Fig. S24: Elliptical cylinder with cross-sections in the (*x*,*y*)-, (*x*,*z*)-, and (*y*,*z*)-planes.

4.6.4.1 Formfactor P(q)

(S.4.6.4.1.1)

$$P(q) = \int_0^{\frac{\pi}{2}} \langle\frac{\sin(qL\cos\theta)}{qL\cos\theta}\rangle^2 \langle\frac{2J_1(qR(\phi)\sin\theta)}{qR(\phi)\sin\theta}\rangle^2 \sin\theta \, d\theta$$

$$R(\phi) = \sqrt{(a\sin\phi)^2 + (c\cos\phi)^2}$$

Therefore the obtained expressions for the cross-sectional parts need to be integrated over ϕ

Regime I.

(S.4.6.4.1.2)

$$\langle P_\perp(q)\rangle = \frac{2}{\pi}\int_0^{\frac{\pi}{2}} \sum_{n=0}^{\infty} \frac{4\left(n+\frac{1}{2}\right)(2\mathrm{n})!}{(\mathrm{n}+2)!\,n!} \frac{(z+1)_{2n}}{(n+1)!\,n!} \left(-\frac{q^2R(\phi)^2}{4}\right)^n d\phi$$

$$= \sum_{n=0}^{\infty} \frac{4\left(n+\frac{1}{2}\right)(2\mathrm{n})!}{(\mathrm{n}+2)!\,n!} \frac{(z+1)_{2n}}{(n+1)!\,n!} \left(-\frac{q^2}{4}\right)^n \frac{2}{\pi}\int_0^{\frac{\pi}{2}}\int_0^{\pi/2} ((a\sin\phi)^2 + (c\cos\phi)^2)^n \, d\phi$$

$$= \sum_{n=0}^{\infty} \frac{4\left(n+\frac{1}{2}\right)(2\mathrm{n})!}{(\mathrm{n}+2)!\,n!} \frac{(z+1)_{2n}}{(n+1)!\,n!} \left(-\frac{q^2}{4}\right)^n \frac{a^{2n}}{\pi} \sum_{m=0}^{n} \frac{\Gamma\left(n-m+\frac{1}{2}\right)\Gamma\left(m+\frac{1}{2}\right)}{(n-m)!\,m!} \left(\frac{c}{a}\right)^{2(n-m)}$$

$$= \frac{1}{\pi} \sum_{n=0}^{\infty} \frac{4\left(n+\frac{1}{2}\right)(2\mathrm{n})!}{(\mathrm{n}+2)!\,n!} \frac{(z+1)_{2n}}{(n+1)!\,n!} \left(-\frac{q^2 a^2}{4}\right)^n \sum_{m=0}^{n} \frac{\Gamma\left(n-m+\frac{1}{2}\right)\Gamma\left(m+\frac{1}{2}\right)}{(n-m)!\,m!} \left(\frac{c}{a}\right)^{2(n-m)}$$

Cylinder, polydisperse, factorized, ellipsoidal, isotropic, P(q)

Regime II. From the square of the scattering amplitude we get (S.4.6.4.1.3)

$$P_{\perp}(q) = \frac{4}{\pi}\left(\left(\frac{1}{qR}\right)^3 - \left(\frac{1}{qR}\right)^3 \sin(2qR) - \frac{9}{8}\left(\frac{1}{qR}\right)^4 \cos(2qR) + \left(\frac{9}{16}\right)^2 \left(\frac{1}{qR}\right)^5 + \left(\frac{9}{16}\right)^2 \left(\frac{1}{qR}\right)^5 \sin(2qR)\right)$$

The corresponding terms for the size-averages consist of non-oscillating and oscillating parts

$$\langle (qR(\phi))^{-3} \rangle = \frac{\Gamma[z-2]}{\Gamma[z+1]} \left(\frac{qR(\phi)}{z+1}\right)^{-3} = \frac{\Gamma[z-2]}{\Gamma[z+1]} \left(\frac{q}{z+1}\right)^{-3} \frac{2}{\pi} \int_0^{\pi/2} \frac{d\phi}{((a\sin\phi)^2 + (c\cos\phi)^2)^{3/2}}$$

$$\langle (qR)^{-3} (\sin(2qR)) \rangle = \frac{\Gamma[z-2]}{\Gamma[z+1]} \left(\frac{qR}{z+1}\right)^{-3} \frac{\sin\left[(z-2)\arctan\left(\frac{2qR}{z+1}\right)\right]}{\left(1+\left(\frac{2qR}{z+1}\right)^2\right)^{\frac{z-2}{2}}}$$

$$\langle (qR)^{-4} (\cos(2qR)) \rangle = \frac{\Gamma[z-3]}{\Gamma[z+1]} \left(\frac{qR}{z+1}\right)^{-4} \frac{\cos\left[(z-3)\arctan\left(\frac{2qR}{z+1}\right)\right]}{\left(1+\left(\frac{2qR}{z+1}\right)^2\right)^{\frac{z-3}{2}}}$$

$$\langle (qR)^{-5} \rangle = \frac{\Gamma[z-4]}{\Gamma[z+1]} \left(\frac{x}{z+1}\right)^{-4}$$

$$\langle (qR)^{-5} (\sin(2qR)) \rangle = \frac{\Gamma[z-4]}{\Gamma[z+1]} \left(\frac{qR}{z+1}\right)^{-5} \frac{\sin\left[(z-4)\arctan\left(\frac{2qR}{z+1}\right)\right]}{\left(1+\left(\frac{2qR}{z+1}\right)^2\right)^{\frac{z-4}{2}}}$$

Regime III. The asymptotic expansion linear in q^3 is (S.4.6.4.1.4)

$$P_{\perp}(q) \to \frac{4}{\pi}\left(\frac{1}{qR}\right)^3$$

$$P_{\perp}(q) \to \frac{4}{\pi} \frac{1}{\mathrm{z}(\mathrm{z}-1)} \left(\frac{z+1}{qR}\right)^3$$

Mathematica code implementation:

```
L=3;
a=1;
c=1.5;
Rell=Sqrt[((a*Sin[phi])^2)+((c*Cos[phi])^2)];
sigma=0.1;
z=(1-sigma*sigma)/(sigma*sigma);
nmax=80;
q=10^lq;
qs=Sqrt[qx*qx+qy*qy];
eee=Table[(1/Pi)*Sum[Gamma[n-m+1/2]*Gamma[m+1/2]*((c/a)^(2*(n-m)))/(((n-m)!)*(m!)), {m, 0, n}],{n, 0, nmax}];
Pqpar=(Sqrt[Pi]/2)*Sum[(4^n)*Pochhammer[z+1,2*n]*((-
q*q*L*L/(4*(z+1)*(z+1)))^n)/((2*n+1)*(n+1)*Gamma[n+3/2]*(n!)), {n, 0, nmax}];
Pqpars=(Sqrt[Pi]/2)*Sum[(4^n)*Pochhammer[z+1,2*n]*((-
qs*qs*L*L/(4*(z+1)*(z+1)))^n)/((2*n+1)*(n+1)*Gamma[n+3/2]*(n!)), {n, 0, nmax}];
Pqcrossell=Sum[4*(n+1/2)*((2*n)!)*Pochhammer[z+1,2*n]*((-
q*q*a*a/(4*(z+1)*(z+1)))^n)*eee[[n+1]]/(((n+2)!)*(n!)*((n+1)!)*(n!)), {n, 0, nmax}];
Pqcrossells=Sum[4*(n+1/2)*((2*n)!)*Pochhammer[z+1,2*n]*((-
qs*qs*a*a/(4*(z+1)*(z+1)))^n)*eee[[n+1]]/(((n+2)!)*(n!)*((n+1)!)*(n!)), {n, 0, nmax}];
ql=q*L/(z+1);
qe=q*Rell/(z+1);
asy=NIntegrate[(Rell)^(-3),{phi, 0, Pi/2}]/(Pi/2);
frr1e=(Gamma[z-2]/Gamma[z+1])*NIntegrate[qe^(-3),{phi, 0, Pi/2}]/(Pi/2);
frr2e=(Gamma[z-2]/Gamma[z+1])*NIntegrate[(qe^(-3))*Sin[(z-2)*ArcTan[2*qe]]/((1+4*qe*qe)^((z-2)/2)), {phi, 0,
Pi/2}]/(Pi/2);
Pqcrossasell=(4/Pi)*(frr1e-frr2e);
Pqparas=(Pi/(2*z))*(ql^(-1))-(Gamma[z-1]/(2*Gamma[z+1]))*(ql^(-2));
Pqcrossas=(4*Gamma[z-2]/(Pi*Gamma[z+1]))*asy*((z+1)/q)^3;
lim1=2;
lim2=2;
pl1=Plot[Log[10,Pqpar], {lq , -1,lim1}, PlotRange->{-2,1}, GridLines->{Automatic}, LabelStyle->Directive[Black,16],AxesLabel-
>{"log q","lg P(q)"}, AxesOrigin->{-1,-2},TicksStyle->Directive[Black,12],PlotStyle->{Black,Dashed}] ;
pl2=Plot[Log[10,Pqparas], {lq , -1,lim1}, PlotRange->{-2,1}, GridLines->{Automatic}, LabelStyle-
>Directive[Black,16],AxesLabel->{"log q","lg P(q)"}, AxesOrigin->{-1,-2},TicksStyle->Directive[Black,12],PlotStyle->{Red}] ;
(* pl1=Plot[Log[10,Pqc], {lq , -1,lim}, PlotRange�{-6,1}, GridLines->{Automatic}, LabelStyle->Directive[Black,16],AxesLabel-
>{"log q","lg P(q)"}, AxesOrigin->{-1,-6},TicksStyle->Directive[Black,12],PlotStyle->{Blue}]; *)
pl3=Plot[Log[10,Pqcrossell], {lq , -1,lim2}, PlotRange->{-6,1}, GridLines->{Automatic}, LabelStyle-
>Directive[Black,16],AxesLabel->{"log q","lg P(q)"}, AxesOrigin->{-1,-6},TicksStyle->Directive[Black,12],PlotStyle-
>{Black,Dashed}] ;
pl4=Plot[Log[10,Pqcrossas], {lq , -1,lim2}, PlotRange->{-6,1}, GridLines->{Automatic}, LabelStyle-
>Directive[Black,16],AxesLabel->{"log q","lg P(q)"}, AxesOrigin->{-1,-6},TicksStyle->Directive[Black,12],PlotStyle->{Red}] ;
pl4a=Plot[Log[10,Pqcrossasell], {lq , -1,lim2}, PlotRange->{-6,1}, GridLines->{Automatic}, LabelStyle-
>Directive[Black,16],AxesLabel->{"log q","lg P(q)"}, AxesOrigin->{-1,-6},TicksStyle->Directive[Black,12],PlotStyle->{Blue}] ;
Show[pl1,pl2]
Show[pl3,pl4,pl4a]
lims=9;
pl5=DensityPlot[Log[10,Pqpars],{qx, -lims, lims}, {qy, -lims, lims}, PlotRange->{-8,0}, PlotPoints->50, PlotLegends-
>Automatic, LabelStyle->Directive[Black,12],AxesLabel->Automatic]
pl6=DensityPlot[Log[10,Pqcrossells],{qx, -lims, lims}, {qy, -lims, lims}, PlotRange->{-8,0}, PlotPoints->50, PlotLegends-
>Automatic, LabelStyle->Directive[Black,12],AxesLabel->Automatic]
```

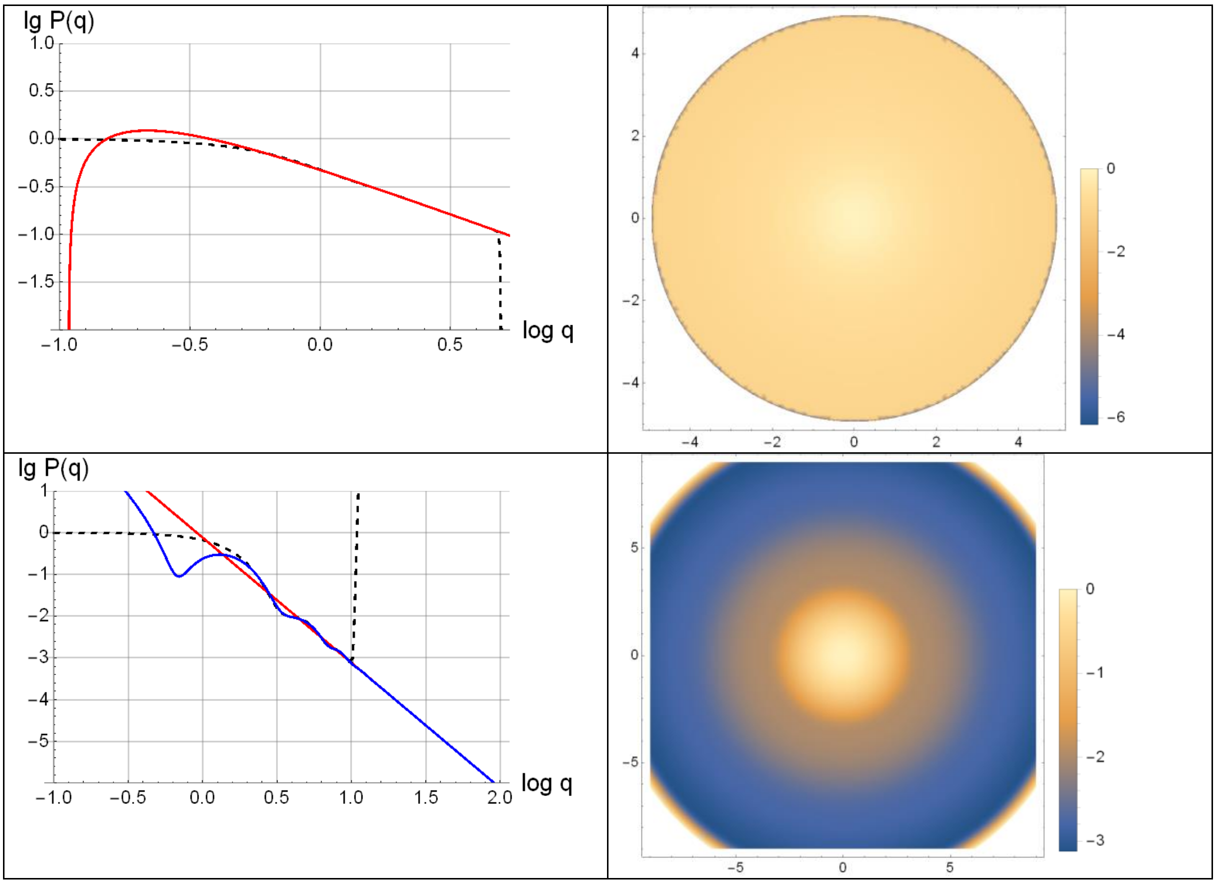

Fig. S25: Factorized formfactor of polydisperse isotropic cylinders with elliptical cross-section, axial and cross-sectional parts.

4.6.4.2 Scattering Amplitude F(q)

The series expansions in the polydisperse case are for the cross-sectional part is given by

$$\langle F_{\perp}(q)\rangle^2 = \frac{2}{\pi}\int_0^{\frac{\pi}{2}} \sum_{n=0}^{\infty}\left(-\frac{q^2R(\phi)^2}{4(z+1)^2}\right)^n \sum_{m=0}^{n}\frac{(z+1)_{2(n-m)}(z+1)_{2m}}{(2)_{n-m}(2)_m(n-m)!\,m!}\,d\phi$$

$$= \sum_{n=0}^{\infty}\left(-\frac{q^2}{4(z+1)^2}\right)^n \sum_{m=0}^{n}\frac{(z+1)_{2(n-m)}(z+1)_{2m}}{(2)_{n-m}(2)_m(n-m)!\,m!}\frac{2}{\pi}\int_0^{\pi/2} R(\phi)^2\,d\phi$$

$$= \sum_{n=0}^{\infty}\left(-\frac{q^2}{4(z+1)^2}\right)^n c_n\frac{2}{\pi}\int_0^{\pi/2}((a\sin\phi)^2+(c\cos\phi)^2)^n\,d\phi$$

$$\frac{2}{\pi}\int_0^{\frac{\pi}{2}}((a\sin\phi)^2+(c\cos\phi)^2)^n d\phi = \frac{2}{\pi}\sum_{m=0}^{n}\binom{n}{m}a^{2m}(c^2)^{n-m}\int_0^{\frac{\pi}{2}}(\sin\phi)^{2m}(\cos\phi)^{2(n-m)}d\phi$$

$$= \frac{2}{\pi}\sum_{m=0}^{n}\binom{n}{m}a^{2m}(c^2)^{n-m}\frac{\Gamma\left(n-m+\frac{1}{2}\right)\Gamma\left(m+\frac{1}{2}\right)}{2\Gamma(n+1)} = \frac{2}{\pi}\sum_{m=0}^{n}\frac{n!}{(n-m)!\,m!}a^{2m}(c^2)^{n-m}\frac{\Gamma\left(n-m+\frac{1}{2}\right)\Gamma\left(m+\frac{1}{2}\right)}{2\mathrm{n}!}$$

$$= \frac{1}{\pi}\sum_{m=0}^{n}\frac{a^{2m}(c^2)^{n-m}}{(n-m)!\,m!}\Gamma\left(n-m+\frac{1}{2}\right)\Gamma\left(m+\frac{1}{2}\right)$$

$$= \frac{a^{2n}}{\pi}\sum_{m=0}^{n}\frac{\Gamma\left(n-m+\frac{1}{2}\right)\Gamma\left(m+\frac{1}{2}\right)}{(n-m)!\,m!}\left(\frac{c}{a}\right)^{2(n-m)}$$

(S.4.6.4.2.1)

$$\langle F_\perp(q)\rangle^2 = \sum_{n=0}^{\infty}\left(-\frac{q^2}{4(z+1)^2}\right)^n c_n\frac{a^{2n}}{\pi}\sum_{m=0}^{n}\frac{\Gamma\left(n-m+\frac{1}{2}\right)\Gamma\left(m+\frac{1}{2}\right)}{(n-m)!\,m!}\left(\frac{c}{a}\right)^{2(n-m)}$$

$$= \frac{1}{\pi}\sum_{n=0}^{\infty}\left(-\frac{q^2a^2}{4(z+1)^2}\right)^n c_n\sum_{m=0}^{n}\frac{\Gamma\left(n-m+\frac{1}{2}\right)\Gamma\left(m+\frac{1}{2}\right)}{(n-m)!\,m!}\left(\frac{c}{a}\right)^{2(n-m)}$$

Cylinder, polydisperse, factorized, ellipsoidal, isotropic, F(q)

Regime II. For the cross-sectional asymptotic terms we also need to integrate over the orientational distribution. (S.4.6.4.2.2)

$$F_\perp(q) = \frac{2}{(\pi)^{\frac{1}{2}}}\left(\frac{1}{qR}\right)^{\frac{3}{2}}\left((\sin(qR)-\cos(qR))+\frac{9}{16(qR)}(\sin(qR)+\cos(qR))\right)$$

The corresponding four terms for the size averages are

$$\langle (qR)^{-\frac{3}{2}}\sin(qR)\rangle = \frac{\Gamma[z-\frac{1}{2}]}{\Gamma[z+1]}\left(\frac{qR}{z+1}\right)^{-3/2}\frac{\sin\left[\left(z-\frac{1}{2}\right)\arctan\left(\frac{qR}{z+1}\right)\right]}{\left(1+\left(\frac{qR}{z+1}\right)^2\right)^{\frac{z-\frac{1}{2}}{2}}}$$

$$\langle (qR)^{-\frac{3}{2}}\cos(qR)\rangle = \frac{\Gamma[z-\frac{1}{2}]}{\Gamma[z+1]}\left(\frac{qR}{z+1}\right)^{-3/2}\frac{\cos\left[\left(z-\frac{1}{2}\right)\arctan\left(\frac{qR}{z+1}\right)\right]}{\left(1+\left(\frac{qR}{z+1}\right)^2\right)^{\frac{z-\frac{1}{2}}{2}}}$$

$$\langle (qR)^{-\frac{5}{2}}\sin(qR)\rangle = \frac{\Gamma[z-\frac{3}{2}]}{\Gamma[z+1]}\left(\frac{qR}{z+1}\right)^{-5/2}\frac{\sin\left[\left(z-\frac{3}{2}\right)\arctan\left(\frac{qR}{z+1}\right)\right]}{\left(1+\left(\frac{qR}{z+1}\right)^2\right)^{\frac{z-\frac{3}{2}}{2}}}$$

$$\langle (qR)^{-\frac{5}{2}} \cos(qR) \rangle = \frac{\Gamma[z-\frac{3}{2}]}{\Gamma[z+1]} \left(\frac{qR}{z+1}\right)^{-5/2} \frac{\cos\left[\left(z-\frac{3}{2}\right)\arctan\left(\frac{qR}{z+1}\right)\right]}{\left(1+\left(\frac{qR}{z+1}\right)^2\right)^{\frac{z-\frac{3}{2}}{2}}}$$

Mathematica code implementation:

```
L=3;
a=1;
c=1.5;
Rell=Sqrt[((a*Sin[phi])^2)+((c*Cos[phi])^2)];
sigma=0.1;
z=(1-sigma*sigma)/(sigma*sigma);
nmax=80;
q=10^lq;
qs=Sqrt[qx*qx+qy*qy];
fff=Table[Sum[Pochhammer[z+1,2*(n-m)]*Pochhammer[z+1,2*m]/(Pochhammer[2,n-m]*Pochhammer[2,m]*((n-m)!)*(m!)), {m, 0, n}],{n, 0, nmax}];
eee=Table[(1/Pi)*Sum[Gamma[n-m+1/2]*Gamma[m+1/2]*((c/a)^(2*(n-m)))/(((n-m)!)*(m!)), {m, 0, n}],{n, 0, nmax}];
Fqcrosselli=Sum[(((-q*q*R*R/(4*(z+1)*(z+1)))^n)*eee[[n+1]]*fff[[n+1]]), {n, 0, nmax}];
Fqcrossellis=Sum[(((-qs*qs*R*R/(4*(z+1)*(z+1)))^n)*eee[[n+1]]*fff[[n+1]]), {n, 0, nmax}];
qe=q*Rell/(z+1);
fcrossa=(Gamma[z-1/2]/Gamma[z+1])*(qe^(-(3/2)))*Sin[(z-1/2)*ArcTan[qe]]/((1+qe*qe)^((z-1/2)/2));
fcrossb=(Gamma[z-1/2]/Gamma[z+1])*(qe^(-(3/2)))*Cos[(z-1/2)*ArcTan[qe]]/((1+qe*qe)^((z-1/2)/2));
fcrossc=(Gamma[z-3/2]/Gamma[z+1])*(qe^(-(5/2)))*Sin[(z-3/2)*ArcTan[qe]]/((1+qe*qe)^((z-3/2)/2));
fcrossd=(Gamma[z-3/2]/Gamma[z+1])*(qe^(-(5/2)))*Cos[(z-3/2)*ArcTan[qe]]/((1+qe*qe)^((z-3/2)/2));
Fqcrossase=(2/Sqrt[Pi])*NIntegrate[(fcrossa-fcrossb+(9/16)*(fcrossc+fcrossd))^2, {phi, 0, Pi/2}]/(Pi/2);
lim2=2;
pl3=Plot[Log[10,Fqcrosselli], {lq , -1,lim2}, PlotRange->{-6,1}, GridLines->{Automatic}, LabelStyle->Directive[Black,16],AxesLabel->{"log q","lg F^2(q)"}, AxesOrigin->{-1,-6},TicksStyle->Directive[Black,12],PlotStyle->{Black,Dashed}] ;
pl4=Plot[Log[10,Fqcrossase], {lq , -1,lim2}, PlotRange->{-6,1}, GridLines->{Automatic}, LabelStyle->Directive[Black,16],AxesLabel->{"log q","lg F^2(q)"}, AxesOrigin->{-1,-6},TicksStyle->Directive[Black,12],PlotStyle->{Red}] ;
Show[pl3,pl4]
lims=12;
pl5=DensityPlot[Log[10,Fqcrossellis],{qx, -lims, lims}, {qy, -lims, lims}, PlotRange->{-8,0}, PlotPoints->50, PlotLegends->Automatic, LabelStyle->Directive[Black,12],AxesLabel->Automatic]
```

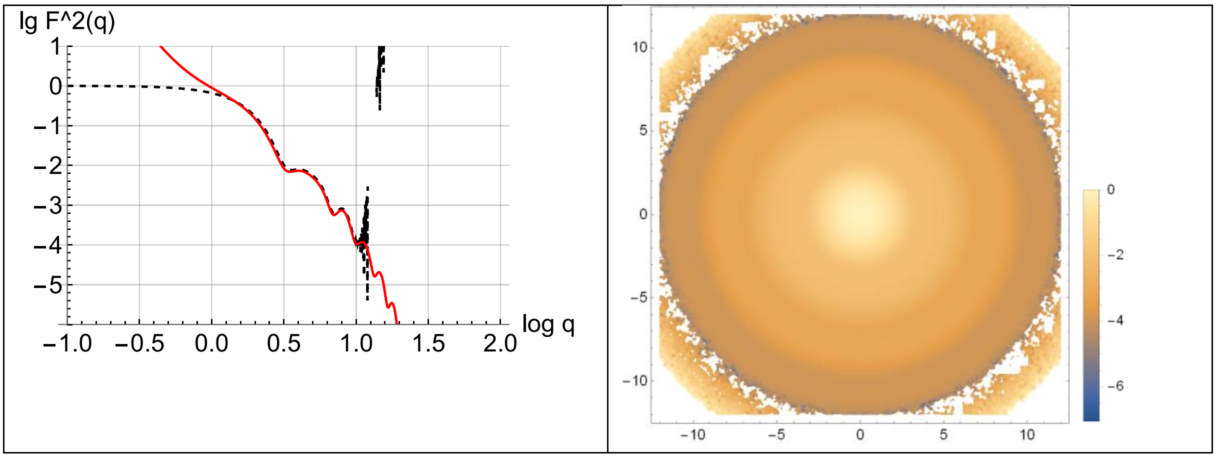

Fig. S26: Factorized scattering amplitude of polydisperse isotropic cylinders with elliptical cross-section, cross-sectional part.

4.7 Homogeneous Disk

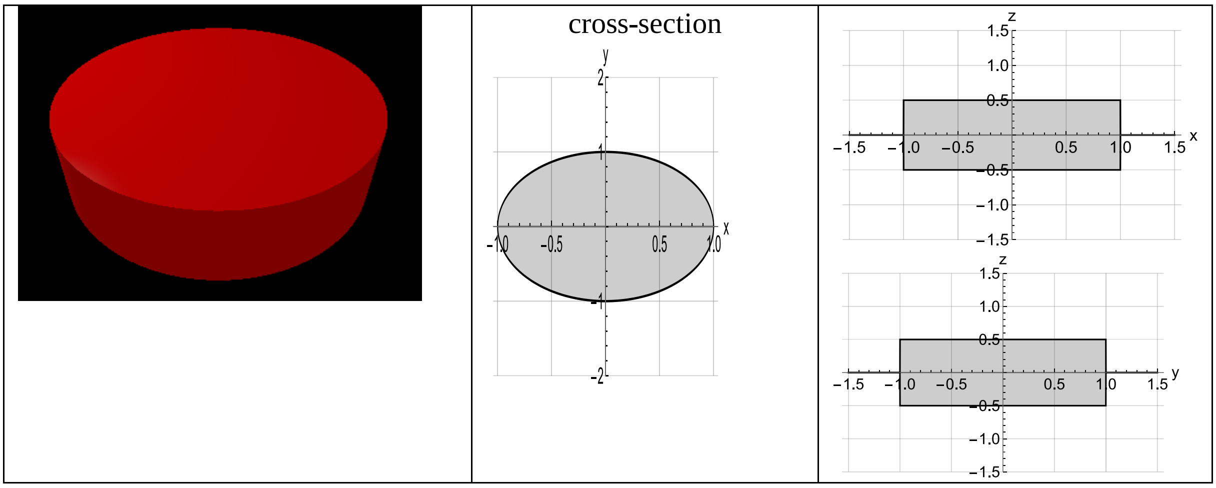

Fig. S27: Disk with cross-sections in the (*x*,*y*)-, (*x*,*z*)-, and (*y*,*z*)-planes.

4.7.1 Formfactor P(q)

For disks with axial Ratios L/R<3 the scattering amplitude and the formfactor are given by (S.4.7.1.1)

$$P(q) = \langle\frac{\sin(qL)^2}{(qL)^2}\rangle \int_0^{\frac{\pi}{2}} \langle\frac{4(J_1(qR\sin\theta)^2}{q^2(R\sin\theta)^2}\rangle \sin\theta\, d\theta$$

Regime I. For disks we have the well-known equations (S.4.7.1.2)

$$P_\perp(q) = \sum_{n=0}^{\infty} \frac{4^{n+1}\Gamma\left(n+\frac{3}{2}\right)}{\sqrt{\pi}\Gamma(n+3)} \frac{(z+1)_{2n}}{(2)_n n!} \left(-\frac{q^2R^2}{4(z+1)^2}\right)^n \int_0^{\frac{\pi}{2}} (\sin(\theta))^{2n} \sin\theta\, d\theta$$

$$\int_0^{\frac{\pi}{2}} (\sin(\theta))^{2n} \sin\theta\, d\theta = \frac{\sqrt{\pi}\Gamma(n+1)}{2\Gamma\left(n+\frac{3}{2}\right)}$$

$$P(q) = \sum_{n=0}^{\infty} \frac{2\cdot 4^n (z+1)_{2n}}{(2)_n (n+2)!} \left(-\frac{q^2R^2}{4(z+1)^2}\right)^n$$

Disks, polydisperse, factorized, isotropic, P(q)

$$P_{\|}(q) = \sum_{n=0}^{\infty} \frac{4^n}{(\mathrm{n}+1)} \frac{(z+1)_{2n}}{\left(\frac{3}{2}\right)_n n!} \left(-\frac{q^2 L^2}{4}\right)^n$$

$$= \frac{\sqrt{\pi}}{2} \sum_{n=0}^{\infty} \frac{4^n}{(\mathrm{n}+1)} \frac{(z+1)_{2n}}{\Gamma\left(n+\frac{3}{2}\right) n!} \left(-\frac{q^2 L^2}{4}\right)^n$$

$$= \frac{\sqrt{\pi}}{2} \sum_{n=0}^{\infty} \frac{4^n (z+1)_{2n}}{\Gamma\left(n+\frac{3}{2}\right)(n+1)!} \left(-\frac{q^2 L^2}{4}\right)^n$$

Relation to Bessel function

We consider the monodisperse case

$$P(q) = \sum_{n=0}^{\infty} \frac{2 \cdot 4^n}{(n+2)(n+1)(2)_n n!} \left(-\frac{q^2 R^2}{4}\right)^n$$

$$= 2 \sum_{n=0}^{\infty} \frac{\Gamma(2)}{(n+2)(n+1)\Gamma(n+2) n!} (-q^2 R^2)^n = 2 \sum_{n=0}^{\infty} \frac{\Gamma(2)}{\Gamma([n+1]+2)[n+1]!} (-q^2 R^2)^n$$

$$= 2 \frac{-q^2 R^2}{-q^2 R^2} \sum_{n=0}^{\infty} \frac{\Gamma(2)}{\Gamma([n+1]+2)[n+1]!} (-q^2 R^2)^n$$

$$= \frac{-2}{q^2 R^2} \sum_{n=0}^{\infty} \frac{\Gamma(2)}{\Gamma([n+1]+2)[n+1]!} (-q^2 R^2)^{n+2}$$

$$= \frac{-2}{q^2 R^2} \left(-1+1+\sum_{n=0}^{\infty} \frac{\Gamma(2)}{\Gamma([n+1]+2)[n+1]!} (-q^2 R^2)^{n+2}\right)$$

$$= -\frac{-2}{q^2 R^2} \left(-1+\sum_{m=0}^{\infty} \frac{\Gamma(2)}{\Gamma(m+2) m!} (-q^2 R^2)^m\right)$$

$$= \frac{2}{q^2 R^2} \left(1 - {}_0F_1(2; -q^2 R^2)\right) = \frac{2}{q^2 R^2} \left(1 - \frac{J_1(2qR)}{qR}\right)$$

Regime II. In terms of trigonometric functions the cross-sectional part is given by (S.4.7.1.2)

$$P_{\|}(q) = \int_0^{\frac{\pi}{2}} \langle \frac{4(J_1(qR\sin\theta)^2}{q^2 (R\sin\theta)^2} \rangle \sin\theta \, d\theta = \frac{2}{(qR)^2} - \frac{2J_1(2qR)}{(qR)^3}$$

Regime III. In the limit of high q we have (S.4.7.1.3)

$$P(q) \rightarrow \frac{2}{(qR)^2}$$

Taking the size average we have

$$2\,\langle\frac{1}{q^2R^2}\rangle = 2\,\frac{\Gamma[z-1]}{\Gamma[z+1]}\frac{(z+1)^2}{q^2R^2}$$

The figure shows the squared scattering amplitudes (series, asymptotic) and the formfactors of the parallel part and the orientationally averaged formfactor of the cross-sectional part (series, asymptotic)

Mathematica code implementation:

```
L=1;
R=3;
sigma=0.1;
z=(1-sigma*sigma)/(sigma*sigma);
nmax=80;
q=10^lq;
qs=Sqrt[qx*qx+qy*qy];
Pqpar=(Sqrt[Pi]/2)*Sum[(4^n)*Pochhammer[z+1,2*n]*((-q*q*L*L/(4*(z+1)*(z+1)))^n)/(Gamma[n+3/2]*(((n+1)!))), {n, 0,
nmax}];
Pqpars=(Sqrt[Pi]/2)*Sum[(4^n)*Pochhammer[z+1,2*n]*((-qs*qs*L*L/(4*(z+1)*(z+1)))^n)/(Gamma[n+3/2]*(((n+1)!))), {n, 0,
nmax}];
Pqcross=Sum[2*(4^n)*Pochhammer[z+1,2*n]*((-q*q*R*R/(4*(z+1)*(z+1)))^n)/( Pochhammer[2,n]*((n+2)!)), {n, 0, nmax}];
Pqcrosss=Sum[2*(4^n)*Pochhammer[z+1,2*n]*((-qs*qs*R*R/(4*(z+1)*(z+1)))^n)/( Pochhammer[2,n]*((n+2)!)), {n, 0,
nmax}];
ql=q*L/(z+1);
qr=q*R/(z+1);
Pqparas=(Gamma[z-1]/(2*Gamma[z+1]))*(ql^(-2))*(1-Cos[(z-1)*ArcTan[2*ql]]/((1+4*ql*ql)^((z-1)/2)));
Pqparasy=(Gamma[z-1]/(2*Gamma[z+1]))*(ql^(-2));
Pqcrossas=(2*Gamma[z-1]/(Gamma[z+1]))*(qr^(-2));
lim1=2;
lim2=2;
pl1=Plot[Log[10,Pqpar], {lq , -1,lim1}, PlotRange->{-4,1}, GridLines->{Automatic}, LabelStyle->Directive[Black,16],AxesLabel-
>{"log q","lg P(q)"}, AxesOrigin->{-1,-4},TicksStyle->Directive[Black,12],PlotStyle->{Black,Dashed}] ;
pl2=Plot[Log[10,Pqparas], {lq , -1,lim1}, PlotRange->{-4,1}, GridLines->{Automatic}, LabelStyle-
>Directive[Black,16],AxesLabel->{"log q","lg P(q)"}, AxesOrigin->{-1,-4},TicksStyle->Directive[Black,12],PlotStyle->{Blue}] ;
pl2a=Plot[Log[10,Pqparasy], {lq , -1,lim1}, PlotRange->{-4,1}, GridLines->{Automatic}, LabelStyle-
>Directive[Black,16],AxesLabel->{"log q","lg P(q)"}, AxesOrigin->{-1,-4},TicksStyle->Directive[Black,12],PlotStyle->{Red}] ;
(* pl1=Plot[Log[10,Pqc], {lq , -1,lim}, PlotRange⍰{-6,1}, GridLines->{Automatic}, LabelStyle->Directive[Black,16],AxesLabel-
>{"log q","lg P(q)"}, AxesOrigin->{-1,-6},TicksStyle->Directive[Black,12],PlotStyle->{Blue}]; *)
pl3=Plot[Log[10,Pqcross], {lq , -1,lim2}, PlotRange->{-6,1}, GridLines->{Automatic}, LabelStyle-
>Directive[Black,16],AxesLabel->{"log q","lg P(q)"}, AxesOrigin->{-1,-6},TicksStyle->Directive[Black,12],PlotStyle-
>{Black,Dashed}] ;
pl4=Plot[Log[10,Pqcrossas], {lq , -1,lim2}, PlotRange->{-6,1}, GridLines->{Automatic}, LabelStyle-
>Directive[Black,16],AxesLabel->{"log q","lg P(q)"}, AxesOrigin->{-1,-6},TicksStyle->Directive[Black,12],PlotStyle->{Red}] ;
Show[pl1,pl2,pl2a]
Show[pl3,pl4]
lims=9;
pl5=DensityPlot[Log[10,Pqpars],{qx, -lims, lims}, {qy, -lims, lims}, PlotRange->{-8,0}, PlotPoints->50, PlotLegends-
>Automatic, LabelStyle->Directive[Black,12],AxesLabel->Automatic]
pl6=DensityPlot[Log[10,Pqcrosss],{qx, -lims, lims}, {qy, -lims, lims}, PlotRange->{-8,0}, PlotPoints->50, PlotLegends-
>Automatic, LabelStyle->Directive[Black,12],AxesLabel->Automatic]
```

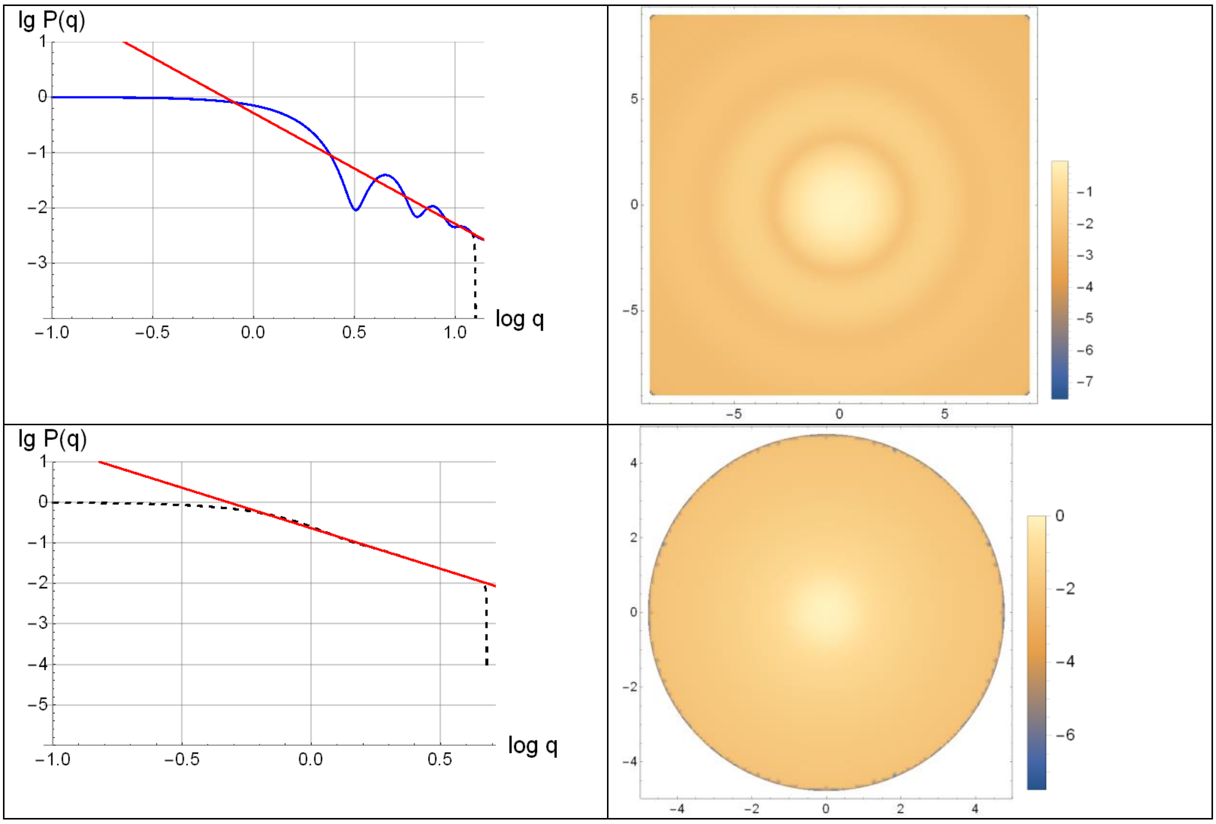

Fig. S28: Factorized formfactor of polydisperse isotropic disks, axial and cross-sectional parts.

4.7.2 Scattering amplitude F(q)

(S.4.7.2.1)

$$F(q) = \langle\frac{\sin(qL)}{qL}\rangle^2 \int_0^{\frac{\pi}{2}} \langle\frac{2J_1(qR\sin\theta)}{qR\sin\theta}\rangle^2 \sin\theta \, d\theta$$

Regime I. The series expansions in the polydisperse case are for the longitudinal part is given by (S.4.7.2.2)

$$\langle F_{\parallel}(q)\rangle^2 = \sum_{n=0}^{\infty}\left(-\frac{q^2L^2}{4(z+1)^2}\right)^n \sum_{m=0}^{n} \frac{(z+1)_{2(n-m)}(z+1)_{2m}}{\left(\frac{3}{2}\right)_{n-m}\left(\frac{3}{2}\right)_m (n-m)!\, m!}$$

$$\langle F_{\parallel}(q)\rangle^2 = \langle\frac{\sin(qL)}{qL}\rangle$$

Disks, polydisperse, factorized, isotropic, F(q)

Regime II. We need the average of (S.4.7.2.3)

$$\langle\frac{\sin(qL)}{qL}\rangle^2$$

with

$$\langle\frac{\sin(qL)}{qL}\rangle = \frac{\Gamma[z+s-1]}{\Gamma[z+s+1]}\left(\frac{qL}{z+1}\right)^{-2}\frac{\sin\left[(z+s-1)\arctan\left(\frac{qL}{z+1}\right)\right]}{\left(1+\left(\frac{qL}{z+1}\right)^{2}\right)^{\frac{z+s-1}{2}}}$$

Mathematica code implementation:

```
L=1;
R=3;
sigma=0.1;
z=(1-sigma*sigma)/(sigma*sigma);
nmax=80;
q=10^lq;
qs=Sqrt[qx*qx+qy*qy];
fff=Table[Sum[Pochhammer[z+1,2*(n-m)]*Pochhammer[z+1,2*m]/(Pochhammer[3/2,n-m]*Pochhammer[3/2,m]*((n-m)!)*(m!)), {m, 0, n}],{n, 0, nmax}];
Fqpar=Sum[(((-q*q*L*L/(4*(z+1)*(z+1)))^n)*fff[[n+1]]), {n, 0, nmax}];
Fqpars=Sum[(((-qs*qs*L*L/(4*(z+1)*(z+1)))^n)*fff[[n+1]]), {n, 0, nmax}];
ql=q*L/(z+1);
qr=q*R/(z+1);
Fqpara=((Gamma[z]/Gamma[z+1])*(ql^(-1))*Sin[z*ArcTan[ql]]/((1+ql*ql)^(z/2)))^2;
lim2=2;
pl3=Plot[Log[10,Fqpar], {lq , -1,lim2}, PlotRange->{-6,1}, GridLines->{Automatic}, LabelStyle->Directive[Black,16],AxesLabel->{"log q","lg F^2(q)"}, AxesOrigin->{-1,-6},TicksStyle->Directive[Black,12],PlotStyle->{Black,Dashed}] ;
pl4=Plot[Log[10,Fqpara], {lq , -1,lim2}, PlotRange->{-6,1}, GridLines->{Automatic}, LabelStyle->Directive[Black,16],AxesLabel->{"log q","lg F^2(q)"}, AxesOrigin->{-1,-6},TicksStyle->Directive[Black,12],PlotStyle->{Red}] ;
Show[pl3,pl4]
lims=18;
pl5=DensityPlot[Log[10,Fqpars],{qx, -lims, lims}, {qy, -lims, lims}, PlotRange->{-8,0}, PlotPoints->50, PlotLegends->Automatic, LabelStyle->Directive[Black,12],AxesLabel->Automatic]
```

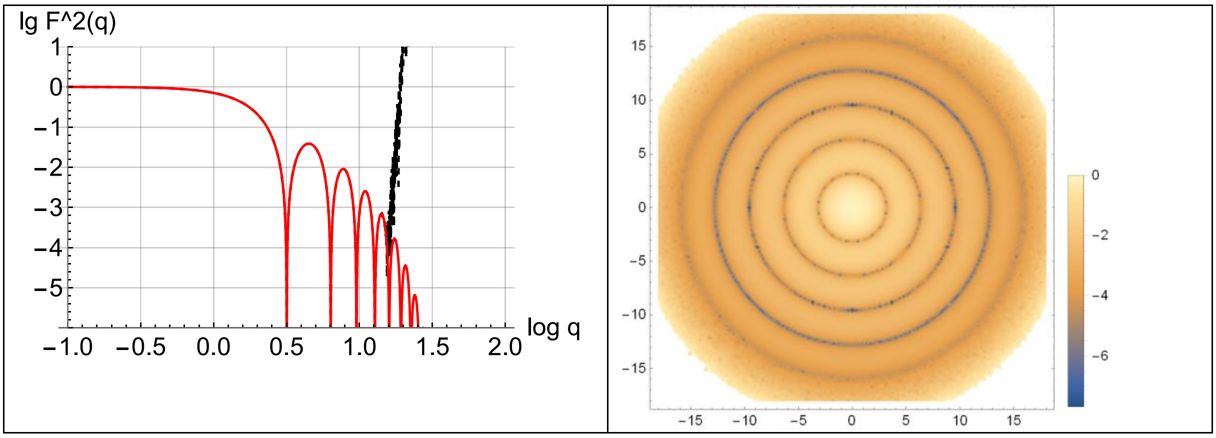

Fig. S29: Factorized scattering amplitude of polydisperse isotropic disks, cross-sectional part.

4.8 Superball

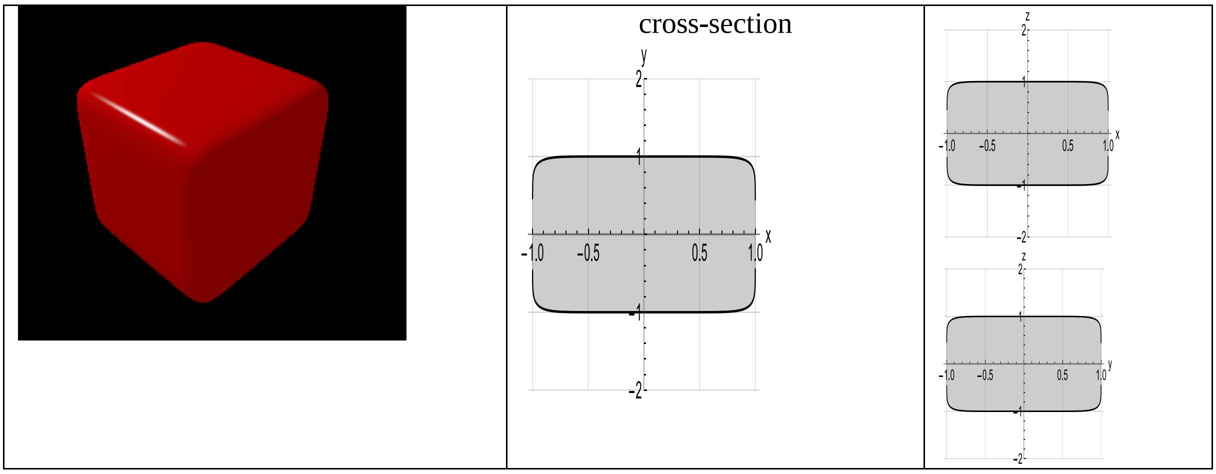

Fig. S30: Superball with cross-sections in the (*x*,*y*)-, (*x*,*z*)-, and (*y*,*z*)-planes.

The superball formfactor has been derived in ref. [19]. It involves five numerical integrations, two over the particle volume, one for the orientational distribution over each of the two polar angles, and a subsequent numerical integration over the size distribution. Our aim is to derive a fast overlapping converging series and asymptotic expansion.

The position vector $r = (x, y, z)$ to a point on the surface of the superball fulfills the equation (S.4.8.1)

$$\left(\frac{x}{a}\right)^p + \left(\frac{y}{b}\right)^q + \left(\frac{z}{c}\right)^r = 1$$

We are interested in the special case $p = q = r$. Then we have

$$\left(\frac{x}{a}\right)^p + \left(\frac{y}{b}\right)^p + \left(\frac{z}{c}\right)^p = 1$$

Therefore we have an expression for $z(x, y)$ (S.4.8.2)

$$\mathrm{z} = c\left(1 - \left(\frac{x}{a}\right)^p - \left(\frac{y}{b}\right)^p\right)^{1/p}$$

and for $z = 0$ an expression $y(x)$ that we need for the y-integration

$$\mathrm{y} = b\left(1 - \left(\frac{x}{a}\right)^p\right)^{1/p}$$

and for $y = z = 0$ finally an equation eeded for the x-integration

$$\mathrm{x} = a$$

Therefore, the integration over the volume of the superball is

$$F(q) = \frac{1}{V}\int_0^a \int_0^{b\left(1-\left(\frac{x}{a}\right)^p\right)^{1/p}} \int_0^{c\left(1-\left(\frac{x}{a}\right)^p+\left(\frac{y}{b}\right)^p\right)^{1/p}} \cos(q_x x)\cos(q_y y)\cos(q_z z)\, dzdydx$$

The first integral over *dz* can be done analytically to obtain

$$F(q) = \frac{1}{V}\frac{1}{q_z}\int_0^a \int_0^{b\left(1-\left(\frac{x}{a}\right)^p\right)^{1/p}} \cos(q_x x)\cos(q_y y)\sin\left(q_z c\left(1-\left(\frac{x}{a}\right)^p+\left(\frac{y}{b}\right)^p\right)^{1/p}\right) dydx$$

The remaining integration have to be done numerically, followed by taking the square, and numerically integrate over the two polar angles θ, ϕ.

We here take another approach and use the series expansions of $\cos(z)$ in terms of hypergeometric functions

$$\cos(q_x x) = \sum_{i=0}^{\infty} \frac{1}{\left(\frac{1}{2}\right)_i} \frac{\left(-\frac{q^2}{4}\right)^i}{i!} x^{2i} = \sum_{i=0}^{\infty} s_i x^{2i}$$

$$\cos\left(q_y y\right) = \sum_{j=0}^{\infty} \frac{1}{\left(\frac{1}{2}\right)_j} \frac{\left(-\frac{q^2}{4}\right)^j}{j!} y^{2j} = \sum_{j=0}^{\infty} s_j y^{2j}$$

$$\cos(q_z z) = \sum_{k=0}^{\infty} \frac{1}{\left(\frac{1}{2}\right)_k} \frac{\left(-\frac{q^2}{4}\right)^k}{k!} z^{2k} = \sum_{k=0}^{\infty} s_k z^{2k}$$

such that we can write for the

$$F(q) = \frac{1}{V}\sum_{i=0}^{\infty} s_i \sum_{j=0}^{\infty} s_j \sum_{k=0}^{\infty} s_k \int_0^a \int_0^{b\left(1-\left(\frac{x}{a}\right)^p\right)^{1/p}} \int_0^{c\left(1-\left(\frac{x}{a}\right)^p+\left(\frac{y}{b}\right)^p\right)^{1/p}} x^{2i} y^{2j} z^{2k} dzdydx$$

We transform the coordinates to $\xi = \frac{1}{h}\left(\frac{x}{a}\right)^p, \eta = \frac{1}{h}\left(\frac{y}{b}\right)^q, \zeta = \frac{1}{h}\left(\frac{z}{c}\right)^r$ such that $x = ah^{1/p}\xi^{1/p}, y = bh^{1/q}\,\eta^{1/q}, z = ch^{1/r}\,\zeta^{1/r}$ and $dx = \frac{a}{p}h^{1/p}\xi^{\frac{1}{p}-1}d\xi, dy = \frac{b}{q}h^{1/q}\,\eta^{\frac{1}{q}-1}d\eta, dz = \frac{c}{r}h^{1/r}\,\zeta^{\frac{1}{r}-1}d\zeta$. Then the equation (S.4.8.1) reads

$$\xi + \eta + \zeta = 1$$

with $\zeta = 1 - \xi$ for $\eta = 0$ and $\zeta = 1$ for $\eta, \zeta = 0$. Then the scattering amplitude is

$$F(q)=\frac{1}{V}\sum_{i=0}^{\infty}s_i\sum_{j=0}^{\infty}s_j\sum_{k=0}^{\infty}s_k\frac{a^{2i+1}b^{2j+1}c^{2k+1}}{p^3}\int_0^1\int_0^{1-\xi}\int_0^{1-\xi-\eta}\xi^{\frac{2i+1}{p}-1}\eta^{\frac{2j+1}{p}-1}\zeta^{\frac{2j+1}{p}-1}d\zeta d\eta d\xi$$

We now use the fact that

$$\int_0^1\int_0^{1-x}\int_0^{1-x-y}x^{l-1}y^{m-1}z^{n-1}dxdydz=\frac{\Gamma(l)\Gamma(m)\Gamma(n)}{\Gamma(l+m+n+1)}$$

such that we obtain

$$F(q)=\frac{1}{V}\sum_{i=0}^{\infty}s_i\sum_{j=0}^{\infty}s_j\sum_{k=0}^{\infty}s_k\frac{a^{2i+1}b^{2j+1}c^{2k+1}}{p^3}\frac{\Gamma\left(\frac{2i+1}{p}\right)\Gamma\left(\frac{2j+1}{p}\right)\Gamma\left(\frac{2k+1}{p}\right)}{\Gamma\left(\frac{2i+2j+2k+3}{p}+1\right)}$$

The volume is calculated as (S.4.8.3)

$$V=\int_0^a\int_0^{b\left(1-\left(\frac{x}{a}\right)^p\right)^{\frac{1}{p}}}\int_0^{c\left(1-\left(\frac{x}{a}\right)^p+\left(\frac{y}{b}\right)^p\right)^{\frac{1}{p}}}dzdydx$$

$$=\frac{abc}{p^3}\int_0^1\int_0^{1-\xi}\int_0^{1-\xi-\eta}\xi^{\frac{1}{p}-1}\eta^{\frac{1}{p}-1}\zeta^{\frac{1}{p}-1}d\zeta d\eta d\xi=\frac{abc}{p^3}\frac{\Gamma^3\left(\frac{1}{p}\right)}{\Gamma\left(\frac{3}{p}+1\right)}$$

Such that (S.4.8.4)

$$F(q)=\frac{p^3\Gamma\left(\frac{3}{p}+1\right)}{\Gamma^3\left(\frac{1}{p}\right)}\sum_{i=0}^{\infty}s_i\sum_{j=0}^{\infty}s_j\sum_{k=0}^{\infty}s_k\frac{a^{2i}b^{2j}c^{2k}}{p^3}\frac{\Gamma\left(\frac{2i+1}{p}\right)\Gamma\left(\frac{2j+1}{p}\right)\Gamma\left(\frac{2k+1}{p}\right)}{\Gamma\left(\frac{2i+2j+2k+3}{p}+1\right)}$$

$$=\frac{\Gamma\left(\frac{3}{p}+1\right)}{\Gamma^3\left(\frac{1}{p}\right)}\sum_{n=0}^{\infty}\frac{\Gamma\left(\frac{2n+1}{p}\right)}{\left(\frac{1}{2}\right)_n}\frac{\left(-\frac{q_x^2a^2}{4}\right)^n}{n!}\sum_{m=0}^{\infty}\frac{\Gamma\left(\frac{2m+1}{p}\right)}{\left(\frac{1}{2}\right)_m}\frac{\left(-\frac{q_y^2b^2}{4}\right)^m}{m!}\sum_{k=0}^{\infty}\frac{\Gamma\left(\frac{2k+1}{p}\right)}{\left(\frac{1}{2}\right)_k}\frac{\left(-\frac{q_z^2c^2}{4}\right)^k}{k!}\frac{1}{\Gamma\left(\frac{2i+2j+2k+3}{p}+1\right)}$$

and for the formfactor P(q) (S.4.8.5)

$$P(q)=\frac{\Gamma^2\left(\frac{3}{p}+1\right)}{\Gamma^6\left(\frac{1}{p}\right)}\sum_{n=0}^{\infty}\left(-\frac{q_x^2a^2}{4}\right)^n\sum_{m=0}^{\infty}\left(-\frac{q_y^2b^2}{4}\right)^m\sum_{k=0}^{\infty}\left(-\frac{q_z^2c^2}{4}\right)^k\sum_{n'=0}^{n}\frac{\Gamma\left(\frac{2(n-n')+1}{p}\right)\Gamma\left(\frac{2n'+1}{p}\right)}{\left(\frac{1}{2}\right)_{n-n'}\left(\frac{1}{2}\right)_{n'}(n-n')!\,(n')!}\sum_{m'}^{m}\frac{\Gamma\left(\frac{2(m-m')+1}{p}\right)\Gamma\left(\frac{2m'+1}{p}\right)}{\left(\frac{1}{2}\right)_{m-m'}\left(\frac{1}{2}\right)_{m'}(m-m')!\,(m')!}\sum_{k'}^{k}\frac{\Gamma\left(\frac{2(k-k')+1}{p}\right)\Gamma\left(\frac{2k'+1}{p}\right)}{\left(\frac{1}{2}\right)_{k-k'}\left(\frac{1}{2}\right)_{k'}(k-k')!\,(k')!}\frac{1}{\Gamma\left(\frac{2(n-n')+2(m-m')+2(k-k')+3}{p}+1\right)}\frac{1}{\Gamma\left(\frac{2n'+2m'+2k'+3}{p}+1\right)}$$

$$P(q)=\frac{\Gamma^2\left(\frac{3}{p}+1\right)}{\Gamma^6\left(\frac{1}{p}\right)}\sum_{n=0}^{\infty}\left(-\frac{q_x^2a^2}{4}\right)^n\sum_{m=0}^{\infty}\left(-\frac{q_y^2b^2}{4}\right)^m\sum_{k=0}^{\infty}\left(-\frac{q_z^2c^2}{4}\right)^k c_{n,m,k}$$

with

$$c_{n,m,k}=\sum_{n'=0}^{n}\frac{\Gamma\left(\frac{2(n-n')+1}{p}\right)\Gamma\left(\frac{2n'+1}{p}\right)}{\left(\frac{1}{2}\right)_{n-n'}\left(\frac{1}{2}\right)_{n'}(n-n')!\,(n')!}\sum_{m'}^{m}\frac{\Gamma\left(\frac{2(m-m')+1}{p}\right)\Gamma\left(\frac{2m'+1}{p}\right)}{\left(\frac{1}{2}\right)_{m-m'}\left(\frac{1}{2}\right)_{m'}(m-m')!\,(m')!}\sum_{k'}^{k}\frac{\Gamma\left(\frac{2(k-k')+1}{p}\right)\Gamma\left(\frac{2k'+1}{p}\right)}{\left(\frac{1}{2}\right)_{k-k'}\left(\frac{1}{2}\right)_{k'}(k-k')!\,(k')!}\frac{1}{\Gamma\left(\frac{2(n-n')+2(m-m')+2(k-k')+3}{p}+1\right)}\frac{1}{\Gamma\left(\frac{2n'+2m'+2k'+3}{p}+1\right)}$$

Introducing the size distribution we have for $\langle F(q)\rangle^2$ (S.4.8.6)

$$\langle F(q)\rangle^2 = \frac{\Gamma^2\left(\frac{3}{p}+1\right)}{\Gamma^6\left(\frac{1}{p}\right)}\sum_{n=0}^{\infty}\left(-\frac{q_x^2a^2}{4(z+1)^2}\right)^n\sum_{n'=0}^{n}\frac{(z+s+1)_{2(n-n')}\,(z+s+1)_{2n'}\Gamma\left(\frac{2(n-n')+1}{p}\right)\Gamma\left(\frac{2n'+1}{p}\right)}{\left(\frac{1}{2}\right)_{n-n'}\left(\frac{1}{2}\right)_{n'}(n-n')!\,(n')!}\sum_{m=0}^{\infty}\left(-\frac{q_y^2b^2}{4(z+1)^2}\right)^m\sum_{m'}^{m}\frac{(z+s+1)_{2(m-m')}\,(z+s+1)_{2m'}\Gamma\left(\frac{2(m-m')+1}{p}\right)\Gamma\left(\frac{2m'+1}{p}\right)}{\left(\frac{1}{2}\right)_{m-m'}\left(\frac{1}{2}\right)_{m'}(m-m')!\,(m')!}\sum_{k=0}^{\infty}\left(-\frac{q_z^2c^2}{4(z+1)^2}\right)^k\sum_{k'}^{k}\frac{\Gamma\left(\frac{2(k-k')+1}{p}\right)\Gamma\left(\frac{2k'+1}{p}\right)}{\left(\frac{1}{2}\right)_{k-k'}\left(\frac{1}{2}\right)_{k'}(k-k')!\,(k')!}$$

$$\frac{(z+s+1)_{2(k-k')}\,(z+s+1)_{2k'}}{\Gamma\left(\frac{2(n-n')+2(m-m')+2(k-k')+3}{p}+1\right)}\frac{1}{\Gamma\left(\frac{2n'+2m'+2k'+3}{p}+1\right)}$$

$$\langle F(q)\rangle^2 = \frac{\Gamma^2\left(\frac{3}{p}+1\right)}{\Gamma^6\left(\frac{1}{p}\right)}\sum_{n=0}^{\infty}\left(-\frac{q_x^2a^2}{4(z+1)^2}\right)^n f_n\sum_{m=0}^{\infty}\left(-\frac{q_y^2b^2}{4(z+1)^2}\right)^m f_m\sum_{k=0}^{\infty}\left(-\frac{q_z^2c^2}{4(z+1)^2}\right)^k f_{n,m,k}$$

$$f_n = \sum_{n'=0}^{n}\frac{(z+s+1)_{2(n-n')}\,(z+s+1)_{2n'}\Gamma\left(\frac{2(n-n')+1}{p}\right)\Gamma\left(\frac{2n'+1}{p}\right)}{\left(\frac{1}{2}\right)_{n-n'}\left(\frac{1}{2}\right)_{n'}(n-n')!\,(n')!}$$

$$f_m = \sum_{m'}^{m}\frac{(z+s+1)_{2(m-m')}\,(z+s+1)_{2m'}\Gamma\left(\frac{2(m-m')+1}{p}\right)\Gamma\left(\frac{2m'+1}{p}\right)}{\left(\frac{1}{2}\right)_{m-m'}\left(\frac{1}{2}\right)_{m'}(m-m')!\,(m')!}$$

$$f_{n,m,k} = \sum_{k'}^{k}\frac{\Gamma\left(\frac{2(k-k')+1}{p}\right)\Gamma\left(\frac{2k'+1}{p}\right)}{\left(\frac{1}{2}\right)_{k-k'}\left(\frac{1}{2}\right)_{k'}(k-k')!\,(k')!}\frac{(z+s+1)_{2(k-k')}\,(z+s+1)_{2k'}}{\Gamma\left(\frac{2(n-n')+2(m-m')+2(k-k')+3}{p}+1\right)}\frac{1}{\Gamma\left(\frac{2n'+2m'+2k'+3}{p}+1\right)}$$

and for the formfactor P(q) (S.4.8.7)

$$\langle P(q)\rangle = \frac{\Gamma^2\left(\frac{3}{p}+1\right)}{\Gamma^6\left(\frac{1}{p}\right)}\sum_{n=0}^{\infty}(z+s+1)_{2n}\left(-\frac{q_x^2a^2}{4(z+1)^2}\right)^n\sum_{n'=0}^{n}\frac{\Gamma\left(\frac{2(n-n')+1}{p}\right)\Gamma\left(\frac{2n'+1}{p}\right)}{\left(\frac{1}{2}\right)_{n-n'}\left(\frac{1}{2}\right)_{n'}(n-n')!\,(n')!}\sum_{m=0}^{\infty}(z+s+1)_{2m}\left(-\frac{q_y^2b^2}{4(z+1)^2}\right)^m\sum_{m'}^{m}\frac{\Gamma\left(\frac{2(m-m')+1}{p}\right)\Gamma\left(\frac{2m'+1}{p}\right)}{\left(\frac{1}{2}\right)_{m-m'}\left(\frac{1}{2}\right)_{m'}(m-m')!\,(m')!}\sum_{k=0}^{\infty}(z+s+1)_{2k}\left(-\frac{q_z^2c^2}{4(z+1)^2}\right)^k\sum_{k'}^{k}\frac{\Gamma\left(\frac{2(k-k')+1}{p}\right)\Gamma\left(\frac{2k'+1}{p}\right)}{\left(\frac{1}{2}\right)_{k-k'}\left(\frac{1}{2}\right)_{k'}(k-k')!\,(k')!}\frac{1}{\Gamma\left(\frac{2(n-n')+2(m-m')+2(k-k')+3}{p}+1\right)}\frac{1}{\Gamma\left(\frac{2n'+2m'+2k'+3}{p}+1\right)}$$

$$= \frac{\Gamma^2\left(\frac{3}{p}+1\right)}{\Gamma^6\left(\frac{1}{p}\right)}\sum_{n=0}^{\infty}(z+s+1)_{2n}\left(-\frac{q_x^2a^2}{4(z+1)^2}\right)^n\sum_{m=0}^{\infty}(z+s+1)_{2m}\left(-\frac{q_y^2b^2}{4(z+1)^2}\right)^m\sum_{k=0}^{\infty}(z+s+1)_{2k}\left(-\frac{q_z^2c^2}{4(z+1)^2}\right)^k c_{n,m,k}$$

This is the expression for polydisperse, oriented superball.

For the isotropic case, the remaining two integrations over the angles can be performed analytically. We have

$$\langle F(q)\rangle^2 = \frac{\Gamma^2\left(\frac{3}{p}+1\right)}{\Gamma^6\left(\frac{1}{p}\right)}\sum_{n=0}^{\infty}\left(-\frac{q^2a^2}{4(z+1)^2}\right)^n f_n\sum_{m=0}^{\infty}\left(-\frac{q^2b^2}{4(z+1)^2}\right)^m f_m\sum_{k=0}^{\infty}\left(-\frac{q^2c^2}{4(z+1)^2}\right)^k f_{n,m,k}\frac{1}{2\pi}\int_0^{2\pi}(\cos\varphi)^{2n}(\sin\varphi)^{2m}d\varphi\int_0^{\pi/2}(\cos\theta)^{2k}(\sin\theta)^{2n+2m}\sin\theta\,d\,\theta$$

$$\langle P(q)\rangle = \frac{\Gamma^2\left(\frac{3}{p}+1\right)}{\Gamma^6\left(\frac{1}{p}\right)}\sum_{n=0}^{\infty}\left(-\frac{q^2a^2}{4(z+1)^2}\right)^n\sum_{m=0}^{\infty}\left(-\frac{q^2b^2}{4(z+1)^2}\right)^m\sum_{k=0}^{\infty}\left(-\frac{q^2c^2}{4(z+1)^2}\right)^k c_{n,m,k}\frac{1}{2\pi}\int_0^{2\pi}(\cos\varphi)^{2n}(\sin\varphi)^{2m}d\varphi\int_0^{\pi/2}(\cos\theta)^{2k}(\sin\theta)^{2n+2m}\sin\theta\,d\,\theta$$

The integrals are

$$\left(\frac{1}{2\pi}\int_0^{2\pi}(\cos\varphi)^{2n}(\sin\varphi)^{2m}d\varphi\right)\left(\int_0^{\frac{\pi}{2}}(\cos\theta)^{2k}(\sin\theta)^{2n+2m}\sin\theta\, d\theta\right)$$
$$=\left(\frac{1}{\pi}\frac{\Gamma\left(n+\frac{1}{2}\right)\Gamma\left(m+\frac{1}{2}\right)}{\Gamma(n+m+1)}\right)\left(\frac{\Gamma\left(k+\frac{1}{2}\right)\Gamma(n+m+1)}{2\Gamma\left(n+m+k+\frac{3}{2}\right)}\right)$$
$$=\frac{1}{2\pi}\frac{\Gamma\left(n+\frac{1}{2}\right)\Gamma\left(m+\frac{1}{2}\right)\Gamma\left(k+\frac{1}{2}\right)}{\Gamma\left(n+m+k+\frac{3}{2}\right)}$$

Such that we obtain for the scattering amplitude (S.4.8.8)

$$\langle F(q)\rangle^2=\frac{\Gamma^2\left(\frac{3}{p}+1\right)}{\Gamma^6\left(\frac{1}{p}\right)}\sum_{n=0}^{\infty}\left(-\frac{q^2a^2}{4(z+1)^2}\right)^n f_n\sum_{m=0}^{\infty}\left(-\frac{q^2b^2}{4(z+1)^2}\right)^m f_m\sum_{k=0}^{\infty}\left(-\frac{q^2c^2}{4(z+1)^2}\right)^k f_{n,m,k}\frac{1}{2\pi}\frac{\Gamma\left(n+\frac{1}{2}\right)\Gamma\left(m+\frac{1}{2}\right)\Gamma\left(k+\frac{1}{2}\right)}{\Gamma\left(n+m+k+\frac{3}{2}\right)}$$

$$\langle F(q)\rangle^2=\frac{\Gamma^2\left(\frac{3}{p}+1\right)}{2\pi\Gamma^6\left(\frac{1}{p}\right)}\sum_{n=0}^{\infty}\Gamma\left(n+\frac{1}{2}\right)\left(-\frac{q^2a^2}{4(z+1)^2}\right)^n f_n\sum_{m=0}^{\infty}\Gamma\left(m+\frac{1}{2}\right)\left(-\frac{q^2b^2}{4(z+1)^2}\right)^m f_m\sum_{k=0}^{\infty}\left(-\frac{q^2c^2}{4(z+1)^2}\right)^k f_{n,m,k}\frac{\Gamma\left(k+\frac{1}{2}\right)}{\Gamma\left(n+m+k+\frac{3}{2}\right)}$$

supercube, polydisperse, isotropic, F(q)

and for the formfactor (S.4.8.9)

$$\langle P(q)\rangle=\frac{\Gamma^2\left(\frac{3}{p}+1\right)}{\Gamma^6\left(\frac{1}{p}\right)}\sum_{n=0}^{\infty}(z+s+1)_{2n}\left(-\frac{q^2a^2}{4(z+1)^2}\right)^n\sum_{m=0}^{\infty}(z+s+1)_{2m}\left(-\frac{q^2b^2}{4(z+1)^2}\right)^m\sum_{k=0}^{\infty}(z+s+1)_{2k}\left(-\frac{q^2c^2}{4(z+1)^2}\right)^k c_{n,m,k}\frac{1}{2\pi}\frac{\Gamma\left(n+\frac{1}{2}\right)\Gamma\left(m+\frac{1}{2}\right)\Gamma\left(k+\frac{1}{2}\right)}{\Gamma\left(n+m+k+\frac{3}{2}\right)}$$

$$\langle P(q)\rangle=\frac{1}{2\pi}\frac{\Gamma^2\left(\frac{3}{p}+1\right)}{\Gamma^6\left(\frac{1}{p}\right)}\sum_{n=0}^{\infty}(z+s+1)_{2n}\Gamma\left(n+\frac{1}{2}\right)\left(-\frac{q^2a^2}{4(z+1)^2}\right)^n\sum_{m=0}^{\infty}(z+s+1)_{2m}\Gamma\left(m+\frac{1}{2}\right)\left(-\frac{q_y^2b^2}{4(z+1)^2}\right)^m\sum_{k=0}^{\infty}(z+s+1)_{2k}\Gamma\left(k+\frac{1}{2}\right)\left(-\frac{q_z^2c^2}{4(z+1)^2}\right)^k\frac{c_{n,m,k}}{\Gamma\left(n+m+k+\frac{3}{2}\right)}$$

supercube, polydisperse, isotropic, P(q)

$$=\frac{1}{2\pi}\frac{\Gamma^2\left(\frac{3}{p}+1\right)}{\Gamma^6\left(\frac{1}{p}\right)}\sum_{n=0}^{\infty}\sum_{m=0}^{\infty}\sum_{k=0}^{\infty}(z+s+1)_{2n}\Gamma\left(n+\frac{1}{2}\right)\left(-\frac{q^2a^2}{4(z+1)^2}\right)^n(z+s+1)_{2m}\Gamma\left(m+\frac{1}{2}\right)\left(-\frac{q^2b^2}{4(z+1)^2}\right)^m(z+s+1)_{2k}\Gamma\left(k+\frac{1}{2}\right)\left(-\frac{q^2c^2}{4(z+1)^2}\right)^k\frac{c_{n,m,k}}{\Gamma\left(n+m+k+\frac{3}{2}\right)}$$

$$=\frac{1}{2\pi}\frac{\Gamma^2\left(\frac{3}{p}+1\right)}{\Gamma^6\left(\frac{1}{p}\right)}\sum_{n=0}^{\infty}\frac{1}{\Gamma\left(n+\frac{3}{2}\right)}\left(-\frac{q^2}{4(z+1)^2}\right)^n\sum_{m=0}^{n}(z+s+1)_{2(n-m)}\Gamma\left(n-m+\frac{1}{2}\right)(a^2)^{n-m}\sum_{k=0}^{m}(z+s+1)_{2(m-k)}\Gamma\left(m-k+\frac{1}{2}\right)(b^2)^{m-k}(z+s+1)_{2k}\,\Gamma\left(k+\frac{1}{2}\right)c^{2k}c_{n-m,m-k,k}$$

For supercube with $a = b = c$: (S.4.8.10)

$$=\frac{1}{2\pi}\frac{\Gamma^2\left(\frac{3}{p}+1\right)}{\Gamma^6\left(\frac{1}{p}\right)}\sum_{n=0}^{\infty}\frac{1}{\Gamma\left(n+\frac{3}{2}\right)}\left(-\frac{a^2q^2}{4(z+1)^2}\right)^n\sum_{m=0}^{n}(z+s+1)_{2(n-m)}\Gamma\left(n-m+\frac{1}{2}\right)\sum_{k=0}^{m}(z+s+1)_{2(m-k)}\Gamma\left(m-k+\frac{1}{2}\right)(z+s+1)_{2k}\,\Gamma\left(k+\frac{1}{2}\right)c_{n-m,m-k,k}$$

Regime II. Numerical Integration

The integral has to be integrated numerically, then over the two angles, and then over the size distribution

$$F(q)=\frac{1}{V}\frac{1}{q_z}\int_0^a\int_0^{b\left(1-\left(\frac{x}{a}\right)^p\right)^{1/p}}\cos(q_x x)\cos(q_y y)\sin\left(q_z c\left(1-\left(\frac{x}{a}\right)^p+\left(\frac{y}{b}\right)^p\right)^{1/p}\right)dydx$$

As there is good overlap between the series and the asymptotic expansion, this only needs to be done in special cases.

Regime III. Porod Asymptote.

For the Porod-asymptote we need to calculate the surface area. For this we need to calculate the differential surface element DS from z(x,y) (Eq. S.4.8.2) with

$$\frac{\partial z}{\partial x}=z_x=-\frac{c\left(\frac{x}{a}\right)^{p-1}\left(1-\left(\frac{x}{a}\right)^p-\left(\frac{y}{b}\right)^p\right)^{\frac{1}{p}-1}}{a}$$

$$\frac{\partial z}{\partial y}=z_y=-\frac{c\left(\frac{y}{b}\right)^{p-1}\left(1-\left(\frac{x}{a}\right)^p-\left(\frac{y}{b}\right)^p\right)^{\frac{1}{p}-1}}{b}$$

$$DS=\sqrt{1+z_x^2+z_y^2}$$

The surface area is then obtained by numerically integrating over the (x,y)-plane as

$$A=8\int_0^a\int_0^{b\left(1-\left(\frac{x}{a}\right)^p\right)^{1/p}}DSdydx$$

From this, the Porod-asymptote can be calculated as (S.4.8.11)

$$P(q) = \frac{\Gamma[z+s-3](z+1)^4}{\Gamma[z+s+1]}\frac{2\pi A}{V^2 q^4}$$

An alternative numerical integration to obtain the surface volume, which can be faster for large p proceeds via the respective polar coordinates. For the superball we have the radial distance between the center a point on the surface given as

$$r = \frac{abc}{((bc)^p|\sin\theta|^p|\cos\phi|^p + (ac)^p|\sin\theta|^p|\sin\phi|^p + (ab)^p|\cos\theta|^p)^{1/m}}$$

The coordinates for a vector $\boldsymbol{r}$=(x,y,z) to the surface are given by

$$x = r\sin\theta\cos\phi$$

$$y = r\sin\theta\sin\phi$$

$$z = r\cos\theta$$

With the partial derivative vectors $\boldsymbol{r}_\theta = \frac{\partial \boldsymbol{r}}{\partial\theta}$ and $\boldsymbol{r}_\phi = \frac{\partial \boldsymbol{r}}{\partial\phi}$ we compute the modulus of the cross-product to obtain

$$|\boldsymbol{r}_\theta \times \boldsymbol{r}_\phi| = \frac{(abc)^2\sqrt{(ab)^{2p}(\cos\theta)^{2p-2}(\sin\theta)^2 + [b^{2p}(\cos\phi)^{2p-2} + a^{2p}(\sin\phi)^{2p-2}](c\sin\theta)^{2p}}}{((ab\cos\theta)^p + [(b\cos\phi)^p + (a\sin\phi)^p](c\sin\theta)^p)^{(2+m)/m}}$$

This is then integrated over the polar angles to obtain the surface area

$$A = 8\int_0^{\pi/2}\int_0^{\pi/2} |\boldsymbol{r}_\theta \times \boldsymbol{r}_\phi| d\phi d\theta$$

```
r=a*b*c*(((((b*c)^m)*(Cos[phi])^m)*((Sin[theta])^m)+((a*c)^m)*((Sin[phi])^m)*((Sin[theta])^
m)+((a*b)^m)*((Cos[theta])^m))^(-1/m));
x1=r*Sin[theta]*Cos[phi];
x2=r*Sin[theta]*Sin[phi];
x3=r*Cos[theta];
xv={x1,x2,x3};
xth=D[xv,theta];
xph=D[xv,phi];
xx=Cross[xth,xph];
xx1=Simplify[xx];
xa=xx1[[1]];
xb=xx1[[2]];
xc=xx1[[3]];
DS=Simplify[Sqrt[xa*xa+xb*xb+xc*xc]]
area=8*NIntegrate[DS,{phi,0,Pi/2},{theta,0,Pi/2}]
```

$$\sqrt{}(a^4b^4c^4((ab)^m\text{Cos[theta]}^m + ((bc)^m\text{Cos[phi]}^m + (ac)^m\text{Sin[phi]}^m)\text{Sin[theta]}^m)^{-\frac{2(2+m)}{m}}((ab)^{2m}\text{Cos[theta]}^{-2+2m}\text{Sin[theta]}^2 + ((bc)^{2m}\text{Cos[phi]}^{-2+2m} + (ac)^{2m}\text{Sin[phi]}^{-2+2m})\text{Sin[theta]}^{2m}))$$

Mathematica code implementation:

```
max=15;
na=max;
ma=max;
la=max;
k=2;
fff=Table[Sum[(Gamma[(2*ns+1)/k]*Gamma[(2*(n-ns)+1)/k]/(Pochhammer[1/2,ns]*(ns!)*Pochhammer[1/2,n-ns]*((n-ns)!)))*
    Sum[(Gamma[(2*ms+1)/k]*Gamma[(2*(m-ms)+1)/k]/(Pochhammer[1/2,ms]*(ms!)*Pochhammer[1/2,m-ms]*((m-ms)!)))*Sum[(Gamma[(2*ls+1)/k]*Gamma[(2*(l-ls)+1)/k]/(Pochhammer[1/2,ls]*(ls!)*Pochhammer[1/2,l-ls]*((l-ls)!)))*(1/(Gamma[(2*(ns+ms+ls)+3)/k+1]*Gamma[(2*(n-ns+m-ms+l-ls)+3)/k+1])),{ls, 0, l}],{ms, 0, m}],{ns, 0, n}], {n, 0, na}, {m, 0, ma},{l, 0, la}];
a1=((Gamma[3/k+1])^2)/((Gamma[1/k])^6);

a=1.0;
b=1.0;
c=1.0;
sigma=0.14;
z=(1-sigma*sigma)/(sigma*sigma);
q=10^lq;
qs=Sqrt[qx*qx+qy*qy];
vol=8*a*b*c*((Gamma[1/k])^3)/((k^3)*Gamma[1+3/k]);
zx=-((c (x/a)^(-1+k) (1 - (x / a)^k - (y / b)^k)^(-1+1/k) )/a);
zy=-((c (y/b)^(-1+k) (1 - (x / a)^k - (y / b)^k)^(-1+1/k) )/b);
DS=Sqrt[1+zx*zx+zy*zy];
area=8*NIntegrate[DS, {x, 0, a}, {y, 0, b*(1-(x/a)^k)^(1/k)}];
apor=Gamma[z-3]*((z+1)^4)/Gamma[z+1];
Pqpor=apor*2*Pi*area/((q^4)*vol*vol);
Pqav=(a1/(2*Pi))*Sum[(Gamma[n+1/2]*((-q*q*a*a/4)^n))*Sum[(Gamma[m+1/2]*((-q*q*b*b/4)^m))*Sum[(Gamma[l+1/2]*((-q*q*c*c/4)^l)/Gamma[n+m+l+3/2])*fff[[n+1,m+1,l+1]], {l, 0, max}],{m, 0, max}], {n , 0, max}];
Pqav1=(a1/(2*Pi))*Sum[(((-q*q/4)^n)/Gamma[n+3/2])*Sum[(Gamma[n-m+1/2]*(a^(2*(n-m))))*Sum[Gamma[m-l+1/2]*(b^(2*(m-l)))*Gamma[l+1/2]*(c^(2*l))*fff[[n-m+1,m-l+1,l+1]], {l, 0, m}],{m, 0, n}], {n , 0, max}];
Pqav1z=(a1/(2*Pi))*Sum[(((-q*q/(4*(z+1)*(z+1)))^n)/Gamma[n+3/2])*Sum[(Pochhammer[z+1,2*(n-m)]*Gamma[n-m+1/2]*(a^(2*(n-m))))*Sum[Pochhammer[z+1,2*(m-l)]*Gamma[m-l+1/2]*(b^(2*(m-l)))*Pochhammer[z+1,2*l]*Gamma[l+1/2]*(c^(2*l))*fff[[n-m+1,m-l+1,l+1]], {l, 0, m}],{m, 0, n}], {n , 0, max}];
Pqavs=(a1/(2*Pi))*Sum[(Gamma[n+1/2]*((-qs*qs*a*a/4)^n))*Sum[(Gamma[m+1/2]*((-qs*qs*b*b/4)^m))*Sum[(Gamma[l+1/2]*((-qs*qs*c*c/4)^l)/Gamma[n+m+l+3/2])*fff[[n+1,m+1,l+1]], {l, 0, max}],{m, 0, max}], {n , 0, max}];
lim=2;
(* pl1=Plot[Log[10,Pqav], {lq , -1,lim1}, PlotRange□{-4,1}, GridLines->{Automatic}, LabelStyle->Directive[Black,16],AxesLabel->{"log q","lg P(q)"}, AxesOrigin->{-1,-4},TicksStyle->Directive[Black,12],PlotStyle->{Black,Dashed}] ; *)
pl2=Plot[Log[10,Pqav1], {lq , -1,lim}, PlotRange->{-6,1}, GridLines->{Automatic}, LabelStyle->Directive[Black,16],AxesLabel->{"log q","lg P(q)"}, AxesOrigin->{-1,-6},TicksStyle->Directive[Black,12],PlotStyle->{Black}] ;
pl3=Plot[Log[10,Pqav1z], {lq , -1,lim}, PlotRange->{-6,1}, GridLines->{Automatic}, LabelStyle->Directive[Black,16],AxesLabel->{"log q","lg P(q)"}, AxesOrigin->{-1,-6},TicksStyle->Directive[Black,12],PlotStyle->{Blue}]  ;
pl4=Plot[Log[10,Pqpor], {lq , -1,lim}, PlotRange->{-6,1}, GridLines->{Automatic}, LabelStyle->Directive[Black,16],AxesLabel->{"log q","lg P(q)"}, AxesOrigin->{-1,-6},TicksStyle->Directive[Black,12],PlotStyle->{Red}]  ;
Show[pl2,pl3,pl4]
lims=9.0;
(* pl5=DensityPlot[Log[10,Pqavs],{qx, -lims, lims}, {qy, -lims, lims}, PlotRange->{-8,0}, PlotPoints->50, PlotLegends->Automatic, LabelStyle□Directive[Black,12],AxesLabel->Automatic]*)
```

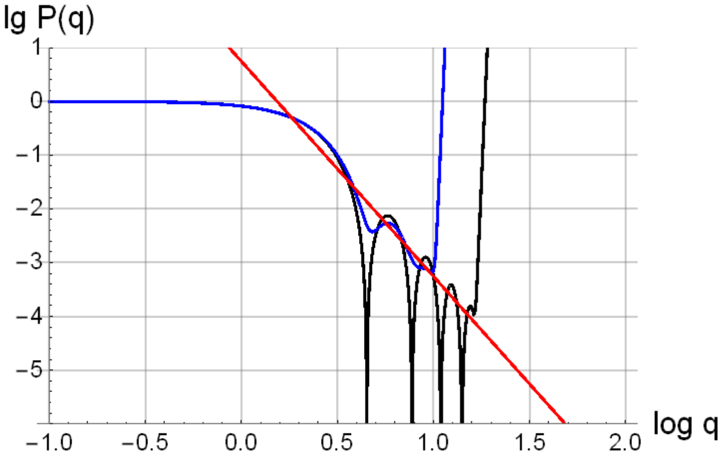

Fig. S31: Formfactor of monodisperse and polydisperse isotropic supercubes.

Scattering Amplitude F(q)

Regime I: We use equation (S.4.8.8) for the polydisperse case

$$\langle F(q)\rangle^2 = \frac{\Gamma^2\left(\frac{3}{p}+1\right)}{2\pi\Gamma^6\left(\frac{1}{p}\right)} \sum_{n=0}^{\infty}\Gamma\left(n+\frac{1}{2}\right)\left(-\frac{q^2a^2}{4(z+1)^2}\right)^n f_n \sum_{m=0}^{\infty}\Gamma\left(m+\frac{1}{2}\right)\left(-\frac{q^2b^2}{4(z+1)^2}\right)^m f_m \sum_{k=0}^{\infty}\left(-\frac{q^2c^2}{4(z+1)^2}\right)^k f_{n,m,k}\frac{\Gamma\left(k+\frac{1}{2}\right)}{\Gamma\left(n+m+k+\frac{3}{2}\right)}$$

No Regime II and III

Mathematica code implementation:

```
max=10;
na=max;
ma=max;
la=max;
k=2;
ccc=Table[Sum[(Gamma[(2*ns+1)/k]*Gamma[(2*(n-ns)+1)/k]/(Pochhammer[1/2,ns]*(ns!)*Pochhammer[1/2,n-ns]*((n-
ns)!)))*
    Sum[(Gamma[(2*ms+1)/k]*Gamma[(2*(m-ms)+1)/k]/(Pochhammer[1/2,ms]*(ms!)*Pochhammer[1/2,m-ms]*((m-
ms)!)))*Sum[(Gamma[(2*ls+1)/k]*Gamma[(2*(l-ls)+1)/k]/(Pochhammer[1/2,ls]*(ls!)*Pochhammer[1/2,l-ls]*((l-
ls)!)))*(1/(Gamma[(2*(ns+ms+ls)+3)/k+1]*Gamma[(2*(n-ns+m-ms+l-ls)+3)/k+1])),{ls, 0, l}],{ms, 0, m}],{ns, 0, n}], {n, 0, na},
{m, 0, ma},{l, 0, la}];
fff=Table[Sum[(Gamma[(2*ns+1)/k]*Gamma[(2*(n-ns)+1)/k]*Pochhammer[z+1,2*(n-
ns)]*Pochhammer[z+1,2*ns]/(Pochhammer[1/2,ns]*(ns!)*Pochhammer[1/2,n-ns]*((n-ns)!)))*
    Sum[(Gamma[(2*ms+1)/k]*Gamma[(2*(m-ms)+1)/k]*Pochhammer[z+1,2*(m-
ms)]*Pochhammer[z+1,2*ms]/(Pochhammer[1/2,ms]*(ms!)*Pochhammer[1/2,m-ms]*((m-
ms)!)))*Sum[(Gamma[(2*ls+1)/k]*Gamma[(2*(l-ls)+1)/k]*Pochhammer[z+1,2*(l-
ls)]*Pochhammer[z+1,2*ls]/(Pochhammer[1/2,ls]*(ls!)*Pochhammer[1/2,l-ls]*((l-
ls)!)))*(1/(Gamma[(2*(ns+ms+ls)+3)/k+1]*Gamma[(2*(n-ns+m-ms+l-ls)+3)/k+1])),{ls, 0, l}],{ms, 0, m}],{ns, 0, n}], {n, 0, na},
{m, 0, ma},{l, 0, la}];
a1=((Gamma[3/k+1])^2)/((Gamma[1/k])^6);
```

```
a=1.0;
b=1.2;
c=1.5;
sigma=0.11;
z=(1-sigma*sigma)/(sigma*sigma);
q=10^lq;
qs=Sqrt[qx*qx+qy*qy];
Fqav=(a1/(2*Pi))*Sum[(Gamma[n+1/2]*((-q*q*a*a/4)^n))*Sum[(Gamma[m+1/2]*((-
q*q*b*b/4)^m))*Sum[(Gamma[l+1/2]*((-q*q*c*c/4)^l)/Gamma[n+m+l+3/2])*ccc[[n+1,m+1,l+1]], {l, 0, max}],{m, 0, max}],
{n , 0, max}];
Fqav1=(a1/(2*Pi))*Sum[(((-q*q/4)^n)/Gamma[n+3/2])*Sum[(Gamma[n-m+1/2]*(a^(2*(n-m))))*Sum[Gamma[m-
l+1/2]*(b^(2*(m-l)))*Gamma[l+1/2]*(c^(2*l))*ccc[[n-m+1,m-l+1,l+1]], {l, 0, m}],{m, 0, n}], {n , 0, max}];
Fqav1z=(a1/(2*Pi))*Sum[(((-q*q/(4*(z+1)*(z+1)))^n)/Gamma[n+3/2])*Sum[(Gamma[n-m+1/2]*(a^(2*(n-
m))))*Sum[Gamma[m-l+1/2]*(b^(2*(m-l)))*Gamma[l+1/2]*(c^(2*l))*fff[[n-m+1,m-l+1,l+1]], {l, 0, m}],{m, 0, n}], {n , 0,
max}];
Fqav1zs=(a1/(2*Pi))*Sum[(((-qs*qs/(4*(z+1)*(z+1)))^n)/Gamma[n+3/2])*Sum[(Gamma[n-m+1/2]*(a^(2*(n-
m))))*Sum[Gamma[m-l+1/2]*(b^(2*(m-l)))*Gamma[l+1/2]*(c^(2*l))*fff[[n-m+1,m-l+1,l+1]], {l, 0, m}],{m, 0, n}], {n , 0,
max}];
lim=2;
(* pl1=Plot[Log[10,Pqav], {lq , -1,lim1}, PlotRange{-4,1}, GridLines->{Automatic}, LabelStyle->Directive[Black,16],AxesLabel-
>{"log q","lg F^2(q)"}, AxesOrigin->{-1,-4},TicksStyle->Directive[Black,12],PlotStyle->{Black,Dashed}] ; *)
pl2=Plot[Log[10,Fqav1], {lq , -1,lim}, PlotRange->{-6,1}, GridLines->{Automatic}, LabelStyle->Directive[Black,16],AxesLabel-
>{"log q","lg F^2(q)"}, AxesOrigin->{-1,-6},TicksStyle->Directive[Black,12],PlotStyle->{Black}]
pl3=Plot[Log[10,Fqav1z], {lq , -1,lim}, PlotRange->{-6,1}, GridLines->{Automatic}, LabelStyle->Directive[Black,16],AxesLabel-
>{"log q","lg F^2(q)"}, AxesOrigin->{-1,-6},TicksStyle->Directive[Black,12],PlotStyle->{Blue}]
Show[pl2,pl3]
lims=9.0;
(* pl5=DensityPlot[Log[10,Pqavs],{qx, -lims, lims}, {qy, -lims, lims}, PlotRange->{-8,0}, PlotPoints->50, PlotLegends-
>Automatic, LabelStyleDirective[Black,12],AxesLabel->Automatic]*)
```

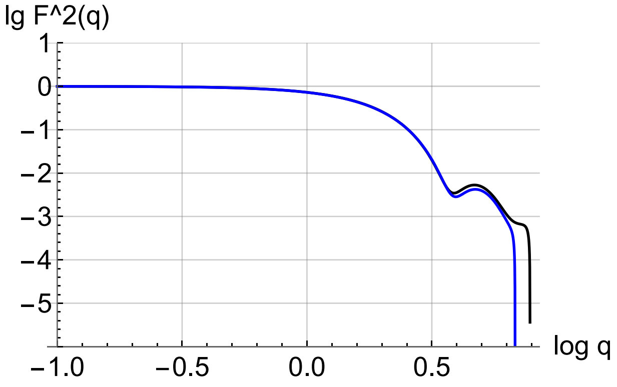

Fig. S32: Scattering amplitudes of a monodisperse and polydisperse isotropic supercubes.

4.9 Super-Ellipsoid

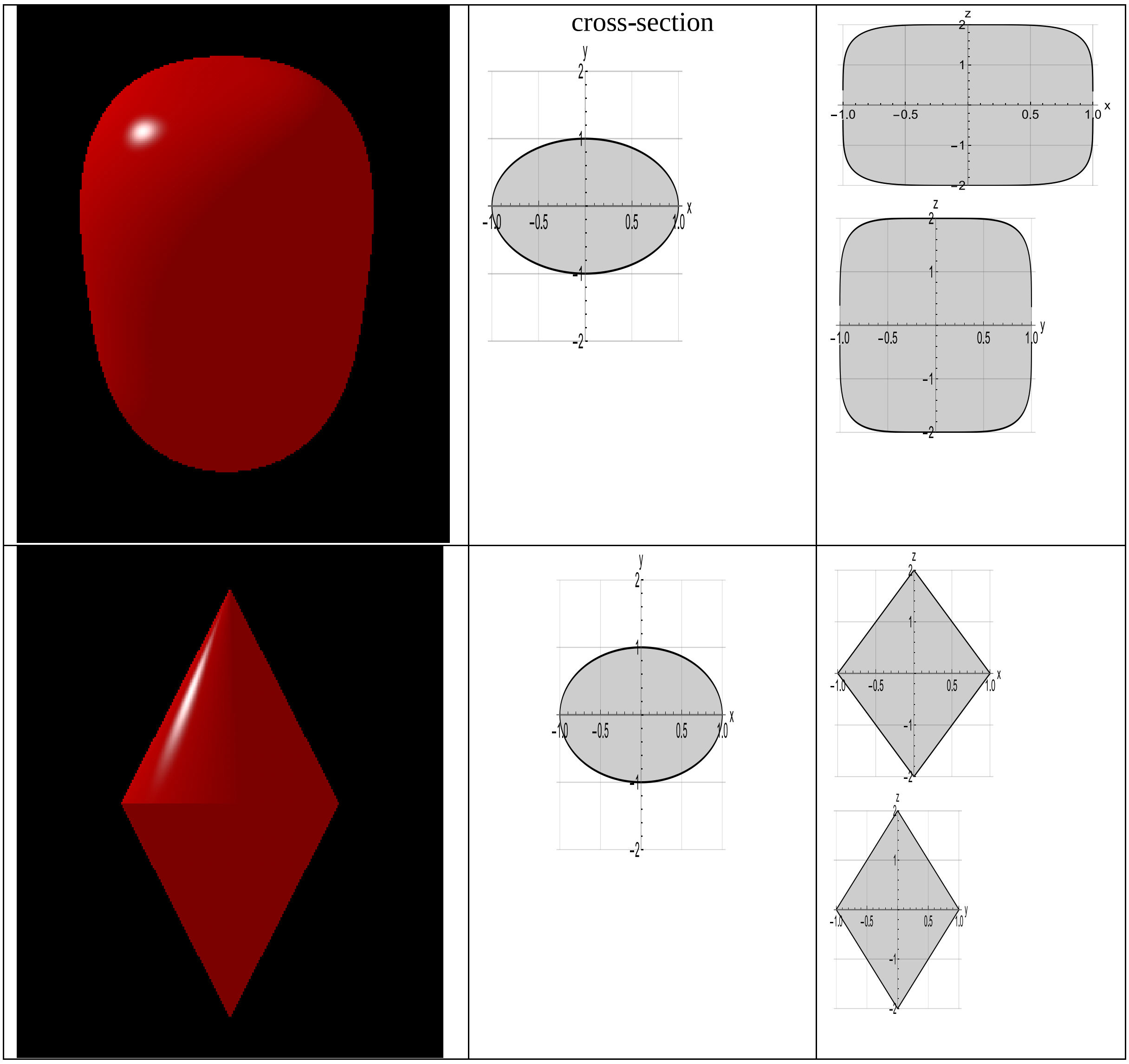

Fig. 33: Superellipsoid with cross-sections in the (x,y)-, (x,z)-, and (y,z)-planes.

4.9.1 Superellipsoidal rotation bodies

We consider rotation bodies that have a rotational symmetry with respect to their characteristic axis, e.g. the z-axis. Then the phase factor is independent of the angle ϕ. The phase factor can then be integrated over the volume of the half space $(0 \leq z \leq L)$ in cylindrical coordinates <u>**(S.4.9.1.1)**</u>

$$F(q) = \frac{1}{V}\int_0^{2\pi} d\phi \int_0^{L}\int_0^{R(z)} F^{(1)}(q_z z)F^{(2)}\big(q_r r(z)\big) r\,dr\,dz$$

$$= \frac{2\pi}{V} \int_0^L \int_0^{R(z)} \cos(q_z z) J_0\big(q_r r(z)\big) r dr dz$$

$$= \frac{2\pi}{V} \int_0^L \cos(q_z z) \int_0^{R(z)} J_0\big(q_r r(z)\big) r dr dz$$

$$= \frac{2\pi}{V} \int_0^L \cos(q_z z) \frac{R(z) J_1\big(q_r R(z)\big)}{q} dz$$

The volume is given by

$$V = \int_0^{2\pi} d\phi \int_0^L \int_0^{R(z)} r dr dz = 2\pi \int_0^L \frac{R^2(z)}{2} dz = \pi \int_0^L R^2(z) dz$$

The integral in Eq. (S.4.9.1.1) would need to be integrated numerically. Our approach is to again use hypergeometric functions as

$$\frac{R(z) J_1\big(q_r R(z)\big)}{q} = \frac{R^2(z)}{2} \; {}_0F_1\left(2; -\frac{q_r^2 R^2(z)}{4}\right)$$

$$\cos(q_z z) = \; {}_0F_1\left(\frac{1}{2}; -\frac{q_z^2 z^2}{4}\right)$$

Then the integral is **<u>(S.4.9.1.2)</u>**

$$F(q) = \frac{2\pi}{V} \int_0^L \cos(q_z z) \frac{R(z) J_1\big(q_r R(z)\big)}{q_r} dz$$

$$= \frac{2\pi}{V} \int_0^L {}_0F_1\left(\frac{1}{2}; -\frac{q_z^2 z^2}{4}\right) \frac{R^2(z)}{2} \; {}_0F_1\left(2; -\frac{q_r^2 R^2(z)}{4}\right) dz$$

$$= \frac{2\pi}{V} \int_0^L \sum_{n=0}^{\infty} \frac{1}{\left(\frac{1}{2}\right)_n n!} \left(-\frac{q_z^2 z^2}{4}\right)^n \frac{R^2(z)}{2} \sum_{m=0}^{\infty} \frac{1}{(2)_m m!} \left(-\frac{q^2 R^2(z)}{4}\right)^m dz$$

$$= \frac{\pi}{V} \sum_{n=0}^{\infty} \frac{1}{\left(\frac{1}{2}\right)_n n!} \left(-\frac{q_z^2}{4}\right)^n \sum_{m=0}^{\infty} \frac{1}{(2)_m m!} \left(-\frac{q_r^2}{4}\right)^m \int_0^L z^{2n} R^{2m+2}(z) dz$$

We assume the following radial dependence:

$$R(z) = R\left(1 - \left(\frac{z}{L}\right)^k\right)^{\frac{1}{k}}$$

Then the integral in Eq. (S.4.9.1.2) can be calculated analytically

$$\int_0^L z^{2n} R^{2m+2}(z)dz = \int_0^L z^{2n} R^{2m+2} \left(1 - \left(\frac{z}{L}\right)^k\right)^{\frac{2m+2}{k}} dz = L^{2n+1} R^{2m+2} \frac{\Gamma\left(\frac{2m+2+k}{k}\right)\Gamma\left(\frac{2n+1}{k}\right)}{k\Gamma\left(\frac{2m+2n+k+3}{k}\right)}$$

$$V = \pi \int_0^L R^2(z)dz = \pi \int_0^L R^2 \left(1 - \left(\frac{z}{L}\right)^k\right)^{\frac{2}{k}} dz = \pi L R^2 \frac{\Gamma\left(\frac{2+k}{k}\right)\Gamma\left(\frac{1}{k}\right)}{\mathrm{k}\Gamma\left(\frac{k+3}{k}\right)}$$

The volume equation reproduces the volume of a half sphere $V = \frac{2\pi}{3} R^3$ (L =R, k=2), the biaxial ellipsoid $V = \frac{2\pi}{3} L R^2$ ($k = 2$), the cylinder $V = \pi L R^2$ ($k \to \infty$) and the cone $V = \frac{\pi}{3} L R^2$ ($k = 1$). The integral and volume can be inserted into Eq. (S.4.9.1.2) to obtain

$$F(q) = \pi \sum_{n=0}^{\infty} \frac{1}{\left(\frac{1}{2}\right)_n n!} \left(-\frac{q_z^2}{4}\right)^n \sum_{m=0}^{\infty} \frac{1}{(2)_m m!} \left(-\frac{q_r^2}{4}\right)^m L^{2n+1} R^{2m+2} \frac{\Gamma\left(\frac{2m+2+k}{k}\right)\Gamma\left(\frac{2n+1}{k}\right)}{k\Gamma\left(\frac{2m+2n+k+3}{k}\right)} \frac{\mathrm{k}\Gamma\left(\frac{k+3}{k}\right)}{\pi L R^2 \Gamma\left(\frac{2+k}{k}\right)\Gamma\left(\frac{1}{k}\right)}$$

$$= \frac{\Gamma\left(\frac{k+3}{k}\right)}{\Gamma\left(\frac{2+k}{k}\right)\Gamma\left(\frac{1}{k}\right)} \sum_{n=0}^{\infty} \frac{1}{\left(\frac{1}{2}\right)_n n!} \left(-\frac{q_z^2 L^n}{4}\right)^n \sum_{m=0}^{\infty} \frac{1}{(2)_m m!} \left(-\frac{q_r^2 R^m}{4}\right)^m \frac{\Gamma\left(\frac{2m+2+k}{k}\right)\Gamma\left(\frac{2n+1}{k}\right)}{\Gamma\left(\frac{2m+2n+k+3}{k}\right)}$$

$$= \frac{\Gamma\left(\frac{k+3}{k}\right)}{\Gamma\left(\frac{2+k}{k}\right)\Gamma\left(\frac{1}{k}\right)} \sum_{n=0}^{\infty} \frac{\Gamma\left(\frac{2n+1}{k}\right)}{\left(\frac{1}{2}\right)_n n!} \left(-\frac{q_z^2 L^n}{4}\right)^n \sum_{m=0}^{\infty} \frac{1}{(2)_m m!} \left(-\frac{q_r^2 R^m}{4}\right)^m \frac{\Gamma\left(\frac{2m+2+k}{k}\right)}{\Gamma\left(\frac{2m+2n+k+3}{k}\right)}$$

This is then squared to obtain the squared scattering amplitude or the formfactor

$$P(q) = F^2(q) = \left(\frac{\Gamma\left(\frac{k+3}{k}\right)}{\Gamma\left(\frac{2+k}{k}\right)\Gamma\left(\frac{1}{k}\right)}\right)^2 \left(\sum_{n=0}^{\infty} \frac{\Gamma\left(\frac{2n+1}{k}\right)}{\left(\frac{1}{2}\right)_n n!} \left(-\frac{q_z^2 L^2}{4}\right)^n \sum_{m=0}^{\infty} \frac{1}{(2)_m m!} \left(-\frac{q_r^2 R^2}{4}\right)^m \frac{\Gamma\left(\frac{2m+2+k}{k}\right)}{\Gamma\left(\frac{2m+2n+k+3}{k}\right)}\right)$$

$$\left(\sum_{n'=0}^{\infty} \frac{\Gamma\left(\frac{2n'+1}{k}\right)}{\left(\frac{1}{2}\right)_{n'} n'!} \left(-\frac{q_z^2 L^2}{4}\right)^{n'} \sum_{m'=0}^{\infty} \frac{1}{(2)_{m'} m'!} \left(-\frac{q_r^2 R^2}{4}\right)^{m'} \frac{\Gamma\left(\frac{2m'+2+k}{k}\right)}{\Gamma\left(\frac{2m'+2n'+k+3}{k}\right)}\right)$$

$$= \left(\frac{\Gamma\left(\frac{k+3}{k}\right)}{\Gamma\left(\frac{2+k}{k}\right)\Gamma\left(\frac{1}{k}\right)}\right)^2 \left(\sum_{n=0}^{\infty}\sum_{m=0}^{\infty}\sum_{n'=0}^{\infty}\sum_{m'=0}^{\infty} \frac{\Gamma\left(\frac{2n+1}{k}\right)}{\left(\frac{1}{2}\right)_n n!} \left(-\frac{q_z^2 L^2}{4}\right)^n \frac{\Gamma\left(\frac{2n'+1}{k}\right)}{\left(\frac{1}{2}\right)_{n'} n'!} \left(-\frac{q_z^2 L^2}{4}\right)^{n'} \frac{1}{(2)_m m!} \left(-\frac{q_r^2 R^2}{4}\right)^m \frac{1}{(2)_{m'} m'!} \left(-\frac{q_r^2 R^2}{4}\right)^{m'} \frac{\Gamma\left(\frac{2m+2+k}{k}\right)}{\Gamma\left(\frac{2m+2n+k+3}{k}\right)} \frac{\Gamma\left(\frac{2m'+2+k}{k}\right)}{\Gamma\left(\frac{2m'+2n'+k+3}{k}\right)}\right)$$

$$(\ )$$

$$= \left(\frac{\Gamma\left(\frac{k+3}{k}\right)}{\Gamma\left(\frac{2+k}{k}\right)\Gamma\left(\frac{1}{k}\right)}\right)^2 \left(\sum_{n=0}^{\infty}\sum_{m=0}^{\infty}\sum_{n'=0}^{n}\sum_{m'=0}^{m} \frac{\Gamma\left(\frac{2(n-n')+1}{k}\right)}{\left(\frac{1}{2}\right)_{n-n'} (n-n')!} \left(-\frac{q_z^2 L^2}{4}\right)^{n-n'} \frac{\Gamma\left(\frac{2n'+1}{k}\right)}{\left(\frac{1}{2}\right)_{n'} n'!} \left(-\frac{q_z^2 L^2}{4}\right)^{n'} \frac{1}{(2)_{m-m'} (m-m')!} \left(-\frac{q_r^2 R^2}{4}\right)^{m-m'} \frac{1}{(2)_{m'} m'!} \left(-\frac{q_r^2 R^2}{4}\right)^{m'} \frac{\Gamma\left(\frac{2(m-m')+2+k}{k}\right)}{\Gamma\left(\frac{2(m-m')+2(n-n')+k+3}{k}\right)} \frac{\Gamma\left(\frac{2m'+2+k}{k}\right)}{\Gamma\left(\frac{2m'+2n'+k+3}{k}\right)}\right)$$

$$= \left(\frac{\Gamma\left(\frac{k+3}{k}\right)}{\Gamma\left(\frac{2+k}{k}\right)\Gamma\left(\frac{1}{k}\right)}\right)^2 \left(\sum_{n=0}^{\infty} \left(-\frac{q_z^2 L^2}{4}\right)^n \sum_{m=0}^{\infty} \left(-\frac{q_r^2 R^2}{4}\right)^m \sum_{n'=0}^{n} \frac{\Gamma\left(\frac{2(n-n')+1}{k}\right)}{\left(\frac{1}{2}\right)_{n-n'} (n-n')!} \frac{\Gamma\left(\frac{2n'+1}{k}\right)}{\left(\frac{1}{2}\right)_{n'} n'!} \sum_{m'=0}^{m} \frac{\Gamma\left(\frac{2(m-m')+2+k}{k}\right)}{(2)_{m-m'} (m-m')!} \frac{\Gamma\left(\frac{2m'+2+k}{k}\right)}{(2)_{m'} m'!} \frac{1}{\Gamma\left(\frac{2(m-m')+2(n-n')+k+3}{k}\right)\Gamma\left(\frac{2m'+2n'+k+3}{k}\right)}\right)$$

This can be written in a more compact form as **(S.4.9.1.3)**

$$P(q) = F^2(q) = \left(\frac{\Gamma\left(\frac{k+3}{k}\right)}{\Gamma\left(\frac{2+k}{k}\right)\Gamma\left(\frac{1}{k}\right)}\right)^2 \left(\sum_{n=0}^{\infty} \left(-\frac{q_z^2 L^2}{4}\right)^n \sum_{m=0}^{\infty} \left(-\frac{q_r^2 R^2}{4}\right)^m\right) c_{n,m}$$

$$c_{n,m}=\sum_{n'=0}^{n}\frac{\Gamma\left(\frac{2(n-n')+1}{k}\right)}{\left(\frac{1}{2}\right)_{n-n'}(n-n')!}\frac{\Gamma\left(\frac{2n'+1}{k}\right)}{\left(\frac{1}{2}\right)_{n'}n'!}\sum_{m'=0}^{m}\frac{\Gamma\left(\frac{2(m-m')+2+k}{k}\right)}{(2)_{m-m'}(m-m')!}\frac{\Gamma\left(\frac{2m'+2+k}{k}\right)}{(2)_{m'}m'!}\frac{1}{\Gamma\left(\frac{2(m-m')+2(n-n')+k+3}{k}\right)\Gamma\left(\frac{2m'+2n'+k+3}{k}\right)}$$

rotation body, monodisperse, oriented

```
ff=Table[Sum[(Gamma[(2*ns+1)/k]*Gamma[(2*(n-ns)+1)/k]/(Pochhammer[1/2,ns]*(ns!)*Pochhammer[1/2,n-ns]*((n-ns)!)))*
    Sum[(Gamma[(2*ms+2+k)/k]*Gamma[(2*(m-ms)+2+k)/k]/(Pochhammer[2,ms]*(ms!)*Pochhammer[2,m-ms]*((m-ms)!)))*(1/(Gamma[(2*(ns+ms)+k+3)/k]*Gamma[(2*(n-ns+m-ms)+k+3)/k])),{ms, 0, m}],{ns, 0, n}], {n, 0, na}, {m, 0, ma}];
a1=Gamma[(k+3)/k]/(Gamma[(2+k)/k]*Gamma[1/k]);
Pqav=a1*a1*Sum[(Gamma[n+1/2]*((-q*q*L*L/4)^n))*Sum[(Gamma[m+1]*((-q*q*R*R/4)^m))*ff[[n+1,m+1]]/(2*Gamma[n+m+3/2]), {m, 0, max}], {n , 0, max}]
```

The coefficient can be written in terms of Gamma functions

$$c_{n,m}=\sum_{n'=0}^{n}\frac{\Gamma\left(\frac{1}{2}\right)\Gamma\left(\frac{2(n-n')+1}{k}\right)}{\Gamma\left(n-n'+\frac{1}{2}\right)(n-n')!}\frac{\Gamma\left(\frac{1}{2}\right)\Gamma\left(\frac{2n'+1}{k}\right)}{\Gamma\left(n'+\frac{1}{2}\right)n'!}\sum_{m'=0}^{m}\frac{\Gamma(2)\Gamma\left(\frac{2(m-m')+2+k}{k}\right)}{\Gamma(m-m'+2)(m-m')!}\frac{\Gamma(2)\Gamma\left(\frac{2m'+2+k}{k}\right)}{\Gamma(m'+2)m'!}\frac{1}{\Gamma\left(\frac{2(m-m')+2(n-n')+k+3}{k}\right)\Gamma\left(\frac{2m'+2n'+k+3}{k}\right)}$$

$$=\pi\sum_{n'=0}^{n}\frac{\Gamma\left(\frac{2(n-n')+1}{k}\right)}{\Gamma\left(n-n'+\frac{1}{2}\right)(n-n')!}\frac{\Gamma\left(\frac{2n'+1}{k}\right)}{\Gamma\left(n'+\frac{1}{2}\right)n'!}\sum_{m'=0}^{m}\frac{\Gamma\left(\frac{2(m-m')+2+k}{k}\right)}{(m-m'+1)!(m-m')!}\frac{\Gamma\left(\frac{2m'+2+k}{k}\right)}{(m'+1)!m'!}\frac{1}{\Gamma\left(\frac{2(m-m')+2(n-n')+k+3}{k}\right)\Gamma\left(\frac{2m'+2n'+k+3}{k}\right)}$$

In the special case of $k=2$ (biaxial ellipsoid) we have

$$c_{n,m}=\pi\sum_{n'=0}^{n}\frac{\Gamma\left(\frac{2(n-n')+1}{2}\right)}{\Gamma\left(n-n'+\frac{1}{2}\right)(n-n')!}\frac{\Gamma\left(\frac{2n'+1}{2}\right)}{\Gamma\left(n'+\frac{1}{2}\right)n'!}\sum_{m'=0}^{m}\frac{\Gamma\left(\frac{2(m-m')+2+2}{2}\right)}{(m-m'+1)!(m-m')!}\frac{\Gamma\left(\frac{2m'+2+2}{2}\right)}{(m'+1)!m'!}\frac{1}{\Gamma\left(\frac{2(m-m')+2(n-n')+2+3}{2}\right)\Gamma\left(\frac{2m'+2n'+2+3}{2}\right)}$$

$$=\pi\sum_{n'=0}^{n}\frac{\Gamma\left(n-n'+\frac{1}{2}\right)}{\Gamma\left(n-n'+\frac{1}{2}\right)(n-n')!}\frac{\Gamma\left(n'+\frac{1}{2}\right)}{\Gamma\left(n'+\frac{1}{2}\right)n'!}\sum_{m'=0}^{m}\frac{\Gamma(m-m'+2)}{(m-m'+1)!(m-m')!}\frac{\Gamma(m'+2)}{(m'+1)!m'!}\frac{1}{\Gamma\left(m-m'+n-n'+\frac{5}{2}\right)\Gamma\left(m'+n'+\frac{5}{2}\right)}$$

$$=\pi\sum_{n'=0}^{n}\frac{1}{(n-n')!n'!}\sum_{m'=0}^{m}\frac{1}{(m-m')!m'!}\frac{1}{\Gamma\left(m-m'+n-n'+\frac{5}{2}\right)\Gamma\left(m'+n'+\frac{5}{2}\right)}$$

In the special case of $k=1$ (cone) we have

$$c_{n,m}=\pi\sum_{n'=0}^{n}\frac{\Gamma\left(\frac{2(n-n')+1}{1}\right)}{\Gamma\left(n-n'+\frac{1}{2}\right)(n-n')!}\frac{\Gamma\left(\frac{2n'+1}{1}\right)}{\Gamma\left(n'+\frac{1}{2}\right)n'!}\sum_{m'=0}^{m}\frac{\Gamma\left(\frac{2(m-m')+2+1}{1}\right)}{(m-m'+1)!(m-m')!}\frac{\Gamma\left(\frac{2m'+2+1}{1}\right)}{(m'+1)!m'!}\frac{1}{\Gamma\left(\frac{2(m-m')+2(n-n')+1+3}{1}\right)\Gamma\left(\frac{2m'+2n'+1+3}{1}\right)}$$

$$=\pi\sum_{n'=0}^{n}\frac{\Gamma(2(n-n')+1)}{\Gamma\left(n-n'+\frac{1}{2}\right)(n-n')!}\frac{\Gamma(2n'+1)}{\Gamma\left(n'+\frac{1}{2}\right)n'!}\sum_{m'=0}^{m}\frac{\Gamma(2(m-m')+3)}{(m-m'+1)!(m-m')!}\frac{\Gamma(2m'+3)}{(m'+1)!m'!}\frac{1}{\Gamma(2(m-m')+2(n-n')+4)\Gamma(2m'+2n'+4)}$$

$$=\pi\sum_{n'=0}^{n}\frac{(2(n-n'))!}{\Gamma\left(n-n'+\frac{1}{2}\right)(n-n')!}\frac{(2n')!}{\Gamma\left(n'+\frac{1}{2}\right)n'!}\sum_{m'=0}^{m}\frac{(2(m-m'+1))!}{(m-m'+1)!(m-m')!}\frac{(2(m'+1))!}{(m'+1)!m'!}\frac{1}{(2(m-m')+2(n-n')+3)!(2m'+2n'+3)!}$$

For polydisperse case we have

$$\langle F(q)\rangle=\frac{\Gamma\left(\frac{k+3}{k}\right)}{\Gamma\left(\frac{2+k}{k}\right)\Gamma\left(\frac{1}{k}\right)}\sum_{n=0}^{\infty}\frac{\Gamma\left(\frac{2n+1}{k}\right)(z+1)_{2n}}{\left(\frac{1}{2}\right)_n n!}\left(-\frac{q_z^2L^n}{4(z+1)^2}\right)^n\sum_{m=0}^{\infty}\frac{(z+1)_{2m}}{(2)_m m!}\left(-\frac{q_r^2R^2}{4(z+1)^2}\right)^m\frac{\Gamma\left(\frac{2m+2+k}{k}\right)}{\Gamma\left(\frac{2m+2n+k+3}{k}\right)}$$

The square is given by

$$\langle F(q)\rangle^2$$

$$=\left(\frac{\Gamma\left(\frac{k+3}{k}\right)}{\Gamma\left(\frac{2+k}{k}\right)\Gamma\left(\frac{1}{k}\right)}\right)^2\sum_{n=0}^{\infty}\frac{\Gamma\left(\frac{2n+1}{k}\right)(z+1)_{2n}}{\left(\frac{1}{2}\right)_n n!}\left(-\frac{q_z^2L^2}{4(z+1)^2}\right)^n\sum_{n'=0}^{\infty}\frac{\Gamma\left(\frac{2n'+1}{k}\right)(z+1)_{2n'}}{\left(\frac{1}{2}\right)_{n'} n'!}\left(-\frac{q_z^2L^2}{4(z+1)^2}\right)^{n'}\sum_{m=0}^{\infty}\frac{(z+1)_{2m}}{(2)_m m!}\left(-\frac{q_r^2R^2}{4(z+1)^2}\right)^m\frac{\Gamma\left(\frac{2m+2+k}{k}\right)}{\Gamma\left(\frac{2m+2n+k+3}{k}\right)}\sum_{m'=0}^{\infty}\frac{(z+1)_{2m'}}{(2)_{m'} m'!}\left(-\frac{q_r^2R^2}{4(z+1)^2}\right)^{m'}\frac{\Gamma\left(\frac{2m'+2+k}{k}\right)}{\Gamma\left(\frac{2m'+2n'+k+3}{k}\right)}$$

$$=\left(\frac{\Gamma\left(\frac{k+3}{k}\right)}{\Gamma\left(\frac{2+k}{k}\right)\Gamma\left(\frac{1}{k}\right)}\right)^2\sum_{n=0}^{\infty}\sum_{n'=0}^{n}\frac{\Gamma\left(\frac{2(n-n')+1}{k}\right)(z+1)_{2(n-n')}}{\left(\frac{1}{2}\right)_{n-n'}(n-n')!}\left(-\frac{q_z^2L^2}{4(z+1)^2}\right)^{n-n'}\frac{\Gamma\left(\frac{2n'+1}{k}\right)(z+1)_{2n'}}{\left(\frac{1}{2}\right)_{n'} n'!}\left(-\frac{q_z^2L^2}{4(z+1)^2}\right)^{n'}$$

$$\sum_{m=0}^{\infty}\sum_{m'=0}^{m}\frac{(z+1)_{2(m-m')}}{(2)_{m-m'}(m-m')!}\left(-\frac{q_r^2R^2}{4(z+1)^2}\right)^{m-m'}\frac{(z+1)_{2m'}}{(2)_{m'}m'!}\left(-\frac{q_r^2R^2}{4(z+1)^2}\right)^{m'}\frac{\Gamma\left(\frac{2(m-m')+2+k}{k}\right)}{\Gamma\left(\frac{2(m-m')+2(n-n')+k+3}{k}\right)}\frac{\Gamma\left(\frac{2m'+2+k}{k}\right)}{\Gamma\left(\frac{2m'+2n'+k+3}{k}\right)}$$

$$=\left(\frac{\Gamma\left(\frac{k+3}{k}\right)}{\Gamma\left(\frac{2+k}{k}\right)\Gamma\left(\frac{1}{k}\right)}\right)^2\sum_{n=0}^{\infty}\left(-\frac{q_z^2L^2}{4(z+1)^2}\right)^n\sum_{n'=0}^{n}\frac{\Gamma\left(\frac{2(n-n')+1}{k}\right)(z+1)_{2(n-n')}}{\left(\frac{1}{2}\right)_{n-n'}(n-n')!}\frac{\Gamma\left(\frac{2n'+1}{k}\right)(z+1)_{2n'}}{\left(\frac{1}{2}\right)_{n'}n'!}\sum_{m=0}^{\infty}\left(-\frac{q_r^2R^2}{4(z+1)^2}\right)^m\sum_{m'=0}^{m}\frac{(z+1)_{2(m-m')}}{(2)_{m-m'}(m-m')!}\frac{(z+1)_{2m'}}{(2)_{m'}m'!}\frac{\Gamma\left(\frac{2(m-m')+2+k}{k}\right)}{\Gamma\left(\frac{2(m-m')+2(n-n')+k+3}{k}\right)}\frac{\Gamma\left(\frac{2m'+2+k}{k}\right)}{\Gamma\left(\frac{2m'+2n'+k+3}{k}\right)}$$

It can be written in more compact form **<u>(S.4.9.1.4)</u>**

$$\langle F(q)\rangle^2=\left(\frac{\Gamma\left(\frac{k+3}{k}\right)}{\Gamma\left(\frac{2+k}{k}\right)\Gamma\left(\frac{1}{k}\right)}\right)^2\sum_{n=0}^{\infty}\left(-\frac{q_z^2L^2}{4(z+1)^2}\right)^n\sum_{m=0}^{\infty}\left(-\frac{q_r^2R^2}{4(z+1)^2}\right)^m f_{n,m}$$

$$f_{n,m}=\sum_{n'=0}^{n}\frac{\Gamma\left(\frac{2(n-n')+1}{k}\right)(z+1)_{2(n-n')}}{\left(\frac{1}{2}\right)_{n-n'}(n-n')!}\frac{\Gamma\left(\frac{2n'+1}{k}\right)(z+1)_{2n'}}{\left(\frac{1}{2}\right)_{n'}n'!}\sum_{m'=0}^{m}\frac{(z+1)_{2(m-m')}}{(2)_{m-m'}(m-m')!}\frac{(z+1)_{2m'}}{(2)_{m'}m'!}\frac{\Gamma\left(\frac{2(m-m')+2+k}{k}\right)}{\Gamma\left(\frac{2(m-m')+2(n-n')+k+3}{k}\right)}\frac{\Gamma\left(\frac{2m'+2+k}{k}\right)}{\Gamma\left(\frac{2m'+2n'+k+3}{k}\right)}$$

Rotation body, polydisperse, oriented, F(q)

For the formfactor we have **<u>(S.4.9.1.5)</u>**

$$\langle P(q)\rangle=\left(\frac{\Gamma\left(\frac{k+3}{k}\right)}{\Gamma\left(\frac{2+k}{k}\right)\Gamma\left(\frac{1}{k}\right)}\right)^2\sum_{n=0}^{\infty}(z+1)_{2n}\left(-\frac{q_z^2L^2}{4(z+1)^2}\right)^n\sum_{m=0}^{\infty}(z+1)_{2n}\left(-\frac{q_r^2R^2}{4(z+1)^2}\right)^m c_{n,m}$$

Rotation body, polydisperse, oriented, P(q)

Regime II. This needs numerical integration of $F(q)$

$$F(q)=\frac{2\pi}{V}\int_0^L\cos(q_z z)\frac{R(z)J_1\left(q_rR(z)\right)}{q}dz$$

and subsequent calculation of the square and the average over the size distribution and the polar angle. It is not needed, because Regime I overlaps with Regime III.

Regime III. The Porod asymptote in the case k=2 is **<u>(S.4.9.1.6)</u>**

$$\lim_{q\to\infty}P(q)=\frac{\Gamma(z-3)(z+1)^4}{\Gamma(z+1)}\frac{9}{2((q_rR)^2+(q_zL)^2)^{4/2}}$$

For the general case we can use the surface area of a corresponding rotation body, which is

$$R(z)=R\left(1-\left(\frac{z}{L}\right)^k\right)^{\frac{1}{k}}$$

$$\frac{\partial R(z)}{\partial z} = R_z = -\frac{R}{L}\left(\frac{z}{L}\right)^{k-1}\left(1-\left(\frac{z}{L}\right)^{k}\right)^{\frac{1}{k}-1}$$

$$DS = \sqrt{1+R_z^2}$$

$$A = 2\pi\int_0^L R(z) DS dz$$

Then the Porod asymptote is

$$P(q) = \frac{\Gamma[z+s-3](z+1)^4}{\Gamma[z+s+1]}\frac{2\pi A}{V^2 q^4}$$

For the orientational average we obtain **(S.4.9.1.7)**

$$\langle P(q)\rangle = \langle F(q)\rangle^2 = \left(\frac{\Gamma\left(\frac{k+3}{k}\right)}{\Gamma\left(\frac{2+k}{k}\right)\Gamma\left(\frac{1}{k}\right)}\right)^2 \sum_{n=0}^{\infty}\left(-\frac{q^2L^2}{4}\right)^n \sum_{m=0}^{\infty}\left(-\frac{q^2R^2}{4}\right)^m c_{n,m}\int_0^{\pi/2}(\cos\theta)^{2n}(\sin\theta)^{2m}\sin\theta\, d\,\theta$$

$$= \left(\frac{\Gamma\left(\frac{k+3}{k}\right)}{\Gamma\left(\frac{2+k}{k}\right)\Gamma\left(\frac{1}{k}\right)}\right)^2 \sum_{n=0}^{\infty}\left(-\frac{q^2L^2}{4}\right)^n \sum_{m=0}^{\infty}\left(-\frac{q^2R^2}{4}\right)^m c_{n,m}\frac{\Gamma(m+1)\Gamma\left(n+\frac{1}{2}\right)}{2\Gamma\left(m+n+\frac{3}{2}\right)}$$

$$= \left(\frac{\Gamma\left(\frac{k+3}{k}\right)}{\Gamma\left(\frac{2+k}{k}\right)\Gamma\left(\frac{1}{k}\right)}\right)^2 \sum_{n=0}^{\infty}\Gamma\left(n+\frac{1}{2}\right)\left(-\frac{q^2L^2}{4}\right)^n \sum_{m=0}^{\infty}\Gamma(m+1)\left(-\frac{q^2R^2}{4}\right)^m \frac{c_{n,m}}{2\Gamma\left(m+n+\frac{3}{2}\right)}$$

$$= \left(\frac{\Gamma\left(\frac{k+3}{k}\right)}{\Gamma\left(\frac{2+k}{k}\right)\Gamma\left(\frac{1}{k}\right)}\right)^2 \sum_{n=0}^{\infty}\Gamma\left(n+\frac{1}{2}\right)\left(-\frac{q^2L^2}{4}\right)^n \sum_{m=0}^{\infty}(m!)\left(-\frac{q^2R^2}{4}\right)^m \frac{c_{n,m}}{2\Gamma\left(m+n+\frac{3}{2}\right)}$$

Rotation body, monodisperse, isotropic, F(q), P(q)

It can be written in more compact form as

$$\langle P(q)\rangle = \left(\frac{\Gamma\left(\frac{k+3}{k}\right)}{\Gamma\left(\frac{2+k}{k}\right)\Gamma\left(\frac{1}{k}\right)}\right)^2 \sum_{n=0}^{\infty}\frac{1}{2\Gamma\left(n+\frac{3}{2}\right)}\left(-\frac{q^2}{4}\right)^n \sum_{m=0}^{n}\Gamma\left(n-m+\frac{1}{2}\right)(L^2)^{n-m}(m!)R^{2m}c_{n-m,m}$$

In the polydisperse case we have **(S.4.9.1.8)**

$$\langle F(q)\rangle^2 = \left(\frac{\Gamma\left(\frac{k+3}{k}\right)}{\Gamma\left(\frac{2+k}{k}\right)\Gamma\left(\frac{1}{k}\right)}\right)^2 \sum_{n=0}^{\infty}\left(-\frac{q^2L^2}{4(z+1)^2}\right)^n \sum_{m=0}^{\infty}\left(-\frac{q^2R^2}{4(z+1)^2}\right)^m f_{n,m}\frac{\Gamma(m+1)\Gamma\left(n+\frac{1}{2}\right)}{2\Gamma\left(m+n+\frac{3}{2}\right)}$$

$$= \left(\frac{\Gamma\left(\frac{k+3}{k}\right)}{\Gamma\left(\frac{2+k}{k}\right)\Gamma\left(\frac{1}{k}\right)}\right)^2 \sum_{n=0}^{\infty}\Gamma\left(n+\frac{1}{2}\right)\left(-\frac{q^2L^2}{4(z+1)^2}\right)^n \sum_{m=0}^{\infty}m!\left(-\frac{q^2R^2}{4(z+1)^2}\right)^m f_{n,m}\frac{1}{2\Gamma\left(m+n+\frac{3}{2}\right)}$$

Rotation body, polydisperse, isotropic

For the formfactor we have **(S.4.9.1.9)**

$$\langle P(q)\rangle = \left(\frac{\Gamma\left(\frac{k+3}{k}\right)}{\Gamma\left(\frac{2+k}{k}\right)\Gamma\left(\frac{1}{k}\right)}\right)^2 \sum_{n=0}^{\infty}(z+1)_{2n}\Gamma\left(n+\frac{1}{2}\right)\left(-\frac{q^2L^2}{4(z+1)^2}\right)^n \sum_{m=0}^{\infty}(m!)(z+1)_{2m}\left(-\frac{q^2R^2}{4(z+1)^2}\right)^m \frac{c_{n,m}}{2\Gamma\left(m+n+\frac{3}{2}\right)}$$

Rotation body, polydisperse, isotropic

For faster evaluation it can be rewritten using Cauchy summation

$$\langle P(q)\rangle = \left(\frac{\Gamma\left(\frac{k+3}{k}\right)}{\Gamma\left(\frac{2+k}{k}\right)\Gamma\left(\frac{1}{k}\right)}\right)^2 \sum_{n=0}^{\infty}\sum_{m=0}^{n}(z+1)_{2(n-m)}\Gamma\left(n-m+\frac{1}{2}\right)\left(-\frac{q^2L^2}{4(z+1)^2}\right)^{n-m}(m!)(z+1)_{2m}\left(-\frac{q^2R^2}{4(z+1)^2}\right)^m \frac{c_{n-m,m}}{2\Gamma\left(m+n-m+\frac{3}{2}\right)}$$

$$= \left(\frac{\Gamma\left(\frac{k+3}{k}\right)}{\Gamma\left(\frac{2+k}{k}\right)\Gamma\left(\frac{1}{k}\right)}\right)^2 \frac{1}{2}\sum_{n=0}^{\infty}\frac{1}{\Gamma\left(n+\frac{3}{2}\right)}\left(-\frac{q^2}{4(z+1)^2}\right)^n \sum_{m=0}^{n}(z+1)_{2(n-m)}\Gamma\left(n-m+\frac{1}{2}\right)L^{2(n-m)}(m!)(z+1)_{2m}R^{2m}\,c_{n-m,m}$$

Mathematica code implementation:

```
k=2.3;
na=40;
ma=na;
ff=Table[Sum[(Gamma[(2*ns+1)/k]*Gamma[(2*(n-ns)+1)/k]/(Pochhammer[1/2,ns]*(ns!)*Pochhammer[1/2,n-ns]*((n-
ns)!)))*Sum[(Gamma[(2*ms+2+k)/k]*Gamma[(2*(m-ms)+2+k)/k]/(Pochhammer[2,ms]*(ms!)*Pochhammer[2,m-ms]*((m-
ms)!)))*(1/(Gamma[(2*(ns+ms)+k+3)/k]*Gamma[(2*(n-ns+m-ms)+k+3)/k])),{ms,0,m}],{ns,0,n}],{n,0,na},{m,0,ma}];
```

```
R=1;
L=1.5;
sigma=0.1;
q=10^lq;
qs=Sqrt[qx*qx+qy*qy];
z=(1-sigma*sigma)/(sigma*sigma);
vol=2*Pi*R*R*L*Gamma[(2+k)/k]*Gamma[1/k]/(k*Gamma[(k+3)/k]);
zzl=-(R/L)*((zz/L)^(k-1))*(1-((zz/L)^k))^((1/k)-1);
DS=Sqrt[1+zzl*zzl];
zzz=R*(1-((zz/L)^k))^(1/k);
area=4*Pi*NIntegrate[DS*zzz,{zz,0,L}];
a1=Gamma[(k+3)/k]/(Gamma[(2+k)/k]*Gamma[1/k]);
apor=Gamma[z-3]*((z+1)^4)/Gamma[z+1];
Pqav=a1*a1*Sum[Gamma[n+1/2]*((-q*q*L*L/4)^n)*Sum[(m!)*((-
q*q*R*R/4)^m)*ff[[n+1,m+1]]/(2*Gamma[m+n+3/2]),{m,0,ma}],{n,0,na}];
Pqavz=a1*a1*Sum[(Gamma[n+1/2]*Pochhammer[z+1,2*n]*((-
q*q*L*L/(4*(z+1)*(z+1)))^n))*Sum[(m!)*Pochhammer[z+1,2*m]*(((-
q*q*R*R/(4*(z+1)*(z+1)))^m))*ff[[n+1,m+1]]/(2*Gamma[m+n+3/2]),{m,0,ma}],{n,0,na}];
```

```
Pqavz1=(a1*a1/2)*Sum[(((-q*q/(4*(z+1)*(z+1)))^n)/Gamma[n+3/2])*Sum[Gamma[n-m+1/2]*Pochhammer[z+1,2*(n-m)]*(m!)*Pochhammer[z+1,2*m]*(L^(2*(n-m)))*(R^(2*m))*ff[[n-m+1,m+1]],{m,0,n}],{n,0,na}];
Pqavzs=a1*a1*Sum[(Gamma[n+1/2]*Pochhammer[z+1,2*n]*((-qs*qs*L*L/(4*(z+1)*(z+1)))^n))*Sum[(m!)*Pochhammer[z+1,2*m]*(((-qs*qs*R*R/(4*(z+1)*(z+1)))^m))*ff[[n+1,m+1]]/(2*Gamma[m+n+3/2]),{m,0,ma}],{n,0,na}];
Pqpor=apor*2*Pi*area/((q^4)*vol*vol);
lim=1.5;
pl1=Plot[Log[10,Pqav], {lq , -1,lim}, PlotRange->{-6,1}, GridLines->{Automatic}, LabelStyle->Directive[Black,16],AxesLabel->{"log q","lg P(q)"}, AxesOrigin->{-1,-6},TicksStyle->Directive[Black,12],PlotStyle->{Black}] ;
pl2=Plot[Log[10,Pqavz], {lq , -1,lim}, PlotRange->{-6,1}, GridLines->{Automatic}, LabelStyle->Directive[Black,16],AxesLabel->{"log q","lg P(q)"}, AxesOrigin->{-1,-6},TicksStyle->Directive[Black,12],PlotStyle->{Blue}]  ;
pl3=Plot[Log[10,Pqpor], {lq , -1,lim}, PlotRange->{-6,1}, GridLines->{Automatic}, LabelStyle->Directive[Black,16],AxesLabel->{"log q","lg P(q)"}, AxesOrigin->{-1,-6},TicksStyle->Directive[Black,12],PlotStyle->{Red}];
Show[pl2,pl3]
lims=10;
pl5=DensityPlot[Log[10,Pqavzs],{qx, -lims, lims}, {qy, -lims, lims}, PlotRange->{-8,0}, PlotPoints->50, PlotLegends->Automatic, LabelStyle->Directive[Black,12],AxesLabel->Automatic]
```

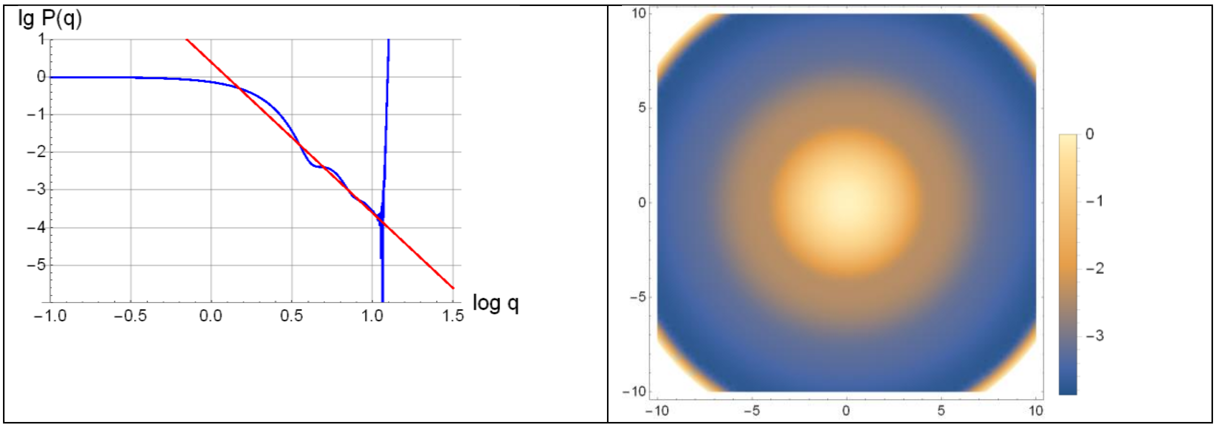

Fig. S34: Formfactor of polydisperse isotropic rotation bodies with circular cross-section.

Scattering Amplitude F(q)

Regime I For the scattering amplitude in the polydisperse case we use Eq. (S.4.9.1.8)

$$\langle F(q)\rangle^2=\left(\frac{\Gamma\left(\frac{k+3}{k}\right)}{\Gamma\left(\frac{2+k}{k}\right)\Gamma\left(\frac{1}{k}\right)}\right)^2\sum_{n=0}^{\infty}\Gamma\left(n+\frac{1}{2}\right)\left(-\frac{q^2L^2}{4(z+1)^2}\right)^n\sum_{m=0}^{\infty}m!\left(-\frac{q^2R^2}{4(z+1)^2}\right)^m f_{n,m}\frac{1}{2\Gamma\left(m+n+\frac{3}{2}\right)}$$

Regime II and III. Here only numerical integration schemes are possible.

Mathematica code implementation:

```
k=2.3;
na=25;
ma=na;
cc=Table[Sum[(Gamma[(2*ns+1)/k]*Gamma[(2*(n-ns)+1)/k]/(Pochhammer[1/2,ns]*(ns!)*Pochhammer[1/2,n-ns]*((n-ns)!)))*Sum[(Gamma[(2*ms+2+k)/k]*Gamma[(2*(m-ms)+2+k)/k]/(Pochhammer[2,ms]*(ms!)*Pochhammer[2,m-ms]*((m-ms)!)))*(1/(Gamma[(2*(ns+ms)+k+3)/k]*Gamma[(2*(n-ns+m-ms)+k+3)/k])),{ms,0,m}],{ns,0,n}],{n,0,na},{m,0,ma}];
ff=Table[Sum[(Gamma[(2*ns+1)/k]*Gamma[(2*(n-ns)+1)/k]*Pochhammer[z+1,2*(n-ns)]*Pochhammer[z+1,2*ns]/(Pochhammer[1/2,ns]*(ns!)*Pochhammer[1/2,n-ns]*((n-ns)!)))*Sum[(Gamma[(2*ms+2+k)/k]*Gamma[(2*(m-ms)+2+k)/k]*Pochhammer[z+1,2*(m-ms)]*Pochhammer[z+1,2*ms]/(Pochhammer[2,ms]*(ms!)*Pochhammer[2,m-ms]*((m-ms)!)))*(1/(Gamma[(2*(ns+ms)+k+3)/k]*Gamma[(2*(n-ns+m-ms)+k+3)/k])),{ms,0,m}],{ns,0,n}],{n,0,na},{m,0,ma}];

R=1;
L=1.5;
sigma=0.1;
q=10^lq;
qs=Sqrt[qx*qx+qy*qy];
z=(1-sigma*sigma)/(sigma*sigma);
a1=Gamma[(k+3)/k]/(Gamma[(2+k)/k]*Gamma[1/k]);
Fqav=a1*a1*Sum[Gamma[n+1/2]*((-q*q*L*L/4)^n)*Sum[(m!)*((-q*q*R*R/4)^m)*cc[[n+1,m+1]]/(2*Gamma[m+n+3/2]),{m,0,ma}],{n,0,na}];
Fqavz=a1*a1*Sum[(Gamma[n+1/2]*((-q*q*L*L/(4*(z+1)*(z+1)))^n))*Sum[(m!)*(((-q*q*R*R/(4*(z+1)*(z+1)))^m))*ff[[n+1,m+1]]/(2*Gamma[m+n+3/2]),{m,0,ma}],{n,0,na}];
Fqavzs=a1*a1*Sum[(Gamma[n+1/2]*((-qs*qs*L*L/(4*(z+1)*(z+1)))^n))*Sum[(m!)*(((-qs*qs*R*R/(4*(z+1)*(z+1)))^m))*ff[[n+1,m+1]]/(2*Gamma[m+n+3/2]),{m,0,ma}],{n,0,na}];
lim=1.5;
pl1=Plot[Log[10,Fqav], {lq , -1,lim}, PlotRange->{-6,1}, GridLines->{Automatic}, LabelStyle->Directive[Black,16],AxesLabel->{"log q","lg F^2(q)"}, AxesOrigin->{-1,-6},TicksStyle->Directive[Black,12],PlotStyle->{Black}] ;
pl2=Plot[Log[10,Fqavz], {lq , -1,lim}, PlotRange->{-6,1}, GridLines->{Automatic}, LabelStyle->Directive[Black,16],AxesLabel->{"log q","lg F^2(q)"}, AxesOrigin->{-1,-6},TicksStyle->Directive[Black,12],PlotStyle->{Blue}]  ;
Show[pl1,pl2]
lims=10;
pl5=DensityPlot[Log[10,Fqavzs],{qx, -lims, lims}, {qy, -lims, lims}, PlotRange->{-8,0}, PlotPoints->50, PlotLegends->Automatic, LabelStyle->Directive[Black,12],AxesLabel->Automatic]
```

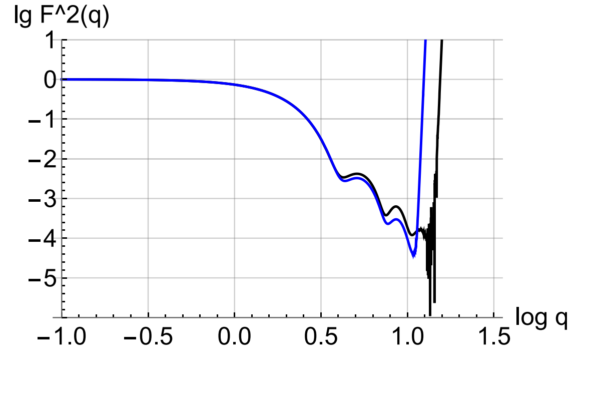

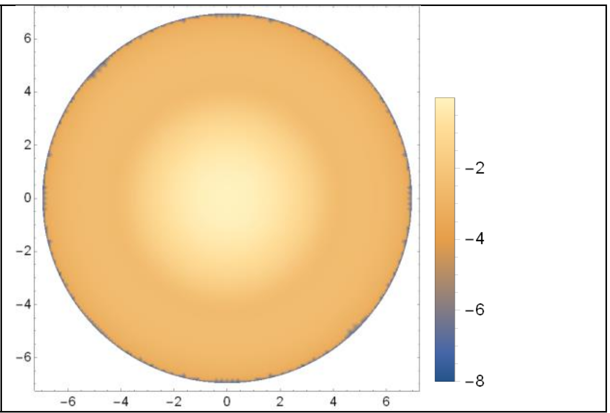

Fig. S35: Scattering amplitude of monodisperse and polydisperse isotropic rotation bodies with circular cross-section.

4.9.2 Superellipsoid-shaped lenses and dumbbells

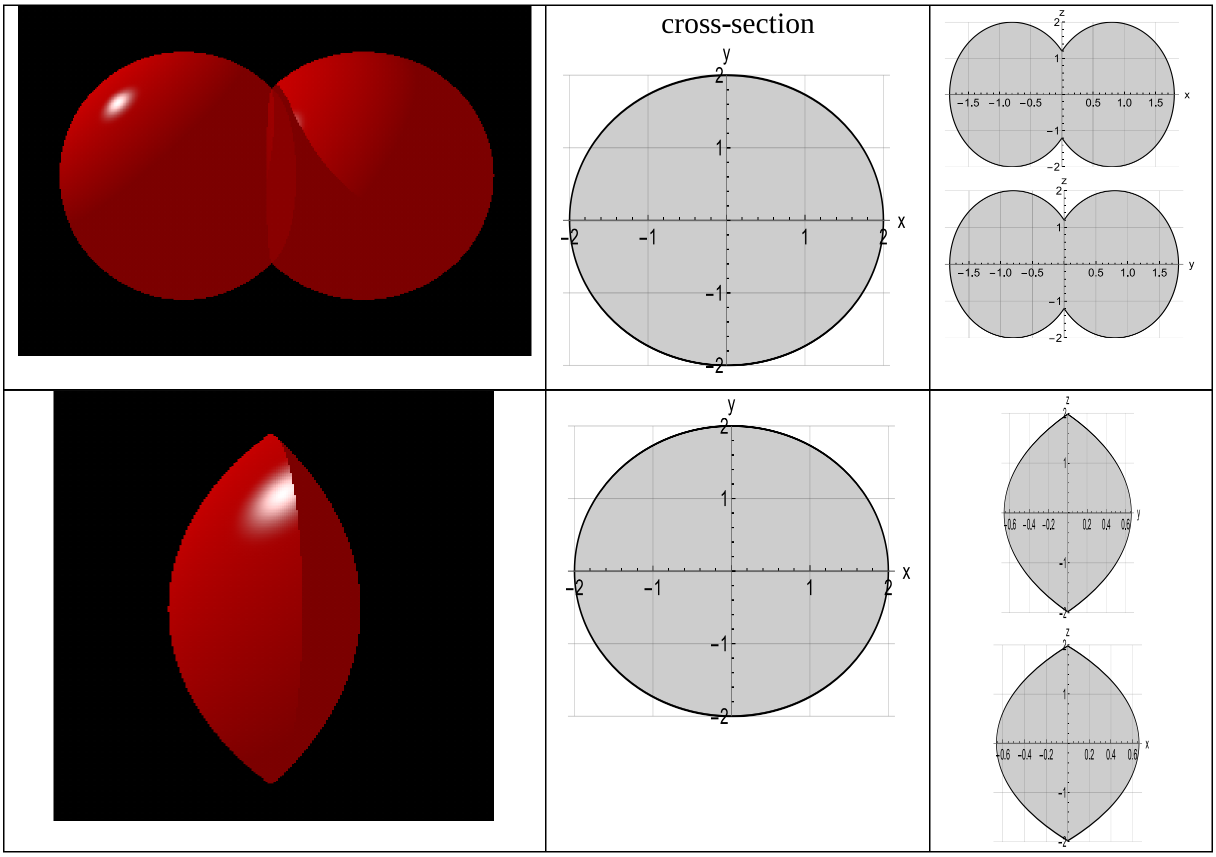

Fig. S36: Dumbbell and lens with cross-sections in the (*x*,*y*)-, (*x*,*z*)-, and (*y*,*z*)-planes.

We can extend the superellipsoid model to shift the z-coordinate to $z \to z - \alpha L$ with $-1 \leq \alpha \leq 1$ to include lens- ($\alpha < 0$) and dumbbell-shaped ($\alpha > 0$) objects. Then the integral is **(S.4.9.2.1)**

$$F(q) = \frac{\pi}{V} \sum_{n=0}^{\infty} \frac{1}{\left(\frac{1}{2}\right)_n n!} \left(-\frac{q_z^2}{4}\right)^n \sum_{m=0}^{\infty} \frac{1}{(2)_m m!} \left(-\frac{q_r^2}{4}\right)^m \int_0^{L(1+\alpha)} z^{2n} R^{2m+2}(z) dz$$

We assume the following radial dependence:

$$R(z) = R\left(1 - \left|\frac{z}{L} - \alpha\right|^k\right)^{\frac{1}{k}}$$

Then the integral in Eq. (S.4.9.2.1) can be calculated numerically

$$\int_0^{L+\alpha L} z^{2n} R^{2m+2}(z) dz = \int_0^{L(1+\alpha)} z^{2n} R^{2m+2} \left(1 - \left|\frac{z}{L} - \alpha\right|^k\right)^{\frac{2m+2}{k}} dz =$$

$$= R^{2m+2}T_{n,m}$$

$$T_{n,m} = \int_0^{L(1+\alpha)} z^{2n}\left(1-\left|\frac{z}{L}-\alpha\right|^k\right)^{\frac{2m+2}{k}} dz$$

$$V = \pi \int_0^{L(1+\alpha)} R^2(z)dz = \pi R^2 \int_0^{L(1+\alpha)} \left(1-\left|\frac{z}{L}-\alpha\right|^k\right)^{\frac{2}{k}} dz = \pi R^2 T_{0,0}$$

The integral and volume can be inserted into Eq. (S.4.9.2.1 to obtain

$$F(q) = \pi \sum_{n=0}^{\infty} \frac{1}{\left(\frac{1}{2}\right)_n n!}\left(-\frac{q_z^2}{4}\right)^n \sum_{m=0}^{\infty} \frac{1}{(2)_m m!}\left(-\frac{q_r^2}{4}\right)^m \frac{R^{2m+2}T_{n,m}}{\pi R^2 T_{0,0}}$$

$$= \frac{1}{T_{0,0}} \sum_{n=0}^{\infty} \frac{1}{\left(\frac{1}{2}\right)_n n!}\left(-\frac{q_z^2}{4}\right)^n \sum_{m=0}^{\infty} \frac{1}{(2)_m m!}\left(-\frac{q_r^2 R^2}{4}\right)^m T_{n,m}$$

This is then squared to obtain the square d scattering amplitude or the formfactor

$$P(q) = F^2(q) = \frac{1}{T_{0,0}^2}\left(\sum_{n=0}^{\infty} \frac{1}{\left(\frac{1}{2}\right)_n n!}\left(-\frac{q_z^2}{4}\right)^n \sum_{m=0}^{\infty} \frac{1}{(2)_m m!}\left(-\frac{q_r^2 R^2}{4}\right)^m T_{n,m}\right)$$

$$\left(\sum_{n'=0}^{\infty} \frac{1}{\left(\frac{1}{2}\right)_{n'} n'!}\left(-\frac{q_z^2}{4}\right)^{n'} \sum_{m'=0}^{\infty} \frac{1}{(2)_{m'} m'!}\left(-\frac{q_r^2 R^2}{4}\right)^{m'} T_{n',m'}\right)$$

$$= \frac{1}{T_{0,0}^2}\left(\sum_{n=0}^{\infty}\sum_{m=0}^{\infty}\sum_{n'=0}^{\infty}\sum_{m'=0}^{\infty} \frac{1}{\left(\frac{1}{2}\right)_n n!}\left(-\frac{q_z^2}{4}\right)^n \frac{1}{\left(\frac{1}{2}\right)_{n'} n'!}\left(-\frac{q_z^2}{4}\right)^{n'} \frac{1}{(2)_m m!}\left(-\frac{q_r^2 R^2}{4}\right)^m \frac{1}{(2)_{m'} m'!}\left(-\frac{q_r^2 R^2}{4}\right)^{m'} T_{n,m}T_{n',m'}\right)$$

( )

$$= \frac{1}{T_{0,0}^2}\left(\sum_{n=0}^{\infty}\sum_{m=0}^{\infty}\sum_{n'=0}^{n}\sum_{m'=0}^{m} \frac{1}{\left(\frac{1}{2}\right)_{n-n'} (n-n')!}\left(-\frac{q_z^2}{4}\right)^{n-n'} \frac{1}{\left(\frac{1}{2}\right)_{n'} n'!}\left(-\frac{q_z^2}{4}\right)^{n'} \frac{1}{(2)_{m-m'} (m-m')!}\left(-\frac{q_r^2 R^2}{4}\right)^{m-m'} \frac{1}{(2)_{m'} m'!}\left(-\frac{q_r^2 R^2}{4}\right)^{m'} T_{n-n',m-m'}T_{n',m'}\right)$$

$$= \frac{1}{T_{0,0}^2}\left(\sum_{n=0}^{\infty}\left(-\frac{q_z^2}{4}\right)^n \sum_{m=0}^{\infty}\left(-\frac{q_r^2 R^2}{4}\right)^m \sum_{n'=0}^{n} \frac{1}{\left(\frac{1}{2}\right)_{n-n'} (n-n')! \left(\frac{1}{2}\right)_{n'} n'!} \sum_{m'=0}^{m} \frac{1}{(2)_{m-m'} (m-m')! (2)_{m'} m'!} T_{n-n',m-m'}T_{n',m'}\right)$$

This can be written in a more compact form as **(S.4.9.2.2)**

$$P(q) = F^2(q) = \frac{1}{T_{0,0}^2} \sum_{n=0}^{\infty}\left(-\frac{q_z^2}{4}\right)^n \sum_{m=0}^{\infty}\left(-\frac{q_r^2 R^2}{4}\right)^m t_{n,m}$$

$$t_{n,m} = \sum_{n'=0}^{n} \frac{1}{\left(\frac{1}{2}\right)_{n-n'} (n-n')! \left(\frac{1}{2}\right)_{n'} n'!} \sum_{m'=0}^{m} \frac{1}{(2)_{m-m'} (m-m')! (2)_{m'} m'!} T_{n-n',m-m'}T_{n',m'}$$

Lens and dumbbell, monodisperse, oriented

For polydisperse case we have

$$\langle F(q)\rangle = \frac{1}{T_{0,0}}\sum_{n=0}^{\infty}\frac{(z+1)_{2n}}{\left(\frac{1}{2}\right)_n n!}\left(-\frac{q_z^2}{4(z+1)^2}\right)^n \sum_{m=0}^{\infty}\frac{(z+1)_{2m}}{(2)_m m!}\left(-\frac{q_r^2R^2}{4(z+1)^2}\right)^m T_{n,m}$$

The square is given by

$$\langle F(q)\rangle^2 = \frac{1}{T_{0,0}^2}\sum_{n=0}^{\infty}\frac{(z+1)_{2n}}{\left(\frac{1}{2}\right)_n n!}\left(-\frac{q_z^2}{4(z+1)^2}\right)^n \sum_{n'=0}^{\infty}\frac{(z+1)_{2n'}}{\left(\frac{1}{2}\right)_{n'} n'!}\left(-\frac{q_z^2}{4(z+1)^2}\right)^{n'} \sum_{m=0}^{\infty}\frac{(z+1)_{2m}}{(2)_m m!}\left(-\frac{q_r^2R^2}{4(z+1)^2}\right)^m \sum_{m'=0}^{\infty}\frac{(z+1)_{2m'}}{(2)_{m'} m'!}\left(-\frac{q_r^2R^2}{4(z+1)^2}\right)^{m'} T_{n,m}T_{n',m'}$$

$$= \frac{1}{T_{0,0}^2}\sum_{n=0}^{\infty}\sum_{n'=0}^{n}\frac{(z+1)_{2(n-n')}}{\left(\frac{1}{2}\right)_{n-n'}(n-n')!}\left(-\frac{q_z^2}{4(z+1)^2}\right)^{n-n'}\frac{(z+1)_{2n'}}{\left(\frac{1}{2}\right)_{n'} n'!}\left(-\frac{q_z^2}{4(z+1)^2}\right)^{n'}$$

$$\sum_{m=0}^{\infty}\sum_{m'=0}^{m}\frac{(z+1)_{2(m-m')}}{(2)_{m-m'}(m-m')!}\left(-\frac{q_r^2R^2}{4(z+1)^2}\right)^{m-m'}\frac{(z+1)_{2m'}}{(2)_{m'} m'!}\left(-\frac{q_r^2R^2}{4(z+1)^2}\right)^{m'} T_{n-n',m-m'}T_{n',m'}$$

$$= \frac{1}{T_{0,0}^2}\sum_{n=0}^{\infty}\left(-\frac{q_z^2}{4(z+1)^2}\right)^n \sum_{n'=0}^{n}\frac{(z+1)_{2(n-n')}}{\left(\frac{1}{2}\right)_{n-n'}(n-n')!}\frac{(z+1)_{2n'}}{\left(\frac{1}{2}\right)_{n'} n'!}\sum_{m=0}^{\infty}\left(-\frac{q_r^2R^2}{4(z+1)^2}\right)^m \sum_{m'=0}^{m}\frac{(z+1)_{2(m-m')}}{(2)_{m-m'}(m-m')!}\frac{(z+1)_{2m'}}{(2)_{m'} m'!}T_{n-n',m-m'}T_{n',m'}$$

It can be written in more compact form **(S.4.9.2.3)**

$$\langle F(q)\rangle^2 = \frac{1}{T_{0,0}^2}\sum_{n=0}^{\infty}\left(-\frac{q_z^2L^2}{4(z+1)^2}\right)^n \sum_{m=0}^{\infty}\left(-\frac{q_r^2R^2}{4(z+1)^2}\right)^m f_{n,m}$$

$$f_{n,m} = \sum_{n'=0}^{n}\frac{(z+1)_{2(n-n')}}{\left(\frac{1}{2}\right)_{n-n'}(n-n')!}\frac{(z+1)_{2n'}}{\left(\frac{1}{2}\right)_{n'} n'!}\sum_{m'=0}^{m}\frac{(z+1)_{2(m-m')}}{(2)_{m-m'}(m-m')!}\frac{(z+1)_{2m'}}{(2)_{m'} m'!}T_{n-n',m-m'}T_{n',m'}$$

Lens and dumbbell, polydisperse, oriented, F(q)

For the formfactor we have **(S.4.9.2.4)**

$$\langle P(q)\rangle = \frac{1}{T_{0,0}^2}\sum_{n=0}^{\infty}(z+1)_{2n}\left(-\frac{q_z^2}{4(z+1)^2}\right)^n \sum_{m=0}^{\infty}(z+1)_{2n}\left(-\frac{q_r^2R^2}{4(z+1)^2}\right)^m t_{n,m}$$

Lens and dumbbell, polydisperse, oriented, P(q)

For the orientational average we obtain **(S.4.9.2.6)**

$$F(q)^2 = \frac{1}{T_{0,0}^2}\sum_{n=0}^{\infty}\left(-\frac{q^2}{4}\right)^n \sum_{m=0}^{\infty}\left(-\frac{q^2R^2}{4}\right)^m t_{n,m}\int_0^{\pi/2}(\cos\theta)^{2n}(\sin\theta)^{2m}\sin\theta\, d\,\theta$$

$$= \frac{1}{T_{0,0}^2}\sum_{n=0}^{\infty}\left(-\frac{q^2}{4}\right)^n \sum_{m=0}^{\infty}\left(-\frac{q^2R^2}{4}\right)^m t_{n,m}\frac{\Gamma(m+1)\Gamma\left(n+\frac{1}{2}\right)}{2\Gamma\left(m+n+\frac{3}{2}\right)}$$

$$= \frac{1}{T_{0,0}^2}\sum_{n=0}^{\infty}\Gamma\left(n+\frac{1}{2}\right)\left(-\frac{q^2}{4}\right)^n \sum_{m=0}^{\infty}\Gamma(m+1)\left(-\frac{q^2R^2}{4}\right)^m \frac{t_{n,m}}{2\Gamma\left(m+n+\frac{3}{2}\right)}$$

$$= \frac{1}{2T_{0,0}^2}\sum_{n=0}^{\infty}\Gamma\left(n+\frac{1}{2}\right)\left(-\frac{q^2}{4}\right)^n \sum_{m=0}^{\infty}(m!)\left(-\frac{q^2R^2}{4}\right)^m \frac{t_{n,m}}{\Gamma\left(m+n+\frac{3}{2}\right)}$$

Lens and dumbbell, monodisperse, isotropic, F(q), P(q)

It can be written in more compact form as

$$\langle P(q)\rangle = \frac{1}{T_{0,0}^2}\sum_{n=0}^{\infty}\frac{1}{2\Gamma\left(n+\frac{3}{2}\right)}\left(-\frac{q^2}{4}\right)^n\sum_{m=0}^{n}\Gamma\left(n-m+\frac{1}{2}\right)(m!)R^{2m}t_{n-m,m}$$

In the polydisperse case we have **(S.4.9.2.7)**

$$\langle F(q)\rangle^2 = \frac{1}{T_{0,0}^2}\sum_{n=0}^{\infty}\left(-\frac{q^2}{4(z+1)^2}\right)^n\sum_{m=0}^{\infty}\left(-\frac{q^2R^2}{4(z+1)^2}\right)^m f_{n,m}\frac{\Gamma(m+1)\Gamma\left(n+\frac{1}{2}\right)}{2\Gamma\left(m+n+\frac{3}{2}\right)}$$

$$= \frac{1}{T_{0,0}^2}\sum_{n=0}^{\infty}\Gamma\left(n+\frac{1}{2}\right)\left(-\frac{q^2}{4(z+1)^2}\right)^n\sum_{m=0}^{\infty}m!\left(-\frac{q^2R^2}{4(z+1)^2}\right)^m f_{n,m}\frac{1}{2\Gamma\left(m+n+\frac{3}{2}\right)}$$

Lens and dumbbell, polydisperse, isotropic, F(q)

For the formfactor we have **(S.4.9.2.8)**

$$\langle P(q)\rangle = \frac{1}{2T_{0,0}^2}\sum_{n=0}^{\infty}(z+1)_{2n}\Gamma\left(n+\frac{1}{2}\right)\left(-\frac{q^2}{4(z+1)^2}\right)^n\sum_{m=0}^{\infty}(m!)(z+1)_{2m}\left(-\frac{q^2R^2}{4(z+1)^2}\right)^m\frac{t_{n,m}}{\Gamma\left(m+n+\frac{3}{2}\right)}$$

Lens and dumbbell, polydisperse, isotropic, P(q)

For faster evaluation it can be rewritten using Cauchy summation

$$\langle P(q)\rangle = \frac{1}{2T_{0,0}^2}\sum_{n=0}^{\infty}\sum_{m=0}^{n}(z+1)_{2(n-m)}\Gamma\left(n-m+\frac{1}{2}\right)\left(-\frac{q^2}{4(z+1)^2}\right)^{n-m}(m!)(z+1)_{2m}\left(-\frac{q^2R^2}{4(z+1)^2}\right)^m\frac{t_{n-m,m}}{\Gamma\left(m+n-m+\frac{3}{2}\right)}$$

$$= \frac{1}{2T_{0,0}^2}\sum_{n=0}^{\infty}\frac{1}{\Gamma\left(n+\frac{3}{2}\right)}\left(-\frac{q^2}{4(z+1)^2}\right)^n\sum_{m=0}^{n}(z+1)_{2(n-m)}\Gamma\left(n-m+\frac{1}{2}\right)(m!)(z+1)_{2m}R^{2m}\,t_{n-m,m}$$

Regime II. In cases, where Regime I does not overlap with Regime III, the scattering amplitude is calculated from the integral

$$F(q) = \frac{2\pi}{V}\int_0^{L(1+\alpha)}\cos(q_z z)\frac{R(z)J_1\left(q_r R(z)\right)}{q_r}dz$$

Then the formfactor is obtained from $P(q) = F^2(q)$, averaged over the azimuthal angle θ, and over the size distribution.

Regime III. We can use the surface area of a corresponding rotation body, which is

$$R(z) = R\left(1 - \left|\frac{z}{L} - \alpha\right|^k\right)^{\frac{1}{k}}$$

$$\frac{\partial R(z)}{\partial z} = R_z = -\frac{R}{L}\left(\left|\frac{z}{L} - \alpha\right|^k\right)^{k-1}\left(1 - \left|\frac{z}{L} - \alpha\right|^k\right)^{\frac{1}{k}-1}$$

$$DS = \sqrt{1 + R_z^2}$$

$$A = 2\pi \int_0^L R(z) DS dz$$

Then the Porod asymptote is **(S.4.9.2.5)**

$$P(q) = \frac{\Gamma[z + s - 3](z + 1)^4}{\Gamma[z + s + 1]} \frac{2\pi A}{V^2 q^4}$$

Mathematica code implementation:

```
L=2.5;
R=1.5;
a=-0.7;
k=2.0;
sigma=0.1;
zs=(1-sigma*sigma)/(sigma*sigma);
nmax=15;
qs=10^lqs;
q=10^lq;
phi=0.3*Pi/2;
qz=qs*Cos[phi];
qr=qs*Sin[phi];
rzz=(-R/L)*((Abs[(zz/L)-a])^(k-1))*((1-((Abs[(zz/L)-a])^k))^((1/k)-1));
rz=R*(1-((Abs[(zz/L)-a])^k))^(1/k);
DS=Sqrt[1+rzz*rzz];
area=2*Pi*NIntegrate[rz*DS, {zz, 0, L*(1+a)}];
arear=N[2*Pi*R*R];
vol=Pi*NIntegrate[rz*rz, {zz, 0, L*(1+a)}];
volr=N[2*Pi*R*R*R/3];
rzu=R*((1-((Abs[(Abs[zu]/L)-a])^k))^(1/k));
pl0a=Plot[rzu, {zu, -5,5}, PlotRange->{-2,2}, LabelStyle->Directive[Black,16],GridLines-
>{Automatic},AxesLabel->{"z","r"}, AxesOrigin->{0,0},TicksStyle-
>Directive[Black,12],PlotStyle->{Black}, Filling->Axis] ;
pl0b=Plot[-rzu, {zu, -5,5}, PlotRange->{-2,2}, LabelStyle->Directive[Black,16],GridLines-
>{Automatic},AxesLabel->{"z","r"}, AxesOrigin->{0,0},TicksStyle-
>Directive[Black,12],PlotStyle->{Black}, Filling->Axis] ;
Show[pl0a,pl0b]
pre=Gamma[(k+3)/k]/(Gamma[(2+k)/k]*Gamma[1/k]);
pht1=Table[1/(Pochhammer[1/2,n]*(n!)), {n, 0, nmax}];
pht2=Table[1/(Pochhammer[2,n]*(n!)), {n, 0, nmax}];
cct=Table[NIntegrate[(z^(2*nn))*((1-((Abs[(z/L)-a])^k))^((2*mm+2)/k)),{z, 0, L*(1+a)}],{mm,
0,nmax},{nn, 0, nmax}] ;
ff=Table[Sum[(Gamma[(2*ns+1)/k]*Gamma[(2*(n-
ns)+1)/k]/(Pochhammer[1/2,ns]*(ns!)*Pochhammer[1/2,n-ns]*((n-
ns)!)))*Sum[(Gamma[(2*ms+2+k)/k]*Gamma[(2*(m-
ms)+2+k)/k]/(Pochhammer[2,ms]*(ms!)*Pochhammer[2,m-ms]*((m-
```

```
ms)!)))*(1/(Gamma[(2*(ns+ms)+k+3)/k]*Gamma[(2*(n-ns+m-
ms)+k+3)/k])),{ms,0,m}],{ns,0,n}],{n,0,nmax},{m,0,nmax}];
tt=Table[Sum[(pht1[[ns+1]]*pht1[[n-ns+1]])*Sum[(cct[[ms+1,ns+1]]*cct[[m-ms+1,n-
ns+1]]*pht2[[ms+1]]*pht2[[m-ms+1]]),{ms,0,m}],{ns,0,n}],{n,0,nmax},{m,0,nmax}];
(* general *)
Pq1=(1/(cct[[1,1]]*cct[[1,1]]))*Sum[(Pochhammer[zs+1,2*n]*((-
qz*qz/(4*((zs+1)^2)))^n))*Sum[Pochhammer[zs+1,2*m]*((-
qr*qr*R*R/(4*((zs+1)^2)))^m)*tt[[n+1,m+1]], {m, 0, nmax}], {n, 0, nmax}];
Pq1a=(1/(cct[[1,1]]*cct[[1,1]]))*Sum[(Pochhammer[zs+1,2*n]*((-
qzz*qzz/(4*((zs+1)^2)))^n))*Sum[Pochhammer[zs+1,2*m]*((-
qrr*qrr*R*R/(4*((zs+1)^2)))^m)*tt[[n+1,m+1]], {m, 0, nmax}], {n, 0, nmax}];
Rq1z=(1/(2*cct[[1,1]]*cct[[1,1]]))*Sum[(Pochhammer[zs+1,2*n]*((-
q*q/(4*((zs+1)^2)))^n)/Gamma[n+3/2])*Sum[(Gamma[n-m+1/2]*(m!)*(R*R)^m)*tt[[n-m+1,m+1]], {m,
0, n}], {n, 0, nmax}];
(* a=0 case *)
Pq2=pre*pre*Sum[(Pochhammer[zs+1,2*n]*((-
qz*qz*L*L/(4*((zs+1)^2)))^n))*Sum[Pochhammer[zs+1,2*m]*((-
qr*qr*R*R/(4*((zs+1)^2)))^m)*ff[[n+1,m+1]], {m, 0, nmax}], {n, 0, nmax}];
Pq2a=pre*pre*Sum[(Pochhammer[zs+1,2*n]*((-
qzz*qzz*L*L/(4*((zs+1)^2)))^n))*Sum[Pochhammer[zs+1,2*m]*((-
qrr*qrr*R*R/(4*((zs+1)^2)))^m)*ff[[n+1,m+1]], {m, 0, nmax}], {n, 0, nmax}];
Rq2z=(pre*pre/2)*Sum[(Pochhammer[zs+1,2*n]*((-
q*q/(4*((zs+1)^2)))^n)/Gamma[n+3/2])*Sum[Gamma[n-m+1/2]*(m!)*((L*L)^(n-m))*((R*R)^m)*ff[[n-
m+1,m+1]], {m, 0, n}], {n, 0, nmax}];
(* sphere limit *)
Psphz=Sum[(6*(4^n)*Pochhammer[zs+1,2*n]*((-
q*q*R*R/(4*((zs+1)^2)))^n)/((n+3)*(n+2)*Pochhammer[5/2,n]*(n!))), {n, 0, nmax}];
fact=((zs+1)^4)/(zs*(zs-1)*(zs-2)*(zs-3));
Pqpor=fact*2*Pi*area/(vol*vol*(q^4));
Pqpors=fact*2*Pi*arear/(volr*volr*(q^4));
lim=1;
pl1=Plot[Log[10,Pq1], {lqs, -1,lim}, PlotRange->{-6,1}, GridLines->{Automatic}, LabelStyle-
>Directive[Black,16],AxesLabel->{"log q","lg P(q)"}, AxesOrigin->{-1,-6},TicksStyle-
>Directive[Black,12],PlotStyle->{Black}] ;
pl2=Plot[Log[10,Pq2], {lqs , -1,lim}, PlotRange->{-6,1}, GridLines->{Automatic},
LabelStyle->Directive[Black,16],AxesLabel->{"log q","lg P(q)"}, AxesOrigin->{-1,-
6},TicksStyle->Directive[Black,12],PlotStyle->{Blue}] ;
Show[pl1,pl2]
pl3=Plot[Log[10,Rq1z], {lq , -1,lim}, PlotRange->{-6,1}, GridLines->{Automatic},
LabelStyle->Directive[Black,16],AxesLabel->{"log q","lg P(q)"}, AxesOrigin->{-1,-
6},TicksStyle->Directive[Black,12],PlotStyle->{Black}] ;
pl4=Plot[Log[10,Rq2z], {lq , -1,lim}, PlotRange->{-6,1}, GridLines->{Automatic},
LabelStyle->Directive[Black,16],AxesLabel->{"log q","lg P(q)"}, AxesOrigin->{-1,-
6},TicksStyle->Directive[Black,12],PlotStyle->{Blue}] ;
pl5=Plot[Log[10,Psphz], {lq , -1,lim}, PlotRange->{-6,1}, GridLines->{Automatic},
LabelStyle->Directive[Black,16],AxesLabel->{"log q","lg P(q)"}, AxesOrigin->{-1,-
6},TicksStyle->Directive[Black,12],PlotStyle->{Red}] ;
pl6=Plot[Log[10,Pqpor], {lq , -1,lim}, PlotRange->{-6,1}, GridLines->{Automatic},
LabelStyle->Directive[Black,16],AxesLabel->{"log q","lg P(q)"}, AxesOrigin->{-1,-
6},TicksStyle->Directive[Black,12],PlotStyle->{Black,Dashed}] ;
pl7=Plot[Log[10,Pqpors], {lq , -1,lim}, PlotRange->{-6,1}, GridLines->{Automatic},
LabelStyle->Directive[Black,16],AxesLabel->{"log q","lg P(q)"}, AxesOrigin->{-1,-
6},TicksStyle->Directive[Black,12],PlotStyle->{Red,Dashed}] ;
Show[pl3,pl4,pl5,pl6,pl7]
Show[pl3,pl4,pl6]
Show[pl3,pl6]
limq=5;
pl20=DensityPlot[Log[10,Pq1a],{qzz, -limq, limq}, {qrr, -limq, limq}, PlotRange->{-8,0},
PlotPoints->50, PlotLegends->Automatic, LabelStyle->Directive[Black,12],AxesLabel-
>Automatic]
pl21=DensityPlot[Log[10,Pq2a],{qzz, -limq, limq}, {qrr, -limq, limq}, PlotRange->{-8,0},
PlotPoints->50, PlotLegends->Automatic, LabelStyle->Directive[Black,12],AxesLabel-
>Automatic]
```

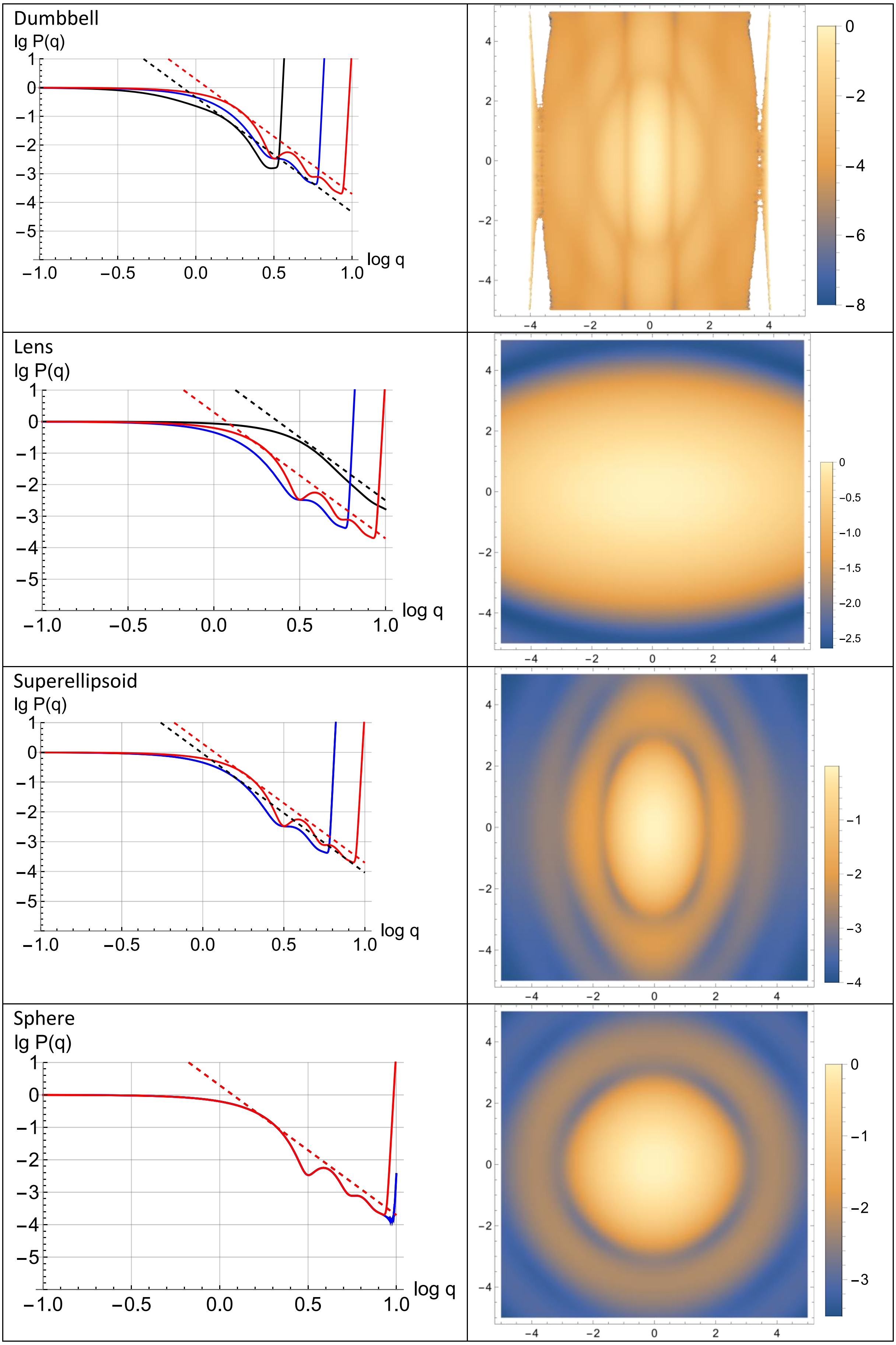
Dumbbell
lg P(q)
log q
Lens
lg P(q)
log q
Superellipsoid
lg P(q)
log q
Sphere
lg P(q)
log q

Fig. S37: Formfactor of polydisperse dumbbells (black), lenses (black), ellipsoids (blue) and spheres (red) with circular cross-section.

4.9.3 Superellipsoid with ellipsoidal cross-section

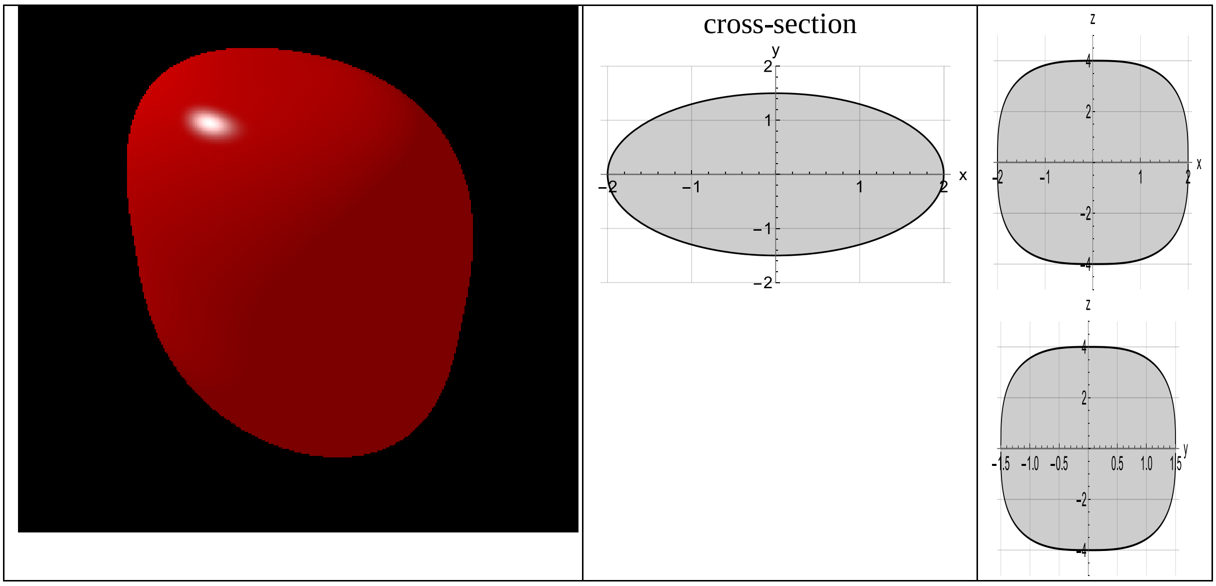

Fig. S38: Superellipsoid and lens with cross-sections in the (*x*,*y*)-, (*x*,*z*)-, and (*y*,*z*)-planes.

This is a special case of the general superellipsoid

$$\left(\left(\frac{x}{a}\right)^r + \left(\frac{y}{c}\right)^r\right)^{\frac{k}{r}} + \left(\frac{z}{L}\right)^k = 1$$

for $r = 2$. We consider $R(\phi)$ as for an ellipsoidal cross-section and use

$$R(\phi) = ((a\sin\phi)^2 + (c\cos\phi)^2)^{\frac{1}{2}} = (a^2(\sin\phi)^2 + a^2(\varepsilon\cos\phi)^2)^{\frac{1}{2}}$$

$$= a((\sin\phi)^2 + \varepsilon^2(\cos\phi)^2)^{1/2}$$

with $\varepsilon = c/a$. Then we can integrate $R(\phi)^{2n}$ over the angle ϕ

$$\langle R(\phi)^{2n}\rangle = \frac{2a^{2n}}{\pi}\int_0^{\frac{\pi}{2}} ((\sin\phi)^2 + \varepsilon^2(\cos\phi)^2)^n d\phi = \frac{2a^{2n}}{\pi}\sum_{m=0}^{n}\binom{n}{m}(\varepsilon^2)^{n-m}\int_0^{\frac{\pi}{2}} (\sin\phi)^{2m}(\cos\phi)^{2(n-m)} d\phi$$

$$= \frac{2a^{2n}}{\pi}\sum_{m=0}^{n}\binom{n}{m}(\varepsilon^2)^{n-m}\frac{\Gamma\left(n-m+\frac{1}{2}\right)\Gamma\left(m+\frac{1}{2}\right)}{2\Gamma(n+1)} = \frac{2a^{2n}}{\pi}\sum_{m=0}^{n}\frac{n!}{(n-m)!\,m!}(\varepsilon^2)^{n-m}\frac{\Gamma\left(n-m+\frac{1}{2}\right)\Gamma\left(m+\frac{1}{2}\right)}{2n!}$$

$$= \frac{a^{2n}}{\pi}\sum_{m=0}^{n}\frac{(\varepsilon^2)^{n-m}}{(n-m)!\,m!}\Gamma\left(n-m+\frac{1}{2}\right)\Gamma\left(m+\frac{1}{2}\right) = a^{2n}e_n$$

$$e_n = \frac{1}{\pi}\sum_{m=0}^{n}\frac{(\varepsilon^2)^{n-m}}{(n-m)!\,m!}\Gamma\left(n-m+\frac{1}{2}\right)\Gamma\left(m+\frac{1}{2}\right)$$

Regime I. We can use Eq. (S.4.9.1.8) for the monodisperse oriented rotation body to insert the integral **(S.4.9.3.1)**

$$P(q) = F^2(q) = \left(\frac{\Gamma\left(\frac{k+3}{k}\right)}{\Gamma\left(\frac{2+k}{k}\right)\Gamma\left(\frac{1}{k}\right)}\right)^2 \sum_{n=0}^{\infty}\left(-\frac{q_z^2L^2}{4}\right)^n \sum_{m=0}^{\infty}\left(-\frac{q_r^2R(\phi)^2}{4}\right)^m c_{n,m}$$

$$= \left(\frac{\Gamma\left(\frac{k+3}{k}\right)}{\Gamma\left(\frac{2+k}{k}\right)\Gamma\left(\frac{1}{k}\right)}\right)^2 \sum_{n=0}^{\infty}\left(-\frac{q_z^2L^2}{4}\right)^n \sum_{m=0}^{\infty}\left(-\frac{q_r^2a^2}{4}\right)^m e_m\, c_{n,m}$$

Rotation body, elliptical, monodisperse, oriented, F(q), P(q)

Similarly, we can use Eq. (S.4.9.1.9) for the polydisperse oriented rotation body to obtain for the scattering amplitude **(S.4.9.3.2)**

$$\langle F(q)\rangle^2 = \left(\frac{\Gamma\left(\frac{k+3}{k}\right)}{\Gamma\left(\frac{2+k}{k}\right)\Gamma\left(\frac{1}{k}\right)}\right)^2 \sum_{n=0}^{\infty}\left(-\frac{q_z^2L^2}{4(z+1)^2}\right)^n \sum_{m=0}^{\infty}\left(-\frac{q_r^2a^2}{4(z+1)^2}\right)^m e_m f_{n,m}$$

and for the formfactor **(S.4.9.3.3)**

$$\langle P(q)\rangle = \left(\frac{\Gamma\left(\frac{k+3}{k}\right)}{\Gamma\left(\frac{2+k}{k}\right)\Gamma\left(\frac{1}{k}\right)}\right)^2 \sum_{n=0}^{\infty}(z+1)_{2n}\left(-\frac{q_z^2L^2}{4(z+1)^2}\right)^n \sum_{m=0}^{\infty}(z+1)_{2n}\left(-\frac{q_r^2a^2}{4(z+1)^2}\right)^m e_m c_{n,m}$$

Rotation body, elliptical, polydisperse, oriented, P(q)

For the monodisperse isotropic case we have **(S.4.9.3.4)**

$$\langle P(q)\rangle = F^2(q) = \left(\frac{\Gamma\left(\frac{k+3}{k}\right)}{\Gamma\left(\frac{2+k}{k}\right)\Gamma\left(\frac{1}{k}\right)}\right)^2 \sum_{n=0}^{\infty}\Gamma\left(n+\frac{1}{2}\right)\left(-\frac{q^2L^2}{4}\right)^n \sum_{m=0}^{\infty}(m!)\left(-\frac{q^2a^2}{4}\right)^m \frac{e_m c_{n,m}}{2\Gamma\left(m+n+\frac{3}{2}\right)}$$

$$= \left(\frac{\Gamma\left(\frac{k+3}{k}\right)}{\Gamma\left(\frac{2+k}{k}\right)\Gamma\left(\frac{1}{k}\right)}\right)^2 \sum_{n=0}^{\infty}\frac{1}{2\Gamma\left(n+\frac{3}{2}\right)}\left(-\frac{q^2}{4}\right)^n \sum_{m=0}^{n}\Gamma\left(n-m+\frac{1}{2}\right)(L^2)^{n-m}(m!)a^{2m}\, e_m\, c_{n-m,m}$$

Rotation body, elliptical, monodisperse, isotropic, F(q), P(q)

In the polydisperse cases we have for the scattering amplitude **(S.4.9.3.5)**

$$\langle F(q)\rangle^2 = \left(\frac{\Gamma\left(\frac{k+3}{k}\right)}{\Gamma\left(\frac{2+k}{k}\right)\Gamma\left(\frac{1}{k}\right)}\right)^2 \sum_{n=0}^{\infty} \Gamma\left(n+\frac{1}{2}\right)\left(-\frac{q^2L^2}{4(z+1)^2}\right)^n \sum_{m=0}^{\infty} m!\left(-\frac{q^2a^2}{4(z+1)^2}\right)^m \frac{e_m f_{n,m}}{2\Gamma\left(m+n+\frac{3}{2}\right)}$$

Rotation body, elliptical, polydisperse, isotropic, F(q)

For the formfactor we have **<u>(S.4.9.3.6)</u>**

$$\langle P(q)\rangle = \left(\frac{\Gamma\left(\frac{k+3}{k}\right)}{\Gamma\left(\frac{2+k}{k}\right)\Gamma\left(\frac{1}{k}\right)}\right)^2 \sum_{n=0}^{\infty} (z+1)_{2n}\Gamma\left(n+\frac{1}{2}\right)\left(-\frac{q^2L^2}{4(z+1)^2}\right)^n \sum_{m=0}^{\infty} (m!)(z+1)_{2m}\left(-\frac{q^2a^2}{4(z+1)^2}\right)^m \frac{e_m c_{n,m}}{2\Gamma\left(m+n+\frac{3}{2}\right)}$$

Rotation body, elliptical, polydisperse, isotropic, P(q)

Regime II. This can only be computed by numerical integration schemes.

Regime III. For the oriented case only in the case of $k = 2$ we have the expression for ellipsoids as **<u>(S.4.9.3.7)</u>**

$$P(q) = \frac{9}{2q^4H^4}$$

$$H = \left((a\sin\theta\cos\phi)^2 + (b\sin\theta\sin\phi)^2 + (c\cos\theta)^2\right)^{1/2}$$

For the Porod-asymptote in the isotropic case we need the volume and the surface area of a rotation body. Assuming a radius a(=R) and a (half) long axis c(=L), we have the surface area

$$\left(\left(\frac{x}{a}\right)^2 + \left(\frac{y}{c}\right)^2\right)^{\frac{k}{2}} + \left(\frac{z}{L}\right)^k = 1$$

We then have

$$z = L\left(1 - \left(\left(\frac{x}{a}\right)^2 + \left(\frac{y}{c}\right)^2\right)^{\frac{k}{2}}\right)^{\frac{1}{k}}$$

with the partial derivatives

$$z_x = \frac{\partial z}{\partial x} = -\frac{Lx\left(\left(\frac{x}{a}\right)^2 + \left(\frac{y}{c}\right)^2\right)^{\frac{k}{2}-1}\left(1 - \left(\left(\frac{x}{a}\right)^2 + \left(\frac{y}{c}\right)^2\right)^{\frac{k}{2}}\right)^{\frac{1}{k}-1}}{a^2}$$

$$z_y = \frac{\partial z}{\partial y} = -\frac{Ly\left(\left(\frac{x}{a}\right)^2 + \left(\frac{y}{c}\right)^2\right)^{\frac{k}{2}-1}\left(1 - \left(\left(\frac{x}{a}\right)^2 + \left(\frac{y}{c}\right)^2\right)^{\frac{k}{2}}\right)^{\frac{1}{k}-1}}{c^2}$$

$$A = 8\int_0^a \int_0^{c\sqrt{1-\left(\frac{x}{a}\right)^2}} \sqrt{1+z_x^2+z_y^2}dydx$$

The volume is given by

$$V = \frac{4\pi}{3k} acL \frac{\Gamma\left(\frac{2}{k}\right)\Gamma\left(\frac{1}{k}\right)}{\Gamma\left(\frac{3}{k}\right)}$$

For $k = 2$ it reduces to the ellipsoid volume

$$V = \frac{4\pi}{3} acL$$

For $k \to \infty$ it reduces to

$$\lim_{k\to\infty} \frac{\Gamma\left(\frac{a}{k}\right)}{\Gamma\left(\frac{b}{k}\right)} = \frac{b}{a}$$

$$\lim_{k\to\infty} \frac{\Gamma\left(\frac{a}{k}\right)}{\mathrm{k}} = \frac{1}{a}$$

to obtain for the volume

$$V = 2\pi acL$$

which is the volume of a cylinder with ellipsoidal cross-section. Then the Porod asymptote can be calculated **(S.4.9.3.8)**

$$P(q) = \frac{2\pi A}{V^2 q^4}$$

Rotation body, elliptical, monodisperse, isotropic, P(q)

Similarly, we obtain for the isotropic polydisperse case **(S.4.9.3.9)**

$$P(q) = \frac{\Gamma[z+s-3](z+1)^4}{\Gamma[z+s+1]} \frac{2\pi A}{V^2 q^4}$$

Rotation body, elliptical, polydisperse, isotropic, P(q)

To summarize, we have for the formfactor P(q) Eq. (4.9.3.6)

$$\langle P(q)\rangle = \left(\frac{\Gamma\left(\frac{k+3}{k}\right)}{\Gamma\left(\frac{2+k}{k}\right)\Gamma\left(\frac{1}{k}\right)}\right)^2 \sum_{n=0}^{\infty} (z+1)_{2n}\Gamma\left(n+\frac{1}{2}\right)\left(-\frac{q^2L^2}{4(z+1)^2}\right)^n \sum_{m=0}^{\infty} (m!)(z+1)_{2m}\left(-\frac{q^2a^2}{4(z+1)^2}\right)^m \frac{e_m c_{n,m}}{2\Gamma\left(m+n+\frac{3}{2}\right)}$$

together with the Porod asymptote (S.4.9.3.9) used for the

Mathematica code implementation:

```
a=1;
c=1.3;
eps=c/a;
k=2.3;
na=40;
ma=na;
ff=Table[Sum[(Gamma[(2*ns+1)/k]*Gamma[(2*(n-ns)+1)/k]/(Pochhammer[1/2,ns]*(ns!)*Pochhammer[1/2,n-ns]*((n-
ns)!)))*Sum[(Gamma[(2*ms+2+k)/k]*Gamma[(2*(m-ms)+2+k)/k]/(Pochhammer[2,ms]*(ms!)*Pochhammer[2,m-ms]*((m-
ms)!)))*(1/(Gamma[(2*(ns+ms)+k+3)/k]*Gamma[(2*(n-ns+m-ms)+k+3)/k])),{ms,0,m}],{ns,0,n}],{n,0,na},{m,0,ma}];
ee=Table[(1/Pi)*Sum[(eps^(2*n-m))*Gamma[n-m+1/2]*Gamma[m+1/2]/(((n-m)!)*(m!)), {m, 0, n}],{n,0,na}];

L=1.5;
sigma=0.1;
q=10^lq;
qs=Sqrt[qx*qx+qy*qy];
z=(1-sigma*sigma)/(sigma*sigma);
vol=4*Pi*a*c*L*Gamma[2/k]*Gamma[1/k]/(3*k*Gamma[3/k]);
zx=-(L*x/(a*a))*((((x/a)^2)+((y/c)^2))^((k/2)-1))*(1-(((x/a)^2)+((y/c)^2))^(k/2))^((1/k)-1);
zy=-(L*y/(c*c))*((((x/a)^2)+((y/c)^2))^((k/2)-1))*(1-(((x/a)^2)+((y/c)^2))^(k/2))^((1/k)-1);
DS=Sqrt[1+zx*zx+zy*zy];
area=8*NIntegrate[DS, {x, 0, a}, {y, 0, c*Sqrt[1-((x/a)^2)]}];
a1=Gamma[(k+3)/k]/(Gamma[(2+k)/k]*Gamma[1/k]);
apor=Gamma[z-3]*((z+1)^4)/Gamma[z+1];
Pqav=a1*a1*Sum[(Gamma[n+1/2]*((-q*q*L*L/(4))^n))*Sum[(m!)*(((-
q*q*a*a/(4))^m))*ee[[m+1]]*ff[[n+1,m+1]]/(2*Gamma[m+n+3/2]),{m,0,ma}],{n,0,na}];
Pqavz=a1*a1*Sum[(Gamma[n+1/2]*Pochhammer[z+1,2*n]*((-
q*q*L*L/(4*(z+1)*(z+1)))^n))*Sum[(m!)*Pochhammer[z+1,2*m]*(((-
q*q*a*a/(4*(z+1)*(z+1)))^m))*ee[[m+1]]*ff[[n+1,m+1]]/(2*Gamma[m+n+3/2]),{m,0,ma}],{n,0,na}];
Pqavzs=a1*a1*Sum[(Gamma[n+1/2]*Pochhammer[z+1,2*n]*((-
qs*qs*L*L/(4*(z+1)*(z+1)))^n))*Sum[(m!)*Pochhammer[z+1,2*m]*(((-
qs*qs*a*a/(4*(z+1)*(z+1)))^m))*ee[[m+1]]*ff[[n+1,m+1]]/(2*Gamma[m+n+3/2]),{m,0,ma}],{n,0,na}];
Pqpor=apor*2*Pi*area/((q^4)*vol*vol);
lim=1.5;
pl1=Plot[Log[10,Pqav], {lq , -1,lim}, PlotRange->{-6,1}, GridLines->{Automatic}, LabelStyle->Directive[Black,16],AxesLabel-
>{"log q","lg P(q)"}, AxesOrigin->{-1,-6},TicksStyle->Directive[Black,12],PlotStyle->{Black}] ;
pl2=Plot[Log[10,Pqavz], {lq , -1,lim}, PlotRange->{-6,1}, GridLines->{Automatic}, LabelStyle->Directive[Black,16],AxesLabel-
>{"log q","lg P(q)"}, AxesOrigin->{-1,-6},TicksStyle->Directive[Black,12],PlotStyle->{Blue}]  ;
pl3=Plot[Log[10,Pqpor], {lq , -1,lim}, PlotRange->{-6,1}, GridLines->{Automatic}, LabelStyle->Directive[Black,16],AxesLabel-
>{"log q","lg P(q)"}, AxesOrigin->{-1,-6},TicksStyle->Directive[Black,12],PlotStyle->{Red}];
Show[pl2,pl3]
lims=10;
pl5=DensityPlot[Log[10,Pqavzs],{qx, -lims, lims}, {qy, -lims, lims}, PlotRange->{-8,0}, PlotPoints->50, PlotLegends-
>Automatic, LabelStyle->Directive[Black,12],AxesLabel->Automatic]
```

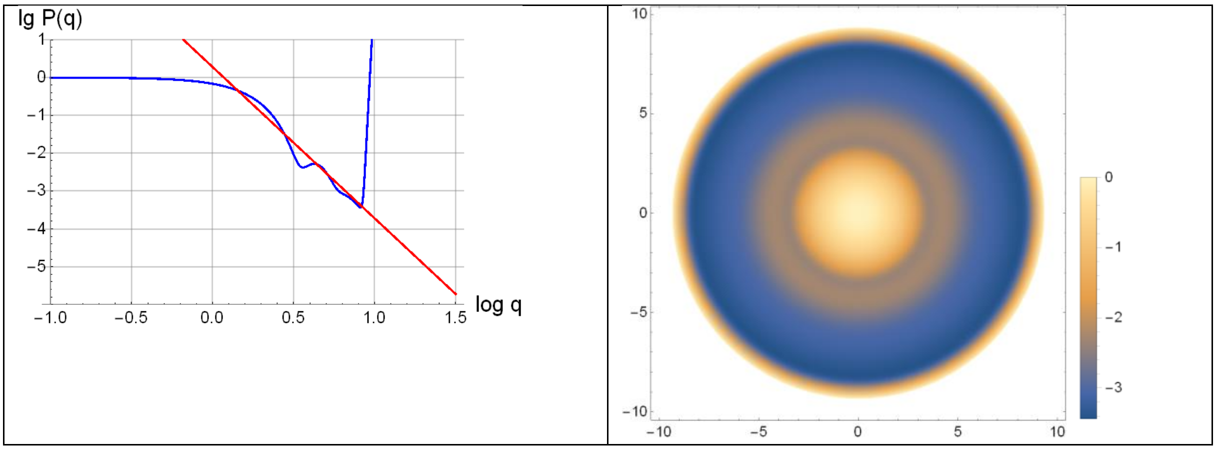

Fig. S39: Formfactor of polydisperse isotropic rotation bodies with elliptical cross-section.

To summarize, we have for the scattering amplitude F(q) Eq. (4.9.1.14)

$$\langle F(q)\rangle^2 = \left(\frac{\Gamma\left(\frac{k+3}{k}\right)}{\Gamma\left(\frac{2+k}{k}\right)\Gamma\left(\frac{1}{k}\right)}\right)^2 \sum_{n=0}^{\infty}\Gamma\left(n+\frac{1}{2}\right)\left(-\frac{q^2L^2}{4(z+1)^2}\right)^n \sum_{m=0}^{\infty} m!\left(-\frac{q^2a^2}{4(z+1)^2}\right)^m \frac{e_m\, f_{n,m}}{2\Gamma\left(m+n+\frac{3}{2}\right)}$$

Mathematica code implementation:

```
a=1;
c=1.3;
eps=c/a;
k=2.3;
na=40;
ma=na;
cc=Table[Sum[(Gamma[(2*ns+1)/k]*Gamma[(2*(n-ns)+1)/k]/(Pochhammer[1/2,ns]*(ns!)*Pochhammer[1/2,n-ns]*((n-
ns)!)))*Sum[(Gamma[(2*ms+2+k)/k]*Gamma[(2*(m-ms)+2+k)/k]/(Pochhammer[2,ms]*(ms!)*Pochhammer[2,m-ms]*((m-
ms)!)))*(1/(Gamma[(2*(ns+ms)+k+3)/k]*Gamma[(2*(n-ns+m-ms)+k+3)/k])),{ms,0,m}],{ns,0,n}],{n,0,na},{m,0,ma}];
ff=Table[Sum[(Gamma[(2*ns+1)/k]*Gamma[(2*(n-ns)+1)/k]*Pochhammer[z+1,2*(n-
ns)]*Pochhammer[z+1,2*ns]/(Pochhammer[1/2,ns]*(ns!)*Pochhammer[1/2,n-ns]*((n-
ns)!)))*Sum[(Gamma[(2*ms+2+k)/k]*Gamma[(2*(m-ms)+2+k)/k]*Pochhammer[z+1,2*(m-
ms)]*Pochhammer[z+1,2*ms]/(Pochhammer[2,ms]*(ms!)*Pochhammer[2,m-ms]*((m-
ms)!)))*(1/(Gamma[(2*(ns+ms)+k+3)/k]*Gamma[(2*(n-ns+m-ms)+k+3)/k])),{ms,0,m}],{ns,0,n}],{n,0,na},{m,0,ma}];
ee=Table[(1/Pi)*Sum[(eps^(2*n-m))*Gamma[n-m+1/2]*Gamma[m+1/2]/(((n-m)!)*(m!)), {m, 0, n}],{n,0,na}];

L=1.5;
sigma=0.1;
q=10^lq;
qs=Sqrt[qx*qx+qy*qy];
z=(1-sigma*sigma)/(sigma*sigma);
a1=Gamma[(k+3)/k]/(Gamma[(2+k)/k]*Gamma[1/k]);
Fqav=a1*a1*Sum[(Gamma[n+1/2]*((-q*q*L*L/(4))^n))*Sum[(m!)*(((-
q*q*a*a/(4))^m))*ee[[m+1]]*cc[[n+1,m+1]]/(2*Gamma[m+n+3/2]),{m,0,ma}],{n,0,na}];
Fqavz=a1*a1*Sum[(Gamma[n+1/2]*((-q*q*L*L/(4*(z+1)*(z+1)))^n))*Sum[(m!)*(((-
q*q*a*a/(4*(z+1)*(z+1)))^m))*ee[[m+1]]*ff[[n+1,m+1]]/(2*Gamma[m+n+3/2]),{m,0,ma}],{n,0,na}];
Fqavzs=a1*a1*Sum[(Gamma[n+1/2]*((-qs*qs*L*L/(4*(z+1)*(z+1)))^n))*Sum[(m!)*(((-
qs*qs*a*a/(4*(z+1)*(z+1)))^m))*ee[[m+1]]*ff[[n+1,m+1]]/(2*Gamma[m+n+3/2]),{m,0,ma}],{n,0,na}];
lim=1.5;
pl1=Plot[Log[10,Fqav], {lq , -1,lim}, PlotRange->{-6,1}, GridLines->{Automatic}, LabelStyle->Directive[Black,16],AxesLabel-
>{"log q","lg F^2(q)"}, AxesOrigin->{-1,-6},TicksStyle->Directive[Black,12],PlotStyle->{Black}]
pl2=Plot[Log[10,Fqavz], {lq , -1,lim}, PlotRange->{-6,1}, GridLines->{Automatic}, LabelStyle->Directive[Black,16],AxesLabel-
>{"log q","lg F^2(q)"}, AxesOrigin->{-1,-6},TicksStyle->Directive[Black,12],PlotStyle->{Blue}]
Show[pl1,pl2]
lims=10;
pl5=DensityPlot[Log[10,Fqavzs],{qx, -lims, lims}, {qy, -lims, lims}, PlotRange->{-8,0}, PlotPoints->50, PlotLegends->Automatic,
LabelStyle->Directive[Black,12],AxesLabel->Automatic]
```

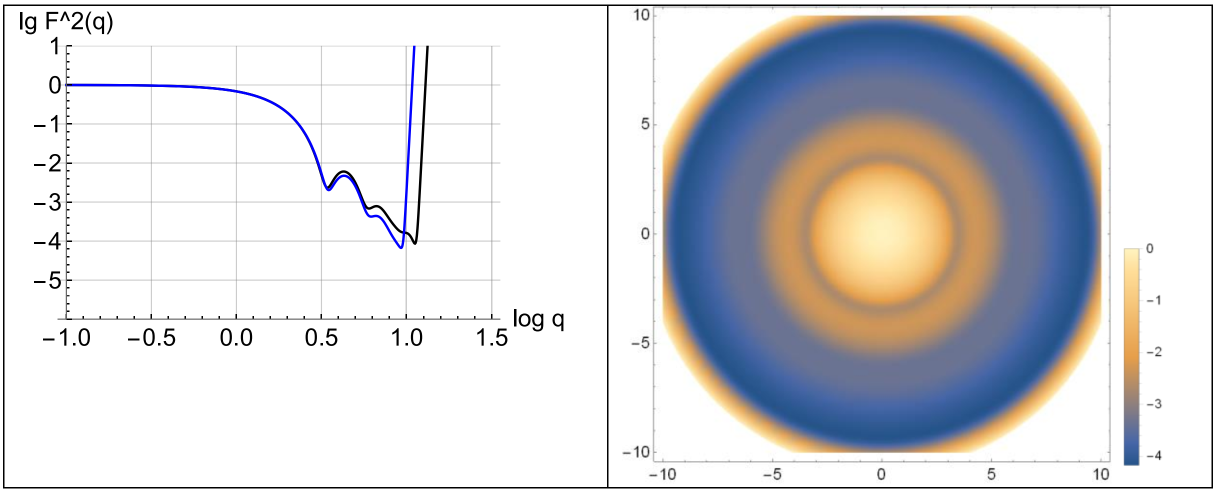

Fig. S40: Scattering amplitude of monodisperse and polydisperse isotropic rotation bodies with elliptical cross-section.

4.10 Exluded Volume Chain

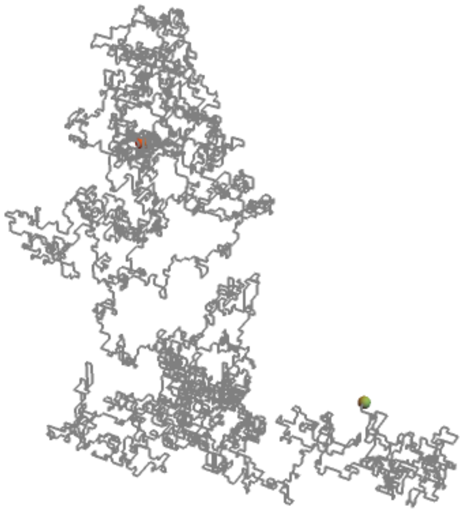

Fig. S41: Polymer excluded volume chain (from demonstrations.wolfram.com)

Regime I. The formfactor of an excluded volume chain has been calculated in ref. [9]. It is given by a series expansion **(S.4.10.1)**

$$P(q) = 2A_s \sum_{n=0}^{\infty} \frac{\Gamma\left(\frac{2+d+\theta+2n}{\delta}\right)}{\Gamma\left(\frac{d}{2}+n\right)} \frac{(1+2\nu)^n(2+2\nu)^n(z+1)_{2\nu n}}{(1+2\nu n)(2+2\nu n)n!} \left(-\frac{q^2R_g^2}{4d(z+1)^2D_s^{2/\delta}}\right)^n$$

Excluded volume chain, polydisperse, P(q)

with a radius of gyration R_g and

$$A_s = \frac{2\pi^{d/2} B_s}{\delta D_s^{\frac{2+d+\theta}{\delta}}}$$

and the normalization constants

$$D_s = \left(\frac{1}{d} \frac{\Gamma\left(\frac{4+d+\theta}{\delta}\right)}{\Gamma\left(\frac{2+d+\theta_s}{\delta}\right)} \right)^{\delta/2}$$

$$B_s = \frac{\delta\Gamma\left(\frac{d}{2}\right)}{2\pi^{d/2}} \frac{D_s^{\frac{2+d+\theta}{\delta}}}{\Gamma\left(\frac{2+d+\theta}{\delta}\right)}$$

with d the dimensionality and the exponents

$$\delta = \frac{1}{1-\nu}$$

$$\theta = \frac{\gamma - 1}{\nu}$$

Regime II. The asymptotic expansion is **(S.4.10.2)**

$$P(q) = A_s \frac{\Gamma\left(\frac{1}{2\nu}\right)\Gamma\left(\frac{2+d+\theta}{\delta} - \frac{1}{\nu\delta}\right)}{\nu z \Gamma\left(\frac{d}{2} - \frac{1}{2\nu}\right)} \left(\frac{(1+2\nu)(2+2\nu)q^2 R_g^2}{4\mathrm{d}(z+1)^{2\nu} D_s^{2/\delta_s}} \right)^{-1/(2\nu)}$$

In case of $d = 3$ renormalization group theory (RNG) predicts the currently best estimates for excluded volume chains are $\nu = 0.588$ and $\gamma = 1.1619$. In the Gaussian limit in $d = 3$ we have $\nu = 1/2$ and $\gamma = 0$ and therefore $\delta_s = 2$ and $\theta_s = -2$. In the monodisperse case the fromfactor then reduces to the Debye formfactor

$$P(q) = 2\sum_{n=0}^{\infty} \frac{(-x)^n}{(n+2)!} = 2\left(\frac{e^{-x}}{x^2} + \frac{1}{x} - \frac{1}{x^2}\right)$$

with $x = q^2 R_g^2$.

```
d=3;
rg=1.2;
sigma=0.1;
z=(1-sigma*sigma)/(sigma*sigma);
q=10^lq;
x=q*q*rg*rg;
nmax=120;
vs=0.588;
gs=1.1619;
deltas=1/(1-vs);
thetas=(gs-1)/vs;
fac=(2+d+thetas)/deltas;
ds=((1/d)*Gamma[(4+d+thetas)/deltas]/Gamma[fac])^(deltas/2);
bs=(deltas*Gamma[d/2]/(2*(Pi^(d/2))))*((ds^fac)/Gamma[fac]);
as=2*(Pi^(d/2))*bs/(deltas*(ds^fac));
```

```
Pq1=2*as*Sum[(Gamma[(2+d+thetas+2*n)/deltas]/Gamma[n+d/2])*(((1+2*vs)^n)*((2+2*vs)^n)*Pochh
ammer[z+1,2*n]/((1+2*vs*n)*(2+2*vs*n)*(n!)))*((-x/(4*d*((z+1)^(2))*(ds^(2/deltas))))^n),
{n, 0, nmax}];
Pq1a=2*Sum[((-x)^n)/((n+2)!), {n, 0, nmax}];
Pq1b=2*((Exp[-x]/(x*x))+(1/x)-1/(x*x));
Pq2=as*(Gamma[1/(2*vs)]*Gamma[fac-1/(vs*deltas)]/(vs*z*Gamma[(d/2)-
(1/(2*vs))]))*((1+2*vs)*(2+2*vs)*x/(4*d*((z+1)^(2*vs))*(ds^(2/deltas))))^(-1/(2*vs));
Pq2a=2*(z+1)/(z*x);
pl1=Plot[Log[10,Pq1], {lq, -1,1}, PlotRange->{-3,1}];
pl2=Plot[Log[10,Pq1a], {lq, -1, 1}, PlotRange->{-3,1}];
pl2b=Plot[Log[10,Pq1b], {lq, -1, 1}, PlotRange->{-3,1}];
pl3=Plot[Log[10,Pq2], {lq, -1, 1}, PlotRange->{-3,1}];
pl4=Plot[Log[10,Pq2a], {lq, -1, 1}, PlotRange->{-3,1}];

pl1=Plot[Log[10,Pq1],{lq, -1,1},PlotRange->{-3,1},GridLines->{Automatic},LabelStyle-
>Directive[Black,16],AxesLabel->{"q","lg P(q)"},AxesOrigin->{-1,-3},TicksStyle-
>Directive[Black,12],PlotStyle->{Black}];
pl2=Plot[Log[10,Pq1a],{lq, -1,1},PlotRange->{-3,1},GridLines->{Automatic},LabelStyle-
>Directive[Black,16],AxesLabel->{"q","lg P(q)"},AxesOrigin->{-1,-3},TicksStyle-
>Directive[Black,12],PlotStyle->{Blue}];
pl3=Plot[Log[10,Pq1b],{lq, -1,1},PlotRange->{-3,1},GridLines->{Automatic},LabelStyle-
>Directive[Black,16],AxesLabel->{"q","lg P(q)"},AxesOrigin->{-1,-3},TicksStyle-
>Directive[Black,12],PlotStyle->{Red, Dashed}];
pl4=Plot[Log[10,Pq2],{lq, -1,1},PlotRange->{-3,1},GridLines->{Automatic},LabelStyle-
>Directive[Black,16],AxesLabel->{"q","lg P(q)"},AxesOrigin->{-1,-3},TicksStyle-
>Directive[Black,12],PlotStyle->{Black}];
pl5=Plot[Log[10,Pq2a],{lq, -1,1},PlotRange->{-3,1},GridLines->{Automatic},LabelStyle-
>Directive[Black,16],AxesLabel->{"q","lg P(q)"},AxesOrigin->{-1,-3},TicksStyle-
>Directive[Black,12],PlotStyle->{Blue}];
Show[pl1,pl4]
Show[pl1,pl2,pl3,pl4,pl5]
```

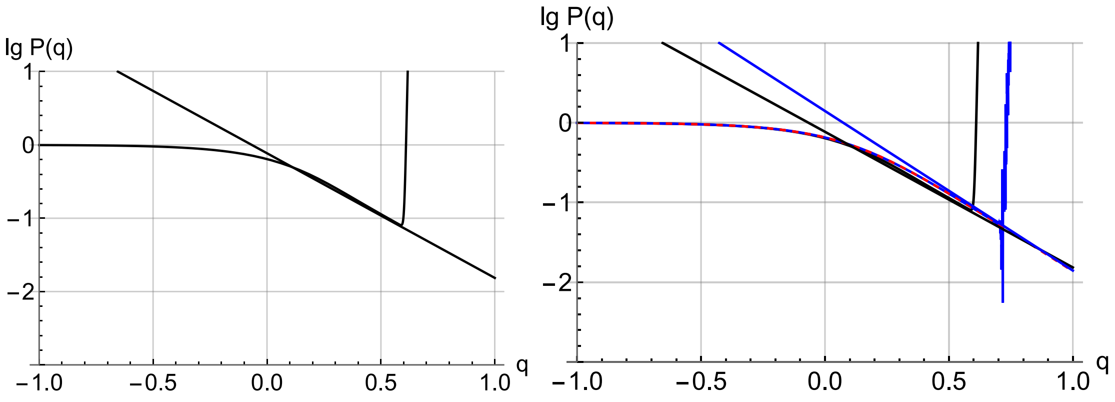

Fig. S42: Formfactor of the excluded volume chain (left, black), and together with Gaussian chain (blue).

5. Porod-asymptote for isotropic and oriented cases

Whereas for isotropic systems Porod's law, i.e. the q^{-4}-asymptote, is very frequently observed and analyzed, it is hardly ever considered for oriented cases. Here, we follow the work of Hamzeh and Bragg [14] for a rotation body with elliptical cross-section. Accordingly,

$$F(q) = \frac{1}{V}\int e^{i\boldsymbol{qr}} d\boldsymbol{r}$$

The integration over the volume is performed over the surface area $s(z,\theta)$ of the intersection of the particle with the plane at z, parallel to the (xy-plane).

$$F(q) = \frac{1}{V}\int s(z,\theta) e^{i\boldsymbol{qr}} dz$$

The calculation of $s(z,\theta)$ proceeds in several steps. We refer the reader to the original publication. As a result, for a biaxial ellipsoid it is

$$s(z,\theta) = \frac{\pi a^2 b}{H}\left(1 - \frac{z^2}{H^2}\right)$$

$$H = (a^2\sin^2\theta + b^2\cos^2\theta)^{1/2}$$

Thus, the angular dependence of the area can be written in terms of a single angular-dependent parameter H. This is unique for ellipsoids.

If we consider a biaxial ellipsoid, the volume is $V = \frac{4}{3}\pi a^2 b$ and we have

$$F(q) = \frac{3}{4\pi a^2 b}\frac{\pi a^2 b}{H}\int_{-H}^{H}\left(1 - \frac{z^2}{H^2}\right)e^{iqz} dz = \frac{3}{4H}\int_{-H}^{H}\left(1 - \frac{z^2}{H^2}\right)e^{iqz} dz$$

$$= \frac{3}{4H}\int_{-H}^{H}\left(1 - \frac{z^2}{H^2}\right)\cos(qz)\, dz = \frac{3}{4H}4\left(\frac{\sin(qH) - qH\cos(qH)}{q^3 H^2}\right)$$

$$= 3\left(\frac{\sin(qH) - qH\cos(qH)}{q^3 H^3}\right)$$

Therefore, the formfactor is

$$P(q) = 9\left(\frac{\sin(qH) - qH\cos(qH)}{q^3 H^3}\right)^2$$

In the Porod-region, the expression can be approximated by the leading term

$$P(q) = 9\left(\frac{\cos(qH)}{q^2 H^2}\right)^2$$

At high q the cosine square term oscillates rapidly, and can be replaced by its average value ½. We therefore have **(S.5.1)**

$$\lim_{q\to\infty} P(q) = \frac{9}{2q^4 H^4}$$

For a tri-axial ellipsoids we have

$$s(z,\theta,\phi) = \frac{\pi abc}{H}\left(1 - \frac{z^2}{H^2}\right)$$

$$H(\theta,\phi) = ((a\sin\theta\cos\phi)^2 + (b\sin\theta\sin\phi)^2 + (c\cos\theta)^2)^{1/2}$$

This similarly leads to the asymptote **(S.5.2)**

$$\lim_{q\to\infty} P(q) = \frac{9}{2q^4H^4}$$

We make extensive use of these results to calculate the scattering patterns for oriented ellipsoids.

6. Perfect Orientation

Perfectly oriented anisotropic particles will be oriented in space with respect to the scattering vector $\boldsymbol{q} = (q_x, q_y, q_z)$. To orient the particles in arbitrary spatial direction we introduce the rotation matrices yielding rotations about the three coordinate axes as

$$\boldsymbol{R}_x = \begin{pmatrix} 1 & 0 & 0 \\ 0 & \cos\alpha & -\sin\alpha \\ 0 & \sin\alpha & \cos\alpha \end{pmatrix}$$

$$\boldsymbol{R}_y = \begin{pmatrix} \cos\beta & 0 & \sin\beta \\ 0 & 1 & 0 \\ -\sin\beta & 0 & \cos\beta \end{pmatrix}$$

$$\boldsymbol{R}_z = \begin{pmatrix} \cos\gamma & -\sin\gamma & 0 \\ \sin\gamma & \cos\gamma & 0 \\ 0 & 0 & 1 \end{pmatrix}$$

$$\boldsymbol{R}_{x,y,z} = \boldsymbol{R}_z\boldsymbol{R}_y\boldsymbol{R}_x$$

$$\boldsymbol{q} = \begin{pmatrix} q_x \\ q_y \\ q_z \end{pmatrix}$$

with $q_z \approx 0$ for small-angle scattering experiments. Then the rotated $\boldsymbol{q}$-vector is

$$\boldsymbol{q}_r = \boldsymbol{R}_{x,y,z}\boldsymbol{q}$$

In a different representation we have

$$\boldsymbol{q} = \begin{pmatrix} q_x \\ q_y \\ q_z \end{pmatrix} = \begin{pmatrix} q\cos\phi\sin\theta \\ q\sin\phi\sin\theta \\ q\cos\theta \end{pmatrix}$$

6.1 Parallelepiped

6.1.1 Formfactor P(q)

Regime I. From Eq. (4.5.1.2) we take the series expansion **(S.6.1.1.1)**

$$\langle P_{cube}(q)\rangle_a = \left(\sum_{n=0}^{\infty}\frac{4^n}{(n+1)}\frac{(z+s+1)_{2n}}{\left(\frac{3}{2}\right)_n n!}\left(-\frac{(q_xa)^2}{4(z+1)^2}\right)^n\right)\left(\sum_{m=0}^{\infty}\frac{4^m}{(n+1)}\frac{(z+s+1)_{2m}}{\left(\frac{3}{2}\right)_m m!}\left(-\frac{(q_yb)^2}{4(z+1)^2}\right)^m\right)\left(\sum_{l=0}^{\infty}\frac{4^l}{(l+1)}\frac{(z+s+1)_{2l}}{\left(\frac{3}{2}\right)_l l!}\left(-\frac{(q_zc)^2}{4(z+1)^2}\right)^l\right)$$

$$= \sum_{n=0}^{\infty}4^n\left(-\frac{1}{4(z+1)^2}\right)^n\sum_{m=0}^{n}\frac{(z+s+1)_{2(n-m)}}{\left(\frac{3}{2}\right)_{n-m}(n-m+1)!}(q_xa)^{2(n-m)}\sum_{l=0}^{m}\frac{(z+s+1)_{2(m-l)}}{\left(\frac{3}{2}\right)_{m-l}(m-l+1)!}(q_yb)^{2(m-l)}\frac{(z+s+1)_{2l}}{\left(\frac{3}{2}\right)_l (l+1)!}(q_zc)^{2l}$$

Regime II. From Eq. (S.4.5.1.4) we take the product of averaged trigonometric functions **(S.6.1.1.2)**

$$\langle P_{cube}(q)\rangle_a = \langle\left(\frac{\sin(q_xa)}{q_xa}\right)^2\rangle_a\langle\left(\frac{\sin(q_yb)}{q_yb}\right)^2\rangle_b\langle\left(\frac{\sin(q_zc)}{q_zc}\right)^2\rangle_c$$

$$\langle\left(\frac{\sin(q_xa)}{q_xa}\right)^2\rangle_a = \frac{\Gamma[z+s-n+1]}{\Gamma[z+s+1]}\frac{1}{2}u^{-2}\left(1-\frac{\cos[(z+s-n+1)\arctan(2au)]}{(1+4a^2u^2)^{\frac{z+s-n+1}{2}}}\right)$$

$$u = \frac{q_ia}{z+1}$$

Regime III. The high-q Porod asymptotes are **(S.6.1.1.3)**

$$\langle\left(\frac{\sin(q_xa)}{q_xa}\right)^2\rangle_a = \frac{\Gamma[z+s-n+1]}{\Gamma[z+s+1]}\frac{1}{2}u^{-2}$$

Mathematica code implementation:

```
a=1;
b=2;
c=3;
sigma=0.1;
z=(1-sigma*sigma)/(sigma*sigma);
phi=0.3*Pi/2;
theta=0.6*Pi/2;
alf=0.3*Pi/2;
beta=0.2*Pi/2;
gam=0.1*Pi/2;
RRx={{1,0,0},{0,Cos[alf],-Sin[alf]},{0,Sin[alf],Cos[alf]}};
RRy={{Cos[beta],0,Sin[beta]},{0,1,0},{-Sin[beta],0,Cos[beta]}};
RRz={{Cos[gam],-Sin[gam],0},{Sin[gam],Cos[gam],0},{0,0,1}};
RR=RRz.RRy.RRz;
qz=0.001;
qv={qx,qy,qz};
qvs=RR.qv;
qxx=qvs[[1]];
qyy=qvs[[2]];
qzz=qvs[[3]];
qs=10^lqs;
qxs=qs*Cos[phi]*Sin[theta];
```

```
qys=qs*Sin[phi]*Sin[theta];
qzs=qs*Cos[theta];
nmax=50;
sum1=Sum[(4^n)*Pochhammer[z+1,2*n]*((-qxx*qxx*a*a/(4*(z+1)*(z+1)))^n)/((n+1)*Pochhammer[3/2,n]*(n!)), {n, 0,
nmax}];
sum2=Sum[(4^n)*Pochhammer[z+1,2*n]*((-qyy*qyy*b*b/(4*(z+1)*(z+1)))^n)/((n+1)*Pochhammer[3/2,n]*(n!)), {n, 0,
nmax}];
sum3=Sum[(4^n)*Pochhammer[z+1,2*n]*((-qzz*qzz*c*c/(4*(z+1)*(z+1)))^n)/((n+1)*Pochhammer[3/2,n]*(n!)), {n, 0,
nmax}];
Pq1=sum1*sum2*sum3;
sum1s=Sum[(4^n)*Pochhammer[z+1,2*n]*((-qxs*qxs*a*a/(4*(z+1)*(z+1)))^n)/((n+1)*Pochhammer[3/2,n]*(n!)), {n, 0,
nmax}];
sum2s=Sum[(4^n)*Pochhammer[z+1,2*n]*((-qys*qys*b*b/(4*(z+1)*(z+1)))^n)/((n+1)*Pochhammer[3/2,n]*(n!)), {n, 0,
nmax}];
sum3s=Sum[(4^n)*Pochhammer[z+1,2*n]*((-qzs*qzs*c*c/(4*(z+1)*(z+1)))^n)/((n+1)*Pochhammer[3/2,n]*(n!)), {n, 0,
nmax}];
Pq1s=sum1s*sum2s*sum3s;
argx=qxx*a/(z+1);
argy=qyy*b/(z+1);
argz=qzz*c/(z+1);
px=(Gamma[z-1]/(2*Gamma[z+1]))*(argx^(-2))*(1-Cos[(z-1)*ArcTan[2*argx]]/((1+4*argx*argx)^((z-1)/2)));
py=(Gamma[z-1]/(2*Gamma[z+1]))*(argy^(-2))*(1-Cos[(z-1)*ArcTan[2*argy]]/((1+4*argy*argy)^((z-1)/2)));
pz=(Gamma[z-1]/(2*Gamma[z+1]))*(argz^(-2))*(1-Cos[(z-1)*ArcTan[2*argz]]/((1+4*argz*argz)^((z-1)/2)));
Pq2=px*py*pz;
argxs=qxs*a/(z+1);
argys=qys*b/(z+1);
argzs=qzs*c/(z+1);
pxs=(Gamma[z-1]/(2*Gamma[z+1]))*(argxs^(-2))*(1-Cos[(z-1)*ArcTan[2*argxs]]/((1+4*argxs*argxs)^((z-1)/2)));
pys=(Gamma[z-1]/(2*Gamma[z+1]))*(argys^(-2))*(1-Cos[(z-1)*ArcTan[2*argys]]/((1+4*argys*argys)^((z-1)/2)));
pzs=(Gamma[z-1]/(2*Gamma[z+1]))*(argzs^(-2))*(1-Cos[(z-1)*ArcTan[2*argzs]]/((1+4*argzs*argzs)^((z-1)/2)));
Pq2s=pxs*pys*pzs;
pxsa=(Gamma[z-1]/(2*Gamma[z+1]))*(argxs^(-2));
pysa=(Gamma[z-1]/(2*Gamma[z+1]))*(argys^(-2));
pzsa=(Gamma[z-1]/(2*Gamma[z+1]))*(argzs^(-2));
Pq2sa=pxsa*pysa*pzsa;
lim=2;
pl1=Plot[Log[10,Pq1s], {lqs, -1, lim}, PlotRange->{-10,1}, GridLines->{Automatic}, LabelStyle->Directive[Black,16],AxesLabel-
>{"log q","lg P(q)"}, AxesOrigin->{-1,-10},TicksStyle->Directive[Black,10],PlotStyle->{Blue,Dashed}];
pl2=Plot[Log[10,Pq2s], {lqs, -1, lim}, PlotRange->{-10,1}, GridLines->{Automatic}, LabelStyle->Directive[Black,16],AxesLabel-
>{"log q","lg P(q)"}, AxesOrigin->{-1,-10},TicksStyle->Directive[Black,10],PlotStyle->{Black}];
pl3=Plot[Log[10,Pq2sa], {lqs, -1, lim}, PlotRange->{-10,1}, GridLines->{Automatic}, LabelStyle->Directive[Black,16],AxesLabel-
>{"log q","lg P(q)"}, AxesOrigin->{-1,-10},TicksStyle->Directive[Black,10],PlotStyle->{Red}];
Show[pl1,pl2,pl3]
lims=10;
pl4=DensityPlot[Log[10,Pq1],{qx, -lims, lims}, {qy, -lims, lims}, PlotRange->{-8,0}, PlotPoints->50, PlotLegends->Automatic,
LabelStyle->Directive[Black,12],AxesLabel->Automatic]
pl5=DensityPlot[Log[10,Pq2],{qx, -lims, lims}, {qy, -lims, lims}, PlotRange->{-8,0}, PlotPoints->50, PlotLegends->Automatic,
LabelStyle->Directive[Black,12],AxesLabel->Automatic]
```

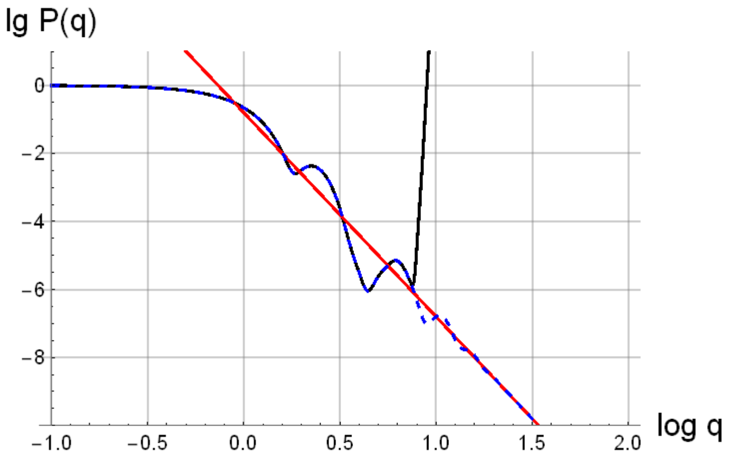

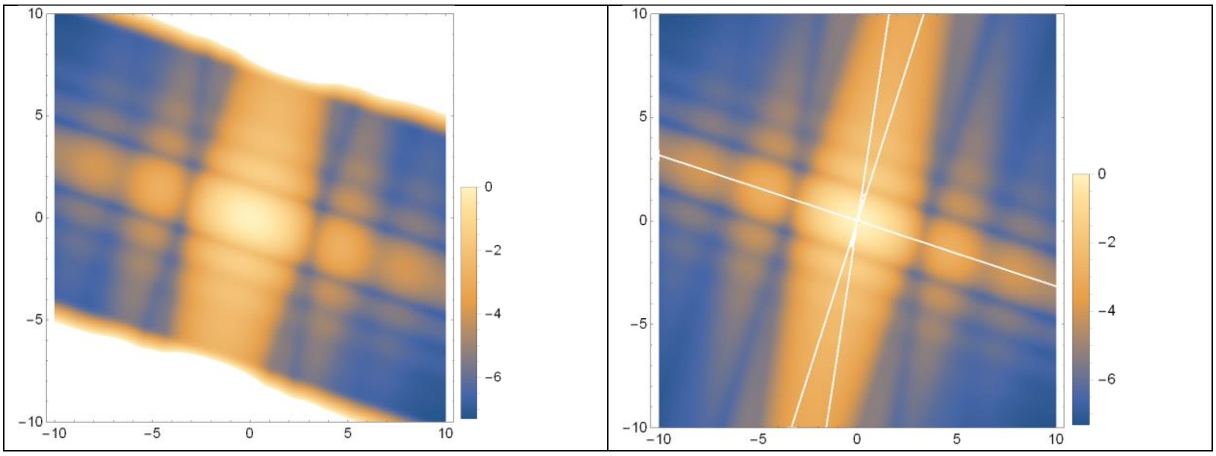

Fig. S43: Formfactor of perfectly oriented polydisperse parallelepipeds for the series expansion (left) and the asymptotic expansion (right).

6.1.2 Scattering Amplitude F(q)

Regime I. From Eq. (S.4.5.2.2) we take the series expansion **(S.6.1.2.1)**

$$\langle F(q)\rangle^2 = \sum_{n=0}^{\infty}\left(-\frac{(q_x a)^2}{4(z+1)^2}\right)^n f_n^{(1)} \sum_{m=0}^{\infty}\left(-\frac{(q_y b)^2}{4(z+1)^2}\right)^m f_m^{(1)} \sum_{l=0}^{\infty}\left(-\frac{(q_z c)^2}{4(z+1)^2}\right)^l f_l^{(1)}$$

$$= \sum_{n=0}^{\infty}\left(-\frac{1}{4(z+1)^2}\right)^{n-m} \sum_{m=0}^{n} (q_x a)^{2(n-m)} f_{n-m}^{(1)} \sum_{l=0}^{\infty} (q_y b)^{2(m-l)} f_{m-l}^{(1)} (q_z c)^{2l} f_l^{(1)}$$

Regime II. From Eq. (S.4.5.2.4) we have the product of averaged trigonometric functions **(S.6.1.2.2)**

$$\langle F(q)\rangle^2 = \left(\langle\frac{\sin(qa\sin\theta\cos\phi)}{qa\sin\theta\cos\phi}\rangle\langle\frac{\sin(qa\sin\theta\sin\phi)}{qa\sin\theta\sin\phi}\rangle\langle\frac{\sin(qa\cos\theta)}{qa\cos\theta}\rangle\right)^2$$

with

$$\langle\frac{\sin(q_x a)}{q_x a}\rangle = \frac{\Gamma[z+s-1]}{\Gamma[z+s+1]}\left(\frac{q_x a}{z+1}\right)^{-1}\frac{\sin\left[(z+s-1)\arctan\left(\frac{q_x a}{z+1}\right)\right]}{\left(1+\left(\frac{q_x a}{z+1}\right)^2\right)^{\frac{z+s-1}{2}}}$$

Mathematica code implementation:

```
a=1;
b=2;
c=3;
sigma=0.1;
z=(1-sigma*sigma)/(sigma*sigma);
phi=0.3*Pi/2;
theta=0.6*Pi/2;
alf=0.3*Pi/2;
beta=0.2*Pi/2;
gam=0.1*Pi/2;
RRx={{1,0,0},{0,Cos[alf],-Sin[alf]},{0,Sin[alf],Cos[alf]}};
RRy={{Cos[beta],0,Sin[beta]},{0,1,0},{-Sin[beta],0,Cos[beta]}};
RRz={{Cos[gam],-Sin[gam],0},{Sin[gam],Cos[gam],0},{0,0,1}};
RR=RRz.RRy.RRz;
qz=0.001;
qv={qx,qy,qz};
qvs=RR.qv;
qxx=qvs[[1]];
qyy=qvs[[2]];
qzz=qvs[[3]];
qs=10^lqs;
qxs=qs*Cos[phi]*Sin[theta];
qys=qs*Sin[phi]*Sin[theta];
qzs=qs*Cos[theta];
nmax=60;
fff=Table[Sum[Pochhammer[z+1,2*(n-m)]*Pochhammer[z+1,2*m]/(Pochhammer[3/2,n-m]*Pochhammer[3/2,m]*((n-
m)!)*(m!)), {m, 0, n}],{n, 0, nmax}];
sum1=Sum[((-qxx*qxx*a*a/(4*(z+1)*(z+1)))^n)*fff[[n+1]],{n, 0, nmax}];
sum2=Sum[((-qyy*qyy*b*b/(4*(z+1)*(z+1)))^n)*fff[[n+1]],{n, 0, nmax}];
sum3=Sum[((-qzz*qzz*c*c/(4*(z+1)*(z+1)))^n)*fff[[n+1]],{n, 0, nmax}];
Pq1=sum1*sum2*sum3;
sum1s=Sum[((-qxs*qxs*a*a/(4*(z+1)*(z+1)))^n)*fff[[n+1]],{n, 0, nmax}];
sum2s=Sum[((-qys*qys*b*b/(4*(z+1)*(z+1)))^n)*fff[[n+1]],{n, 0, nmax}];
sum3s=Sum[((-qzs*qzs*c*c/(4*(z+1)*(z+1)))^n)*fff[[n+1]],{n, 0, nmax}];
Pq1s=sum1s*sum2s*sum3s;
argx=qxx*a/(z+1);
argy=qyy*b/(z+1);
argz=qzz*c/(z+1);
px=((Gamma[z]/(Gamma[z+1]))*(argx^(-1))*Sin[(z)*ArcTan[argx]]/((1+argx*argx)^((z)/2)))^2;
py=((Gamma[z]/(Gamma[z+1]))*(argy^(-1))*Sin[(z)*ArcTan[argy]]/((1+argy*argy)^((z)/2)))^2;
pz=((Gamma[z]/(Gamma[z+1]))*(argz^(-1))*Sin[(z)*ArcTan[argz]]/((1+argz*argz)^((z)/2)))^2;
Pq2=px*py*pz;
argxs=qxs*a/(z+1);
argys=qys*b/(z+1);
argzs=qzs*c/(z+1);
pxs=((Gamma[z]/(Gamma[z+1]))*(argxs^(-1))*Sin[(z)*ArcTan[argxs]]/((1+argxs*argxs)^((z)/2)))^2;
pys=((Gamma[z]/(Gamma[z+1]))*(argys^(-1))*Sin[(z)*ArcTan[argys]]/((1+argys*argys)^((z)/2)))^2;
```

```
pzs=((Gamma[z]/(Gamma[z+1]))*(argzs^(-1))*Sin[(z)*ArcTan[argzs]]/((1+argzs*argzs)^((z)/2)))^2;
Pq2s=pxs*pys*pzs;
lim=2;
pl1=Plot[Log[10,Pq1s], {lqs, -1, lim}, PlotRange->{-10,1}, GridLines->{Automatic}, LabelStyle->Directive[Black,16],AxesLabel->{"log q","lg F^2(q)"}, AxesOrigin->{-1,-10},TicksStyle->Directive[Black,10],PlotStyle->{Black}];
pl2=Plot[Log[10,Pq2s], {lqs, -1, lim}, PlotRange->{-10,1}, GridLines->{Automatic}, LabelStyle->Directive[Black,16],AxesLabel->{"log q","lg F^2(q)"}, AxesOrigin->{-1,-10},TicksStyle->Directive[Black,10],PlotStyle->{Blue}];
Show[pl1,pl2]
lims=10;
pl4=DensityPlot[Log[10,Pq1],{qx, -lims, lims}, {qy, -lims, lims}, PlotRange->{-8,0}, PlotPoints->50, PlotLegends->Automatic, LabelStyle->Directive[Black,12],AxesLabel->Automatic]
pl5=DensityPlot[Log[10,Pq2],{qx, -lims, lims}, {qy, -lims, lims}, PlotRange->{-8,0}, PlotPoints->50, PlotLegends->Automatic, LabelStyle->Directive[Black,12],AxesLabel->Automatic]
```

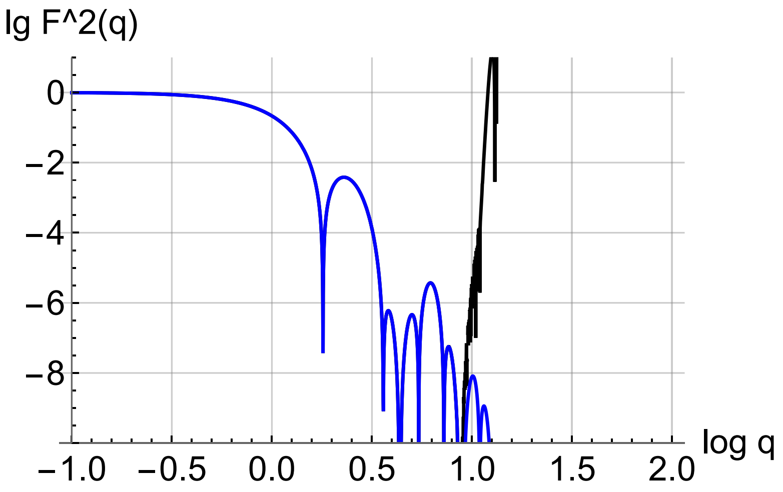

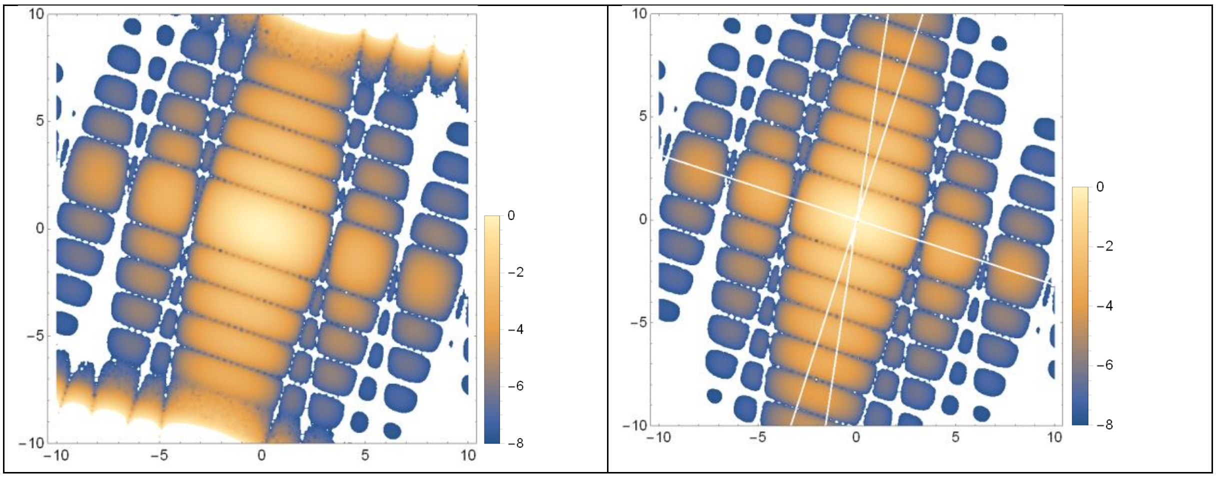

Fig. S44: Scattering amplitude of perfectly oriented polydisperse parallelepipeds.

6.1.3 Formfactor P(q), equatorially averaged

Regime I. The angular dependent formfactor for the perfectly oriented parallelepiped is given by (S.6.1.1.1)

$$P(q,\theta,\phi) = \sum_{n=0}^{\infty}\frac{4^n}{(n+1)\left(\frac{3}{2}\right)_n(n!)}\left(-\frac{q^2a^2(\sin\theta\cos\phi)^2}{4}\right)^n\sum_{m=0}^{\infty}\frac{4^m}{(m+1)\left(\frac{3}{2}\right)_m(m!)}\left(-\frac{q^2b^2(\sin\theta\sin\phi)^2}{4}\right)^m\sum_{l=0}^{\infty}\frac{4^l}{(l+1)\left(\frac{3}{2}\right)_l(l!)}\left(-\frac{q^2c^2(\cos\theta)^2}{4}\right)^l$$

We need to integrate over the angle ϕ

$$\langle P(q)\rangle = \sum_{n=0}^{\infty}\frac{4^n(z+1)_{2n}}{(n+1)\left(\frac{3}{2}\right)_n(n!)}\left(-\frac{q^2a^2(\sin\theta)^2}{4(z+1)^2}\right)^n\sum_{m=0}^{\infty}\frac{4^m(z+1)_{2m}}{(m+1)\left(\frac{3}{2}\right)_m(m!)}\left(-\frac{q^2b^2(\sin\theta)^2}{4(z+1)^2}\right)^m\left(\sum_{l=0}^{\infty}\frac{4^l(z+1)_{2l}}{(l+1)\left(\frac{3}{2}\right)_l(l!)}\left(-\frac{q^2c^2(\cos\theta)^2}{4(z+1)^2}\right)^l\right)\frac{2}{\pi}\int_0^{\pi/2}(\cos\phi)^{2n}(\sin\phi)^{2m}d\phi$$

The integral can be solved analytically

$$\frac{2}{\pi}\int_0^{\pi/2}(\cos\phi)^{2n}(\sin\phi)^{2m}d\phi = \frac{\Gamma\left(n+\frac{1}{2}\right)\Gamma\left(m+\frac{1}{2}\right)}{\pi\Gamma(n+m+1)}$$

We therefore have for the formfactor

$$\langle P(q)\rangle = \sum_{n=0}^{\infty}\frac{4^n(z+1)_{2n}}{(n+1)\left(\frac{3}{2}\right)_n(n!)}\left(-\frac{q^2a^2(\sin\theta)^2}{4(z+1)^2}\right)^n\sum_{m=0}^{\infty}\frac{4^m(z+1)_{2m}}{(m+1)\left(\frac{3}{2}\right)_m(m!)}\left(-\frac{q^2b^2(\sin\theta)^2}{4(z+1)^2}\right)^m\left(\sum_{l=0}^{\infty}\frac{4^l(z+1)_{2l}}{(l+1)\left(\frac{3}{2}\right)_l(l!)}\left(-\frac{q^2c^2(\cos\theta)^2}{4(z+1)^2}\right)^l\right)\frac{\Gamma\left(n+\frac{1}{2}\right)\Gamma\left(m+\frac{1}{2}\right)}{\pi\Gamma(n+m+1)}$$

$$= \frac{1}{\pi}\sum_{n=0}^{\infty}\frac{4^n\Gamma\left(n+\frac{1}{2}\right)(z+1)_{2n}}{(n+1)\left(\frac{3}{2}\right)_n(n!)}\left(-\frac{q^2a^2(\sin\theta)^2}{4(z+1)^2}\right)^n\sum_{m=0}^{\infty}\frac{4^m\Gamma\left(m+\frac{1}{2}\right)(z+1)_{2m}}{\Gamma(n+m+1)(m+1)\left(\frac{3}{2}\right)_m(m!)}\left(-\frac{q^2b^2(\sin\theta)^2}{4(z+1)^2}\right)^m\left(\sum_{l=0}^{\infty}\frac{4^l(z+1)_{2l}}{(l+1)\left(\frac{3}{2}\right)_l(l!)}\left(-\frac{q^2c^2(\cos\theta)^2}{4(z+1)^2}\right)^l\right)$$

We introduce the scattering vector components $q_r = q\sin\theta$ and $q_z = q\cos\theta$ to write

$$\langle P(q)\rangle = \frac{1}{\pi}\sum_{n=0}^{\infty}\frac{4^n\Gamma\left(n+\frac{1}{2}\right)(z+1)_{2n}}{(n+1)\left(\frac{3}{2}\right)_n(n!)}\left(-\frac{q_r^2a^2}{4(z+1)^2}\right)^n\sum_{m=0}^{\infty}\frac{4^m\Gamma\left(m+\frac{1}{2}\right)(z+1)_{2m}}{\Gamma(n+m+1)(m+1)\left(\frac{3}{2}\right)_m(m!)}\left(-\frac{q_r^2b^2}{4(z+1)^2}\right)^m\left(\sum_{l=0}^{\infty}\frac{4^l(z+1)_{2l}}{(l+1)\left(\frac{3}{2}\right)_l(l!)}\left(-\frac{q_z^2c^2}{4(z+1)^2}\right)^l\right)$$

Expressing the Pochhammer factorials in terms of Gamma functions yields

$$\langle P(q)\rangle = \frac{1}{\pi}\sum_{n=0}^{\infty}\frac{4^n\Gamma\left(n+\frac{1}{2}\right)\Gamma\left(\frac{3}{2}\right)(z+1)_{2n}}{\Gamma\left(n+\frac{3}{2}\right)(n+1)!}\left(-\frac{q_r^2a^2}{4(z+1)^2}\right)^n\sum_{m=0}^{\infty}\frac{4^m\Gamma\left(m+\frac{1}{2}\right)\Gamma\left(\frac{3}{2}\right)(z+1)_{2m}}{\Gamma(n+m+1)\Gamma\left(m+\frac{3}{2}\right)(m+1)!}\left(-\frac{q_r^2b^2}{4(z+1)^2}\right)^m\left(\sum_{l=0}^{\infty}\frac{4^l(z+1)_{2l}}{\left(\frac{3}{2}\right)_l(l+1)!}\left(-\frac{q_z^2c^2}{4(z+1)^2}\right)^l\right)$$

$$= \frac{1}{4}\sum_{n=0}^{\infty}\frac{4^n(z+1)_{2n}}{\left(n+\frac{1}{2}\right)(n+1)!}\left(-\frac{q_r^2a^2}{4(z+1)^2}\right)^n\sum_{m=0}^{\infty}\frac{4^m(z+1)_{2m}}{\Gamma(n+m+1)\left(m+\frac{1}{2}\right)(m+1)!}\left(-\frac{q_r^2b^2}{4(z+1)^2}\right)^m\left(\sum_{l=0}^{\infty}\frac{4^l(z+1)_{2l}}{\left(\frac{3}{2}\right)_l(l+1)!}\left(-\frac{q_z^2c^2}{4(z+1)^2}\right)^l\right)$$

Introducing $b = \delta a$ we can rewrite

$$\langle P(q)\rangle = \frac{1}{4}\sum_{n=0}^{\infty}\frac{4^n(z+1)_{2n}}{\left(n+\frac{1}{2}\right)(n+1)!}\left(-\frac{q_r^2a^2}{4(z+1)^2}\right)^n\sum_{m=0}^{\infty}\frac{4^m\Gamma(z+1)_{2m}}{\Gamma(n+m+1)\left(m+\frac{1}{2}\right)(m+1)!}\left(-\frac{q_r^2\delta^2a^2}{4(z+1)^2}\right)^m\left(\sum_{l=0}^{\infty}\frac{4^l(z+1)_{2l}}{\left(\frac{3}{2}\right)_l(l+1)!}\left(-\frac{q_z^2c^2}{4(z+1)^2}\right)^l\right)$$

which can be recast as **(S.6.1.3.1)**

$$\langle P(q)\rangle = \frac{1}{4}\sum_{n=0}^{\infty} 4^n \left(-\frac{q_r^2 a^2}{4(z+1)^2}\right)^{n-m} \sum_{m=0}^{n} \frac{(z+1)_{2(n-m)}\Gamma(z+1)_{2m}\delta^{2m}}{\left(n-m+\frac{1}{2}\right)(n-m+1)!\,\Gamma(n+m+1)\left(m+\frac{1}{2}\right)(m+1)!} \left(\sum_{l=0}^{\infty} \frac{4^l (z+1)_{2l}}{\left(\frac{3}{2}\right)_l (l+1)!}\left(-\frac{q_z^2 c^2}{4(z+1)^2}\right)^l\right)$$

Parallelepiped, equatorially averaged, P(q)

Regime II. From Eq. (S.4.5.1.4) we have the product of averaged trigonometric functions **(S.6.1.3.2)**

$$P_x(q,\phi) = \langle\frac{(\sin(q_r a))^2}{(q_r a\cos(\phi))^2}\rangle = \frac{\Gamma[z-1]}{\Gamma[z+1]}\frac{1}{2}\left(\frac{z+1}{q_r a\cos(\phi)}\right)^2 \left(1 - \frac{\cos\left[(z-1)\arctan\left(\frac{2q_r a\cos(\phi)}{z+1}\right)\right]}{\left(1+\left(\frac{2q_r a\cos(\phi)}{z+1}\right)^2\right)^{\frac{z-1}{2}}}\right)$$

$$P_y(q,\phi) = \langle\frac{(\sin(q_r b))^2}{(q_r b\sin(\phi))^2}\rangle = \frac{\Gamma[z-1]}{\Gamma[z+1]}\frac{1}{2}\left(\frac{z+1}{q_r b\sin(\phi)}\right)^2 \left(1 - \frac{\cos\left[(z-1)\arctan\left(\frac{2q_r b\sin(\phi)}{z+1}\right)\right]}{\left(1+\left(\frac{2q_r b\sin(\phi)}{z+1}\right)^2\right)^{\frac{z-1}{2}}}\right)$$

$$P_z(q) = \langle\frac{(\sin(q_z c))^2}{(q_z c)^2}\rangle = \frac{\Gamma[z-1]}{\Gamma[z+1]}\frac{1}{2}\left(\frac{z+1}{q_z c}\right)^2 \left(1 - \frac{\cos\left[(z-1)\arctan\left(\frac{2q_z c}{z+1}\right)\right]}{\left(1+\left(\frac{2q_z c}{z+1}\right)^2\right)^{\frac{z-1}{2}}}\right)$$

$$P(q) = P_z(q)\frac{2}{\pi}\int_0^{\pi/2} P_x(q,\phi)P_y(q,\phi)d\phi$$

Regime III. There is no simple Porod asymptote.

Mathematica code implementation:

```
a=1;
b=2;
c=3;
sigma=0.1;
z=(1-sigma*sigma)/(sigma*sigma);
theta=0.6*Pi/2;
alf=0.3*Pi/2;
beta=0.2*Pi/2;
gam=0.1*Pi/2;
RRx={{1,0,0},{0,Cos[alf],-Sin[alf]},{0,Sin[alf],Cos[alf]}};
RRy={{Cos[beta],0,Sin[beta]},{0,1,0},{-Sin[beta],0,Cos[beta]}};
RRz={{Cos[gam],-Sin[gam],0},{Sin[gam],Cos[gam],0},{0,0,1}};
RR=RRz.RRy.RRz;
qz=0.001;
qv={qx,qy,qz};
qvs=RR.qv;
qxx=qvs[[1]];
qyy=qvs[[2]];
qzz=qvs[[3]];
```

```
qrr=Sqrt[qxx*qxx+qyy*qyy];
qs=10^lqs;
qrs=qs*Sin[theta];
qzs=qs*Cos[theta];
nmax=60;
sum1=Sum[((4^n)*Pochhammer[z+1,2*n]*((-
qrr*qrr*a*a/(4*(z+1)*(z+1)))^n)/((n+1/2)*((n+1)!)))*Sum[(4^m)*Pochhammer[z+1,2*m]*((-
qrr*qrr*b*b/(4*(z+1)*(z+1)))^m)/((m+1/2)*((n+m)!)*((m+1)!)), {m, 0, nmax}], {n, 0, nmax}];
sum3=Sum[(4^n)*Pochhammer[z+1,2*n]*((-
qzz*qzz*c*c/(4*(z+1)*(z+1)))^n)/((n+1)*Pochhammer[3/2,n]*(n!)), {n, 0, nmax}];
Pq1=(1/4)*sum1*sum3;
sum1s=Sum[((4^n)*Pochhammer[z+1,2*n]*((-
qrs*qrs*a*a/(4*(z+1)*(z+1)))^n)/((n+1/2)*((n+1)!)))*Sum[(4^m)*Pochhammer[z+1,2*m]*((-
qrs*qrs*b*b/(4*(z+1)*(z+1)))^m)/((m+1/2)*((n+m)!)*((m+1)!)), {m, 0, nmax}], {n, 0, nmax}];
sum3s=Sum[(4^n)*Pochhammer[z+1,2*n]*((-
qzs*qzs*c*c/(4*(z+1)*(z+1)))^n)/((n+1)*Pochhammer[3/2,n]*(n!)), {n, 0, nmax}];
Pq1s=(1/4)*sum1s*sum3s;
argx=qrr*a*Cos[phi]/(z+1);
argy=qrr*b*Sin[phi]/(z+1);
argz=qzz*c/(z+1);
px=(Gamma[z-1]/(2*Gamma[z+1]))*(argx^(-2))*(1-Cos[(z-
1)*ArcTan[2*argx]]/((1+4*argx*argx)^((z-1)/2)));
py=(Gamma[z-1]/(2*Gamma[z+1]))*(argy^(-2))*(1-Cos[(z-
1)*ArcTan[2*argy]]/((1+4*argy*argy)^((z-1)/2)));
pxy=NIntegrate[px*py, {phi, 0, Pi/2}]/(Pi/2);
pz=(Gamma[z-1]/(2*Gamma[z+1]))*(argz^(-2))*(1-Cos[(z-
1)*ArcTan[2*argz]]/((1+4*argz*argz)^((z-1)/2)));
Pq2=pxy*pz;
argxs=qrs*a*Cos[phi]/(z+1);
argys=qrs*b*Sin[phi]/(z+1);
argzs=qzs*c/(z+1);
pxs=(Gamma[z-1]/(2*Gamma[z+1]))*(argxs^(-2))*(1-Cos[(z-
1)*ArcTan[2*argxs]]/((1+4*argxs*argxs)^((z-1)/2)));
pys=(Gamma[z-1]/(2*Gamma[z+1]))*(argys^(-2))*(1-Cos[(z-
1)*ArcTan[2*argys]]/((1+4*argys*argys)^((z-1)/2)));
pxys=NIntegrate[pxs*pys, {phi, 0, Pi/2}]/(Pi/2);
pzs=(Gamma[z-1]/(2*Gamma[z+1]))*(argzs^(-2))*(1-Cos[(z-
1)*ArcTan[2*argzs]]/((1+4*argzs*argzs)^((z-1)/2)));
Pq2s=pxys*pzs;
lim=1.5;
pl1=Plot[Log[10,Pq1s], {lqs, -1, lim}, PlotRange->{-10,1}, GridLines->{Automatic},
LabelStyle->Directive[Black,16],AxesLabel->{"log q","lg P(q)"}, AxesOrigin->{-1,-
10},TicksStyle->Directive[Black,10],PlotStyle->{Black}];
pl2=Plot[Log[10,Pq2s], {lqs, -1, lim}, PlotRange->{-10,1}, GridLines->{Automatic},
LabelStyle->Directive[Black,16],AxesLabel->{"log q","lg P(q)"}, AxesOrigin->{-1,-
10},TicksStyle->Directive[Black,10],PlotStyle->{Blue, Dashed}] ;
Show[pl1,pl2]
lims=10;
pl4=DensityPlot[Log[10,Pq1],{qx, -lims, lims}, {qy, -lims, lims}, PlotRange->{-8,0},
PlotPoints->50, PlotLegends->Automatic, LabelStyle->Directive[Black,12],AxesLabel-
>Automatic]
pl5=DensityPlot[Log[10,Pq2],{qx, -lims, lims}, {qy, -lims, lims}, PlotRange->{-8,0},
PlotPoints->10, PlotLegends->Automatic, LabelStyle->Directive[Black,12],AxesLabel-
>Automatic]
```

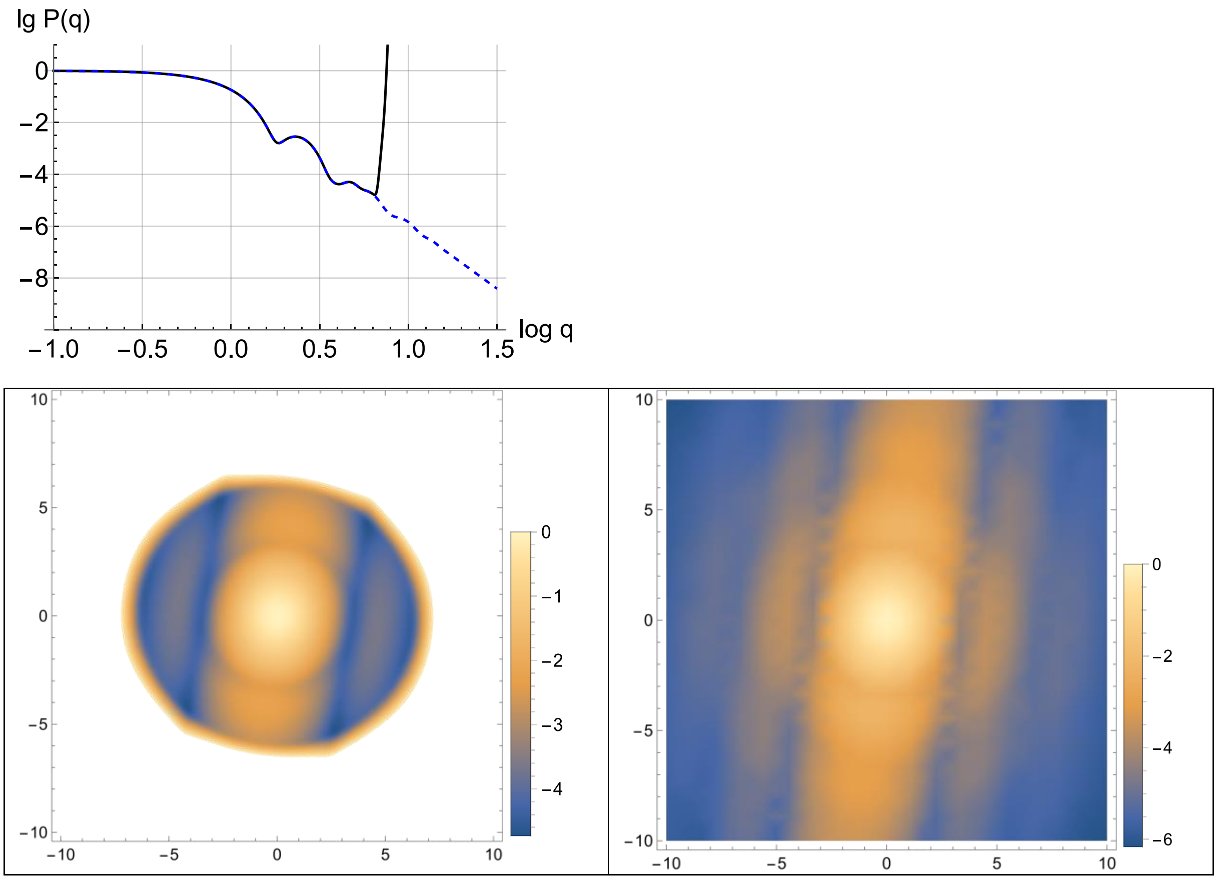

Fig. S45: Formfactor of equatorially averaged polydisperse parallelepipeds.

6.1.4 Scattering amplitude F(q), equatorially averaged

Regime I. For the parallelepiped (S.4.5.2.2) we have the angular-dependent series expansion

$$\langle F(q,\theta,\phi)\rangle^2 = \sum_{n=0}^{\infty}\left(-\frac{q^2a^2(\sin\theta\cos\phi)^2}{4(z+1)^2}\right)^n f_n^{(1)} \sum_{m=0}^{\infty}\left(-\frac{q^2b^2(\sin\theta\sin\phi)^2}{4(z+1)^2}\right)^m f_m^{(1)} \sum_{l=0}^{\infty}\left(-\frac{q^2c^2(\cos\theta)^2}{4(z+1)^2}\right)^l f_l^{(1)}$$

We need to integrate over the angle ϕ to obtain

$$\langle F(q)\rangle^2 = \sum_{n=0}^{\infty}\left(-\frac{q^2a^2(\sin\theta)^2}{4(z+1)^2}\right)^n f_n^{(1)} \sum_{m=0}^{\infty}\left(-\frac{q^2b^2(\sin\theta)^2}{4(z+1)^2}\right)^m f_m^{(1)} \left(\sum_{l=0}^{\infty}\left(-\frac{q^2c^2(\cos\theta)^2}{4(z+1)^2}\right)^l f_l^{(1)}\right)\frac{2}{\pi}\int_0^{\pi/2}(\cos\phi)^{2n}(\sin\phi)^{2m}d\phi$$

We take the integral as solved above to have

$$\langle F(q)\rangle^2 = \sum_{n=0}^{\infty}\left(-\frac{q^2a^2(\sin\theta)^2}{4(z+1)^2}\right)^n f_n^{(1)} \sum_{m=0}^{\infty}\left(-\frac{q^2b^2(\sin\theta)^2}{4(z+1)^2}\right)^m f_m^{(1)} \left(\sum_{l=0}^{\infty}\left(-\frac{q^2c^2(\cos\theta)^2}{4(z+1)^2}\right)^l f_l^{(1)}\right)\frac{\Gamma\left(n+\frac{1}{2}\right)\Gamma\left(m+\frac{1}{2}\right)}{\pi\Gamma(n+m+1)}$$

$$= \frac{1}{\pi}\sum_{n=0}^{\infty}\Gamma\left(n+\frac{1}{2}\right)\left(-\frac{q_r^2a^2}{4(z+1)^2}\right)^n f_n^{(1)} \sum_{m=0}^{\infty}\frac{\Gamma\left(m+\frac{1}{2}\right)}{\Gamma(n+m+1)}\left(-\frac{q_r^2b^2}{4(z+1)^2}\right)^m f_m^{(1)} \left(\sum_{l=0}^{\infty}\left(-\frac{q_z^2c^2}{4(z+1)^2}\right)^l f_l^{(1)}\right)$$

With $b = \delta a$ it can be simplified to **(S.6.1.4.1)**

$$\langle F(q)\rangle^2 = \frac{1}{\pi}\sum_{n=0}^{\infty}\Gamma\left(n+\frac{1}{2}\right)\left(-\frac{q_r^2 a^2}{4(z+1)^2}\right)^n f_n^{(1)} \sum_{m=0}^{\infty}\frac{\Gamma\left(m+\frac{1}{2}\right)}{\Gamma(n+m+1)}\left(-\frac{q_r^2\delta^2 a^2}{4(z+1)^2}\right)^m f_m^{(1)}\left(\sum_{l=0}^{\infty}\left(-\frac{q_z^2 c^2}{4(z+1)^2}\right)^l f_l^{(1)}\right)$$

Parallelepiped, equatorially averaged, F(q)

Regime II. From Eq. (S.4.5.2.4) we have the product of averaged trigonometric functions **(S.6.1.4.2)**

$$\langle F(q)\rangle^2 = \left(\langle\frac{\sin(qc\cos\theta)}{qc\cos\theta}\rangle\right)^2 \frac{2}{\pi}\int_0^{\pi/2}\left(\langle\frac{\sin(qa\sin\theta\cos\phi)}{qa\sin\theta\cos\phi}\rangle\langle\frac{\sin(qb\sin\theta\sin\phi)}{qb\sin\theta\sin\phi}\rangle\right)^2 d\phi$$

with

$$\langle\frac{\sin(q_i x)}{q_i x}\rangle = \frac{\Gamma[z+s]}{\Gamma[z+s+1]}\left(\frac{q_i x}{z+1}\right)^{-1}\frac{\sin\left[(z+s)\arctan\left(\frac{q_i x}{z+1}\right)\right]}{\left(1+\left(\frac{q_i x}{z+1}\right)^2\right)^{\frac{z+s}{2}}}$$

Mathematica code implementation:

```
sigma=0.1;
z=(1-sigma*sigma)/(sigma*sigma);
nmax=80;
fff=Table[Sum[Pochhammer[z+1,2*(n-m)]*Pochhammer[z+1,2*m]/(Pochhammer[3/2,n-m]*Pochhammer[3/2,m]*((n-m)!)*(m!)), {m, 0, n}],{n, 0, nmax}];

a=1;
b=2;
c=3;
theta=0.6*Pi/2;
alf=0.3*Pi/2;
beta=0.2*Pi/2;
gam=0.1*Pi/2;
RRx={{1,0,0},{0,Cos[alf],-Sin[alf]},{0,Sin[alf],Cos[alf]}};
RRy={{Cos[beta],0,Sin[beta]},{0,1,0},{-Sin[beta],0,Cos[beta]}};
RRz={{Cos[gam],-Sin[gam],0},{Sin[gam],Cos[gam],0},{0,0,1}};
RR=RRz.RRy.RRz;
qz=0.001;
qv={qx,qy,qz};
qvs=RR.qv;
qxx=qvs[[1]];
qyy=qvs[[2]];
qzz=qvs[[3]];
qrr=Sqrt[qxx*qxx+qyy*qyy];
qs=10^lqs;
qrs=qs*Sin[theta];
qzs=qs*Cos[theta];
sum1=Sum[(Gamma[n+1/2]*((-qrr*qrr*a*a/(4*(z+1)*(z+1)))^n)*fff[[n+1]])*Sum[Gamma [m+1/2]*((-qrr*qrr*b*b/(4*(z+1)*(z+1)))^m)*fff[[m+1]]/(((n+m)!)), {m, 0, nmax}], {n, 0, nmax}];
sum3=Sum[((-qzz*qzz*c*c/(4*(z+1)*(z+1)))^n)*fff[[n+1]], {n, 0, nmax}];
Fq1=(1/(Pi))*sum1*sum3;
sum1s=Sum[(Gamma[n+1/2]*((-qrs*qrs*a*a/(4*(z+1)*(z+1)))^n)*fff[[n+1]])*Sum[Gamma [m+1/2]*((-qrs*qrs*b*b/(4*(z+1)*(z+1)))^m)*fff[[m+1]]/(((n+m)!)), {m, 0, nmax}], {n, 0, nmax}];
sum3s=Sum[((-qzs*qzs*c*c/(4*(z+1)*(z+1)))^n)*fff[[n+1]], {n, 0, nmax}];
Fq1s=(1/(Pi))*sum1s*sum3s;
argx=qrr*a*Cos[phi]/(z+1);
argy=qrr*b*Sin[phi]/(z+1);
argz=qzz*c/(z+1);
```

```
px=((Gamma[z]/(Gamma[z+1]))*(argx^(-1))*Sin[(z)*ArcTan[argx]]/((1+argx*argx)^((z)/2)))^2;
py=((Gamma[z]/(Gamma[z+1]))*(argy^(-1))*Sin[(z)*ArcTan[argy]]/((1+argy*argy)^((z)/2)))^2;
pxy=NIntegrate[px*py, {phi, 0, Pi/2}]/(Pi/2);
pz=((Gamma[z]/(Gamma[z+1]))*(argz^(-1))*Sin[(z)*ArcTan[argz]]/((1+argz*argz)^((z)/2)))^2;
Fq2=pxy*pz;
argxs=qrs*a*Cos[phi]/(z+1);
argys=qrs*b*Sin[phi]/(z+1);
argzs=qzs*c/(z+1);
pxs=((Gamma[z]/(Gamma[z+1]))*(argxs^(-
1))*Sin[(z)*ArcTan[argxs]]/((1+argxs*argxs)^((z)/2)))^2;
pys=((Gamma[z]/(Gamma[z+1]))*(argys^(-
1))*Sin[(z)*ArcTan[argys]]/((1+argys*argys)^((z)/2)))^2;
pxys=NIntegrate[pxs*pys, {phi, 0, Pi/2}]/(Pi/2);
pzs=((Gamma[z]/(Gamma[z+1]))*(argzs^(-
1))*Sin[(z)*ArcTan[argzs]]/((1+argzs*argzs)^((z)/2)))^2;
Fq2s=pxys*pzs;
lim=1.5;
pl1=Plot[Log[10,Fq1s], {lqs, -1, lim}, PlotRange->{-10,1}, GridLines->{Automatic},
LabelStyle->Directive[Black,16],AxesLabel->{"log q","lg F^2(q)"}, AxesOrigin->{-1,-
10},TicksStyle->Directive[Black,10],PlotStyle->{Black}];
pl2=Plot[Log[10,Fq2s], {lqs, -1, lim}, PlotRange->{-10,1}, GridLines->{Automatic},
LabelStyle->Directive[Black,16],AxesLabel->{"log q","lg F^2(q)"}, AxesOrigin->{-1,-
10},TicksStyle->Directive[Black,10],PlotStyle->{Blue, Dashed}];
Show[pl1,pl2]
lims=10;
pl4=DensityPlot[Log[10,Fq1],{qx, -lims, lims}, {qy, -lims, lims}, PlotRange->{-8,0},
PlotPoints->50, PlotLegends->Automatic, LabelStyle->Directive[Black,12],AxesLabel-
>Automatic]
pl5=DensityPlot[Log[10,Fq2],{qx,  -lims,  lims},  {qy,  -lims,  lims},  PlotRange->{-8,0},
PlotPoints->20,     PlotLegends->Automatic,     LabelStyle->Directive[Black,12],AxesLabel-
>Automatic]
```

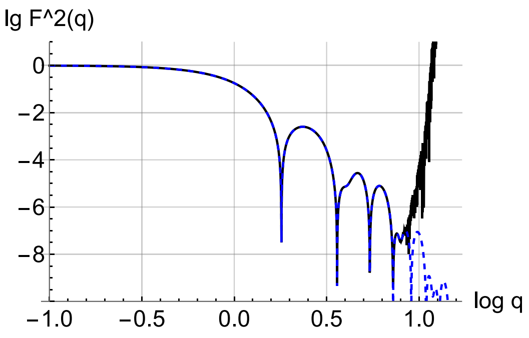

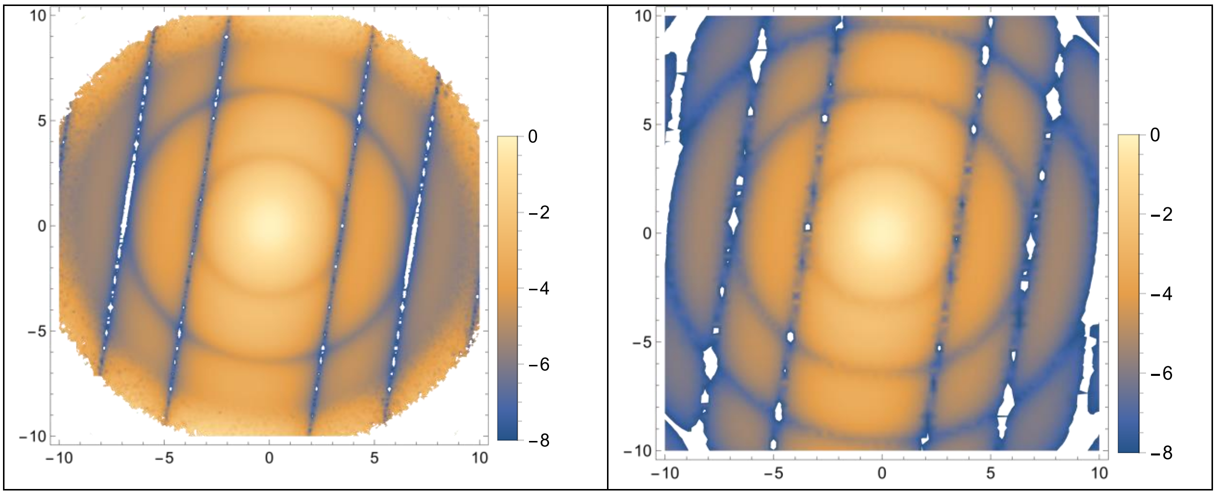

Fig. S46: Scattering amplitude of equatorially averaged polydisperse parallelepipeds.

6.2 Cylinders and Disks

6.2.1 Formfactor P(q)

Regime I. From Eq. (S.1.2.50, S.1.2.53) we have for perfectly oriented cylinders or disks the series expansion **(S.6.2.1.1)**

$$P(q) = \sum_{n=0}^{\infty} \frac{4^n}{(n+1)} \frac{(z_L+s_L+1)_{2n}}{\left(\frac{3}{2}\right)_n n!} \left(-\frac{q_z^2 L^2}{4(z_L+1)^2}\right)^n \sum_{m=0}^{\infty} \frac{4^{m+1}\Gamma\left(m+\frac{3}{2}\right)}{\sqrt{\pi}\,\Gamma(m+3)} \frac{(z_R+s_R+1)_{2m}}{(2)_m m!} \left(-\frac{q_r^2 R^2}{4(z_R+1)^2}\right)^m$$

Cylinders, disks, polydisperse, perfect orientation, P(q)

Regime II. From Eq. (S.1.2.49, S.1.2.52) we have the product of averaged trigonometric functions **(S.6.2.1.2)**

$$P(q) = \left\langle \left(\frac{\sin(qL\cos\theta)}{qL\cos\theta}\right)^2 \right\rangle_L \left\langle \left(\frac{2J_1(qR\sin\theta)}{qR\sin\theta}\right)^2 \right\rangle_R$$

with

$$\left\langle \left(\frac{\sin(q_x a)}{q_x a}\right)^2 \right\rangle_a = \frac{\Gamma[z+s-1]}{\Gamma[z+s+1]} \frac{1}{2} u^{-2} \left(1 - \frac{\cos[(z+s-n+1)\arctan(2u)]}{(1+4u^2)^{\frac{z+s-1}{2}}}\right)$$

$$u = \frac{q_i x}{z+1}$$

and

$$\left\langle \frac{4(J_1(qR\sin\theta)^2}{q^2(R\sin\theta)^2} \right\rangle = \frac{4}{\pi}\left(\left(\frac{1}{qR}\right)^3 - \left(\frac{1}{qR}\right)^3 \sin(2qR) - \frac{9}{8}\left(\frac{1}{qR}\right)^4 \cos(2qR) + \left(\frac{9}{16}\right)^2 \left(\frac{1}{qR}\right)^5 + \left(\frac{9}{16}\right)^2 \left(\frac{1}{qR}\right)^5 \sin(2qR)\right)$$

$$\langle (qR)^{-3} \rangle = \frac{\Gamma[z+s-2]}{\Gamma[z+s+1]} \left(\frac{qR}{z+1}\right)^{-3}$$

$$\left\langle \frac{\sin(2qR)}{(qR)^3} \right\rangle = \frac{\Gamma[z+s-2]}{\Gamma[z+s+1]} \left(\frac{qR}{z+1}\right)^{-3} \frac{\sin\left[(z+s-2)\arctan\left(\frac{2qR}{z+1}\right)\right]}{\left(1+\left(\frac{2qR}{z+1}\right)^2\right)^{\frac{z+s-2}{2}}}$$

$$\left\langle \frac{\cos(2qR)}{(qR)^4} \right\rangle = \frac{\Gamma[z+s-3]}{\Gamma[z+s+1]} \left(\frac{qR}{z+1}\right)^{-4} \frac{\cos\left[(z+s-3)\arctan\left(\frac{2qR}{z+1}\right)\right]}{\left(1+\left(\frac{2qR}{z+1}\right)^2\right)^{\frac{z+s-3}{2}}}$$

$$\langle (qR)^{-5} \rangle = \frac{\Gamma[z+s-4]}{\Gamma[z+s+1]} \left(\frac{x}{z+1}\right)^{-4}$$

$$\langle\frac{\sin(2qR)}{(qR)^5}\rangle = \frac{\Gamma[z+s-4]}{\Gamma[z+s+1]}\left(\frac{qR}{z+1}\right)^{-5}\frac{\sin\left[(z+s-4)\arctan\left(\frac{2qR}{z+1}\right)\right]}{\left(1+\left(\frac{2qR}{z+1}\right)^2\right)^{\frac{z+s-4}{2}}}$$

Regime III. The high-q Porod asymptotes are **(S.6.2.1.3)**

$$\langle\left(\frac{\sin(q_x a)}{q_x a}\right)^2\rangle_a = \frac{\Gamma[z+s-1]}{\Gamma[z+s+1]}\frac{1}{2}u^{-2}$$

$$\langle\frac{4(J_1(qR\sin\theta)^2}{q^2(R\sin\theta)^2}\rangle = \frac{4}{\pi}\left(\frac{1}{qR}\right)^3$$

$$\langle(qR)^{-3}\rangle = \frac{\Gamma[z+s-2]}{\Gamma[z+s+1]}\left(\frac{qR}{z+1}\right)^{-3}$$

Mathematica code implementation:

```
L=2;
R=1;
sigma=0.1;
z=(1-sigma*sigma)/(sigma*sigma);
nmax=80;
alf=0.3*Pi/2;
beta=0.2*Pi/2;
gam=0.1*Pi/2;
RRx={{1,0,0},{0,Cos[alf],-Sin[alf]},{0,Sin[alf],Cos[alf]}};
RRy={{Cos[beta],0,Sin[beta]},{0,1,0},{-Sin[beta],0,Cos[beta]}};
RRz={{Cos[gam],-Sin[gam],0},{Sin[gam],Cos[gam],0},{0,0,1}};
RR=RRz.RRy.RRz;
qqz=0.001;
qv={qqx,qqy,qqz};
qvs=RR.qv;
qqxs=qvs[[1]];
qqys=qvs[[2]];
qqzs=qvs[[3]];
qqrs=Sqrt[qqxs*qqxs+qqys*qqys];

qs=10^lqs;
theta=0.3*Pi/2;
qzs=qs*Cos[theta];
qrs=qs*Sin[theta];
Pql1=Sum[((4^n)*Pochhammer[z+1,2*n]*((-
qqzs*qqzs*L*L/(4*(z+1)*(z+1)))^n)/((n+1)*Pochhammer[3/2,n]*(n!))),{n,0,nmax}];
Pql1s=Sum[((4^n)*Pochhammer[z+1,2*n]*((-
qzs*qzs*L*L/(4*(z+1)*(z+1)))^n)/((n+1)*Pochhammer[3/2,n]*(n!))),{n,0,nmax}];
Pql1ss=Sum[((4^n)*Pochhammer[z+1,2*n]*((-
qzss*qzss*L*L/(4*(z+1)*(z+1)))^n)/((n+1)*Pochhammer[3/2,n]*(n!))),{n,0,nmax}];
Pqr1=Sum[((4^(m+1))*Gamma[m+3/2]*Pochhammer[z+1,2*m]*((-
qqrs*qqrs*R*R/(4*(z+1)*(z+1)))^m)/(Sqrt[Pi]*Gamma[m+3]*Pochhammer[2,m]*(m!))),{m,0,nmax}];
Pqr1s=Sum[((4^(m+1))*Gamma[m+3/2]*Pochhammer[z+1,2*m]*((-
qrs*qrs*R*R/(4*(z+1)*(z+1)))^m)/(Sqrt[Pi]*Gamma[m+3]*Pochhammer[2,m]*(m!))),{m,0,nmax}];
Pqr1ss=Sum[((4^(m+1))*Gamma[m+3/2]*Pochhammer[z+1,2*m]*((-
qrss*qrss*R*R/(4*(z+1)*(z+1)))^m)/(Sqrt[Pi]*Gamma[m+3]*Pochhammer[2,m]*(m!))),{m,0,nmax}];
ql=qqzs*L/(z+1);
qr=qqrs*R/(z+1);
```

```
qls=qzs*L/(z+1);
qrs=qrs*R/(z+1);
Pql2=(Gamma[z-1]/(2*Gamma[z+1]))*(ql^(-2))*(1-Cos[(z-1)*ArcTan[2*ql]]/((1+4*ql*ql)^((z-1)/2)));
Pql2s=(Gamma[z-1]/(2*Gamma[z+1]))*(qls^(-2))*(1-Cos[(z-1)*ArcTan[2*qls]]/((1+4*qls*qls)^((z-1)/2)));
Pql2sa=(Gamma[z-1]/(2*Gamma[z+1]))*(qls^(-2));
fr1=(Gamma[z-2]/(Gamma[z+1]))*(qr^(-3));
fr2=(Gamma[z-2]/(Gamma[z+1]))*(qr^(-3))*Sin[(z-2)*ArcTan[2*qr]]/((1+4*qr*qr)^((z-2)/2));
fr3=(Gamma[z-3]/(Gamma[z+1]))*(qr^(-4))*Cos[(z-3)*ArcTan[2*qr]]/((1+4*qr*qr)^((z-3)/2));
Pqr2=(4/Pi)*(fr1-fr2-(9/8)*fr3);
fr1s=(Gamma[z-2]/(Gamma[z+1]))*(qrs^(-3));
fr2s=(Gamma[z-2]/(Gamma[z+1]))*(qrs^(-3))*Sin[(z-2)*ArcTan[2*qrs]]/((1+4*qrs*qrs)^((z-2)/2));
fr3s=(Gamma[z-3]/(Gamma[z+1]))*(qrs^(-4))*Cos[(z-3)*ArcTan[2*qrs]]/((1+4*qrs*qrs)^((z-3)/2));
Pqr2s=(4/Pi)*(fr1s-fr2s-(9/8)*fr3s);
Pqr2sa=(4/Pi)*fr1s;
lim=1.5;
pl1=Plot[Log[10,Pql1s],{lqs,-1,lim},PlotRange->{-6,1},GridLines->{Automatic},LabelStyle->Directive[Black,16],AxesLabel->{"log q","lg P(q)"},AxesOrigin->{-1,-6},TicksStyle->Directive[Black,12],PlotStyle->{Black}];
pl2=Plot[Log[10,Pql2s],{lqs,-1,lim},PlotRange->{-6,1},GridLines->{Automatic},LabelStyle->Directive[Black,16],AxesLabel->{"log q","lg P(q)"},AxesOrigin->{-1,-6},TicksStyle->Directive[Black,12],PlotStyle->{Blue,Dashed}];
pl3=Plot[Log[10,Pql2sa],{lqs,-1,lim},PlotRange->{-6,1},GridLines->{Automatic},LabelStyle->Directive[Black,16],AxesLabel->{"log q","lg P(q)"},AxesOrigin->{-1,-6},TicksStyle->Directive[Black,12],PlotStyle->{Red}];
pl4=Plot[Log[10,Pqr1s],{lqs,-1,lim},PlotRange->{-6,1},GridLines->{Automatic},LabelStyle->Directive[Black,16],AxesLabel->{"log q","lg P(q)"},AxesOrigin->{-1,-6},TicksStyle->Directive[Black,10],PlotStyle->{Black}];
pl5=Plot[Log[10,Pqr2s],{lqs,-1,lim},PlotRange->{-6,1},GridLines->{Automatic},LabelStyle->Directive[Black,16],AxesLabel->{"log q","lg P(q)"},AxesOrigin->{-1,-6},TicksStyle->Directive[Black,10],PlotStyle->{Blue,Dashed}];
pl6=Plot[Log[10,Pqr2sa],{lqs,-1,lim},PlotRange->{-6,1},GridLines->{Automatic},LabelStyle->Directive[Black,16],AxesLabel->{"log q","lg P(q)"},AxesOrigin->{-1,-6},TicksStyle->Directive[Black,10],PlotStyle->{Red}];
Show[pl1,pl3,pl2]
Show[pl4,pl6,pl5]
lims=15;
pl7=DensityPlot[Log[10,Pql1*Pqr1],{qqx,-lims,lims},{qqy,-lims,lims},PlotRange->{-8,0},PlotPoints->50,PlotLegends->Automatic,LabelStyle->Directive[Black,12],AxesLabel->Automatic]
pl8=DensityPlot[Log[10,Pql2*Pqr2],{qqx,-lims,lims},{qqy,-lims,lims},PlotRange->{-8,0},PlotPoints->50,PlotLegends->Automatic,LabelStyle->Directive[Black,12],AxesLabel->Automatic]
limss=8;
pl9=DensityPlot[Log[10,Pql1ss*Pqr1ss],{qzss,-limss,limss},{qrss,-limss,limss},PlotRange->{-8,0},PlotPoints->50,PlotLegends->Automatic,LabelStyle->Directive[Black,12],AxesLabel->Automatic]
```

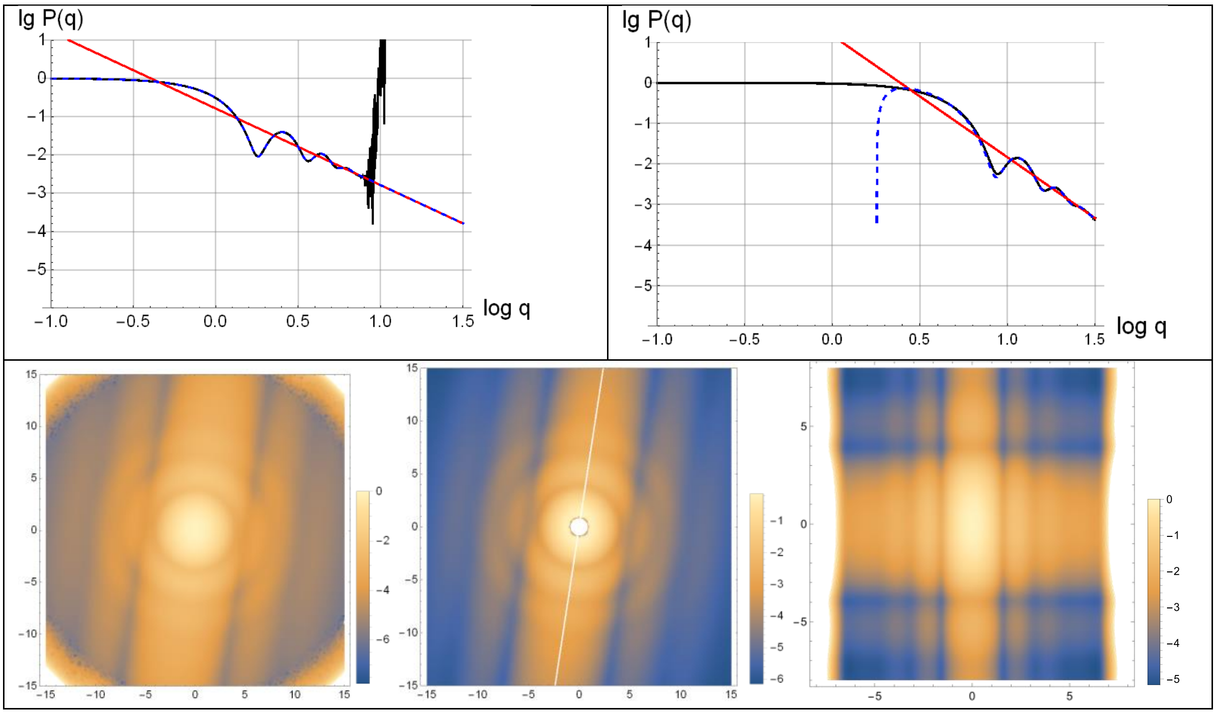

Fig. S47: Formfactor of perfectly oriented polydisperse cylinders, with 2D-plots in an oblique orientation (left, center) and a perfect in plane orientation (right).

6.2.2 Scattering Amplitude F(q)

Regime I. From Eq. (S.1.2.30, S.1.2.33) we have the series expansion **(S.6.2.2.1)**

$$\langle F_{cyl}(q)\rangle_{L,R}^2 = \left(\langle F^{(1)}(q_z L)\rangle_L \langle F^{(2)}(q_r R)\rangle_R\right)^2$$

$$\left(\langle F^{(1)}(q_z L)\rangle_L\right)^2 = \sum_{n=0}^{\infty}\left(-\frac{(qL\cos\theta)^2}{4(z_L+1)^2}\right)^n \sum_{m=0}^{n} \frac{(z_L+s_L+1)_{2(n-m)}(z_L+s_L+1)_{2m}}{\left(\frac{3}{2}\right)_{n-m}(n-m)!\left(\frac{3}{2}\right)_n n!}$$

$$\left(\langle F^{(2)}(q_r R)\rangle_R\right)^2 = \sum_{n=0}^{\infty}\left(-\frac{(qR\sin\theta)^2}{4(z_R+1)^2}\right)^n \sum_{m=0}^{n} \frac{(z_R+s_R+1)_{2(n-m)}(z_R+s_R+1)_{2m}}{(2)_{n-m}(n-m)!\,(2)_n n!}$$

Cylinder, disks, polydisperse, perfect orientation, F(q)

Regime II. From Eqs. (S.1.2.29, S.1.2.32) we have the product of averaged trigonometric functions **(S.6.2.2.2)**

$$F(q) = \langle\frac{2J_1(qR)}{qR}\rangle^2 \langle\frac{\sin(qL\cos\theta)}{qL\cos\theta}\rangle^2$$

$$\langle\frac{\sin(q_x a)}{q_x a}\rangle = \frac{\Gamma[z+s-1]}{\Gamma[z+s+1]}\left(\frac{q_x a}{z+1}\right)^{-1} \frac{\sin\left[(z+s-1)\arctan\left(\frac{q_x a}{z+1}\right)\right]}{\left(1+\left(\frac{q_x a}{z+1}\right)^2\right)^{\frac{z+s-1}{2}}}$$

$$F(q) = \frac{2}{\sqrt{\pi}}\left(\langle\frac{\sin(qR)}{(qR)^{3/2}}\rangle_R - \langle\frac{\cos(qR)}{(qR)^{3/2}}\rangle_R + \frac{9}{16}\left(\langle\frac{\cos(qR)}{(qR)^{5/2}}\rangle_R + \langle\frac{\sin(qR)}{(qR)^{5/2}}\rangle_R\right)\right)$$

$$\langle(qR)^{-\frac{3}{2}}\sin(qR)\rangle = \frac{\Gamma[z-\frac{1}{2}]}{\Gamma[z+1]}\left(\frac{qR}{z+1}\right)^{-3/2} \frac{\sin\left[\left(z-\frac{1}{2}\right)\arctan\left(\frac{qR}{z+1}\right)\right]}{\left(1+\left(\frac{qR}{z+1}\right)^2\right)^{\frac{z-\frac{1}{2}}{2}}}$$

$$\langle(qR)^{-\frac{3}{2}}\cos(qR)\rangle = \frac{\Gamma[z-\frac{1}{2}]}{\Gamma[z+1]}\left(\frac{qR}{z+1}\right)^{-3/2} \frac{\cos\left[\left(z-\frac{1}{2}\right)\arctan\left(\frac{qR}{z+1}\right)\right]}{\left(1+\left(\frac{qR}{z+1}\right)^2\right)^{\frac{z-\frac{1}{2}}{2}}}$$

$$\langle (qR)^{-\frac{5}{2}}\sin(qR)\rangle = \frac{\Gamma[z-\frac{3}{2}]}{\Gamma[z+1]}\left(\frac{qR}{z+1}\right)^{-5/2}\frac{\sin\left[\left(z-\frac{3}{2}\right)\arctan\left(\frac{qR}{z+1}\right)\right]}{\left(1+\left(\frac{qR}{z+1}\right)^2\right)^{\frac{z-\frac{3}{2}}{2}}}$$

$$\langle (qR)^{-\frac{5}{2}}\cos(qR)\rangle = \frac{\Gamma[z-\frac{3}{2}]}{\Gamma[z+1]}\left(\frac{qR}{z+1}\right)^{-5/2}\frac{\cos\left[\left(z-\frac{3}{2}\right)\arctan\left(\frac{qR}{z+1}\right)\right]}{\left(1+\left(\frac{qR}{z+1}\right)^2\right)^{\frac{z-\frac{3}{2}}{2}}}$$

Mathematica code implementation:

```
L=2;
R=1;
sigma=0.1;
z=(1-sigma*sigma)/(sigma*sigma);
nmax=100;
alf=0.3*Pi/2;
beta=0.2*Pi/2;
gam=0.1*Pi/2;
RRx={{1,0,0},{0,Cos[alf],-Sin[alf]},{0,Sin[alf],Cos[alf]}};
RRy={{Cos[beta],0,Sin[beta]},{0,1,0},{-Sin[beta],0,Cos[beta]}};
RRz={{Cos[gam],-Sin[gam],0},{Sin[gam],Cos[gam],0},{0,0,1}};
RR=RRz.RRy.RRz;
qqz=0.001;
qv={qqx,qqy,qqz};
qvs=RR.qv;
qqxs=qvs[[1]];
qqys=qvs[[2]];
qqzs=qvs[[3]];
qqrs=Sqrt[qqxs*qqxs+qqys*qqys];

qs=10^lqs;
theta=0.3*Pi/2;
qzs=qs*Cos[theta];
qrs=qs*Sin[theta];
Fql1=Sum[((-qqzs*qqzs*L*L/(4*(z+1)*(z+1)))^n)*Sum[Pochhammer[z+1,2*m]*Pochhammer[z+1,2*(n-
m)]/(Pochhammer[3/2,n-m]*Pochhammer[3/2,m]*((n-m)!)*(m!)),{m, 0, n}], {n, 0, nmax}];
Fql1s=Sum[((-qzs*qzs*L*L/(4*(z+1)*(z+1)))^n)*Sum[Pochhammer[z+1,2*m]*Pochhammer[z+1,2*(n-
m)]/(Pochhammer[3/2,n-m]*Pochhammer[3/2,m]*((n-m)!)*(m!)),{m, 0, n}], {n, 0, nmax}];
Fqr1=Sum[((-qqrs*qqrs*R*R/(4*(z+1)*(z+1)))^n)*Sum[Pochhammer[z+1,2*m]*Pochhammer[z+1,2*(n-
m)]/(Pochhammer[2,n-m]*Pochhammer[2,m]*((n-m)!)*(m!)),{m, 0, n}], {n, 0, nmax}];
Fqr1s=Sum[((-qrs*qrs*R*R/(4*(z+1)*(z+1)))^n)*Sum[Pochhammer[z+1,2*m]*Pochhammer[z+1,2*(n-
m)]/(Pochhammer[2,n-m]*Pochhammer[2,m]*((n-m)!)*(m!)),{m, 0, n}], {n, 0, nmax}];
ql=qqzs*L/(z+1);
qr=qqrs*R/(z+1);
qls=qzs*L/(z+1);
qrs=qrs*R/(z+1);
Fql2=((Gamma[z]/(Gamma[z+1]))*(ql^(-1))*Sin[(z)*ArcTan[ql]]/((1+ql*ql)^((z)/2)))^2;
Fql2s=((Gamma[z]/(Gamma[z+1]))*(qls^(-1))*Sin[(z)*ArcTan[qls]]/((1+qls*qls)^((z)/2)))^2;
fcrossa=(Gamma[z-1/2]/Gamma[z+1])*(qr^(-(3/2)))*Sin[(z-1/2)*ArcTan[qr]]/((1+qr*qr)^((z-1/2)/2));
fcrossb=(Gamma[z-1/2]/Gamma[z+1])*(qr^(-(3/2)))*Cos[(z-1/2)*ArcTan[qr]]/((1+qr*qr)^((z-1/2)/2));
fcrossc=(Gamma[z-3/2]/Gamma[z+1])*(qr^(-(5/2)))*Sin[(z-3/2)*ArcTan[qr]]/((1+qr*qr)^((z-3/2)/2));
fcrossd=(Gamma[z-3/2]/Gamma[z+1])*(qr^(-(5/2)))*Cos[(z-3/2)*ArcTan[qr]]/((1+qr*qr)^((z-3/2)/2));
Fqr2=((2/Sqrt[Pi])*(fcrossa-fcrossb+(9/16)*(fcrossc+fcrossd)))^2;
fcrossas=(Gamma[z-1/2]/Gamma[z+1])*(qrs^(-(3/2)))*Sin[(z-1/2)*ArcTan[qrs]]/((1+qrs*qrs)^((z-1/2)/2));
fcrossbs=(Gamma[z-1/2]/Gamma[z+1])*(qrs^(-(3/2)))*Cos[(z-1/2)*ArcTan[qrs]]/((1+qrs*qrs)^((z-1/2)/2));
```

```
fcrosscs=(Gamma[z-3/2]/Gamma[z+1])*(qrs^(-(5/2)))*Sin[(z-3/2)*ArcTan[qrs]]/((1+qrs*qrs)^((z-3/2)/2));
fcrossds=(Gamma[z-3/2]/Gamma[z+1])*(qrs^(-(5/2)))*Cos[(z-3/2)*ArcTan[qrs]]/((1+qrs*qrs)^((z-3/2)/2));
Fqr2s=((2/Sqrt[Pi])*(fcrossas-fcrossbs+(9/16)*(fcrosscs+fcrossds)))^2;
lim=1.5;
pl1=Plot[Log[10,Fql1s], {lqs, -1,lim}, PlotRange->{-6,1}, GridLines->{Automatic}, LabelStyle->Directive[Black,16],AxesLabel->{"log q","lg P(q)"}, AxesOrigin->{-1,-6},TicksStyle->Directive[Black,12],PlotStyle->{Black}] ;
pl2=Plot[Log[10,Fql2s], {lqs, -1,lim}, PlotRange->{-6,1}, GridLines->{Automatic}, LabelStyle->Directive[Black,16],AxesLabel->{"log q","lg F^2(q)"}, AxesOrigin->{-1,-6},TicksStyle->Directive[Black,12],PlotStyle->{Blue,Dashed}] ;
pl4=Plot[Log[10,Fqr1s], {lqs , -1,lim}, PlotRange->{-6,1}, GridLines->{Automatic}, LabelStyle->Directive[Black,16],AxesLabel->{"log q","lg F^2(q)"}, AxesOrigin->{-1,-6},TicksStyle->Directive[Black,10],PlotStyle->{Black}] ;
pl5=Plot[Log[10,Fqr2s], {lqs , -1,lim}, PlotRange->{-6,1}, GridLines->{Automatic}, LabelStyle->Directive[Black,16],AxesLabel->{"log q","lg F^2(q)"}, AxesOrigin->{-1,-6},TicksStyle->Directive[Black,10],PlotStyle->{Blue, Dashed}] ;
Show[pl1,pl2]
Show[pl4,pl5]
lims=15;
pl7=DensityPlot[Log[10,Fql1*Fqr1],{qqx, -lims, lims}, {qqy, -lims, lims}, PlotRange->{-8,0}, PlotPoints->50, PlotLegends->Automatic, LabelStyle->Directive[Black,12],AxesLabel->Automatic]
pl8=DensityPlot[Log[10,Fql2*Fqr2],{qqx, -lims, lims}, {qqy, -lims, lims}, PlotRange->{-8,0}, PlotPoints->50, PlotLegends->Automatic, LabelStyle->Directive[Black,12],AxesLabel->Automatic]
```

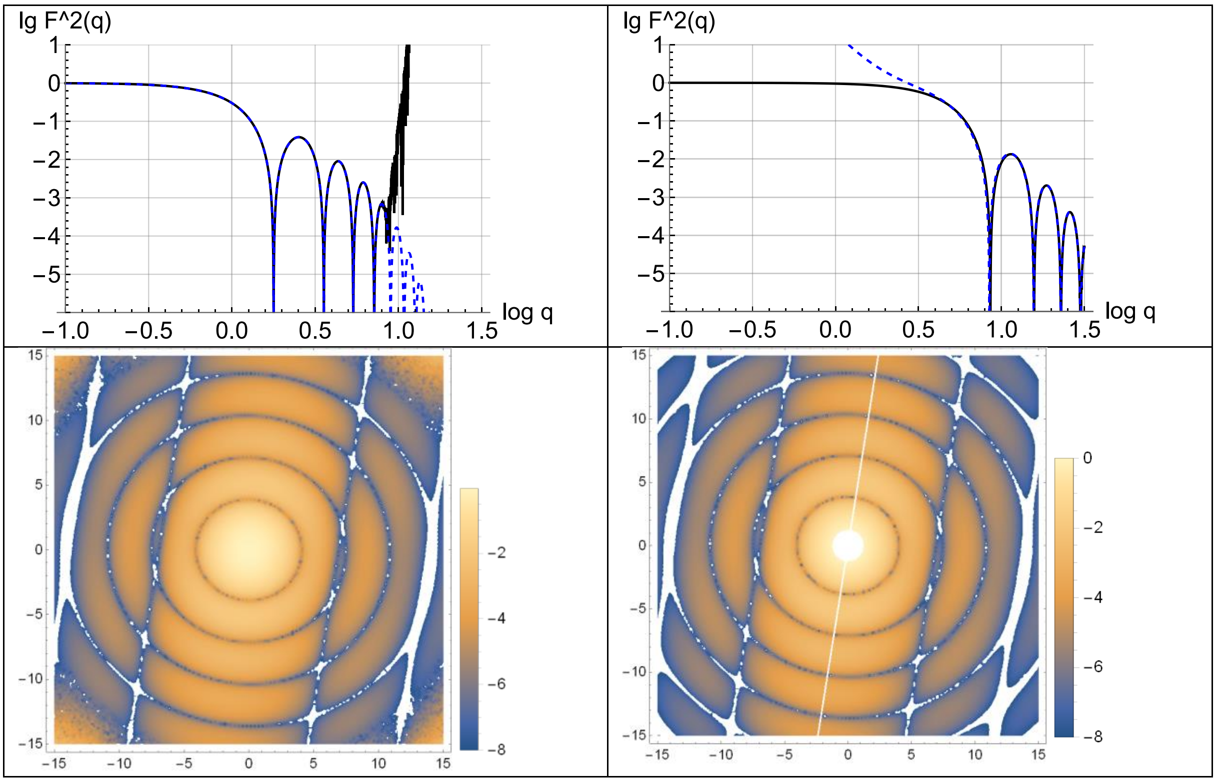

Fig. S48: Scattering amplitude of perfectly oriented polydisperse cylinders.

6.3 Biaxial Ellipsoid

The perfectly oriented biaxial ellipsoid corresponds to the case of the superellipsoid with $k = 2$.

6.3.1 Formfactor P(q)

Regime I. From Eq. (S.4.9.1.5) we have the series expansion

$$P(q)=\left(\frac{\Gamma\left(\frac{k+3}{k}\right)}{\Gamma\left(\frac{2+k}{k}\right)\Gamma\left(\frac{1}{k}\right)}\right)^{2}\sum_{n=0}^{\infty}(z+1)_{2n}\left(-\frac{q_z^2L^2}{4(z+1)^2}\right)^{n}\sum_{m=0}^{\infty}(z+1)_{2n}\left(-\frac{q_r^2R^2}{4(z+1)^2}\right)^{m}c_{n,m}$$

which in the special case $k=2$ yields **(S.6.3.1.1)**

$$P(q)=\left(\frac{9}{16}\right)\sum_{n=0}^{\infty}(z+1)_{2n}\left(-\frac{q_z^2L^2}{4(z+1)^2}\right)^{n}\sum_{m=0}^{\infty}(z+1)_{2n}\left(-\frac{q_r^2R^2}{4(z+1)^2}\right)^{m}c_{n,m}$$

with

$$c_{n,m}=\pi\sum_{n\prime=0}^{n}\frac{1}{(n-n')!\,n'!}\sum_{m\prime=0}^{m}\frac{1}{(m-m')!\,m'!}\frac{1}{\Gamma\left(m-m'+n-n'+\frac{5}{2}\right)\Gamma\left(m'+n'+\frac{5}{2}\right)}$$

Biaxial ellipsoid, polydisperse, perfect orientation, P(q)

Regime III. There is a direct overlap to the Porod regime with **(S.6.3.1.2)**

$$\lim_{q\to\infty}P(q)=\frac{\Gamma(z-3)(z+1)^4}{\Gamma(z+1)}\frac{9}{2((q_rR)^2+(q_zL)^2)^{4/2}}$$

Mathematica code implementation:

```
na=80;
ma=na;
ff=Table[Pi*Sum[(1/((ns!)*((n-ns)!)))*Sum[(1/((ms!)*((m-ms)!)))*(1/(Gamma[ns+ms+5/2]*Gamma[n-ns+m-ms+5/2])),{ms,0,m}],{ns,0,n}],{n,0,na},{m,0,ma}];

R=1;
L=2;
sigma=0.1;
z=(1-sigma*sigma)/(sigma*sigma);
alf=0.3*Pi/2;
beta=0.2*Pi/2;
gam=0.1*Pi/2;
RRx={{1,0,0},{0,Cos[alf],-Sin[alf]},{0,Sin[alf],Cos[alf]}};
RRy={{Cos[beta],0,Sin[beta]},{0,1,0},{-Sin[beta],0,Cos[beta]}};
RRz={{Cos[gam],-Sin[gam],0},{Sin[gam],Cos[gam],0},{0,0,1}};
RR=RRz.RRy.RRz;
qqz=0.001;
qv={qqx,qqy,qqz};
qvs=RR.qv;
qqxs=qvs[[1]];
qqys=qvs[[2]];
qqzs=qvs[[3]];
qqrs=Sqrt[qqxs*qqxs+qqys*qqys];

qs=10^lqs;
theta=0.2*Pi/2;
qzs=qs*Cos[theta];
qrs=qs*Sin[theta];
a1=9/16;
```

```
apor=Gamma[z-3]*((z+1)^4)/Gamma[z+1];
Pqav=a1*Sum[(Pochhammer[z+1,2*n]*((-qqzs*qqzs*L*L/(4*(z+1)*(z+1)))^n))*Sum[Pochhammer[z+1,2*m]*(((-
qqrs*qqrs*R*R/(4*(z+1)*(z+1)))^m))*ff[[n+1,m+1]],{m,0,ma}],{n,0,na}];
Pqavs=a1*Sum[(Pochhammer[z+1,2*n]*((-qzs*qzs*L*L/(4*(z+1)*(z+1)))^n))*Sum[Pochhammer[z+1,2*m]*(((-
qrs*qrs*R*R/(4*(z+1)*(z+1)))^m))*ff[[n+1,m+1]],{m,0,ma}],{n,0,na}];
Rkk=Sqrt[((R*Sin[theta])^2)+((L*Cos[theta])^2)];
Pqpor=(9*apor /2)/((qs^4)*(Rkk^4));
lim=1.5;
pl1=Plot[Log[10,Pqavs], {lqs, -1, lim}, PlotRange->{-6,1}, GridLines->{Automatic}, LabelStyle->Directive[Black,16],AxesLabel-
>{"log q","lg P(q)"}, AxesOrigin->{-1,-6},TicksStyle->Directive[Black,10],PlotStyle->{Blue}];
pl2=Plot[Log[10,Pqpor], {lqs, -1, lim}, PlotRange->{-6,1}, GridLines->{Automatic}, LabelStyle->Directive[Black,16],AxesLabel-
>{"log q","lg P(q)"}, AxesOrigin->{-1,-6},TicksStyle->Directive[Black,10],PlotStyle->{Red}];
Show[pl1,pl2]
lims=8;
pl5=DensityPlot[Log[10,Pqav],{qqx, -lims, lims}, {qqy, -lims, lims}, PlotRange->{-8,0}, PlotPoints->50, PlotLegends-
>Automatic, LabelStyle->Directive[Black,12],AxesLabel->Automatic]
```

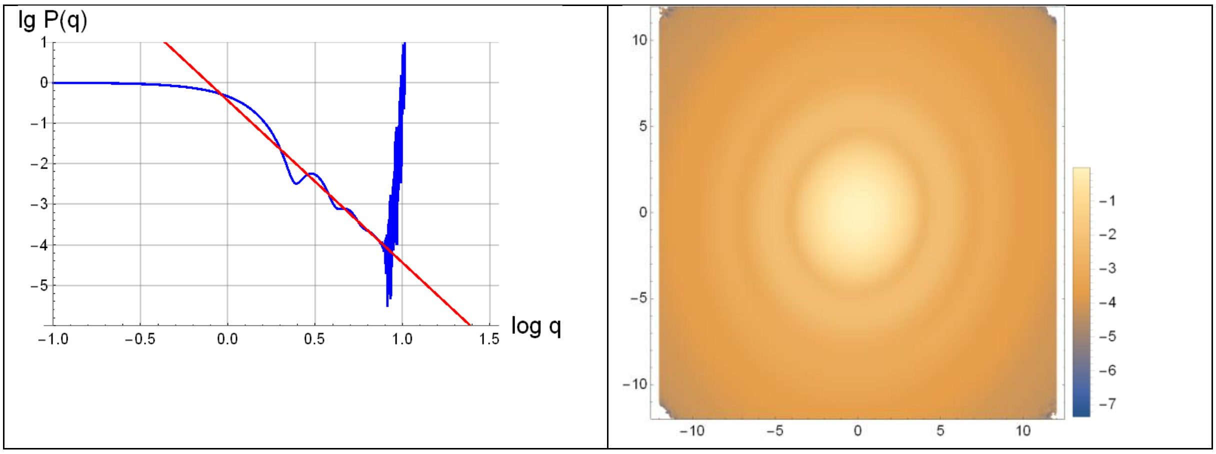

Fig. S49: Formfactor of perfectly oriented biaxial polydisperse ellipsoids.

6.3.2 Scattering amplitude F(q)

We can derive an expression for the scattering amplitude, but it only serves to describe the low-q regime.

Regime I. From Eq. (S.4.9.1.4) we have the series expansion **(S.6.3.2.1)**

$$\langle F(q)\rangle^2 = \left(\frac{9}{16}\right)\sum_{n=0}^{\infty}\left(-\frac{q_z^2L^2}{4(z+1)^2}\right)^n\sum_{m=0}^{\infty}\left(-\frac{q_r^2R^2}{4(z+1)^2}\right)^m f_{n,m}$$

$$f_n = \sum_{n'=0}^{n}\frac{\Gamma\left(\frac{2(n-n')+1}{k}\right)(z+1)_{2(n-n')}}{\left(\frac{1}{2}\right)_{n-n'}(n-n')!}\frac{\Gamma\left(\frac{2n'+1}{k}\right)(z+1)_{2n'}}{\left(\frac{1}{2}\right)_{n'}n'!}\sum_{m'=0}^{m}\frac{(z+1)_{2(m-m')}}{(2)_{m-m'}(m-m')!}\frac{(z+1)_{2m'}}{(2)_{m'}m'!}\frac{\Gamma\left(\frac{2(m-m')+2+k}{k}\right)}{\Gamma\left(\frac{2(m-m')+2(n-n')+k+3}{k}\right)}\frac{\Gamma\left(\frac{2m'+2+k}{k}\right)}{\Gamma\left(\frac{2m'+2n'+k+3}{k}\right)}$$

$$f_n = \sum_{n'=0}^{n} \frac{\Gamma\left(\frac{2(n-n')+1}{2}\right)(z+1)_{2(n-n')}}{\left(\frac{1}{2}\right)_{n-n'}(n-n')!} \frac{\Gamma\left(\frac{2n'+1}{2}\right)(z+1)_{2n'}}{\left(\frac{1}{2}\right)_{n'} n'!} \sum_{m'=0}^{m} \frac{(z+1)_{2(m-m')}}{(2)_{m-m'}(m-m')!} \frac{(z+1)_{2m'}}{(2)_{m'} m'!} \frac{\Gamma\left(\frac{2(m-m')+2+2}{2}\right)}{\Gamma\left(\frac{2(m-m')+2(n-n')+2+3}{2}\right)} \frac{\Gamma\left(\frac{2m'+2+2}{2}\right)}{\Gamma\left(\frac{2m'+2n'+2+3}{2}\right)}$$

$$= \sum_{n'=0}^{n} \frac{\Gamma\left(n-n'+\frac{1}{2}\right)(z+1)_{2(n-n')}}{\left(\frac{1}{2}\right)_{n-n'}(n-n')!} \frac{\Gamma\left(n'+\frac{1}{2}\right)(z+1)_{2n'}}{\left(\frac{1}{2}\right)_{n'} n'!} \sum_{m'=0}^{m} \frac{(z+1)_{2(m-m')}}{(2)_{m-m'}(m-m')!} \frac{(z+1)_{2m'}}{(2)_{m'} m'!} \frac{\Gamma(m-m'+2)}{\Gamma\left(m-m'+n-n'+\frac{5}{2}\right)} \frac{\Gamma(m'+2)}{\Gamma\left(m'+n'+\frac{5}{2}\right)}$$

$$= \sum_{n'=0}^{n} \frac{\Gamma\left(n-n'+\frac{1}{2}\right)\Gamma\left(\frac{1}{2}\right)(z+1)_{2(n-n')}}{\Gamma\left(n-n'+\frac{1}{2}\right)(n-n')!} \frac{\Gamma\left(n'+\frac{1}{2}\right)\Gamma\left(\frac{1}{2}\right)(z+1)_{2n'}}{\Gamma\left(n'+\frac{1}{2}\right)n'!} \sum_{m'=0}^{m} \frac{(z+1)_{2(m-m')}}{\Gamma(m-m'+2)(m-m')!} \frac{(z+1)_{2m'}}{\Gamma(m'+2)m'!} \frac{\Gamma(2)\,\Gamma(m-m'+2)}{\Gamma\left(m-m'+n-n'+\frac{5}{2}\right)} \frac{\Gamma(2)\Gamma(m'+2)}{\Gamma\left(m'+n'+\frac{5}{2}\right)}$$

$$= \pi \sum_{n'=0}^{n} \frac{(z+1)_{2(n-n')}}{(n-n')!} \frac{(z+1)_{2n'}}{n'!} \sum_{m'=0}^{m} \frac{(z+1)_{2(m-m')}}{(m-m')!} \frac{(z+1)_{2m'}}{m'!} \frac{1}{\Gamma\left(m-m'+n-n'+\frac{5}{2}\right)} \frac{1}{\Gamma\left(m'+n'+\frac{5}{2}\right)}$$

Biaxial ellipsoid, polydisperse, perfect orientation, F(q)

6.4 Triaxial Ellipsoid

The perfectly oriented triaxial ellipsoid corresponds to the case of the superball with $k = 2$.

6.4.1 Formfactor P(q)

Regime I. From Eq. (S.4.8.7) we have the series expansion

$$P(q) = \frac{\Gamma^2\left(\frac{3}{p}+1\right)}{\Gamma^6\left(\frac{1}{p}\right)} \sum_{n=0}^{\infty}\left(-\frac{q_x^2a^2}{4}\right)^n \sum_{m=0}^{\infty}\left(-\frac{q_y^2b^2}{4}\right)^m \sum_{k=0}^{\infty}\left(-\frac{q_z^2c^2}{4}\right)^k \sum_{n'=0}^{n} \frac{\Gamma\left(\frac{2(n-n')+1}{p}\right)\Gamma\left(\frac{2n'+1}{p}\right)}{\left(\frac{1}{2}\right)_{n-n'}\left(\frac{1}{2}\right)_{n'}(n-n')!\,(n')!} \sum_{m'}^{m} \frac{\Gamma\left(\frac{2(m-m')+1}{p}\right)\Gamma\left(\frac{2m'+1}{p}\right)}{\left(\frac{1}{2}\right)_{m-m'}\left(\frac{1}{2}\right)_{m'}(m-m')!\,(m')!} \sum_{k'}^{k} \frac{\Gamma\left(\frac{2(k-k')+1}{p}\right)\Gamma\left(\frac{2k'+1}{p}\right)}{\left(\frac{1}{2}\right)_{k-k'}\left(\frac{1}{2}\right)_{k'}(k-k')!\,(k')!} \frac{1}{\Gamma\left(\frac{2(n-n')+2(m-m')+2(k-k')+3}{p}+1\right)} \frac{1}{\Gamma\left(\frac{2n'+2m'+2k'+3}{p}+1\right)}$$

$$P(q) = \frac{\Gamma^2\left(\frac{3}{p}+1\right)}{\Gamma^6\left(\frac{1}{p}\right)} \sum_{n=0}^{\infty}\left(-\frac{q_x^2a^2}{4}\right)^n \sum_{m=0}^{\infty}\left(-\frac{q_y^2b^2}{4}\right)^m \sum_{k=0}^{\infty}\left(-\frac{q_z^2c^2}{4}\right)^k c_{n,m,k}$$

with

$$c_{n,m,k} = \sum_{n'=0}^{n} \frac{\Gamma\left(\frac{2(n-n')+1}{p}\right)\Gamma\left(\frac{2n'+1}{p}\right)}{\left(\frac{1}{2}\right)_{n-n'}\left(\frac{1}{2}\right)_{n'}(n-n')!\,(n')!} \sum_{m'}^{m} \frac{\Gamma\left(\frac{2(m-m')+1}{p}\right)\Gamma\left(\frac{2m'+1}{p}\right)}{\left(\frac{1}{2}\right)_{m-m'}\left(\frac{1}{2}\right)_{m'}(m-m')!\,(m')!} \sum_{k'}^{k} \frac{\Gamma\left(\frac{2(k-k')+1}{p}\right)\Gamma\left(\frac{2k'+1}{p}\right)}{\left(\frac{1}{2}\right)_{k-k'}\left(\frac{1}{2}\right)_{k'}(k-k')!\,(k')!} \frac{1}{\Gamma\left(\frac{2(n-n')+2(m-m')+2(k-k')+3}{p}+1\right)} \frac{1}{\Gamma\left(\frac{2n'+2m'+2k'+3}{p}+1\right)}$$

For k=2 we have

$$P(q) = \frac{\left(\frac{9\pi}{16}\right)}{\Gamma^6\left(\frac{1}{2}\right)} \sum_{n=0}^{\infty}\left(-\frac{q_x^2a^2}{4}\right)^n \sum_{m=0}^{\infty}\left(-\frac{q_y^2b^2}{4}\right)^m \sum_{k=0}^{\infty}\left(-\frac{q_z^2c^2}{4}\right)^k c_{n,m,k}$$

$$c_{n,m,k} = \sum_{n'=0}^{n} \frac{\Gamma\left(\frac{2(n-n')+1}{2}\right)\Gamma\left(\frac{2n'+1}{2}\right)}{\left(\frac{1}{2}\right)_{n-n'}\left(\frac{1}{2}\right)_{n'}(n-n')!\,(n')!} \sum_{m'}^{m} \frac{\Gamma\left(\frac{2(m-m')+1}{2}\right)\Gamma\left(\frac{2m'+1}{2}\right)}{\left(\frac{1}{2}\right)_{m-m'}\left(\frac{1}{2}\right)_{m'}(m-m')!\,(m')!} \sum_{k'}^{k} \frac{\Gamma\left(\frac{2(k-k')+1}{2}\right)\Gamma\left(\frac{2k'+1}{2}\right)}{\left(\frac{1}{2}\right)_{k-k'}\left(\frac{1}{2}\right)_{k'}(k-k')!\,(k')!} \frac{1}{\Gamma\left(\frac{2(n-n')+2(m-m')+2(k-k')+3}{2}+1\right)} \frac{1}{\Gamma\left(\frac{2n'+2m'+2k'+3}{2}+1\right)}$$

$$= \sum_{n'=0}^{n} \frac{\Gamma\left(\frac{1}{2}\right)\Gamma\left(n-n'+\frac{1}{2}\right)\Gamma\left(\frac{1}{2}\right)\Gamma\left(n'+\frac{1}{2}\right)}{\Gamma\left(n-n'+\frac{1}{2}\right)\Gamma\left(n'+\frac{1}{2}\right)(n-n')!\,(n')!} \sum_{m'}^{m} \frac{\Gamma\left(\frac{1}{2}\right)\Gamma\left(m-m'+\frac{1}{2}\right)\Gamma\left(\frac{1}{2}\right)\Gamma\left(m'+\frac{1}{2}\right)}{\Gamma\left(m-m'+\frac{1}{2}\right)\Gamma\left(m'+\frac{1}{2}\right)(m-m')!\,(m')!} \sum_{k'}^{k} \frac{\Gamma\left(\frac{1}{2}\right)\Gamma\left(k-k'+\frac{1}{2}\right)\Gamma\left(\frac{1}{2}\right)\Gamma\left(k'+\frac{1}{2}\right)}{\Gamma\left(k-k'+\frac{1}{2}\right)\Gamma\left(k'+\frac{1}{2}\right)(k-k')!\,(k')!} \frac{1}{\Gamma\left(n-n'+m-m'+k-k'+\frac{5}{2}\right)} \frac{1}{\Gamma\left(n'+m'+k'+\frac{5}{2}\right)}$$

$$= \sum_{n'=0}^{n} \frac{\Gamma\left(\frac{1}{2}\right)\Gamma\left(\frac{1}{2}\right)}{(n-n')!\,(n')!} \sum_{m'}^{m} \frac{\Gamma\left(\frac{1}{2}\right)\Gamma\left(\frac{1}{2}\right)}{(m-m')!\,(m')!} \sum_{k'}^{k} \frac{\Gamma\left(\frac{1}{2}\right)\Gamma\left(\frac{1}{2}\right)}{(k-k')!\,(k')!} \frac{1}{\Gamma\left(n-n'+m-m'+k-k'+\frac{5}{2}\right)} \frac{1}{\Gamma\left(n'+m'+k'+\frac{5}{2}\right)}$$

$$= \Gamma^6\left(\frac{1}{2}\right) \sum_{n'=0}^{n} \frac{1}{(n-n')!\,(n')!} \sum_{m'}^{m} \frac{1}{(m-m')!\,(m')!} \sum_{k'}^{k} \frac{1}{(k-k')!\,(k')!} \frac{1}{\Gamma\left(n-n'+m-m'+k-k'+\frac{5}{2}\right)} \frac{1}{\Gamma\left(n'+m'+k'+\frac{5}{2}\right)}$$

Thus we have **(S.6.4.1.1)**

$$P(q)=\left(\frac{9\pi}{16}\right)\sum_{n=0}^{\infty}\left(-\frac{q_x^2a^2}{4}\right)^n\sum_{m=0}^{\infty}\left(-\frac{q_y^2b^2}{4}\right)^m\sum_{k=0}^{\infty}\left(-\frac{q_z^2c^2}{4}\right)^k c_{n,m,k}$$

$$c_{n,m,k}=\sum_{n'=0}^{n}\frac{1}{(n-n')!\,(n')!}\sum_{m'}^{m}\frac{1}{(m-m')!\,(m')!}\sum_{k'}^{k}\frac{1}{(k-k')!\,(k')!}\frac{1}{\Gamma\left(n-n'+m-m'+k-k'+\frac{5}{2}\right)}\frac{1}{\Gamma\left(n'+m'+k'+\frac{5}{2}\right)}$$

Triaxial ellipsoid, polydisperse, perfect orientation, P(q)

Regime III. There is a direct overlap to the Porod regime with **(S.6.4.1.2)**

$$\lim_{q\to\infty}P(q)=\frac{\Gamma(z-3)(z+1)^4}{\Gamma(z+1)}\frac{9}{2\left((q_xa)^2+\left(q_yb\right)^2+(q_zc)^2\right)^{4/2}}$$

Mathematica code implementation:

```
max=30;
na=max;
ma=max;
la=max;
fff=Table[Sum[(1/((ns!)*((n-ns)!)))*
    Sum[(1/((ms!)*((m-ms)!)))*Sum[(1/((ls!)*((l-ls)!)))*(1/(Gamma[ns+ms+ls+5/2]*Gamma[n-ns+m-ms+l-ls+5/2])),{ls, 0,
l}],{ms, 0, m}],{ns, 0, n}], {n, 0, na}, {m, 0, ma},{l, 0, la}];
afff=9*Pi/16;

a=1.0;
b=2;
c=3;
sigma=0.11;
z=(1-sigma*sigma)/(sigma*sigma);

alf=0.3*Pi/2;
beta=0.2*Pi/2;
gam=0.4*Pi/2;
RRx={{1,0,0},{0,Cos[alf],-Sin[alf]},{0,Sin[alf],Cos[alf]}};
RRy={{Cos[beta],0,Sin[beta]},{0,1,0},{-Sin[beta],0,Cos[beta]}};
RRz={{Cos[gam],-Sin[gam],0},{Sin[gam],Cos[gam],0},{0,0,1}};
RR=RRz.RRy.RRz;
qz=0.001;
qv={qx,qy,qz};
qvs=RR.qv;
qxx=qvs[[1]];
qyy=qvs[[2]];
qzz=qvs[[3]];
qs=10^lqs;
phi=0.3*Pi/2;
theta=0.1*Pi/2;
qxs=qs*Cos[phi]*Sin[theta];
qys=qs*Sin[phi]*Sin[theta];
qzs=qs*Cos[theta];
Pqav=(afff)*Sum[(((-1/(4*(z+1)*(z+1)))^n))*Sum[(Pochhammer[z+1,2*(n-m)]*((a*a*qxx*qxx)^(n-
m)))*Sum[(Pochhammer[z+1,2*(m-l)]*((b*b*qyy*qyy)^(m-l)))*(Pochhammer[z+1,2*l]*((c*c*qzz*qzz)^l))*fff[[n-m+1,m-
l+1,l+1]], {l, 0, m}],{m, 0, n}], {n , 0, max}];
```

```
Pqavs=(afff)*Sum[(((-1/(4*(z+1)*(z+1)))^n))*Sum[(Pochhammer[z+1,2*(n-m)]*((a*a*qxs*qxs)^(n-m)))*Sum[(Pochhammer[z+1,2*(m-l)]*((b*b*qys*qys)^(m-l)))*(Pochhammer[z+1,2*l]*((c*c*qzs*qzs)^l))*fff[[n-m+1,m-l+1,l+1]], {l, 0, m}],{m, 0, n}], {n , 0, max}];
apor=Gamma[z-3]*((z+1)^4)/Gamma[z+1];
Rkk=Sqrt[((a*Cos[phi]*Sin[theta])^2)+((b*Sin[phi]*Sin[theta])^2)+((c*Cos[theta])^2)];
Pqpor=(9*apor /2)/((qs^4)*(Rkk^4));
lim=1.5;
pl1=Plot[Log[10,Pqavs], {lqs, -1, lim}, PlotRange->{-6,1}, GridLines->{Automatic}, LabelStyle->Directive[Black,16],AxesLabel->{"log q","lg P(q)"}, AxesOrigin->{-1,-6},TicksStyle->Directive[Black,10],PlotStyle->{Blue,Dashed}];
pl2=Plot[Log[10,Pqpor], {lqs, -1, lim}, PlotRange->{-6,1}, GridLines->{Automatic}, LabelStyle->Directive[Black,16],AxesLabel->{"log q","lg P(q)"}, AxesOrigin->{-1,-6},TicksStyle->Directive[Black,10],PlotStyle->{Red}];
Show[pl2,pl1]
lims=5;
pl5=DensityPlot[Log[10,Pqav],{qx, -lims, lims}, {qy, -lims, lims}, PlotRange->{-8,0}, PlotPoints->50, PlotLegends->Automatic, LabelStyle->Directive[Black,12],AxesLabel->Automatic]
```

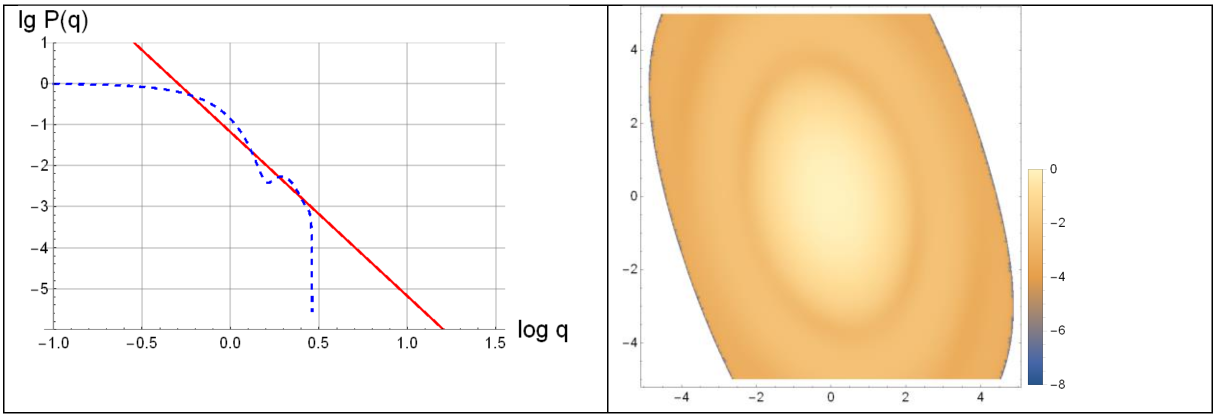

Fig. S50: Formfactor of perfectly oriented triaxial polydisperse ellipsoids.

6.4.2 Scattering amplitude F(q)

We can derive an expression for the scattering amplitude, but it only serves to describe the low-q regime

Regime I. From Eq. (S.4.8.8) we have the series expansion

$$\langle F(q)\rangle^2 = \frac{\Gamma^2\left(\frac{3}{p}+1\right)}{\Gamma^6\left(\frac{1}{p}\right)}\sum_{n=0}^{\infty}\left(-\frac{q_x^2a^2}{4(z+1)^2}\right)^n\sum_{n'=0}^{n}\frac{(z+s+1)_{2(n-n')}\,(z+s+1)_{2n'}\Gamma\left(\frac{2(n-n')+1}{p}\right)\Gamma\left(\frac{2n'+1}{p}\right)}{\left(\frac{1}{2}\right)_{n-n'}\left(\frac{1}{2}\right)_{n'}(n-n')!\,(n')!}\sum_{m=0}^{\infty}\left(-\frac{q_y^2b^2}{4(z+1)^2}\right)^m\sum_{m'}^{m}\frac{(z+s+1)_{2(m-m')}\,(z+s+1)_{2m'}\Gamma\left(\frac{2(m-m')+1}{p}\right)\Gamma\left(\frac{2m'+1}{p}\right)}{\left(\frac{1}{2}\right)_{m-m'}\left(\frac{1}{2}\right)_{m'}(m-m')!\,(m')!}\sum_{k=0}^{\infty}\left(-\frac{q_z^2c^2}{4(z+1)^2}\right)^k\sum_{k'}^{k}\frac{\Gamma\left(\frac{2(k-k')+1}{p}\right)\Gamma\left(\frac{2k'+1}{p}\right)}{\left(\frac{1}{2}\right)_{k-k'}\left(\frac{1}{2}\right)_{k'}(k-k')!\,(k')!}$$

$$\frac{(z+s+1)_{2(k-k')}\,(z+s+1)_{2k'}}{\Gamma\left(\frac{2(n-n')+2(m-m')+2(k-k')+3}{p}+1\right)}\frac{1}{\Gamma\left(\frac{2n'+2m'+2k'+3}{p}+1\right)}$$

$$\langle F(q)\rangle^2 = \frac{\Gamma^2\left(\frac{3}{p}+1\right)}{\Gamma^6\left(\frac{1}{p}\right)}\sum_{n=0}^{\infty}\left(-\frac{q_x^2a^2}{4(z+1)^2}\right)^n f_n\sum_{m=0}^{\infty}\left(-\frac{q_y^2b^2}{4(z+1)^2}\right)^m f_m\sum_{k=0}^{\infty}\left(-\frac{q_z^2c^2}{4(z+1)^2}\right)^k f_{n,m,k}$$

$$f_n = \sum_{n'=0}^{n} \frac{(z+s+1)_{2(n-n')}\,(z+s+1)_{2n'}\Gamma\left(\frac{2(n-n')+1}{p}\right)\Gamma\left(\frac{2n'+1}{p}\right)}{\left(\frac{1}{2}\right)_{n-n'}\left(\frac{1}{2}\right)_{n'}(n-n')!\,(n')!}$$

$$f_m = \sum_{m'}^{m} \frac{(z+s+1)_{2(m-m')}\,(z+s+1)_{2m'}\Gamma\left(\frac{2(m-m')+1}{p}\right)\Gamma\left(\frac{2m'+1}{p}\right)}{\left(\frac{1}{2}\right)_{m-m'}\left(\frac{1}{2}\right)_{m'}(m-m')!\,(m')!}$$

$$f_{n,m,k} = \sum_{k'}^{k} \frac{\Gamma\left(\frac{2(k-k')+1}{p}\right)\Gamma\left(\frac{2k'+1}{p}\right)}{\left(\frac{1}{2}\right)_{k-k'}\left(\frac{1}{2}\right)_{k'}(k-k')!\,(k')!}\frac{(z+s+1)_{2(k-k')}\,(z+s+1)_{2k'}}{\Gamma\left(\frac{2(n-n')+2(m-m')+2(k-k')+3}{p}+1\right)}\frac{1}{\Gamma\left(\frac{2n'+2m'+2k'+3}{p}+1\right)}$$

Thus, in the case k=2

$$\langle F(q)\rangle^2 = \frac{\left(\frac{9\pi}{16}\right)}{\Gamma^6\left(\frac{1}{2}\right)}\sum_{n=0}^{\infty}\left(-\frac{q_x^2a^2}{4(z+1)^2}\right)^n f_n \sum_{m=0}^{\infty}\left(-\frac{q_y^2b^2}{4(z+1)^2}\right)^m f_m \sum_{k=0}^{\infty}\left(-\frac{q_z^2c^2}{4(z+1)^2}\right)^k f_{n,m,k}$$

$$f_n = \sum_{n'=0}^{n} \frac{(z+s+1)_{2(n-n')}\,(z+s+1)_{2n'}\Gamma\left(\frac{2(n-n')+1}{2}\right)\Gamma\left(\frac{2n'+1}{2}\right)}{\left(\frac{1}{2}\right)_{n-n'}\left(\frac{1}{2}\right)_{n'}(n-n')!\,(n')!}$$

$$= \sum_{n'=0}^{n} \frac{(z+s+1)_{2(n-n')}\,(z+s+1)_{2n'}\Gamma\left(\frac{1}{2}\right)\Gamma\left(n-n'+\frac{1}{2}\right)\Gamma\left(\frac{1}{2}\right)\Gamma\left(n'+\frac{1}{2}\right)}{\Gamma\left(n-n'+\frac{1}{2}\right)\Gamma\left(n'+\frac{1}{2}\right)(n-n')!\,(n')!}$$

$$= \left(\Gamma\left(\frac{1}{2}\right)\right)^2 \sum_{n'=0}^{n} \frac{(z+s+1)_{2(n-n')}\,(z+s+1)_{2n'}}{(n-n')!\,(n')!}$$

$$f_m = \sum_{m'}^{m} \frac{(z+s+1)_{2(m-m')}\,(z+s+1)_{2m'}\Gamma\left(\frac{2(m-m')+1}{2}\right)\Gamma\left(\frac{2m'+1}{2}\right)}{\left(\frac{1}{2}\right)_{m-m'}\left(\frac{1}{2}\right)_{m'}(m-m')!\,(m')!}$$

$$= \sum_{m'}^{m} \frac{(z+s+1)_{2(m-m')}\,(z+s+1)_{2m'}\Gamma\left(\frac{1}{2}\right)\Gamma\left(m-m'+\frac{1}{2}\right)\Gamma\left(\frac{1}{2}\right)\Gamma\left(m'+\frac{1}{2}\right)}{\Gamma\left(m-m'+\frac{1}{2}\right)\Gamma\left(m'+\frac{1}{2}\right)(m-m')!\,(m')!}$$

$$= \left(\Gamma\left(\frac{1}{2}\right)\right)^2 \sum_{m'}^{m} \frac{(z+s+1)_{2(m-m')}\,(z+s+1)_{2m'}}{(m-m')!\,(m')!}$$

$$f_{n,m,k} = \sum_{k'}^{k} \frac{\Gamma\left(\frac{2(k-k')+1}{2}\right)\Gamma\left(\frac{2k'+1}{2}\right)}{\left(\frac{1}{2}\right)_{k-k'}\left(\frac{1}{2}\right)_{k'}(k-k')!\,(k')!}\frac{(z+s+1)_{2(k-k')}\,(z+s+1)_{2k'}}{\Gamma\left(\frac{2(n-n')+2(m-m')+2(k-k')+3}{2}+1\right)}\frac{1}{\Gamma\left(\frac{2n'+2m'+2k'+3}{2}+1\right)}$$

$$= \sum_{k'}^{k} \frac{\Gamma\left(\frac{1}{2}\right)\Gamma\left(k-k'+\frac{1}{2}\right)\Gamma\left(\frac{1}{2}\right)\Gamma\left(k'+\frac{1}{2}\right)}{\Gamma\left(k-k'+\frac{1}{2}\right)\Gamma\left(k'+\frac{1}{2}\right)(k-k')!\,(k')!}\frac{(z+s+1)_{2(k-k')}\,(z+s+1)_{2k'}}{\Gamma\left(n-n'+m-m'+k-k'+\frac{5}{2}\right)}\frac{1}{\Gamma\left(n'+m'+k'+\frac{5}{2}\right)}$$

$$= \left(\Gamma\left(\frac{1}{2}\right)\right)^2 \sum_{k'}^{k} \frac{1}{(k-k')!\,(k')!}\frac{(z+s+1)_{2(k-k')}\,(z+s+1)_{2k'}}{\Gamma\left(n-n'+m-m'+k-k'+\frac{5}{2}\right)}\frac{1}{\Gamma\left(n'+m'+k'+\frac{5}{2}\right)}$$

$$f_n f_m f_{n,m,k} = \left(\Gamma\left(\frac{1}{2}\right)\right)^6 \sum_{n'=0}^{n} \frac{(z+s+1)_{2(n-n')}\,(z+s+1)_{2n'}}{(n-n')!\,(n')!} \sum_{m'}^{m} \frac{(z+s+1)_{2(m-m')}\,(z+s+1)_{2m'}}{(m-m')!\,(m')!} \sum_{k'}^{k} \frac{1}{(k-k')!\,(k')!} \frac{(z+s+1)_{2(k-k')}\,(z+s+1)_{2k'}}{\Gamma\left(n-n'+m-m'+k-k'+\frac{5}{2}\right)} \frac{1}{\Gamma\left(n'+m'+k'\right.} [illegible]$$

Thus, we have **<u>(S.6.4.2.1)</u>**

$$\langle F(q)\rangle^2 = \left(\frac{9\pi}{16}\right) \sum_{n=0}^{\infty} \left(-\frac{q_x^2 a^2}{4(z+1)^2}\right)^n \sum_{m=0}^{\infty} \left(-\frac{q_y^2 b^2}{4(z+1)^2}\right)^m \sum_{k=0}^{\infty} \left(-\frac{q_z^2 c^2}{4(z+1)^2}\right)^k f_n f_m f_{n,m,k}$$

$$f_n f_m f_{n,m,k} = \sum_{n'=0}^{n} \frac{(z+s+1)_{2(n-n')}\,(z+s+1)_{2n'}}{(n-n')!\,(n')!} \sum_{m'}^{m} \frac{(z+s+1)_{2(m-m')}\,(z+s+1)_{2m'}}{(m-m')!\,(m')!} \sum_{k'}^{k} \frac{1}{(k-k')!\,(k')!} \frac{(z+s+1)_{2(k-k')}\,(z+s+1)_{2k'}}{\Gamma\left(n-n'+m-m'+k-k'+\frac{5}{2}\right)} \frac{1}{\Gamma\left(n'+m'+k'+\frac{5}{2}\right)}$$

Triaxial ellipsoid, polydisperse, perfect orientation, F(q)

7. Uniaxial orientational distributions

7.1 Coordinate System

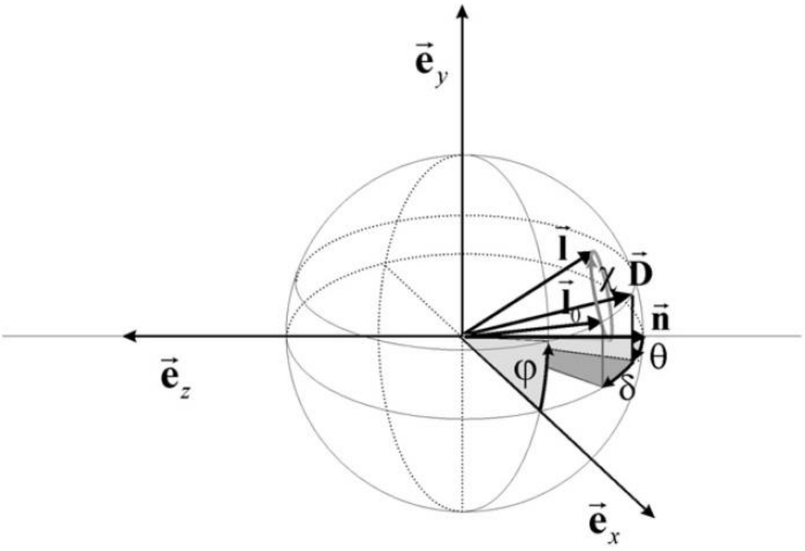

Fig. S51: Coordinate system for the calculation of uniaxial orientational distributions.

To specify the orientation of anisometric particles, we use the coordinate system shown in Fig. S34. The direction of the probe beam is ***n***=(0,0,-1). The polar angle θ is measured from the y-axis, which corresponds to the vertical axis of the detector. The azimuthal angle φ is measured in the (x,z)-plane from the x-axis, which corresponds to the z-axis of the detector. The two angles specify the spatial orientation of the director **D**. The characteristic axis of a given particle may have a deviation angle δ with respect to the director. We will assume a certain orientational

distribution function h(δ) to describe the distribution of deviation angles of the particles. In the examples we assume a Gaussian distribution

7.2. Orientational Distribution Functions

There are different types of orientational distribution functions such as the Gaussian function

$$h(\delta) = \exp\left[-\left(\frac{\delta}{\bar{\delta}}\right)^2\right]$$

Any other angular distribution function such as an exponential or Onsager function could be used as well. Similar to the size distribution function the distribution function is normalized such

$$\langle F \rangle = \frac{\int_0^{\pi/2} F(\delta) h(\delta) \sin\delta}{\int_0^{\pi/2} h(\delta) \sin\delta}$$

In particular, the corresponding orientational order parameter S is given by

$$S = \frac{\int_0^{\pi/2} \left(\frac{3\cos^2\delta - 1}{2}\right) h(\delta) \sin\delta}{\int_0^{\pi/2} h(\delta) \sin\delta}$$

and can thus be directly computed in the algorithm.

7.3 Calculation Scheme

To calculate the average of the formfactor $\langle P(q) \rangle$ over the orientational distribution requires to integrate over the angular range $0 \leq \delta < \pi/2$ and over the cone angle $0 \leq \chi < 2\pi$. In the following we will show that in the small-angle approximation a particularly simple expression for the phase $\boldsymbol{qL}$ can be derived, which can be directly implemented in the series and asymptotic expansions as well as in the Porod-asymptote derived above.

The director is specified by the unit vector $\boldsymbol{D}$ in terms of the angles φ, θ.

$$\boldsymbol{D} = \begin{pmatrix} \cos(\varphi)\sin(\theta) \\ \sin(\varphi)\sin(\theta) \\ -\cos(\theta) \end{pmatrix}$$

We consider a characteristic axis vector $\boldsymbol{L}_\delta$ of a anisometric particle, which may be the cylinder, ellipsoid or parallelepiped axis c. It has a deviation angle δ with respect to the director, such that the corresponding vector $\boldsymbol{L}_\delta$ is given by

$$\boldsymbol{L}_\delta = L\begin{pmatrix}\cos(\varphi)\sin(\theta-\delta)\\ \sin(\varphi)\sin(\theta-\delta)\\ -\cos(\theta-\delta)\end{pmatrix}$$

We further introduce a rotation matrix

$$\boldsymbol{M} = \begin{pmatrix} c\chi+(1-c\chi)s^2\theta c^2\varphi & -c\theta s\chi+(1-c\chi)s^2\theta c\varphi s\varphi & -s\varphi s\theta s\chi-(1-c\chi)c\varphi c\theta s\theta \\ c\theta s\chi+(1-c\chi)s^2\theta c\varphi s\varphi & c\chi+(1-c\chi)s^2\theta s^2\varphi & s\theta c\varphi s\chi-(1-c\chi)c\theta s\theta s\varphi \\ s\theta s\varphi s\chi-(1-c\chi)c\varphi c\theta s\theta & -s\theta c\varphi s\chi+(1-c\chi)c\theta s\varphi s\theta & c\chi+(1-c\chi)c^2\theta \end{pmatrix}$$

where $\cos(\varphi)=c\varphi$, $\sin(\varphi)=s\varphi$, $\cos(\theta)=c\theta$, $\sin(\theta)=s\theta$, $\cos(\chi)=c\chi$, $\sin(\chi)=s\chi$. The matrix serves to calculate the vectors $\boldsymbol{L}(\delta,\chi)$ that have a deviation angle δ and are located on the cone in Fig. xx with a cone angle χ a.

$$\boldsymbol{L}(\delta,\chi) = \boldsymbol{M}\boldsymbol{L}_\delta$$

As an example, we consider the frequent cases where the particles are aligned parallel to the $x-$, $y-$, $z-$ axis, together with the general case that the particles are aligned with respect to any spatial direction.

7.4. Alignment parallel to x-axis

The case that $\boldsymbol{D} \parallel x$ corresponds to the angles φ = 0, $\theta = \pi/2$. The director $\boldsymbol{D}$, axis-vector $\boldsymbol{L}_\delta$, the matrix $\boldsymbol{M}$, the vector $\boldsymbol{L}(\delta,\chi)$, and the phase $\boldsymbol{qL}(\delta,\chi)$ are then given by

$$\boldsymbol{D} = \begin{pmatrix}1\\0\\0\end{pmatrix} \quad \boldsymbol{L}_\delta = L\begin{pmatrix}\cos(\delta)\\0\\-\sin(\delta)\end{pmatrix} \quad \boldsymbol{M} = \begin{pmatrix}1 & 0 & 0\\ 0 & c\chi & s\chi\\ 0 & -s\chi & c\chi\end{pmatrix} \quad \boldsymbol{L}(\delta,\chi) = L\begin{pmatrix}\cos(\delta)\\ -\sin(\chi)\sin(\delta)\\ -\cos(\chi)\sin(\delta)\end{pmatrix}$$

$$\boldsymbol{qL}(\delta,\chi) = \boldsymbol{qML}_\delta = L\big(q_x\cos(\delta) - q_y\sin(\chi)\sin(\delta) - q_z\cos(\chi)\sin(\delta)\big)$$

In the small-angle approximation we have $q_z \approx 0$ such that

$$\boldsymbol{qL}(\delta,\chi) \approx L\big(q_x\cos(\delta) - q_y\sin(\chi)\sin(\delta)\big)$$

For perfect orientation with $\delta = 0$ the expression reduces to $\boldsymbol{qL}(\delta,\chi) = q_xL$.

7.4.1 Cylinders

For the series and asymptotic expansion, and for the Porod asymptote we need the averaged phase term $\boldsymbol{qL}$. For cylinders L represents the long axis such that the phase term is given by (S.7.4.1.1)

$$\boldsymbol{qL} = qL\cos(\beta)$$

where β is the angle enclosed by the scattering vector $\boldsymbol{q}$ and the axis vector $\boldsymbol{L}$. This is similar for ellipsoids or parallelepipeds considering the alignment of the **c**-axis.

The enclosed angle β can be expressed in terms of the deviation and cone angles δ, χ as (S.7.4.1.2)

$$\boldsymbol{qL} = qL\cos(\beta) = qL\cos(\arccos[\boldsymbol{qML}_{\delta}]) = qL\left[\hat{q}_x\cos(\delta) - \hat{q}_y\sin(\chi)\sin(\delta)\right]$$

with $\hat{q}_x = \frac{q_x}{q}$ and $\hat{q}_y = \frac{q_y}{q}$. For perfect orientation with $\delta = 0$ the expression reduces to

$$\boldsymbol{qL} = q_x L$$

For the formfactor we need to the expression $(\boldsymbol{qL})^{2n}$

$$(\boldsymbol{qL})^{2n} = (qL)^{2n}\left(\hat{q}_x\cos(\delta) - \hat{q}_y\sin(\chi)\sin(\delta)\right)^{2n}$$

We use the binomial form

$$(\boldsymbol{qL})^{2n} = (qL)^{2n}\sum_{m=0}^{2n}\binom{2n}{m}\left(\hat{q}_x\right)^m\left(-\hat{q}_y\right)^{2n-m}\left[(\cos(\delta))^m(\sin(\chi)\sin(\delta))^{2n-m}\right]$$

The angular part […] has to be integrated over the cone angle χ and the deviation angle δ

$$\langle(\boldsymbol{qL})^{2n}\rangle = (qL)^{2n}\sum_{m=0}^{2n}\binom{2n}{m}(\hat{q}_x)^m(-\hat{q}_y)^{2n-m}\left(\frac{1}{2\pi}\int_{\mathbf{0}}^{2\pi}(\sin(\chi))^{2n-m}\boldsymbol{d}\chi\right)\left(\int_{\mathbf{0}}^{\frac{\pi}{2}}(\cos(\delta))^m(\sin(\delta))^{2n-m}\sin(\delta)\,d\delta\right)$$

The integral of the cone angle χ can be solved analytically

$$\frac{1}{2\pi}\int_0^{2\pi}(\sin(\chi))^{2n-m}d\chi = \frac{(1+(-1)^{2n-m})\sqrt{\pi}\,\Gamma\left[\frac{2n-m+1}{2}\right]}{2\pi\Gamma\left[\frac{2n-m+2}{2}\right]}$$

The integral of the deviation angle δ is given by (S.7.4.1.3)

$$\frac{\int_0^{\pi/2}(\cos(\delta))^m(\sin(\delta))^{2n-m}h(\delta)\sin(\delta)\,d\delta}{\int_0^{\pi/2}h(\delta)\sin(\delta)\,d\delta} = H_{m,2n-m+1}$$

where for $H_{i,j}$ the first index *i* is equal to the overall $\cos(\delta)$-exponent, and the index *j* equal to the overall $\sin(\delta)$-exponent. The integral is computed numerically. It is q-independent, such that it needs to be computed only once for a scattering pattern. We now have

$$\langle(\boldsymbol{qL})^{2n}\rangle = (qL)^{2n}\sum_{m=0}^{2n}\frac{(2n)!}{m!\,(n-m)!}(\hat{q}_x)^m\left(-\hat{q}_y\right)^{2n-m}\frac{(1+(-1)^{2n-m})\sqrt{\pi}\Gamma\left[\frac{2n-m+1}{2}\right]}{2\pi\Gamma\left[\frac{2n-m+2}{2}\right]}H_{m,2n-m}$$

We observe that there are contributions to the sum only if *m* is even, i.e. $m = 2l$. We therefore change the summation index from *m* to *l* and simplify to obtain

$$\langle(\boldsymbol{qL})^{2n}\rangle = (qL)^{2n}(2n)!\sum_{l=0}^{n}\frac{1}{(2l)!\,\Gamma[n-2l+1]}\left(\hat{q}_x^2\right)^l\left(\hat{q}_y^2\right)^{n-l}\frac{\sqrt{\pi}\Gamma\left[\frac{2n-2l+1}{2}\right]}{\pi(\mathrm{n}-\mathrm{l})!}H_{2l,2n-2l}$$

With a further simplification using **(S.7.4.1.4)**

$$\frac{\Gamma\left[\frac{x}{2}\right]}{\Gamma[x]} = \frac{\sqrt{\pi}}{2^{x-1}\Gamma\left[\frac{x+1}{2}\right]}$$

with $x = 2n - 2l + 1$ to remove all Gamma functions we finally obtain an expression that can be inserted into the expansions and asymptotes **(S.7.4.1.5)**

$$\langle(\boldsymbol{qL})^{2n}\rangle = (qL)^{2n}\frac{(2n)!}{4^n}\sum_{l=0}^{n}\frac{4^l}{(2l)!\left((\mathrm{n}-\mathrm{l})!\right)^2}(\hat{q}_x^2)^l\left(\hat{q}_y^2\right)^{n-l}H_{2l,2n-2l}$$

Regime I. This can then be inserted into the cylinder formfactor (Eq. S.4.6.1.1.2), and simplified to **(S.7.4.1.6)**

$$\langle P_{\|}(q,L)\rangle = \sum_{n=0}^{\infty}\frac{4^n}{(\mathrm{n}+1)}\frac{(z+1)_{2n}}{\left(\frac{3}{2}\right)_n n!}\left(-\frac{(\boldsymbol{qL})^2}{4(z+1)^2}\right)^n$$

$$= \sum_{n=0}^{\infty}\frac{(2n)!}{(\mathrm{n}+1)}\frac{(z+1)_{2n}}{\left(\frac{3}{2}\right)_n n!}\left(-\frac{q^2L^2}{4(z+1)^2}\right)^n\sum_{l=0}^{n}\frac{4^l(\hat{q}_x^2)^l\left(\hat{q}_y^2\right)^{n-l}}{(2l)!\left((\mathrm{n}-\mathrm{l})!\right)^2}H_{2l,2n-2l}$$

Cylinder, polydisperse, axial distribution, x-axis, P(q)

For rapid calculations the q-independent coefficients are precalculated, such that the series can be quickly calculated for each pixel (q_x, q_y)

$$\langle P_{\|}(q_x,q_y,L)\rangle = \sum_{n=0}^{\infty}a_n\sum_{l=0}^{n}b_{l,n}(q_x^2)^l\left(q_y^2\right)^{n-l}$$

Mathematica code implementation:

```
del=0.1;
max=80;
ccc=Table[NIntegrate[((Cos[delta])^(2*l))*((Sin[delta])^(2*n-2*l+1))*Exp[-delta/del],
{delta, 0, Pi/2}]/NIntegrate[Exp[-delta/del]*Sin[delta], {delta, 0, Pi/2}], {n, 0, max},{l,
0, max}];

L=5;
R=1;
sigma=0.1;
z=(1-sigma*sigma)/(sigma*sigma);
phi1=0.1*Pi/2;
phi2=0.9*Pi/2;
qs=10^lqs;
qq=Sqrt[qx*qx+qy*qy];
qxs1=qs*Cos[phi1];
qys1=qs*Sin[phi1];
qxs2=qs*Cos[phi2];
qys2=qs*Sin[phi2];
(* isotropic formfactor *)
Pqiso=Sum[(4^n)*Pochhammer[z+1,2*n]*((-
qs*qs*L*L/(4*(z+1)*(z+1)))^n)/((2*n+1)*(n+1)*Pochhammer[3/2,n]*(n!)), {n, 0, 60}];
Pqisoa=Pi*(z+1)/(2*z*qs*L);
(* perfectly oriented formfactor *)
Pq1a1=Sum[(4^n)*Pochhammer[z+1,2*n]*((-
1/(4*(z+1)*(z+1)))^n)*((qxs1*L)^(2*n))/((n+1)*Pochhammer[3/2,n]*(n!)), {n, 0, max}];
Pq1a2=Sum[(4^n)*Pochhammer[z+1,2*n]*((-
1/(4*(z+1)*(z+1)))^n)*((qxs2*L)^(2*n))/((n+1)*Pochhammer[3/2,n]*(n!)), {n, 0, max}];
(* series expansion *)
Pq5a1=Sum[(((2*n)!)*Pochhammer[z+1,2*n]*((-
qs*qs*L*L/(4*(z+1)*(z+1)))^n)/((n+1)*Pochhammer[3/2,n]*(n!)))*Sum[(4^l)*((qxs1/qs)^(2*l))*(
(qys1/qs)^(2*(n-l)))*ccc[[n+1,l+1]]/(((2*l)!)*((n-l)!)*((n-l)!)), {l, 0, n}],{n, 0, max}];
Pq5a2=Sum[(((2*n)!)*Pochhammer[z+1,2*n]*((-
qs*qs*L*L/(4*(z+1)*(z+1)))^n)/((n+1)*Pochhammer[3/2,n]*(n!)))*Sum[(4^l)*((qxs2/qs)^(2*l))*(
(qys2/qs)^(2*(n-l)))*ccc[[n+1,l+1]]/(((2*l)!)*((n-l)!)*((n-l)!)), {l, 0, n}],{n, 0, max}];
(* numerical integration *)
Pq3=Sum[(((2*n)!)*Pochhammer[z+1,2*n]*((-
qq*qq*L*L/(4*(z+1)*(z+1)))^n)/((n+1)*Pochhammer[3/2,n]*(n!)))*Sum[(4^l)*((qx/qq)^(2*l))*((q
y/qq)^(2*(n-l)))*ccc[[n+1,l+1]]/(((2*l)!)*((n-l)!)*((n-l)!)), {l, 0, n}],{n, 0, max}];
(* numerical integration *)
u1=Gamma[z-1]/(2*Gamma[z+1]);
argz1=(L*(qxs1*Cos[delta]-qys1*Sin[chi]*Sin[delta])/(z+1));
argz2=(L*(qxs2*Cos[delta]-qys2*Sin[chi]*Sin[delta])/(z+1));
Pqintz1=NIntegrate[((argz1)^(-2))*(1-Cos[(z-1)*ArcTan[2*argz1]]/((1+4*argz1*argz1)^((z-
1)/2)))*Exp[-delta/del]*Sin[delta], {chi, 0, 2*Pi},{delta, 0, Pi/2}];
Pqintz2=NIntegrate[((argz2)^(-2))*(1-Cos[(z-1)*ArcTan[2*argz2]]/((1+4*argz2*argz2)^((z-
1)/2)))*Exp[-delta/del]*Sin[delta], {chi, 0, 2*Pi},{delta, 0, Pi/2}];
Pqintn=NIntegrate[Exp[-delta/del]*Sin[delta], {delta, 0, Pi/2}];
Pqint1=(u1/(2*Pi))*Pqintz1/Pqintn;
Pqint2=(u1/(2*Pi))*Pqintz2/Pqintn;
lim=1;
pl1=Plot[Log[10,Pq1a1], {lqs, -1, lim}, PlotRange->{-3,1}, GridLines->{Automatic},
LabelStyle->Directive[Black,16],AxesLabel->{"log q","lg P(q)"}, AxesOrigin->{-1,-
3},TicksStyle->Directive[Black,10],PlotStyle->{Blue,Dashed}];
pl2=Plot[Log[10,Pq1a2], {lqs, -1, lim}, PlotRange->{-3,1}, GridLines->{Automatic},
LabelStyle->Directive[Black,16],AxesLabel->{"log q","lg P(q)"}, AxesOrigin->{-1,-
3},TicksStyle->Directive[Black,10],PlotStyle->{Blue,Dashed}];
pl3=Plot[Log[10,Pq5a1], {lqs, -1, lim}, PlotRange->{-3,1}, GridLines->{Automatic},
LabelStyle->Directive[Black,16],AxesLabel->{"log q","lg P(q)"}, AxesOrigin->{-1,-
3},TicksStyle->Directive[Black,10],PlotStyle->{Black}];
pl4=Plot[Log[10,Pq5a2], {lqs, -1, lim}, PlotRange->{-3,1}, GridLines->{Automatic},
LabelStyle->Directive[Black,16],AxesLabel->{"log q","lg P(q)"}, AxesOrigin->{-1,-
3},TicksStyle->Directive[Black,10],PlotStyle->{Black}];
```

```
pl5=Plot[Log[10,Pqiso], {lqs, -1, lim}, PlotRange->{-3,1}, GridLines->{Automatic},
LabelStyle->Directive[Black,16],AxesLabel->{"log q","lg P(q)"}, AxesOrigin->{-1,-
3},TicksStyle->Directive[Black,10]];
pl6=Plot[Log[10,Pqisoa], {lqs, -1, lim}, PlotRange->{-3,1}, GridLines->{Automatic},
LabelStyle->Directive[Black,16],AxesLabel->{"log q","lg P(q)"}, AxesOrigin->{-1,-
3},TicksStyle->Directive[Black,10]];
(* pl7=Plot[Log[10,Pqint1], {lqs, -1, lim}, PlotRange⍰{-3,1}, GridLines->{Automatic},
LabelStyle->Directive[Black,16],AxesLabel->{"log q","lg P(q)"}, AxesOrigin->{-1,-
3},TicksStyle->Directive[Black,10],PlotStyle->{Red,Dashed}]
pl8=Plot[Log[10,Pqint2], {lqs, -1, lim}, PlotRange⍰{-3,1}, GridLines->{Automatic},
LabelStyle->Directive[Black,16],AxesLabel->{"log q","lg P(q)"}, AxesOrigin->{-1,-
3},TicksStyle->Directive[Black,10],PlotStyle->{Red,Dashed}] *)
Show[pl1,pl2,pl3,pl4,pl5,pl6]
lims=3;
pl5=DensityPlot[Log[10,Pq3],{qx,-lims,lims},{qy,-lims,lims},PlotRange->{-8,0},PlotPoints-
>50,PlotLegends->Automatic,LabelStyle->Directive[Black,12],AxesLabel->Automatic]
```

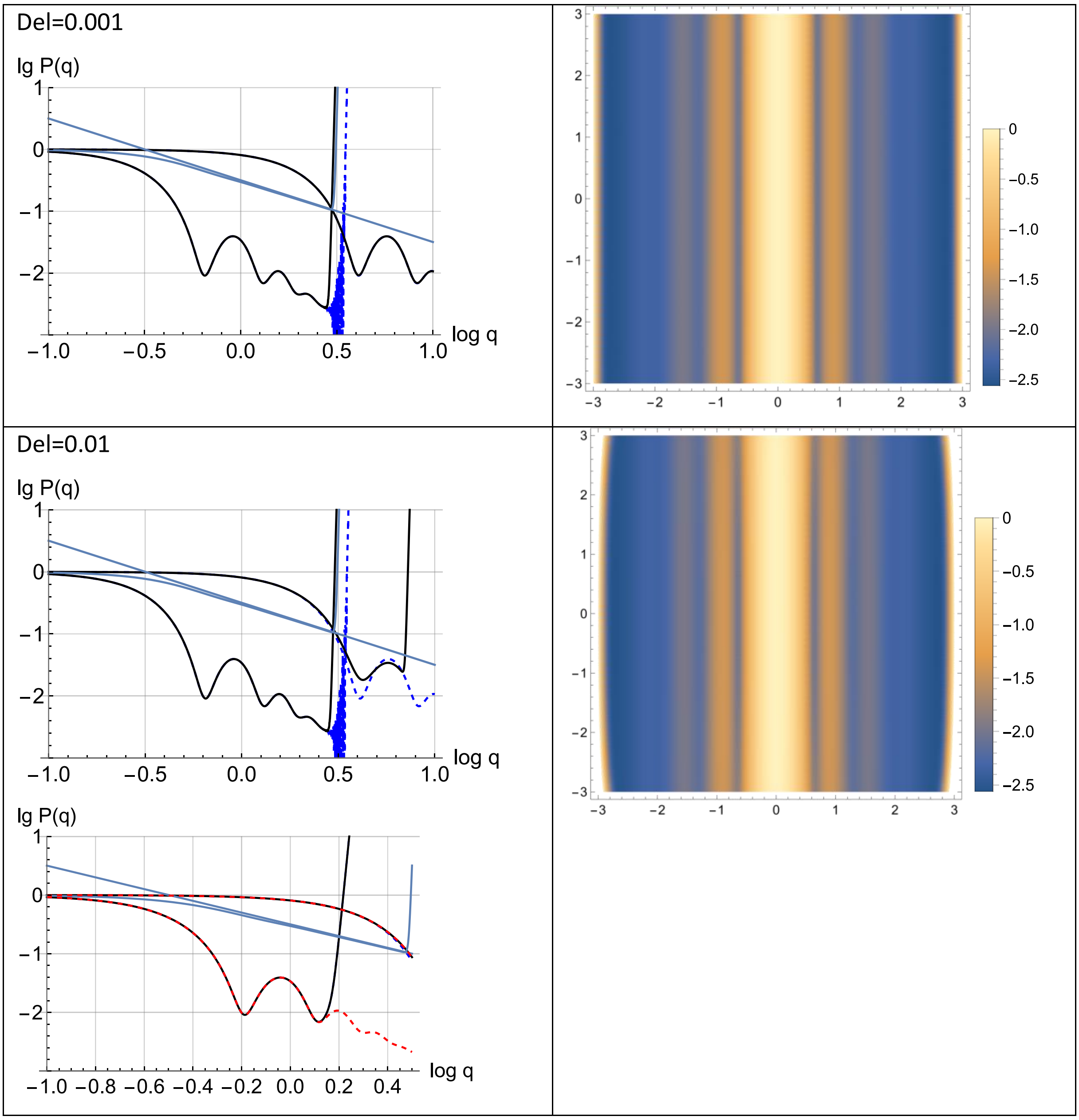

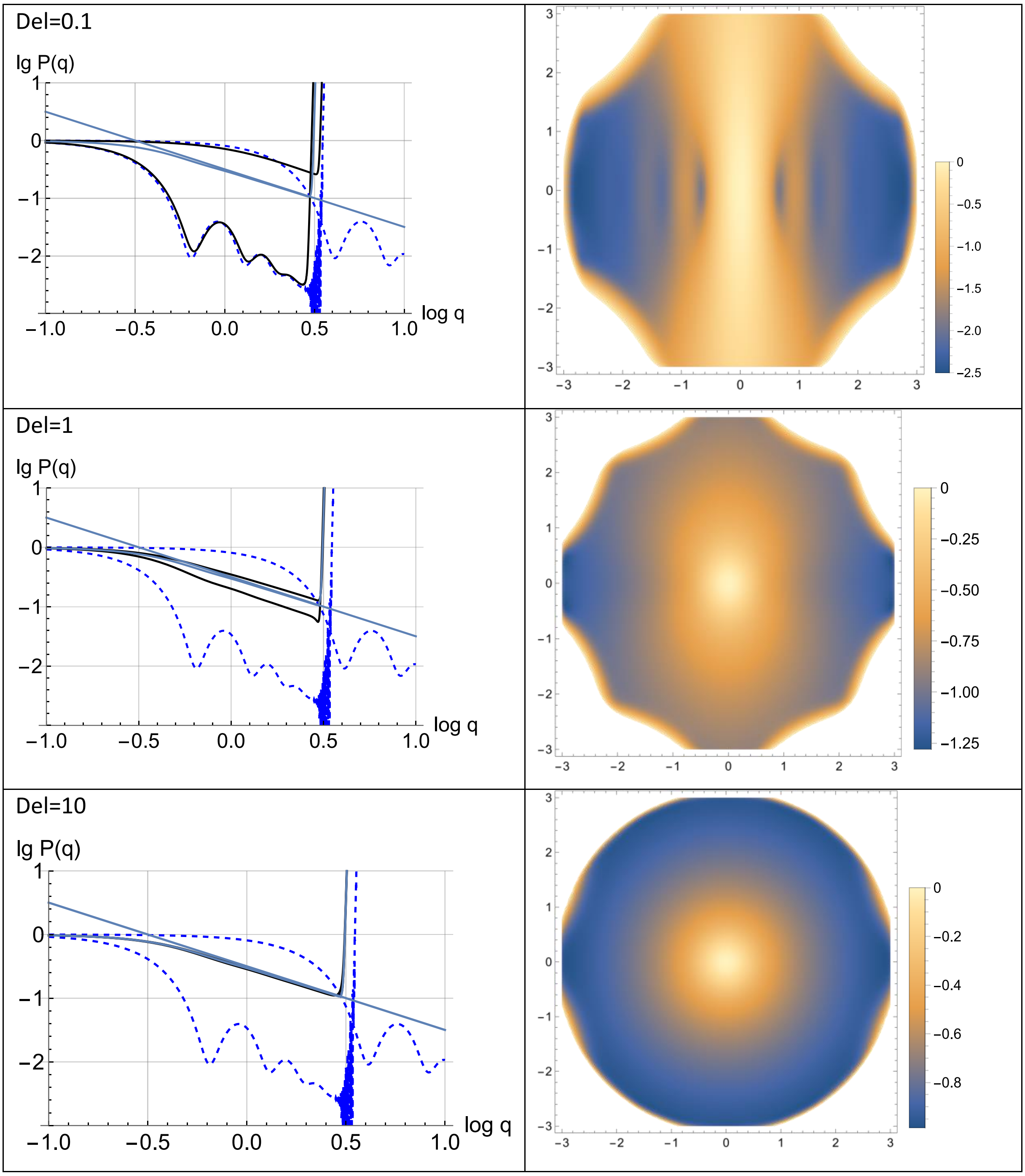

Fig. S52: Formfactor of oriented polydisperse cylinders with x-axial orientational distributions varying from perfectly oriented to the isotropic state.

Isotropic Limit

In the following we will show that the expression correctly yields the isotropic limit for the case $\bar{\delta} \to \infty$, for which $h(\delta) \to 1$. Then the integral over the deviation angle can be solved analytically to obtain

$$\int_0^{\pi/2} (\cos(\delta))^m (\sin(\delta))^{2n-m+1} d\delta = \frac{\Gamma\left[\frac{m+1}{2}\right]\Gamma\left[\frac{2n-m+2}{2}\right]}{2\Gamma\left[\frac{2n+3}{2}\right]}$$

This can be inserted into Eq. (S.7.4.1.5) using $m = 2l$ to obtain

$$\langle(\boldsymbol{qL})^{2n}\rangle = (qL)^{2n}\frac{(2n)!}{4^n}\sum_{l=0}^{n}\frac{4^l}{(2l)!\left((\mathrm{n}-\mathrm{l})!\right)^2}(\hat{q}_x^2)^l\left(\hat{q}_y^2\right)^{n-l}\frac{\Gamma\left[\frac{2l+1}{2}\right]\Gamma[n-l+1]}{2\Gamma\left[n+\frac{3}{2}\right]}$$

using Eq. (S.7.4.1.4) with $x = 2l + 1$ yields the simplified expression

$$\langle(\boldsymbol{qL})^{2n}\rangle = (qL)^{2n}\frac{(2n)!\sqrt{\pi}}{2n!\;4^n\Gamma\left[n+\frac{3}{2}\right]}\sum_{l=0}^{n}\frac{n!}{\mathrm{l}!\,(\mathrm{n}-\mathrm{l})!}(\hat{q}_x^2)^l\left(\hat{q}_y^2\right)^{n-l}$$

With the identity **(S.7.4.1.7)**

$$\frac{(2n)!\sqrt{\pi}}{n!\;4^n} = \Gamma\left[n+\frac{1}{2}\right]$$

we can further simplify the expression, re-expressing the binomial to obtain

$$\langle(\boldsymbol{qL})^{2n}\rangle = (qL)^{2n}\frac{1}{(2n+1)}\left(\hat{q}_x^2+\hat{q}_y^2\right)^n = (qL)^{2n}\frac{1}{(2n+1)}$$

This is the desired equation that is equal to the expression in Eq. (S.4.6.2.1.2) relating to the integral

$$\int_0^{\frac{\pi}{2}} (\cos(\phi))^{2n}\sin\phi\, d\phi = \frac{1}{2n+1}$$

Regime II. For intermediate and large q-values we use the numerical integration

$$\langle P_{\parallel}(q,L)\rangle = \frac{1}{2\pi H}\int_0^{\pi/2}\int_0^{2\pi}\langle\frac{(\sin(\boldsymbol{qL}))^2}{(\boldsymbol{qL})^2}\rangle\, h(\delta)(\sin(\delta)\, d\chi d\delta$$

$$H = \int_0^{\pi/2} h(\delta)(\sin(\delta)\, d\delta$$

where the integrand is computed in terms of the series expansion Eq. (S.1.2.40) as

$$\langle\left(\frac{\sin(\boldsymbol{qL})}{\boldsymbol{qL}}\right)^2\rangle_L = \sum_{n=0}^{\infty}\frac{4^n}{(\mathrm{n}+1)}\frac{(z_L+s_L+1)_{2n}}{\left(\frac{3}{2}\right)_n n!}\left(-\frac{\boldsymbol{q}^2\boldsymbol{L}^2}{4(z_L+1)^2}\right)^n$$

or via the asymptotic expansion Eq. (S.4.4.1.3) as

$$\langle\frac{(\sin(\boldsymbol{qL}))^2}{(\boldsymbol{qL})^2}\rangle = \frac{\Gamma[z-1]}{\Gamma[z+1]}\frac{1}{2}\left(\frac{z+1}{\boldsymbol{qL}}\right)^2\left(1+\frac{\cos\left[(z-1)\arctan\left(\frac{2\boldsymbol{qL}}{z+1}\right)\right]}{\left(1+\left(\frac{2\boldsymbol{qL}}{z+1}\right)^2\right)^{\frac{z-1}{2}}}\right)$$

with

$$\boldsymbol{qL} = qL\left[\hat{q}_x\cos(\delta) - \hat{q}_y\sin(\chi)\sin(\delta)\right]$$

Where for the integrand the expressions for Regime I, II, and III can be used, depending on the value of the argument $\boldsymbol{qL}$**.**

The function **(S.7.4.1.8)**

$$P(\boldsymbol{qL}) = \frac{1}{1+\frac{2\Gamma[z+1]}{\Gamma[z-1]}\left(\frac{\boldsymbol{qL}}{z+1}\right)^2}$$

can be well used as an approximation for polydisperse cases or for broad orientational distributions where the formfactor oscillations are smoothed out.

Mathematica code implementation:

```
L=1;
sigma1=0.1;
sigma2=0.3;
z1=(1-sigma1*sigma1)/(sigma1*sigma1);
z2=(1-sigma2*sigma2)/(sigma2*sigma2);
q=10^lq;
u1=Gamma[z1-1]/(2*Gamma[z1+1]);
u2=Gamma[z2-1]/(2*Gamma[z2+1]);
argz1=(L*q/(z1+1));
argz2=(L*q/(z2+1));
Pq1=u1*((argz1)^(-2))*(1-Cos[(z1-1)*ArcTan[2*argz1]]/((1+4*argz1*argz1)^((z1-1)/2)));
Pq2=u2*((argz2)^(-2))*(1-Cos[(z2-1)*ArcTan[2*argz2]]/((1+4*argz2*argz2)^((z2-1)/2)));
Gq1=1/(1+argz1*argz1/u1);
Gq2=1/(1+argz2*argz2/u2);
pl1=Plot[Log[10,Pq1],{lq,-1,2},PlotRange->{-5,1},GridLines->{Automatic},LabelStyle-
>Directive[Black,16],AxesLabel->{"log q","lg P(q)"},AxesOrigin->{-1,-5},TicksStyle-
>Directive[Black,10],PlotStyle->{Black}];
pl2=Plot[Log[10,Gq1],{lq,-1,2},PlotRange->{-5,1},GridLines->{Automatic},LabelStyle-
>Directive[Black,16],AxesLabel->{"log q","lg P(q)"},AxesOrigin->{-1,-5},TicksStyle-
>Directive[Black,10],PlotStyle->{Red, Dashed}];
pl3=Plot[Log[10,Pq2],{lq,-1,2},PlotRange->{-5,1},GridLines->{Automatic},LabelStyle-
>Directive[Black,16],AxesLabel->{"log q","lg P(q)"},AxesOrigin->{-1,-5},TicksStyle-
>Directive[Black,10],PlotStyle->{Blue}];
pl4=Plot[Log[10,Gq2],{lq,-1,2},PlotRange->{-5,1},GridLines->{Automatic},LabelStyle-
>Directive[Black,16],AxesLabel->{"log q","lg P(q)"},AxesOrigin->{-1,-5},TicksStyle-
>Directive[Black,10],PlotStyle->{Red, Dashed}];
```

```
Show[pl1,pl2,pl3,pl4]
```

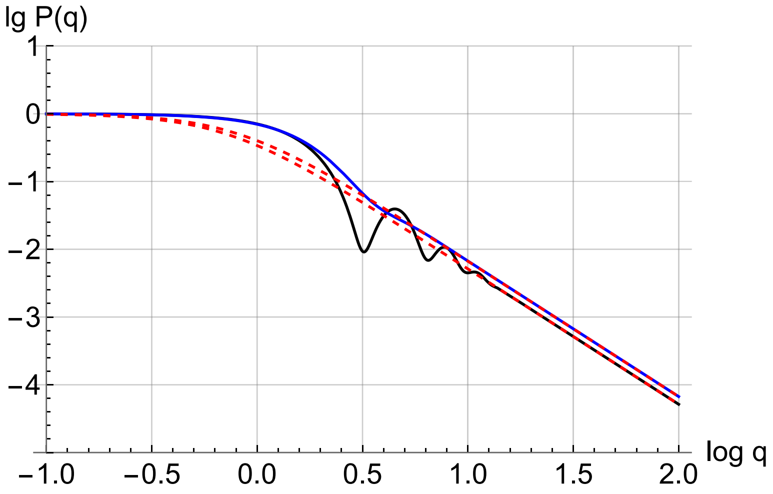

Fig. S53: Red dashed line shows approximation.

```
del=0.5;
max=70;
ccc=Table[NIntegrate[((Cos[delta])^(2*l))*((Sin[delta])^(2*n-2*l+1))*Exp[-
delta/del],{delta,0,Pi/2}]/NIntegrate[Exp[-
delta/del]*Sin[delta],{delta,0,Pi/2}],{n,0,max},{l,0,max}];

L=5;
sigma=0.1;
z=(1-sigma*sigma)/(sigma*sigma);
qs=10^lqs;
qq=Sqrt[qx*qx+qy*qy];

u1=Gamma[z-1]/(2*Gamma[z+1]);
argz1=(L*(qx*Cos[delta]-qy*Sin[chi]*Sin[delta])/(z+1));
Gqintz1=NIntegrate[(1/(1+argz1*argz1/u1))*Exp[-
delta/del]*Sin[delta],{chi,0,2*Pi},{delta,0,Pi/2}];
Pqintn=NIntegrate[Exp[-delta/del]*Sin[delta],{delta,0,Pi/2}];
Gqint1=(1/(2*Pi))*Gqintz1/Pqintn;

Pq3=Sum[(((2*n)!)*Pochhammer[z+1,2*n]*((-
qq*qq*L*L/(4*(z+1)*(z+1)))^n)/((n+1)*Pochhammer[3/2,n]*(n!)))*Sum[(4^l)*((qx/qq)^(2*l))*((q
y/qq)^(2*(n-l)))*ccc[[n+1,l+1]]/(((2*l)!)*((n-l)!)*((n-l)!)),{l,0,n}],{n,0,max}];
lims=3;
pl1=DensityPlot[Log[10,Pq3],{qx,-lims,lims},{qy,-lims,lims},PlotRange->{-8,0},PlotPoints-
>50,PlotLegends->Automatic,LabelStyle->Directive[Black,12],AxesLabel->Automatic]
pl2=DensityPlot[Log[10,Gqint1],{qx,-lims,lims},{qy,-lims,lims},PlotRange->{-
8,0},PlotPoints->50,PlotLegends->Automatic,LabelStyle->Directive[Black,12],AxesLabel-
>Automatic]
```

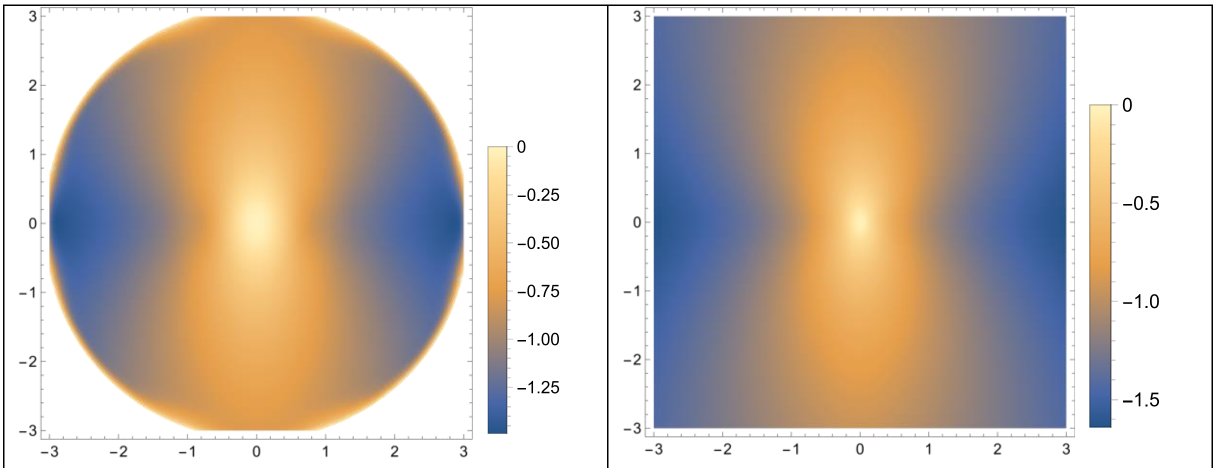

Series expansion | approximation

Fig. S54: Cylinder, x-axis, series expansion (left) and approximation (right).

7.4.2 Disks

For the series and asymptotic expansion, and for the Porod asymptote we need the averaged phase term $\boldsymbol{qR}$. For disks $\boldsymbol{R}$ represents the radial axis such that the phase term is given by (S.7.4.2.1)

$$\boldsymbol{qR} = qL\sin(\beta)$$

where β is the angle enclosed by the scattering vector $\boldsymbol{q}$ and the radial vector $\boldsymbol{R}$. This is similar for ellipsoids or parallelepipeds considering the alignment of the **a**-, or **b**-axis.

The enclosed angle β can be expressed in terms of the deviation and cone angles δ, χ as (S.7.4.2.2)

$$\boldsymbol{qR} = qR\sin(\beta) = qR\sin(\arccos[\boldsymbol{qMR}_\delta]) = qR\sqrt{1-(\boldsymbol{qMR}_\delta)^2}$$
$$= qR\sqrt{1-\left(\hat{q}_x\cos(\delta)-\hat{q}_y\sin(\chi)\sin(\delta)\right)^2}$$

with $\hat{q}_x = \frac{q_x}{q}$ and $\hat{q}_y = \frac{q_y}{q}$. For perfect orientation with $\delta = 0$ the expression reduces to

$$\boldsymbol{qR} = q_y R$$

For the formfactor we need to the expression $(\boldsymbol{qR})^{2n}$

$$(\boldsymbol{qR})^{2n} = (qr)^{2n}\left(1-\left(\hat{q}_x\cos(\delta)-\hat{q}_y\sin(\chi)\sin(\delta)\right)^2\right)^n$$

We use the binomial form

$$(\boldsymbol{qR})^{2n} = (qr)^{2n} \sum_{m=0}^{n} \binom{n}{m} (-1)^m \left(\hat{q}_x \cos(\delta) - \hat{q}_y \sin(\chi)\sin(\delta)\right)^{2m}$$

which can be expressed in terms of a second binomial

$$(\boldsymbol{qR})^{2n} = (qR)^{2n} \sum_{m=0}^{n} \binom{n}{m} (-1)^m \sum_{\boldsymbol{k=0}}^{\boldsymbol{2m}} \binom{2m}{k} (\hat{q}_x)^k \left(-\hat{q}_y\right)^{2m-k} [(\cos(\delta))^k (\sin(\chi)\sin(\delta))^{2m-k}]$$

The angular part [...] has to be integrated over the cone angle χ and the deviation angle δ

$$(\boldsymbol{qR})^{2n} = (qR)^{2n} \sum_{m=0}^{n} \binom{n}{m} (-1)^m \sum_{\boldsymbol{k=0}}^{\boldsymbol{2m}} \binom{2m}{k} (\hat{q}_x)^k (-\hat{q}_y)^{2m-k} \left(\left\{\frac{1}{2\pi}\int_{\boldsymbol{0}}^{2\pi} (\sin(\chi))^{2m-k} d\chi\right\}\right)\left(\int_{\boldsymbol{0}}^{\frac{\pi}{2}} (\cos(\delta))^k (\sin(\delta))^{2m-k+1} d\delta\right)$$

The integral of the cone angle χ can be solved analytically

$$\frac{1}{2\pi}\int_0^{2\pi} (\sin(\chi))^{2m-k} d\chi = \frac{2(1+(-1)^{2m-k})\Gamma\left[\frac{1}{2}\right]\Gamma\left[\frac{2m-k+1}{2}\right]}{4\pi\Gamma\left[\frac{2m-k+2}{2}\right]}$$

The integral of the deviation angle δ is given by (S.7.4.2.3)

$$\frac{\int_0^{\pi/2} (\cos(\delta))^k (\sin(\delta))^{2m-k} h(\delta)\sin(\delta)\, d\delta}{\int_0^{\pi/2} h(\delta)\sin(\delta)\, d\delta} = H_{k,2m-k+1}$$

We now have

$$(\boldsymbol{qR})^{2n} = (qR)^{2n} \sum_{m=0}^{n} \frac{(n)!\,(-1)^m}{m!\,(n-m)!} \sum_{k=0}^{2m} \frac{(2m)!}{k!\,(2m-k)!} \left(\hat{q}_x\right)^k \left(-\hat{q}_y\right)^{2m-k} \left\{\frac{(1+(-1)^{2m-k})\Gamma\left[\frac{1}{2}\right]\Gamma\left[\frac{2m-k+1}{2}\right]}{2\pi\Gamma\left[\frac{2m-k+2}{2}\right]}\right\} H_{k,2m-k+1}$$

We observe that there are contributions to the sum only if *m* is even, i.e. $m = 2l$. We therefore change the summation index from *m* to *l* and simplify to obtain

$$(\boldsymbol{qR})^{2n} = (qr)^{2n}(n)! \sum_{m=0}^{n} \frac{(2m)!\,(-1)^m}{m!\,(n-m)!} \sum_{l=0}^{m} \frac{(\hat{q}_x)^{2l}(-\hat{q}_y)^{2m-2l}}{(2l)!\,(2m-2l)!} \left\{\frac{\Gamma\left[\frac{2m-2l+1}{2}\right]}{\sqrt{\pi}\Gamma\left[\frac{2m-2l+2}{2}\right]}\right\} H_{2l,2m-2l+1}$$

We use Eq. (S.7.4.1.4) with $x = 2m - 2l + 1$ to remove all Gamma functions we finally obtain an expression that can be inserted into the expansions and asymptotes.

$$(\boldsymbol{qR})^{2n} = (qR)^{2n}(n)! \sum_{m=0}^{n} \frac{(2m)!\,(-1)^m}{4^m m!\,(n-m)!} \sum_{\boldsymbol{l=0}}^{\boldsymbol{m}} \frac{4^l (\hat{q}_x^2)^l \left(\hat{q}_y^2\right)^{m-l}}{(2l)!\left((\mathrm{m}-\mathrm{l})!\right)^2} H_{2l,2m-2l+1}$$

Regime I. This can then be inserted into the disk formfactor (Eq. 4.6.1.1.2), and simplified to **(S.7.4.2.4)**

$$P(q,R)=\sum_{n=0}^{\infty}\frac{4^{n+1}\Gamma\left(n+\frac{3}{2}\right)}{\sqrt{\pi}\Gamma(n+3)}\frac{(z+1)_{2n}}{(2)_n n!}(-1)^n\langle\left(\frac{\boldsymbol{qR}}{2}\right)^{2n}\rangle$$

$$=\sum_{n=0}^{\infty}\frac{4^{n+1}\Gamma\left(n+\frac{3}{2}\right)}{\sqrt{\pi}\Gamma(n+3)}\frac{(z+1)_{2n}}{(2)_n}\left(-\frac{q^2R^2}{4(z+1)^2}\right)^n\sum_{m=0}^{n}\frac{(2m)!\,(-1)^m}{4^m m!\,(n-m)!}\sum_{\boldsymbol{l=0}}^{\boldsymbol{m}}\frac{4^l(\hat{q}_x^2)^l(\hat{q}_y^2)^{m-l}}{(2l)!\,((\mathrm{m}-\mathrm{l})!)^2}H_{2l,2m-2l+1}$$

Disk, polydisperse, uni-axial, x-axis, P(q)

Mathematica code implementation:

```
del=0.001;
max=30;
ccc=Table[NIntegrate[((Cos[delta])^(2*l))*((Sin[delta])^(2*n-2*l+1))*Exp[-delta/del],
{delta, 0, Pi/2}]/NIntegrate[Exp[-delta/del]*Sin[delta], {delta, 0, Pi/2}], {n, 0, max},{l,
0, max}];

L=1;
R=5;
sigma=0.1;
z=Sqrt[1-sigma*sigma]/(sigma*sigma);
phi1=0.1*Pi/2;
phi2=0.9*Pi/2;
qs=10^lqs;
qq=Sqrt[qx*qx+qy*qy];
qxs1=qs*Cos[phi1];
qys1=qs*Sin[phi1];
qxs2=qs*Cos[phi2];
qys2=qs*Sin[phi2];
(* isotropic formfactor *)
Pqiso=Sum[2*(4^n)*Pochhammer[z+1,2*n]*((-
qs*qs*R*R/(4*(z+1)*(z+1)))^n)/(Pochhammer[2,n]*((n+2)!)), {n, 0, 60}];
Pqisoa=2*Gamma[z-1]*((z+1)^2)/(Gamma[z+1]*qs*qs*R*R);
(* perfectly oriented formfactor *)
Pq1a1=Sum[(4^(n+1))*Gamma[n+3/2]*Pochhammer[z+1,2*n]*((-
1/(4*(z+1)*(z+1)))^n)*((qxs1*R)^(2*n))/(Sqrt[Pi]*((n+2)!)*Pochhammer[2,n]*(n!)), {n, 0,
max}];
Pq1a2=Sum[(4^(n+1))*Gamma[n+3/2]*Pochhammer[z+1,2*n]*((-
1/(4*(z+1)*(z+1)))^n)*((qxs2*R)^(2*n))/(Sqrt[Pi]*((n+2)!)*Pochhammer[2,n]*(n!)), {n, 0,
max}];
(* series expansion *)
Pq5a1=Sum[((4^(n+1))*Gamma[n+3/2]*Pochhammer[z+1,2*n]*((-
qs*qs*R*R/(4*(z+1)*(z+1)))^n)/(Sqrt[Pi]*((n+2)!)*Pochhammer[2,n]))*Sum[(((2*m)!)*((-
1)^m)/((4^m)*(m!)*((n-m)!)))*Sum[(4^l)*((qxs1/qs)^(2*l))*((qys1/qs)^(2*(m-
l)))*ccc[[m+1,l+1]]/(((2*l)!)*((m-l)!)*((m-l)!)), {l, 0, m}],{m, 0, n}],{n, 0, max}];
Pq5a2=Sum[((4^(n+1))*Gamma[n+3/2]*Pochhammer[z+1,2*n]*((-
qs*qs*R*R/(4*(z+1)*(z+1)))^n)/(Sqrt[Pi]*((n+2)!)*Pochhammer[2,n]))*Sum[(((2*m)!)*((-
1)^m)/((4^m)*(m!)*((n-m)!)))*Sum[(4^l)*((qxs2/qs)^(2*l))*((qys2/qs)^(2*(m-
l)))*ccc[[m+1,l+1]]/(((2*l)!)*((m-l)!)*((m-l)!)), {l, 0, m}],{m, 0, n}],{n, 0, max}];
(* 2D *)
Pq3=Sum[((4^(n+1))*Gamma[n+3/2]*Pochhammer[z+1,2*n]*((-
qq*qq*R*R/(4*(z+1)*(z+1)))^n)/(Sqrt[Pi]*((n+2)!)*Pochhammer[2,n]))*Sum[(((2*m)!)*((-
1)^m)/((4^m)*(m!)*((n-m)!)))*Sum[(4^l)*((qx/qq)^(2*l))*((qy/qq)^(2*(m-
l)))*ccc[[m+1,l+1]]/(((2*l)!)*((m-l)!)*((m-l)!)), {l, 0, m}],{m, 0, n}],{n, 0, max}];
(* numerical integration *)
u1=Gamma[z-1]/(2*Gamma[z+1]);
(* argz1=(L*(qxs1*Cos[delta]-qys1*Sin[chi]*Sin[delta])/(z+1));
```

```
argz2=(L*(qxs2*Cos[delta]-qys2*Sin[chi]*Sin[delta])/(z+1));
Pqintz1=NIntegrate[((argz1)^(-2))*(1-Cos[(z-1)*ArcTan[2*argz1]]/((1+4*argz1*argz1)^((z-
1)/2)))*Exp[-delta/del]*Sin[delta], {chi, 0, 2*Pi},{delta, 0, Pi/2}];
Pqintz2=NIntegrate[((argz2)^(-2))*(1-Cos[(z-1)*ArcTan[2*argz2]]/((1+4*argz2*argz2)^((z-
1)/2)))*Exp[-delta/del]*Sin[delta], {chi, 0, 2*Pi},{delta, 0, Pi/2}];
Pqintn=NIntegrate[Exp[-delta/del]*Sin[delta], {delta, 0, Pi/2}];
Pqint1=(u1/(2*Pi))*Pqintz1/Pqintn;
Pqint2=(u1/(2*Pi))*Pqintz2/Pqintn; *)
lim=1;
pl1=Plot[Log[10,Pq1a1], {lqs, -1, lim}, PlotRange->{-3,1}, GridLines->{Automatic},
LabelStyle->Directive[Black,16],AxesLabel->{"log q","lg P(q)"}, AxesOrigin->{-1,-
3},TicksStyle->Directive[Black,10],PlotStyle->{Blue,Dashed}];
pl2=Plot[Log[10,Pq1a2], {lqs, -1, lim}, PlotRange->{-3,1}, GridLines->{Automatic},
LabelStyle->Directive[Black,16],AxesLabel->{"log q","lg P(q)"}, AxesOrigin->{-1,-
3},TicksStyle->Directive[Black,10],PlotStyle->{Blue,Dashed}];
pl3=Plot[Log[10,Pq5a1], {lqs, -1, lim}, PlotRange->{-3,1}, GridLines->{Automatic},
LabelStyle->Directive[Black,16],AxesLabel->{"log q","lg P(q)"}, AxesOrigin->{-1,-
3},TicksStyle->Directive[Black,10],PlotStyle->{Black}];
pl4=Plot[Log[10,Pq5a2], {lqs, -1, lim}, PlotRange->{-3,1}, GridLines->{Automatic},
LabelStyle->Directive[Black,16],AxesLabel->{"log q","lg P(q)"}, AxesOrigin->{-1,-
3},TicksStyle->Directive[Black,10],PlotStyle->{Black}];
pl5=Plot[Log[10,Pqiso], {lqs, -1, lim}, PlotRange->{-3,1}, GridLines->{Automatic},
LabelStyle->Directive[Black,16],AxesLabel->{"log q","lg P(q)"}, AxesOrigin->{-1,-
3},TicksStyle->Directive[Black,10]];
pl6=Plot[Log[10,Pqisoa], {lqs, -1, lim}, PlotRange->{-3,1}, GridLines->{Automatic},
LabelStyle->Directive[Black,16],AxesLabel->{"log q","lg P(q)"}, AxesOrigin->{-1,-
3},TicksStyle->Directive[Black,10]];
(* pl7=Plot[Log[10,Pqint1], {lqs, -1, lim}, PlotRange⍰{-3,1}, GridLines->{Automatic},
LabelStyle->Directive[Black,16],AxesLabel->{"log q","lg P(q)"}, AxesOrigin->{-1,-
3},TicksStyle->Directive[Black,10],PlotStyle->{Red,Dashed}]
pl8=Plot[Log[10,Pqint2], {lqs, -1, lim}, PlotRange⍰{-3,1}, GridLines->{Automatic},
LabelStyle->Directive[Black,16],AxesLabel->{"log q","lg P(q)"}, AxesOrigin->{-1,-
3},TicksStyle->Directive[Black,10],PlotStyle->{Red,Dashed}] *)
Show[pl1,pl2,pl3,pl4,pl5,pl6]
lims=3;
pl5=DensityPlot[Log[10,Pq3],{qx,-lims,lims},{qy,-lims,lims},PlotRange->{-8,0},PlotPoints-
>50,PlotLegends->Automatic,LabelStyle->Directive[Black,12],AxesLabel->Automatic]
```

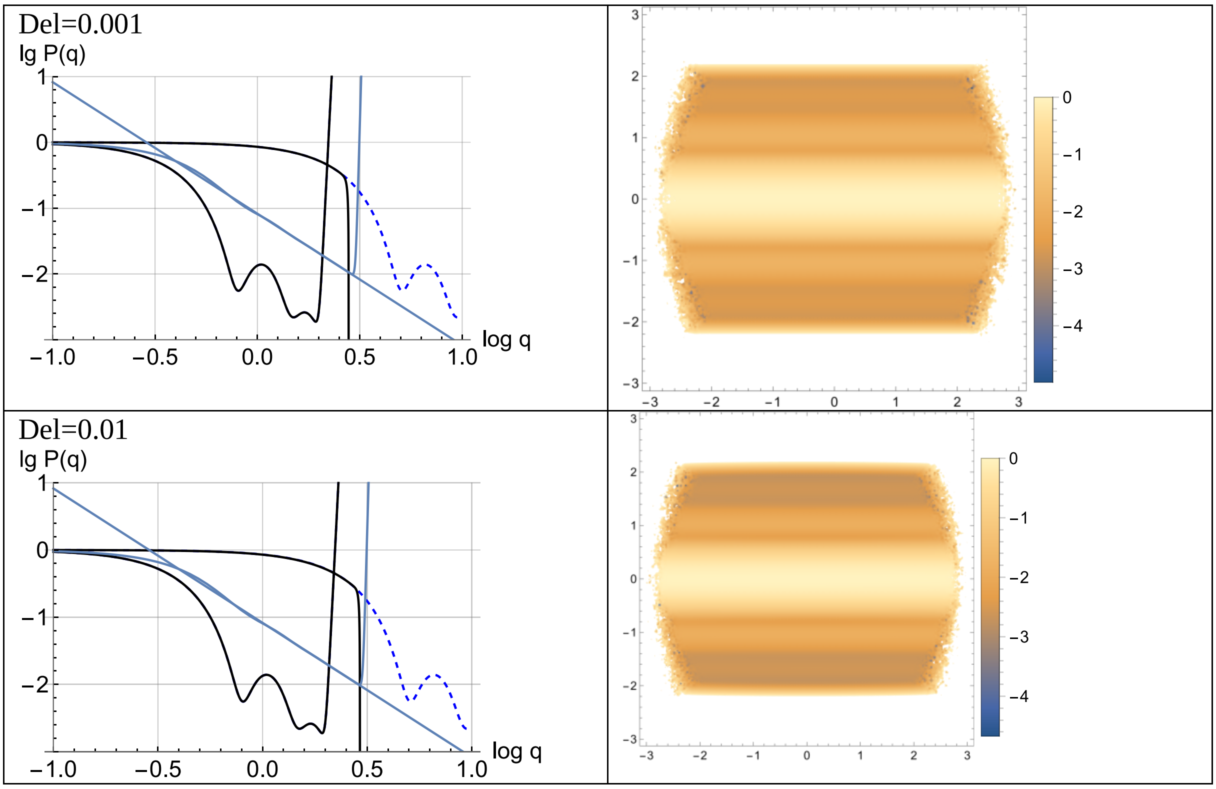

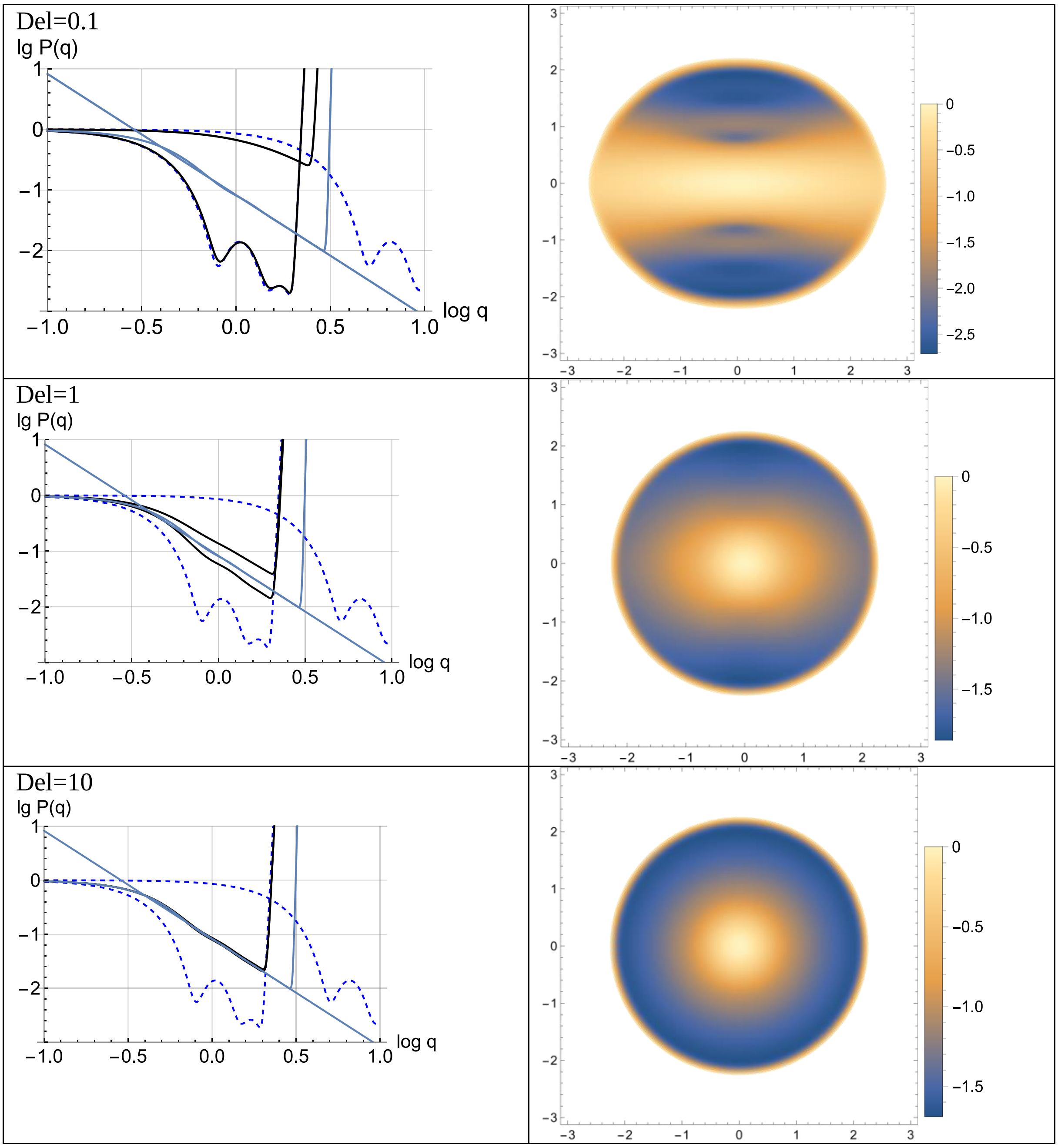

Fig. S55: Formfactor of oriented polydisperse disks with x-axial orientational distributions varying from perfectly oriented to the isotropic state.

For rapid calculations the q-independent coefficients are precalculated, such that the series can be quickly calculated for each pixel (q_x, q_y)

$$\langle P_{\parallel}(q_x, q_y, L)\rangle = \sum_{n=0}^{\infty} a_n \sum_{l=0}^{n} b_{n,m} \sum_{l=0}^{m} c_{m,l} (q_x^2)^l (q_y^2)^{m-l}$$

Isotropic Limit

In the following we will show that the expression correctly yields the isotropic limit for the case $\bar{\delta} \to \infty$, for which $h(\delta) \to 1$. Then the integral over the deviation angle can be solved analytically to obtain

$$H_{2l,2m-2l+1} = \int_0^{\pi/2} (\cos(\delta))^{2l}(\sin(\delta))^{2m-2l+1} d\delta = \frac{\Gamma\left[\frac{2l+1}{2}\right]\Gamma\left[\frac{2m-2l+2}{2}\right]}{2\Gamma\left[\frac{2m+3}{2}\right]}$$

This can be inserted into Eq. (S.7.4.2.4) using $m = 2l$ to obtain

$$(\boldsymbol{qR})^{2n} = (qR)^{2n}(n)! \sum_{m=0}^{n} \frac{(2m)!\,(-1)^m}{4^m m!\,(n-m)!} \sum_{\boldsymbol{l=0}}^{\boldsymbol{m}} \frac{4^l \left(\hat{q}_x^2\right)^l \left(\hat{q}_y^2\right)^{m-l}}{\Gamma[2l+1](\mathrm{m}-\mathrm{l})!} \frac{\Gamma\left[\frac{2l+1}{2}\right]}{2\Gamma\left[\frac{2m+3}{2}\right]}$$

using Eq. (S.7.4.1.4) with $x = 2l + 1$ yields the simplified expression

$$(\boldsymbol{qR})^{2n} = (qR)^{2n}(n)!\sqrt{\pi} \sum_{m=0}^{n} \frac{(2m)!\,(-1)^m}{4^m m!\,(n-m)!\,2\Gamma\left[\frac{2m+3}{2}\right] m!} \sum_{\boldsymbol{l=0}}^{\boldsymbol{m}} \frac{\left(\hat{q}_x^2\right)^l \left(\hat{q}_y^2\right)^{m-l}}{(\mathrm{m}-\mathrm{l})!} \frac{m!}{(\mathrm{l}!)}$$

to re-express the binomial to obtain

$$(\boldsymbol{qR})^{2n} = (qR)^{2n}(n)! \sum_{m=0}^{n} \frac{(-1)^m}{m!\,(n-m)!\,2} \frac{(2m)!\sqrt{\pi}}{m!\,4^m\Gamma\left[\frac{2m+3}{2}\right]} \left(\hat{q}_x^2 + \hat{q}_y^2\right)^m$$

It can be further reduces with Eq. (S.7.4.1.7) to result in

$$(\boldsymbol{qR})^{2n} = (qr)^{2n} \frac{(n)!}{2} \sum_{m=0}^{n} \frac{(-1)^m}{m!\,(n-m)!} \frac{1}{\left(m+\frac{1}{2}\right)}$$

With the identity **(S.7.4.2.5)**

$$\sum_{m=0}^{n} \frac{(-1)^m}{\left(m+\frac{1}{2}\right) m!\,(n-m)!} = \frac{\sqrt{\pi}}{\Gamma\left[n+\frac{3}{2}\right]}$$

we finally obtain the relation

$$(\boldsymbol{qR})^{2n} = (qR)^{2n} \frac{\sqrt{\pi}(n)!}{2\Gamma\left[n+\frac{3}{2}\right]}$$

This is the desired equation that is equal to the expression in Eq. (S.4.7.1.2) relating to the integral

$$\int_0^{\frac{\pi}{2}} (\sin(\phi))^{2n} \sin\phi \, d\phi = \frac{\sqrt{\pi}\Gamma(n+1)}{2\Gamma\left(n+\frac{3}{2}\right)} = \frac{\sqrt{\pi}(n)!}{2\Gamma\left(n+\frac{3}{2}\right)}$$

Regime II. For intermediate and large q-values we use the numerical integration

$$P^{(2)}(q_r R) = \langle P_\perp(q_r R)\rangle = \frac{1}{2\pi H}\int_0^{\pi/2}\int_0^{2\pi} \langle\left(\frac{2J_1(\boldsymbol{qR})}{\boldsymbol{qR}}\right)^2\rangle\, h(\delta)(\sin(\delta)\, d\chi d\delta$$

$$H = \int_0^{\pi/2} h(\delta)(\sin(\delta)\, d\delta$$

where the integrand is computed in terms of the series expansion Eq. (S.1.2.53) as

$$\langle\left(\frac{2J_1(\boldsymbol{qR})}{\boldsymbol{qR}}\right)^2\rangle = \sum_{n=0}^{\infty} \frac{4^{n+1}\Gamma\left(n+\frac{3}{2}\right)}{\sqrt{\pi}\Gamma(n+3)} \frac{(z_R+s_R+1)_{2n}}{(2)_n n!}\left(-\frac{\boldsymbol{q}^2\boldsymbol{R}^2}{4(z_R+1)^2}\right)^n$$

or via the asymptotic expansion Eq. (S.1.2.54) as

$$\langle\left(\frac{2J_1(\boldsymbol{qR})}{\boldsymbol{qR}}\right)^2\rangle = \frac{4}{\pi}\left(\left(\frac{1}{\boldsymbol{qR}}\right)^3 - \left(\frac{1}{\boldsymbol{qR}}\right)^3 \sin(2\boldsymbol{qR}) - \frac{9}{8}\left(\frac{1}{\boldsymbol{qR}}\right)^4 \cos(2\boldsymbol{qR}) + \left(\frac{9}{16}\right)^2\left(\frac{1}{\boldsymbol{qR}}\right)^5 + \left(\frac{9}{16}\right)^2\left(\frac{1}{\boldsymbol{qR}}\right)^5 \sin(2\boldsymbol{qR})\right)$$

with

$$\boldsymbol{qR} = qR\sqrt{1-\left(\hat{q}_x\cos(\delta) - \hat{q}_y\sin(\chi)\sin(\delta)\right)^2}$$

Where for the integrand the expressions for regime I, II, and III can be used, depending on the value of the argument $\boldsymbol{qR}$**.**

The function **(S.7.4.2.6)**

$$P(\boldsymbol{qR}) = \frac{1}{1+\frac{\pi\Gamma[z+1]}{4\Gamma[z-2]}\left(\frac{\boldsymbol{qR}}{z+1}\right)^3}$$

can be well uased as an approximation for polydisperse cases or broad orientational distributions where the formfactor oscillations are smoothed out.

Mathematica code implementation:

```
R=1;
sigma1=0.1;
sigma2=0.3;
z1=(1-sigma1*sigma1)/(sigma1*sigma1);
z2=(1-sigma2*sigma2)/(sigma2*sigma2);
q=10^lqr;
qr1=q*R/(z1+1);
```

```
qr2=q*R/(z2+1);
f11=(Gamma[z1-2]/(Gamma[z1+1]))*(qr1^(-3));
f12=(Gamma[z1-2]/(Gamma[z1+1]))*(qr1^(-3))*Sin[(z1-2)*ArcTan[2*qr1]]/((1+4*qr1*qr1)^((z1-
2)/2));
f13=(Gamma[z1-3]/(Gamma[z1+1]))*(qr1^(-4))*Cos[(z1-3)*ArcTan[2*qr1]]/((1+4*qr1*qr1)^((z1-
3)/2));
f14=(Gamma[z1-4]/(Gamma[z1+1]))*(qr1^(-5));
f15=(Gamma[z1-4]/(Gamma[z1+1]))*(qr1^(-5))*Sin[(z1-4)*ArcTan[2*qr1]]/((1+4*qr1*qr1)^((z1-
4)/2));
Pq1=(4/Pi)*(f11-f12-(9/8)*f13+(9/16)*(9/16)*f14+(9/16)*(9/16)*f15);
f21=(Gamma[z2-2]/(Gamma[z2+1]))*(qr2^(-3));
f22=(Gamma[z2-2]/(Gamma[z2+1]))*(qr2^(-3))*Sin[(z2-2)*ArcTan[2*qr2]]/((1+4*qr2*qr2)^((z2-
2)/2));
f23=(Gamma[z2-3]/(Gamma[z2+1]))*(qr2^(-4))*Cos[(z2-3)*ArcTan[2*qr2]]/((1+4*qr2*qr2)^((z2-
3)/2));
f24=(Gamma[z2-4]/(Gamma[z2+1]))*(qr2^(-5));
f25=(Gamma[z2-4]/(Gamma[z2+1]))*(qr2^(-5))*Sin[(z2-4)*ArcTan[2*qr2]]/((1+4*qr2*qr2)^((z2-
4)/2));
Pq2=(4/Pi)*(f21-f22-(9/8)*f23+(9/16)*(9/16)*f24+(9/16)*(9/16)*f25);
argz1=q*R/(z1+1);
argz2=q*R/(z2+1);
u1=Pi*Gamma[z1+1]/(4*Gamma[z1-2]);
u2=Pi*Gamma[z2+1]/(4*Gamma[z2-2]);
gq1=1/(1+u1*((argz1)^3));
gq2=1/(1+u2*((argz2)^3));

pl1=Plot[Log[10,Pq1],{lqr,-1,2},PlotRange->{-6,1},GridLines->{Automatic},LabelStyle-
>Directive[Black,16],AxesLabel->{"log q","lg P(q)"},AxesOrigin->{-1,-6},TicksStyle-
>Directive[Black,10],PlotStyle->{Black}];
pl2=Plot[Log[10,gq1],{lqr,-1,2},PlotRange->{-6,1},GridLines->{Automatic},LabelStyle-
>Directive[Black,16],AxesLabel->{"log q","lg P(q)"},AxesOrigin->{-1,-6},TicksStyle-
>Directive[Black,10],PlotStyle->{Red, Dashed}];
pl3=Plot[Log[10,Pq2],{lqr,-1,2},PlotRange->{-6,1},GridLines->{Automatic},LabelStyle-
>Directive[Black,16],AxesLabel->{"log q","lg P(q)"},AxesOrigin->{-1,-6},TicksStyle-
>Directive[Black,10],PlotStyle->{Blue}];
pl4=Plot[Log[10,gq2],{lqr,-1,2},PlotRange->{-6,1},GridLines->{Automatic},LabelStyle-
>Directive[Black,16],AxesLabel->{"log q","lg P(q)"},AxesOrigin->{-1,-6},TicksStyle-
>Directive[Black,10],PlotStyle->{Red, Dashed}];
Show[pl1,pl2,pl3,pl4]
```

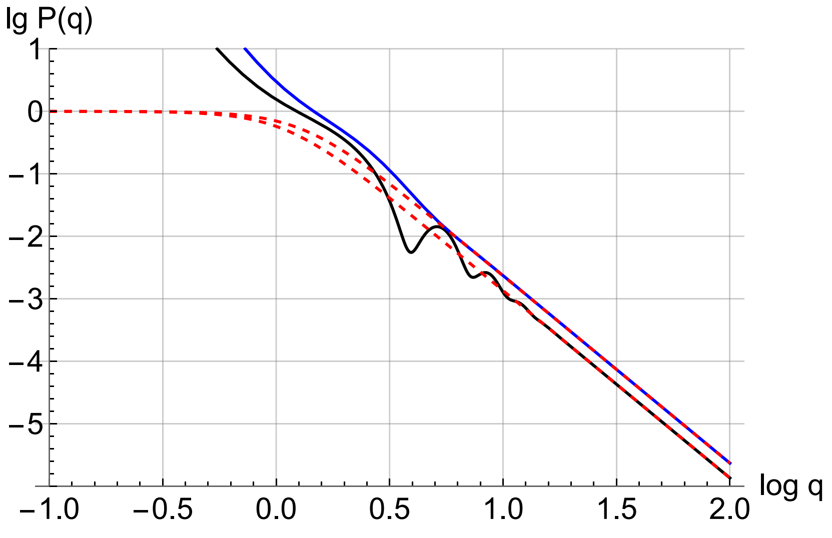

Fig. S56: Red dashed line shows approximation

```
del=0.5;
max=55;
```

```
ccc=Table[NIntegrate[((Cos[delta])^(2*l))*((Sin[delta])^(2*n-2*l+1))*Exp[-delta/del],
{delta, 0, Pi/2}]/NIntegrate[Exp[-delta/del]*Sin[delta], {delta, 0, Pi/2}], {n, 0, max},{l,
0, max}];

R=5;
sigma=0.1;
z=(1-sigma*sigma)/(sigma*sigma);
qq=Sqrt[qx*qx+qy*qy];
Pq3=Sum[((4^(n+1))*Gamma[n+3/2]*Pochhammer[z+1,2*n]*((-
qq*qq*R*R/(4*(z+1)*(z+1)))^n)/(Sqrt[Pi]*((n+2)!)*Pochhammer[2,n]))*Sum[(((2*m)!)*((-
1)^m)/((4^m)*(m!)*((n-m)!)))*Sum[(4^l)*((qx/qq)^(2*l))*((qy/qq)^(2*(m-
l)))*ccc[[m+1,l+1]]/(((2*l)!)*((m-l)!)*((m-l)!)),{l,0,m}],{m,0,n}],{n,0,max}];

u1=Pi*Gamma[z+1]/(4*Gamma[z-2]);
qxx=qx/qq;
qyy=qy/qq;
argz1=qq*R*Sqrt[1-(qxx*Cos[delta]-qyy*Sin[chi]*Sin[delta])^2]/(z+1);
Gqintz1=NIntegrate[(1/(1+u1*((argz1)^3)))*Exp[-
delta/del]*Sin[delta],{chi,0,2*Pi},{delta,0,Pi/2}];
Pqintn=NIntegrate[Exp[-delta/del]*Sin[delta],{delta,0,Pi/2}];
Gqint1=(1/(2*Pi))*Gqintz1/Pqintn;

lims=3;
pl1=DensityPlot[Log[10,Pq3],{qx,-lims,lims},{qy,-lims,lims},PlotRange->{-8,0},PlotPoints-
>50,PlotLegends->Automatic,LabelStyle->Directive[Black,12],AxesLabel->Automatic]
pl2=DensityPlot[Log[10,Gqint1],{qx,-lims,lims},{qy,-lims,lims},PlotRange->{-
8,0},PlotPoints->50,PlotLegends->Automatic,LabelStyle->Directive[Black,12],AxesLabel-
>Automatic]
```

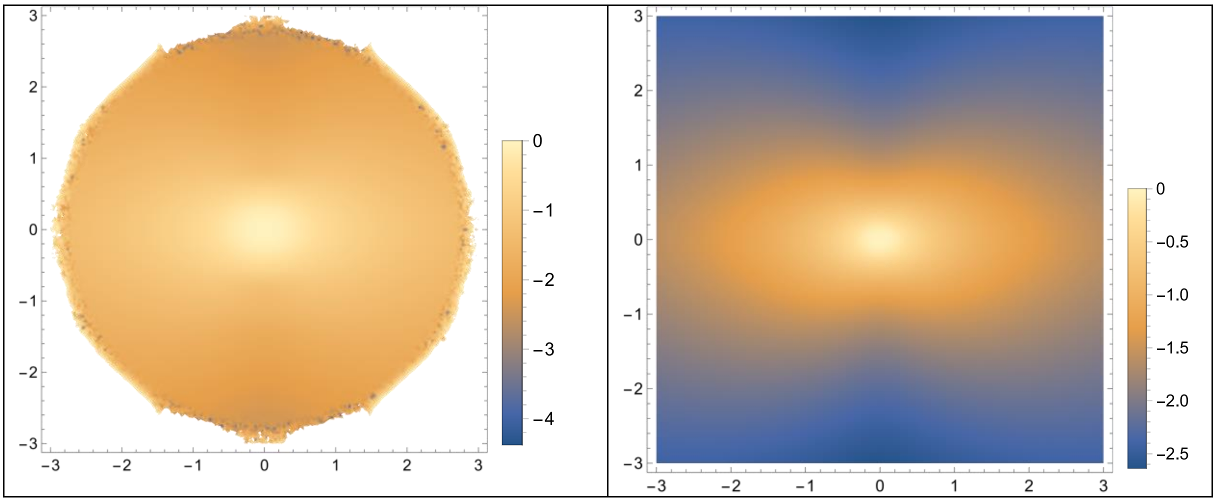

Fig. S57: Disk, x-axis, series expansion (left) and approximation (right)

7.5 Alignment parallel to y-axis

The case that $\boldsymbol{D} \parallel y$ corresponds to the angles $\varphi = \pi/2$, $\theta = \pi/2$. The director $\boldsymbol{D}$, axis-vector $\boldsymbol{L}_\delta$, the matrix $\boldsymbol{M}$, the vector $\boldsymbol{L}(\delta,\chi)$, and the phase $\boldsymbol{qL}(\delta,\chi)$ are then given by

$$\boldsymbol{D}=\begin{pmatrix}0\\1\\0\end{pmatrix}\quad \boldsymbol{L}_\delta=L\begin{pmatrix}0\\\cos(\delta)\\-\sin(\delta)\end{pmatrix}\quad \boldsymbol{M}=\begin{pmatrix}c\chi & 0 & -s\chi\\0 & 1 & 0\\s\chi & 0 & c\chi\end{pmatrix}\quad \boldsymbol{L}(\delta,\chi)=L\begin{pmatrix}\sin(\chi)\sin(\delta)\\\cos(\delta)\\-\cos(\chi)\sin(\delta)\end{pmatrix}$$

$$\boldsymbol{qL}(\delta,\chi)=\boldsymbol{qML}_\delta=q_x\sin(\chi)\sin(\delta)+q_y\cos(\delta)-q_z\cos(\chi)\sin(\delta)$$

In the small-angle approximation we have $q_z\approx 0$ such that

$$MI_0q\approx q_x\sin(\chi)\sin(\delta)+q_y\cos(\delta)$$

For perfect orientation with $\delta=0$ the expression reduces to $\boldsymbol{qL}(\delta,\chi)=q_yL$.

7.5.1 Cylinders

For the series and asymptotic expansion, and for the Porod asymptote we need the averaged phase term $\boldsymbol{qL}$. For cylinders L represents the long axis such that the phase term is given by (S.7.5.1.1)

$$\boldsymbol{qL}=qL\cos(\beta)$$

where β is the angle enclosed by the scattering vector $\boldsymbol{q}$ and the axis vector $\boldsymbol{L}$.

The enclosed angle β can be expressed in terms of the deviation and cone angles δ,χ as (S.7.5.1.2)

$$\boldsymbol{qL}=qL\cos(\beta)=qL\cos(\arccos[MI_0q])=qL\left[q_x\sin(\chi)\sin(\delta)+q_y\cos(\delta)\right]$$

with $\hat{q}_x=\frac{q_x}{q}$ and $\hat{q}_y=\frac{q_y}{q}$. For perfect orientation with $\delta=0$ the expression reduces to

$$\boldsymbol{qL}=q_yL$$

For the formfactor we need to the expression $(\boldsymbol{qL})^{2n}$

$$(\boldsymbol{qL})^{2n}=(qL)^{2n}\left(q_x\sin(\chi)\sin(\delta)+q_y\cos(\delta)\right)^{2n}$$

We use the binomial form

$$(\boldsymbol{qL})^{2n}=(qL)^{2n}\sum_{m=0}^{2n}\binom{2n}{m}\left(q_x\right)^m\left(q_y\right)^{2n-m}\left[(\sin(\chi)\sin(\delta))^m(\cos(\delta))^{2n-m}\right]$$

The angular part […] has to be integrated over the cone angle χ and the deviation angle δ

$$(\boldsymbol{qL})^{2n}=(qL)^{2n}\sum_{m=0}^{2n}\binom{2n}{m}\left(q_x\right)^m\left(q_y\right)^{2n-m}\left(\frac{1}{2\pi}\int_{\mathbf{0}}^{2\pi}(\sin(\chi))^m\boldsymbol{d}\chi\right)\left(\int_{\mathbf{0}}^{\frac{\pi}{2}}(\cos(\delta))^{2n-m}(\sin(\delta))^m\sin(\delta)\,d\delta\right)$$

The integral of the cone angle χ can be solved analytically

$$\frac{1}{2\pi}\int_0^{2\pi}(\sin(\chi))^m d\chi = \frac{(1+(-1)^m)\Gamma\left[\frac{1}{2}\right]\Gamma\left[\frac{m+1}{2}\right]}{2\pi\Gamma\left[\frac{m+2}{2}\right]}$$

The integral of the deviation angle δ is given by (S.7.5.1.3)

$$\frac{\int_0^{\pi/2}(\cos(\delta))^{2n-m}(\sin(\delta))^m h(\delta)\sin(\delta)\,d\delta}{\int_0^{\pi/2}h(\delta)\sin(\delta)\,d\delta} = H_{2n-m,m+1}$$

We now have

$$(\boldsymbol{qL})^{2n} = (qL)^{2n}\sum_{m=0}^{2n}\frac{(2n)!}{m!\,(2n-m)!}(q_x)^m(q_y)^{2n-m}\frac{(1+(-1)^m)\Gamma\left[\frac{1}{2}\right]\Gamma\left[\frac{m+1}{2}\right]}{2\pi\Gamma\left[\frac{m+2}{2}\right]}H_{2n-m,m+1}$$

We observe that there are contributions to the sum only if *m* is even, i.e. $m = 2l$. We therefore change the summation index from *m* to *l* and simplify to obtain

$$(\boldsymbol{qL})^{2n} = (qL)^{2n}(2n)!\sum_{l=0}^{n}\frac{1}{\Gamma[2l+1](2(n-l))!}\left(q_x\right)^{2l}\left(q_y\right)^{2n-2l}\frac{\Gamma\left[\frac{2l+1}{2}\right]}{\sqrt{\pi}(l!)}H_{2n-2l,2l+1}$$

With a further simplification using Eq. (S.7.4.1.4) and $x = 2l+1$ we remove all Gamma functions to finally obtain an expression that can be inserted into the expansions and asymptotes (S.7.5.1.4)

$$(\boldsymbol{qL})^{2n} = (qL)^{2n}(2n)!\sum_{l=0}^{n}\frac{1}{4^l(2(n-l))!\,(l!)^2}(q_x^2)^l\left(q_y^2\right)^{n-l}H_{2n-2l,2l}$$

Regime I. This can then be inserted into the cylinder formfactor (Eq. S.4.6.1.1.2), and simplified to **<u>(S.7.5.1.5)</u>**

$$\langle P_{\parallel}(q,L)\rangle = \sum_{n=0}^{\infty}\frac{4^n}{(n+1)}\frac{(z+1)_{2n}}{\left(\frac{3}{2}\right)_n n!}\left(-\frac{(\boldsymbol{qL})^2}{4(z+1)^2}\right)^n$$

$$= \sum_{n=0}^{\infty}\frac{4^n(2n)!}{(n+1)}\frac{(z+1)_{2n}}{\left(\frac{3}{2}\right)_n n!}\left(-\frac{q^2L^2}{4}\right)^n\sum_{l=0}^{n}\frac{(q_x^2)^l\left(q_y^2\right)^{n-l}}{4^l(2(n-l))!\,(l!)^2}H_{2n-2l,2l}$$

Cylinder, polydisperse, uni-axial, y-axis, P(q)

Mathematica code implementation:

```
del=0.01;
max=30;
```

```
ccc=Table[NIntegrate[((Cos[delta])^(2*n-2*l))*((Sin[delta])^(2*l+1))*Exp[-delta/del],
{delta, 0, Pi/2}]/NIntegrate[Exp[-delta/del]*Sin[delta], {delta, 0, Pi/2}], {n, 0, max},{l,
0, max}];

L=5;
R=1;
sigma=0.1;
z=(1-sigma*sigma)/(sigma*sigma);
phi1=0.1*Pi/2;
phi2=0.9*Pi/2;
qs=10^lqs;
qq=Sqrt[qx*qx+qy*qy];
qxs1=qs*Cos[phi1];
qys1=qs*Sin[phi1];
qxs2=qs*Cos[phi2];
qys2=qs*Sin[phi2];
(* isotropic formfactor *)
Pqiso=Sum[(4^n)*Pochhammer[z+1,2*n]*((-
qs*qs*L*L/(4*(z+1)*(z+1)))^n)/((2*n+1)*(n+1)*Pochhammer[3/2,n]*(n!)), {n, 0, 60}];
Pqisoa=Pi*(z+1)/(2*z*qs*L);
(* perfectly oriented formfactor *)
Pq1a1=Sum[(4^n)*Pochhammer[z+1,2*n]*((-
1/(4*(z+1)*(z+1)))^n)*((qys1*L)^(2*n))/((n+1)*Pochhammer[3/2,n]*(n!)), {n, 0, max}];
Pq1a2=Sum[(4^n)*Pochhammer[z+1,2*n]*((-
1/(4*(z+1)*(z+1)))^n)*((qys2*L)^(2*n))/((n+1)*Pochhammer[3/2,n]*(n!)), {n, 0, max}];
(* series expansion *)
Pq5a1=Sum[((4^n)*((2*n)!)*Pochhammer[z+1,2*n]*((-
qs*qs*L*L/(4*(z+1)*(z+1)))^n)/((n+1)*Pochhammer[3/2,n]*(n!)))*Sum[((qxs1/qs)^(2*l))*((qys1/
qs)^(2*(n-l)))*ccc[[n+1,l+1]]/((4^l)*((2*(n-l))!)*((l)!)*((l)!)), {l, 0, n}],{n, 0, max}];
Pq5a2=Sum[((4^n)*((2*n)!)*Pochhammer[z+1,2*n]*((-
qs*qs*L*L/(4*(z+1)*(z+1)))^n)/((n+1)*Pochhammer[3/2,n]*(n!)))*Sum[((qxs2/qs)^(2*l))*((qys2/
qs)^(2*(n-l)))*ccc[[n+1,l+1]]/((4^l)*((2*(n-l))!)*((l)!)*((l)!)), {l, 0, n}],{n, 0, max}];
(* numerical integration *)
Pq3=Sum[((4^n)*((2*n)!)*Pochhammer[z+1,2*n]*((-
qq*qq*L*L/(4*(z+1)*(z+1)))^n)/((n+1)*Pochhammer[3/2,n]*(n!)))*Sum[((qx/qq)^(2*l))*((qy/qq)^
(2*(n-l)))*ccc[[n+1,l+1]]/((4^l)*((2*(n-l))!)*((l)!)*((l)!)), {l, 0, n}],{n, 0, max}];
(* numerical integration *)
u1=Gamma[z-1]/(2*Gamma[z+1]);
argz1=(L*(qxs1*Sin[chi]*Sin[delta]+qys1*Cos[delta])/(z+1));
argz2=(L*(qxs2*Sin[chi]*Sin[delta]+qys2*Cos[delta])/(z+1));
Pqintz1=NIntegrate[((argz1)^(-2))*(1-Cos[(z-1)*ArcTan[2*argz1]]/((1+4*argz1*argz1)^((z-
1)/2)))*Exp[-delta/del]*Sin[delta], {chi, 0, 2*Pi},{delta, 0, Pi/2}];
Pqintz2=NIntegrate[((argz2)^(-2))*(1-Cos[(z-1)*ArcTan[2*argz2]]/((1+4*argz2*argz2)^((z-
1)/2)))*Exp[-delta/del]*Sin[delta], {chi, 0, 2*Pi},{delta, 0, Pi/2}];
Pqintn=NIntegrate[Exp[-delta/del]*Sin[delta], {delta, 0, Pi/2}];
Pqint1=(u1/(2*Pi))*Pqintz1/Pqintn;
Pqint2=(u1/(2*Pi))*Pqintz2/Pqintn;
lim=0.5;
pl1=Plot[Log[10,Pq1a1], {lqs, -1, lim}, PlotRange->{-3,1}, GridLines->{Automatic},
LabelStyle->Directive[Black,16],AxesLabel->{"log q","lg P(q)"}, AxesOrigin->{-1,-
3},TicksStyle->Directive[Black,10],PlotStyle->{Blue,Dashed}];
pl2=Plot[Log[10,Pq1a2], {lqs, -1, lim}, PlotRange->{-3,1}, GridLines->{Automatic},
LabelStyle->Directive[Black,16],AxesLabel->{"log q","lg P(q)"}, AxesOrigin->{-1,-
3},TicksStyle->Directive[Black,10],PlotStyle->{Blue,Dashed}];
pl3=Plot[Log[10,Pq5a1], {lqs, -1, lim}, PlotRange->{-3,1}, GridLines->{Automatic},
LabelStyle->Directive[Black,16],AxesLabel->{"log q","lg P(q)"}, AxesOrigin->{-1,-
3},TicksStyle->Directive[Black,10],PlotStyle->{Black}];
pl4=Plot[Log[10,Pq5a2], {lqs, -1, lim}, PlotRange->{-3,1}, GridLines->{Automatic},
LabelStyle->Directive[Black,16],AxesLabel->{"log q","lg P(q)"}, AxesOrigin->{-1,-
3},TicksStyle->Directive[Black,10],PlotStyle->{Black}];
pl5=Plot[Log[10,Pqiso], {lqs, -1, lim}, PlotRange->{-3,1}, GridLines->{Automatic},
LabelStyle->Directive[Black,16],AxesLabel->{"log q","lg P(q)"}, AxesOrigin->{-1,-
3},TicksStyle->Directive[Black,10]];
pl6=Plot[Log[10,Pqisoa], {lqs, -1, lim}, PlotRange->{-3,1}, GridLines->{Automatic},
LabelStyle->Directive[Black,16],AxesLabel->{"log q","lg P(q)"}, AxesOrigin->{-1,-
3},TicksStyle->Directive[Black,10]];
```

```
pl7=Plot[Log[10,Pqint1], {lqs, -1, lim}, PlotRange->{-3,1}, GridLines->{Automatic},
LabelStyle->Directive[Black,16],AxesLabel->{"log q","lg P(q)"}, AxesOrigin->{-1,-
3},TicksStyle->Directive[Black,10],PlotStyle->{Red,Dashed}];
pl8=Plot[Log[10,Pqint2], {lqs, -1, lim}, PlotRange->{-3,1}, GridLines->{Automatic},
LabelStyle->Directive[Black,16],AxesLabel->{"log q","lg P(q)"}, AxesOrigin->{-1,-
3},TicksStyle->Directive[Black,10],PlotStyle->{Red,Dashed}] ;
Show[pl1,pl2,pl3,pl4,pl5,pl6,pl7,pl8]
lims=3;
pl5=DensityPlot[Log[10,Pq3],{qx,-lims,lims},{qy,-lims,lims},PlotRange->{-8,0},PlotPoints-
>20,PlotLegends->Automatic,LabelStyle->Directive[Black,12],AxesLabel->Automatic]
```

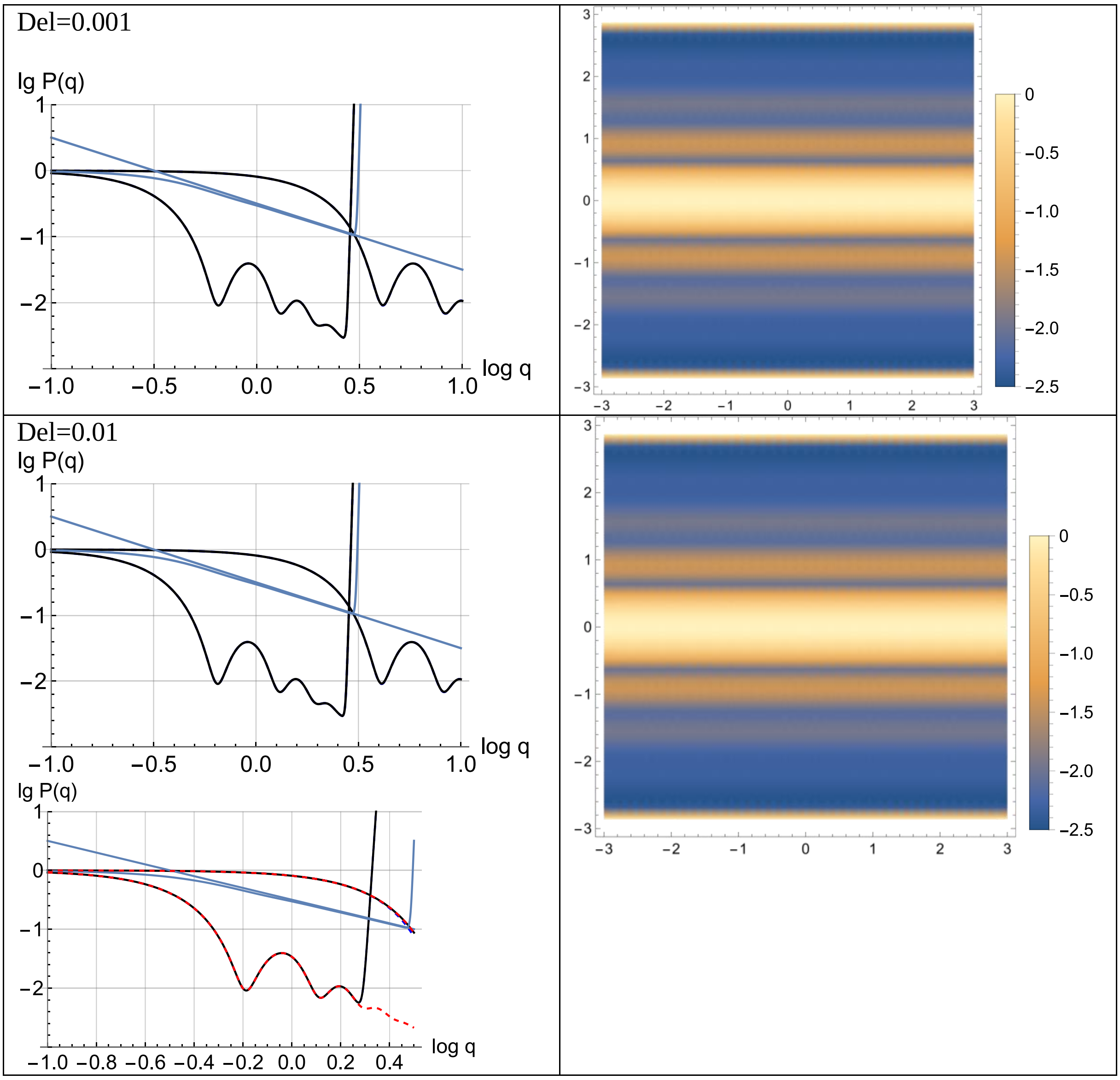

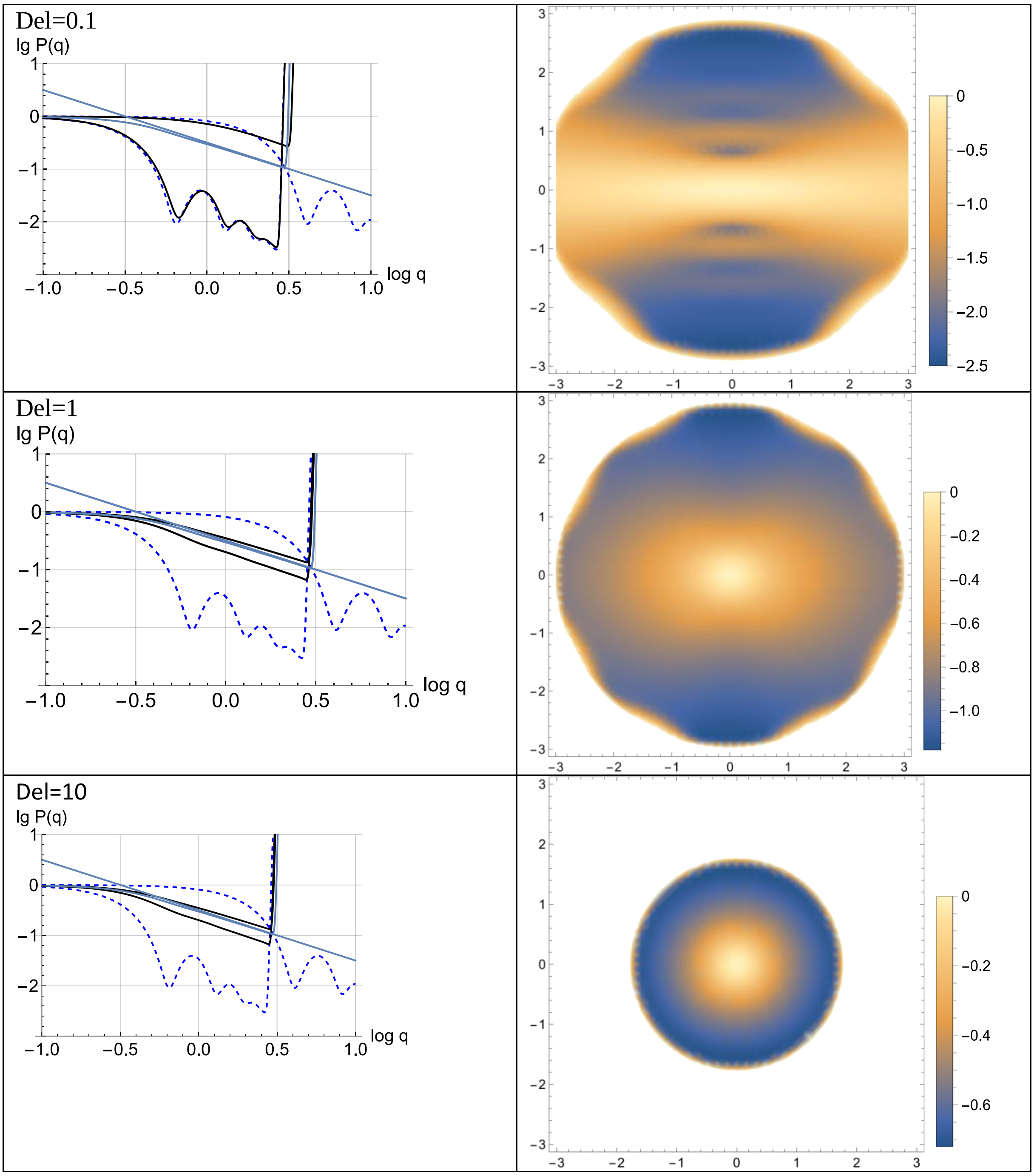

Fig. S58: Formfactor of oriented polydisperse cylinders with y-axial orientational distributions varying from perfectly oriented to the isotropic state.

Isotropic Limit

In the following we will show that the expression correctly yields the isotropic limit for the case $\bar{\delta} \rightarrow \infty$, for which $h(\delta) \rightarrow 1$. Then the integral over the deviation angle can be solved analytically to obtain

$$H_{2n-2l,2l+1} = \int_0^{\pi/2} (\cos(\delta))^{2n-2l}(\sin(\delta))^{2l+1} d\delta = \frac{\Gamma\left[\frac{2n-2l+1}{2}\right]\Gamma\left[\frac{2l+2}{2}\right]}{2\Gamma\left[\frac{2n+3}{2}\right]}$$

This can be inserted into Eq. (S.7.4.1.5) to obtain

$$\langle(\boldsymbol{qL})^{2n}\rangle = (qL)^{2n}\frac{(2n)!}{2\Gamma\left[\frac{2n+3}{2}\right]}\sum_{l=0}^{n}\frac{\Gamma\left[\frac{2n-2l+1}{2}\right]}{4^l\Gamma[2n-2l+1](\mathrm{l!})}(q_x^2)^l(q_y^2)^{n-l}$$

Using Eq. (S.7.4.1.4) with $x = 2n - 2l + 1$ and reducing the binomial it simplifies to

$$\langle(\boldsymbol{qL})^{2n}\rangle = (qL)^{2n}\frac{(2n)!\sqrt{\pi}}{24^n(\mathrm{n!})\Gamma\left[\frac{2n+3}{2}\right]}$$

Using the identity Eq. (S.7.4.1.7) we finally have

$$\langle(\boldsymbol{qL})^{2n}\rangle = (qL)^{2n}\frac{1}{(2n+1)}$$

This is the desired equation that is equal to the expression in Eq. (S.4.6.2.1.2) relating to the integral

$$\int_0^{\frac{\pi}{2}} (\cos(\phi))^{2n}\sin\phi\, d\phi = \frac{1}{2n+1}$$

Regime II. For intermediate and large q-values we use the numerical integration

$$\langle P_{\parallel}(q,L)\rangle = \frac{1}{2\pi H}\int_0^{\pi/2}\int_0^{2\pi}\langle\frac{(\sin(\boldsymbol{qL}))^2}{(\boldsymbol{qL})^2}\rangle\, h(\delta)(\sin(\delta)\, d\chi d\delta$$

$$H = \int_0^{\pi/2} h(\delta)(\sin(\delta)\, d\delta$$

where the integrand is computed in terms of the series expansion Eq. (S.1.2.40) as

$$\langle\left(\frac{\sin(\boldsymbol{qL})}{\boldsymbol{qL}}\right)^2\rangle_L = \sum_{n=0}^{\infty}\frac{4^n}{(\mathrm{n}+1)}\frac{(z_L+s_L+1)_{2n}}{\left(\frac{3}{2}\right)_n n!}\left(-\frac{\boldsymbol{q}^2\boldsymbol{L}^2}{4(z_L+1)^2}\right)^n$$

or via the asymptotic expansion Eq. (S.4.4.1.3) as

$$\langle\frac{(\sin(\boldsymbol{qL}))^2}{(\boldsymbol{qL})^2}\rangle = \frac{\Gamma[z-1]}{\Gamma[z+1]}\frac{1}{2}\left(\frac{z+1}{\boldsymbol{qL}}\right)^2\left(1+\frac{\cos\left[(z-1)\arctan\left(\frac{2\boldsymbol{qL}}{z+1}\right)\right]}{\left(1+\left(\frac{2\boldsymbol{qL}}{z+1}\right)^2\right)^{\frac{z-1}{2}}}\right)$$

with

$$\boldsymbol{qL} = qL\left[q_x \sin(\chi)\sin(\delta) + q_y \cos(\delta)\right]$$

where for the inner integral the expressions for regime I, II, and III can be used, depending on the value of the argument $\boldsymbol{qL}$.

The function (S.7.4.1.8)

$$P(\boldsymbol{qL}) = \frac{1}{1 + \frac{2\Gamma[z+1]}{\Gamma[z-1]}\left(\frac{\boldsymbol{qL}}{z+1}\right)^2}$$

can be well used as an approximation for polydisperse cases or for broad orientational distributions where the formfactor oscillations are smoothed out.

7.5.2 Disks with normal parallel to y-axis

For disks the phase term $\boldsymbol{qR} = qr\sin(\beta)$ with the enclosed angle β can be expressed in terms of the deviation and cone angles δ, χ as (S.7.5.2.1)

$$\boldsymbol{qR} = qR\sin(\beta) = qR\sin(\arccos[MI_0 q]) = qR\sqrt{1-(MI_0 q)^2}$$
$$= qR\sqrt{1-\left(\hat{q}_x \sin(\chi)\sin(\delta) + \hat{q}_y \cos(\delta)\right)^2}$$

with $\hat{q}_x = \frac{q_x}{q}$ and $\hat{q}_y = \frac{q_y}{q}$. For perfect orientation with $\delta = 0$ the expression reduces to

$$\boldsymbol{qR} = q_y R$$

For the formfactor we need to the expression $(\boldsymbol{qR})^{2n}$ (S.7.5.2.2)

$$(\boldsymbol{qR})^{2n} = (qR)^{2n}\left(1-\left(\hat{q}_x \sin(\chi)\sin(\delta) + \hat{q}_y \cos(\delta)\right)^2\right)^n$$

We use the binomial form

$$(\boldsymbol{qR})^{2n} = (qR)^{2n}\sum_{m=0}^{n}\binom{n}{m}(-1)^m\left(\hat{q}_x \sin(\chi)\sin(\delta) + \hat{q}_y \cos(\delta)\right)^{2m}$$

which can be expressed in terms of a second binomial

$$(\boldsymbol{qR})^{2n} = (qR)^{2n}\sum_{m=0}^{n}\binom{n}{m}(-1)^m\sum_{k=0}^{2m}\binom{2m}{k}\left(\hat{q}_x\right)^k\left(\hat{q}_y\right)^{2m-k}\left[(\sin(\chi)\sin(\delta))^k(\cos(\delta))^{2m-k}\right]$$

The angular part […] has to be integrated over the cone angle χ and the deviation angle δ

$(\boldsymbol{qR})^{2n}$

$$= (qR)^{2n} \sum_{m=0}^{n} \binom{n}{m} (-1)^m \sum_{\boldsymbol{k=0}}^{\boldsymbol{2m}} \binom{2m}{k} \left(q_x\right)^k \left(q_y\right)^{2m-k} \left(\frac{1}{2\pi} \int_{\boldsymbol{0}}^{2\pi} (\cos(\chi))^0 (\sin(\chi))^k d\chi\right) \left(\int_{\boldsymbol{0}}^{\frac{\pi}{2}} (\cos(\delta))^{2m-k} (\sin(\delta))^{k+1} d\delta\right)$$

The integral of the cone angle χ can be solved analytically

$$\frac{1}{2\pi} \int_0^{2\pi} (\sin(\chi))^k d\chi = \frac{\left(1 + (-1)^k\right) \Gamma\left[\frac{1}{2}\right] \Gamma\left[\frac{k+1}{2}\right]}{2\pi \Gamma\left[\frac{k+2}{2}\right]}$$

The integral of the deviation angle δ is given by (S.7.5.2.3)

$$\frac{\int_0^{\pi/2} (\cos(\delta))^{2m-k} (\sin(\delta))^k h(\delta) \sin(\delta)\, d\delta}{\int_0^{\pi/2} h(\delta) \sin(\delta)\, d\delta} = H_{2m-k,k+1}$$

We now have

$(\boldsymbol{qR})^{2n}$

$$= (qR)^{2n} (n)! \sum_{m=0}^{n} \frac{(2m)!\,(-1)^m}{m!\,(n-m)!} \sum_{k=0}^{2m} \frac{1}{k!\,(2m-k)!} (q_x)^k \left(q_y\right)^{2m-k} \left(\frac{\left(1 + (-1)^k\right) \Gamma\left[\frac{1}{2}\right] \Gamma\left[\frac{k+1}{2}\right]}{2\pi \Gamma\left[\frac{k+2}{2}\right]}\right) H_{2m-k,k+1}$$

We observe that there are contributions to the sum only if *m* is even, i.e. $m = 2l$. We therefore change the summation index from *m* to *l* and simplify to obtain

$$(\boldsymbol{qR})^{2n} = (qR)^{2n} (n)! \sum_{m=0}^{n} \frac{(2m)!\,(-1)^m}{m!\,(n-m)!} \sum_{\boldsymbol{l=0}}^{\boldsymbol{m}} \frac{\left(q_x\right)^{2l} \left(q_y\right)^{2m-2l}}{\Gamma[2l+1](2m-2l)!} \frac{\Gamma\left[\frac{2l+1}{2}\right]}{\sqrt{\pi}\Gamma\left[\frac{2l+2}{2}\right]} H_{2m-2l,2l+1}$$

We use Eq. (S.7.4.1.4) with $x = 2l + 1$ to remove all Gamma functions we finally obtain an expression that can be inserted into the expansions and asymptotes.

$$(\boldsymbol{qR})^{2n} = (qR)^{2n} (n)! \sum_{m=0}^{n} \frac{(2m)!\,(-1)^m}{m!\,(n-m)!} \sum_{\boldsymbol{l=0}}^{\boldsymbol{m}} \frac{(q_x^2)^l \left(q_y^2\right)^{m-l}}{4^l (2m-2l)!\,(l!)^2} H_{2m-2l,2l+1}$$

Regime I. This can then be inserted into the disk formfactor (Eq. 4.6.1.1.2), and simplified to

$$P(q,R) = \sum_{n=0}^{\infty} \frac{4^{n+1} \Gamma\left(n + \frac{3}{2}\right)}{\sqrt{\pi}\Gamma(n+3)} \frac{(z+1)_{2n}}{(2)_n n!} (-1)^n \langle \left(\frac{\boldsymbol{qR}}{2}\right)^{2n} \rangle$$

with **<u>(S.7.5.2.4)</u>**

$$P(q,R) = \sum_{n=0}^{\infty} \frac{4^{n+1} \Gamma\left(n + \frac{3}{2}\right)}{\sqrt{\pi}\Gamma(n+3)} \frac{(z+1)_{2n}}{(2)_n} \left(-\frac{q^2 R^2}{4}\right)^n \sum_{m=0}^{n} \frac{(2m)!\,(-1)^m}{m!\,(n-m)!} \sum_{\boldsymbol{l=0}}^{\boldsymbol{m}} \frac{\left(q_x^2\right)^l \left(q_y^2\right)^{m-l}}{4^l (2m-2l)!\,(l!)^2} H_{2m-2l,2l+1}$$

Disks, polydisperse, uni-axial distribution, y-axis, P(q)

Mathematica code implementation:

```
del=10;
max=30;
ccc=Table[NIntegrate[((Cos[delta])^(2*n-2*l))*((Sin[delta])^(2*l+1))*Exp[-delta/del],
{delta, 0, Pi/2}]/NIntegrate[Exp[-delta/del]*Sin[delta], {delta, 0, Pi/2}], {n, 0, max},{l,
0, max}];

L=1;
R=5;
sigma=0.1;
z=Sqrt[1-sigma*sigma]/(sigma*sigma);
phi1=0.1*Pi/2;
phi2=0.9*Pi/2;
qs=10^lqs;
qq=Sqrt[qx*qx+qy*qy];
qxs1=qs*Cos[phi1];
qys1=qs*Sin[phi1];
qxs2=qs*Cos[phi2];
qys2=qs*Sin[phi2];
(* isotropic formfactor *)
Pqiso=Sum[2*(4^n)*Pochhammer[z+1,2*n]*((-
qs*qs*R*R/(4*(z+1)*(z+1)))^n)/(Pochhammer[2,n]*((n+2)!)), {n, 0, 60}];
Pqisoa=2*Gamma[z-1]*((z+1)^2)/(Gamma[z+1]*qs*qs*R*R);
(* perfectly oriented formfactor *)
Pq1a1=Sum[(4^(n+1))*Gamma[n+3/2]*Pochhammer[z+1,2*n]*((-
1/(4*(z+1)*(z+1)))^n)*((qys1*R)^(2*n))/(Sqrt[Pi]*((n+2)!)*Pochhammer[2,n]*(n!)), {n, 0,
max}];
Pq1a2=Sum[(4^(n+1))*Gamma[n+3/2]*Pochhammer[z+1,2*n]*((-
1/(4*(z+1)*(z+1)))^n)*((qys2*R)^(2*n))/(Sqrt[Pi]*((n+2)!)*Pochhammer[2,n]*(n!)), {n, 0,
max}];
(* series expansion *)
Pq5a1=Sum[((4^(n+1))*Gamma[n+3/2]*Pochhammer[z+1,2*n]*((-
qs*qs*R*R/(4*(z+1)*(z+1)))^n)/(Sqrt[Pi]*((n+2)!)*Pochhammer[2,n]))*Sum[(((2*m)!)*((-
1)^m)/((m!)*((n-m)!)))*Sum[((qxs1/qs)^(2*l))*((qys1/qs)^(2*(m-
l)))*ccc[[m+1,l+1]]/((4^l)*(l!)*(l!)*((2*(m-l))!)), {l, 0, m}],{m, 0, n}],{n, 0, max}];
Pq5a2=Sum[((4^(n+1))*Gamma[n+3/2]*Pochhammer[z+1,2*n]*((-
qs*qs*R*R/(4*(z+1)*(z+1)))^n)/(Sqrt[Pi]*((n+2)!)*Pochhammer[2,n]))*Sum[(((2*m)!)*((-
1)^m)/((m!)*((n-m)!)))*Sum[((qxs2/qs)^(2*l))*((qys2/qs)^(2*(m-
l)))*ccc[[m+1,l+1]]/((4^l)*(l!)*(l!)*((2*(m-l))!)), {l, 0, m}],{m, 0, n}],{n, 0, max}];
(* 2D *)
Pq3=Sum[((4^(n+1))*Gamma[n+3/2]*Pochhammer[z+1,2*n]*((-
qq*qq*R*R/(4*(z+1)*(z+1)))^n)/(Sqrt[Pi]*((n+2)!)*Pochhammer[2,n]))*Sum[(((2*m)!)*((-
1)^m)/((m!)*((n-m)!)))*Sum[((qx/qq)^(2*l))*((qy/qq)^(2*(m-
l)))*ccc[[m+1,l+1]]/((4^l)*(l!)*(l!)*((2*(m-l))!)), {l, 0, m}],{m, 0, n}],{n, 0, max}];
(* numerical integration *)
u1=Gamma[z-1]/(2*Gamma[z+1]);
(* argz1=(L*(qxs1*Cos[delta]-qys1*Sin[chi]*Sin[delta])/(z+1));
argz2=(L*(qxs2*Cos[delta]-qys2*Sin[chi]*Sin[delta])/(z+1));
Pqintz1=NIntegrate[((argz1)^(-2))*(1-Cos[(z-1)*ArcTan[2*argz1]]/((1+4*argz1*argz1)^((z-
1)/2)))*Exp[-delta/del]*Sin[delta], {chi, 0, 2*Pi},{delta, 0, Pi/2}];
Pqintz2=NIntegrate[((argz2)^(-2))*(1-Cos[(z-1)*ArcTan[2*argz2]]/((1+4*argz2*argz2)^((z-
1)/2)))*Exp[-delta/del]*Sin[delta], {chi, 0, 2*Pi},{delta, 0, Pi/2}];
Pqintn=NIntegrate[Exp[-delta/del]*Sin[delta], {delta, 0, Pi/2}];
Pqint1=(u1/(2*Pi))*Pqintz1/Pqintn;
Pqint2=(u1/(2*Pi))*Pqintz2/Pqintn; *)
lim=1;
pl1=Plot[Log[10,Pq1a1], {lqs, -1, lim}, PlotRange->{-3,1}, GridLines->{Automatic},
LabelStyle->Directive[Black,16],AxesLabel->{"log q","lg P(q)"}, AxesOrigin->{-1,-
3},TicksStyle->Directive[Black,10],PlotStyle->{Blue,Dashed}];
```

```
pl2=Plot[Log[10,Pq1a2], {lqs, -1, lim}, PlotRange->{-3,1}, GridLines->{Automatic},
LabelStyle->Directive[Black,16],AxesLabel->{"log q","lg P(q)"}, AxesOrigin->{-1,-
3},TicksStyle->Directive[Black,10],PlotStyle->{Blue,Dashed}];
pl3=Plot[Log[10,Pq5a1], {lqs, -1, lim}, PlotRange->{-3,1}, GridLines->{Automatic},
LabelStyle->Directive[Black,16],AxesLabel->{"log q","lg P(q)"}, AxesOrigin->{-1,-
3},TicksStyle->Directive[Black,10],PlotStyle->{Black}];
pl4=Plot[Log[10,Pq5a2], {lqs, -1, lim}, PlotRange->{-3,1}, GridLines->{Automatic},
LabelStyle->Directive[Black,16],AxesLabel->{"log q","lg P(q)"}, AxesOrigin->{-1,-
3},TicksStyle->Directive[Black,10],PlotStyle->{Black}];
pl5=Plot[Log[10,Pqiso], {lqs, -1, lim}, PlotRange->{-3,1}, GridLines->{Automatic},
LabelStyle->Directive[Black,16],AxesLabel->{"log q","lg P(q)"}, AxesOrigin->{-1,-
3},TicksStyle->Directive[Black,10]];
pl6=Plot[Log[10,Pqisoa], {lqs, -1, lim}, PlotRange->{-3,1}, GridLines->{Automatic},
LabelStyle->Directive[Black,16],AxesLabel->{"log q","lg P(q)"}, AxesOrigin->{-1,-
3},TicksStyle->Directive[Black,10]];
(* pl7=Plot[Log[10,Pqint1], {lqs, -1, lim}, PlotRange⍰{-3,1}, GridLines->{Automatic},
LabelStyle->Directive[Black,16],AxesLabel->{"log q","lg P(q)"}, AxesOrigin->{-1,-
3},TicksStyle->Directive[Black,10],PlotStyle->{Red,Dashed}]
pl8=Plot[Log[10,Pqint2], {lqs, -1, lim}, PlotRange⍰{-3,1}, GridLines->{Automatic},
LabelStyle->Directive[Black,16],AxesLabel->{"log q","lg P(q)"}, AxesOrigin->{-1,-
3},TicksStyle->Directive[Black,10],PlotStyle->{Red,Dashed}] *)
Show[pl1,pl2,pl3,pl4,pl5,pl6]
lims=3;
pl5=DensityPlot[Log[10,Pq3],{qx,-lims,lims},{qy,-lims,lims},PlotRange->{-8,0},PlotPoints-
>50,PlotLegends->Automatic,LabelStyle->Directive[Black,12],AxesLabel->Automatic]
```

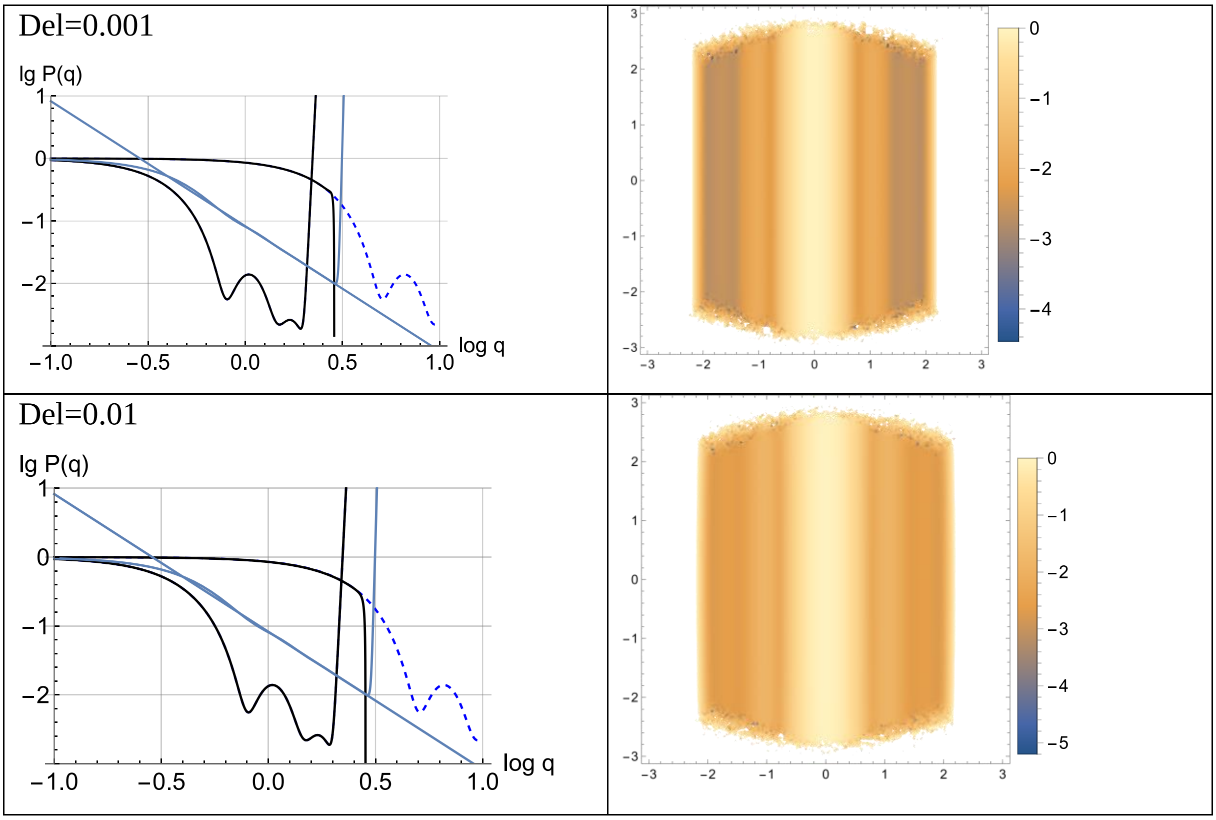

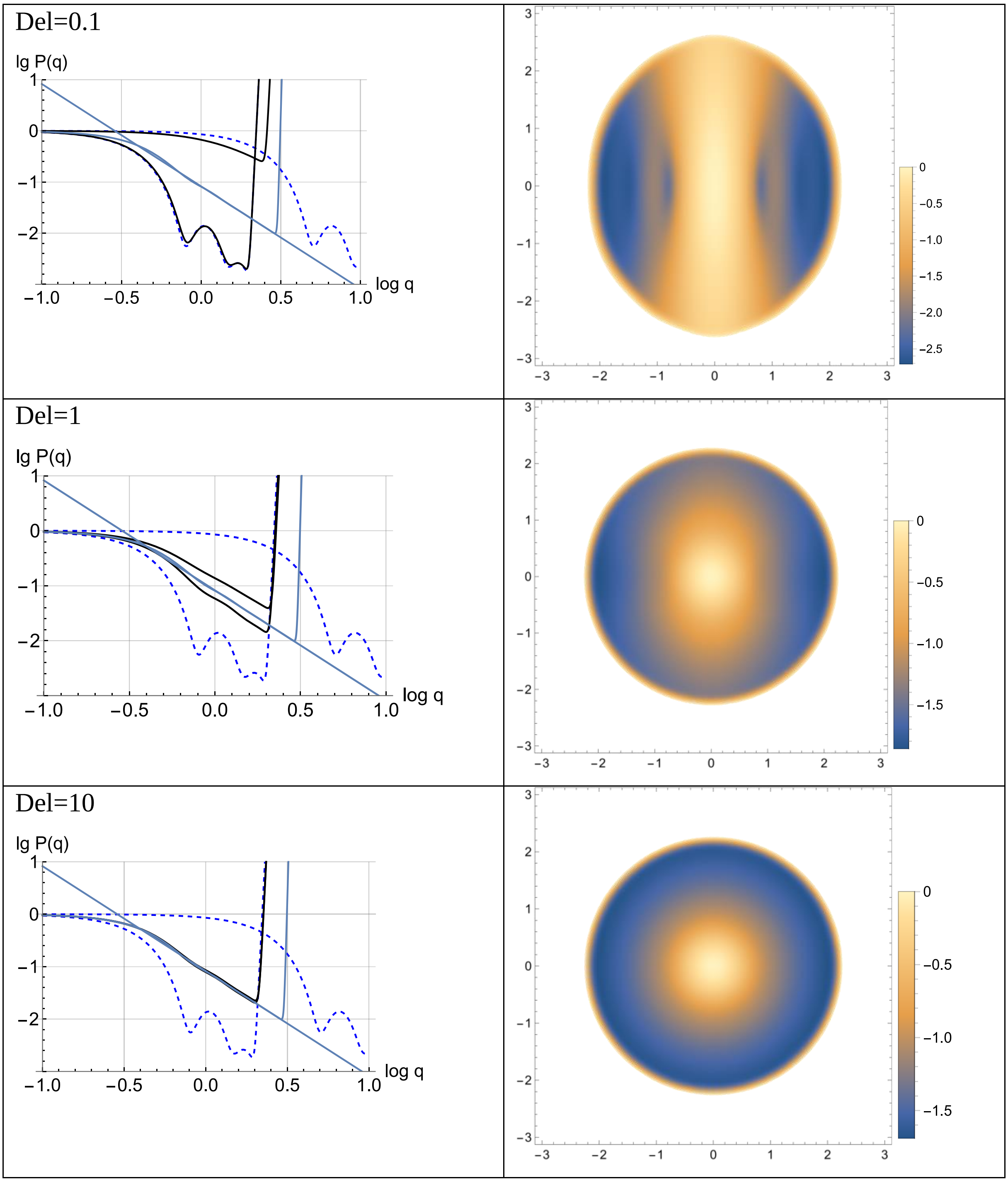

Fig. S59: Formfactor of oriented polydisperse disks with y-axial orientational distributions varying from perfectly oriented to the isotropic state.

For rapid calculations the q-independent coefficients are precalculated, such that the series can be quickly calculated for each pixel (q_x, q_y)

$$\langle P_{\parallel}(q_x, q_y, L)\rangle = \sum_{n=0}^{\infty} a_n \sum_{l=0}^{n} b_{n,m} \sum_{l=0}^{m} c_{m,l} (q_x^2)^l (q_y^2)^{m-l}$$

Isotropic Limit

In the following we will show that the expression correctly yields the isotropic limit for the case $\bar{\delta} \to \infty$, for which $h(\delta) \to 1$. Then the integral over the deviation angle can be solved analytically to obtain

$$H_{2m-2l,2l+1} = \int_0^{\pi/2} (\cos(\delta))^{2m-2l} (\sin(\delta))^{2l+1} d\delta = \frac{\Gamma\left[\frac{2m-2l+1}{2}\right]\Gamma\left[\frac{2l+2}{2}\right]}{2\Gamma\left[\frac{2m+3}{2}\right]}$$

This can be inserted into Eq. (S.7.5.2.4) using $m = 2l$ to obtain

$$(\boldsymbol{qR})^{2n} = (qR)^{2n}(n)! \sum_{m=0}^{n} \frac{(2m)!\,(-1)^m}{m!\,(n-m)!\,2\Gamma\left[\frac{2m+3}{2}\right]} \sum_{\boldsymbol{l=0}}^{\boldsymbol{m}} \frac{\Gamma\left[\frac{2m-2l+1}{2}\right](q_x^2)^l (q_y^2)^{m-l}}{4^l \Gamma[2m-2l+1](l!)}$$

using Eq. (S.7.4.1.4) with $x = 2m - 2l + 1$ yields the simplified expression

$$(\boldsymbol{qR})^{2n} = (qR)^{2n}(n)! \sum_{m=0}^{n} \frac{\sqrt{\pi}(2m)!\,(-1)^m}{m!\,4^m (n-m)!\,2\Gamma\left[\frac{2m+3}{2}\right] m!} \sum_{\boldsymbol{l=0}}^{\boldsymbol{m}} \frac{m!\,(q_x^2)^l (q_y^2)^{m-l}}{(\mathrm{m}-\mathrm{l})!\,(l!)}$$

to re-express the binomial to obtain

$$(\boldsymbol{qR})^{2n} = (qr)^{2n}(n)! \sum_{m=0}^{n} \frac{(2m)!\sqrt{\pi}}{2m!\,4^m \Gamma\left[\frac{2m+3}{2}\right]} \frac{(-1)^m}{(n-m)!\,m!}$$

It can be further reduced with Eq. (S.7.4.1.7) to result in

$$(\boldsymbol{qR})^{2n} = (qR)^{2n} \frac{(n)!}{2} \sum_{m=0}^{n} \frac{1}{(m+\frac{1}{2})} \frac{(-1)^m}{(n-m)!\,m!}$$

Using the identitiy Eq. (S.4.2.5) we finally have

$$(\boldsymbol{qR})^{2n} = (qR)^{2n} \frac{(n)!\sqrt{\pi}}{2\Gamma\left[n+\frac{3}{2}\right]}$$

This is the desired equation that is equal to the expression in Eq. (S.4.7.1.2) relating to the integral

$$\int_0^{\frac{\pi}{2}} (\sin(\phi))^{2n} \sin\phi\, d\phi = \frac{\sqrt{\pi}\Gamma(n+1)}{2\Gamma\left(n+\frac{3}{2}\right)} = \frac{\sqrt{\pi}(n)!}{2\Gamma\left(n+\frac{3}{2}\right)}$$

Regime II. For intermediate and large q-values we use the numerical integration

$$P^{(2)}(q_r R) = \langle P_{\perp}(q_r R)\rangle = \frac{1}{2\pi H}\int_0^{\pi/2}\int_0^{2\pi} \langle\left(\frac{2J_1(\boldsymbol{qR})}{\boldsymbol{qR}}\right)^2\rangle\, h(\delta)(\sin(\delta)\, d\chi d\delta$$

$$H = \int_0^{\pi/2} h(\delta)(\sin(\delta)\, d\delta$$

where the integrand is computed in terms of the series expansion Eq. (S.1.2.53) as

$$\langle\left(\frac{2J_1(\boldsymbol{qR})}{\boldsymbol{qR}}\right)^2\rangle = \sum_{n=0}^{\infty} \frac{4^{n+1}\Gamma\left(n+\frac{3}{2}\right)}{\sqrt{\pi}\Gamma(n+3)} \frac{(z_R + s_R + 1)_{2n}}{(2)_n n!}\left(-\frac{\boldsymbol{q}^2\boldsymbol{R}^2}{4(z_R+1)^2}\right)^n$$

or via the asymptotic expansion Eq. (S.1.2.54) as

$$\langle\left(\frac{2J_1(\boldsymbol{qR})}{\boldsymbol{qR}}\right)^2\rangle = \frac{4}{\pi}\left(\left(\frac{1}{\boldsymbol{qR}}\right)^3 - \left(\frac{1}{\boldsymbol{qR}}\right)^3 \sin(2\boldsymbol{qR}) - \frac{9}{8}\left(\frac{1}{\boldsymbol{qR}}\right)^4 \cos(2\boldsymbol{qR}) + \left(\frac{9}{16}\right)^2\left(\frac{1}{\boldsymbol{qR}}\right)^5 + \left(\frac{9}{16}\right)^2\left(\frac{1}{\boldsymbol{qR}}\right)^5 \sin(2\boldsymbol{qR})\right)$$

with

$$\boldsymbol{qR} = qR\sqrt{1-\left(\hat{q}_x \sin(\chi)\sin(\delta) + \hat{q}_y\cos(\delta)\right)^2}$$

Where for the integrand the expressions for regime I, II, and III can be used, depending on the value of the argument $\boldsymbol{qR}$**.**

The function

$$P(\boldsymbol{qR}) = \frac{1}{1 + \frac{\pi\Gamma[z+1]}{4\Gamma[z-2]}\left(\frac{\boldsymbol{qR}}{z+1}\right)^3}$$

can be well uased as an approximation for polydisperse cases or broad orientational distributins where the formfactor oscillations are smoothed out.

7.6 Alignment parallel to z-axis

7.6.1 Cylinders parallel to z-axis

The case that $\boldsymbol{D} \parallel z$ corresponds to the angles $\varphi = \pi/2$, $\theta = 0$. The director $\boldsymbol{D}$, axis-vector $\boldsymbol{L}_\delta$, the matrix $\boldsymbol{M}$, the vector $\boldsymbol{L}(\delta,\chi)$, and the phase $\boldsymbol{qL}(\delta,\chi)$ are then given by

$$\boldsymbol{D} = \begin{pmatrix} 0 \\ 0 \\ -1 \end{pmatrix} \quad \boldsymbol{L}_\delta = L\begin{pmatrix} 0 \\ -\sin(\delta) \\ -\cos(\delta) \end{pmatrix} \quad \boldsymbol{M} = \begin{pmatrix} c\chi & -s\chi & 0 \\ s\chi & c\chi & 0 \\ 0 & 0 & 2c\chi - 1 \end{pmatrix}$$

$$\boldsymbol{L}(\delta,\chi) = L\begin{pmatrix} \sin(\chi)\sin(\delta) \\ -\cos(\chi)\sin(\delta) \\ -\cos(\delta) \end{pmatrix}$$

$$\boldsymbol{qL}(\delta,\chi) = \boldsymbol{qML}_\delta = q_x\sin(\chi)\sin(\delta) - q_y\cos(\chi)\sin(\delta) - q_z\cos(\delta)$$

In the small-angle approximation we have $q_z \approx 0$ such that

$$\boldsymbol{qL}(\delta,\chi) \approx q_x\sin(\chi)\sin(\delta) - q_y\cos(\chi)\sin(\delta)$$

For perfect orientation with $\delta = 0$ the expression reduces to $\boldsymbol{qL}(\delta,\chi) = 0$.

7.6.1 Cylinders

For cylinders the phase term is given by $\boldsymbol{qL} = qL\cos(\beta)$ which can be expressed in terms of the deviation and cone angles δ,χ as (S.7.6.1.1)

$$\boldsymbol{qL} = qL\cos(\beta) = qL\cos(\arccos[MI_0q]) = qL\big[q_x\sin(\chi)\sin(\delta) - q_y\cos(\chi)\sin(\delta)\big]$$

For the formfactor we need to the expression $(\boldsymbol{qL})^{2n}$ (S.7.6.1.2)

$$(\boldsymbol{qL})^{2n} = (qL)^{2n}\left(q_x\sin(\chi)\sin(\delta) - q_y\cos(\chi)\sin(\delta)\right)^{2n}$$

We use the binomial form

$$(\boldsymbol{qL})^{2n} = (qL)^{2n}\sum_{m=0}^{2n}\binom{2n}{m}\left(q_x\right)^m\left(-q_y\right)^{2n-m}\left[(\cos(\chi))^{2n-m}(\sin(\chi))^m(\sin(\delta))^{2n}\right]$$

The angular part [...] has to be integrated over the cone angle χ and the deviation angle δ

$$(\boldsymbol{qL})^{2n} = (qL)^{2n}\sum_{m=0}^{2n}\binom{2n}{m}(q_x)^m(-q_y)^{2n-m}\left(\frac{1}{2\pi}\int_{\boldsymbol{0}}^{2\pi}(\cos(\chi))^{2n-m}(\sin(\chi))^m\boldsymbol{d}\chi\right)\left(\int_{\boldsymbol{0}}^{\frac{\pi}{2}}(\sin(\delta))^{2n}\sin(\delta)\,d\delta\right)$$

The integral of the cone angle χ can be solved analytically

$$\frac{1}{2\pi}\int_0^{2\pi}(\cos(\chi))^{2n-m}(\sin(\chi))^m d\chi = \frac{(1+(-1)^{2n-m})(1+(-1)^{2n})\Gamma\left[\frac{2n-m+1}{2}\right]\Gamma\left[\frac{m+1}{2}\right]}{4\pi\Gamma\left[\frac{2n+2}{2}\right]}$$

The integral of the deviation angle δ is given by (S.7.6.1.2)

$$\frac{\int_0^{\pi/2}(\sin(\delta))^{2n}h(\delta)\sin(\delta)\,d\delta}{\int_0^{\pi/2}h(\delta)\sin(\delta)\,d\delta} = H_{0,2n+1}$$

We now have

$$(\boldsymbol{qL})^{2n} = (qL)^{2n} \sum_{m=0}^{2n} \frac{(2n)!}{m!\,(2n-m)!} (q_x)^m (-q_y)^{2n-m} \frac{(1+(-1)^{2n-m})\Gamma\left[\frac{2n-m+1}{2}\right]\Gamma\left[\frac{m+1}{2}\right]}{2\pi\Gamma\left[\frac{2n+2}{2}\right]} H_{0,2n+1}$$

We observe that there are contributions to the sum only if *m* is even, i.e. $m = 2l$. We therefore change the summation index from *m* to *l* and simplify to obtain

$$(\boldsymbol{qL})^{2n} = (qL)^{2n} \sum_{l=0}^{n} \frac{(2n)!}{\Gamma[2l+1]\Gamma[2n-2l+1]} (q_x)^{2l} (q_y)^{2n-2l} \frac{\Gamma\left[\frac{2n-2l+1}{2}\right]\Gamma\left[\frac{2l+1}{2}\right]}{\pi\Gamma\left[\frac{2n+2}{2}\right]} H_{0,2n+1}$$

With a further simplification using Eq. (S.7.4.1.4) with $x = 2l+1$ and $x' = 2n-2l+1$ to remove all Gamma functions we finally obtain an expression that can be inserted into the expansions and asymptotes

$$(\boldsymbol{qL})^{2n} = (qL)^{2n} \frac{(2n)!}{4^n(\mathrm{n!})(\mathrm{n!})} H_{0,2n} \sum_{l=0}^{n} \binom{n}{l} (q_x^2)^l (q_y^2)^{n-l}$$

The binomial can be simplified to obtain

$$(\boldsymbol{qL})^{2n} = (qL)^{2n} \frac{(2n)!}{4^n(\mathrm{n!})(\mathrm{n!})} H_{0,2n}$$

Regime I. This can then be inserted into the cylinder formfactor Eq. (S.4.6.1.1.2), and simplified to **(S.7.6.1.3)**

$$\langle P_{\parallel}(q,L)\rangle = \sum_{n=0}^{\infty} \frac{4^n}{(\mathrm{n}+1)} \frac{(z+1)_{2n}}{\left(\frac{3}{2}\right)_n n!} \left(-\frac{(\boldsymbol{qL})^2}{4(z+1)^2}\right)^n$$

$$= \sum_{n=0}^{\infty} \frac{(2n)!\,(z+1)_{2n}}{\left(\frac{3}{2}\right)_n (\mathrm{n}+1)!\,\mathrm{n}!\,n!} \left(-\frac{q^2L^2}{4}\right)^n H_{0,2n}$$

Cylinder, polydisperse, uni-axial distribution, z-axis, P(q)

Mathematica code implementation:

```
del=10;
max=20;
ccc=Table[NIntegrate[((Cos[delta])^(0))*((Sin[delta])^(2*l+1))*Exp[-delta/del], {delta, 0, Pi/2}]/NIntegrate[Exp[-delta/del]*Sin[delta], {delta, 0, Pi/2}], {n, 0, max},{l, 0, max}];

L=5;
R=1;
sigma=0.1;
z=Sqrt[1-sigma*sigma]/(sigma*sigma);
phi1=0.1*Pi/2;
phi2=0.9*Pi/2;
qs=10^lqs;
qq=Sqrt[qx*qx+qy*qy];
qxs1=qs*Cos[phi1];
```

```
qys1=qs*Sin[phi1];
qxs2=qs*Cos[phi2];
qys2=qs*Sin[phi2];
(* isotropic formfactor *)
Pqiso=Sum[(4^n)*Pochhammer[z+1,2*n]*((-
qs*qs*L*L/(4*(z+1)*(z+1)))^n)/((2*n+1)*(n+1)*Pochhammer[3/2,n]*(n!)), {n, 0, 60}];
Pqisoa=Pi*(z+1)/(2*z*qs*L);
(* perfectly oriented formfactor *)
Pq1a1=Sum[(4^n)*Pochhammer[z+1,2*n]*((-
1/(4*(z+1)*(z+1)))^n)*((qys1*L)^(2*n))/((n+1)*Pochhammer[3/2,n]*(n!)), {n, 0, max}];
Pq1a2=Sum[(4^n)*Pochhammer[z+1,2*n]*((-
1/(4*(z+1)*(z+1)))^n)*((qys2*L)^(2*n))/((n+1)*Pochhammer[3/2,n]*(n!)), {n, 0, max}];
(* series expansion *)
Pq5a1=Sum[(((2*n)!)*Pochhammer[z+1,2*n]*((-
qs*qs*L*L/(4*(z+1)*(z+1)))^n)*ccc[[1,n+1]]/(Pochhammer[3/2,n]*((n+1)!)*(n!)*(n!))),{n, 0,
max}];
Pq5a2=Sum[(((2*n)!)*Pochhammer[z+1,2*n]*((-
qs*qs*L*L/(4*(z+1)*(z+1)))^n)*ccc[[1,n+1]]/(Pochhammer[3/2,n]*((n+1)!)*(n!)*(n!))),{n, 0,
max}];
(* numerical integration *)
Pq3=Sum[(((2*n)!)*Pochhammer[z+1,2*n]*((-
qq*qq*L*L/(4*(z+1)*(z+1)))^n)*ccc[[1,n+1]]/(Pochhammer[3/2,n]*((n+1)!)*(n!)*(n!))),{n, 0,
max}];
(* numerical integration
u1=Gamma[z-1]/(2*Gamma[z+1]);
argz1=(L*(qxs1*Sin[chi]*Sin[delta]+qys1*Cos[delta])/(z+1));
argz2=(L*(qxs2*Sin[chi]*Sin[delta]+qys2*Cos[delta])/(z+1));
Pqintz1=NIntegrate[((argz1)^(-2))*(1-Cos[(z-1)*ArcTan[2*argz1]]/((1+4*argz1*argz1)^((z-
1)/2)))*Exp[-delta/del]*Sin[delta], {chi, 0, 2*Pi},{delta, 0, Pi/2}];
Pqintz2=NIntegrate[((argz2)^(-2))*(1-Cos[(z-1)*ArcTan[2*argz2]]/((1+4*argz2*argz2)^((z-
1)/2)))*Exp[-delta/del]*Sin[delta], {chi, 0, 2*Pi},{delta, 0, Pi/2}];
Pqintn=NIntegrate[Exp[-delta/del]*Sin[delta], {delta, 0, Pi/2}];
Pqint1=(u1/(2*Pi))*Pqintz1/Pqintn;
Pqint2=(u1/(2*Pi))*Pqintz2/Pqintn; *)
lim=1;
pl1=Plot[Log[10,Pq1a1], {lqs, -1, lim}, PlotRange->{-3,1}, GridLines->{Automatic},
LabelStyle->Directive[Black,16],AxesLabel->{"log q","lg P(q)"}, AxesOrigin->{-1,-
3},TicksStyle->Directive[Black,10],PlotStyle->{Blue,Dashed}];
pl2=Plot[Log[10,Pq1a2], {lqs, -1, lim}, PlotRange->{-3,1}, GridLines->{Automatic},
LabelStyle->Directive[Black,16],AxesLabel->{"log q","lg P(q)"}, AxesOrigin->{-1,-
3},TicksStyle->Directive[Black,10],PlotStyle->{Blue,Dashed}];
pl3=Plot[Log[10,Pq5a1], {lqs, -1, lim}, PlotRange->{-3,1}, GridLines->{Automatic},
LabelStyle->Directive[Black,16],AxesLabel->{"log q","lg P(q)"}, AxesOrigin->{-1,-
3},TicksStyle->Directive[Black,10],PlotStyle->{Black}];
pl4=Plot[Log[10,Pq5a2], {lqs, -1, lim}, PlotRange->{-3,1}, GridLines->{Automatic},
LabelStyle->Directive[Black,16],AxesLabel->{"log q","lg P(q)"}, AxesOrigin->{-1,-
3},TicksStyle->Directive[Black,10],PlotStyle->{Black}];
pl5=Plot[Log[10,Pqiso], {lqs, -1, lim}, PlotRange->{-3,1}, GridLines->{Automatic},
LabelStyle->Directive[Black,16],AxesLabel->{"log q","lg P(q)"}, AxesOrigin->{-1,-
3},TicksStyle->Directive[Black,10]];
pl6=Plot[Log[10,Pqisoa], {lqs, -1, lim}, PlotRange->{-3,1}, GridLines->{Automatic},
LabelStyle->Directive[Black,16],AxesLabel->{"log q","lg P(q)"}, AxesOrigin->{-1,-
3},TicksStyle->Directive[Black,10]];
(* pl7=Plot[Log[10,Pqint1], {lqs, -1, lim}, PlotRange{-3,1}, GridLines->{Automatic},
LabelStyle->Directive[Black,16],AxesLabel->{"log q","lg P(q)"}, AxesOrigin->{-1,-
3},TicksStyle->Directive[Black,10],PlotStyle->{Red,Dashed}]
pl8=Plot[Log[10,Pqint2], {lqs, -1, lim}, PlotRange{-3,1}, GridLines->{Automatic},
LabelStyle->Directive[Black,16],AxesLabel->{"log q","lg P(q)"}, AxesOrigin->{-1,-
3},TicksStyle->Directive[Black,10],PlotStyle->{Red,Dashed}] *)
Show[pl1,pl2,pl3,pl4,pl5,pl6]
lims=3;
pl5=DensityPlot[Log[10,Pq3],{qx,-lims,lims},{qy,-lims,lims},PlotRange->{-8,0},PlotPoints-
>20,PlotLegends->Automatic,LabelStyle->Directive[Black,12],AxesLabel->Automatic]
```

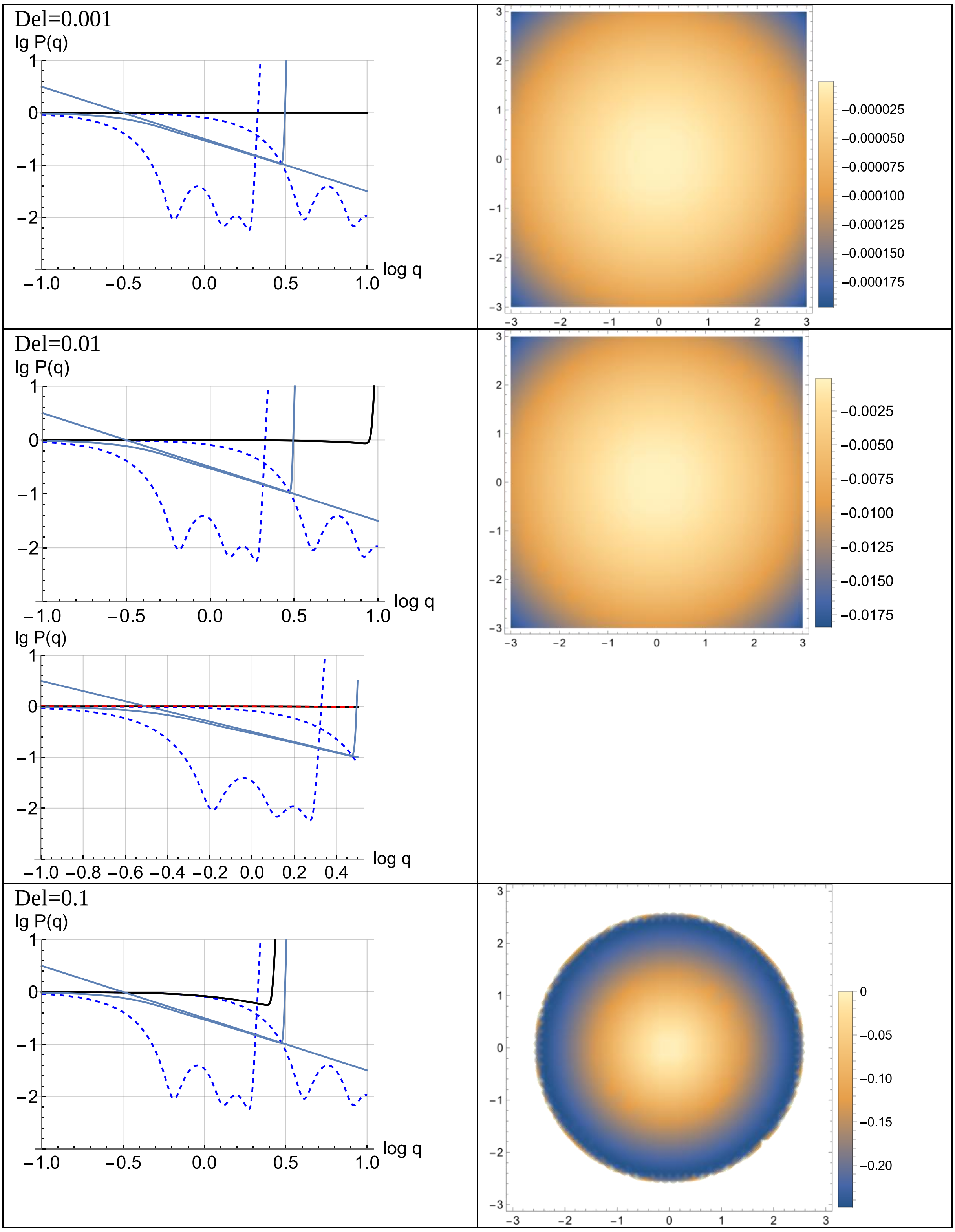
Del=0.001
lg P(q)
log q
−0.000025
−0.000050
−0.000075
−0.000100
−0.000125
−0.000150
−0.000175
Del=0.01
lg P(q)
log q
−0.0025
−0.0050
−0.0075
−0.0100
−0.0125
−0.0150
−0.0175
lg P(q)
log q
Del=0.1
lg P(q)
log q
0
−0.05
−0.10
−0.15
−0.20

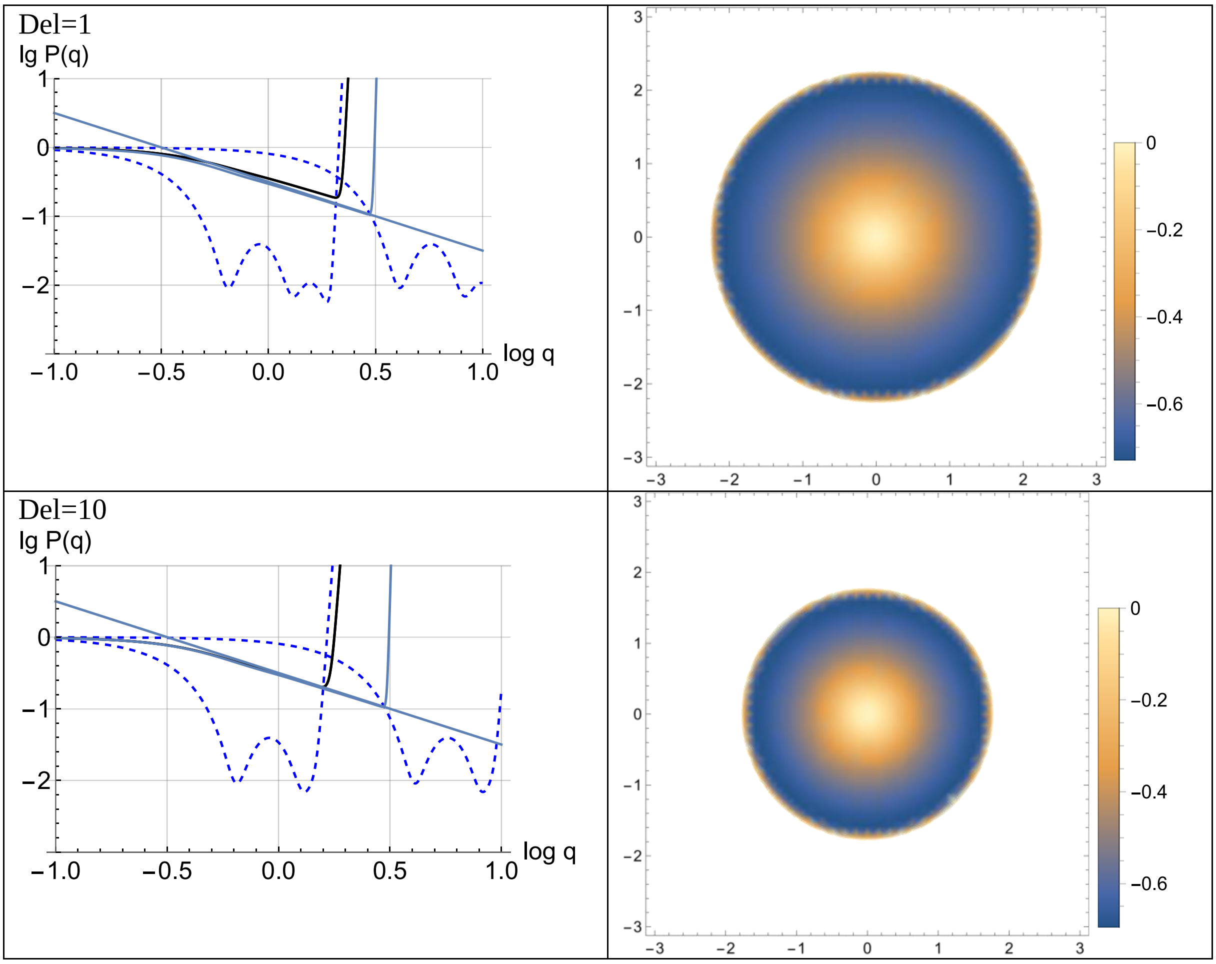

Fig. S60: Formfactor of oriented polydisperse cylinders with z-axial orientational distributions varying from perfectly oriented to the isotropic state.

For rapid calculations the q-independent coefficients are precalculated, such that the series can be quickly calculated for each pixel (q_x, q_y)

$$\langle P_{\parallel}(q_x, q_y, L)\rangle = \sum_{n=0}^{\infty} a_n q^{2n}$$

Isotropic Limit

In the following we will show that the expression correctly yields the isotropic limit for the case $\bar{\delta} \to \infty$, for which $h(\delta) \to 1$. Then the integral over the deviation angle can be solved analytically to obtain

$$\int_0^{\pi/2} (\cos(\delta))^0 (\sin(\delta))^{2n+1} d\delta = \frac{\Gamma\left[\frac{1}{2}\right]\Gamma\left[\frac{2n+2}{2}\right]}{2\Gamma\left[\frac{2n+3}{2}\right]}$$

This can be inserted into Eq. (S.7.6.1.3) using $m = 2l$ to obtain

$$\langle(\boldsymbol{qL})^{2n}\rangle = (qL)^{2n}\frac{(2n)!}{4^n(\mathrm{n!})}\frac{\sqrt{\pi}}{2\Gamma\left[\frac{2n+3}{2}\right]}$$

With the identity in Eq. (S.7.4.1.7) we obtain finally

$$\langle(\boldsymbol{qL})^{2n}\rangle = (qL)^{2n}\frac{1}{(2n+1)}$$

This is the desired equation that is equal to the expression in Eq. (S.4.6.2.1.2) relating to the integral

$$\int_0^{\frac{\pi}{2}} (\cos(\phi))^{2n}\sin\phi\, d\phi = \frac{1}{2n+1}$$

Regime II. For intermediate and large q-values we use the numerical integration

$$\langle P_{\parallel}(q,L)\rangle = \frac{1}{2\pi H}\int_0^{\pi/2}\int_0^{2\pi}\langle\frac{(\sin(\boldsymbol{qL}))^2}{(\boldsymbol{qL})^2}\rangle h(\delta)(\sin(\delta)\, d\chi d\delta$$

$$H = \int_0^{\pi/2} h(\delta)(\sin(\delta)\, d\delta$$

where the integrand is computed in terms of the series expansion Eq. (S.1.2.40) as

$$\langle\left(\frac{\sin(\boldsymbol{qL})}{\boldsymbol{qL}}\right)^2\rangle_L = \sum_{n=0}^{\infty}\frac{4^n}{(\mathrm{n}+1)}\frac{(z_L+s_L+1)_{2n}}{\left(\frac{3}{2}\right)_n n!}\left(-\frac{q^2\boldsymbol{L}^2}{4(z_L+1)^2}\right)^n$$

or via the asymptotic expansion Eq. (S.4.4.1.3) as

$$\langle\frac{(\sin(\boldsymbol{qL}))^2}{(\boldsymbol{qL})^2}\rangle = \frac{\Gamma[z-1]}{\Gamma[z+1]}\frac{1}{2}\left(\frac{z+1}{\boldsymbol{qL}}\right)^2\left(1+\frac{\cos\left[(z-1)\arctan\left(\frac{2\boldsymbol{qL}}{z+1}\right)\right]}{\left(1+\left(\frac{2\boldsymbol{qL}}{z+1}\right)^2\right)^{\frac{z-1}{2}}}\right)$$

with

$$\boldsymbol{qL} = qL\left[q_x\sin(\chi)\sin(\delta) - q_y\cos(\chi)\sin(\delta)\right]$$

where for the inner integral the expressions for regime I, II, and III can be used, depending on the value of the argument $\boldsymbol{qL}$.

The function

$$P(\boldsymbol{qL}) = \frac{1}{1+\frac{2\Gamma[z+1]}{\Gamma[z-1]}\left(\frac{\boldsymbol{qL}}{z+1}\right)^2}$$

can be well used as an approximation for polydisperse cases or for broad orientational distributions where the formfactor oscillations are smoothed out.

7.6.2 Disks with normal parallel to z-axis

For disks the phase factor is $\boldsymbol{qR} = qR\sin(\beta)$ which can be expressed in terms of the deviation and cone angles δ, χ as (S.7.6.2.1)

$$\boldsymbol{qR} = qR\sin(\beta) = qR\sin(\arccos[MI_0 q]) = qR\sqrt{1-(MI_0 q)^2}$$
$$= qR\sqrt{1-\left(q_x\sin(\chi)\sin(\delta) - q_y\cos(\chi)\sin(\delta)\right)^2}$$

with $\hat{q}_x = \frac{q_x}{q}$ and $\hat{q}_y = \frac{q_y}{q}$. For perfect orientation with $\delta = 0$ the expression reduces to

$$\boldsymbol{qR} = qR$$

For the formfactor we need to the expression $(\boldsymbol{qR})^{2n}$ (S.7.6.2.2)

$$(\boldsymbol{qR})^{2n} = (qR)^{2n}\left(1-\left(q_x\sin(\chi)\sin(\delta) - q_y\cos(\chi)\sin(\delta)\right)^2\right)^n$$

We use the binomial form

$$(\boldsymbol{qR})^{2n} = (qR)^{2n}\sum_{m=0}^{n}\binom{n}{m}(-1)^m\left(q_x\sin(\chi)\sin(\delta) - q_y\cos(\chi)\sin(\delta)\right)^{2m}$$

which can be expressed in terms of a second binomial

$$(\boldsymbol{qR})^{2n}$$
$$= (qR)^{2n}\sum_{m=0}^{n}\binom{n}{m}(-1)^m\sum_{\boldsymbol{k=0}}^{\boldsymbol{2m}}\binom{2m}{k}(\hat{q}_x)^k\left(-\hat{q}_y\right)^{2m-k}\left[(\cos(\chi))^{2m-k}(\sin(\chi))^k(\sin(\delta))^{2m}\right]$$

The angular part [...] has to be integrated over the cone angle χ and the deviation angle δ

The integral of the deviation angle δ is given by (S.7.6.2.3)

$$\frac{\int_0^{\pi/2}(\sin(\delta))^{2m}h(\delta)\sin(\delta)\,d\delta}{\int_0^{\pi/2}h(\delta)\sin(\delta)\,d\delta} = H_{0,2m+1}$$

We now have

$$(\boldsymbol{qR})^{2n} = (qR)^{2n}\sum_{m=0}^{n}\binom{n}{m}(-1)^m\sum_{\boldsymbol{k=0}}^{\boldsymbol{2m}}\binom{2m}{k}(q_x)^k\left(-q_y\right)^{2m-k}\left\{\frac{\left(1+(-1)^{2m-k}\right)\Gamma\left[\frac{2m-k+1}{2}\right]\Gamma\left[\frac{k+1}{2}\right]}{2\pi\Gamma\left[\frac{2m+2}{2}\right]}\right\}H_{0,2m+1}$$

We observe that there are contributions to the sum only if *m* is even, i.e. $m = 2l$. We therefore change the summation index from *m* to *l* and simplify to obtain

$$(\boldsymbol{qR})^{2n} = (qR)^{2n}(n)!\sum_{m=0}^{n}\frac{(2m)!\,(-1)^m}{m!\,(n-m)!}\sum_{\boldsymbol{l=0}}^{\boldsymbol{m}}\frac{(q_x)^{2l}\left(-q_y\right)^{2m-2l}}{\Gamma[2l+1]\Gamma[2m-2l+1]}\left\{\frac{\Gamma\left[\frac{2m-2l+1}{2}\right]\Gamma\left[\frac{2l+1}{2}\right]}{\pi\Gamma\left[\frac{2m+2}{2}\right]}\right\}H_{0,2m+1}$$

We use Eq. (S.7.4.1.4) with $x = 2m - 2l + 1$ and $x' = 2l + 1$ to remove all Gamma functions we finally obtain an expression that can be inserted into the expansions and asymptotes.

$$(\boldsymbol{qR})^{2n} = (qR)^{2n}(n)!\sum_{m=0}^{n}\frac{(2m)!\,(-1)^m}{4^m m!\,\mathrm{m}!\,\mathrm{m}!\,(n-m)!}H_{0,2m+1}\sum_{l=0}^{m}\frac{\mathrm{m}!\,(q_x^2)^l\left(q_y^2\right)^{m-l}}{(\mathrm{m}-\mathrm{l})!\,\mathrm{l}!}$$

We can simplify one binomial to obtain

$$(\boldsymbol{qR})^{2n} = (qR)^{2n}(n)!\sum_{m=0}^{n}\frac{(2m)!\,(-1)^m}{4^m m!\,\mathrm{m}!\,\mathrm{m}!\,(n-m)!}H_{0,2m+1}$$

Regime I. This can then be inserted into the cylinder formfactor (Eq. S.7.6.1.3), and simplified to **(S.7.6.2.4)**

$$P(q,R) = \sum_{n=0}^{\infty}\frac{4^{n+1}\Gamma\left(n+\frac{3}{2}\right)}{\sqrt{\pi}\Gamma(n+3)}\frac{(z+1)_{2n}}{(2)_n n!}(-1)^n\left\langle\left(\frac{\boldsymbol{qR}}{2}\right)^{2n}\right\rangle$$

$$= \sum_{n=0}^{\infty}\frac{4^{n+1}\Gamma\left(n+\frac{3}{2}\right)}{\sqrt{\pi}\Gamma(n+3)}\frac{(z+1)_{2n}}{(2)_n}\left(-\frac{q^2R^2}{4(z+1)^2}\right)^n\sum_{m=0}^{n}\frac{(2m)!\,(-1)^m}{4^m m!\,\mathrm{m}!\,\mathrm{m}!\,(n-m)!}H_{0,2m+1}$$

Disk, polydisperse, uni-axial distribution, z-axis, P(q)

Mathematica code implementation:

```
del=1;
max=40;
ccc=Table[NIntegrate[((Cos[delta])^(0))*((Sin[delta])^(2*l+1))*Exp[-delta/del], {delta, 0, Pi/2}]/NIntegrate[Exp[-delta/del]*Sin[delta], {delta, 0, Pi/2}], {n, 0, max},{l, 0, max}];

L=1;
R=5;
sigma=0.1;
z=Sqrt[1-sigma*sigma]/(sigma*sigma);
phi1=0.1*Pi/2;
phi2=0.9*Pi/2;
qs=10^lqs;
qq=Sqrt[qx*qx+qy*qy];
qxs1=qs*Cos[phi1];
qys1=qs*Sin[phi1];
qxs2=qs*Cos[phi2];
qys2=qs*Sin[phi2];
(* isotropic formfactor *)
Pqiso=Sum[2*(4^n)*Pochhammer[z+1,2*n]*((-
qs*qs*R*R/(4*(z+1)*(z+1)))^n)/(Pochhammer[2,n]*((n+2)!)), {n, 0, 60}];
Pqisoa=2*Gamma[z-1]*((z+1)^2)/(Gamma[z+1]*qs*qs*R*R);
(* perfectly oriented formfactor *)
```

```
Pq1a1=Sum[(4^(n+1))*Gamma[n+3/2]*Pochhammer[z+1,2*n]*((-
1/(4*(z+1)*(z+1)))^n)*((qys1*R)^(2*n))/(Sqrt[Pi]*((n+2)!)*Pochhammer[2,n]*(n!)), {n, 0,
max}];
Pq1a2=Sum[(4^(n+1))*Gamma[n+3/2]*Pochhammer[z+1,2*n]*((-
1/(4*(z+1)*(z+1)))^n)*((qys2*R)^(2*n))/(Sqrt[Pi]*((n+2)!)*Pochhammer[2,n]*(n!)), {n, 0,
max}];
(* series expansion *)
Pq5a1=Sum[((4^(n+1))*Gamma[n+3/2]*Pochhammer[z+1,2*n]*((-
qs*qs*R*R/(4*(z+1)*(z+1)))^n)/(Sqrt[Pi]*((n+2)!)*Pochhammer[2,n]))*Sum[(((2*m)!)*((-
1)^m)*ccc[[1,m+1]]/((4^m)*(m!)*(m!)*(m!)*((n-m)!))),{m, 0, n}],{n, 0, max}];
Pq5a2=Sum[((4^(n+1))*Gamma[n+3/2]*Pochhammer[z+1,2*n]*((-
qs*qs*R*R/(4*(z+1)*(z+1)))^n)/(Sqrt[Pi]*((n+2)!)*Pochhammer[2,n]))*Sum[(((2*m)!)*((-
1)^m)*ccc[[1,m+1]]/((4^m)*(m!)*(m!)*(m!)*((n-m)!))),{m, 0, n}],{n, 0, max}];
(* 2D *)
Pq3=Sum[((4^(n+1))*Gamma[n+3/2]*Pochhammer[z+1,2*n]*((-
qq*qq*R*R/(4*(z+1)*(z+1)))^n)/(Sqrt[Pi]*((n+2)!)*Pochhammer[2,n]))*Sum[(((2*m)!)*((-
1)^m)*ccc[[1,m+1]]/((4^m)*(m!)*(m!)*(m!)*((n-m)!))),{m, 0, n}],{n, 0, max}];
(* numerical integration *)
u1=Gamma[z-1]/(2*Gamma[z+1]);
(* argz1=(L*(qxs1*Cos[delta]-qys1*Sin[chi]*Sin[delta])/(z+1));
argz2=(L*(qxs2*Cos[delta]-qys2*Sin[chi]*Sin[delta])/(z+1));
Pqintz1=NIntegrate[((argz1)^(-2))*(1-Cos[(z-1)*ArcTan[2*argz1]]/((1+4*argz1*argz1)^((z-
1)/2)))*Exp[-delta/del]*Sin[delta], {chi, 0, 2*Pi},{delta, 0, Pi/2}];
Pqintz2=NIntegrate[((argz2)^(-2))*(1-Cos[(z-1)*ArcTan[2*argz2]]/((1+4*argz2*argz2)^((z-
1)/2)))*Exp[-delta/del]*Sin[delta], {chi, 0, 2*Pi},{delta, 0, Pi/2}];
Pqintn=NIntegrate[Exp[-delta/del]*Sin[delta], {delta, 0, Pi/2}];
Pqint1=(u1/(2*Pi))*Pqintz1/Pqintn;
Pqint2=(u1/(2*Pi))*Pqintz2/Pqintn; *)
lim=1;
pl1=Plot[Log[10,Pq1a1], {lqs, -1, lim}, PlotRange->{-3,1}, GridLines->{Automatic},
LabelStyle->Directive[Black,16],AxesLabel->{"log q","lg P(q)"}, AxesOrigin->{-1,-
3},TicksStyle->Directive[Black,10],PlotStyle->{Blue,Dashed}];
pl2=Plot[Log[10,Pq1a2], {lqs, -1, lim}, PlotRange->{-3,1}, GridLines->{Automatic},
LabelStyle->Directive[Black,16],AxesLabel->{"log q","lg P(q)"}, AxesOrigin->{-1,-
3},TicksStyle->Directive[Black,10],PlotStyle->{Blue,Dashed}];
pl3=Plot[Log[10,Pq5a1], {lqs, -1, lim}, PlotRange->{-3,1}, GridLines->{Automatic},
LabelStyle->Directive[Black,16],AxesLabel->{"log q","lg P(q)"}, AxesOrigin->{-1,-
3},TicksStyle->Directive[Black,10],PlotStyle->{Black}];
pl4=Plot[Log[10,Pq5a2], {lqs, -1, lim}, PlotRange->{-3,1}, GridLines->{Automatic},
LabelStyle->Directive[Black,16],AxesLabel->{"log q","lg P(q)"}, AxesOrigin->{-1,-
3},TicksStyle->Directive[Black,10],PlotStyle->{Black}];
pl5=Plot[Log[10,Pqiso], {lqs, -1, lim}, PlotRange->{-3,1}, GridLines->{Automatic},
LabelStyle->Directive[Black,16],AxesLabel->{"log q","lg P(q)"}, AxesOrigin->{-1,-
3},TicksStyle->Directive[Black,10]];
pl6=Plot[Log[10,Pqisoa], {lqs, -1, lim}, PlotRange->{-3,1}, GridLines->{Automatic},
LabelStyle->Directive[Black,16],AxesLabel->{"log q","lg P(q)"}, AxesOrigin->{-1,-
3},TicksStyle->Directive[Black,10]];
(* pl7=Plot[Log[10,Pqint1], {lqs, -1, lim}, PlotRange{-3,1}, GridLines->{Automatic},
LabelStyle->Directive[Black,16],AxesLabel->{"log q","lg P(q)"}, AxesOrigin->{-1,-
3},TicksStyle->Directive[Black,10],PlotStyle->{Red,Dashed}]
pl8=Plot[Log[10,Pqint2], {lqs, -1, lim}, PlotRange{-3,1}, GridLines->{Automatic},
LabelStyle->Directive[Black,16],AxesLabel->{"log q","lg P(q)"}, AxesOrigin->{-1,-
3},TicksStyle->Directive[Black,10],PlotStyle->{Red,Dashed}] *)
Show[pl1,pl2,pl3,pl4,pl5,pl6]
lims=3;
pl5=DensityPlot[Log[10,Pq3],{qx,-lims,lims},{qy,-lims,lims},PlotRange->{-8,0},PlotPoints-
>50,PlotLegends->Automatic,LabelStyle->Directive[Black,12],AxesLabel->Automatic]
```

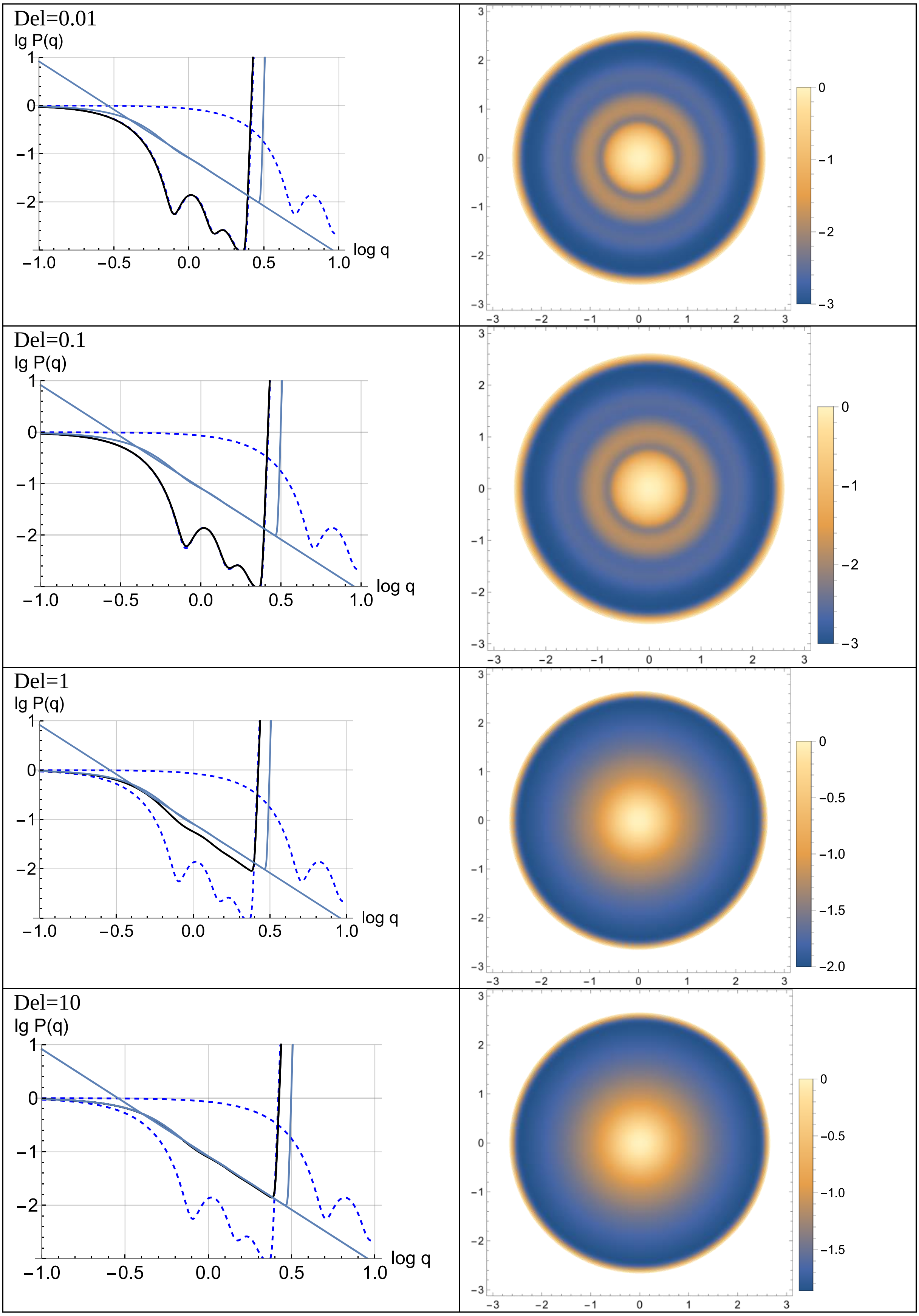

Fig. S61: Formfactor of oriented polydisperse disks with z-axial orientational distributions varying from perfectly oriented to the isotropic state.

For rapid calculations the q-independent coefficients are precalculated, such that the series can be quickly calculated for each pixel (q_x, q_y)

$$\langle P_{\parallel}(q_x, q_y, L)\rangle = \sum_{n=0}^{\infty} a_n \sum_{l=0}^{n} b_{n,m} \sum_{l=0}^{m} c_{m,l} (q_x^2)^l (q_y^2)^{m-l}$$

Isotropic Limit

In the following we will show that the expression correctly yields the isotropic limit for the case $\bar{\delta} \to \infty$, for which $h(\delta) \to 1$. Then the integral over the deviation angle can be solved analytically to obtain

$$\int_0^{\pi/2} (\sin(\delta))^{2m+1} d\delta = \frac{\Gamma\left[\frac{1}{2}\right]\Gamma\left[\frac{2m+2}{2}\right]}{2\Gamma\left[\frac{2m+3}{2}\right]}$$

This can be inserted into Eq. (S.7.6.2.4) to obtain

$$(\boldsymbol{qR})^{2n} = (qR)^{2n}(n)! \sum_{m=0}^{n} \frac{(-1)^m}{m!\,(n-m)!} \frac{(2m)!\sqrt{\pi}}{2\mathrm{m}!\,4^m\Gamma\left[m+\frac{3}{2}\right]}$$

using Eq. (S.7.4.1.7) yields the simplified expression

$$(\boldsymbol{qR})^{2n} = (qR)^{2n} \frac{(n)!}{2} \sum_{m=0}^{n} \frac{(-1)^m}{m!\,(n-m)!} \frac{1}{\left(m+\frac{1}{2}\right)}$$

and further the identity Eq. (S.7.4.2.5) we finally obtain the relation

$$(\boldsymbol{qR})^{2n} = (qR)^{2n} \frac{(n)!\sqrt{\pi}}{2\Gamma\left[n+\frac{3}{2}\right]}$$

This is the desired equation that is equal to the expression in Eq. (S.7.4.1.2) relating to the integral

$$\int_0^{\frac{\pi}{2}} (\sin(\phi))^{2n} \sin\phi\, d\phi = \frac{\sqrt{\pi}\Gamma(n+1)}{2\Gamma\left(n+\frac{3}{2}\right)} = \frac{\sqrt{\pi}(n)!}{2\Gamma\left(n+\frac{3}{2}\right)}$$

Regime II. For intermediate and large q-values we use the numerical integration

$$P^{(2)}(q_r R) = \langle P_{\perp}(q_r R)\rangle = \frac{1}{2\pi H} \int_0^{\pi/2} \int_0^{2\pi} \langle \left(\frac{2J_1(\boldsymbol{qR})}{\boldsymbol{qR}}\right)^2 \rangle\, h(\delta)(\sin(\delta)\, d\chi d\delta$$

$$H = \int_0^{\pi/2} h(\delta)(\sin(\delta)\, d\delta$$

where the integrand is computed in terms of the series expansion Eq. (S.1.2.53) as

$$\langle\left(\frac{2J_1(\boldsymbol{qR})}{\boldsymbol{qR}}\right)^2\rangle = \sum_{n=0}^{\infty} \frac{4^{n+1}\Gamma\left(n+\frac{3}{2}\right)}{\sqrt{\pi}\Gamma(n+3)} \frac{(z_R+s_R+1)_{2n}}{(2)_n n!}\left(-\frac{\boldsymbol{q}^2\boldsymbol{R}^2}{4(z_R+1)^2}\right)^n$$

or via the asymptotic expansion Eq. (S.1.2.54) as

$$\langle\left(\frac{2J_1(\boldsymbol{qR}}{\boldsymbol{qR}}\right)^2\rangle = \frac{4}{\pi}\left(\left(\frac{1}{\boldsymbol{qR}}\right)^3 - \left(\frac{1}{\boldsymbol{qR}}\right)^3 \sin(2\boldsymbol{qR}) - \frac{9}{8}\left(\frac{1}{\boldsymbol{qR}}\right)^4 \cos(2\boldsymbol{qR}) + \left(\frac{9}{16}\right)^2\left(\frac{1}{\boldsymbol{qR}}\right)^5 + \left(\frac{9}{16}\right)^2\left(\frac{1}{\boldsymbol{qR}}\right)^5 \sin(2\boldsymbol{qR})\right)$$

with

$$\boldsymbol{qR} = qR\sqrt{1-\left(q_x\sin(\chi)\sin(\delta) - q_y\cos(\chi)\sin(\delta)\right)^2}$$

Where for the integrand the expressions for regime I, II, and III can be used, depending on the value of the argument $\boldsymbol{qR}$**.**

The function

$$P(\boldsymbol{qR}) = \frac{1}{1+\frac{\pi\Gamma[z+1]}{4\Gamma[z-2]}(\frac{\boldsymbol{qR}}{z+1})^3}$$

can be well uased as an approximation for polydisperse cases or broad orientational distributins where the formfactor oscillations are smoothed out.

7.7 General alignment direction

The general case that $\boldsymbol{D} \parallel x$ corresponds to the angles φ = 0, $\theta = \pi/2$. The director $\boldsymbol{D}$, axis-vector $\boldsymbol{L}_\delta$, the matrix $\boldsymbol{M}$, the vector $\boldsymbol{L}(\delta,\chi)$, and the phase $\boldsymbol{qL}(\delta,\chi)$ are then given by

Case IV: general φ = 0, we have

$$\boldsymbol{D} = \begin{pmatrix} \sin(\theta) \\ 0 \\ -\cos(\theta) \end{pmatrix} \quad \boldsymbol{L}_\delta = L\begin{pmatrix} -\sin(\delta-\theta) \\ 0 \\ -\cos(\delta-\theta) \end{pmatrix}$$

and

$$\boldsymbol{qL}(\delta,\chi) = \boldsymbol{qML}_{\delta}$$
$$= q_x(\cos(\delta)\sin(\theta) - \cos(\chi)\cos(\theta)\sin(\delta)) - q_y\sin(\chi)\sin(\delta) - q_z(\cos(\theta)\cos(\delta) + \cos(\chi)\sin(\theta)\sin(\delta))$$

In the small-angle approximation we have $q_z \approx 0$ such that

$$\boldsymbol{qL}(\delta,\chi) \approx q_x L(\cos(\delta)\sin(\theta) - \cos(\chi)\cos(\theta)\sin(\delta)) - q_y\sin(\chi)\sin(\delta)$$

For perfect orientation with $\delta = 0$ the expression reduces to $\boldsymbol{qL}(\delta,\chi) = q_x L\sin(\theta) - q_z L\cos(\theta)$.

7.7.1 Cylinders

For cylinders the phase term $\boldsymbol{qL} = qL\cos(\beta)$ can be expressed in terms of the deviation and cone angles δ,χ as (S.7.7.1.1)

$$\boldsymbol{qL} = qL\cos(\beta) = qL\cos(\arccos[MI_0 q])$$
$$= qL\left[q_x(\cos(\delta)\sin(\theta) - \cos(\chi)\cos(\theta)\sin(\delta)) - q_y\sin(\chi)\sin(\delta)\right]$$

For the formfactor we need to the expression $(\boldsymbol{qL})^{2n}$ (S.7.7.1.2)

$$(\boldsymbol{qL})^{2n} = (qL)^{2n}\left(q_x(\cos(\delta)\sin(\theta) - \cos(\chi)\cos(\theta)\sin(\delta)) - q_y\sin(\chi)\sin(\delta)\right)^{2n}$$

We use the binomial form

$$(\boldsymbol{qL})^{2n} = (qL)^{2n}\sum_{m=0}^{2n}\binom{2n}{m}(q_x)^m\left(-q_y\right)^{2n-m}(\cos(\delta)\sin(\theta) - \cos(\chi)\cos(\theta)\sin(\delta))^m(\sin(\chi)\sin(\delta))^{2n-m}$$

and use a further binomial to obtain

$$(\boldsymbol{qL})^{2n} = (qL)^{2n}\sum_{m=0}^{2n}\binom{2n}{m}\left(q_x\right)^m\left(-q_y\right)^{2n-m}\sum_{\boldsymbol{l=0}}^{\boldsymbol{m}}\binom{m}{l}(\sin(\theta))^l(-\cos(\theta))^{m-l}[(\cos(\chi))^{m-l}(\sin(\chi))^{2n-m}(\cos(\delta))^l(\sin(\delta))^{2n-l}]$$

The angular part [...] has to be integrated over the cone angle χ and the deviation angle δ

$$(\boldsymbol{qL})^{2n} = (qL)^{2n}\sum_{m=0}^{2n}\binom{2n}{m}(q_x)^m\left(-q_y\right)^{2n-m}\sum_{\boldsymbol{l=0}}^{\boldsymbol{m}}\binom{m}{l}(\sin(\theta))^l(-\cos(\theta))^{m-l}\left(\frac{1}{2\pi}\int_{\boldsymbol{0}}^{2\pi}(\cos(\chi))^{m-l}(\sin(\chi))^{2n-m}\boldsymbol{d}\chi\right)\left(\int_{\boldsymbol{0}}^{\frac{\pi}{2}}(\cos(\delta))^l(\sin(\delta))^{2n-l}\sin(\delta)\,d\delta\right)$$

The integral of the cone angle χ can be solved analytically

$$\frac{1}{2\pi}\int_0^{2\pi}(\cos(\chi))^{m-l}(\sin(\chi))^{2n-m}d\chi = \frac{(1+(-1)^{m-l})(1+(-1)^{2n-l})\Gamma\left[\frac{m-l+1}{2}\right]\Gamma\left[\frac{2n-m+1}{2}\right]}{4\pi\Gamma\left[\frac{2n-l+2}{2}\right]}$$

The integral of the deviation angle δ is given by (S.7.7.1.3)

$$\frac{\int_0^{\pi/2}(\cos(\delta))^l(\sin(\delta))^{2n-l}h(\delta)\sin(\delta)\,d\delta}{\int_0^{\pi/2}h(\delta)\sin(\delta)\,d\delta} = H_{l,2n-l+1}$$

We now have

$$(\boldsymbol{qL})^{2n} = (qL)^{2n}\sum_{m=0}^{2n}\frac{(2n)!}{m!\,(2n-m)!}(q_x)^m(-q_y)^{2n-m}\sum_{\boldsymbol{l=0}}^{\boldsymbol{m}}\frac{(m)!}{l!\,(m-l)!}(\sin(\theta))^l(-\cos(\theta))^{m-l}\left\{\frac{(1+(-1)^{m-l})(1+(-1)^{2n-l})\Gamma\left[\frac{m-l+1}{2}\right]\Gamma\left[\frac{2n-m+1}{2}\right]}{4\pi\Gamma\left[\frac{2n-l+2}{2}\right]}\right\}H_{l,2n-l+1}$$

We observe that there are contributions to the sum only if *m* is even, i.e. $m = 2k$. We therefore change the summation index from *m* to *k* and simplify to obtain

$$= (qr)^{2n}\sum_{k=0}^{n}\frac{(2n)!}{(2k)!\,(2n-2k)!}(q_x)^{2k}(-q_y)^{2n-2k}\sum_{\boldsymbol{l=0}}^{2k}\frac{(2k)!}{l!\,(2k-l)!}(\sin(\theta))^l(-\cos(\theta))^{2k-l}\left\{\frac{(1+(-1)^{2k-l})(1+(-1)^{2n-l})\Gamma\left[\frac{2k-l+1}{2}\right]\Gamma\left[\frac{2n-2k+1}{2}\right]}{4\pi\Gamma\left[\frac{2n-l+2}{2}\right]}\right\}H_{l,2n-l+1}$$

We observe further that there are contributions to the sum only if *m* is even, i.e. $l = 2m$. We therefore change the summation index from *l* to m and rewrite to obtain

$$= (qL)^{2n}(2n)!\sum_{k=0}^{n}\frac{(q_x)^{2k}(-q_y)^{2n-2k}}{(2k)!\,(2n-2k)!}\sum_{\boldsymbol{m=0}}^{k}\frac{(2k)!}{(2m)!\,\Gamma[2k-2m+1]}(\sin(\theta))^{2m}(-\cos(\theta))^{2k-2m}\left\{\frac{\Gamma\left[\frac{2k-2m+1}{2}\right]\Gamma\left[\frac{2n-2k+1}{2}\right]}{\pi\Gamma\left[\frac{2n-2m+2}{2}\right]}\right\}H_{2m,2n-2m+1}$$

With a further simplification using Eq. (S.7.4.1.4) with $x = 2k-2m+1$ and $x = 2n-2k+1$ to remove all Gamma functions we finally obtain an expression that can be inserted into the expansions and asymptotes.

$$\langle(\boldsymbol{qL})^{2n}\rangle = (qL)^{2n}\frac{(2n)!}{4^n}\sum_{k=0}^{n}\frac{(q_x^2)^k(q_y^2)^{n-k}}{(\mathrm{n-k})!}\sum_{\boldsymbol{m=0}}^{k}\frac{4^m(\sin(\theta))^{2m}(\cos(\theta))^{2(k-m)}}{(2m)!\,(\mathrm{k-m})!\,(\mathrm{n-m})!}H_{2m,2n-2m+1}$$

Regime I. This can then be inserted into the cylinder formfactor (Eq. S.4.6.1.1.2), and simplified to **(S.7.7.1.4)**

$$\langle P_{\parallel}(q,L)\rangle = \sum_{n=0}^{\infty}\frac{4^n}{(\mathrm{n}+1)}\frac{(z+1)_{2n}}{\left(\frac{3}{2}\right)_n n!}\left(-\frac{(\boldsymbol{qL})^2}{4(z+1)^2}\right)^n$$

$$= \sum_{n=0}^{\infty}\frac{(2n)!}{(\mathrm{n}+1)}\frac{(z+1)_{2n}}{\left(\frac{3}{2}\right)_n n!}\left(-\frac{q^2L^2}{4}\right)^n\sum_{k=0}^{n}\frac{\left(q_x^2\right)^k\left(q_y^2\right)^{n-k}}{(\mathrm{n-k})!}\sum_{\boldsymbol{m=0}}^{k}\frac{4^m(\sin(\theta))^{2m}(\cos(\theta))^{2(k-m)}}{(2m)!\,(\mathrm{k-m})!\,(\mathrm{n-m})!}H_{2m,2n-2m+1}$$

Cylinder, polydisperse, uni-axial distribution, general axis, P(q)

Mathematica code implementation:

```
del=0.1;
max=40;
ccc=Table[NIntegrate[((Cos[delta])^(2*l))*((Sin[delta])^(2*n-2*l+1))*Exp[-delta/del],
{delta, 0, Pi/2}]/NIntegrate[Exp[-delta/del]*Sin[delta], {delta, 0, Pi/2}], {n, 0, max},{l,
0, max}];

L=5;
R=1;
sigma=0.1;
z=(1-sigma*sigma)/(sigma*sigma);
theta=0.2*Pi/2;
phi=0.3*Pi/2;
qxx=qx*Cos[phi]-qy*Sin[phi];
qyy=qx*Sin[phi]+qy*Cos[phi];

phi1=0.1*Pi/2;
phi2=0.9*Pi/2;
qs=10^lqs;
qq=Sqrt[qx*qx+qy*qy];
qxs1=qs*Cos[phi1];
qys1=qs*Sin[phi1];
qxs2=qs*Cos[phi2];
qys2=qs*Sin[phi2];
(* isotropic formfactor *)
Pqiso=Sum[(4^n)*Pochhammer[z+1,2*n]*((-
qs*qs*L*L/(4*(z+1)*(z+1)))^n)/((2*n+1)*(n+1)*Pochhammer[3/2,n]*(n!)), {n, 0, 60}];
Pqisoa=Pi*(z+1)/(2*z*qs*L);
(* perfectly oriented formfactor *)
Pq1a1=Sum[(4^n)*Pochhammer[z+1,2*n]*((-
1/(4*(z+1)*(z+1)))^n)*((qxs1*L)^(2*n))/((n+1)*Pochhammer[3/2,n]*(n!)), {n, 0, max}];
Pq1a2=Sum[(4^n)*Pochhammer[z+1,2*n]*((-
1/(4*(z+1)*(z+1)))^n)*((qxs2*L)^(2*n))/((n+1)*Pochhammer[3/2,n]*(n!)), {n, 0, max}];
(* series expansion *)
Pq5a1=Sum[(((2*n)!)*Pochhammer[z+1,2*n]*((-
qs*qs*L*L/(4*(z+1)*(z+1)))^n)/((n+1)*Pochhammer[3/2,n]*(n!)))*Sum[(((qxs1/qs)^(2*k))*((qys1
/qs)^(2*(n-k)))/((n-k)!))*Sum[(4^m)*((Sin[theta])^(2*m))*((Cos[theta])^(2*(k-
m)))*ccc[[n+1,m+1]]/(((2*m)!)*((k-m)!)*((n-m)!)), {m, 0,k}],{k, 0, n}],{n, 0, max}];
Pq5a2=Sum[(((2*n)!)*Pochhammer[z+1,2*n]*((-
qs*qs*L*L/(4*(z+1)*(z+1)))^n)/((n+1)*Pochhammer[3/2,n]*(n!)))*Sum[(((qxs2/qs)^(2*k))*((qys2
/qs)^(2*(n-k)))/((n-k)!))*Sum[(4^m)*((Sin[theta])^(2*m))*((Cos[theta])^(2*(k-
m)))*ccc[[n+1,m+1]]/(((2*m)!)*((k-m)!)*((n-m)!)), {m, 0,k}],{k, 0, n}],{n, 0, max}];
(* numerical integration *)
Pq3=Sum[(((2*n)!)*Pochhammer[z+1,2*n]*((-
qq*qq*L*L/(4*(z+1)*(z+1)))^n)/((n+1)*Pochhammer[3/2,n]*(n!)))*Sum[(((qxx/qq)^(2*k))*((qyy/q
q)^(2*(n-k)))/((n-k)!))*Sum[(4^m)*((Sin[theta])^(2*m))*((Cos[theta])^(2*(k-
m)))*ccc[[n+1,m+1]]/(((2*m)!)*((k-m)!)*((n-m)!)), {m, 0,k}],{k, 0, n}],{n, 0, max}];
(* numerical integration *)
u1=Gamma[z-1]/(2*Gamma[z+1]);
argz1=(L*(qxs1*Cos[delta]-qys1*Sin[chi]*Sin[delta])/(z+1));
argz2=(L*(qxs2*Cos[delta]-qys2*Sin[chi]*Sin[delta])/(z+1));
Pqintz1=NIntegrate[((argz1)^(-2))*(1-Cos[(z-1)*ArcTan[2*argz1]]/((1+4*argz1*argz1)^((z-
1)/2)))*Exp[-delta/del]*Sin[delta], {chi, 0, 2*Pi},{delta, 0, Pi/2}];
Pqintz2=NIntegrate[((argz2)^(-2))*(1-Cos[(z-1)*ArcTan[2*argz2]]/((1+4*argz2*argz2)^((z-
1)/2)))*Exp[-delta/del]*Sin[delta], {chi, 0, 2*Pi},{delta, 0, Pi/2}];
Pqintn=NIntegrate[Exp[-delta/del]*Sin[delta], {delta, 0, Pi/2}];
Pqint1=(u1/(2*Pi))*Pqintz1/Pqintn;
Pqint2=(u1/(2*Pi))*Pqintz2/Pqintn;
lim=1;
pl1=Plot[Log[10,Pq1a1], {lqs, -1, lim}, PlotRange->{-3,1}, GridLines->{Automatic},
LabelStyle->Directive[Black,16],AxesLabel->{"log q","lg P(q)"}, AxesOrigin->{-1,-
3},TicksStyle->Directive[Black,10],PlotStyle->{Blue,Dashed}];
```

```
pl2=Plot[Log[10,Pq1a2], {lqs, -1, lim}, PlotRange->{-3,1}, GridLines->{Automatic},
LabelStyle->Directive[Black,16],AxesLabel->{"log q","lg P(q)"}, AxesOrigin->{-1,-
3},TicksStyle->Directive[Black,10],PlotStyle->{Blue,Dashed}];
pl3=Plot[Log[10,Pq5a1], {lqs, -1, lim}, PlotRange->{-3,1}, GridLines->{Automatic},
LabelStyle->Directive[Black,16],AxesLabel->{"log q","lg P(q)"}, AxesOrigin->{-1,-
3},TicksStyle->Directive[Black,10],PlotStyle->{Black}];
pl4=Plot[Log[10,Pq5a2], {lqs, -1, lim}, PlotRange->{-3,1}, GridLines->{Automatic},
LabelStyle->Directive[Black,16],AxesLabel->{"log q","lg P(q)"}, AxesOrigin->{-1,-
3},TicksStyle->Directive[Black,10],PlotStyle->{Black}];
pl5=Plot[Log[10,Pqiso], {lqs, -1, lim}, PlotRange->{-3,1}, GridLines->{Automatic},
LabelStyle->Directive[Black,16],AxesLabel->{"log q","lg P(q)"}, AxesOrigin->{-1,-
3},TicksStyle->Directive[Black,10]];
pl6=Plot[Log[10,Pqisoa], {lqs, -1, lim}, PlotRange->{-3,1}, GridLines->{Automatic},
LabelStyle->Directive[Black,16],AxesLabel->{"log q","lg P(q)"}, AxesOrigin->{-1,-
3},TicksStyle->Directive[Black,10]];
(* pl7=Plot[Log[10,Pqint1], {lqs, -1, lim}, PlotRange�{-3,1}, GridLines->{Automatic},
LabelStyle->Directive[Black,16],AxesLabel->{"log q","lg P(q)"}, AxesOrigin->{-1,-
3},TicksStyle->Directive[Black,10],PlotStyle->{Red,Dashed}]
pl8=Plot[Log[10,Pqint2], {lqs, -1, lim}, PlotRange�{-3,1}, GridLines->{Automatic},
LabelStyle->Directive[Black,16],AxesLabel->{"log q","lg P(q)"}, AxesOrigin->{-1,-
3},TicksStyle->Directive[Black,10],PlotStyle->{Red,Dashed}] *)
Show[pl1,pl2,pl3,pl4,pl5,pl6]
lims=3;
pl5=DensityPlot[Log[10,Pq3],{qx,-lims,lims},{qy,-lims,lims},PlotRange->{-8,0},PlotPoints-
>50,PlotLegends->Automatic,LabelStyle->Directive[Black,12],AxesLabel->Automatic]
```

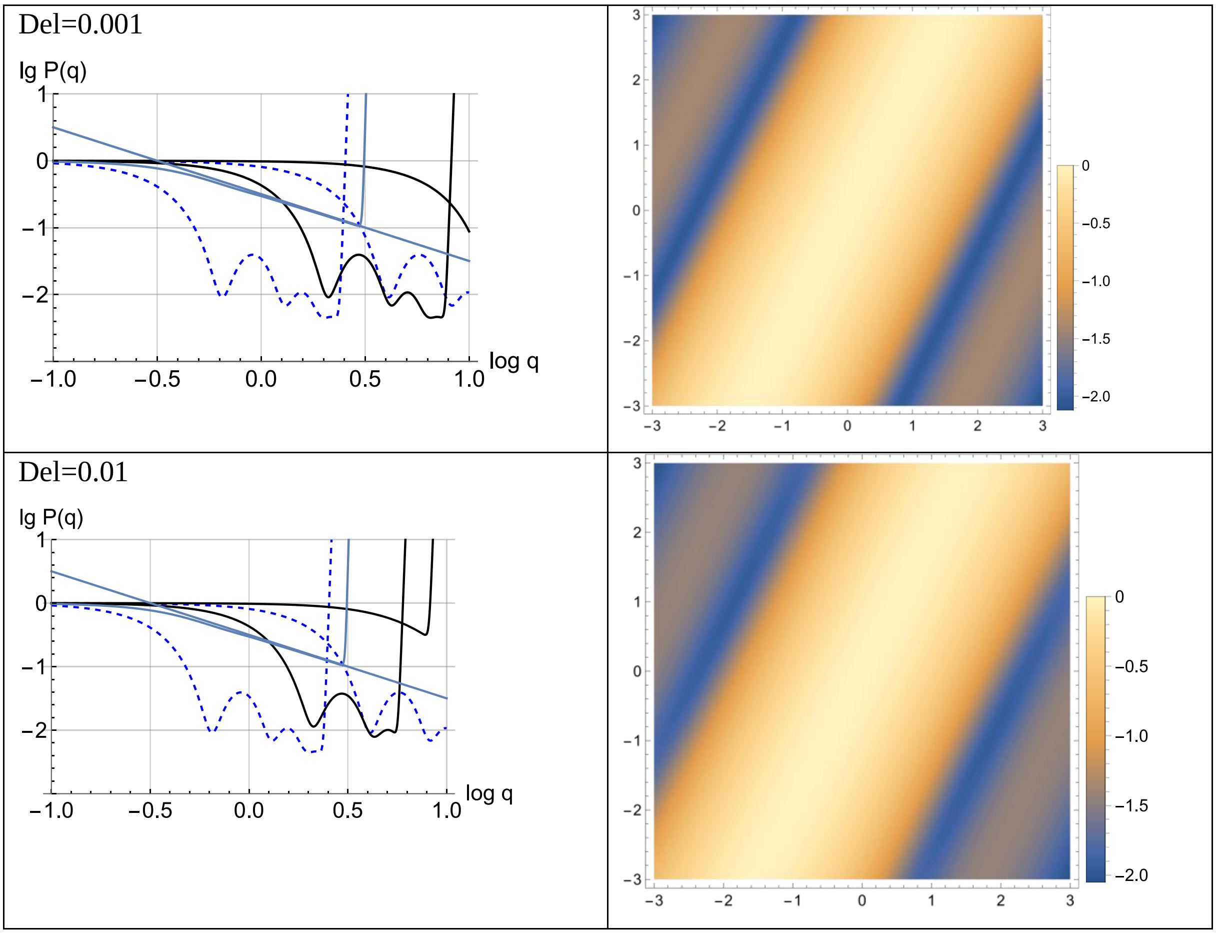

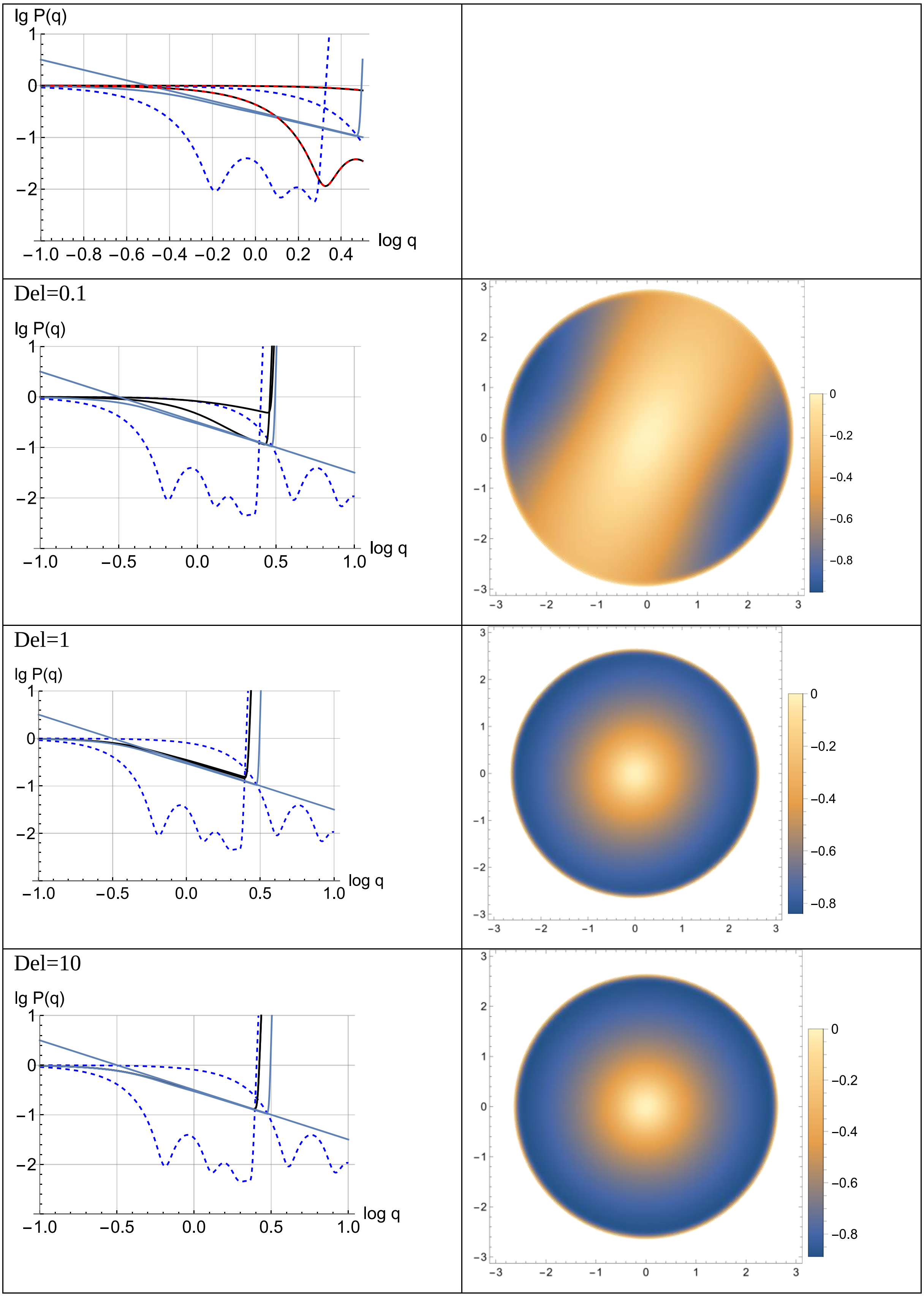

Fig. S62: Formfactor of oriented polydisperse cylinders with orientational distributions varying from perfectly oriented to the isotropic state.

For rapid calculations the q-independent coefficients are precalculated, such that the series can be quickly calculated for each pixel (q_x, q_y)

$$\langle P_{\parallel}(q_x, q_y, L)\rangle = \sum_{n=0}^{\infty} a_n q^{2n} \sum_{k=0}^{n} b_{k,n} (q_x^2)^k (q_y^2)^{n-k}$$

Isotropic Limit

In the following we will show that the expression correctly yields the isotropic limit for the case $\bar{\delta} \to \infty$, for which $h(\delta) \to 1$. Then the integral over the deviation angle can be solved analytically to obtain

$$H_{2m,2n-2m+1} = \int_0^{\pi/2} (\cos(\delta))^{2m} (\sin(\delta))^{2n-2m+1} d\delta = \frac{\Gamma\left[\frac{2m+1}{2}\right]\Gamma\left[\frac{2n-2m+2}{2}\right]}{2\Gamma\left[\frac{2n+3}{2}\right]}$$

This can be inserted into Eq. (S.7.7.1.4) to obtain

$$\langle (\boldsymbol{qL})^{2n}\rangle = (qL)^{2n} \frac{(2n)!}{4^n} \sum_{k=0}^{n} \frac{(q_x^2)^k (q_y^2)^{n-k}}{(\mathrm{n}-\mathrm{k})!} \sum_{\boldsymbol{m=0}}^{k} \frac{4^m (\sin(\theta))^{2m} (\cos(\theta))^{2(k-m)}}{\Gamma[2m+1](\mathrm{k}-\mathrm{m})!} \frac{\Gamma\left[\frac{2m+1}{2}\right]}{2\Gamma\left[\frac{2n+3}{2}\right]}$$

using Eq. (S.7.4.1.4) with $x = 2m + 1$ yields the simplified expression

$$\langle (\boldsymbol{qL})^{2n}\rangle = (qL)^{2n} \frac{(2n)!}{4^n} \frac{\sqrt{\pi}}{2\Gamma\left[\frac{2n+3}{2}\right]} \sum_{k=0}^{n} \frac{(q_x^2)^k (q_y^2)^{n-k}}{(\mathrm{n}-\mathrm{k})!} \sum_{\boldsymbol{m=0}}^{k} \frac{(\sin(\theta))^{2m} (\cos(\theta))^{2(k-m)}}{\mathrm{m}!\,(\mathrm{k}-\mathrm{m})!}$$

Simplifying the last binomial yields

$$\langle (\boldsymbol{qL})^{2n}\rangle = (qL)^{2n} \frac{(2n)!}{4^n} \frac{\sqrt{\pi}}{2\Gamma\left[\frac{2n+3}{2}\right](n!)} \sum_{k=0}^{n} \frac{(n!)(q_x^2)^k (q_y^2)^{n-k}}{(\mathrm{n}-\mathrm{k})!\,(\mathrm{k}!)}$$

Where the remaining binomial can be simplified as well to obtain

$$\langle (\boldsymbol{qL})^{2n}\rangle = (qL)^{2n} \frac{(2n)!\sqrt{\pi}}{2(n!)4^n\Gamma\left[\Gamma\left[\frac{2n+3}{2}\right]\right]}$$

With the identity Eq. (S.7.4.1.7) this reduces to

$$\langle (\boldsymbol{qL})^{2n}\rangle = (qL)^{2n} \frac{1}{(2n+1)}$$

This is the desired equation that is equal to the expression in Eq. (S.4.6.2.1.2) relating to the integral

$$\int_0^{\frac{\pi}{2}} (\cos(\phi))^{2n} \sin\phi \, d\phi = \frac{1}{2n+1}$$

Regime II. For intermediate and large q-values we use the numerical integration

$$\langle P_{\|}(q,L)\rangle = \frac{1}{2\pi H}\int_0^{\pi/2}\int_0^{2\pi} \langle\frac{(\sin(\boldsymbol{qL}))^2}{(\boldsymbol{qL})^2}\rangle\, h(\delta)(\sin(\delta)\, d\chi d\delta$$

$$H = \int_0^{\pi/2} h(\delta)(\sin(\delta)\, d\delta$$

where the integrand is computed in terms of the series expansion Eq. (S.1.2.40) as

$$\langle\left(\frac{\sin(\boldsymbol{qL})}{\boldsymbol{qL}}\right)^2\rangle_L = \sum_{n=0}^{\infty} \frac{4^n}{(\mathrm{n}+1)} \frac{(z_L + s_L + 1)_{2n}}{\left(\frac{3}{2}\right)_n n!} \left(-\frac{\boldsymbol{q}^2\boldsymbol{L}^2}{4(z_L+1)^2}\right)^n$$

or via the asymptotic expansion Eq. (S.4.4.1.3) as

$$\langle\frac{(\sin(\boldsymbol{qL}))^2}{(\boldsymbol{qL})^2}\rangle = \frac{\Gamma[z-1]}{\Gamma[z+1]}\frac{1}{2}\left(\frac{z+1}{\boldsymbol{qL}}\right)^2 \left(1 + \frac{\cos\left[(z-1)\arctan\left(\frac{2\boldsymbol{qL}}{z+1}\right)\right]}{\left(1+\left(\frac{2\boldsymbol{qL}}{z+1}\right)^2\right)^{\frac{z-1}{2}}}\right)$$

with

$$\boldsymbol{qL} = qL\left[q_x(\cos(\delta)\sin(\theta) - \cos(\chi)\cos(\theta)\sin(\delta)) - q_y\sin(\chi)\sin(\delta)\right]$$

where for the inner integral the expressions for regime I, II, and III can be used, depending on the value of the argument $\boldsymbol{qL}$.

This allows to compute the series expansion and even the double integral efficiently.

The function

$$P(\boldsymbol{qL}) = \frac{1}{1 + \frac{2\Gamma[z+1]}{\Gamma[z-1]}\left(\frac{\boldsymbol{qL}}{z+1}\right)^2}$$

can be well used as an approximation for polydisperse cases or for broad orientational distributions where the formfactor oscillations are smoothed out.

7.7.2 Disks with off-axis orientation

For disks the phase term is given by $\boldsymbol{qR} = qL\sin(\beta)$ which can be expressed in terms of the deviation and cone angles δ, χ as (S.7.7.2.1)

$$\boldsymbol{qR} = qR\sin(\beta) = qR\sin(\arccos[MI_0 q]) = qR\sqrt{1-(MI_0 q)^2}$$
$$= qr\sqrt{1-\left(q_x(\cos(\delta)\sin(\theta)-\cos(\chi)\cos(\theta)\sin(\delta))-q_y\sin(\chi)\sin(\delta)\right)^2}$$

with $\hat{q}_x = \frac{q_x}{q}$ and $\hat{q}_y = \frac{q_y}{q}$. For perfect orientation with $\delta = 0$ the expression reduces to

$$\boldsymbol{qR} = qr\sqrt{1-\left(q_x(\sin(\theta))\right)^2}$$

For the formfactor we need to the expression $(\boldsymbol{qR})^{2n}$ (S.7.7.2.2)

$$(\boldsymbol{qR})^{2n} = (qr)^{2n}\left(1-\left(q_x(\cos(\delta)\sin(\theta)-\cos(\chi)\cos(\theta)\sin(\delta))-q_y\sin(\chi)\sin(\delta)\right)^2\right)^n$$

We use the binomial form

$$(\boldsymbol{qR})^{2n} = (qR)^{2n}\sum_{m=0}^{n}\binom{n}{m}(-1)^m\left(q_x(\cos(\delta)\sin(\theta)-\cos(\chi)\cos(\theta)\sin(\delta))-q_y\sin(\chi)\sin(\delta)\right)^{2m}$$

With a further binomial

$$(\boldsymbol{qR})^{2n} = (qR)^{2n}\sum_{m=0}^{n}\binom{n}{m}(-1)^m\sum_{\boldsymbol{k=0}}^{\boldsymbol{2m}}\binom{2m}{k}(\hat{q}_x)^k(-\hat{q}_y)^{2m-k}(\cos(\delta)\sin(\theta)-\cos(\chi)\cos(\theta)\sin(\delta))^k(\sin(\chi)\sin(\delta))^{2m-k}$$

and a third binomial

$$= (qR)^{2n}\sum_{m=0}^{n}\binom{n}{m}(-1)^m\sum_{\boldsymbol{k=0}}^{\boldsymbol{2m}}\binom{2m}{k}(\hat{q}_x)^k(-\hat{q}_y)^{2m-k}\sum_{\boldsymbol{l=0}}^{\boldsymbol{k}}\binom{k}{l}(\sin(\theta))^l(-\cos(\theta))^{k-l}[(\cos(\chi))^{k-l}(\sin(\chi))^{2m-k}(\cos(\delta))^l(\sin(\delta))^{2m-l}]$$

The angular part $[\ldots]$ has to be integrated over the cone angle χ and the deviation angle δ

$$= (qR)^{2n}\sum_{m=0}^{n}\binom{n}{m}(-1)^m\sum_{\boldsymbol{k=0}}^{\boldsymbol{2m}}\binom{2m}{k}(\hat{q}_x)^k(-\hat{q}_y)^{2m-k}\sum_{\boldsymbol{l=0}}^{\boldsymbol{k}}\binom{k}{l}(\sin(\theta))^l(-\cos(\theta))^{k-l}\left(\frac{1}{2\pi}\int_0^{2\pi}(\cos(\chi))^{k-l}(\sin(\chi))^{2m-k}d\chi\right)\left(\int_0^{\frac{\pi}{2}}(\cos(\delta))^l(\sin(\delta))^{2m-l}\sin(\delta)\,d\delta\right)$$

The integral of the cone angle χ can be solved analytically

$$\frac{1}{2\pi}\int_0^{2\pi}(\cos(\chi))^{k-l}(\sin(\chi))^{2m-k}d\chi = \frac{(1+(-1)^{k-l})(1+(-1)^{2m-l})\Gamma\left[\frac{k-l+1}{2}\right]\Gamma\left[\frac{2m-k+1}{2}\right]}{4\pi\Gamma\left[\frac{2m-l+2}{2}\right]}$$

The integral of the deviation angle δ is given by (S.7.7.2.3)

$$\frac{\int_0^{\pi/2}(\cos(\delta))^l(\sin(\delta))^{2m-l}h(\delta)\sin(\delta)\,d\delta}{\int_0^{\pi/2}h(\delta)\sin(\delta)\,d\delta} = H_{l,2m-l+1}$$

We now have

$$= (qR)^{2n}(n)!\sum_{m=0}^{n}\frac{(2m)!\,(-1)^m}{m!\,(n-m)!}\sum_{\boldsymbol{k=0}}^{\boldsymbol{2m}}\frac{(\hat{q}_x)^k(-\hat{q}_y)^{2m-k}}{(2m-k)!}\sum_{\boldsymbol{l=0}}^{\boldsymbol{k}}\frac{(\sin(\theta))^l(-\cos(\theta))^{k-l}}{l!\,(k-l)!}\left\{\frac{(1+(-1)^{k-l})(1+(-1)^{2m-l})\Gamma\left[\frac{k-l+1}{2}\right]\Gamma\left[\frac{2m-k+1}{2}\right]}{4\pi\Gamma\left[\frac{2m-l+2}{2}\right]}\right\}H_{l,2m-l+1}$$

We observe that there are contributions to the sum only if *k* is even, i.e. $k = 2p$. We therefore change the summation index from *k* to *p* and simplify to obtain

$$= (qr)^{2n}(n)! \sum_{m=0}^{n} \frac{(2m)!\,(-1)^m}{m!\,(n-m)!} \sum_{\boldsymbol{p=0}}^{\boldsymbol{m}} \frac{(\hat{q}_x)^{2p}(-\hat{q}_y)^{2m-2p}}{(2m-2p)!} \sum_{\boldsymbol{l=0}}^{2p} \frac{(\sin(\theta))^l(-\cos(\theta))^{2p-l}}{l!\,(2p-l)!} \left\{ \frac{(1+(-1)^{2p-l})(1+(-1)^{2m-l})\Gamma\left[\frac{2p-l+1}{2}\right]\Gamma\left[\frac{2m-2p+1}{2}\right]}{4\pi\Gamma\left[\frac{2m-l+2}{2}\right]} \right\} H_{l,2m-l}$$

We observe that there are contributions to the sum only if *l* is even, i.e. $l = 2q$. We therefore change the summation index from *l* to *q* and simplify to obtain

$$= (qR)^{2n}(n)! \sum_{m=0}^{n} \frac{(2m)!\,(-1)^m}{m!\,(n-m)!} \sum_{\boldsymbol{p=0}}^{\boldsymbol{m}} \frac{(\hat{q}_x)^{2p}(-\hat{q}_y)^{2m-2p}}{\Gamma[2m-2p+1]} \sum_{\boldsymbol{q=0}}^{p} \frac{(\sin(\theta))^{2q}(-\cos(\theta))^{2p-2q}}{(2q)!\,\Gamma[2p-2q+1]} \left\{ \frac{\Gamma\left[\frac{2p-2q+1}{2}\right]\Gamma\left[\frac{2m-2p+1}{2}\right]}{\pi\Gamma\left[\frac{2m-2q+2}{2}\right]} \right\} H_{2q,2m-2q+1}$$

We use Eq. (S.7.4.1.4) with $x = 2m - 2p + 1$ and $x' = 2p - 2q + 1$ to remove all Gamma functions we finally obtain an expression that can be inserted into the expansions and asymptotes.

$$(\boldsymbol{qR})^{2n} = (qR)^{2n}(n)! \sum_{m=0}^{n} \frac{(2m)!\,(-1)^m}{4^m m!\,(n-m)!} \sum_{\boldsymbol{k=0}}^{\boldsymbol{m}} \frac{(q_x^2)^k (q_y^2)^{m-k}}{(\mathrm{m-k})!} \sum_{\boldsymbol{l=0}}^{k} \frac{4^l \left(\sin^2(\theta)\right)^l \left(\cos^2(\theta)\right)^{k-l}}{(2l)!\,(\mathrm{k-l})!\,(\mathrm{m-l})!} H_{2l,2m-2l+1}$$

Regime I. This can then be inserted into the disk formfactor (Eq. 4.6.1.1.2), and simplified to **<u>(S.7.7.2.4)</u>**

$$P(q,R) = \sum_{n=0}^{\infty} \frac{4^{n+1}\Gamma\left(n+\frac{3}{2}\right)}{\sqrt{\pi}\Gamma(n+3)} \frac{(z+1)_{2n}}{(2)_n n!} (-1)^n \langle \left(\frac{\boldsymbol{qR}}{2}\right)^{2n} \rangle$$

$$= \sum_{n=0}^{\infty} \frac{4^{n+1}\Gamma\left(n+\frac{3}{2}\right)}{\sqrt{\pi}\Gamma(n+3)} \frac{(z+1)_{2n}}{(2)_n} \left(-\frac{q^2R^2}{4}\right)^n \sum_{m=0}^{n} \frac{(2m)!\,(-1)^m}{4^m m!\,(n-m)!} \sum_{\boldsymbol{k=0}}^{\boldsymbol{m}} \frac{(q_x^2)^k (q_y^2)^{m-k}}{(\mathrm{m-k})!} \sum_{\boldsymbol{l=0}}^{k} \frac{4^l \left(\sin^2(\theta)\right)^l \left(\cos^2(\theta)\right)^{k-l}}{(2l)!\,(\mathrm{k-l})!\,(\mathrm{m-l})!} H_{2l,2m-2l+1}$$

Disks, polydisperse, uni-axial distribution, general axis, P(q)

Mathematica code implementation:

```
del=0.01;
max=25;
ccc=Table[NIntegrate[((Cos[delta])^(2*l))*((Sin[delta])^(2*n-2*l+1))*Exp[-delta/del],
{delta, 0, Pi/2}]/NIntegrate[Exp[-delta/del]*Sin[delta], {delta, 0, Pi/2}], {n, 0, max},{l,
0, max}];

L=1;
R=5;
sigma=0.1;
z=Sqrt[1-sigma*sigma]/(sigma*sigma);
theta=0.8*Pi/2;
phi=0.3*Pi/2;
qxx=qx*Cos[phi]-qy*Sin[phi];
qyy=qx*Sin[phi]+qy*Cos[phi];
```

```
phi1=0.1*Pi/2;
phi2=0.9*Pi/2;
qs=10^lqs;
qq=Sqrt[qx*qx+qy*qy];
qxs1=qs*Cos[phi1];
qys1=qs*Sin[phi1];
qxs2=qs*Cos[phi2];
qys2=qs*Sin[phi2];
(* isotropic formfactor *)
Pqiso=Sum[2*(4^n)*Pochhammer[z+1,2*n]*((-
qs*qs*R*R/(4*(z+1)*(z+1)))^n)/(Pochhammer[2,n]*((n+2)!)), {n, 0, 60}];
Pqisoa=2*Gamma[z-1]*((z+1)^2)/(Gamma[z+1]*qs*qs*R*R);
(* perfectly oriented formfactor *)
Pq1a1=Sum[(4^(n+1))*Gamma[n+3/2]*Pochhammer[z+1,2*n]*((-
1/(4*(z+1)*(z+1)))^n)*((qxs1*R)^(2*n))/(Sqrt[Pi]*((n+2)!)*Pochhammer[2,n]*(n!)), {n, 0,
max}];
Pq1a2=Sum[(4^(n+1))*Gamma[n+3/2]*Pochhammer[z+1,2*n]*((-
1/(4*(z+1)*(z+1)))^n)*((qxs2*R)^(2*n))/(Sqrt[Pi]*((n+2)!)*Pochhammer[2,n]*(n!)), {n, 0,
max}];
(* series expansion *)
Pq5a1=Sum[((4^(n+1))*Gamma[n+3/2]*Pochhammer[z+1,2*n]*((-
qs*qs*R*R/(4*(z+1)*(z+1)))^n)/(Sqrt[Pi]*((n+2)!)*Pochhammer[2,n]))*Sum[(((2*m)!)*((-
1)^m)/((4^m)*(m!)*((n-m)!)))*Sum[(((qxs1/qs)^(2*k))*((qys1/qs)^(2*(m-k)))/((m-k)!))*Sum[
(4^l)*((Sin[theta])^(2*l)) *((Cos[theta])^(2*(k-l)))*ccc[[m+1,l+1]]/(((2*l)!)*((k-l)!)*((m-
l)!)), {l, 0, k}], {k, 0, m}],{m, 0, n}],{n, 0, max}];
Pq5a2=Sum[((4^(n+1))*Gamma[n+3/2]*Pochhammer[z+1,2*n]*((-
qs*qs*R*R/(4*(z+1)*(z+1)))^n)/(Sqrt[Pi]*((n+2)!)*Pochhammer[2,n]))*Sum[(((2*m)!)*((-
1)^m)/((4^m)*(m!)*((n-m)!)))*Sum[(((qxs2/qs)^(2*k))*((qys2/qs)^(2*(m-k)))/((m-k)!))*Sum[
(4^l)*((Sin[theta])^(2*l)) *((Cos[theta])^(2*(k-l)))*ccc[[m+1,l+1]]/(((2*l)!)*((k-l)!)*((m-
l)!)), {l, 0, k}], {k, 0, m}],{m, 0, n}],{n, 0, max}];
(* 2D *)
Pq3=Sum[((4^(n+1))*Gamma[n+3/2]*Pochhammer[z+1,2*n]*((-
qq*qq*R*R/(4*(z+1)*(z+1)))^n)/(Sqrt[Pi]*((n+2)!)*Pochhammer[2,n]))*Sum[(((2*m)!)*((-
1)^m)/((4^m)*(m!)*((n-m)!)))*Sum[(((qxx/qq)^(2*k))*((qyy/qq)^(2*(m-k)))/((m-k)!))*Sum[
(4^l)*((Sin[theta])^(2*l)) *((Cos[theta])^(2*(k-l)))*ccc[[m+1,l+1]]/(((2*l)!)*((k-l)!)*((m-
l)!)), {l, 0, k}], {k, 0, m}],{m, 0, n}],{n, 0, max}];
(* numerical integration *)
u1=Gamma[z-1]/(2*Gamma[z+1]);
(* argz1=(L*(qxs1*Cos[delta]-qys1*Sin[chi]*Sin[delta])/(z+1));
argz2=(L*(qxs2*Cos[delta]-qys2*Sin[chi]*Sin[delta])/(z+1));
Pqintz1=NIntegrate[((argz1)^(-2))*(1-Cos[(z-1)*ArcTan[2*argz1]]/((1+4*argz1*argz1)^((z-
1)/2)))*Exp[-delta/del]*Sin[delta], {chi, 0, 2*Pi},{delta, 0, Pi/2}];
Pqintz2=NIntegrate[((argz2)^(-2))*(1-Cos[(z-1)*ArcTan[2*argz2]]/((1+4*argz2*argz2)^((z-
1)/2)))*Exp[-delta/del]*Sin[delta], {chi, 0, 2*Pi},{delta, 0, Pi/2}];
Pqintn=NIntegrate[Exp[-delta/del]*Sin[delta], {delta, 0, Pi/2}];
Pqint1=(u1/(2*Pi))*Pqintz1/Pqintn;
Pqint2=(u1/(2*Pi))*Pqintz2/Pqintn; *)
lim=1;
pl1=Plot[Log[10,Pq1a1], {lqs, -1, lim}, PlotRange->{-3,1}, GridLines->{Automatic},
LabelStyle->Directive[Black,16],AxesLabel->{"log q","lg P(q)"}, AxesOrigin->{-1,-
3},TicksStyle->Directive[Black,10],PlotStyle->{Blue,Dashed}];
pl2=Plot[Log[10,Pq1a2], {lqs, -1, lim}, PlotRange->{-3,1}, GridLines->{Automatic},
LabelStyle->Directive[Black,16],AxesLabel->{"log q","lg P(q)"}, AxesOrigin->{-1,-
3},TicksStyle->Directive[Black,10],PlotStyle->{Blue,Dashed}];
pl3=Plot[Log[10,Pq5a1], {lqs, -1, lim}, PlotRange->{-3,1}, GridLines->{Automatic},
LabelStyle->Directive[Black,16],AxesLabel->{"log q","lg P(q)"}, AxesOrigin->{-1,-
3},TicksStyle->Directive[Black,10],PlotStyle->{Black}];
pl4=Plot[Log[10,Pq5a2], {lqs, -1, lim}, PlotRange->{-3,1}, GridLines->{Automatic},
LabelStyle->Directive[Black,16],AxesLabel->{"log q","lg P(q)"}, AxesOrigin->{-1,-
3},TicksStyle->Directive[Black,10],PlotStyle->{Black}];
pl5=Plot[Log[10,Pqiso], {lqs, -1, lim}, PlotRange->{-3,1}, GridLines->{Automatic},
LabelStyle->Directive[Black,16],AxesLabel->{"log q","lg P(q)"}, AxesOrigin->{-1,-
3},TicksStyle->Directive[Black,10]];
pl6=Plot[Log[10,Pqisoa], {lqs, -1, lim}, PlotRange->{-3,1}, GridLines->{Automatic},
LabelStyle->Directive[Black,16],AxesLabel->{"log q","lg P(q)"}, AxesOrigin->{-1,-
3},TicksStyle->Directive[Black,10]];
```

```
(* pl7=Plot[Log[10,Pqint1], {lqs, -1, lim}, PlotRange□{-3,1}, GridLines->{Automatic},
LabelStyle->Directive[Black,16],AxesLabel->{"log q","lg P(q)"}, AxesOrigin->{-1,-
3},TicksStyle->Directive[Black,10],PlotStyle->{Red,Dashed}]
pl8=Plot[Log[10,Pqint2], {lqs, -1, lim}, PlotRange□{-3,1}, GridLines->{Automatic},
LabelStyle->Directive[Black,16],AxesLabel->{"log q","lg P(q)"}, AxesOrigin->{-1,-
3},TicksStyle->Directive[Black,10],PlotStyle->{Red,Dashed}] *)
Show[pl1,pl2,pl3,pl4,pl5,pl6]
lims=3;
pl5=DensityPlot[Log[10,Pq3],{qx,-lims,lims},{qy,-lims,lims},PlotRange->{-8,0},PlotPoints-
>50,PlotLegends->Automatic,LabelStyle->Directive[Black,12],AxesLabel->Automatic]
```

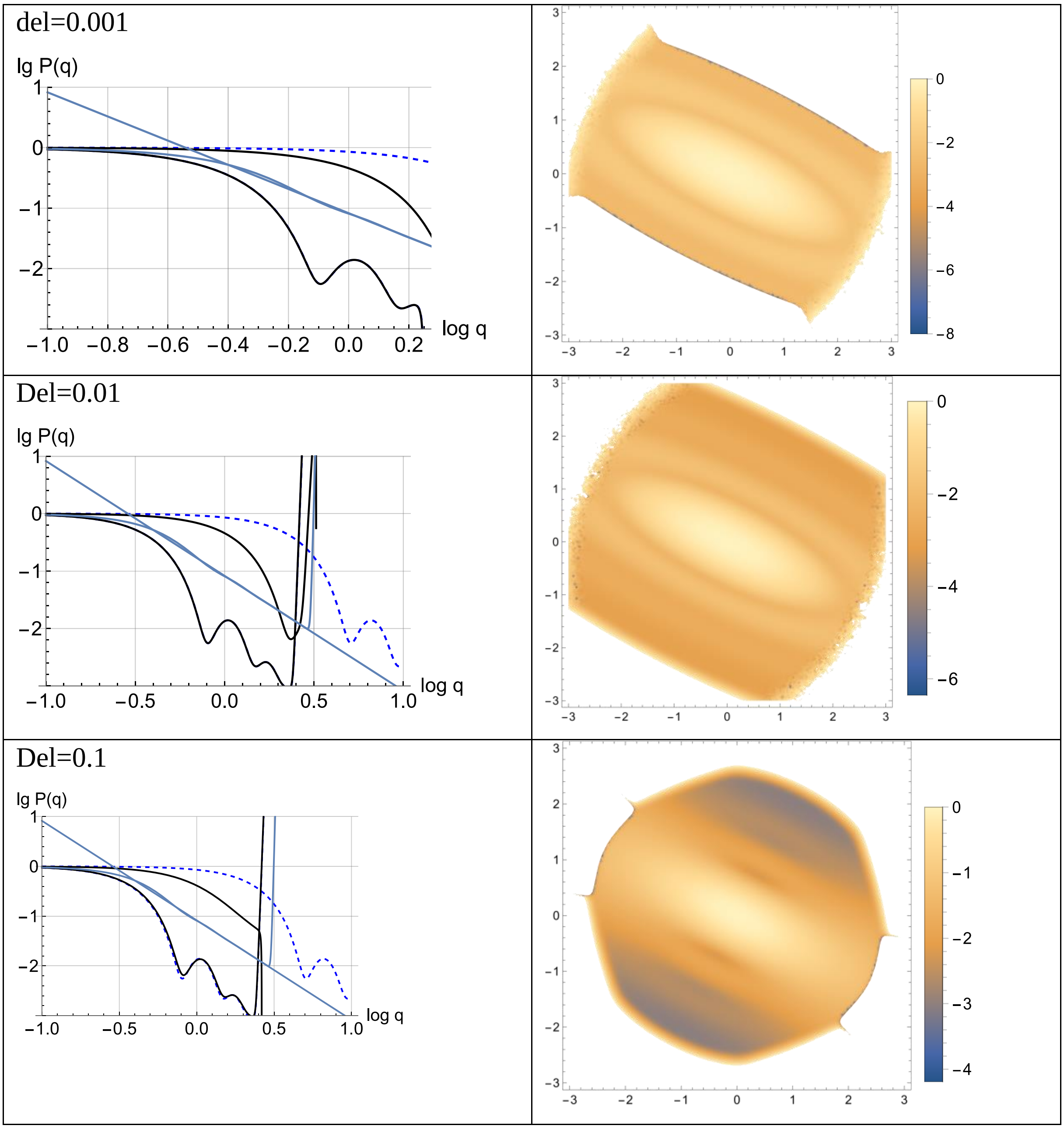

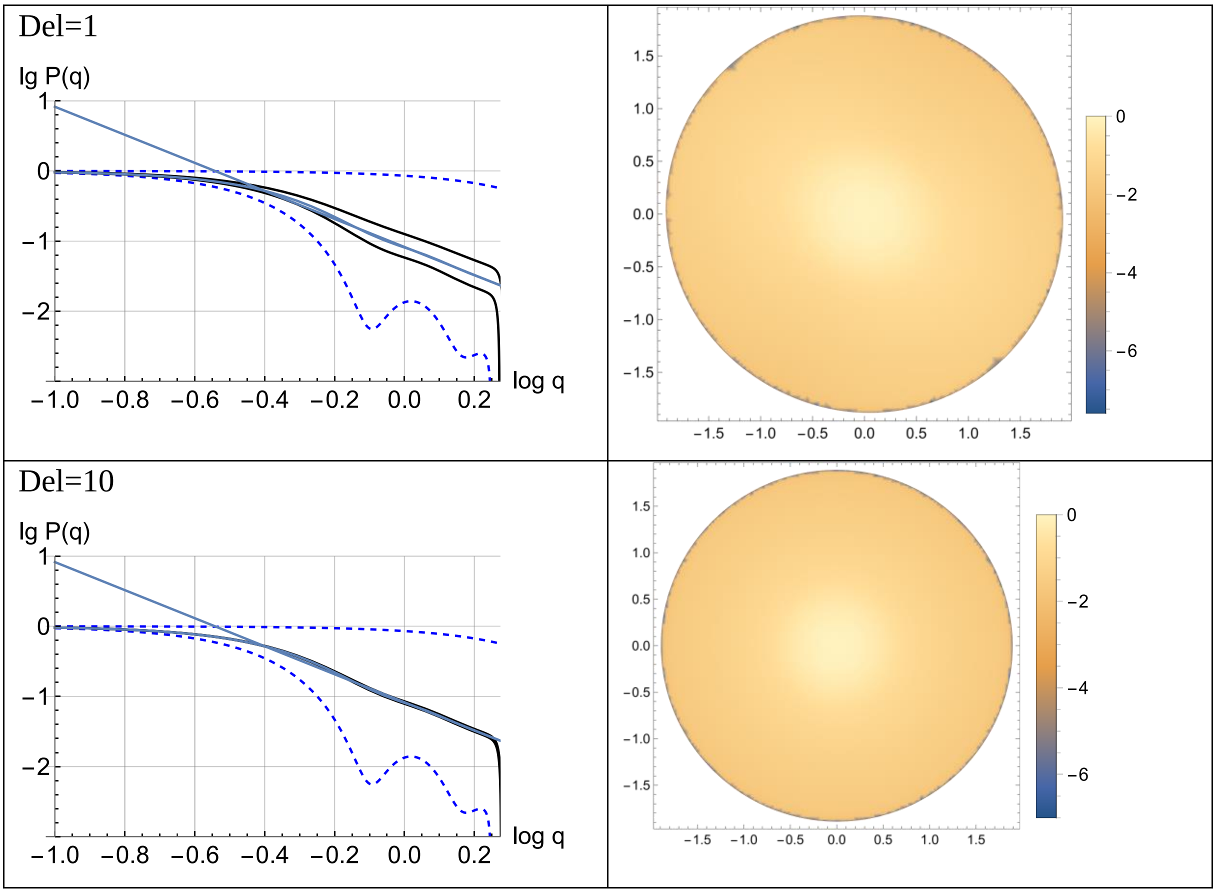

Fig. S63: Formfactor of oriented polydisperse disks with orientational distributions varying from perfectly oriented to the isotropic state.

For rapid calculations the q-independent coefficients are precalculated, such that the series can be quickly calculated for each pixel (q_x, q_y)

$$\langle P_{\parallel}(q_x, q_y, L)\rangle = \sum_{n=0}^{\infty} a_n q^{2n} \sum_{m=0}^{n} b_{n,m} \sum_{k=0}^{m} c_{k,l} (q_x^2)^k (q_y^2)^{m-k}$$

Isotropic Limit

In the following we will show that the expression correctly yields the isotropic limit for the case $\bar{\delta} \to \infty$, for which $h(\delta) \to 1$. Then the integral over the deviation angle can be solved analytically to obtain

$$H_{2l,2m-2l+1} = \int_0^{\pi/2} (\cos(\delta))^{2l} (\sin(\delta))^{2m-2l+1} d\delta = \frac{\Gamma\left[\frac{2l+1}{2}\right]\Gamma\left[\frac{2m-2l+2}{2}\right]}{2\Gamma\left[\frac{2m+3}{2}\right]}$$

This can be inserted into Eq. (S.7.7.2.4) to obtain

$$(\boldsymbol{qR})^{2n} = (qR)^{2n}(n)!\sum_{m=0}^{n}\frac{(2m)!\,(-1)^m}{4^m m!\,(n-m)!\,\Gamma\left[\frac{2m+3}{2}\right]}\sum_{\boldsymbol{k=0}}^{\boldsymbol{m}}\frac{(q_x^2)^k\left(q_y^2\right)^{m-k}}{(\mathrm{m-k})!}\sum_{\boldsymbol{l=0}}^{k}\frac{4^l\left(\sin^2(\theta)\right)^l\left(\cos^2(\theta)\right)^{k-l}}{\Gamma[2l+1](\mathrm{k-l})!}\frac{\Gamma\left[\frac{2l+1}{2}\right]}{2}$$

using Eq. (S.7.4.1.4) with $x = 2l + 1$ yields the simplified expression

$$(\boldsymbol{qR})^{2n} = (qR)^{2n}\frac{(n)!\sqrt{\pi}}{2}\sum_{m=0}^{n}\frac{(2m)!\,(-1)^m}{4^m m!\,(n-m)!\,\Gamma\left[\frac{2m+3}{2}\right]}\sum_{\boldsymbol{k=0}}^{\boldsymbol{m}}\frac{\left(q_x^2\right)^k\left(q_y^2\right)^{m-k}}{(\mathrm{m-k})!\,\mathrm{k}!}\sum_{\boldsymbol{l=0}}^{k}\frac{k!\left(\sin^2(\theta)\right)^l\left(\cos^2(\theta)\right)^{k-l}}{(\mathrm{k-l})!\,\mathrm{l}!}$$

Where the last binomial can be simplified to obtain

$$(\boldsymbol{qR})^{2n} = (qR)^{2n}\frac{(n)!\sqrt{\pi}}{2}\sum_{m=0}^{n}\frac{(2m)!\,(-1)^m}{4^m m!\,(n-m)!\,\Gamma\left[\frac{2m+3}{2}\right]}\sum_{\boldsymbol{k=0}}^{\boldsymbol{m}}\frac{(q_x^2)^k\left(q_y^2\right)^{m-k}}{(\mathrm{m-k})!\,\mathrm{k}!}$$

Which can be further reduced to

$$(\boldsymbol{qR})^{2n} = (qr)^{2n}(n)!\sum_{m=0}^{n}\frac{(2m)!\sqrt{\pi}}{2m!\,4^m\Gamma\left[m+\frac{3}{2}\right]}\frac{(-1)^m}{(n-m)!\,m!}$$

With the identity Eq. (S.7.4.1.7) we have

$$(\boldsymbol{qR})^{2n} = (qR)^{2n}\frac{(n)!}{2}\sum_{m=0}^{n}\frac{1}{\left(m+\frac{1}{2}\right)}\frac{(-1)^m}{(n-m)!\,m!}$$

Which with the special identiy Eq. (S.7.4.2.5) simplifies to

$$(\boldsymbol{qR})^{2n} = (qR)^{2n}\frac{(n)!\sqrt{\pi}}{2\Gamma\left[n+\frac{3}{2}\right]}$$

This is the desired equation that is equal to the expression in Eq. (S.4.7.1.2) relating to the integral

$$\int_0^{\frac{\pi}{2}}(\sin(\phi))^{2n}\sin\phi\,d\phi = \frac{\sqrt{\pi}\Gamma(n+1)}{2\Gamma\left(n+\frac{3}{2}\right)} = \frac{\sqrt{\pi}(n)!}{2\Gamma\left(n+\frac{3}{2}\right)}$$

Regime II. For intermediate and large q-values we use the numerical integration

$$P^{(2)}(q_rR) = \langle P_\perp(q_rR)\rangle = \frac{1}{2\pi H}\int_0^{\pi/2}\int_0^{2\pi}\langle\left(\frac{2J_1(\boldsymbol{qR})}{\boldsymbol{qR}}\right)^2\rangle\, h(\delta)(\sin(\delta)\,d\chi d\delta$$

$$H = \int_0^{\pi/2} h(\delta)(\sin(\delta)\,d\delta$$

where the integrand is computed in terms of the series expansion Eq. (S.1.2.53) as

$$\langle\left(\frac{2J_1(\boldsymbol{qR})}{\boldsymbol{qR}}\right)^2\rangle = \sum_{n=0}^{\infty}\frac{4^{n+1}\Gamma\left(n+\frac{3}{2}\right)}{\sqrt{\pi}\Gamma(n+3)}\frac{(z_R+s_R+1)_{2n}}{(2)_n n!}\left(-\frac{\boldsymbol{q}^2\boldsymbol{R}^2}{4(z_R+1)^2}\right)^n$$

or via the asymptotic expansion Eq. (S.1.2.54) as

$$\langle\left(\frac{2J_1(\boldsymbol{qR})}{\boldsymbol{qR}}\right)^2\rangle = \frac{4}{\pi}\left(\left(\frac{1}{\boldsymbol{qR}}\right)^3 - \left(\frac{1}{\boldsymbol{qR}}\right)^3 \sin(2\boldsymbol{qR}) - \frac{9}{8}\left(\frac{1}{\boldsymbol{qR}}\right)^4 \cos(2\boldsymbol{qR}) + \left(\frac{9}{16}\right)^2\left(\frac{1}{\boldsymbol{qR}}\right)^5 + \left(\frac{9}{16}\right)^2\left(\frac{1}{\boldsymbol{qR}}\right)^5 \sin(2\boldsymbol{qR})\right)$$

with

$$\boldsymbol{qR} = qR\sqrt{1-\left(q_x(\cos(\delta)\sin(\theta)-\cos(\chi)\cos(\theta)\sin(\delta)) - q_y\sin(\chi)\sin(\delta)\right)^2}$$

where for the integrand the expressions for regime I, II, and III can be used, depending on the value of the argument $\boldsymbol{qR}$.

The function

$$P(\boldsymbol{qR}) = \frac{1}{1+\frac{\pi\Gamma[z+1]}{4\Gamma[z-2]}\left(\frac{\boldsymbol{qR}}{z+1}\right)^3}$$

can be well uased as an approximation for polydisperse cases or broad orientational distributins where the formfactor oscillations are smoothed out.

7.8. Parallelepipeds with axial orientational distribution

We use the equatorially averaged formfactor to describe the axial orientation (S.6.1.3.1)

Regime I. For prolate shapes (c>a,b) and oblate shapes (c<a,b) the above derived equations can be used.

Prolate: (S.7.8.1)

$$\langle P(q)\rangle = \frac{1}{4}\sum_{n=0}^{\infty}\frac{4^n(z+1)_{2n}}{\left(n+\frac{1}{2}\right)(n+1)!}\left(-\frac{q^2a^2}{4(z+1)^2}\right)^n \sum_{m=0}^{\infty}\frac{4^m(z+1)_{2m}}{\Gamma(n+m+1)\left(m+\frac{1}{2}\right)(m+1)!}\left(-\frac{q^2\delta^2a^2}{4(z+1)^2}\right)^m \left(\sum_{l=0}^{\infty}\frac{4^l(z+1)_{2l}}{\left(\frac{3}{2}\right)_l(l+1)!}\left(-\frac{\boldsymbol{q^2c^2}}{4(z+1)^2}\right)^l\right)$$

Oblate: (S.7.8.2)

$$\langle P(q)\rangle = \frac{1}{4}\sum_{n=0}^{\infty}\frac{4^n(z+1)_{2n}}{\left(n+\frac{1}{2}\right)(n+1)!}\left(-\frac{\boldsymbol{q_r^2a^2}}{4(z+1)^2}\right)^n \sum_{m=0}^{\infty}\frac{4^m(z+1)_{2m}}{\Gamma(n+m+1)\left(m+\frac{1}{2}\right)(m+1)!}\left(-\frac{q_r^2\delta^2a^2}{4(z+1)^2}\right)^m \left(\sum_{l=0}^{\infty}\frac{4^l(z+1)_{2l}}{\left(\frac{3}{2}\right)_l(l+1)!}\left(-\frac{q^2c^2}{4(z+1)^2}\right)^l\right)$$

As an example we consider the oblate case

$$\langle(\boldsymbol{qc})^{2l}\rangle = (qc)^{2l}\frac{(2l)!}{4^l}\sum_{k=0}^{l}\frac{4^k(\hat{q}_x^2)^k(\hat{q}_y^2)^{l-k}}{(2k)!\left((\mathrm{l-k})!\right)^2}H_{2k,2l-2k}$$

and insert into the series expansion

$$
\begin{aligned}
&\langle P(q)\rangle \\
&= \frac{1}{4}\sum_{n=0}^{\infty}\frac{4^n (z+1)_{2n}}{\left(n+\frac{1}{2}\right)(n+1)!}\left(-\frac{q^2a^2}{4(z+1)^2}\right)^n \sum_{m=0}^{\infty}\frac{4^m (z+1)_{2m}}{\Gamma(n+m+1)\left(m+\frac{1}{2}\right)(m+1)!}\left(-\frac{q^2\delta^2a^2}{4(z+1)^2}\right)^m \left(\sum_{l=0}^{\infty}\frac{4^l (z+1)_{2l}}{\left(\frac{3}{2}\right)_l (l+1)!}\left(-\frac{\boldsymbol{q}^2\boldsymbol{c}^2}{4(z+1)^2}\right)^l \frac{(2l)!}{4^l}\sum_{k=0}^{l}\frac{4^k (\hat{q}_x^2)^k \left(\hat{q}_y^2\right)^{l-k}}{(2k)!\left((l-k)!\right)^2} H_{2k,2l-2k}\right) \\
&= \frac{1}{4}\sum_{n=0}^{\infty}\frac{4^n (z+1)_{2n}}{\left(n+\frac{1}{2}\right)(n+1)!}\left(-\frac{q^2a^2}{4(z+1)^2}\right)^n \sum_{m=0}^{\infty}\frac{4^m (z+1)_{2m}}{(n+m)!\left(m+\frac{1}{2}\right)(m+1)!}\left(-\frac{q^2\delta^2a^2}{4(z+1)^2}\right)^m \left(\sum_{l=0}^{\infty}\frac{(2l)!\,(z+1)_{2l}}{\left(\frac{3}{2}\right)_l (l+1)!}\left(-\frac{\boldsymbol{q}^2\boldsymbol{c}^2}{4(z+1)^2}\right)^l \sum_{k=0}^{l}\frac{4^k (\hat{q}_x^2)^k \left(\hat{q}_y^2\right)^{l-k}}{(2k)!\left((l-k)!\right)^2} H_{2k,2l-2k}\right)
\end{aligned}
$$

Regime II. From Eq. (S.6.2.1.2) we have the product of averaged trigonometric functions

$$
P_x(q,\phi) = \left\langle \frac{(\sin(qa))^2}{(qa\cos(\phi))^2}\right\rangle = \frac{\Gamma[z-1]}{\Gamma[z+1]}\frac{1}{2}\left(\frac{z+1}{qa\cos(\phi)}\right)^2 \left(1-\frac{\cos\left[(z-1)\arctan\left(\frac{2qa\cos(\phi)}{z+1}\right)\right]}{\left(1+\left(\frac{2qa\cos(\phi)}{z+1}\right)^2\right)^{\frac{z-1}{2}}}\right)
$$

$$
P_y(q,\phi) = \left\langle \frac{(\sin(qb))^2}{(qb\sin(\phi))^2}\right\rangle = \frac{\Gamma[z-1]}{\Gamma[z+1]}\frac{1}{2}\left(\frac{z+1}{qb\sin(\phi)}\right)^2 \left(1-\frac{\cos\left[(z-1)\arctan\left(\frac{2qb\sin(\phi)}{z+1}\right)\right]}{\left(1+\left(\frac{2qb\sin(\phi)}{z+1}\right)^2\right)^{\frac{z-1}{2}}}\right)
$$

$$
P_z(q) = \left\langle \frac{(\sin(\boldsymbol{qc}))^2}{(\boldsymbol{qc})^2}\right\rangle = \frac{\Gamma[z-1]}{\Gamma[z+1]}\frac{1}{2}\left(\frac{z+1}{\boldsymbol{qc}}\right)^2 \left(1-\frac{\cos\left[(z-1)\arctan\left(\frac{2\boldsymbol{qc}}{z+1}\right)\right]}{\left(1+\left(\frac{2\boldsymbol{qc}}{z+1}\right)^2\right)^{\frac{z-1}{2}}}\right)
$$

$$
P(q) = \langle P_z(q)\rangle \frac{2}{\pi}\int_0^{\pi/2} P_x(q,\phi)P_y(q,\phi)d\phi
$$

with

$$
\langle P_z(q)\rangle = \frac{1}{2\pi H}\int_0^{\pi/2}\int_0^{2\pi} \left\langle \frac{(\sin(\boldsymbol{qc}))^2}{(\boldsymbol{qc})^2}\right\rangle h(\delta)(\sin(\delta)\,d\chi d\delta
$$

$$
H = \int_0^{\pi/2} h(\delta)(\sin(\delta)\,d\delta
$$

$$
(\boldsymbol{qc})^2 = (qc)^2\left(\hat{q}_x\cos(\delta) - \hat{q}_y\sin(\chi)\sin(\delta)\right)^2
$$

Mathematica code implementation:

```
del=0.01;
max=40;
```

```
ccc=Table[NIntegrate[((Cos[delta])^(2*l))*((Sin[delta])^(2*n-2*l+1))*Exp[-delta/del],
{delta, 0, Pi/2}]/NIntegrate[Exp[-delta/del]*Sin[delta], {delta, 0, Pi/2}], {n, 0, max},{l,
0, max}];

a=1;
b=2;
c=3;
del=0.01;
sigma=0.1;
z=(1-sigma*sigma)/(sigma*sigma);
qs=10^lqs;
theta=0.6*Pi/2;
qrs=qs*Sin[theta ];
qzs=qs*Cos[theta];
q=Sqrt[qr*qr+qz*qz];
Pq1=(1/4)*Sum[(Pochhammer[z+1,2*n]*(4^n)*((-
qs*qs*a*a/(4*(z+1)*(z+1)))^n)/((n+1/2)*((n+1)!)))*Sum[(Pochhammer[z+1,2*m]*(4^m)*((-
qs*qs*b*b/(4*(z+1)*(z+1)))^m)/((m+1/2)*((m+1)!)*((n+m)!)))*Sum[(((2*l)!)*Pochhammer[z+1,2*l
]*((-
qs*qs*c*c/(4*(z+1)*(z+1)))^l)/(Pochhammer[3/2,l]*((l+1)!)))*Sum[(4^k)*((qrs/qs)^(2*k))*((qz
s/qs)^(2*(l-k)))*ccc[[l+1,k+1]]/(((2*k)!)*((l-k)!)*((l-k)!)), {k, 0, l}],{l, 0, max}], {m,
0, max}], {n, 0, max}];
Pq1a=(1/4)*Sum[(Pochhammer[z+1,2*n]*(4^n)*((-
q*q*a*a/(4*(z+1)*(z+1)))^n)/((n+1/2)*((n+1)!)))*Sum[(Pochhammer[z+1,2*m]*(4^m)*((-
q*q*b*b/(4*(z+1)*(z+1)))^m)/((m+1/2)*((m+1)!)*((n+m)!)))*Sum[(((2*l)!)*Pochhammer[z+1,2*l]*
((-
q*q*c*c/(4*(z+1)*(z+1)))^l)/(Pochhammer[3/2,l]*((l+1)!)))*Sum[(4^k)*((qr/q)^(2*k))*((qz/q)^
(2*(l-k)))*ccc[[l+1,k+1]]/(((2*k)!)*((l-k)!)*((l-k)!)), {k, 0, l}],{l, 0, max}], {m, 0,
max}], {n, 0, max}];
argxs=qs*a*Cos[phi]/(z+1);
argys=qs*b*Sin[phi]/(z+1);
argzs=qs*c*((qrs/qs)*Cos[delta]-(qzs/qs)*Sin[chi]*Sin[delta])/(z+1);
pxs=(Gamma[z-1]/(2*Gamma[z+1]))*(argxs^(-2))*(1-Cos[(z-
1)*ArcTan[2*argxs]]/((1+4*argxs*argxs)^((z-1)/2)));
pys=(Gamma[z-1]/(2*Gamma[z+1]))*(argys^(-2))*(1-Cos[(z-
1)*ArcTan[2*argys]]/((1+4*argys*argys)^((z-1)/2)));
pzs=(Gamma[z-1]/(2*Gamma[z+1]))*(argzs^(-2))*(1-Cos[(z-
1)*ArcTan[2*argzs]]/((1+4*argzs*argzs)^((z-1)/2)));
pxys=NIntegrate[pxs*pys, {phi, 0, Pi/2 }]/(Pi/2);
pzsint=(2/Pi)*NIntegrate[pzs*Exp[-delta/del]*Sin[delta], {chi, 0, Pi/2},{delta, 0, Pi/2}]/
NIntegrate[Exp[-delta/del]*Sin[delta], {delta, 0, Pi/2}];
Pq2=pxys*pzsint;
lim=1;
pl1=Plot[Log[10,Pq1], {lqs, -1,lim}, PlotRange->{-6,1}, GridLines->{Automatic}, LabelStyle-
>Directive[Black,16],AxesLabel->{"log q","lg P(q)"}, AxesOrigin->{-1,-6},TicksStyle-
>Directive[Black,12],PlotStyle->{Black}] ;
pl2=Plot[Log[10,Pq2], {lqs, -1,lim}, PlotRange->{-6,1}, GridLines->{Automatic}, LabelStyle-
>Directive[Black,16],AxesLabel->{"log q","lg P(q)"}, AxesOrigin->{-1,-6},TicksStyle-
>Directive[Black,12],PlotStyle->{Blue,Dashed}];
Show[pl1,pl2]
lims=3;
pl3=DensityPlot[Log[10,Pq1a],{qr,  -lims,  lims},  {qz,  -lims,  lims},  PlotRange->{-5,0},
PlotPoints->50,     PlotLegends->Automatic,     LabelStyle->Directive[Black,12],AxesLabel-
>Automatic]
```

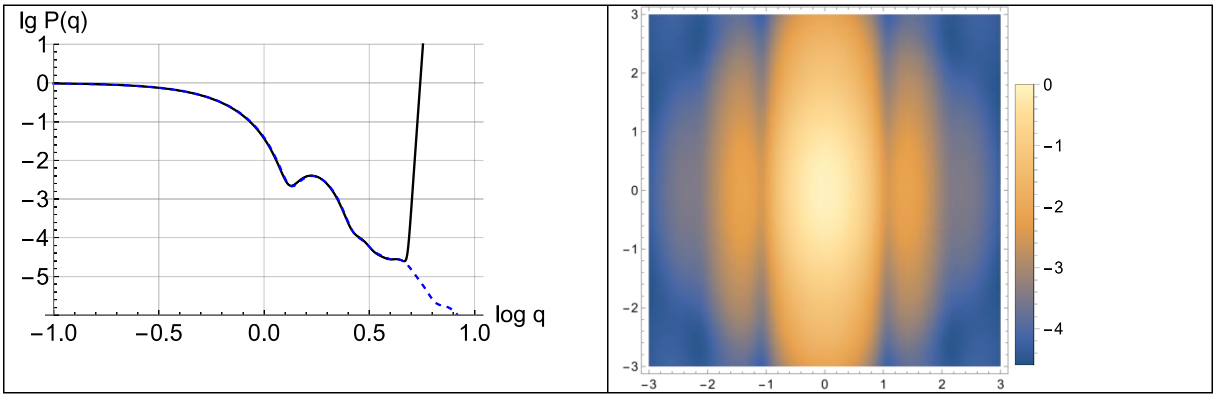

Fig. S64: Formfactor of oriented polydisperse parallelepiped with a=1, b=2, and c=3 nm.

7.9. Oriented Ellipsoids

7.9.1 Rotation Body

Regime I. We consider the case $k = 2$ for the rotation body. In the prolate case we have (S.7.9.1.1)

$$P(q) = \frac{9}{16}\sum_{n=0}^{\infty}(z+1)_{2n}\left(-\frac{\boldsymbol{q_z^2 L^2}}{4(z+1)^2}\right)^n \sum_{m=0}^{\infty}(z+1)_{2n}\left(-\frac{q^2R^2}{4(z+1)^2}\right)^m c_{n,m}$$

and for the oblate case we have (S.7.9.1.2)

$$P(q) = \frac{9}{16}\sum_{n=0}^{\infty}(z+1)_{2n}\left(-\frac{q^2L^2}{4(z+1)^2}\right)^n \sum_{m=0}^{\infty}(z+1)_{2n}\left(-\frac{\boldsymbol{q_r^2 R^2}}{4(z+1)^2}\right)^m c_{n,m}$$

We now consider the prolate case with the axis parallel to the x-axis as an example. For the series expansion we therefore use Eq. (7.4.1.5)

$$\langle(\boldsymbol{qL})^{2n}\rangle = (qL)^{2n}\frac{(2n)!}{4^n}\sum_{l=0}^{n}\frac{4^l(\hat{q}_x^2)^l(\hat{q}_y^2)^{n-l}}{(2l)!\left((\mathrm{n}-\mathrm{l})!\right)^2}H_{2l,2n-2l}$$

wich is inserted into the series expansion Eq. (7.9.1.1) to obtain

$$P(q) = \frac{9}{16}\sum_{n=0}^{\infty}\frac{(2n)!\,(z+1)_{2n}}{4^n}\left(-\frac{q^2L^2}{4(z+1)^2}\right)^n\left(\sum_{l=0}^{n}\frac{4^l(\hat{q}_x^2)^l(\hat{q}_y^2)^{n-l}}{(2l)!\left((\mathrm{n}-\mathrm{l})!\right)^2}H_{2l,2n-2l}\right)\left(\sum_{m=0}^{\infty}(z+1)_{2n}\left(-\frac{q^2R^2}{4(z+1)^2}\right)^m c_{n,m}\right)$$

Regime III. We take the Porod-asymptote for the biaxial ellipsoid Eq. (6.3.1.2)

$$\lim_{q\to\infty} P(q) = \frac{\Gamma(z-3)(z+1)^4}{\Gamma(z+1)}\frac{9}{2(q^2R^2+\boldsymbol{q^2L^2})^2}$$

and use Eq. (S.7.4.1.2)

$$(\boldsymbol{qL})^2 = (qL)^2 \left(\hat{q}_x \cos(\delta) - \hat{q}_y \sin(\chi) \sin(\delta)\right)^2$$

This is then integrated over the angles as

$$\langle P_{\parallel}(q, L)\rangle = \frac{1}{2\pi H} \int_0^{\pi/2} \int_0^{2\pi} P(q)\, h(\delta)(\sin(\delta)\, d\chi d\delta$$

$$= \frac{\Gamma(z-3)(z+1)^4}{\Gamma(z+1)} \frac{1}{2\pi H} \frac{9}{2} \int_0^{\pi/2} \int_0^{2\pi} \frac{h(\delta)}{\left(q^2R^2 + (qL)^2\left(\hat{q}_x \cos(\delta) - \hat{q}_y \sin(\chi)\sin(\delta)\right)^2\right)^2} \sin(\delta)\, d\chi d\delta$$

$$H = \int_0^{\pi/2} h(\delta)(\sin(\delta)\, d\delta$$

Mathematica code implementation:

```
k=2;
del=0.01;
na=45;
ma=na;
ff=Table[Sum[(Gamma[(2*ns+1)/k]*Gamma[(2*(n-
ns)+1)/k]/(Pochhammer[1/2,ns]*(ns!)*Pochhammer[1/2,n-ns]*((n-
ns)!)))*Sum[(Gamma[(2*ms+2+k)/k]*Gamma[(2*(m-
ms)+2+k)/k]/(Pochhammer[2,ms]*(ms!)*Pochhammer[2,m-ms]*((m-
ms)!)))*(1/(Gamma[(2*(ns+ms)+k+3)/k]*Gamma[(2*(n-ns+m-
ms)+k+3)/k])),{ms,0,m}],{ns,0,n}],{n,0,na},{m,0,ma}];
ccc=Table[NIntegrate[((Cos[delta])^(2*l))*((Sin[delta])^(2*n-2*l+1))*Exp[-delta/del],
{delta, 0, Pi/2}]/NIntegrate[Exp[-delta/del]*Sin[delta], {delta, 0, Pi/2}], {n, 0, na},{l,
0, na}];

R1=1.5;
L1=4;
sigma=0.11;
zz=(1-sigma*sigma)/(sigma*sigma);
theta=0.1*Pi/2-0.001;
qs=10^lqs;
qrs=qs*Sin[theta];
qzs=qs*Cos[theta];
q=Sqrt[qr*qr+qz*qz];

a1=3/4;
apor=Gamma[zz-3]*((zz+1)^4)/Gamma[zz+1];
Pqav=a1*a1*Sum[(((2*n)!)*Pochhammer[zz+1,2*n]*((-
qs*qs*L1*L1/(4*(zz+1)*(zz+1)))^n)/(4^n))*Sum[((4^l)*((qrs/qs)^(2*l))*((qzs/qs)^(2*(n-
l)))*ccc[[n+1,l+1]]/(((2*l)!)*((n-l)!)*((n-l)!))), {l, 0, n}]*Sum[(Pochhammer[zz+1,2*m]*((-
qs*qs*R1*R1/(4*(zz+1)*(zz+1)))^m))*ff[[n+1,m+1]],{m,0,ma}],{n,0,na}];
Pqavz=a1*a1*Sum[(((2*n)!)*Pochhammer[zz+1,2*n]*((-
q*q*L1*L1/(4*(zz+1)*(zz+1)))^n)/(4^n))*Sum[((4^l)*((qr/q)^(2*l))*((qz/q)^(2*(n-
l)))*ccc[[n+1,l+1]]/(((2*l)!)*((n-l)!)*((n-l)!))), {l, 0, n}]*Sum[(Pochhammer[zz+1,2*m]*((-
q*q*R1*R1/(4*(zz+1)*(zz+1)))^m))*ff[[n+1,m+1]],{m,0,ma}],{n,0,na}];
Rkk=Sqrt[((qs*R1)^2)+((qs*L1*((qrs/qs)*Cos[delta]-(qzs/qs)*Sin[chi]*Sin[delta]))^2)];
Pqpor=(9*apor/2)*(2/Pi)*NIntegrate[Exp[-delta/del]*Sin[delta]/(Rkk^4), {chi, 0,
Pi/2},{delta, 0, Pi/2}]/NIntegrate[Exp[-delta/del]*Sin[delta],{delta, 0, Pi/2}];
lim=1.5;
pl1=Plot[Log[10,Pqav], {lqs, -1,lim}, PlotRange->{-6,1}, GridLines->{Automatic},
LabelStyle->Directive[Black,16],AxesLabel->{"log q","lg P(q)"}, AxesOrigin->{-1,-
6},TicksStyle->Directive[Black,12],PlotStyle->{Black}] ;
pl2=Plot[Log[10,Pqpor], {lqs, -1,lim}, PlotRange->{-6,1}, GridLines->{Automatic},
LabelStyle->Directive[Black,16],AxesLabel->{"log q","lg P(q)"}, AxesOrigin->{-1,-
6},TicksStyle->Directive[Black,12],PlotStyle->{Blue,Dashed}] ;
```

```
Show[pl1,pl2]
lims=5;
pl3=DensityPlot[Log[10,Pqavz],{qr, -lims, lims}, {qz, -lims, lims}, PlotRange->{-5,0}, PlotPoints->50, PlotLegends->Automatic, LabelStyle->Directive[Black,12],AxesLabel->Automatic]
```

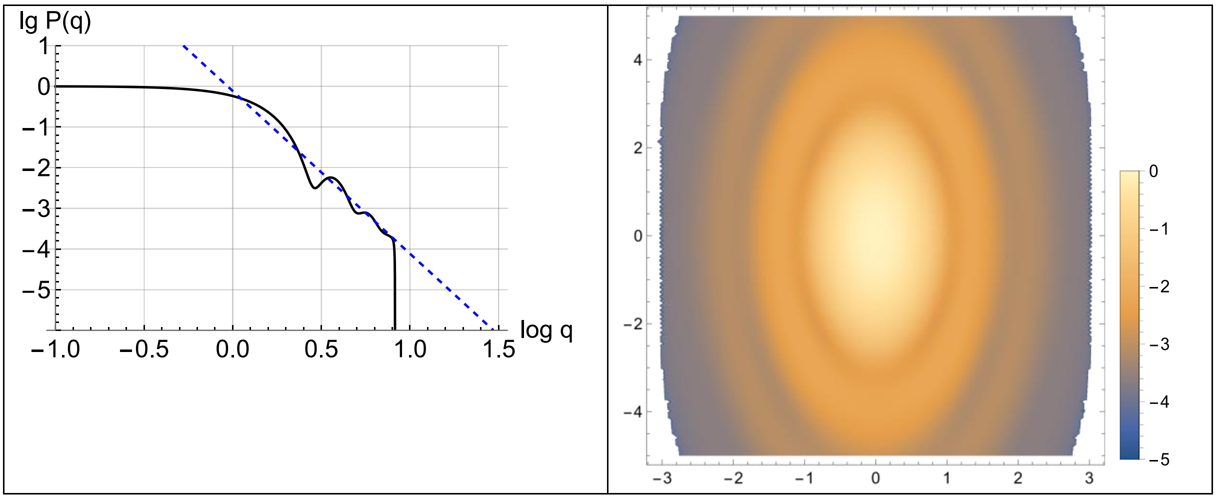

Fig. S65: Formfactor of oriented polydisperse ellipsoid with circular cross-section and R=1.5 and L=4.

We do not consider F(q), because there is no Porod-asymptote and thus no high-q option.

7.9.2 Rotation body with elliptical cross-section

Regime I. We consider the case $k = 2$ for the rotation body. In the prolate case we have (S.7.9.2.1)

$$P(q) = \frac{9}{16}\sum_{n=0}^{\infty}(z+1)_{2n}\left(-\frac{\boldsymbol{q^2L^2}}{4(z+1)^2}\right)^n \sum_{m=0}^{\infty}(z+1)_{2n}\left(-\frac{q^2a^2}{4(z+1)^2}\right)^m e_m c_{n,m}$$

In the oblate case we have (S.7.9.2.2)

$$P(q) = \frac{9}{16}\sum_{n=0}^{\infty}(z+1)_{2n}\left(-\frac{q^2L^2}{4(z+1)^2}\right)^n \sum_{m=0}^{\infty}(z+1)_{2n}\left(-\frac{\boldsymbol{q^2a^2}}{4(z+1)^2}\right)^m e_m c_{n,m}$$

$$e_m = \frac{1}{\pi}\sum_{l=0}^{m}\frac{(\varepsilon^2)^{m-l}}{(m-l)!\,l!}\Gamma\left(m-l+\frac{1}{2}\right)\Gamma\left(l+\frac{1}{2}\right)$$

with $\varepsilon = b/a$. We now consider the prolate case with the axis parallel to the x-axis as an example. For the series expansion we therefore use

$$\langle(\boldsymbol{qL})^{2n}\rangle = (qL)^{2n}\frac{(2n)!}{4^n}\sum_{l=0}^{n}\frac{4^l(\hat{q}_x^2)^l(\hat{q}_y^2)^{n-l}}{(2l)!\,((\mathrm{n}-\mathrm{l})!)^2}H_{2l,2n-2l}$$

wich is inserted into the series expansion to obtain

$$P(q) = \frac{9}{16}\sum_{n=0}^{\infty}\frac{(2n)!\,(z+1)_{2n}}{4^n}\left(-\frac{q^2L^2}{4(z+1)^2}\right)^n\left(\sum_{l=0}^{n}\frac{4^l(\hat{q}_x^2)^l(\hat{q}_y^2)^{n-l}}{(2l)!\,((\mathrm{n}-\mathrm{l})!)^2}H_{2l,2n-2l}\right)\left(\sum_{m=0}^{\infty}(z+1)_{2n}\left(-\frac{q^2a^2}{4(z+1)^2}\right)^m e_m c_{n,m}\right)$$

Regime III. We take the Porod-asymptote for the biaxial ellipsoid

$$\lim_{q\to\infty}P(q,\phi) = \frac{\Gamma(z-3)(z+1)^4}{\Gamma(z+1)}\frac{9}{2((qa\cos\phi)^2+(qb\sin\phi)^2+(qL)^2)^2}$$

$$= \frac{\Gamma(z-3)(z+1)^4}{\Gamma(z+1)}\frac{9}{2(q^2(a^2-b^2)(\cos\phi)^2+q^2b^2+q^2L^2)^2}$$

We integrate

$$\lim_{q\to\infty}P(q) = \frac{\Gamma(z-3)(z+1)^4}{\Gamma(z+1)}\frac{9}{2}\frac{2}{\pi}\int_0^{\pi/2}\frac{1}{(q^2(a^2-b^2)(\cos\phi)^2+q^2b^2+q^2L^2)^2}d\phi$$

The integral has an analytical solution

$$\frac{2}{\pi}\int_0^{\pi/2}\frac{1}{(s+t(\cos\phi)^2)^2}d\phi = \frac{2}{\pi}\frac{\pi(2s+t)}{4s^{3/2}(s+t)^{3/2}} = \frac{(2s+t)}{2s^{3/2}(s+t)^{3/2}}$$

with $s = q^2b^2+q^2L^2$ and $t = q^2(a^2-b^2)$. Inserting into the Porod asymptote yields

$$\lim_{q\to\infty}P(q) = \frac{\Gamma(z-3)(z+1)^4}{\Gamma(z+1)}\frac{9}{2}\frac{(2(q^2b^2+q^2L^2)+q^2(a^2-b^2))}{2(q^2b^2+q^2L^2)^{3/2}(q^2b^2+q^2L^2+q^2(a^2-b^2))^{3/2}}$$

$$= \frac{\Gamma(z-3)(z+1)^4}{\Gamma(z+1)}\frac{9}{4}\frac{(q^2a^2+q^2b^2+2q^2L^2)}{(q^2b^2+q^2L^2)^{3/2}(q^2a^2+q^2L^2)^{3/2}}$$

We use

$$(\boldsymbol{qL})^2 = (qL)^2\left(\hat{q}_x\cos(\delta)-\hat{q}_y\sin(\chi)\sin(\delta)\right)^2$$

to obtain

$$\lim_{q\to\infty}P(q)$$
$$= \frac{\Gamma(z-3)(z+1)^4}{\Gamma(z+1)}\frac{9}{4}\frac{(q^2a^2+q^2b^2+2(qL)^2(\hat{q}_x\cos(\delta)-\hat{q}_y\sin(\chi)\sin(\delta))^2)}{(q^2b^2+(qL)^2(\hat{q}_x\cos(\delta)-\hat{q}_y\sin(\chi)\sin(\delta))^2)^{3/2}\left(q^2a^2+(qL)^2(\hat{q}_x\cos(\delta)-\hat{q}_y\sin(\chi)\sin(\delta))^2\right)^{3/2}}$$

$$= \frac{\Gamma(z-3)(z+1)^4}{\Gamma(z+1)}\frac{9}{4q^4}\frac{(a^2+b^2+2L^2(\hat{q}_x\cos(\delta)-\hat{q}_y\sin(\chi)\sin(\delta))^2)}{(b^2+L^2(\hat{q}_x\cos(\delta)-\hat{q}_y\sin(\chi)\sin(\delta))^2)^{3/2}\left(a^2+L^2(\hat{q}_x\cos(\delta)-\hat{q}_y\sin(\chi)\sin(\delta))^2\right)^{3/2}}$$

This is then integrated over the angles as

$$\langle P_{\parallel}(q,L)\rangle = \frac{1}{2\pi H}\int_0^{\pi/2}\int_0^{2\pi} P(q)\, h(\delta)(\sin(\delta)\, d\chi d\delta$$

$$= \frac{\Gamma(z-3)(z+1)^4}{\Gamma(z+1)}\frac{9}{4q^4}\frac{1}{2\pi H}\int_0^{\pi/2}\int_0^{2\pi}\frac{(a^2+b^2+2L^2\left(\hat{q}_x\cos(\delta)-\hat{q}_y\sin(\chi)\sin(\delta)\right)^2)h(\delta)}{(b^2+L^2\left(\hat{q}_x\cos(\delta)-\hat{q}_y\sin(\chi)\sin(\delta)\right)^2)^{3/2}\left(a^2+L^2\left(\hat{q}_x\cos(\delta)-\hat{q}_y\sin(\chi)\sin(\delta)\right)^2\right)^{3/2}}\sin(\delta)\, d\chi d\delta$$

$$H = \int_0^{\pi/2} h(\delta)(\sin(\delta)\, d\delta$$

Mathematica code implementation:

```
a=1.5;
b=2.3;
eps=b/a;
k=2;
del=0.01;
na=55;
ma=na;
ff=Table[Sum[(Gamma[(2*ns+1)/k]*Gamma[(2*(n-
ns)+1)/k]/(Pochhammer[1/2,ns]*(ns!)*Pochhammer[1/2,n-ns]*((n-
ns)!)))*Sum[(Gamma[(2*ms+2+k)/k]*Gamma[(2*(m-
ms)+2+k)/k]/(Pochhammer[2,ms]*(ms!)*Pochhammer[2,m-ms]*((m-
ms)!)))*(1/(Gamma[(2*(ns+ms)+k+3)/k]*Gamma[(2*(n-ns+m-
ms)+k+3)/k])),{ms,0,m}],{ns,0,n}],{n,0,na},{m,0,ma}];
ccc=Table[NIntegrate[((Cos[delta])^(2*l))*((Sin[delta])^(2*n-2*l+1))*Exp[-delta/del],
{delta, 0, Pi/2}]/NIntegrate[Exp[-delta/del]*Sin[delta], {delta, 0, Pi/2}], {n, 0, na},{l,
0, na}];
ee=Table[(1/Pi)*Sum[(eps^(2*(m-l)))*Gamma[m-l+1/2]*Gamma[l+1/2]/(((m-l)!)*(l!)), {l, 0,
m}], {m, 0, ma}];

L1=4;
sigma=0.11;
z=(1-sigma*sigma)/(sigma*sigma);
theta=0.1*Pi/2-0.001;
qs=10^lqs;
qrs=qs*Sin[theta];
qzs=qs*Cos[theta];
q=Sqrt[qr*qr+qz*qz];
a1=3/4;
apor=Gamma[z-3]*((z+1)^4)/Gamma[z+1];
Pqav=a1*a1*Sum[(((2*n)!)*Pochhammer[z+1,2*n]*((-
qs*qs*L1*L1/(4*(z+1)*(z+1)))^n)/(4^n))*Sum[((4^l)*((qrs/qs)^(2*l))*((qzs/qs)^(2*(n-
l)))*ccc[[n+1,l+1]]/(((2*l)!)*((n-l)!)*((n-l)!))), {l, 0, n}]*Sum[(Pochhammer[z+1,2*m]*((-
qs*qs*a*a/(4*(z+1)*(z+1)))^m))*ff[[n+1,m+1]]*ee[[m+1]],{m,0,ma}],{n,0,na}];
Pqavz=a1*a1*Sum[(((2*n)!)*Pochhammer[z+1,2*n]*((-
q*q*L1*L1/(4*(z+1)*(z+1)))^n)/(4^n))*Sum[((4^l)*((qr/q)^(2*l))*((qz/q)^(2*(n-
l)))*ccc[[n+1,l+1]]/(((2*l)!)*((n-l)!)*((n-l)!))), {l, 0, n}]*Sum[(Pochhammer[z+1,2*m]*((-
q*q*a*a/(4*(z+1)*(z+1)))^m))*ff[[n+1,m+1]]*ee[[m+1]],{m,0,ma}],{n,0,na}];

Lax2=((qs*L1*((qrs/qs)*Cos[delta]-(qzs/qs)*Sin[chi]*Sin[delta]))^2);
Rkk=(((qs*a)^2)+((qs*b)^2)+2*Lax2)/(((((qs*b)^2)+Lax2)^(3/2))*((((qs*a)^2)+Lax2)^(3/2)));
Pqpor=(9*apor/(4))*(2/Pi)*NIntegrate[Exp[-delta/del]*Sin[delta]*Rkk, {chi, 0, Pi/2},{delta,
0, Pi/2}]/NIntegrate[Exp[-delta/del]*Sin[delta],{delta, 0, Pi/2}];
lim=1.5;
pl1=Plot[Log[10,Pqav], {lqs, -1,lim}, PlotRange->{-6,1}, GridLines->{Automatic},
LabelStyle->Directive[Black,16],AxesLabel->{"log q","lg P(q)"}, AxesOrigin->{-1,-
6},TicksStyle->Directive[Black,12],PlotStyle->{Black}] ;
pl2=Plot[Log[10,Pqpor], {lqs, -1,lim}, PlotRange->{-6,1}, GridLines->{Automatic},
LabelStyle->Directive[Black,16],AxesLabel->{"log q","lg P(q)"}, AxesOrigin->{-1,-
6},TicksStyle->Directive[Black,12],PlotStyle->{Blue, dashed}] ;
Show[pl1,pl2]
lims=4;
```

```
pl3=DensityPlot[Log[10,Pqavz],{qr, -lims, lims}, {qz, -lims, lims}, PlotRange->{-5,0},
PlotPoints->50, PlotLegends->Automatic, LabelStyle->Directive[Black,12],AxesLabel-
>Automatic]
```

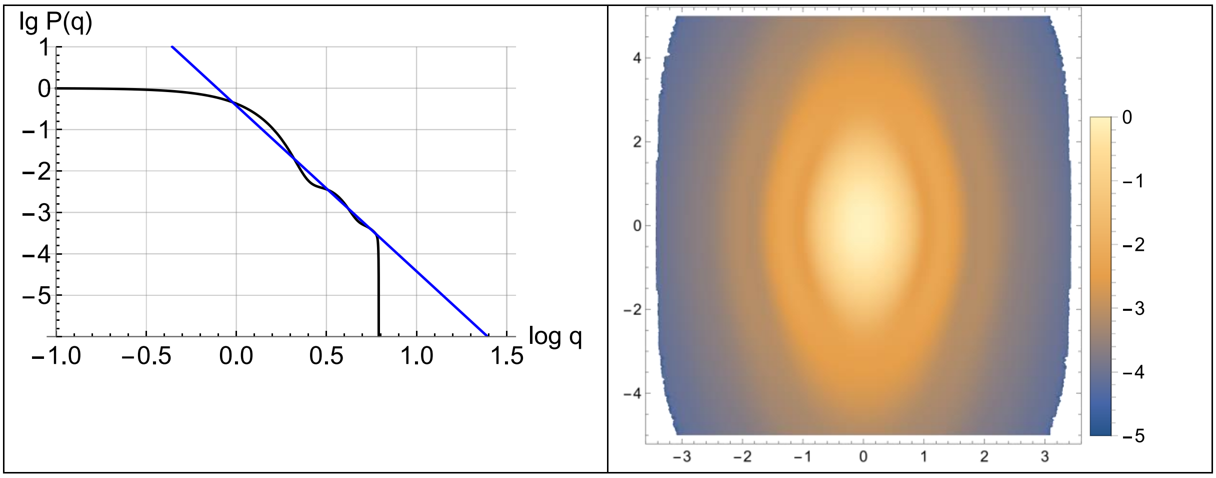

Fig. S66: Formfactor of oriented polydisperse ellipsoids with elliptical cross-section with a=1.5, b=2.3 and c=4 nm.

8. Ordered Lattices

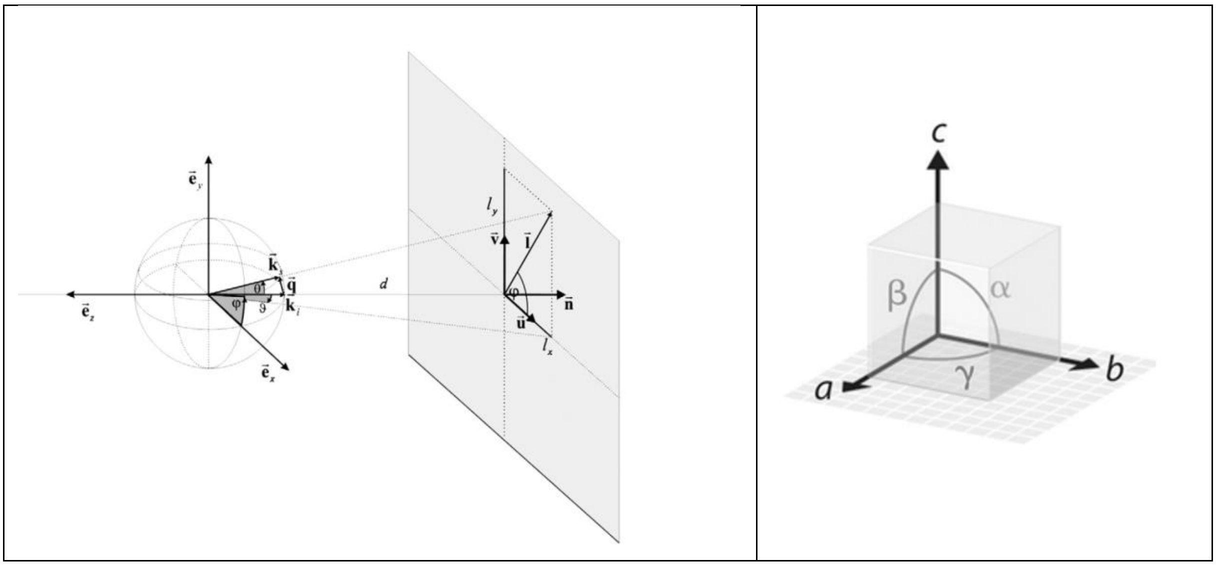

Fig. S67: Lattice spatial orientations (left) and unit cell (right).

8.1 Spatial Orientations

We assume the orthonormal Carthesian lab-coordinate system as in Fig. S50. In this system, the *x*-axis is parallel to the detector horizontal axis, the *y*-axis is parallel to the detector vertical

axis, and the primary beam direction $\boldsymbol{n}$ is in the negative z-axis direction, i.e. $\boldsymbol{n} = (0,0,-1)$. The unit vectors in the *x*-, *y*-, and *z*-direction are $\boldsymbol{e}_x$, $\boldsymbol{e}_y$, and $\boldsymbol{e}_z$.

We assume to have a unit cell given by the unit cell edge lengths *a*, *b*, *c* and the three angles α, β, γ as shown in Fig. S50. They define the unit cell base vectors $\boldsymbol{a_A}, \boldsymbol{b_A}, \boldsymbol{c_A}$ in the unit cell coordinate system $\boldsymbol{A} = (\boldsymbol{a_A}, \boldsymbol{b_A}, \boldsymbol{c_A})$.

We introduce the parameters

$$(ca) = \cos\alpha\,, \qquad (sa) = \sin\alpha$$

$$(cb) = \cos\beta\,, \qquad (sb) = \sin\beta$$

$$(cg) = \cos\gamma, \qquad (sg) = \sin\gamma$$

Then the volume of the unit cell is given by

$$V = abc\sqrt{1-(ca)^2-(cb)^2-(cg)^2+2(ca)(cb)(cg)}$$

We use the convention that the c-axis of the unit cell or the c-axis of an anisometric particle is parallel to $\boldsymbol{e}_z$. Then the primary beam transects the unit cell or the particle parallel to its c-axis from the top to the bottom. We further chose to have the a-axis of the unit cell or the particle in the (x, y)-plane such that e.g. in orthogonal unit cells it is parallel to $\boldsymbol{e}_x$.

A vector $\boldsymbol{r_{uvw}}$ specifying a certain position (u, v, w) or crystallographic direction $[u, v, w]$ within the unit cell can be given in terms of the unit cell base vectors as

$$\boldsymbol{r_{uvw}} = u\boldsymbol{a_A} + v\boldsymbol{b_A} + w\boldsymbol{c_A}$$

In the Carthesian lab coordinate system $\boldsymbol{E} = (\boldsymbol{e}_x, \boldsymbol{e}_y, \boldsymbol{e}_z)$ the vector is given by

$$\boldsymbol{r}_{xyz} = x\boldsymbol{e}_x + y\boldsymbol{e}_x + z\boldsymbol{e}_x$$

The coordinates of the vector are related by a matrix $\boldsymbol{M}$ as

$$\boldsymbol{X}_E = \begin{pmatrix} x \\ y \\ z \end{pmatrix} = \boldsymbol{M}\begin{pmatrix} u \\ v \\ w \end{pmatrix} = \boldsymbol{M}\boldsymbol{X}_A$$

We derive the matrix $\boldsymbol{M}$ by introducing the parameters

$$n_1 = 0, \qquad n_2 = 0, \qquad n_3 = 1$$

$$l_1 = (sb), \qquad l_2 = 0, \qquad l_3 = (cb)$$

$$m_1 = \frac{(cg)-(cb)(ca)}{(sb)}, \qquad m_2 = \sqrt{1-m_1^2-m_3^2}, \qquad m_3 = (ca)$$

to define the matrix

$$\boldsymbol{M_S} = \begin{pmatrix} al_1 & al_2 & al_3 \\ bm_1 & bm_2 & bm_3 \\ cn_1 & cn_2 & cn_3 \end{pmatrix}$$

The desired matrix $\boldsymbol{M}$ is obtained as the transposed matrix **(S.8.1.1)**

$$\boldsymbol{M} = \boldsymbol{M_S^T}$$

Then we have

$$\boldsymbol{a_E} = \boldsymbol{M}\boldsymbol{a_A}$$

$$\boldsymbol{b_E} = \boldsymbol{M}\boldsymbol{b_A}$$

$$\boldsymbol{c_E} = \boldsymbol{M}\boldsymbol{c_A}$$

The inverse martrix is given by

$$\boldsymbol{M^{-1}} = \begin{pmatrix} -\frac{m_2}{a(l_2m_1 - l_1m_2)} & \frac{m_1}{a(l_2m_1 - l_1m_2)} & 0 \\ \frac{l_2}{b(l_2m_1 - l_1m_2)} & -\frac{l_1}{b(l_2m_1 - l_1m_2)} & 0 \\ \frac{l_3m_2 - l_2m_3}{c(l_2m_1 - l_1m_2)} & -\frac{l_3m_1 - l_1m_3}{c(l_2m_1 - l_1m_2)} & \frac{1}{c} \end{pmatrix}$$

We here just give two examples for a rhombohedral $(a, b, c;\ \alpha = \beta = \gamma = \pi/2)$ and a hexagonal unit cell $(a, a, c;\ \alpha = \beta = \frac{\pi}{2}, \gamma = \frac{4\pi}{3})$

$$\boldsymbol{M} = \begin{pmatrix} a & 0 & 0 \\ 0 & b & 0 \\ 0 & 0 & c \end{pmatrix}$$

$$\boldsymbol{M} = \begin{pmatrix} a & 0 & 0 \\ \frac{a}{2} & \frac{\sqrt{3}a}{2} & 0 \\ 0 & 0 & c \end{pmatrix}$$

We suppose that the unit cell is oriented such that a direction $\boldsymbol{u_E} = (u_x, u_y, u_z)$ in terms of the unit cell base vectors in **E** is aligned parallel to the beam direction $\boldsymbol{n_E} = (0,0,-1)$. This defines a rotation matrix $\boldsymbol{R}$ which we can use to rotate the base vectors accordingly. The matrix is given by **(S.8.1.2)**

$$\boldsymbol{R} = \begin{pmatrix} \cos\beta\cos\gamma & \sin\alpha\sin\beta\cos\gamma - \cos\alpha\sin\gamma & \cos\alpha\sin\beta\cos\gamma + \sin\alpha\sin\gamma \\ \cos\beta\sin\gamma & \sin\alpha\sin\beta\sin\gamma + \cos\alpha\cos\gamma & \cos\alpha\sin\beta\sin\gamma - \sin\alpha\cos\gamma \\ -\sin\beta & \sin\alpha\cos\beta & \cos\alpha\cos\beta \end{pmatrix}$$

with $\alpha = -\text{sgn}(u_y)\arccos\left[\frac{-u_z}{\sqrt{u_y^2+u_z^2}}\right]$, $\beta = \text{sgn}(u_x)\arccos\left[\frac{-u_{rz}}{\sqrt{u_x^2+u_{rz}^2}}\right]$, $u_{rz} = u_y\sin\alpha + u_z\cos\alpha$, and γ freely selectable. The inverse matrix is $\boldsymbol{R^{-1}} = \boldsymbol{R^T}$. With this matrix we can rotate the base vectors to obtain the set of rotated base vectors $(\boldsymbol{a_{Er}}, \boldsymbol{b_{Er}}, \boldsymbol{c_{Er}})$

$$\boldsymbol{a_{E,r}} = \boldsymbol{Ra_E}$$

$$\boldsymbol{b_{E,r}} = \boldsymbol{Rb_E}$$

$$\boldsymbol{c_{E,r}} = \boldsymbol{Rc_E}$$

These are then used to compute the reciprocal space vectors $(\boldsymbol{a}^*, \boldsymbol{b}^*, \boldsymbol{c}^*)$. Therefore, we use the matrix

$$\widetilde{\boldsymbol{G}} = (\boldsymbol{a_{E,r}}, \boldsymbol{b_{E,r}}, \boldsymbol{c_{E,r}})$$

and compute **(S.8.1.3)**

$$\boldsymbol{G} = \widetilde{\boldsymbol{G}}^{-\boldsymbol{1}} = \begin{pmatrix} \boldsymbol{a}^* \\ \boldsymbol{b}^* \\ \boldsymbol{c}^* \end{pmatrix}$$

to obtain the set of reciprocal space base vectors $(\boldsymbol{a}^*, \boldsymbol{b}^*, \boldsymbol{c}^*)$. Using the previous relations S.8.1.1, S.8.1.2 and S.8.1.3, we can directly calculate the reciprocal space vectors as

$$\begin{pmatrix} \boldsymbol{a}^* \\ \boldsymbol{b}^* \\ \boldsymbol{c}^* \end{pmatrix} = \boldsymbol{G} = (\boldsymbol{RM}^*\boldsymbol{a_A}, \boldsymbol{RM}^*\boldsymbol{b_A}, \boldsymbol{RM}^*\boldsymbol{b_A})^{-\boldsymbol{1}}$$

The reciprocal space vector $\boldsymbol{q}_{hkl}^*$ is then obtained as **(S.8.1.4)**

$$\boldsymbol{q}_{hkl}^* = \begin{pmatrix} \boldsymbol{a}^* \\ \boldsymbol{b}^* \\ \boldsymbol{c}^* \end{pmatrix}^T \begin{pmatrix} h \\ k \\ l \end{pmatrix} = (\boldsymbol{a}^*, \boldsymbol{b}^*, \boldsymbol{c}^*) \begin{pmatrix} h \\ k \\ l \end{pmatrix} = h\boldsymbol{a}^* + k\boldsymbol{b}^* + l\boldsymbol{c}^*$$

If needed, the detector x- and y-axis directions can be expressed by backrotation $\boldsymbol{R}^{-1}$ and inverse transform $\boldsymbol{M}^{-1}$ to the respective crystallographic directions in the unit cell.

Mathematica code implementation:

```
(* define crystal *)
a = 1;
b = 2;
c = 3;
alf = 90*Pi/180;
bet = 90*Pi/180;
gam = 90*Pi/180;
(* define crystal base vectors *)
aav = {1, 0, 0};
bav = {0, 1, 0};
cav = {0, 0, 1};
(* calculate crystal A - orthogonal E transformation matrix M *)
(* assume c||ez, a in (ez,ex)-plane *)
ca = Cos[alf];       cb = Cos[bet];       cg = Cos[gam];
sa = Sin[alf];       sb = Sin[bet];       sg = Sin[gam];
```

```
V = a*b*c*Sqrt[1 - ca*ca - cb*cb - cg*cg + 2*ca*cb*cg];
n1 = 0;
n2 = 0;
n3 = 1;
l1 = sb;
l2 = 0;
l3 = cb;
m1 = (cg - cb*ca)/sb;
m3 = Cos[alf];
m2 = Sqrt[1 - m1*m1 - m3*m3];
ll = a*{l1, l2, l3};
mm = b*{m1, m2, m3};
nn = c*{n1, n2, n3};
MS = {ll, mm, nn};
M = Transpose[MS];
MatrixForm[M];
aev = M . aav
bev = M . bav
cev = M . cav
(**************************)
(* pick u||beam, normalize *)
(**************************)
u = {1, 0, 0.00000000001};
ux = u[[1]];
uy = u[[2]];
uz = u[[3]];
uxs = Sign[ux];
uys = Sign[uy];
(* project u in (y,z)-plane, such that x=0 *)
uyz = {0, uy, uz};
(* determine enclosed angle with beam = {0,0,-1} *)
beam = {0, 0, -1};
luyz = Norm[uyz];
alfa = -uys*ArcCos[uyz . beam/luyz];
(* rotate u by alfa *)
Rx = {{1, 0, 0}, {0, Cos[alfa], -Sin[alfa]}, {0, Sin[alfa],
    Cos[alfa]}};
ur = Rx . u;
urx = ur[[1]];
ury = ur[[2]];
urz = ur[[3]];
(* determine enclosed angle with beam *)
lur = Norm[ur];
beta = uxs*ArcCos[ur . beam/lur];
(* rotate ur by beta *)
Ry = {{Cos[beta], 0, Sin[beta]}, {0, 1, 0}, {-Sin[beta], 0,
    Cos[beta]}};
urr = Ry . ur;
(* rotate urr by gamma around z-axis *)
gamma = 0;
Rz = {{Cos[gamma], -Sin[gamma], 0}, {Sin[gamma], Cos[gamma], 0}, {0,
    0, 1}};
urrr = Rz . urr;
RR = Rz . Ry . Rx;
MatrixForm[RR];
(* rotated unit cell base vectors *)
aed = N[RR . aev]
bed = N[RR . bev]
ced = N[RR . cev]
(* create matrix Gi *)
Gi = {aed, bed, ced};
Git = Transpose[Gi];
MatrixForm[Git];
G = Inverse[Git];
MatrixForm[G];
aer = G[[1]]
ber = G[[2]]
cer = G[[3]]
```

```
Output:
unit cell vectors in E: {1,0,0}, {0,2,0}, {0,0,3}
rotated unit cell vectors in E: {0,0,-1}, {0,2,0}, {3,0,0}
reciprocal unit cell vectors in E: {0,0,-1}, {0,1/2,0}, {1/3,0,0}
```

To summarize, the set of reciprocal space vectors $(\boldsymbol{a}^*, \boldsymbol{b}^*, \boldsymbol{c}^*)$ is calculated by three transformations

- $\boldsymbol{M}$ from the unit cell base vectors $(\boldsymbol{a_A}, \boldsymbol{b_A}, \boldsymbol{c_A})$ in the unit cell coordinate system $\boldsymbol{A}$ to the unit cell base vectors $(\boldsymbol{a_E}, \boldsymbol{b_E}, \boldsymbol{c_E})$ given in the Carthesian lab coordinate system $\boldsymbol{E}$,
- $\boldsymbol{R}$ to rotate the vector $\boldsymbol{u_E}$ into a direction parallel to the beam $\boldsymbol{u_E}||\boldsymbol{n}$ from the base vectors $(\boldsymbol{a_E}, \boldsymbol{b_E}, \boldsymbol{c_E})$ to the rotated base vectors $(\boldsymbol{a_{Er}}, \boldsymbol{b_{Er}}, \boldsymbol{c_{Er}})$**,**
- $\boldsymbol{G}$ to transform the rotated base vectors $(\boldsymbol{a_{Er}}, \boldsymbol{b_{Er}}, \boldsymbol{c_{Er}})$ to the corresponding reciprocal space vectors $(\boldsymbol{a}^*, \boldsymbol{b}^*, \boldsymbol{c}^*)$

These are supplied to the (i,j)-loops of the algorithm.

8.2 Lattice Factor

To compute the lattice factor $Z(\boldsymbol{q})$ requires to calculate the structure factors f_{hkl}, the reciprocal space vectors $\boldsymbol{q}^*_{hkl}$, and the parameters of the peak shape function L_{hkl} for all (h,k,l) combinations **(S.8.2.1)**

$$Z(\boldsymbol{q}) = \sum_{h,k,l} f_{hkl} L_{hkl}(\boldsymbol{q}, \boldsymbol{q}^*)$$

Since f_{hkl} , $\boldsymbol{q}^*_{hkl}$ and many peak shape function pameters are q-independent, they can be pre-calculated and provided to the (i,j)-loops in the algorithm for all non-zero f_{hkl}-values.

As an example, we take as the peak shape function a normalized Gaussian wich is of the form **(S.8.2.2)**

$$L(\boldsymbol{q}, \boldsymbol{q}^*_{hkl}) = \left(\frac{2}{\pi}\right)^3 \frac{1}{\sigma_x \sigma_y \sigma_z} \exp\left[-\frac{4}{\pi}\left[\left(\frac{\left(q_x - q^*_{x,hkl}\right)^2}{\sigma_x^2}\right) + \left(\frac{\left(q_y - q^*_{y,hkl}\right)^2}{\sigma_y^2}\right) + \left(\frac{\left(q_z - q^*_{z,hkl}\right)^2}{\sigma_z^2}\right)\right]\right]$$

where the peak widths $\sigma_x, \sigma_y, \sigma_z$ are q-independent and the corresponding factors can be pre-calculated.

Finally, the scattering intensity $I(\boldsymbol{q})$ of the ordered ensembles of objects is given at the level of the decoupling approximation [7,8] **(S.8.2.3)**

$$I(\boldsymbol{q}) = (\Delta b)^2 \rho_N \langle P(\boldsymbol{q})\rangle \left[1 + \frac{\langle F(\boldsymbol{q})\rangle^2}{\langle P(\boldsymbol{q})\rangle}(\langle Z(\boldsymbol{q})\rangle - 1)G(\boldsymbol{q})\right]$$

where Δb is a sample specific contrast factor, ρ_N the particle number density, and $G(q)$ the Debye-Waller factor.

8.3 Orientational averaged lattice factors

The peak shape function can be factorized into a radial part that depends on the modulus q^*_{hkl} of the lattice vector, and an azimuthal part that depends on the angle enclosed by the scattering vector and the reciprocal lattice vector, ψ_{hkl}, as **(S.8.3.1)** [S2]

$$L(\boldsymbol{q}, \boldsymbol{q}^*_{hkl}) = \left(\frac{2}{\pi}\right)^3 L_q(\boldsymbol{q}, q^*_{hkl}) L_\psi(\boldsymbol{q}, \psi_{hkl})$$

$$\psi_{hkl} = \arccos \frac{\boldsymbol{q}\boldsymbol{q}^*_{hkl}}{|\boldsymbol{q}||\boldsymbol{q}^*_{hkl}|}$$

with in case of Gaussian peak shapes

$$L_q(\boldsymbol{q}, q^*_{hkl}) = \frac{2}{\pi\sigma_q} \exp\left[-\frac{4(q_x - q^*_{hkl})^2}{\pi\sigma_q^2}\right]$$

$$L_\psi(\boldsymbol{q}, \psi_{hkl}) = c_G \exp\left[-\frac{4\psi_q^2 q^2}{\pi\sigma_\psi^2}\right]$$

$$c_G = \frac{1}{2\pi q^2_{hkl} K(a_{hkl})}$$

$$a_{hkl} = \frac{4q^2}{\pi\sigma_\psi^2}$$

The function $K(a)$ can be calculated via a series expansion for small a

$$K(a) = 2 - (\pi^2 - 4)a + \frac{(48 - 12\pi^2 + \pi^4)}{2}a^2 - \frac{(-1440 + 360\pi^2 - 30\pi^4 + \pi^6)}{6}a^3 + \cdots$$

and for large a via an asymptotic expansion

$$K(a) = \frac{(1 - e^{-a\pi^2})}{2a} - \frac{1 - e^{-a\pi^2}(1 + a\pi^2)}{12a^2} + \frac{1 - e^{-a\pi^2}(2 + 2a\pi^2 + a^2\pi^4)}{240a^3} - \frac{6 - e^{-a\pi^2}(6 + 6a\pi^2 + 3a^2\pi^4 + a^3\pi^6)}{10080a^4} + \cdots$$

with an overlapping region between the both expansions.

9. Grazing Incidence Scattering

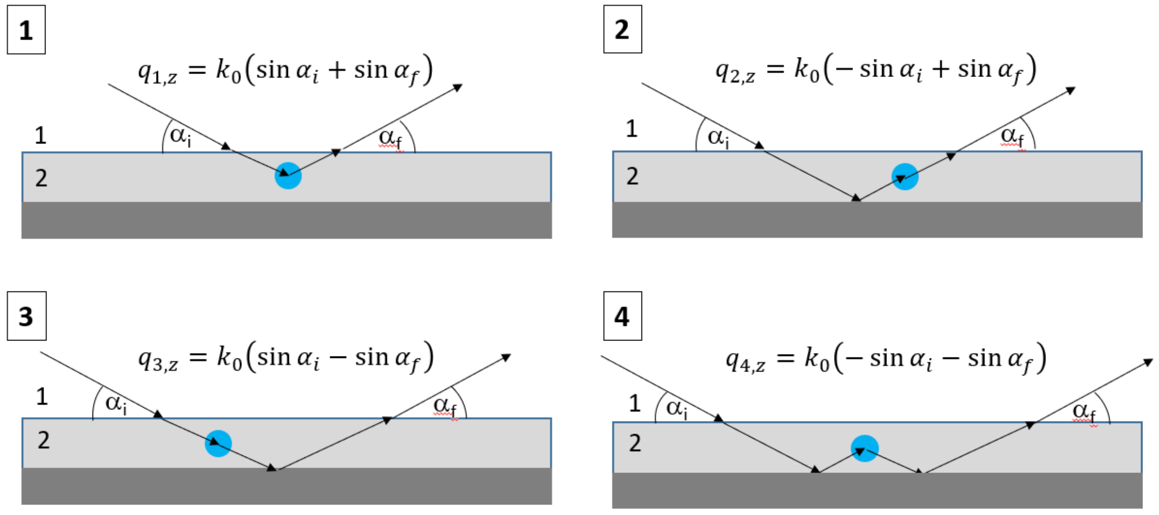

Fig. S68: The four scattering/reflection events considered in the Distorted Wave Born Approximation DWBA. The respective z-components of the scattering vectors are indicated. In the Born approximation (BA) just the first term is considered. Since the refractive index of the layer 2 is $n_2 \approx 1 - 10^{-6}$, the incident and outgoing angles α_i and α_f are practically unchanged upon refraction at the surface.

The analysis of the scattering at angles close to the critical angle requires a calculation in terms of the distorted wave Born approximation (DWBA). The calculation scheme involves integrating the complex phase factor $e^{i\mathbf{qr}}$ over all intraparticle, interparticle, and interface correlations of the objects, taking into account the complex Fresnel transmission and reflection coefficients for the four most important scattering/refraction events as shown in Fig. S51. For these calculation schemes established software packages are available [6].

If intra- and interparticle correlations are already treated at the level of the decoupling approximation (Eq. S.8.2.3), the GISAS scattering intensity can be formulated as a sum over the four terms representing the DWBA scattering/reflection events of the object assembly and the film/substrate interface [16,17] **(S.9.1)**

$$I_{GI}(\boldsymbol{q}) = |T_i|^2|T_f|^2\left(I(\boldsymbol{q}_1) + |R_i|^2 I(\boldsymbol{q}_2) + |R_f|^2 I(\boldsymbol{q}_3) + |R_i|^2|R_f|^2 I(\boldsymbol{q}_4)\right)$$

where the $\boldsymbol{q}_n$ are the scattering vectors of the four scattering/reflection events as shown in Fig. S51, and the $T_{i,f}$ and $R_{i,f}$ are the Fresnel transmission and reflection coefficients, respectively.

In the Born Approximation (BA), just the first term $I(\boldsymbol{q}_1)$ is considered. The scattering vector in these experiments is expressed in terms of the horizontal component q_r and the z-component q_z as $\boldsymbol{q} = (q_r, q_z)$.

The description can be extended to include the incident plane specular reflection and diffuse scattering intensity as [16] **(S.9.2)**

$$\tilde{I}_{GI}(\boldsymbol{q}) = I_{GI}(\boldsymbol{q}) + |T_i|^2 |T_f|^2 [S_{\text{spec}}(\boldsymbol{q}) + S_{\text{diff}}(\boldsymbol{q})\ ]$$

with the specular reflectivity

$$S_{\text{spec}}(q_r, q_z) = \frac{4\pi^2 |R|^2}{q_z^2} \exp[-q_z^2 \sigma^2]\ \delta_{r,z}$$

where σ_z is the roughness, and $\delta_{r,z}$ is the reflection peak, and the diffuse reflectivity

$$S_{\text{diff}}(q_r, q_z) = \frac{A\pi}{\left(q_r^2 + \frac{A^2}{4} q_z^2\right)^{3/2}}$$

with $A = \frac{2\sigma_z^2}{\xi^{2h}}$, where ξ is the cut-off length. This expression is obtained from

$$S_{\text{diff}}(q_r, q_z) = \frac{2\pi}{q_z^2} \int_0^\infty \exp\left[-\frac{q_z^2}{2} A R^{2h}\right] J_0(q_r R) R dR$$

for the case of $h = 1/2$. It merges with the more elaborate expression

$$S_{\text{diff}}(q_r, q_z) = \frac{2\pi}{q_z^2} \exp[-q_z^2 \sigma^2] \int_0^\infty \left(\exp\left[q_z^2 \sigma^2 \exp\left[-\left(\frac{R}{\xi}\right)^{2h}\right]\right] - 1\right) J_0(q_r R) R dR$$

for large q. The latter expression correctly describes the low-q behavior, but can only be computed by numerical integration. A quantitative comparison is provided in Fig. S52.

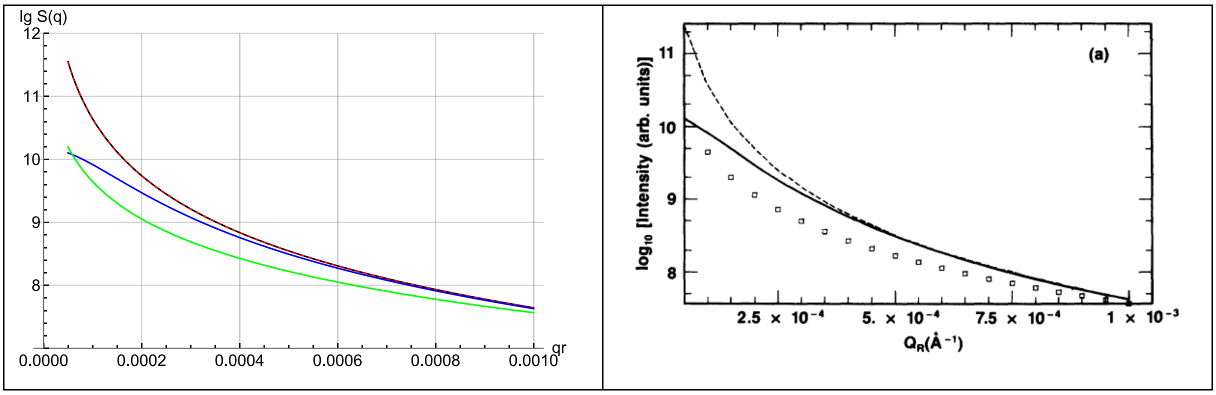

Fig. S69: Incident plane diffuse scattering intensity $S_{\text{diff}}(q_r, q_z)$ in the case $\sigma_z = 7$, ξ=7000, $h = 0.5$, $q_z = 0.01$ according to the analytical expression (black), the integral without cutoff (red, dashed), and the integral with cutoff (blue), and with $h = 0.2$ using the integral with cut-off (green). The data can be directly compared to Fig. 2a of ref. [7].

Mathematica code implementation for Fig. S52:

```
sig=7;
xi=7000;
h=0.5;
A=2*sig*sig/(xi^(2*h));
qz=0.01;
(* analytical, no cutoff *)
fs1=A*Pi/((qr*qr+(qz^4)*A*A/4)^(3/2));
(* integral, with cutoff *)
frr1=BesselJ[0,qr*rr]*rr*(Exp[qz*qz*sig*sig*Exp[-((rr/xi)^(2*h))]]-1);
int1=NIntegrate[frr1, {rr, 0, Infinity}];
ffi1=(2*Pi/(qz*qz))*Exp[-qz*qz*sig*sig]*int1;
(* integral, no cutoff *)
fa1=BesselJ[0,qr*rr]*rr*Exp[-qz*qz*A*(rr^(2*h))/2];
inta1=NIntegrate[fa1, {rr, 0, Infinity}];
ffa1=(2*Pi/(qz*qz))*inta1;
pl1=Plot[Log[10,ffa1],{qr, 0.00005, 0.001},PlotRange->{7,12},GridLines-
>{Automatic},LabelStyle->Directive[Black,16],AxesLabel->{"qr","lg S(q)"},AxesOrigin-
>{0,7},TicksStyle->Directive[Black,12],PlotStyle->{Black}];
pl2=Plot[Log[10,fs1],{qr, 0.00005, 0.001},PlotRange->{7,12},GridLines-
>{Automatic},LabelStyle->Directive[Black,16],AxesLabel->{"qr","lg S(q)"},AxesOrigin-
>{0,7},TicksStyle->Directive[Black,12],PlotStyle->{Red, Dashed}];
pl3=Plot[Log[10,ffi1],{qr, 0.00005, 0.001},PlotRange->{7,12},GridLines-
>{Automatic},LabelStyle->Directive[Black,16],AxesLabel->{"qr","lg S(q)"},AxesOrigin-
>{0,7},TicksStyle->Directive[Black,12],PlotStyle->{Blue}];
Show[pl1,pl2,pl3]
```

The Fresnel transmission and reflection factors are

$$T_{i,f} = \frac{2\alpha_{i,f}}{\alpha_{i,f} + \sqrt{\alpha_{i,f}^2 - \alpha_c^2 + 2i\beta}}$$

$$R_{i,f} = \frac{\alpha_{i,f} - \sqrt{\alpha_{i,f}^2 - \alpha_c^2 + 2i\beta}}{\alpha_{i,f} + \sqrt{\alpha_{i,f}^2 - \alpha_c^2 + 2i\beta}}$$

where α_i is the incident angle, α_f the final angle, α_c the critical angle and β the absorption coefficients. They are related to the refractive index of the material

$$n_2 = 1 - \delta + i\beta$$

with a typical value of the refractive part $\delta \sim 10^{-6}$ and the absorptive part $\beta \sim 10^{-8}$. At the film/air interface with air having a refractive index $n_1 = 1$, one calculates from Snell's law the critical angle α_c as

$$n_2 = \cos \alpha_c$$

with a value of $\alpha_c \sim 0.081°$ derived from the values above. The angle of incidence α_i extends from slightly below α_c to larger values, typically $\alpha_i \sim 0.05 - 0.5$ to cover the Yoneda peak at the critical angle α_c. With the wave vector

$$k_0 = \frac{2\pi}{\lambda}$$

the scattering vector of the horizon is given by

$$q_{z,h} = k_0 \sin \alpha_i$$

and the scattering vector at the critical angle is

$$q_{z,c} = k_0 (\sin \alpha_i + \sin \alpha_c)$$

The scattering vector of the reflected specular beam $\delta_{r,z}$ has a q_z -value of

$$q_{z,ref} = 2q_{z,h}$$

If the particles are buried in a medium of refractive index n_2, the scattering vector q_z of the scattered beam from the particles for the four scattering events is given by

$$q_{1,z} = k_0 (\sin \alpha_{2,i} + \sin \alpha_{2.f})$$

$$q_{2,z} = k_0 (-\sin \alpha_{2,i} + \sin \alpha_{2.f})$$

$$q_{3,z} = k_0 (\sin \alpha_{2,i} - \sin \alpha_{2.f})$$

$$q_{4,z} = k_0 (-\sin \alpha_{2,i} - \sin \alpha_{2.f})$$

$\alpha_{2.i}$ is the angle of incidence on the particle in the material 2, and $\alpha_{2,f}$ the outgoing angle of the scattered beam. They differ from the incident α_i and outgoing beam angles α_f at the surface of the material according to Snell's law

$$\alpha_{2,i} = \arccos \left(\frac{\cos \alpha_i}{n_2} \right)$$

$$\alpha_{2,f} = \arccos\left(\frac{\cos\alpha_{1,f}}{n_2}\right)$$

Since $n_2 \approx 1-\delta \cong 1$, $\alpha_{2,i} \cong \alpha_i$ and $\alpha_{2,f} \cong \alpha_f$. This is illustrated in Fig. S51.

The coefficients T_i and R on the incident angle and the optical properties of the material, are q-independent and can be pre-calculated. Only the transmission coefficient T_f is q-dependent, but can be rapidly computed.

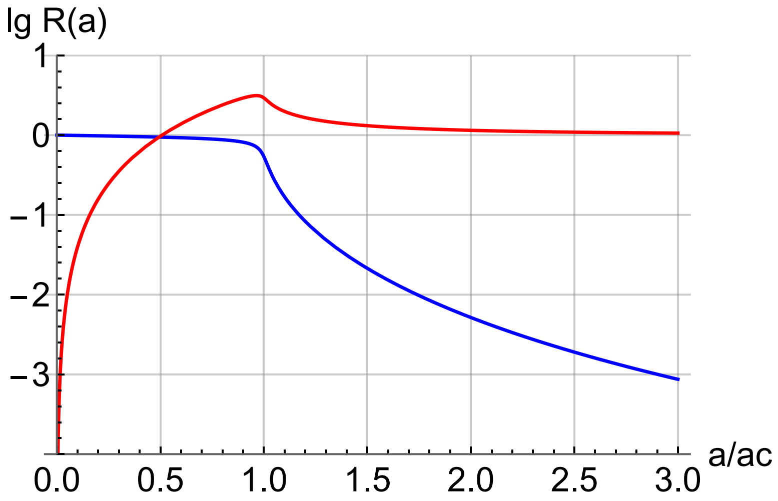

Fig. S70. Reflection coefficient $|R|^2$ and transmission coefficient $|T|^2$ as a function of the reduced incidence angle α/α_c. The transmission shows the Yoneda peak at $\alpha = \alpha_c$. The reflection coefficient quickly decays to small values with increasing angle.

As an illustration, we use typical values with $\delta = 10^{-6}$ and $\beta \sim 5 \cdot 10^{-8}$ and compare the absolute square of the Fresnel transmission $|T|^2$ and reflection coefficient $|R|^2$ in Figure S.53. We observe that already at angles $\alpha/\alpha_c > 1.7$ the Fresnel reflection coefficient is $|R|^2 \ll 0.01$, such that the error in the calculated intensities is <1% and therefore the Born Approximation (BA) can be well used. A comparison between GISAS-patterns calculated in the BA- and DWBA-approximation is provided in Fig. S54.

Mathematica code implementation for Fig. S53:

```
delta=10^(-6);
beta=5*10^(-8);
n1=1.0;
n2=1-delta;
alfac=N[ArcCos[n2]/n1];
alfai=x*alfac;
Ref=Abs[(alfai-Sqrt[alfai*alfai-alfac*alfac+2*I*beta])/(alfai+Sqrt[alfai*alfai-
alfac*alfac+2*I*beta])];
Ref2=Ref*Ref;
Tra=Abs[2*alfai/(alfai+Sqrt[alfai*alfai-alfac*alfac+2*I*beta])];
Tra2=Tra*Tra;
```

```
pl1=Plot[Log[10,Ref2], {x, 0,3}, PlotRange->{-4,1}, GridLines->{Automatic}, LabelStyle->Directive[Black,16],AxesLabel->{"a/ac","lg R(a)"}, AxesOrigin->{0,-4},TicksStyle->Directive[Black,10],PlotStyle->{Blue}] ;
pl2=Plot[Log[10,Tra2], {x, 0,3}, PlotRange->{-4,1}, GridLines->{Automatic}, LabelStyle->Directive[Black,16],AxesLabel->{"a/ac","lg R(a)"}, AxesOrigin->{0,-4},TicksStyle->Directive[Black,10],PlotStyle->{Red}] ;
Show[pl1,pl2]
```

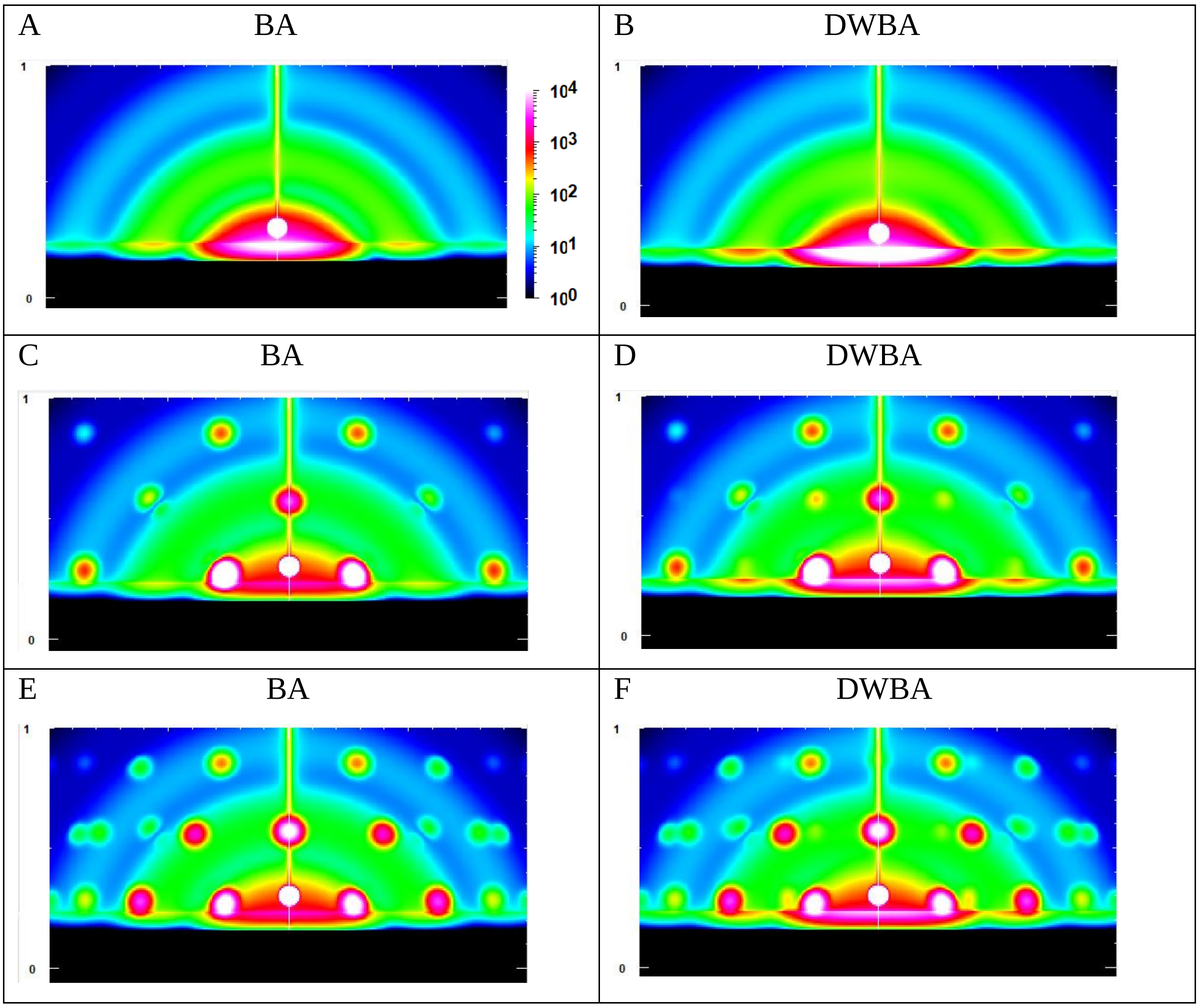

Fig. S71: Calculated GISAS-patterns for spheres (R=10, σ=0.08, top row A/B), spheres ordered in a BCC-lattice (a = 22nm, (001)||beam, middle row C/D), and spheres in BCC-lattices (a = 22nm, (001)||beam fiber pattern, lower row E/F) applying a beam with incident angle α_i = 0.15° and a horizon at $q_{z,horiz}$ = 0.15 nm^{-1} in a material with $q_{z,c}$ = 0.23 nm^{-1}, corresponding to a critical angle α_c = 0.081°. The refractive index parameters are $\delta = 10^{-6}$ and $\beta=10^{-8}$. The incident plane specular and diffuse scattering is calculated with Eq. (S.9.2) using an interfacial roughness of σ_z = 1 nm and a cut-off length of ξ =10^5. The reflected beam position is simulated at $2q_{z,horiz}$. The left column is calculated with just the first DWBA-term (Born Approximation), the right column including all four DWBA-terms to show the additional features due to the reflectivity contributions. F is well comparable to published GISAS-patterns of spherical nanoparticles ordered in BCC-superlattices [S4].

In effect, by using the decoupling approximation, the calculation of the scattered intensity $I(\boldsymbol{q})$ in the GISAS-experiment only requires multiplication with the Fresnel coefficients and thus can be computed at nearly the same speed as the transmission scattering patterns.

10. Implementation

10.1 Recursion Relations

Powers z^n, factorials $n!$, gamma functions $\Gamma(n+z)$, Pochhammer factorials $(z)_n$ and $(z)_{2n}$, and binomials can be efficiently computed via the recursion relations summarized in Table S-9.

Table S-9.

c_n	Recursion relation	Relation to preceeding coefficient	c_0
z^n	$z^n = zz^{n-1}$	zc_{n-1}	1
n!	$n! = n(n-1)!$	nc_{n-1}	1
$\Gamma(n+z)$	$\Gamma(n+z) = (n+z-1)\Gamma(n+z-1)$	$(n+z-1)c_{n-1}$	$\Gamma(z)$
$(z)_n$	$(z)_n = \frac{\Gamma(n+z)}{\Gamma(z)} = (n+z-1)\frac{\Gamma(n-1+z)}{\Gamma(z)}$	$(n+z-1)c_{n-1}$	1
$(z)_{2n}$	$(z)_{2n} = \frac{\Gamma(2n+z)}{\Gamma(z)} = \frac{(2n+z-1)(2n+z-2)\Gamma(2(n-1)+z)}{\Gamma(z)}$	$(2n+z-1)$ $(2n+z-2)c_{2(n-1)}$	1
$\frac{(x)_{2n}}{(y)_n}\frac{z^n}{n!}$	$c_n = \frac{(2n+x-1)(2n+x-2)}{(n+y-1)}\frac{z}{n}\frac{(x)_{2(n-1)}z^{n-1}}{(y)_{n-1}(n-1)!}$	$\frac{(2n-1+x)(2n-2+x)z}{(n+y-1)n}c_{n-1}$	1
$\binom{n}{m}$	$c_{n,m} = \binom{n}{m} = \left(\frac{n+1-m}{m}\right)\binom{n}{m-1}$	$\left(\frac{n+1-m}{m}\right)c_{n,m-1}$	1

Sphere: Recursive calculation of the coeffients of the sphere formfactor ($d = 3$):

$$P(q) = \sum_{n=0}^{\infty} \frac{6 \cdot 4^n}{(\mathrm{n}+3)(\mathrm{n}+2)} \frac{(z+1)_{2n}}{\left(\frac{5}{2}\right)_n n!} \left(-\frac{q^2R^2}{4(z+1)^2}\right)^n$$

$$= 6\sum_{n=0}^{\infty} \frac{1}{(\mathrm{n}+3)(\mathrm{n}+2)} \frac{(z+1)_{2n}}{\left(\frac{5}{2}\right)_n n!} \left(-\frac{R^2}{(z+1)^2}\right)^n q^{2n} = 6\sum_{n=0}^{\infty} c_n q^{2n}$$

$$c_n = \frac{1}{(\mathrm{n}+3)(\mathrm{n}+2)} \frac{(z+1)_{2n}}{\left(\frac{5}{2}\right)_n n!} \left(-\frac{R^2}{(z+1)^2}\right)^n = \frac{1}{(\mathrm{n}+3)(\mathrm{n}+2)} u_n$$

$$u_n = \frac{(z+1)_{2n}}{\left(\frac{5}{2}\right)_n n!} \left(-\frac{R^2}{(z+1)^2}\right)^n$$

$$= \frac{(2n+z)(2n+z-1)}{\left(n+\frac{3}{2}\right)n}\left(-\frac{R^2}{(z+1)^2}\right)\frac{(z+s+1)_{2(n-1)}}{\left(\frac{5}{2}\right)_{n-1}(n-1)!}\left(-\frac{R^2}{(z+1)^2}\right)^{n-1}$$

$$= \frac{(2n+z)(2n+z-1)}{\left(n+\frac{3}{2}\right)n}\left(-\frac{R^2}{(z+1)^2}\right)^{n} u_{n-1}$$

$$u_0 = 1$$

$$c_0 = \frac{1}{6}$$

As an example we show the implementation in C++:

```
rn = -R*R/((z+1)*(z+1));
un[0] = 1;
cn[0] = 1/6.0;
for ( i=1; i<=nmax; i++ )
{
      un[n] = (2*n+z)*(2*n+z-1)*rn*un[n-1]/((n+3/2.0)*n);
      cn[n] = un[n]/((n+3)*(n+2));
}
```

Disk: Recursive calculation of the coeffients of the disk formfactor ($d = 2$):

$$P_{\perp}(q) = \sum_{n=0}^{\infty} \frac{4^{n+1}\Gamma\left(n+\frac{3}{2}\right)}{\sqrt{\pi}\,\Gamma(n+3)} \frac{(z+1)_{2n}}{(2)_n n!}\left(-\frac{q^2R^2}{4(z+1)^2}\right)^{n}$$

$$= \frac{4}{\sqrt{\pi}}\sum_{n=0}^{\infty} \frac{\Gamma\left(n+\frac{3}{2}\right)}{(n+2)!}\frac{(z+1)_{2n}}{(2)_n n!}\left(-\frac{R^2}{(z+1)^2}\right)^{n} q^{2n} = \frac{4}{\sqrt{\pi}}\sum_{n=0}^{\infty} c_n q^{2n}$$

$$c_n = \frac{\Gamma\left(n+\frac{3}{2}\right)}{(n+2)!}\frac{(z+1)_{2n}}{(2)_n n!}\left(-\frac{R^2}{(z+1)^2}\right)^{n} = \frac{\left(n+\frac{1}{2}\right)(2n+z)(2n+z-1)}{(n+2)(n+1)n}\left(-\frac{R^2}{(z+1)^2}\right)c_{n-1}$$

$$c_0 = \Gamma\left(\frac{3}{2}\right)2! = \sqrt{\pi}$$

The recursive calculation does not require the calculation of gamma functions or factorials, is fast, and avoids fractions with very large denominators and enumerators which improves precision. As an example we show the implementation in C++:

```
rn = -R*R/((z+1)*(z+1));
cn[0] = sqrt(M_PI);
for ( i=1; i<=nmax; i++ )
```

```
     cn[n] = (n+1/2.0)*(2*n+z)*(2*n+z-1)*rn*cn[n-1]/((n+2)*(n+1)*n);
```

Cylinder: Recursive calculation of the coeffients of the cylinder formfactor ($d = 1$):

$$P_{\|}(q) = \sum_{n=0}^{\infty} \frac{4^n}{(\mathrm{n}+1)} \frac{(z+1)_{2n}}{\left(\frac{3}{2}\right)_n n!} \left(-\frac{q^2 L^2}{4(z+1)^2}\right)^n$$

$$= \sum_{n=0}^{\infty} \frac{1}{(\mathrm{n}+1)} \frac{(z+1)_{2n}}{\left(\frac{3}{2}\right)_n n!} \left(-\frac{L^2}{(z+1)^2}\right)^n q^{2n} = \sum_{n=0}^{\infty} c_n q^{2n}$$

$$c_n = \frac{1}{(\mathrm{n}+1)} \frac{(z+1)_{2n}}{\left(\frac{3}{2}\right)_n n!} \left(-\frac{L^2}{(z+1)^2}\right)^n = \frac{1}{(\mathrm{n}+1)} u_n$$

$$u_n = \frac{(z+1)_{2n}}{\left(\frac{3}{2}\right)_n n!} \left(-\frac{L^2}{(z+1)^2}\right)^n$$

$$= \frac{(2n+z)(2n+z-1)}{\left(n+\frac{1}{2}\right) n} \left(-\frac{L^2}{(z+1)^2}\right) \frac{(z+1)_{2(n-1)}}{\left(\frac{3}{2}\right)_{n-1} (n-1)!} \left(-\frac{L^2}{(z+1)^2}\right)^{n-1}$$

$$= \frac{(2n+z)(2n+z-1)}{\left(n+\frac{1}{2}\right) n} \left(-\frac{L^2}{(z+1)^2}\right) u_{n-1}$$

$$u_0 = 1$$

As an example we show the implementation in C++:

```
ln = -L*L/((z+1)*(z+1));
un[0] = 1;
cn[0] = 1;
for ( i=1; i<=nmax; i++ )
{
     un[n] = (2*n+z)*(2*n+z-1)*ln*un[n-1]/((n+1/2.0)*n);
     cn[n] = un[n]/(n+1);
}
```

Scattering amplitudes: We now consider the coefficients for the scattering amplitudes. We have

$$\langle F^{(d)}(q) \rangle^2 = \sum_{n=0}^{\infty} \left(-\frac{q^2 R^2}{4(z+1)^2}\right)^n f_n^{(d)} = \sum_{n=0}^{\infty} c_n q^{2n}$$

$$c_n = \left(-\frac{q^2R^2}{4(z+1)^2}\right)^n f_n^{(d)} = \left(-\frac{q^2R^2}{4(z+1)^2}\right)^n \sum_{m=0}^{n} \frac{(z+1)_{2(n-m)}(z+1)_{2m}}{\left(\frac{d+2}{2}\right)_{n-m}\left(\frac{d+2}{2}\right)_m (n-m)!\,m!} =$$

We consider the coefficients

$$u_m = \frac{(z+1)_{2m}}{\left(\frac{d+2}{2}\right)_m m!} = \frac{(2m+z)(2m+z-1)}{\left(m+\frac{d}{2}\right)m} \frac{(z+1)_{2(m-1)}}{\left(\frac{d+2}{2}\right)_{m-1}(m-1)!}$$
$$= \frac{(2m+z)(2m+z-1)}{\left(m+\frac{d}{2}\right)m} u_{m-1}$$

$$u_0 = 1$$

$$v_n = \left(-\frac{q^2R^2}{4(z+1)^2}\right)^n = \left(-\frac{q^2R^2}{4(z+1)^2}\right) v_{n-1}$$

$$v_0 = 1$$

Then we have

$$c_n = v_n \sum_{m=0}^{n} u_{n-m} u_m$$

Biaxial Ellipsoid. For the biaxial ellipsoid we have to compute the coefficient

$$e_n^{(\theta)} = \sum_{m=0}^{n} \binom{n}{m} \frac{(\varepsilon^2-1)^m}{(2m+1)}$$

We introduce $u_{n,m}$ and use the recursion relation for the binomial coefficient:

$$u_{n,m} = \binom{n}{m}(\varepsilon^2-1)^m = \left(\frac{n+1-m}{m}\right)(\varepsilon^2-1)\binom{n}{m-1}(\varepsilon^2-1)^{m-1}$$
$$= \left(\frac{n+1-m}{m}\right)(\varepsilon^2-1)u_{n,m-1}$$

$$u_{n,0} = 1$$

Then the sum to evaluate is

$$e_n^{(\theta)} = \sum_{m=0}^{n} \frac{u_{n,m}}{(2m+1)}$$

Superellipsoid. For the formfactor of the superellipsoid we have to evaluate the coefficients

$$c_{n,m} = \sum_{n'=0}^{n} \frac{\Gamma\left(\frac{2(n-n')+1}{k}\right)}{\left(\frac{1}{2}\right)_{n-n'}(n-n')!} \frac{\Gamma\left(\frac{2n'+1}{k}\right)}{\left(\frac{1}{2}\right)_{n'} n'!} \sum_{m'=0}^{m} \frac{\Gamma\left(\frac{2(m-m')+2+k}{k}\right)}{(2)_{m-m'}(m-m')!} \frac{\Gamma\left(\frac{2m'+2+k}{k}\right)}{(2)_{m'}m'!} \frac{1}{\Gamma\left(\frac{2(m-m')+2(n-n')+k+3}{k}\right)\Gamma\left(\frac{2m'+2n'+k+3}{k}\right)}$$

Since there is no recursion relation for the gamma functions $\Gamma\left(\frac{2n+1}{k}\right)$, $\Gamma\left(\frac{2n+k+2}{k}\right)$, and $\Gamma\left(\frac{2n+k+3}{k}\right)$ we once evaluate and store the latter for a given value of k in the coefficients

$$g_n = \Gamma\left(\frac{2n+k+3}{k}\right)$$

We then introduce the recursively defined coefficient

$$u_{1,n} = \frac{1}{\left(\frac{1}{2}\right)_n n!} = \frac{1}{\left(n-\frac{1}{2}\right)n} u_{n-1}$$

$$u_0 = 1$$

$$u_{2,n} = \frac{1}{(2)_n n!} = \frac{1}{(n+1)n} u_{2,n-1}$$

and store the coefficients

$$v_{1,n} = \Gamma\left(\frac{2n+1}{k}\right) u_{1,n}$$

$$v_{2,n} = \Gamma\left(\frac{2n+2+k}{k}\right) u_{2.n}$$

Then the coefficients $c_{n,m}$ can be efficiently evaluated as

$$c_{n,m} = \sum_{n'=0}^{n} v_{1,n-n'}\, v_{1,n'} \sum_{m'=0}^{m} \frac{v_{2,m-m'}\, v_{2,m'}}{g_{n'+m'} g_{n-n'+m-m'}}$$

Implementation in C++:

```
u[0] = 1;
for ( n=1; n<=max; n++ )
{
   u1[n] = u1[n-1]/((n-1/2.0)*n);
   u2[n] = u2[n-1]/((n+1)*n);
}
for ( n=0; n<=max; n++ )
{
   v1[n] = gamma((2*n+1)/k)*u1[n];
   v2[n] = gamma((2*n+2+k)/k)*u2[n];
}
for ( n=0; n<=2*max; n++ ) g[n] = gamma((2*n+k+3)/k);
for ( n=0; n<=max; n++ )
```

```
{
   for ( m=0; m<=max; m++ )
   {
      sum1 = 0.0;
      for ( ns=0; ns<=n; ns++ )
      {
         sum2 = 0.0;
         for ( ms=0; ms<=m; ms++ ) sum2 = sum2+v[m-ms]*v[ms]/(g[ns+ms]*g[n-ns+m-ms]);
         sum1 = sum1+v[n-ns]*v[ns]*sum2;
      }   /* ns */
      cnm[n][m] = sum1;
   }  /* m */
}  /*  n  */
```

Superball. For the formfactor of the superball we have to evaluate the coefficients

$$c_{n,m,k} = \sum_{n'=0}^{n} \frac{\Gamma\left(\frac{2(n-n')+1}{p}\right)\Gamma\left(\frac{2n'+1}{p}\right)}{\left(\frac{1}{2}\right)_{n-n'}\left(\frac{1}{2}\right)_{n'}(n-n')!\,(n')!} \sum_{m'}^{m} \frac{\Gamma\left(\frac{2(m-m')+1}{p}\right)\Gamma\left(\frac{2m'+1}{p}\right)}{\left(\frac{1}{2}\right)_{m-m'}\left(\frac{1}{2}\right)_{m'}(m-m')!\,(m')!} \sum_{k'}^{k} \frac{\Gamma\left(\frac{2(k-k')+1}{p}\right)\Gamma\left(\frac{2k'+1}{p}\right)}{\left(\frac{1}{2}\right)_{k-k'}\left(\frac{1}{2}\right)_{k'}(k-k')!\,(k')!} \frac{1}{\Gamma\left(\frac{2(n-n')+2(m-m')+2(k-k')+3}{p}+1\right)} \frac{1}{\Gamma\left(\frac{2n'+2m'+2k'+3}{p}+1\right)}$$

Since there is no recursion relation for the gamma functions $\Gamma\left(\frac{2n+1}{p}\right)$ and $\Gamma\left(\frac{2n+3}{p}+1\right)$ we once evaluate and store them for the latter for a given value of p in the coefficients

$$g_n = \Gamma\left(\frac{2n+3}{p}+1\right)$$

We then introduce the recursively defined coefficient

$$u_n = \frac{1}{\left(\frac{1}{2}\right)_n n!} = \frac{1}{\left(n-\frac{1}{2}\right)n} u_{n-1}$$

$$u_0 = 1$$

and store the coefficients

$$v_n = \Gamma\left(\frac{2n+1}{p}\right)u_n$$

Then the coefficients $c_{n,m,k}$ can be efficiently evaluated as

$$c_{n,m,k} = \sum_{n'=0}^{n} v_{n-n'}\, v_{n'} \sum_{m'=0}^{m} v_{m-m'}\, v_{m'} \sum_{k'=0}^{k} \frac{v_{k-k'}\, v_{k'}}{g_{n'+m'+k'}\, g_{n-n'+m-m'+k-k'}}$$

where we can exploit the symmetry $c_{n,m,k} = c_{m,n,k}$

Implementation in C++:

```
u[0] = 1;
for ( n=1; n<=max; n++ ) u[n] = u[n-1]/((n-1/2.0)*n);
for ( n=0; n<=max; n++ ) v[n] = gamma((2*n+1)/p)*u[n];
for ( n=0; n<=3*max; n++ ) g[n] = gamma(1+(2*n+3)/p);
for ( n=0; n<=max; n++ )
{
   for ( m=0; m<=n; m++ )
   {
      for ( k=0; k<=max; k++ )
      {
         sumns = 0.0;
         for ( ns=0; ns<=n; ns++ )
         {
            summs = 0.0;
            for ( ms=0; ms<=m; ms++ )
            {
               sumks = 0.0;
               for ( ks=0; ks<=k; ks++ )
                  sumks = sumks+v[k-ks]*v[ks]/(g[ns+ms+ks]*g[n-ns+m-ms+k-ks]);
               summs = summs+v[m-ms]*v[ms]*sumks;
            }
            sumns = sumns+v[n-ns]*v[ns]*summs;
         }
         cnmk[n][m][k] = sumns;
         cnmk[m][n][k] = sumns;
      }
   }
}
```

10.2 GPU Implementation

The algorithm can use an installed GPU to accelerate the calculation of the pixel intensities. After addressing the relevant technical features of CPUs and GPUs, the actual implementation is described.

Each CPU consists of multiple (e.g. 16) cores, each of which can run one thread to calculate pixel intensities. With the modern Hyper-Threading Technology, two threads on each core are possible.

Each GPU contains more than 1000 specialized cores, allowing to run more than 1000 threads simultanously. These cores have separate calculation registers for single floating point and double precision numbers. Addionally they have a special part for matrix multiplication used by tensor calculations in AI algorithms. All threads execute the same code, but can take different paths in the program flow.

To implement the usage of the GPU, the CUDA library is used. Therefore, the C++ codepart for the calculations is compiled with a special compiler (nvcc) to generate a kernel program which is copied to the GPU at the first launch of the kernel inside the main program. This is embedded in normal Windows/Linux code.

The threads inside a GPU are grouped into blocks. Multiple blocks are logically part of a grid. At kernel launch the programmer informs the GPU about the number of blocks which should be used, and how many threads in each block should be started. This is necessary, because GPU memory limits require to adapt the threads according to the size of the coefficient arrays.

The numbers of blocks and threads can be given in grid dimensions (x,y,z). In the algorithm a 2D matrix is calculated, where the two dimensions (i,j) correspond to the first two dimensions (x,y) of thre grid. Inside the kernel, variables containing the current and maximum indices for each dimension can be accessed and written into the destination matrix. If a thread is finished, the next thread of the destination is automatically started, thereby in effect looping over all pixels.

If no GPU is found at program start, the algorithm simulates the two loops over the dimensions (x,y) calling the same routine. For accelerated computation this is done with a maximum number of threads set by the user, but limited by the number of cores of the CPU.

For the GPU-calculations, an NVIDIA GeForce RTX 2070 Super graphic card with an Intel Core i5-8500 (3GHz, 6 cores) CPU was used.

11. Benchmark CPU-times

For the numerical integrations we used an extended trapezoidal rule algorithm which converges fast for integrals over functions of intermediate smoothness such as in the present cases [S3]. Gauss-Legendre integration algorithms required longer integration times to reach the required precision. In all cases, the cosine-functions in the ellipsoid integrals were substituted by a linear function for accelerated numerical integration (Eq. S.4.2.1.2).

11.1 CPU times for polydisperse particles

For benchmarking we compared the CPU times for the calculation of the formfactors of polydisperse particles (spheres, biaxial ellipsoids, triaxial ellipsoids, cylinders, disks, cubes, supercubes) using series expansions and numerical integrations. The CPU times are provided in Table S-10.

Table S-10

	**Sphere**		**Biaxial Ellipsoid**		**Triaxial Ellipsoid**		**Cylinder**		**Disk**		**Cube**	
Size/nm	R=2		a=b=1, c=3		a=1, b=2, c=3		R=1, L=8		R=8, L=1		a=1	
Data points	Series ms	Integral ms	Series ms	Integral ms	Series ms	Integral ms	Series ms	Integral Ms	Series ms	Integral ms	Series ms	Integral ms
49	0.3	1.3	4.2	93.9	73	18707	1.5	1222.5	1.1	10350.9	5.7	13606.3
81	0.4	2	4.1	120.2	78	22225	1.5	1723.5	1.1	17437.9	5.9	27565.1
121	0.5	3.1	4.9	172.3	101	25920	1.5	2607.5	1.2	26399.8	6.1	30104.9
169	0.5	4.5	5.6	231	115	30011	1.5	3096.5	1.3	35986.7	5.9	43686.1
225	0.5	5.5	7.1	289.3	121	36243	1.5	4624.5	1.4	49542.6	6	55182
289	0.6	6.5	7.6	350.8	159	44991	1.6	6957.4	1.4	60080.6	6.1	82153.9
361	0.7	8.4	9.8	439.2	182	54811	1.7	8013.3	1.5	78206.5	6.2	87645.8
441	0.7	10.3	10.5	537.5	206	67000	1.8	9945.2	1.6	101824.4	6.2	108900.8
529	1	12.2	12.1	640.2	240	72680	1.9	12054.1	1.7	114587.3	6.2	146041.8
625	1	15	13.8	736.7	248	86192	2	14933	2.4	134543.6	6.3	159561.7
1681	1.8	38.5	33.4	1912	605	198193	2.9	31790.1	2.5	392525.5	7.2	406933.8
3721	3.2	87	69.3	5015	1328	471971	4.2	76623.8	3.7	873588.3	8.4	996330.6
14641	10.8	338.6	265.4	16463	4977	1710612	11	297160	9.6	3235610.4	14.6	3810447.4
58081	36.2	1327	1040.3	70552	19536	6715952	35	1081330	28.8	1.4557E7	35.8	18891762.2
103041	65	2520	1966.2	127696.8	35526	1.19305E7	64	1899361	55.8	2.35787E7	60.7	30924330.3

Calculation Parameters:

- relative standard deviation of size distributions: σ=0.1
- max. q-value: $q_{max}=6\ nm^{-1}$
- relative precision: 10^{-4}

For superball we used a=2 nm, b=2.5 nm, c=3 nm, k=5.5. The average CPU time was 2550 ms. The calculations were done on a simple consumer notebook with an Intel Core i5-8265U 3.9 GHz CPU using a single core.

11.2 CPU times for different precisions

For the calculations in Table S-10 the expansions and numerical integrations were performed with a relative precision of 10^{-4}. For the case of polydisperse spheres and polydisperse biaxial ellipsoids we investigated the effect of varying precision on the CPU times, which are summarized in Table S-11 and visualized in Fig. S-72.

Table S-11

	Sphere		Biaxial Ellipsoid	
Size/nm	R=2<br>103041 points		a=b=1, c=3<br>1681 points	
Relative precision	Series ms	Integral ms	Series ms	Integral ms
1E-02	59	2193	16	736
1E-03	60	2376	16	963
1E-04	60	2410	25	2042
1E-05	60	2496	44	6830
1E-06	60	2820	53	18661
1E-07	60	3430	87	69623

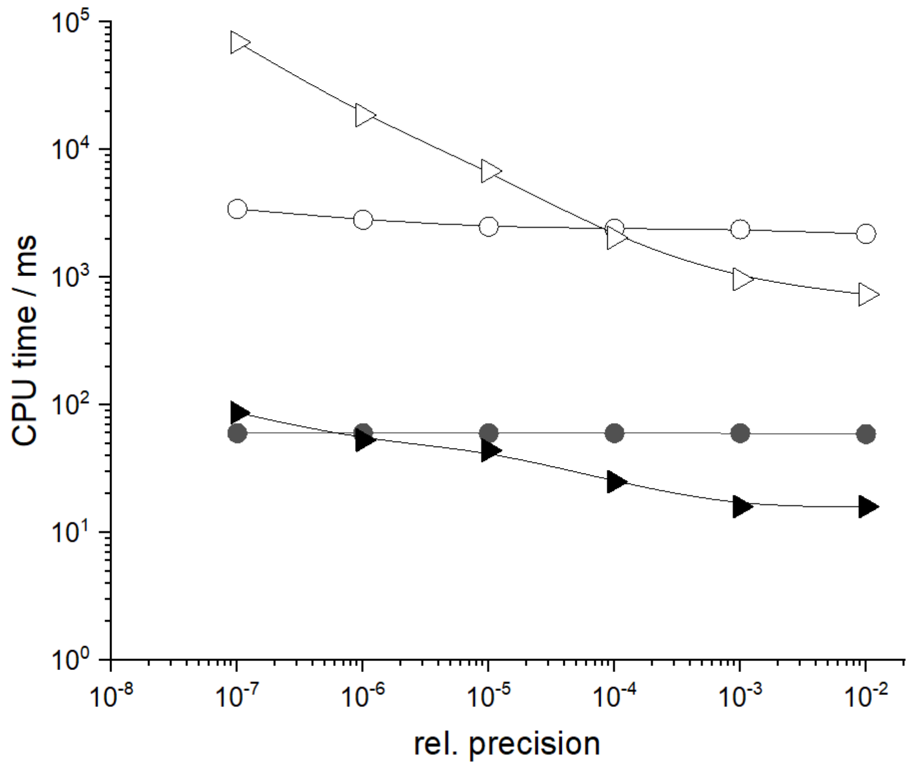

Fig. S72: CPU time to calculate the formfactor of polydisperse spheres by series expansions (●) and numerical integration (○), and of polydisperse biaxial ellipsoids by series expansion (▶) and numerical integration (▷), for different relative precisions. The size parameters are the same as for Table S-10. We observe that the series expansions converge rapidly, such that variations in the relative precision have only minor effects on the CPU time, compared to numerical integrations, where a variation of the relative precision can change CPU times by two orders of magnitude.

11.3 CPU times for analytical formfactor expressions

For the comparisons in Sections 11.1 and 11.2 we considered the formfactors of polydisperse particles, where numerical integrations at least over the size distributions are required. In the following we provide a more challenging test, where we compare the performance of the algorithm in comparison to analytical expressions for the formfactors of monodisperse spheres and biaxial ellipsoids.

Sphere

The formfactor of monodisperse spheres with radius *R* is given by **S.1.2.45** (Table S-4).

$$P(q) = 9\left(\frac{\sin(qR) - qR\cos(qR)}{(qR)^3}\right)^2$$

For the series expansion we have S.1.2.46 (Table S-4).

$$P(q) = 6\sum_{n=0}^{\infty}\frac{4^n}{(\mathrm{n}+3)(\mathrm{n}+2)}\frac{1}{\left(\frac{5}{2}\right)_n n!}\left(-\frac{q^2R^2}{4}\right)^n$$

$$= 6\sum_{n=0}^{\infty}\frac{1}{(\mathrm{n}+3)(\mathrm{n}+2)}\frac{1}{\left(\frac{5}{2}\right)_n n!}(-q^2R^2)^n$$

For the coefficients

$$u_n = \frac{1}{\left(\frac{5}{2}\right)_n n!}(-R^2)^n$$

we apply the recursion relation

$$u_n = \frac{1}{\left(n+\frac{3}{2}\right)n}(-R^2)u_{n-1}$$

$$u_0 = 1$$

to obtain the coefficients

$$c_n = \frac{u_n}{(n+3)(n+2)}$$

such that the formfactor is computed as **(S.11.3.1)**

$$P(q) = 6\sum_{n=0}^{\infty}c_n q^{2n}$$

The asymptotic expansion is identical to the analytical expansion S.1.2.47 (Table S-4). The formfactors for the analytical expression and the series expansion are shown in Fig. S73. The CPU times are provided in Table S-12.

Mathematica code implementation:

```
R=1;
nmax=60;
cns=RecurrenceTable[{u[n+1]==u[n]*(-R*R)/((n+3/2)*n), u[1]==1},u,{n,1,nmax+1}];

q=10^lq;
lim=1.5;
Pqs=9*((Sin[q*R]-q*R*Cos[q*R])/(q*q*q*R*R*R))^2;
Pqssa=6*Sum[(cns[[n+1]]/((n+3)*(n+2)))*((q*q)^n), {n, 0, nmax}];
pl1=Plot[Log[10,Pqs],{lq,-1,lim},PlotRange->{-8,1},GridLines->{Automatic},LabelStyle->Directive[Black,16],AxesLabel->{"log q","lg P(q)"},AxesOrigin->{-1,-8},TicksStyle->Directive[Black,12],PlotStyle->{Black}];
pl2=Plot[Log[10,Pqssa],{lq,-1,lim},PlotRange->{-8,1},GridLines->{Automatic},LabelStyle->Directive[Black,16],AxesLabel->{"log q","lg P(q)"},AxesOrigin->{-1,-8},TicksStyle->Directive[Black,12],PlotStyle->{Blue,dashed}];
Show[pl1,pl2]
```

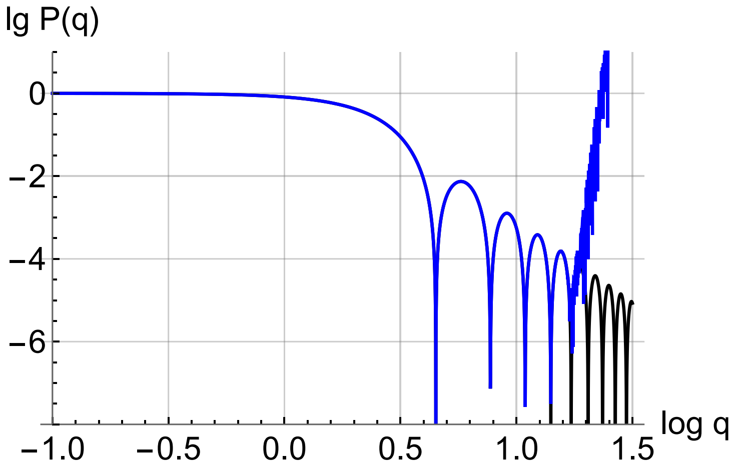

Fig. S73: Formfactor of monodisperse spheres calculated using the analytical expression (black line, S.1.2.45) and the series expansion (blue line, S.11.3.1). To compute the complete formfactor in the latter case, the analytical expressions is used for the high-*q* region.

Biaxial ellipsoid

The formfactor of isotropic monodisperse biaxial ellipsoids with semiaxis *a* and *b* is given by **(S.4.2.1.1)**

$$P(q) = 9 \int_0^{\pi/2} \frac{\left(\sin\left(qR(\theta)\right) - qR(\theta)\cos\left(qR(\theta)\right)\right)^2}{\left(qR(\theta)\right)^6} \sin\theta \, d\theta$$

$$R(\theta) = \sqrt{(a\sin\theta)^2 + (c\cos\theta)^2}$$

To accelerate the integration we can use the ratio $\varepsilon = a/b$ and substitute

$$P(q) = 9\int_0^1 \frac{\left(\sin\left(qR(x)\right) - qR(x)\cos\left(qR(x)\right)\right)^2}{\left(qR(x)\right)^6}dx$$

$$R(x) = a(1 + (\varepsilon^2 - 1)x^2)^{1/2}$$

For the series expansion we obtain from (S.4.3.1.3)

$$P(q) = 6\sum_{n=0}^{\infty} \frac{1}{(\mathrm{n}+3)(\mathrm{n}+2)} \frac{1}{\left(\frac{5}{2}\right)_n n!}(-a^2q^2)^n \sum_{m=0}^{n} \binom{n}{m} \frac{(\varepsilon^2-1)^m}{(2m+1)}$$

For the coefficient in the second sum we have the recursion relation

$$u_{n,m} = \binom{n}{m}(\varepsilon^2 - 1)^m = \left(\frac{n+1-m}{m}\right)(\varepsilon^2 - 1)u_{n,m-1}$$

$$u_{n,0} = 1$$

from which the second sum can be evaluated as

$$e_n = \sum_{m=0}^{n} \frac{u_{n,m}}{(2m+1)}$$

For the coefficient in the first sum we have the recursion relation

$$v_n = \frac{1}{\left(\frac{5}{2}\right)_n n!}(-a^2)^n = \frac{1}{\left(n+\frac{3}{2}\right)n}(-a^2)u_{n-1}$$

$$v_0 = 1$$

from which the overall coefficient including both sums can be obtained as

$$c_n = \frac{v_n e_n}{(\mathrm{n}+3)(\mathrm{n}+2)}$$

such that the formfactor can be calculated as **(S.11.3.2)**

$$P(q) = 6\sum_{n=0}^{\infty} c_n q^{2n}$$

The asymptotic expansion is identical to the analytical expansion. The formfactors for the analytical expression and the series expansion are shown in Fig. S74. The CPU times are provided in Table S-12.

Mathematica code implementation:

```
a=1;
b=2;
eps=b/a;
nmax=100;
cns=RecurrenceTable[{u[n+1]== u[n]*(-a*a)/((n+3/2)*n),u[1]==1},u,{n,1,nmax+1}];
cnt=Table[RecurrenceTable[{u[m+1]== u[m]*(n+1-m)*(eps*eps-1)/m,u[1]==1},u,{m,1,nmax+1}], {n, 0, nmax}];
ce=Table[Sum[cnt[[n+1,m+1]]/(2*m+1), {m, 0, n}], {n, 0, nmax}];
cc=Table[cns[[n+1]]*ce[[n+1]]/((n+3)*(n+2)), {n, 0, nmax}];

q=10^lq;
lim=1.5;
rx=a*Sqrt[1+(eps*eps-1)*x*x];
Pqe=9*NIntegrate[((Sin[q*rx]-q*rx*Cos[q*rx])/(q*q*q*rx*rx*rx))^2, {x, 0, 1}];
Pqesa=6*Sum[cc[[n+1]]*((q*q)^n), {n, 0, nmax}];
pl1=Plot[Log[10,Pqe],{lq,-1,lim},PlotRange->{-8,1},GridLines->{Automatic},LabelStyle-
>Directive[Black,16],AxesLabel->{"log q","lg P(q)"},AxesOrigin->{-1,-8},TicksStyle->Directive[Black,12],PlotStyle-
>{Black}];
pl2=Plot[Log[10,Pqesa],{lq,-1,lim},PlotRange->{-8,1},GridLines->{Automatic},LabelStyle-
>Directive[Black,16],AxesLabel->{"log q","lg P(q)"},AxesOrigin->{-1,-8},TicksStyle->Directive[Black,12],PlotStyle-
>{Blue,dashed}];
Show[pl1,pl2]
```

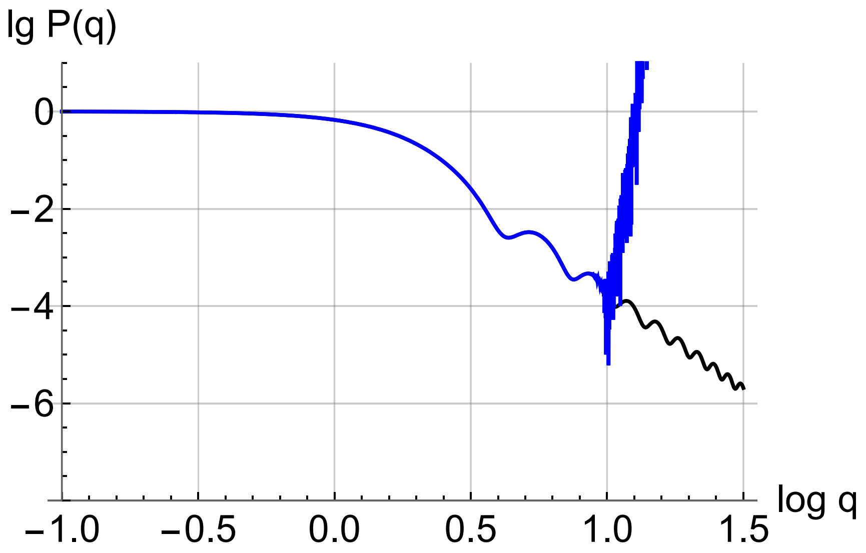

Fig. S74: Formfactor of monodisperse biaxial ellipsoids calculated using the analytical expression (black line, S.4.2.1.1) and the series expansion (blue line, S.11.3.2). To compute the complete formfactor in the latter case, the analytical expressions is used for the high-q region.

Table S-12

	Sphere		Biaxial Ellipsoid	
Size/nm	R=2		a=b=1, c=3	
Data points	Series ms	Integral ms	Series ms	Integral ms
49	0.17	0.15	0.75	54
81	0.22	0.15	0.85	54
121	0.22	0.15	0.95	54
169	0.22	0.2	1.25	54
225	0.22	0.2	1.35	54
289	0.22	0.2	1.55	54
361	0.32	0.2	1.85	54
441	0.32	0.2	2.15	54
529	0.32	0.3	2.45	54
625	0.32	0.3	2.95	55
1681	0.62	0.5	6.95	59
2601	0.92	0.8	10.25	64
3721	1.02	1	14.75	69
14641	4.02	3	55.25	114
58081	15.02	12	215.25	298
103041	30.02	25	386.25	494

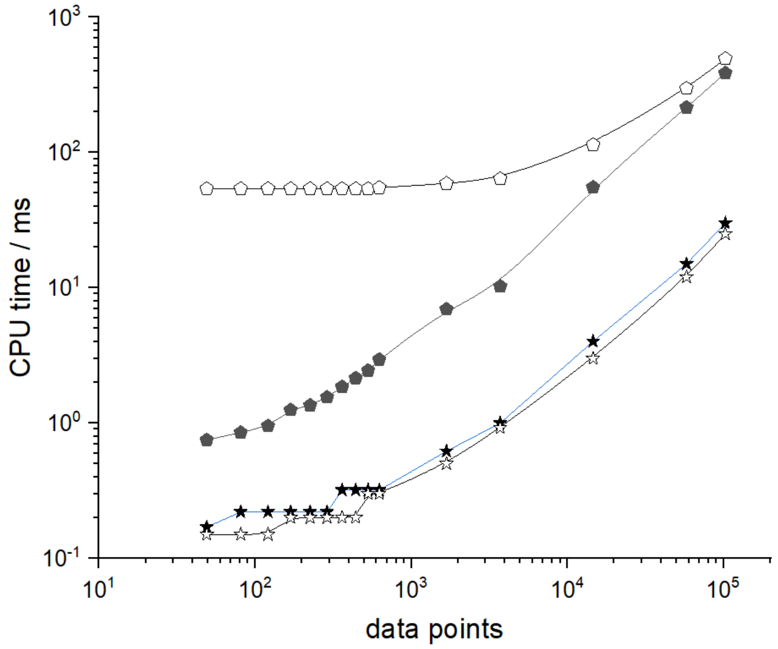

Fig. S75: CPU times to compute the formfactor of monodisperse spheres (star symbol) and biaxial ellipsoids (pentagon symbol) using the series expansions (★, ⬟) and the analytical expressions (☆,⬠).

We observe from Table S-12 and Fig. S75, that – as expected – the CPU times for the simple analytical expression for monodisperse spheres (S.1.2.45) are always smaller compared to the series expansions (S.11.3.1). However, the difference is only ca. 20%. Already for the biaxial ellipsoid the series expansion (S.11.3.2) is always faster compared to the analytical expression S.4.2.1.1.

12. Applications

12.1 X-ray, neutron, light, electron scattering applications

Small-angle X-ray scattering (SAXS)

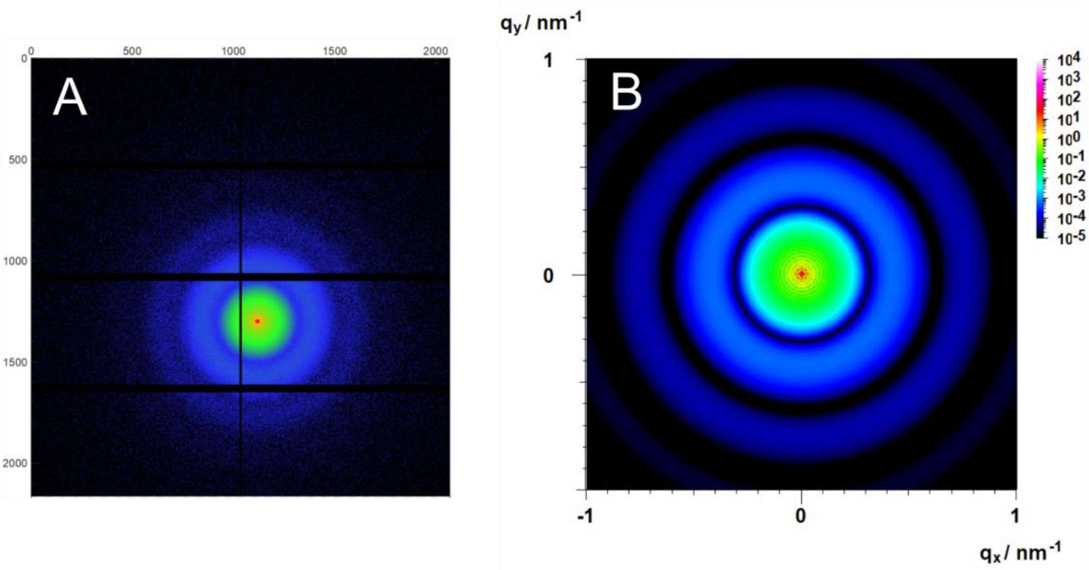

Figure S76: Small-angle X-ray scattering (SAXS) patterns of monodisperse hollow silica partices in water showing two formfactor oscillations with superimposed fine hiqh-frequency oscillations. A: Experiment (Detector: Dectris Eiger2R 4M). B: Calculation; SAXS data courtesy of Baohu Wu.

Small-angle neutron scattering (SANS)

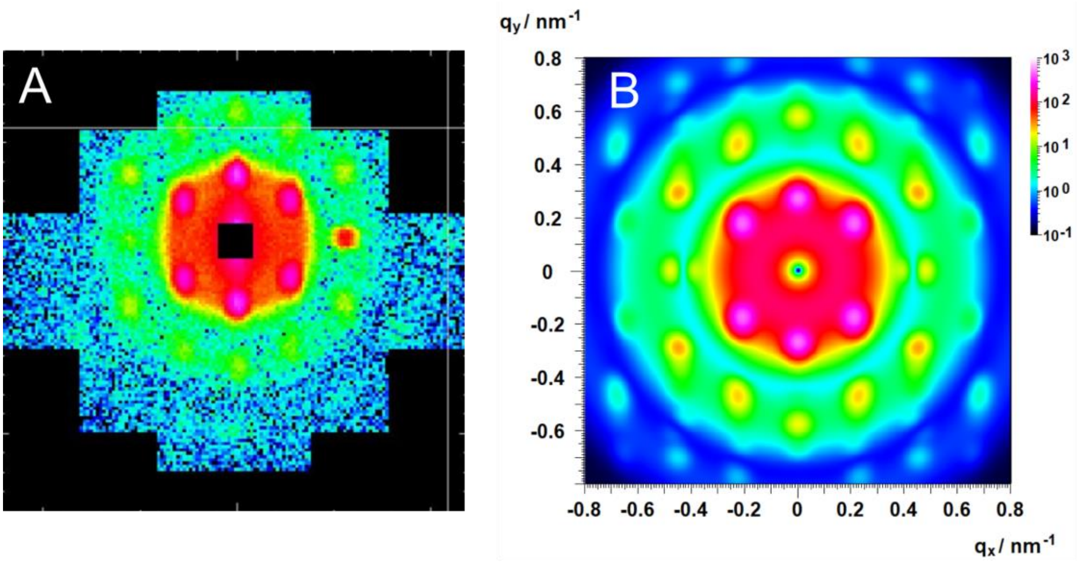

Figure S77: Small-angle neutron scattering (SANS) patterns of a BCT superlattice of Fe_3O_4 nanoparticles in an external magnetic field at 3 Tesla: A: Experiment (Beamline KWS-1, MLZ, ^{3}He 144x144 detector), B: Calculation; SANS data courtesy of Artem Feoktystov, Lisa Fruhner, Artur Feld, and Agnes Weimer.

Small-angle light scattering (SALS)

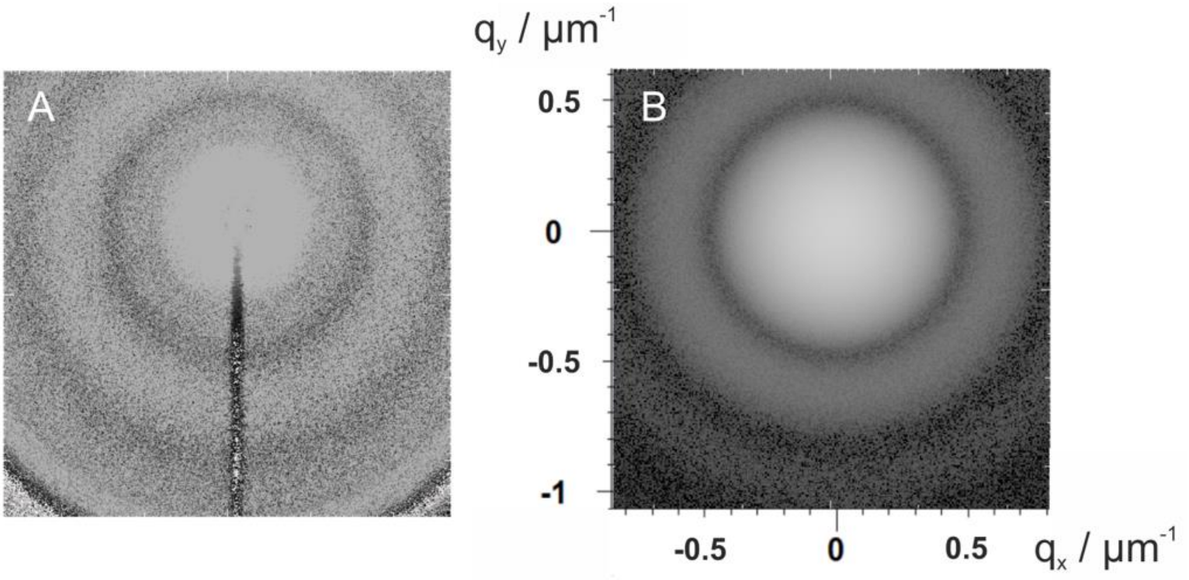

Figure S78: Small-angle laser light scattering (SALS) patterns of 10 µm monodisperse latex particles in water. Left: Experiment (Basler 2k CMOS camera acA2040-90um, 2048 x 2048 pixels), Right: Calculation; SALS data courtesy of Jörg Stellbrink and Stephan Hauschild.

Selected Area Electron Diffraction (SAED)

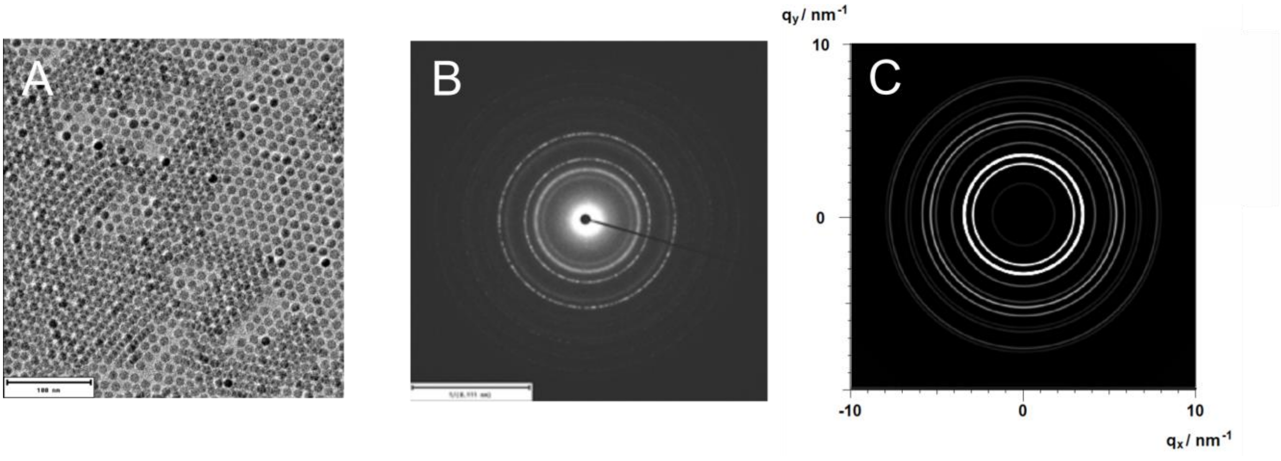

Figure S79: Selected area electron diffraction image (SAED) of monodisperse Fe_3O_4 nanoparticles: A: TEM-image; B: Experiment (JEOL JEM F200, TVIPS TemCam-XF416(ES) 4k x 4k camera), C: Calculation; SAED data courtesy of Sascha Ehlert

12.2 2D-Fitting

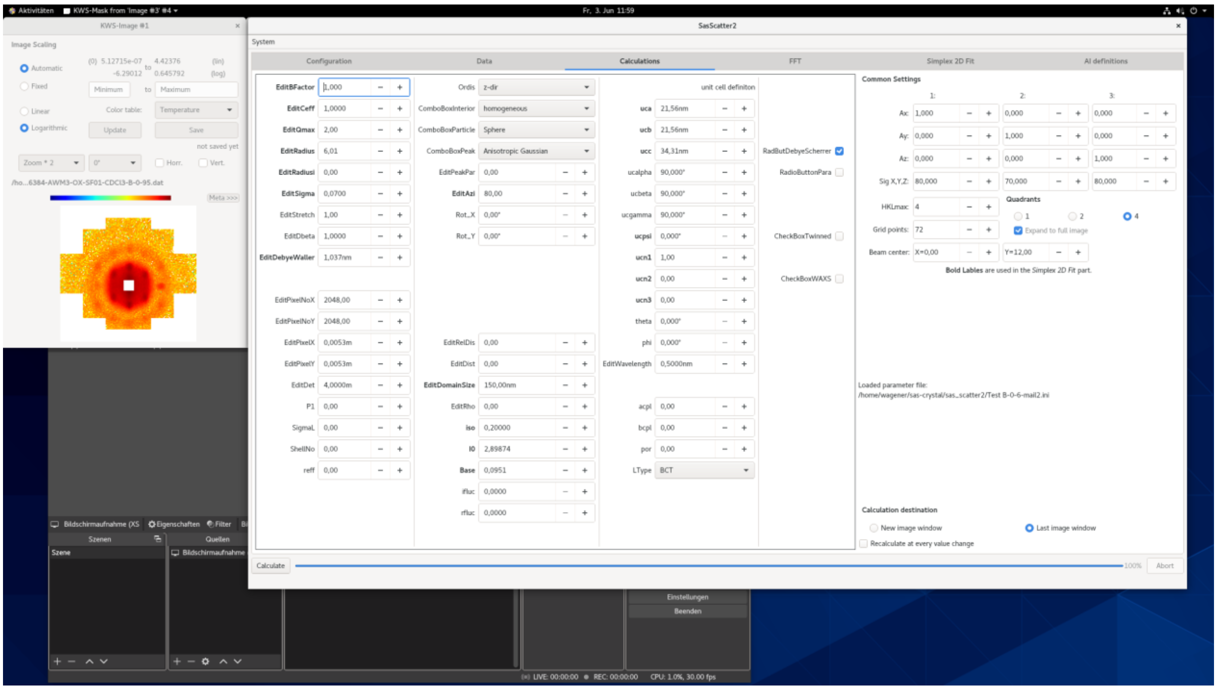

Figure S80: Screenshot of a video demonstrating 2D-fitting of the experimental 2D-SANS-data shown in Fig. S73. The video is attached as Supporting Information.

We provide a video demonstrating GPU-accelerated fitting of an experimental 2D-SANS-pattern of ordered nanparticles (Fig. S80) measured at the small-angle beamline KWS-1 at MLZ, Garching. The 144 x 144 pixel detector data set is fitted in 4.565 s with 593 iterations where the 2D-patterns are calculated.

12.3 Trainig data for neural networks

The fast algorithm enables generating simulated training data for 2D scattering pattern classification by neural networks. We show an example of the calculated scattering pattern of polydisperse spheres which was generated with Poisson noise, blind detector lines, and a simulated beam stop with an off-center beam position.

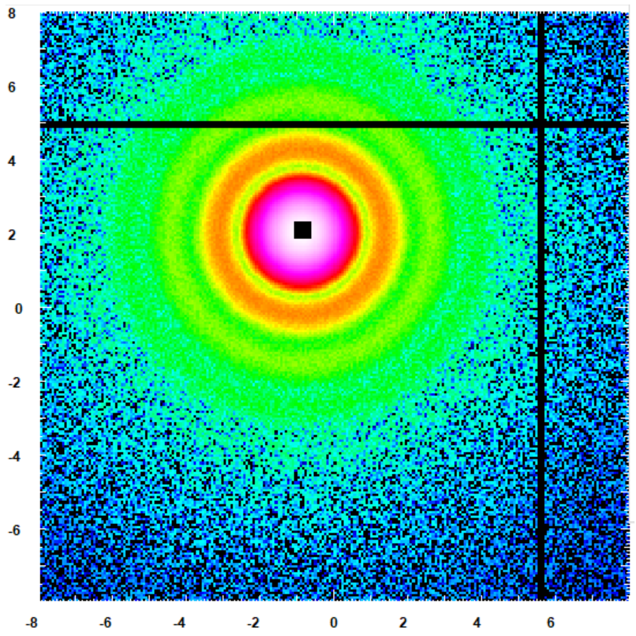

Figure S81: Simulated training data for neural networks for polydisperse spheres including Poisson random noise, an off-center primary beam position, a rectangular beam stop, and blind detector lines.

The significant saving in computation time of factors of 10^5 – 10^7 also improves the IT energy efficiency, as CPUs consume up to 50 – 100 W.

13. References

[S1] Heck, A. T., Asymptotic formulas for large arguments of hypergeometric-type functions using the Barnes integral, Ph.D. Thesis, University of Central Florida, 2004.

[S2] Förster, S., Fischer, S., Zielske, K., Schellbach, C., Sztucki, M., Lindner, P. & Perlich J. Calculation of scattering patterns of ordered nano- and mesoscale materials, Adv. Coll. Interface Sci. 163, 53-83 (2011).

[S3] Press, W. H., Flannery, B. P., Teukolsky, S. A & Vetterling, W. T. Numerical Recipies in Pascal, Cambridge University Press, pp. 125, 1990.

[S4] Weidman, M. C., Smilgies, D.-M. & Tisdale, W. A. Kinetics of the self-assembly of nanocrystal superlattices measured by in situ X-ray scattering, Nat. Mater. 15, 775 (2016).
